# Stereoselectivity control in Rh-catalyzed *β*-OH elimination for chiral allene formation

**DOI:** 10.1038/s41467-023-42660-1

**Published:** 2023-11-16

**Authors:** Jie Wang, Wei-Feng Zheng, Xue Zhang, Hui Qian, Shengming Ma

**Affiliations:** 1 Research Center for Molecular Recognition and Synthesis, Department of Chemistry, Fudan University, Shanghai, 200433 PR China; 2 State Key Laboratory of Organometallic Chemistry, Shanghai Institute of Organic Chemistry, Chinese Academy of Sciences, Shanghai, 200032 PR China

**Keywords:** Homogeneous catalysis, Stereochemistry, Reaction mechanisms

## Abstract

Stereoselectivity control and understanding in the metal-catalyzed reactions are fundamental issues in catalysis. Here we report sterically controlled rhodium-catalyzed S_N_2’-type substitution reactions of optically active tertiary propargylic alcohols with arylmetallic species affording the non-readily available enantioenriched tetrasubstituted allenes via either exclusive *syn*- or *anti*-*β*-OH elimination, respectively, under two sets of different reaction parameters. Detailed mechanistic experiments and density functional theory (DFT) studies reveal that the exclusive *anti*-Rh(I)-OH elimination is dictated by the simultaneous aid of in situ generated boric acid and ambient water, which act as the shuttle in the hydroxy relay to facilitate the Rh(I)-OH elimination process via a unique ten-membered cyclic transition state (*anti*-**TS2_u**). By contrast, the *syn*-Rh(III)-OH elimination in C–H bond activation-based allenylation reaction is controlled by a four-membered cyclic transition state (*syn-***TS3**) due to the steric surroundings around the Rh(III) center preventing the approach of the other assisting molecules. Under the guidance of these mechanistic understandings, a stereodivergent protocol to construct the enantiomer of optically active tetrasubstituted allenes from the same starting materials is successfully developed.

## Abstract (web summary)

The chiral allene formation reactions catalyzed by palladium occur via a *syn* elimination mechanism, but there is a lack in understanding of the stereoselectivity in rhodium catalyzed reactions, specifically the *β*-OH elimination step. Here, the authors report the sterically controlled Rh-catalyzed S_N_2’-type substitution reactions of tertiary propargylic alcohols with arylmetallic species affording the non-readily available enantioenriched tetrasubstituted allenes via either exclusive *syn*- or *anti*-*β*-OH elimination.

## Introduction

**Fig. 1:**
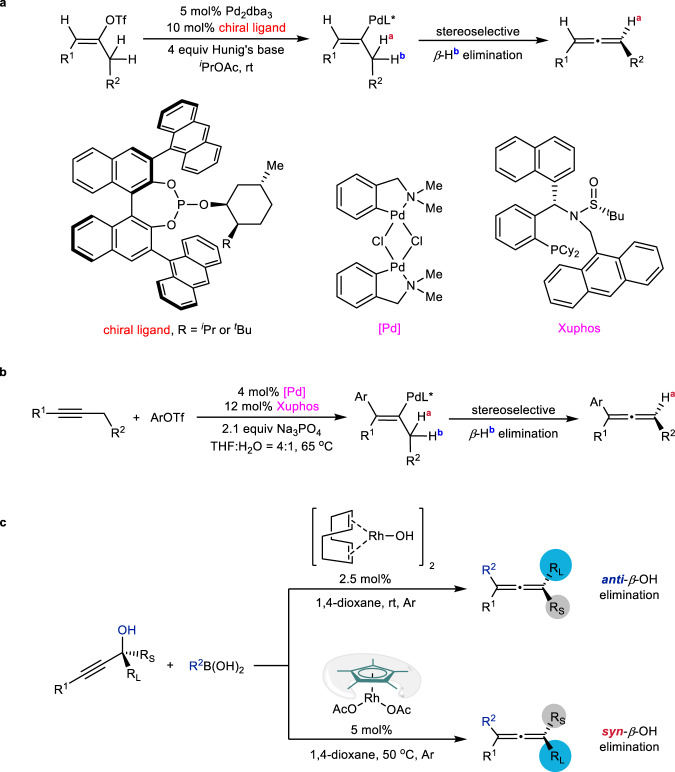
Metal-catalyzed stereoselective *β*-elimination processes for syntheses of chiral allenes. **a** Enantioselective vinyl triflate-based *β*-H elimination. **b** Enantioselective alkyne-based *β*-H elimination. **c** Steric control on *β*-OH elimination: *syn*- vs *anti*-.

Stereoselectivity control is an ever-lasting topic in synthesis. Transition metal-catalyzed asymmetric coupling reaction is one of the most powerful tools to construct optically active allenes^1–3^. Stereoselective *β*-elimination of vinylic metallic species has become one of the most common approaches for asymmetric allene syntheses: In 2013, Frantz and coworkers reported an elegant Pd-catalyzed enantioselective vinyl triflate-based *β*-H elimination reaction to prepare chiral 1,3-disubstituted allenes (Fig. 1a)^4^. Recently, Zhang’s group and our group have jointly demonstrated a Pd-catalyzed asymmetric Heck reaction between aryl triflates and internal alkynes to furnish chiral trisubstituted allenes in good yields with high ees via *β*-H elimination (Fig. 1b)^5^. The chiral allene formation reactions catalyzed by palladium occur via a well-known *syn*-elimination mechanism; however, a comprehensive understanding of the stereoselectivity in rhodium catalyzed reactions, specifically the *β*-OH elimination step, remains unexplored. To the best of our knowledge, the rhodium-catalyzed *anti*-*β*-OH elimination has not been reported, therefore, the studies on this elementary reaction process are of fundamental interest. Herein, we wish to report the steric control in rhodium-catalyzed S_N_2’-type substitution reaction between optically active tertiary propargylic alcohols and arylmetallic species affording enantioenriched tetrasubstituted allenes^6–42^ under two sets of different reaction conditions (Fig. 1c). We believe that such understanding on steric control of *β*-OH elimination provides a new perspective to the stereoselectivity control in metal-catalyzed allene formation reactions.

## Results and discussion

We began our study on the reaction of (*S*)-2-phenyloct-3-yn-2-ol (*S*)-**1a** of ≥99% ee with phenylboronic acid **2a** in the presence of different rhodium catalysts with 1,4-dioxane as the solvent. [Cp*RhCl_2_]_2_ and Rh_2_(OAc)_4_ failed to afford the desired tetrasubstituted allene product **3aa** with 98-99% recovery of (*S*)-**1a** (Table 1, entries 1−2). Interestingly, 65% yield of (*R*)-**3aa** with 93% ee was obtained when [Rh(COD)Cl]_2_ was applied as the catalyst (Table 1, entry 3). Then different Rh(I) catalysts were examined for the reactions in 1,4-dioxane (Table 1, entries 4−8) and [Rh(COD)OH]_2_ turned out to be best, affording (*R*)-**3aa** in 89% yield and 97% ee at 50 ^°^C (Table 1, entry 8). The absolute configuration of optically active **3aa** was tentatively assigned to be *R* based on the Lowe-Brewster rule^43,44^. The solvent effect was further studied. Ethers, such as THF, DME, Et_2_O, and MTBE, are also suitable solvents for this transformation, which afforded (*R*)-**3aa** in 81−87% yields with no less than 91% ees (Table 1, entries 10−13). Ethyl acetate or even acetone could afford (*R*)-**3aa** in 99% or 97% ee (Table 1, entries 14 and 15). Interestingly, even MeOH could afford (*R*)-**3aa** with 93% ee (Table 1, entry 16). When DCM, DCE, toluene, or DMF was used as the solvent, (*R*)-**3aa** was obtained in diminished yields (Table 1, entries 17−20). 100% of (*S*)-**1a** was recovered when the reaction was conducted in MeCN (Table 1, entry 21, for more details on the solvent effect, see: page S50 in the Supplementary Information). Furthermore, 94% yield of (*R*)-**3aa** was obtained with 98% ee when 2.5 mol% of the rhodium catalyst was applied (Table 1, entry 22). Thus, the optimal reaction conditions for the exclusive *anti*-*β*-OH elimination^45,46^ have been identified as shown in entry 22 of Table 1, which have been defined as standard Conditions A.

**Table 1:** Optimization of reaction conditions


Entry	[Rh]	solvent	T (^o^C)	Yield, ee^§^ of (*R*)-3aa (%)	Recovery of (*S*)-1a (%)	yield of (*R*)-4aa	yield of (*S*)-5aa
1	[Cp*RhCl_2_]_2_	1,4-Dioxane	50	0, /	98	0	0
2	Rh_2_(OAc)_4_	1,4-Dioxane	50	0, /	99	0	0
3	[Rh(COD)Cl]_2_	1,4-Dioxane	50	65, 93	11	3	6
4	[Rh(COE)_2_Cl]_2_	1,4-Dioxane	50	0, /	97	0	0
5	[Rh(C_2_H_4_)_2_Cl]_2_	1,4-Dioxane	50	0, /	95	0	0
6	[Rh(NBD)Cl]_2_	1,4-Dioxane	50	3, /	95	0	0
7	Rh(NBD)_2_BF_4_	1,4-Dioxane	50	0, /	96	0	0
8	[Rh(COD)OH]_2_	1,4-Dioxane	50	89, 97	0	0	10
9	[Rh(COD)OH]_2_	1,4-Dioxane	rt	91, 98	0	0	9
10	[Rh(COD)OH]_2_	THF	rt	87, 98	/	/	5
11	[Rh(COD)OH]_2_	DME	rt	81, 98	/	/	4
12	[Rh(COD)OH]_2_	Et_2_O	rt	83, 91	/	1	16
13	[Rh(COD)OH]_2_	MTBE	rt	82, 92	/	1	16
14	[Rh(COD)OH]_2_	Ethyl acetate	rt	87, 99	/	/	13
15	[Rh(COD)OH]_2_	Acetone	rt	55, 97	34	/	2
16	[Rh(COD)OH]_2_	MeOH	rt	57, 93	/	30	10
17	[Rh(COD)OH]_2_	DCM	rt	8, /	82	0	3
18	[Rh(COD)OH]_2_	DCE	rt	9, /	78	0	3
19	[Rh(COD)OH]_2_	Toluene	rt	19, 92	57	1	9
20	[Rh(COD)OH]_2_	DMF	rt	15, 94	81	/	/
21	[Rh(COD)OH]_2_	MeCN	rt	/, /	100	/	/
22^#^	[Rh(COD)OH]_2_	1,4-Dioxane	rt	94, 98	0	0	8

Reaction conditions: (*S*)-**1a** (0.2 mmol), PhB(OH)_2_ (0.4 mmol), and [Rh] (5 mol%) in solvent (1 mL) at T ^o^C unless otherwise noted. Yield and recovery were determined by ^1^H NMR analysis using dibromomethane as the internal standard. ^§^ Determined by HPLC analysis. ^#^ 2.5 mol% of [Rh(COD)OH]_2_ was used. Cp* (pentamethylcyclopentadienyl), COD (1,5-cyclooctadiene), COE (cyclooctene), NBD (2,5-norbornadiene), THF (tetrahydrofuran), DME (1,2-ethanediol dimethyl ether), MTBE (methyl tert-butyl ether), DCM (dichloromethane), DCE (1,2-dichloroethane), DMF (N,N-dimethylformamide).

### Mechanistic studies

**Fig. 2:**
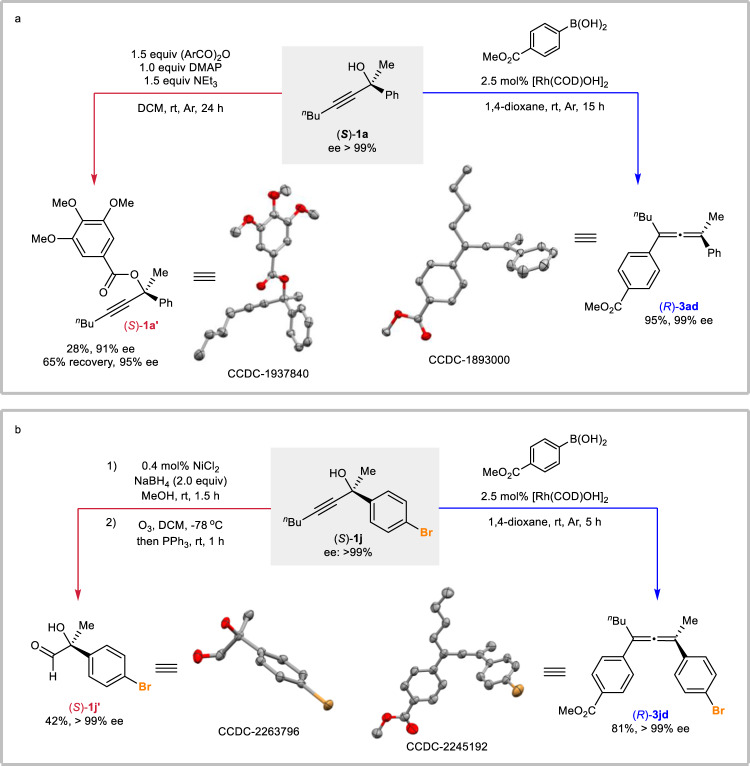
Establishment of the stereochemical outcome. **a** X-ray diffraction studies of (*S*)-**1a’** and (*R*)-**3ad**. **b** X-ray diffraction studies of (*S*)-**1j’** and (*R*)-**3jd** with bromine. The hydrogen atoms are omitted for clarity.

In order to firmly establish the steric outcome of this reaction, (*S*)-**1a** was converted to its 3,4,5-trimethoxybenzoate (*S*)-**1a’**. The absolute configurations of propargylic alcohol (*S*)-**1a** and the corresponding allene product (*R*)-**3ad** were then determined via the single crystal X-ray crystallography of (*S*)-**1a’** and (*R*)-**3ad** (Fig. 2a). With the purpose of further confirming the steric outcome, (*S*)-**1j** with a heavy atom of bromine was applied (Fig. 2b). The X-ray diffraction studies of solid aldehyde (*S*)-**1j’** and the corresponding product (*R*)-**3jd** double confirmed the exclusive *anti*-Rh-OH elimination process.

**Fig. 3:**
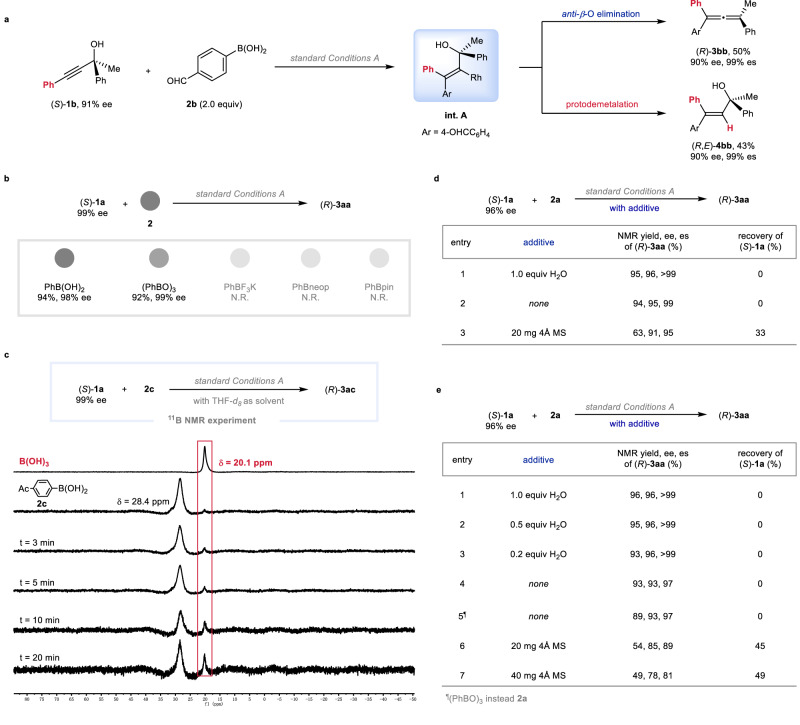
Mechanistic studies. **a** The reaction of (*S*)-**1b** with aryl boronic acid **2b**. **b** The reaction of (*S*)-**1a** with different boron reagents. **c** Monitoring experiment via ^11^B NMR. **d** The role of H_2_O. **e** Further investigation on the effect of H_2_O by using dried starting materials and solvent with the deliberate addition of water.

A series of designed experiments were conducted to get insight into the nature of this reaction: The reaction of (*S*)-2,4-diphenylbut-3-yn-2-ol (*S*)-**1b** (91% ee) furnished the target product (*R*)-**3bb** in 50% yield with 90% ee and 43% yield of the hydroarylation product (*R*,*E*)-**4bb** with 90% ee (Fig. 3a). This result indicated that an alkenylrhodium **int. A** was formed via a regioselective *syn*-insertion of the C-C triple bond into the aryl-rhodium bond formed by the transmetalation of rhodium hydroxide species with arylboronic acid.

When other boron reagent, such as (PhBO)_3_, PhBF_3_K, PhBneop, or PhBpin, was submitted to the standard Conditions A, only triphenylboroxin afforded the desired product in 92% yield with 99% ee (Fig. 3b). In the presence of propargylic alcohol and ambient water, triphenylboroxin may undergo hydrolysis to form phenylboronic acid. These results suggested that boric acid, which was formed after transmetalation, may involve in the reaction and play a critical role. To confirm this hypothesis, the reaction of (*S*)-**1a** with arylboronic acid **2c** in the THF-*d*_*8*_ was monitored by ^11^B NMR experiment and the signal of B(OH)_3_ (*δ* = 20.1 ppm) was observed (Fig. 3c).

To unveil the effect of ambient water in this reaction, several control experiments were performed. The reaction in the presence of 1.0 equiv of H_2_O afforded an even slightly better result (95% yield, 96% ee, >99% es) as compared to the result under the standard conditions (Fig. 3d, entry 1 vs entry 2). When 20 mg of 4 Å molecular sieve were submitted to the reaction, the target product (*R*)-**3aa** was obtained in 63% yield with 91% ee (Fig. 3d, entry 3).

Based on these results, the starting materials ((*S*)-**1a** and organoboron **2a**) and 1,4-dioxane were carefully dried (for full details, see: pages S49−50 in Supplementary Information) to further explore the effect of H_2_O on the Rh-*β*-OH elimination process. The reactions proceeded smoothly via an exclusive *anti*-OH elimination process in the presence of a specific amount of H_2_O, forming (*R*)-**3aa** in very high yields (Fig. 3e, entries 1-3). There was no significant change when PhB(OH)_2_ or (PhBO)_3_ was applied (Fig. 3e, entries 4 and 5). However, compared to the results under the standard conditions (Fig. 3e, entry 4 vs Fig. 3d, entry 2), a slight influence on the ee value was observed (93% ee *vs* 95% ee). Much lower ee for allene product (*R*)-**3aa** was observed by running the reaction in the presence of 20 mg or 40 mg of 4 Å molecular sieve (Fig. 3e, entries 6 and 7). All these results indicated that the ambient water may play a critical role on the stereoselectivity of this Rh-OH elimination reaction.

**Fig. 4:**
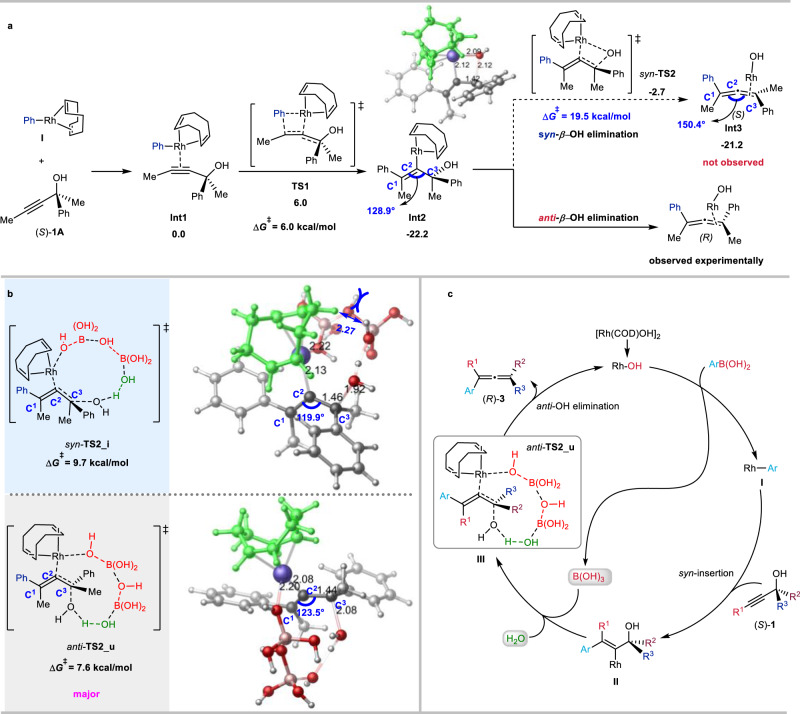
DFT calculations and proposed mechanism for the reaction of (*S*)-2-phenylpent-3-yn-2-ol (*S*)-1A with phenyl-rhodium I. **a** Energy profiles for the *syn*-insertion and the direct *β*-OH elimination processes of (*S*)-**1A** with phenyl-rhodium **I** (Free energies are given in kcal/mol with respect of **Int1**). **b** The transition states for B(OH)_3_ and H_2_O assisted *anti*-*β*-OH elimination process (Bond lengths are given in angstroms). **c** The proposed mechanism.

To shed light on the unique nature of *anti*-OH elimination, detailed density functional theory (DFT) calculations were carried out by using (*S*)-2-phenylpent-3-yn-2-ol (*S*)-**1A** as the model (for details of the computational methods, see: page S75 in the Supplementary Information and calculated coordinates of the optimized structures in Supplementary Data 1). The formation of complex **Int1**, which was selected as the free energy reference, involves the coordination of the triple bond in (*S*)-**1A** with the rhodium atom in metallic species **I** (Fig. 4a). The *syn*-insertion proceeds irreversibly via transition state **TS1** with a free energy barrier of only 6.0 kcal/mol resulting in the formation of the intermediate **Int2**. The subsequent direct *syn-β*-OH elimination via a four-membered ring transition structure *syn-***TS2** would lead to the formation of the final (*S*)-product. However, considering the fact that (*R*)-products were obtained via an exclusive *anti*-*β*-OH elimination experimentally, there must be other possible pathways.

Previous control experiments (Fig. 3b–e) confirmed the critical role of the in situ generated boric acid and ambient water in the reaction. Thus, by utilizing one, two, or three molecules of boric acid or water as the hydroxy shuttle, a series of concerted six-, eight-, or ten-membered cyclic transition structures **TS2_a-z** were obtained regarding Rh-*β*-OH elimination (for details, see: Supplementary Figs. 4–7). Among these transition states, the concerted ten-membered *syn*-**TS2_i** and *anti*-**TS2_u**, were obtained, respectively, as the most stable *syn*- and *anti*-*β*-OH elimination transition structures (Fig. 4b). These two transition structures exhibit activation free energy barriers of 9.7 and 7.6 kcal/mol, respectively, which are more favorable than the direct four-membered ring *syn*-**TS2** (Fig. 4a, 19.5 kcal/mol). Notably, both *syn*-**TS2_i** and *anti*-**TS2_u** involve the collaboration of two molecules of boric acid and one molecule of water. Further examination discovered that the *syn-***TS2_i** experiences a steric repulsion caused by the assisting species B(OH)_3_ with COD ligand, exhibiting a minimal H–H bond distance of only 2.27 Å. As a comparison, no apparent steric repulsions have been detected in *anti*-**TS2_u**. Moreover, there is also difference of the bond angle of C^1^, C^2^ and C^3^, which are 119.9° in *syn-***TS2_i** and 123.5° in *anti-***TS2_u**, respectively (Fig. 4b). By comparison, the bond angle of C^1^, C^2^ and C^3^ is 128.9° in their precursor **Int2** and 150.4° in the corresponding product **Int3**, which indicates that the C^1^, C^2^, and C^3^ unit in *syn-***TS2_i** experiences a greater distortion than that in *anti*-**TS2_u** (Supplementary Fig. 7). Thus, the relative instability of *syn-***TS2_i** compared to *anti-***TS2_u** originates from the steric repulsion between B(OH)_3_ and COD ligand as well as the greater distortion of the C^1^, C^2^, and C^3^ unit in *syn*-**TS2_i**. We, therefore, concluded that the participation of molecules like boric acid and/or water could potentially act as a hydroxy shuttle, aiding in the formation of larger ring transition structures to facilitate the *anti*-*β*-OH elimination.

Based on the experimental and computational data, a rationale for the reaction has been proposed as shown in Fig. 4c: Transmetalation of Rh–OH with arylboronic acid would form the boric acid and aryl-rhodium intermediate **I**, which was followed by the regioselective *syn*-insertion of the C-C triple bond to form the intermediate **II**^42^. Subsequently, in situ generated boric acid and ambient water facilitated the stereospecific *anti*-*β*-OH elimination via concerted ten-membered cyclic transition state *anti*-**TS2_u**, leading to the formation of the final product (*R*)-**3**.

**Fig. 5:**
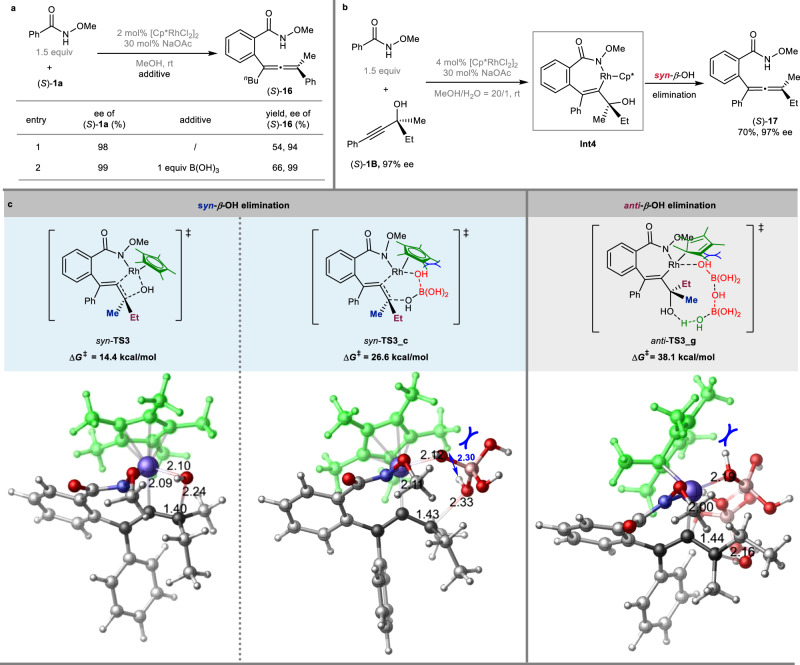
Control experiments and DFT calculations for [Cp*RhCl_2_]_2_ catalyzed reaction of chiral propargylic alcohol (*S*)-1B with *N*-methoxybenzamide^45^. **a** Control experiments with or without boric acid. **b** [Cp*RhCl_2_]_2_ catalyzed reaction of chiral propargylic alcohol (*S*)-**1B** with *N*-methoxybenzamide affording chiral allene (*S*)-**17** via *syn*-*β*-OH elimination of **Int 4**. **c** The transition states for *syn*- or *anti*-*β*-OH elimination process (Bond lengths are given in angstroms).

Subsequently, we turned our attention back to investigate the factor governing the unique exclusive *syn*-*β*-OH elimination observed in our earlier study on the Rh-catalyzed direct reactions of optically active tertiary propargylic alcohols with *N*-methoxybenzamides^45^. As mentioned above, the simultaneous participation of boric acid and water may play a crucial role in the *anti*-Rh-OH elimination pathway. Thus, we carried out the control experiments to investigate the effect of boric acid on the *syn*-*β*-OH elimination reaction. Obviously, the reaction of (*S*)-**1a** still afforded (*S*)-**16** in the presence of 1 equiv of boric acid (Fig. 5a, entry 1 vs entry 2). Further DFT calculations were subsequently conducted on the *β*-OH elimination step from the corresponding rhodacyclic intermediate **Int4** (Fig. 5b), which was generated via the Rh-involved CMD (concerted metalation/deprotonation) process and subsequent regiospecific insertion of the alkyne. In the absence of the assisting molecules, the direct *β*-OH elimination of **Int4** proceeds in *syn* manner via a four-membered transition structure *syn*-**TS3** (Fig. 5c), which requires an energy barrier of 14.4 kcal/mol, leading to the formation of the final product (*S*)-**17**^45^. We conducted further DFT calculations to investigate the effect of the assisting species, such as water, acetic acid, and boric acid, on the *β*-OH elimination step. Unfortunately, computational analysis revealed that all transition states **TS3_a-g** involving the assisting molecules (for details, see: Supplementary Figs. 8–10) were predicted to be unfavorable when compared to *syn*-**TS3** (Fig. 5c). This is attributed to the strong coordination of the *η*^5^-Cp* ligand with the Rh(III) center, creating a very crowded environment preventing the approaching of other assisting molecules. For example, the participation of a boric acid molecule raises the activation energy of the *syn*-*β*-OH elimination to 26.6 kcal/mol via *syn*-**TS3_c** (Fig. 5c), characterized by an minimal H–H bond distance of only 2.30 Å. Remarkably, to circumvent steric hindrance, the Cp* ligand would exhibit an unusual *η*^1^-coordination mode with the Rh(III) center in the *anti*-**TS3_g** structure instead of the conventional *η*^5^-coordination, resulting in the much unfavorable *anti*-**TS3_g** with a high energy barrier of 38.1 kcal/mol (Fig. 5c). Therefore, it is reasonable to conclude that the highly congested surroundings created by the strong coordination of the *η*^5^-Cp* ligand with the Rh(III) center effectively block the approach of other assisting molecules, thus, making direct *syn*-*β*-OH elimination (*syn*-**TS3**) the most favorable pathway.

After unveiling the steric hinderance of Cp* ligand around the rhodium center as the possible critical factor for the *syn*-*β*-OH elimination process for the reaction of the chiral propargylic alcohols with *N*-methoxybenzamides^45^, further optimization experiments were performed for realizing the *syn*-*β*-OH elimination for the reaction of optically active propargylic alcohols with aryl boronic acids. Indeed, with (*S*)-2-phenyloct-3-yn-2-ol (*S*)-**1a** and phenylboronic acid **2a** as the model starting materials, when [Cp*RhCl_2_]_2_ and NaOAc were applied instead of [Rh(COD)OH]_2_, 17% yield of (*S*)-allene product, (*S*)-**3aa**, was formed. To further improve the efficiency of this transformation, the reaction parameters including the loading of NaOAc, temperature, and reaction time were further optimized, however, the conversion was still low (Table 2, entries 2–6). We then turned our attention to the different Cp* ligand containing rhodium catalysts: No desired allene product was observed with Cp*Rh(MeCN)_3_(SbF_6_)_2_ as catalyst (Table 2, entry 7); interestingly, (*S*)-**3aa** was obtained in 90% yield with 99% ee by employing the Cp*Rh(OAc)_2_ as catalyst. Thus, the second set of optimal reaction conditions for the exclusive *syn*-*β*-OH elimination, defined as standard Conditions B, has been established as shown in entry 8 of Table 2.

**Table 2:** Optimization of reaction conditions for exclusive *syn*-*β*-OH elimination


Entry	[Rh]	T (^o^C)	t (h)	Yield, ee^a^ of (*S*)-3aa (%)	Recovery of (*S*)-1a (%)	Yield of byproducts (%)
1	[Cp*RhCl_2_]_2_	rt	5	17, 99	79	5
2^b^	[Cp*RhCl_2_]_2_	rt	5	7, 99	86	8
3	[Cp*RhCl_2_]_2_	50	7	27, 99	71	2
4	[Cp*RhCl_2_]_2_	70	7	35, 99	63	2
5	[Cp*RhCl_2_]_2_	90	7	45, 98	51	3
6	[Cp*RhCl_2_]_2_	90	19	40, 98	42	/
7^c^	Cp*Rh(MeCN)_3_(SbF_6_)_2_	50	16	/, /	/	8
8^c,d^	Cp*Rh(OAc)_2_	50	4.5	90, 99	/	7

Reaction conditions: (*S*)-**1a** (0.2 mmol), PhB(OH)_2_ (0.4 mmol), and [Rh] (5 mol%) in 1,4-dioxane (1 mL) at T ^o^C unless otherwise noted. Yield and recovery were determined by ^1^H NMR analysis using dibromomethane as the internal standard.

^a^Determined by HPLC analysis.

^b^2.0 Equiv of NaOAc were used.

^c^The reaction was conducted in the absence of NaOAc.

^d^3.0 Equiv of **2a** were used.

**Fig. 6:**
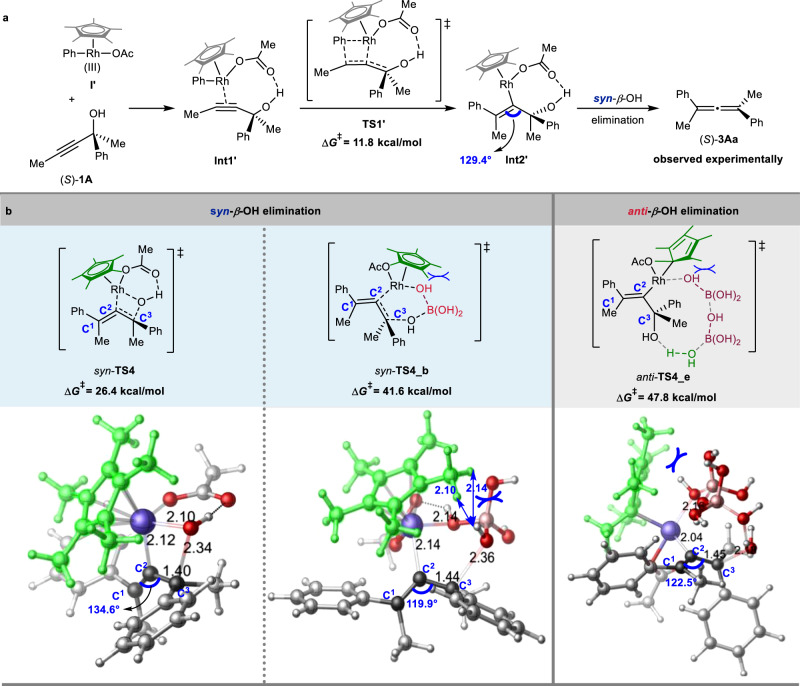
DFT calculations on Cp*Rh(OAc)_2_ catalyzed *syn-β*-OH elimination of (*S*)-2-phenylpent-3-yn-2-ol (*S*)-1A with Cp*PhRh(III)(OAc) I’. **a** Energy profiles for reaction of (*S*)-**1A** with Cp*PhRh(OAc) involving the *syn*-insertion and *syn-β*-OH elimination process of **Int 2’** (Free energies are given in kcal/mol). **b** The transition states for *syn*- or *anti*- *β*-OH elimination process (Bond lengths are given in angstroms).

Detailed DFT calculations were then conducted on the Cp*Rh(OAc)_2_-catalyzed *syn*-*β*-OH elimination process (Fig. 6). The insertion step is computed to be exergonic (ΔG_sol_ = -33.9 kcal/mol) and requires an activation barrier of 11.8 kcal/mol (**TS1’**, Fig. 6a), leading to the formation of the intermediate **Int2’**. Subsequent direct *syn*-*β*-OH elimination step would afford (*S*)-**3Aa** via *syn*-**TS4** requiring a free energy barrier of 26.4 kcal/mol. When the assisting species (water or/and boric acid) are taken into consideration, the *syn*-*β*-OH elimination **TS**s and *anti*-*β*-OH elimination **TS**s are all less favorable as compared with *syn*-**TS4** (for details, see: Supplementary Figs. 11–14). For instance, one molecule of boric acid destabilized the *syn-β-*OH elimination by 15.2 kcal/mol (*syn-***TS4_b** vs. *syn-***TS4**, Fig. 6b). A substantial difference is discernible referring to the bond angle of C^1^, C^2^, and C^3^, measuring 119.9° in *syn-***TS4_b** and 134.6° in *syn-***TS4**, respectively (Fig. 6b). This remarkable difference suggests that the C^1^, C^2^, and C^3^ unit undergoes greater distortion in *syn-***TS4_b** as compared to *syn-***TS4** (Supplementary Fig. 14). Furthermore, *anti*-**TS4_e**, exhibiting an abnormal *η*^1^-coordination mode of the Cp* ligand with Rh(III) center, displayed a relatively high free energy barrier of 47.8 kcal/mol. Thus, the same conclusion can be drown that the strong coordination of the *η*^5^-Cp* ligand with the Rh(III) center produces a highly congested environment that hinders the approach of other assisting molecules, ultimately resulting in a preference for direct *syn*-*β*-OH elimination.

### Substrate scope

**Fig. 7:**
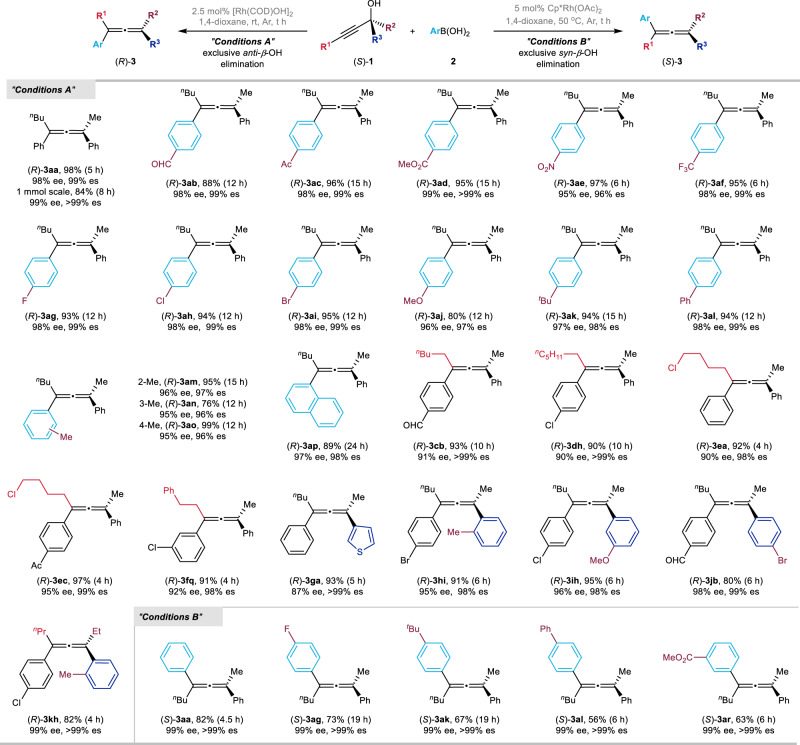
The substrate scope. Conditions A for exclusive *anti*-*β*-OH elimination: (*S*)-**1** (0.2 mmol), **2** (0.4 mmol), and [Rh(COD)OH]_2_ (2.5 mol%) in 1,4-dioxane (1 mL) at rt. Conditions B for exclusive *syn*-*β*-OH elimination: (*S*)-**1** (0.2 mmol), **2** (0.6 mmol), and Cp*Rh(OAc)_2_ (5 mol%) in 1,4-dioxane (1 mL) at 50 ^°^C.

By employing the above optimized conditions (Table 1, entry 22), the scope of the reaction involving different aryl boronic acids was first explored with (*S*)-**1a** (Fig. 7, Condition A). Various electron-withdrawing synthetically versatile functional groups including formyl, acetyl, ester, nitro, trifluoromethyl, and halides were well tolerated in this reaction, affording the desired products (*R*)-**3ab**−**3ai** in 88–97% yields with no less than 95% ee. Introducing the electron-donating groups, such as methoxy and *tert*-butyl, to the *para*-position of the phenyl group rendered the reaction to give products (*R*)-**3aj** and (*R*)-**3ak** in 80% yield with 96% ee and 94% yield with 97% ee, respectively. Aryl boronic acids with the *ortho*-, *meta*-, and *para*-methyl-substituent in the phenyl moiety afforded the desired products (*R*)-**3am**−**3ao** in high yields (76%–99%) with 95%–96% ees, indicating there is no obvious steric effect. Moreover, 1-naphthylboronic acid also worked smoothly affording (*R*)-**3ap**. Various tertiary propargylic alcohols were also surveyed under the optimized reaction conditions. R^1^ group could be ^*n*^C_5_H_11_, ^*n*^C_6_H_13_, -(CH_2_)_4_Cl, and -(CH_2_)_2_Ph, affording products (*R*)-**3cb**, (*R*)-**3dh**, (*R*)-**3ea**, (*R*)-**3ec**, and (*R*)-**3fq** in gratifying results (90%–97% yields with 90%–95% ees). The substrate containing 3-thienyl group reacted under the standard conditions to give the corresponding product (*R*)-**3ga** in 93% yield with 87% ee. R^3^ may also be an aryl group substituted with different substituents on the 2-, 3-, or 4-position, providing the desired products (*R*)-**3hi**, (*R*)-**3ih**, and (*R*)-**3jb** in good yields with an excellent enantiomeric excess. When R^2^ is an Et group, the corresponding product (*R*)-**3kh** was obtained in 82% yield with 99% ee. On the other hand, using the steric hindered catalyst Cp*Rh(OAc)_2_ (conditions B), exclusive *syn*-elimination process has been achieved: The reaction of phenylboronic acid with (*S*)-**1a** could give (*S*)-**3aa** in 82% yield with 99% ee; the reaction is amenable to both the electron-withdrawing groups, such as fluoro, phenyl, and ester, and electron-donating group *tert*-butyl with the formation of the corresponding enantiomeric allene products (*S*)-**3ag**-**3ar** in 56–73% yields with 99% ees from the same starting materials.

### Synthetic applications

**Fig. 8:**
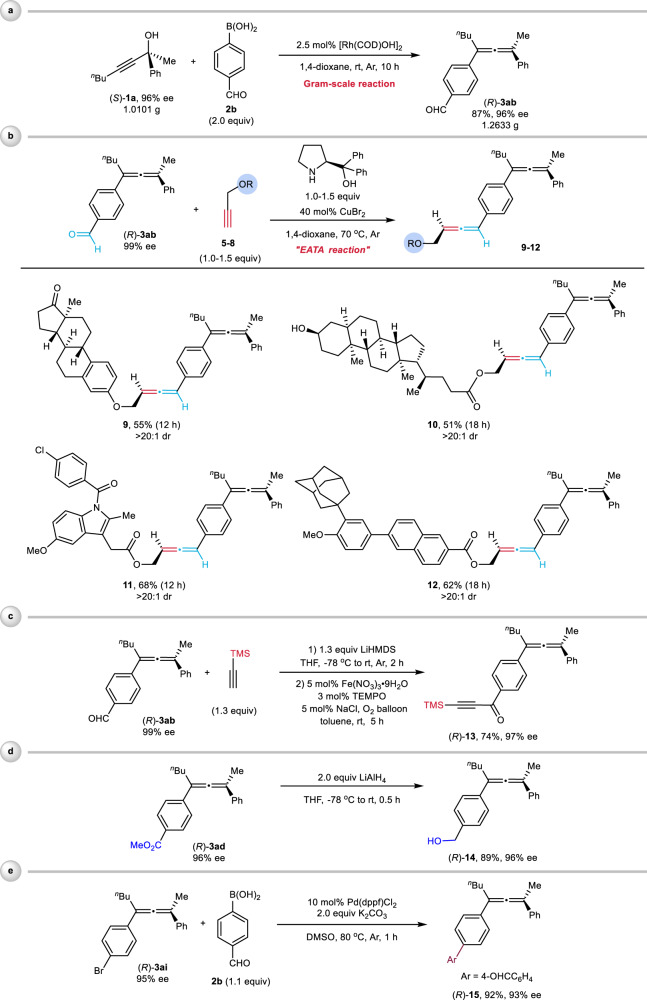
Gram-scale reaction and synthetic transformations of the products. **a** Gram-scale reaction. **b** Copper-catalyzed enantioselective allenation of (*R*)-**3ab** with terminal propargylic ethers **5**–**8**. **c** Synthesis of alkynyl ketone (*R*)-**13** from (*R*)-**3ab**. **d** Reduction of (*R*)-**3ad** to alcohol (*R*)-**14** with LiAlH_4_. **e** Suzuki coupling reaction of (*R*)-**3ai** to (*R*)-**15** with **2b**.

To demonstrate the synthetic potentials of this protocol, 1.0101 g of (*S*)-**1a** and 2.0 equiv of 4-formylphenylboronic acid **2b** were subjected to the optimized reaction conditions A to give (*R*)-**3ab** in 87% yield with 96% ee (Fig. 8a). The skeletons of bioactive compounds (estrone and lithocolic acid) and drug molecules (indometacin and adapalene) were successfully introduced via the copper-catalyzed enantioselective allenation of their terminal propargylic ethers **5**-**8** (EATA reaction)^15,47^ with the aldehyde functionality in chiral allene (*R*)-**3ab** affording a series of bisallene products **9**–**12**, which contain a chiral tetrasubstituted allene unit and a chiral 1,3-disubstituted allene motif in moderate yields with excellent diastereoselectivities (Fig. 8b). In addition, the 1,2-addition of (trimethylsilyl)ethynyl lithium with this aldehyde entity followed by iron-catalyzed aerobic oxidation reaction^48^ afforded alkynyl ketone (*R*)-**13** in 74% yield with 97% ee without touching the allene unit (Fig. 8c). The ester functionality in (*R*)-**3ad** may be reduced with LiAlH_4_ to afford the corresponding alcohol (*R*)-**14** in 89% yield without erosion of ee (Fig. 8d). Allene (*R*)-**15** was obtained in 92% yield with 93% ee by Suzuki coupling reaction^49^ of (*R*)-**3ai** with 4-formylphenylboronic acid (Fig. 8e).

In this work, we have demonstrated the Rh(I)-catalyzed exclusive *anti-β*-OH elimination of an alkenylmetal species forming enantioenriched tetrasubstituted allenes from readily available optically active tertiary propargylic alcohols^50^ and arylboronic acids with an excellent efficiency of chirality transfer. Experimental evidences reveal that assisting molecules such as boric acid and water play a critical role on this exclusive *anti*-Rh(I)-OH elimination process and DFT calculations led to a ten-membered cyclic transition state consisting of [2B(OH)_3_]-[1H_2_O] with an energy as low as 7.6 kcal/mol. And DFT studies suggest that the previously reported exclusive *syn*-Rh(III)-OH elimination is controlled by a four-membered cyclic transition state (*syn-***TS3**), due to the steric surroundings around the Rh(III) center preventing the approaching of the other assisting molecules. Based on these mechanistic insights, *syn-β*-OH elimination has also been realized by using Cp*Rh(OAc)_2_ as the catalyst affording the enantiomer from the same starting materials. Further studies are currently underway in our laboratory.

## Methods

### General method for the Rh-catalyzed *β*-OH elimination for allene formation reaction is provided in the following

To an oven-dried Schlenk tube (25 mL) was added phenylboronic acid **2a** (48.9 mg, 0.4 mmol), which was then transferred to a glovebox. After adding [Rh(COD)OH]_2_ (2.4 mg, 0.005 mmol) in the glovebox, it was transferred out of the glovebox. After replacing nitrogen with argon for three times by vaccum, (*S*)-**1a** (40.3 mg, 0.2 mmol, 99% ee) and freshly distilled dioxane (1 mL) were added. The resulting mixture was vigorously stirred at rt for 5 h as monitored by TLC, diluted with ethyl acetate (1 mL), filtered through a short column of silica gel (1 cm), eluted with ethyl acetate (5 mL), and concentrated. The residue was purified by chromatography on silica gel to afford (*R*)-**3aa** (51.4 mg, 98%) [eluent: petroleum ether/ethyl acetate = 60/1 (~120 mL)]: 98% ee (HPLC conditions: OJ-H column, hexane/*i*-PrOH = 99.5/0.5, 0.7 mL/min, λ = 214 nm, *t*_R_ (major) = 6.3 min, *t*_R_ (minor) = 8.0 min); [*α*]_D_^27^ = -326.1 (*c* = 1.17, CHCl_3_); oil; ^**1**^**H NMR** (400 MHz, CDCl_3_): δ = 7.43 (t, *J* = 7.2 Hz, 4 H, Ar-H), 7.29 (m, 4 H, Ar-H), 7.23–7.12 (m, 2 H, Ar-H), 2.62–2.47 (m, 2 H, CH_2_), 2.20 (s, 3 H, CH_3_), 1.64–1.49 (m, 2 H, CH_2_), 1.49-1.35 (m, 2 H, CH_2_), 0.90 (t, *J* = 7.4 Hz, 3 H, CH_3_); ^**13**^**C NMR** (100 MHz, CDCl_3_): δ = 205.5, 137.2, 137.0, 128.41, 128.37, 126.7, 126.6, 126.0, 125.6, 107.8, 103.6, 30.1, 30.0, 22.6, 16.8, 14.0; **IR** (neat): *v* = 3058, 2954, 2925, 2858, 1932, 1596, 1491, 1443, 1025 cm^-1^; **MS** (70 eV, EI) *m/z* (%): 263 (M^+^ + 1, 1.21), 262 (M^+^, 5.56), 205 (100); **HRMS** calcd for C_20_H_22_ [M^+^]: 262.1716, found: 262.1725.

### Supplementary information

Supplementary Information

Description of Additional Supplementary Files

Supplementary Data 1

## Data Availability

The X-ray structure data generated in this study have been deposited in the Cambridge Crystallographic Data Center (CCDC: 1937840 for (*S*)-**1a**’, 1893000 for (*R*)-**3ad**, 2263796 for (*S*)-**1j**’, and 2245192 for (*R*)-**3jd**). Copies of the data can be obtained free of charge via https://www.ccdc.cam.ac.uk/structures/. Characterization and spectra of new compounds are included in the Supplemental information. Experimental procedures and characterization of the new compounds are available in the Supplementary Information. Calculated coordinates of the optimized structures are available in the Supplementary Data file. All other data are available from the authors upon request.
