# Supplementary material for: Stereoselectivity control in Rh-catalyzed β-OH elimination for chiral allene formation
Source: Nat Commun. 2023 Nov 16;14:7399. doi: 10.1038/s41467-023-42660-1 (PMC10651921; doi:10.1038/s41467-023-42660-1)
Supplement: Supplementary file 1 — Supplementary Information [file 41467_2023_42660_MOESM1_ESM.pdf]

Supplementary Information

Stereoselectivity Control in Rh-catalyzed *β*-OH Elimination for Chiral Allene Formation

Jie Wang,[1,3] Wei-Feng Zheng,[1,3] Xue Zhang,*[2] Hui Qian,*[1] and Shengming Ma*[1,2]

[1]Research Center for Molecular Recognition and Synthesis, Department of Chemistry, Fudan University, 220 Handan Lu, Shanghai 200433, P. R. China. E-mail: qian_hui@fudan.edu.cn

[2]State Key Laboratory of Organometallic Chemistry, Shanghai Institute of Organic Chemistry, Chinese Academy of Sciences, 345 Lingling Lu, Shanghai 200032, P. R. China. E-mail: masm@sioc.ac.cn; xzhang@sioc.ac.cn

[3]These authors contributed equally.

**1. Supplementary methods**	
1.1. General information	S2
1.2. Preparation of racemic allenes	S3-S19
1.3. Preparation of chiral allenes	S19-S42
1.4. Preparation and characterization of the byproducts **4aa** and **5aa**	S42-S45
1.5. Mechanistic studies	S45-S50
1.6. Detail studies on absolute configuration determination	S51-S63
1.7. Synthetic application	S63-S74
**2. Supplementary DFT calculational details**	S75-S87
**3. Supplementary ^{1}H and ^{13}C NMR and HPLC spectra**	S88-S372
**4. Supplementary references**	S372-S374

1. Supplementary methods

1.1. General Information. NMR spectra were taken with a Bruker Avance III spectrometer (400 MHz for ^{1}H NMR, 100 MHz for ^{13}C NMR, 376 MHz for ^{19}F NMR, and 128 MHz for ^{11}B NMR) in $CDCl_3$. All ^{1}H NMR experiments were measured with tetramethylsilane (0 ppm) in $CDCl_3$ as the internal reference; ^{13}C NMR experiments were measured in relative to the signal of $CDCl_3$ (77.0 ppm). All reactions were carried out in Schlenk tubes or Schlenk flasks. Petroleum ether (b.p. 60-90 °C) was purchased from Shanghai Titan Scientific Co., Ltd. Tetrahydrofuran and dioxane were dried over sodium wire with benzophenone as the indicator and distilled freshly before use. All the temperatures are referred to the oil baths used. Recoveries of substrates were determined by ^{1}H NMR analysis using dibromomethane as the internal standard. The chiral propargylic alcohols were prepared according to the literature methods.[1] The crude allene product was loaded to the top of the column by dropper as a solution in 0.5~1.0 mL of DCM.

1.2. Preparation of racemic allenes.

(1) Preparation of 2-phenyl-4-(4-formylphenyl)-2,3-octadiene (*rac*-3ab) (zwf-5-036)

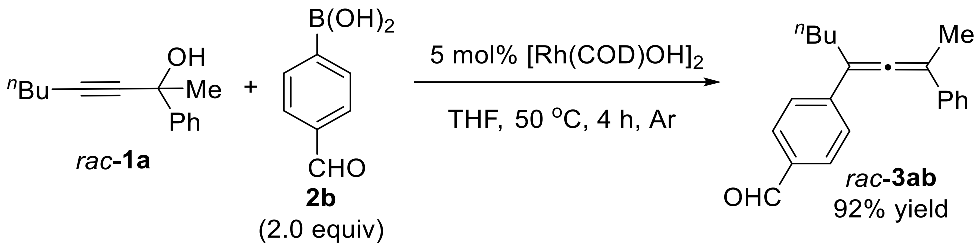

Typical Procedure I: To an oven-dried Schlenk tube (25 mL) was added 4-formylphenylboronic acid **2b** (60.2 mg, 0.4 mmol), which was then transferred to a glovebox. After adding $[Rh(COD)OH]_2$ (4.7 mg, 0.01 mmol) in glovebox, it was transferred out of the glovebox. After replacing nitrogen with argon for three times at rt by vacuum, *rac*-**1a** (40.5 mg, 0.2 mmol) and freshly distilled THF (1 mL) were added. The resulting mixture was vigorously stirred at 50 ºC for 4 h as monitored by TLC, diluted with ethyl acetate (1 mL), filtered through a short column of silica gel (1 cm), eluted with ethyl acetate (5 mL), and concentrated. The residue was purified by chromatography on silica gel to afford *rac*-**3ab**[2] (53.4 mg, 92%) [eluent: petroleum ether = 100 mL, petroleum ether / ethyl acetate = 20/1 (~120 mL)]: oil; **^{1}H NMR** (400 MHz, $CDCl_3$): δ = 9.96 (s, 1 H, CHO), 7.81 (d, *J* = 8.0 Hz, 2 H, Ar-H), 7.57 (d, *J* = 8.0 Hz, 2 H, Ar-H), 7.43 (d, *J* = 7.6 Hz, 2 H, Ar-H), 7.34 (t, *J* = 7.6 Hz, 2 H, Ar-H), 7.27-7.18 (m, 1 H, Ar-H), 2.66-2.48 (m, 2 H, CH_2), 2.24 (s, 3 H, CH_3), 1.63-1.50 (m, 2 H, CH_2), 1.50-1.37 (m, 2 H, CH_2), 0.92 (t, *J* = 7.4 Hz, 3 H, CH_3); **^{13}C NMR** (100 MHz, $CDCl_3$): δ = 207.2, 191.7, 143.7, 136.3, 134.7, 129.9, 128.5, 127.0, 126.4, 125.7, 107.5, 104.5, 30.0, 29.8, 22.5, 16.6, 13.9; **IR** (neat): *v* = 2956, 2928, 1930, 1698, 1599, 1212, 1168 cm^{-1}; **MS** (70 eV, EI) *m/z* (%): 291 (M^++1, 2.38), 290 (M^+, 9.77), 205 (100).

(2) Preparation of 2-phenyl-4-(4-acetylphenyl)-2,3-octadiene (*rac*-3ac) (zwf-6-176)

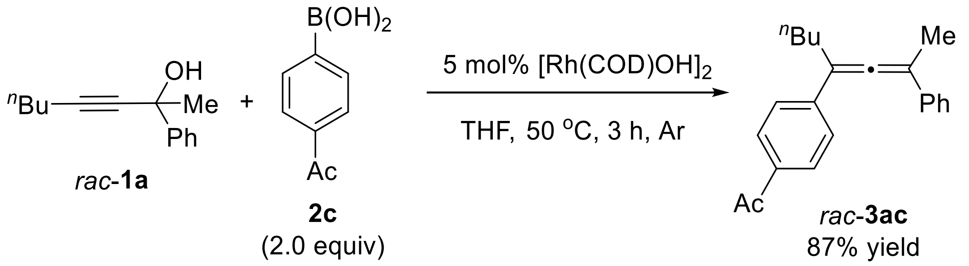

Following Typical Procedure I, the reaction of *rac*-**1a** (40.3 mg, 0.2 mmol), 4-acetylphenylboronic acid **2c** (65.8 mg, 0.4 mmol), [Rh(COD)OH]$_2$ (4.4 mg, 0.01 mmol) and THF (1 mL) afforded *rac*-**3ac**[2] (52.6 mg, 87%) [eluent: petroleum ether / ethyl acetate = 60/1 (~360 mL)]: oil; **^{1}H NMR** (400 MHz, CDCl$_3$): δ = 7.89 (d, J = 8.0 Hz, 2 H, Ar-H), 7.50 (d, J = 8.0 Hz, 2 H, Ar-H), 7.43 (d, J = 8.0 Hz, 2 H, Ar-H), 7.33 (t, J = 7.6 Hz, 2 H, Ar-H), 7.26-7.16 (m, 1 H, Ar-H), 2.64-2.45 (m, 5 H, CH$_2$ and CH$_3$), 2.23 (s, 3 H, CH$_3$), 1.64-1.50 (m, 2 H, CH$_2$), 1.50-1.34 (m, 2 H, CH$_2$), 0.92 (t, J = 7.2 Hz, 3 H, CH$_3$); **^{13}C NMR** (100 MHz, CDCl$_3$): δ = 206.8, 197.5, 142.2, 136.5, 135.3, 128.54, 128.46, 126.9, 126.0, 125.6, 107.4, 104.3, 30.0, 29.8, 26.5, 22.5, 16.6, 13.9; **IR** (neat): v = 2955, 2927, 2870, 1930, 1681, 1600, 1265, 1026 cm^{-1}; **MS** (70 eV, EI) *m/z* (%): 305 (M$^+$+1, 8.75), 304 (M$^+$, 32.82), 247 (100).

(3) Preparation of 2-phenyl-4-(4-(methoxycarbonyl)phenyl)-2,3-octadiene (*rac*-3ad) (zwf-5-041)

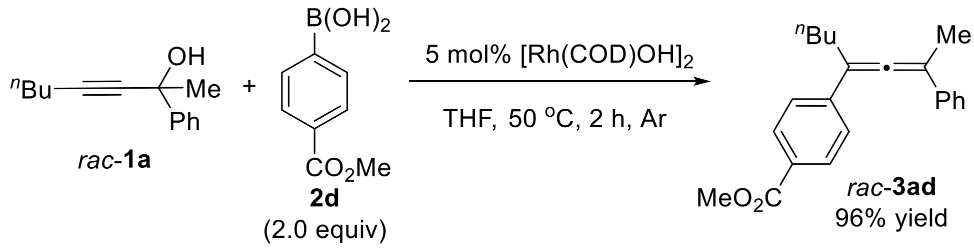

Following Typical Procedure I, the reaction of *rac*-**1a** (40.8 mg, 0.2 mmol), 4-(methoxycarbonyl)phenylboronic acid **2d** (72.4 mg, 0.4 mmol), [Rh(COD)OH]$_2$ (4.6 mg, 0.01 mmol) and THF (1 mL) afforded *rac*-**3ad**[2] (62.2 mg, 96%) [eluent: petroleum ether / ethyl acetate = 40/1 (~120 mL)]: white solid; m.p. 69.4-71.2 °C (petroleum ether/DCM); **^{1}H NMR** (400 MHz, CDCl$_3$): δ = 7.96 (d, J = 8.4 Hz, 2 H, Ar-H), 7.55-7.38 (m, 4 H, Ar-H), 7.33 (t, J = 7.6 Hz, 2 H, Ar-H), 7.27-7.18 (m, 1 H, Ar-H), 3.90 (s,

3 H, OCH_3), 2.65-2.48 (m, 2 H, CH_2), 2.23 (s, 3 H, CH_3), 1.60-1.50 (m, 2 H, CH_2), 1.50-1.33 (m, 2 H, CH_2), 0.91 (t, J = 7.2 Hz, 3 H, CH_3); **^{13}C NMR** (100 MHz, $CDCl_3$): δ = 206.6, 166.9, 142.0, 136.6, 129.7, 128.5, 128.2, 126.9, 125.8, 125.6, 107.5, 104.2, 51.9, 30.0, 29.8, 22.5, 16.7, 13.9; **IR** (neat): v = 2955, 2924, 2862, 1929, 1714, 1603, 1437, 1272, 1107 cm^{-1}; **MS** (70 eV, EI) *m/z* (%): 321 (M^++1, 2.51), 320 (M^+, 9.47), 263 (100).

(4) Preparation of 2-phenyl-4-(4-nitrophenyl)-2,3-octadiene (*rac*-3ae) (zwf-7-004)

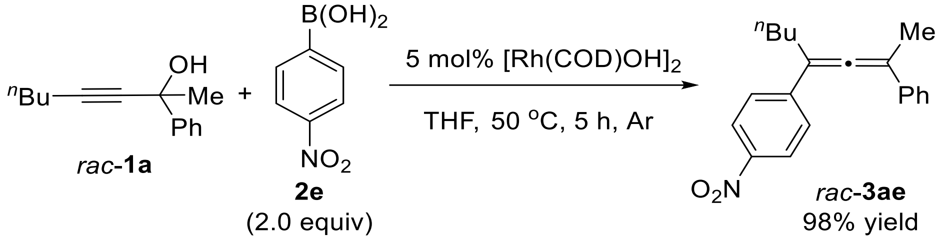

Following Typical Procedure I, the reaction of *rac*-**1a** (40.1 mg, 0.2 mmol), 4-nitrophenylboronic acid **2e** (66.5 mg, 0.4 mmol), $[Rh(COD)OH]_2$ (4.6 mg, 0.01 mmol) and THF (1 mL) afforded *rac*-**3ae** (60.0 mg, 98%) [eluent: petroleum ether / ethyl acetate = 50/1 (~200 mL)]: oil; **^{1}H NMR** (400 MHz, $CDCl_3$): δ = 8.14 (d, J = 8.8 Hz, 2 H, Ar-H), 7.53 (d, J = 8.8 Hz, 2 H, Ar-H), 7.42 (d, J = 7.6 Hz, 2 H, Ar-H), 7.34 (t, J = 7.6 Hz, 2 H, Ar-H), 7.24 (t, J = 7.2 Hz, 1 H, Ar-H), 2.65-2.46 (m, 2 H, CH_2), 2.24 (s, 3 H, CH_3), 1.64-1.53 (m, 2 H, CH_2), 1.50-1.35 (m, 2 H, CH_2), 0.92 (t, J = 7.2 Hz, 3 H, CH_3); **^{13}C NMR** (100 MHz, $CDCl_3$): δ = 207.4, 146.3, 144.3, 136.0, 128.6, 127.2, 126.5, 125.7, 123.7, 107.0, 104.9, 29.9, 29.8, 22.5, 16.6, 13.9; **IR** (neat): v = 2956, 2928, 2859, 1929, 1591, 1513, 1337, 1108, 1026 cm^{-1}; **MS** (70 eV, EI) *m/z* (%): 308 (M^++1, 2.14), 307 (M^+, 8.07), 250 (100); **HRMS** calcd *m/z* for $C_{20}H_{21}NO_2$ [M^+]: 307.1567, found: 307.1565.

(5) Preparation of 2-phenyl-4-(4-trifluoromethylphenyl)-2,3-octadiene (*rac*-3af) (zwf-7-003)

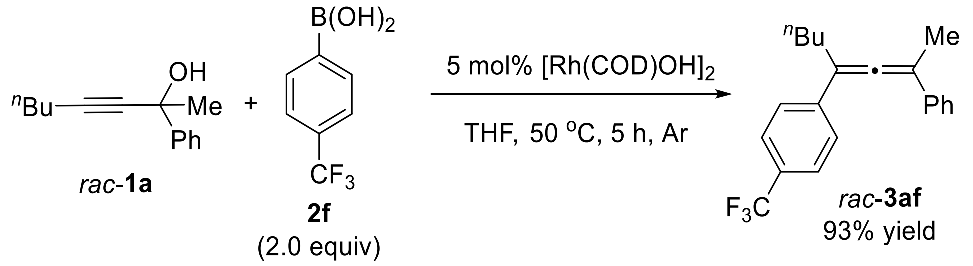

Following Typical Procedure I, the reaction of *rac*-**1a** (39.6 mg, 0.2 mmol), 4-trifluoromethylphenylboronic acid **2f** (76.2 mg, 0.4 mmol), $[Rh(COD)OH]_2$ (4.5 mg, 0.01 mmol) and THF (1 mL) afforded *rac*-**3af**[2] (60.5 mg, 93%) [eluent: petroleum ether / ethyl acetate = 50/1 (~200 mL)]: oil; **^{1}H NMR** (400 MHz, $CDCl_3$): δ = 7.61-7.46 (m, 4 H, Ar-H), 7.43 (d, J = 8.0 Hz, 2 H, Ar-H), 7.33 (t, J = 7.6 Hz, 2 H, Ar-H), 7.26-7.16 (m, 1 H, Ar-H), 2.61-2.46 (m, 2 H, CH_2), 2.20 (s, 3 H, CH_3), 1.62-1.52 (m, 2 H, CH_2), 1.48-1.35 (m, 2 H, CH_2), 0.92 (t, J = 7.2 Hz, 3 H, CH_3); **^{13}C NMR** (100 MHz, $CDCl_3$): δ = 206.3, 141.0, 136.6, 128.6 (q, J = 32.1 Hz), 128.5, 127.0, 126.2, 125.7, 125.3 (q, J = 3.7 Hz), 124.3 (q, J = 270.2 Hz), 107.1, 104.4, 30.0, 29.9, 22.6, 16.7, 13.9; **^{19}F NMR** (376 MHz, $CDCl_3$): δ = -62.9; **IR** (neat): v = 2958, 2930, 2861, 1933, 1615, 1493, 1322, 1163, 1112, 1026 cm^{-1}; **MS** (70 eV, EI) m/z (%): 331 (M^++1, 2.00), 330 (M^+, 8.69), 273 (100).

(6) Preparation of 2-phenyl-4-(4-fluorophenyl)-2,3-octadiene (*rac*-3ag) (zwf-6-166)

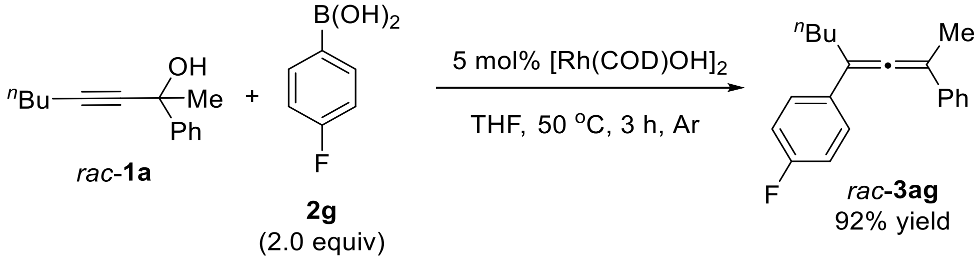

Following Typical Procedure I, the reaction of *rac*-**1a** (40.0 mg, 0.2 mmol), 4-fluorophenylboronic acid **2g** (56.9 mg, 0.4 mmol), $[Rh(COD)OH]_2$ (4.7 mg, 0.01 mmol) and THF (1 mL) afforded *rac*-**3ag**[2] (51.3 mg, 92%) [eluent: petroleum ether / ethyl acetate = 60/1 (~120 mL)]: oil; **^{1}H NMR** (400 MHz, $CDCl_3$): δ = 7.43 (d, J = 8.0 Hz, 2 H, Ar-H), 7.40-7.26 (m, 4 H, Ar-H), 7.20 (t, J = 7.2 Hz, 1 H, Ar-H), 6.98 (t, J = 8.6 Hz, 2 H, Ar-H), 2.59-2.42 (m, 2 H, CH_2), 2.20 (s, 3 H, CH_3), 1.62-1.49 (m, 2 H, CH_2), 1.48-

1.35 (m, 2 H, CH_2), 0.90 (t, J = 7.4 Hz, 3 H, CH_3); **^{13}C NMR** (100 MHz, $CDCl_3$): δ = 205.2 (d, J = 1.5 Hz), 161.8 (d, J = 244.1 Hz), 137.1, 133.0 (d, J = 3.2 Hz), 128.4, 127.5 (d, J = 7.9 Hz), 126.7, 125.6, 115.2 (d, J = 21.4 Hz), 107.0, 103.8, 30.2, 30.0, 22.6, 16.8, 13.9; **^{19}F NMR** (376 MHz, $CDCl_3$): δ = -116.7; **IR** (neat): v = 2956, 2927, 2859, 1933, 1601, 1506, 1492, 1464, 1227, 1158, 1026 cm^{-1}; **MS** (70 eV, EI) *m/z* (%): 281 (M^++1, 2.13), 280 (M^+, 8.94), 223 (100).

(7) Preparation of 2-phenyl-4-(4-chlorophenyl)-2,3-octadiene (*rac*-3ah) (zwf-6-173)

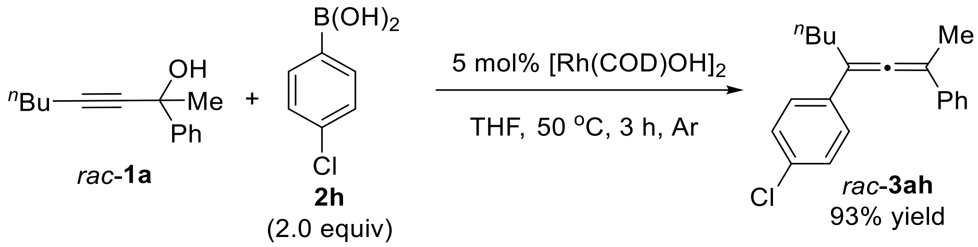

Following Typical Procedure I, the reaction of *rac*-**1a** (40.5 mg, 0.2 mmol), 4-chlorophenylboronic acid **2h** (62.8 mg, 0.4 mmol), $[Rh(COD)OH]_2$ (4.5 mg, 0.01 mmol) and THF (1 mL) afforded *rac*-**3ah**[2] (55.1 mg, 93%) [eluent: petroleum ether / ethyl acetate = 60/1 (~120 mL)]: oil; **^{1}H NMR** (400 MHz, $CDCl_3$): δ = 7.42 (d, J = 7.6 Hz, 2 H, Ar-H), 7.38-7.28 (m, 4 H, Ar-H), 7.28-7.18 (m, 3 H, Ar-H), 2.59-2.42 (m, 2 H, CH_2), 2.20 (s, 3 H, CH_3), 1.61-1.49 (m, 2 H, CH_2), 1.47-1.34 (m, 2 H, CH_2), 0.90 (t, J = 7.2 Hz, 3 H, CH_3); **^{13}C NMR** (100 MHz, $CDCl_3$): δ = 205.5, 136.9, 135.6, 132.3, 128.5, 128.4, 127.3, 126.8, 125.6, 107.0, 104.1, 30.0, 29.9, 22.6, 16.8, 13.9; **IR** (neat): v = 2958, 2925, 2856, 1933, 1596, 1490, 1092, 1014 cm^{-1}; **MS** (70 eV, EI) *m/z* (%): 298 (M^+(^{37}Cl), 3.74), 296 (M^+(^{35}Cl), 8.80), 239 (100).

(8) Preparation of 2-phenyl-4-(4-bromophenyl)-2,3-octadiene (*rac*-3ai) (zwf-5-035)

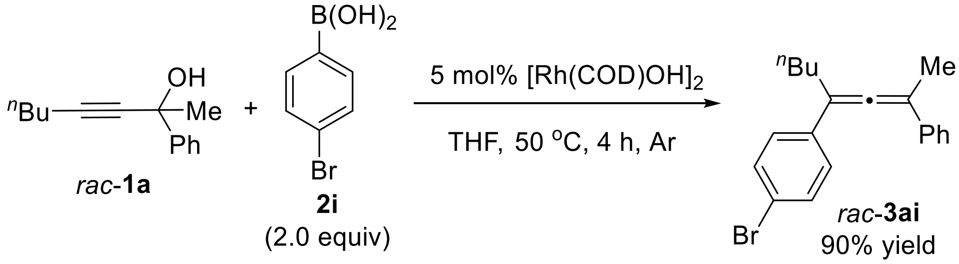

Following Typical Procedure I, the reaction of *rac*-**1a** (40.0 mg, 0.2 mmol), 4-bromophenylboronic acid **2i** (80.4 mg, 0.4 mmol), $[Rh(COD)OH]_2$ (4.5 mg, 0.01 mmol) and THF (1 mL) afforded *rac*-**3ai** (60.6 mg, 90%) [eluent: petroleum ether = 200 mL]:white solid; m.p. 58.6-59.1 °C (petroleum ether/DCM); ^{1}**H NMR** (400 MHz, $CDCl_3$): δ = 7.47-7.36 (m, 4 H, Ar-H), 7.36-7.26 (m, 4 H, Ar-H), 7.24-7.16 (m, 1 H, Ar-H), 2.61-2.41 (m, 2 H, CH_2), 2.20 (s, 3 H, CH_3), 1.61-1.47 (m, 2 H, CH_2), 1.47-1.33 (m, 2 H, CH_2), 0.90 (t, J = 7.4 Hz, 3 H, CH_3); ^{13}**C NMR** (100 MHz, $CDCl_3$): δ = 205.5, 136.8, 136.0, 131.4, 128.4, 127.6, 126.8, 125.6, 120.4, 107.1, 104.1, 30.0, 29.9, 22.6, 16.7, 14.0; **IR** (neat): v = 2959, 2924, 2857, 1929, 1487, 1071, 1007 cm^{-1}; **MS** (70 eV, EI) *m/z* (%): 342 (M^+(^{81}Br), 5.22), 340 (M^+(^{79}Br), 5.36), 204 (100); Anal. Calcd. for $C_{20}H_{21}{}^{79}Br$: C 70.39, H 6.20; found C 70.39, H 6.22.

(9) Preparation of 2-phenyl-4-(4-methoxyphenyl)-2,3-octadiene (*rac*-3aj) (zwf-6-178)

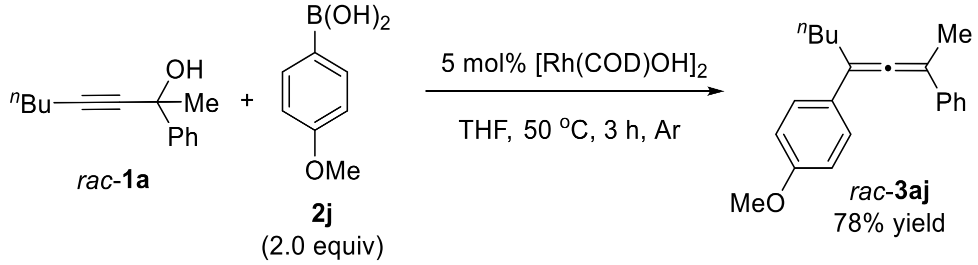

Following Typical Procedure I, the reaction of *rac*-**1a** (40.0 mg, 0.2 mmol), 4-methoxyphenylboronic acid **2j** (60.9 mg, 0.4 mmol), $[Rh(COD)OH]_2$ (4.6 mg, 0.01 mmol) and THF (1 mL) afforded *rac*-**3aj**[2] (45.2 mg, 78%) [eluent: petroleum ether / ethyl acetate = 60/1 (~120 mL)]: oil; ^{1}**H NMR** (400 MHz, $CDCl_3$): δ = 7.49-7.38 (m, 2 H, Ar-H), 7.38-7.25 (m, 4 H, Ar-H), 7.23-7.17 (m, 1 H, Ar-H), 6.91-6.77 (m, 2 H, Ar-H), 3.78 (s, 3 H, OCH_3), 2.60-2.43 (m, 2 H, CH_2), 2.19 (s, 3 H, CH_3), 1.61-1.51 (m, 2 H, CH_2), 1.47-1.34 (m, 2 H, CH_2), 0.90 (t, J = 7.2 Hz, 3 H, CH_3); ^{13}**C NMR** (100 MHz, $CDCl_3$): δ = 205.0, 158.6, 137.5, 129.3, 128.3, 127.1, 126.5, 125.6, 113.9, 107.3, 103.4, 55.3, 30.13, 30.09, 22.6, 16.9, 14.0; **IR** (neat): v = 2954, 2928, 2858, 1933, 1605, 1509, 1245, 1176, 1026 cm^{-1}; **MS** (70 eV, EI) *m/z* (%): 293 (M^++1, 4.21), 292 (M^+, 20.45), 235 (100).

(10) Preparation of 2-phenyl-4-(4-*tert*-butylphenyl)-2,3-octadiene (*rac*-3ak) (zwf-6-174)

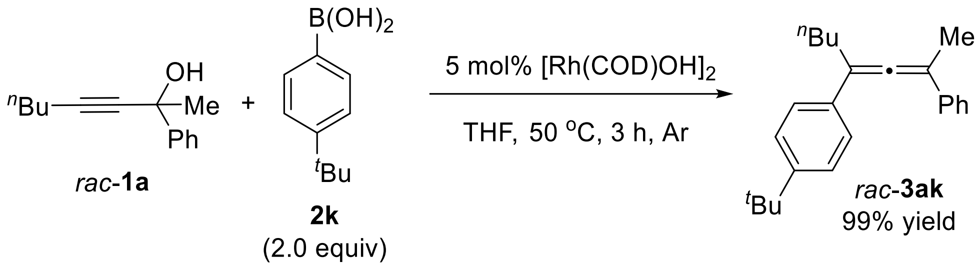

Following Typical Procedure I, the reaction of *rac*-**1a** (39.8 mg, 0.2 mmol), 4-*tert*-butylphenylboronic acid **2k** (71.6 mg, 0.4 mmol), $[Rh(COD)OH]_2$ (4.4 mg, 0.01 mmol) and THF (1 mL) afforded *rac*-**3ak**[2] (61.9 mg, 99%) [eluent: petroleum ether / ethyl acetate = 60/1 (~120 mL)]: oil; **^{1}H NMR** (400 MHz, $CDCl_3$): δ = 7.44 (d, *J* = 7.6 Hz, 2 H, Ar-H), 7.38-7.25 (m, 6 H, Ar-H), 7.22-7.14 (m, 1 H, Ar-H), 2.60-2.45 (m, 2 H, CH_2), 2.19 (s, 3 H, CH_3), 1.62-1.51 (m, 2 H, CH_2), 1.47-1.37 (m, 2 H, CH_2), 1.30 (s, 9 H, 3 x CH_3), 0.90 (t, *J* = 7.4 Hz, 3 H, CH_3); **^{13}C NMR** (100 MHz, $CDCl_3$): δ = 205.5, 149.7, 137.4, 134.0, 128.4, 126.5, 125.7, 125.6, 125.3, 107.5, 103.5, 34.4, 31.3, 30.2, 29.9, 22.6, 16.9, 14.0; **IR** (neat): *v* = 2954, 2922, 2853, 1930, 1492, 1465, 1363, 1268, 1028 cm^{-1}; **MS** (70 eV, EI) *m/z* (%): 318 (M^+, 1.68), 261 (100).

(11) Preparation of 2-phenyl-4-(4-phenylphenyl)-2,3-octadiene (*rac*-3al) (zwf-6-175)

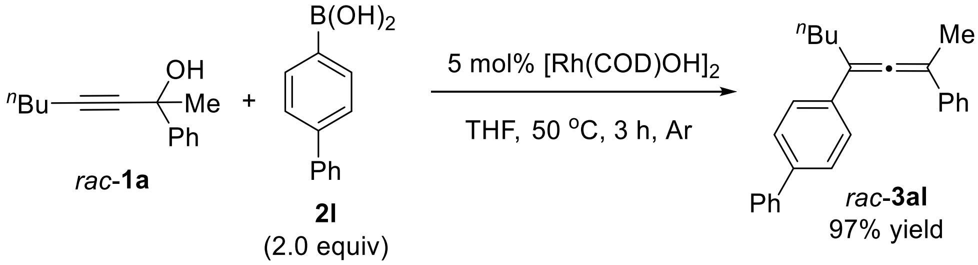

Following Typical Procedure I, the reaction of *rac*-**1a** (40.0 mg, 0.2 mmol), 4-biphenylboronic acid **2l** (79.4 mg, 0.4 mmol), $[Rh(COD)OH]_2$ (4.5 mg, 0.01 mmol) and THF (1 mL) afforded *rac*-**3al**[2] (64.8 mg, 97%) [eluent: petroleum ether = 320 mL]: oil; **^{1}H NMR** (400 MHz, $CDCl_3$): δ = 7.64-7.37 (m, 10 H, Ar-H), 7.36-7.26 (m, 3 H, Ar-H),

7.24-7.17 (m, 1 H, Ar-H), 2.65-2.50 (m, 2 H, CH_2), 2.22 (s, 3 H, CH_3), 1.65-1.54 (m, 2 H, CH_2), 1.48-1.37 (m, 2 H, CH_2), 0.92 (t, J = 7.4 Hz, 3 H, CH_3); **^{13}C NMR** (100 MHz, $CDCl_3$): δ = 205.8, 140.8, 139.5, 137.2, 136.0, 128.7, 128.4, 127.14, 127.13, 126.9, 126.7, 126.4, 125.7, 107.5, 103.7, 30.1, 30.0, 22.6, 16.9, 14.0; **IR** (neat): v = 3057, 2958, 2925, 2867, 1933, 1597, 1463, 1065, 1027 cm^{-1}; **MS** (70 eV, EI) *m/z* (%): 339 (M^++1, 4.78), 338 (M^+, 20.24), 281 (100).

(12) Preparation of 2-phenyl-4-(2-methylphenyl)-2,3-octadiene (*rac*-3am) (zwf-5-056)

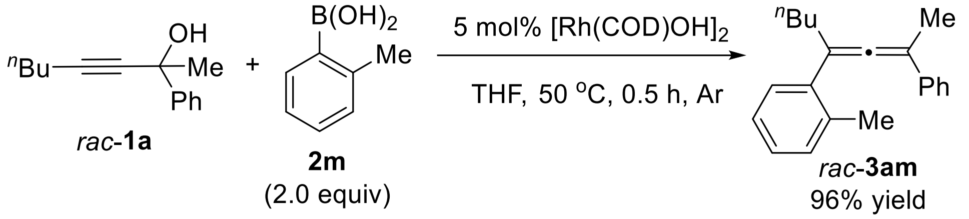

Following Typical Procedure I, the reaction of *rac*-**1a** (40.8 mg, 0.2 mmol), 2-methylphenylboronic acid **2m** (54.2 mg, 0.4 mmol), $[Rh(COD)OH]_2$ (4.8 mg, 0.01 mmol) and THF (1 mL) afforded *rac*-**3am**[2] (53.8 mg, 96%) [eluent: petroleum ether / ethyl acetate = 50/1 (~150 mL)]: oil; **^{1}H NMR** (400 MHz, $CDCl_3$): δ = 7.42 (d, J = 7.6 Hz, 2 H, Ar-H), 7.36-7.23 (m, 3 H, Ar-H), 7.23-7.06 (m, 4 H, Ar-H), 2.59-2.25 (m, 5 H, CH_2 and CH_3), 2.15 (s, 3 H, CH_3), 1.58-1.32 (m, 4 H, 2 x CH_2), 0.89 (t, J = 7.0 Hz, 3 H, CH_3); **^{13}C NMR** (100 MHz, $CDCl_3$): δ = 203.3, 138.1, 137.8, 135.8, 130.4, 128.2, 128.0, 126.7, 126.3, 125.74, 125.71, 106.7, 100.7, 34.0, 30.1, 22.5, 20.7, 17.1, 14.0; **IR** (neat): v = 2956, 2926, 2859, 1943, 1490, 1459, 1027 cm^{-1}; **MS** (70 eV, EI) *m/z* (%): 276 (M^+, 2.02), 115 (100).

(13) Preparation of 2-phenyl-4-(3-methylphenyl)-2,3-octadiene (*rac*-3an) (zwf-6-163)

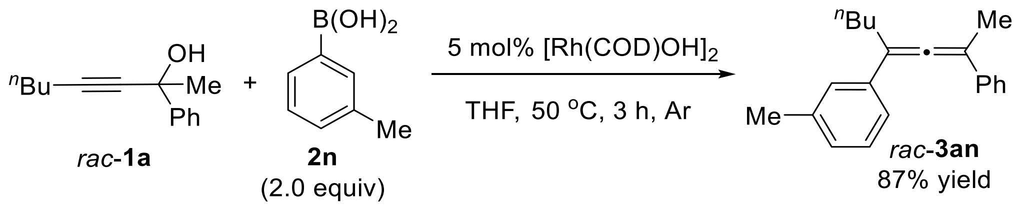

Following Typical Procedure I, the reaction of *rac*-**1a** (40.0 mg, 0.2 mmol), 3-methylphenylboronic acid **2n** (54.6 mg, 0.4 mmol), $[Rh(COD)OH]_2$ (4.5 mg, 0.01 mmol) and THF (1 mL) afforded *rac*-**3an**[2] (47.7 mg, 87%) [eluent: petroleum ether / ethyl acetate = 60/1 (~120 mL)]: oil; **^{1}H NMR** (400 MHz, $CDCl_3$): δ = 7.44 (d, J = 7.2 Hz, 2 H, Ar-H), 7.31 (t, J = 7.6 Hz, 2 H, Ar-H), 7.27-7.14 (m, 4 H, Ar-H), 7.01 (d, J = 7.2 Hz, 1 H, Ar-H), 2.61-2.45 (m, 2 H, CH_2), 2.32 (s, 3 H, CH_3), 2.20 (s, 3 H, CH_3), 1.62-1.50 (m, 2 H, CH_2), 1.47-1.34 (m, 2 H, CH_2), 0.90 (t, J = 7.2 Hz, 3 H, CH_3); **^{13}C NMR** (100 MHz, $CDCl_3$): δ = 205.5, 137.9, 137.3, 137.0, 128.4, 128.3, 127.5, 126.7, 126.6, 125.6, 123.2, 107.8, 103.4, 30.11, 30.05, 22.6, 21.5, 16.8, 14.0; **IR** (neat): v = 2955, 2925, 2858, 1934, 1600, 1463, 1026 cm^{-1}; **MS** (70 eV, EI) *m/z* (%): 277 (M^++1, 2.57), 276 (M^+, 11.17), 219 (100).

(14) Preparation of 2-phenyl-4-(4-methylphenyl)-2,3-octadiene (*rac*-3ao) (zwf-6-164)

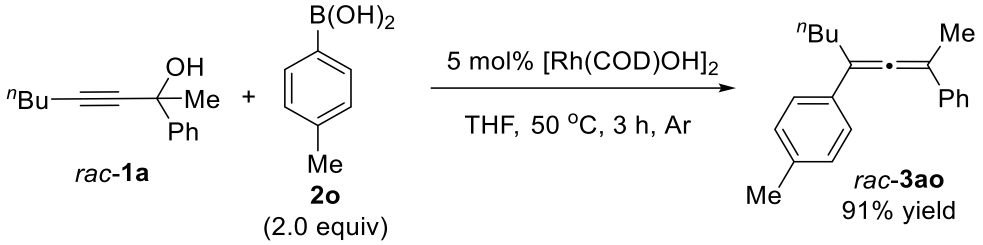

Following Typical Procedure I, the reaction of *rac*-**1a** (39.1 mg, 0.2 mmol), 4-methylphenylboronic acid **2o** (55.0 mg, 0.4 mmol), $[Rh(COD)OH]_2$ (4.5 mg, 0.01 mmol) and THF (1 mL) afforded *rac*-**3ao**[2] (48.7 mg, 91%) [eluent: petroleum ether / ethyl acetate = 60/1 (~120 mL)]: oil; **^{1}H NMR** (400 MHz, $CDCl_3$): δ = 7.43 (d, J = 8.0 Hz, 2 H, Ar-H), 7.36-7.23 (m, 4 H, Ar-H), 7.22-7.15 (m, 1 H, Ar-H), 7.11 (d, J = 8.0 Hz, 2 H, Ar-H), 2.60-2.44 (m, 2 H, CH_2), 2.32 (s, 3 H, CH_3), 2.19 (s, 3 H, CH_3), 1.62-1.51 (m, 2 H, CH_2), 1.47-1.35 (m, 2 H, CH_2), 0.90 (t, J = 7.4 Hz, 3 H, CH_3); **^{13}C NMR** (100 MHz, $CDCl_3$): δ = 205.3, 137.4, 136.4, 134.0, 129.1, 128.4, 126.5, 126.0, 125.6, 107.6, 103.4, 30.1, 30.0, 22.6, 21.0, 16.9, 14.0; **IR** (neat): v = 2957, 2928, 2858, 1928, 1595, 1491, 1445, 1026 cm^{-1}; **MS** (70 eV, EI) *m/z* (%): 276 (M^+, 10.12), 219 (100).

(15) Preparation of 2-phenyl-4-(1-naphthyl)-2,3-octadiene (*rac*-3ap) (zwf-7-098)

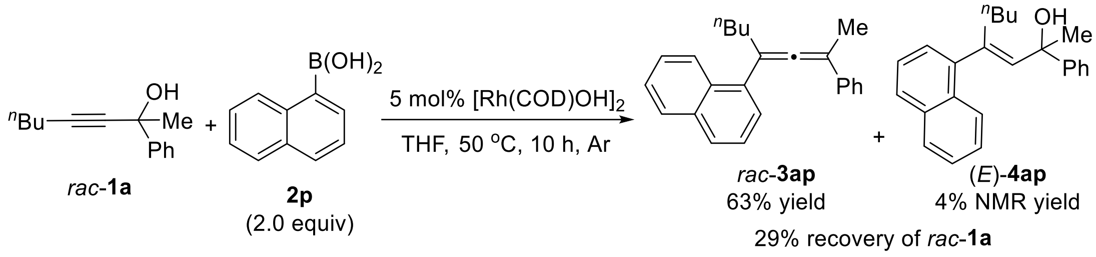

Following Typical Procedure I, the reaction of *rac*-**1a** (40.3 mg, 0.2 mmol), 1-naphthaleneboronic acid **2p** (68.1 mg, 0.4 mmol), $[Rh(COD)OH]_2$ (4.6 mg, 0.01 mmol) and THF (1 mL) afforded a mixture of *rac*-**3ap** (70% NMR yield), (*E*)-**4ap** (4% NMR yield), and 29% recovery of *rac*-**1a**, which was determined by ^{1}H NMR analysis of the crude product with CH_2Br_2 (14 uL) as the internal standard. The mixture was purified by chromatography on silica gel to afford the pure product *rac*-**3ap** (39.5 mg, 63%) [eluent: petroleum ether = 200 mL]: oil; ^{1}**H NMR** (400 MHz, $CDCl_3$): δ = 8.28-8.13 (m, 1 H, Ar-H), 7.88-7.77 (m, 1 H, Ar-H), 7.73 (d, J = 7.6 Hz, 1 H, Ar-H), 7.56-7.36 (m, 6 H, Ar-H), 7.31 (t, J = 7.6 Hz, 2 H, Ar-H), 7.17 (t, J = 7.2 Hz, 1 H, Ar-H), 2.70-2.45 (m, 2 H, CH_2), 2.20 (s, 3 H, CH_3), 1.64-1.50 (m, 2 H, CH_2), 1.47-1.34 (m, 2 H, CH_2), 0.89 (t, J = 7.2 Hz, 3 H, CH_3); ^{13}**C NMR** (100 MHz, $CDCl_3$): δ = 203.8, 137.8, 136.9, 134.0, 131.4, 128.4, 128.3, 127.3, 126.4, 125.8, 125.7, 125.6, 125.5, 125.4, 106.1, 100.9, 34.8, 30.3, 22.5, 17.2, 14.0; **IR** (neat): v = 3057, 2955, 2926, 2857, 1944, 1593, 1493, 1443, 1065, 1026 cm^{-1}; **MS** (70 eV, EI) m/z (%): 313 (M^++1, 8.07), 312 (M^+, 32.00), 255 (100); **HRMS** calcd m/z for $C_{24}H_{24}$ [M^+]: 312.1873, found: 312.1877.

(16) Preparation of 2-phenyl-4-(4-formylphenyl)-2,3-nonadiene (*rac*-3cb) (zwf-7-086)

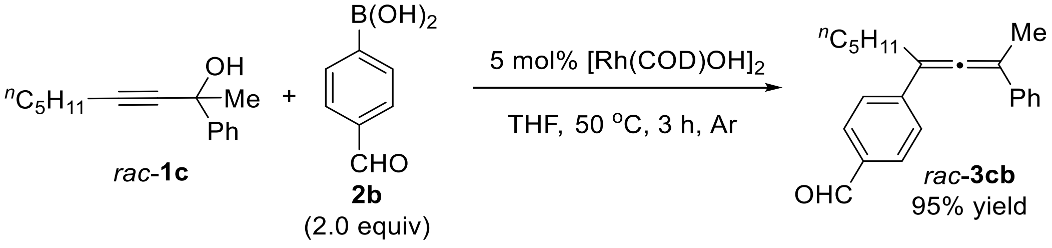

Following Typical Procedure I, the reaction of *rac*-**1c** (43.4 mg, 0.2 mmol), 4-

formylphenylboronic acid **2b** (60.0 mg, 0.4 mmol), $[Rh(COD)OH]_2$ (4.4 mg, 0.01 mmol) and THF (1 mL) afforded *rac*-**3cb** (58.1 mg, 95%) [eluent: petroleum ether / ethyl acetate = 50/1 (~200 mL)]: oil; **^{1}H NMR** (400 MHz, $CDCl_3$): δ = 9.96 (s, 1 H, CHO), 7.80 (d, *J* = 8.4 Hz, 2 H, Ar-H), 7.56 (d, *J* = 8.0 Hz, 2 H, Ar-H), 7.43 (d, *J* = 7.6 Hz, 2 H, Ar-H), 7.33 (t, *J* = 7.8 Hz, 2 H, Ar-H), 7.23 (t, *J* = 7.4 Hz, 1 H, Ar-H), 2.64-2.47 (m, 2 H, CH_2), 2.23 (s, 3 H, CH_3), 1.65-1.51 (m, 2 H, CH_2), 1.43-1.22 (m, 4 H, 2 x CH_2), 0.86 (t, *J* = 7.0 Hz, 3 H, CH_3); **^{13}C NMR** (100 MHz, $CDCl_3$): δ = 207.2, 191.7, 143.7, 136.3, 134.7, 129.9, 128.5, 127.0, 126.4, 125.7, 107.5, 104.5, 31.6, 30.0, 27.5, 22.5, 16.6, 14.0; **IR** (neat): *v* = 2953, 2926, 2857, 1929, 1697, 1599, 1211, 1026 cm^{-1}; **MS** (70 eV, EI) *m/z* (%): 305 (M^++1, 3.42), 304 (M^+, 13.21), 248 (100); **HRMS** calcd for *m/z* $C_{22}H_{24}O$ [M^+]: 304.1822, found: 304.1823.

(17) Preparation of 2-phenyl-4-(4-chlorophenyl)-2,3-decadiene (*rac*-3dh) (zwf-7-087)

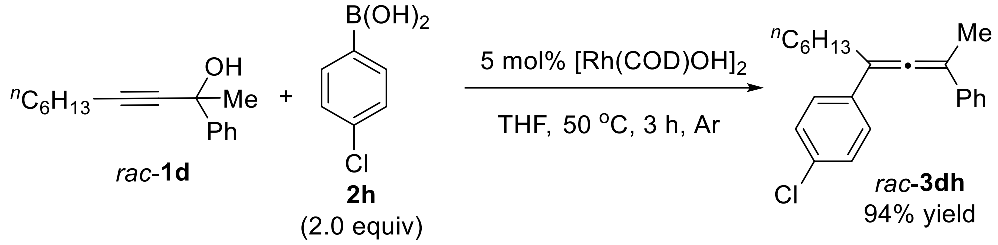

Following Typical Procedure I, the reaction of *rac*-**1d** (46.3 mg, 0.2 mmol), 4-chlorophenylboronic acid **2h** (62.1 mg, 0.4 mmol), $[Rh(COD)OH]_2$ (4.3 mg, 0.01 mmol) and THF (1 mL) afforded *rac*-**3dh** (61.4 mg, 94%) [eluent: petroleum ether / ethyl acetate = 50/1 (~200 mL)]: oil; **^{1}H NMR** (400 MHz, $CDCl_3$): δ = 7.42 (d, *J* = 7.6 Hz, 2 H, Ar-H), 7.36-7.28 (m, 4 H, Ar-H), 7.27-7.16 (m, 3 H, Ar-H), 2.57-2.42 (m, 2 H, CH_2), 2.20 (s, 3 H, CH_3), 1.60-1.51 (m, 2 H, CH_2), 1.43-1.31 (m, 2 H, CH_2), 1.31-1.18 (m, 4 H, CH_2), 0.85 (t, *J* = 7.0 Hz, 3 H, CH_3); **^{13}C NMR** (100 MHz, $CDCl_3$): δ = 205.5, 136.9, 135.6, 132.3, 128.5, 128.4, 127.3, 126.8, 125.6, 107.0, 104.0, 31.7, 30.2, 29.2, 27.8, 22.6, 16.8, 14.0; **IR** (neat): *v* = 2953, 2926, 2857, 1933, 1597, 1489, 1092, 1011 cm^{-1}; **MS** (70 eV, EI) *m/z* (%): 326 (M^+(^{37}Cl), 2.05), 324 (M^+(^{35}Cl), 7.03), 239 (100); **HRMS** calcd *m/z* for $C_{22}H_{25}{}^{35}Cl$ [M^+]: 324.1639, found: 324.1640.

(18) Preparation of 2,4-diphenyl-8-chloro-2,3-octadiene (*rac*-3ea) (zwf-6-192)

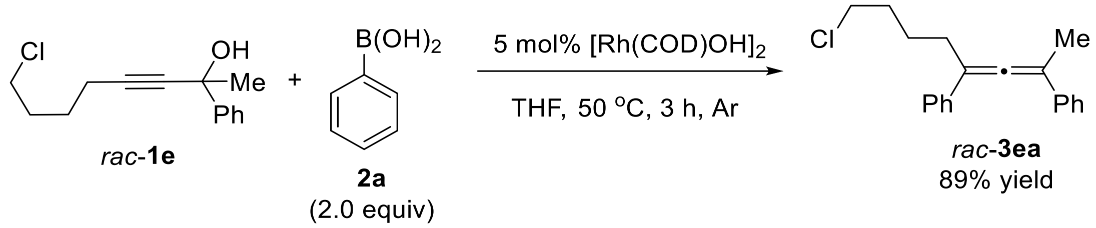

Following Typical Procedure I, the reaction of *rac*-**1e** (47.6 mg, 0.2 mmol), phenylboronic acid **2a** (48.7 mg, 0.4 mmol), $[Rh(COD)OH]_2$ (4.5 mg, 0.01 mmol) and THF (1 mL) afforded *rac*-**3ea**[2] (53.1 mg, 89%) [eluent: petroleum ether = 320 mL]: oil; **^{1}H NMR** (400 MHz, $CDCl_3$): δ = 7.44-7.33 (m, 4 H, Ar-H), 7.33-7.22 (m, 4 H, Ar-H), 7.24-7.16 (m, 2 H, Ar-H), 3.51 (t, *J* = 6.8 Hz, 2 H, CH_2), 2.63-2.50 (m, 2 H, CH_2), 2.21 (s, 3 H, CH_3), 1.93-1.80 (m, 2 H, CH_2), 1.80-1.65 (m, 2 H, CH_2); **^{13}C NMR** (100 MHz, $CDCl_3$): δ = 205.4, 137.0, 136.7, 128.5, 128.4, 126.84, 126.80, 126.0, 125.6, 107.2, 104.1, 44.8, 32.3, 29.4, 25.1, 16.9; **IR** (neat): v = 2940, 2863, 1933, 1596, 1444, 1065, 1026 cm^{-1}; **MS** (70 eV, EI) *m/z* (%): 298 (M^+(^{37}Cl), 2.66), 296 (M^+(^{35}Cl), 7.49), 205 (100).

(19) Preparation of 2-phenyl-4-(4-acetylphenyl)-8-chloro-2,3-octadiene (*rac*-3ec) (zwf-7-092)

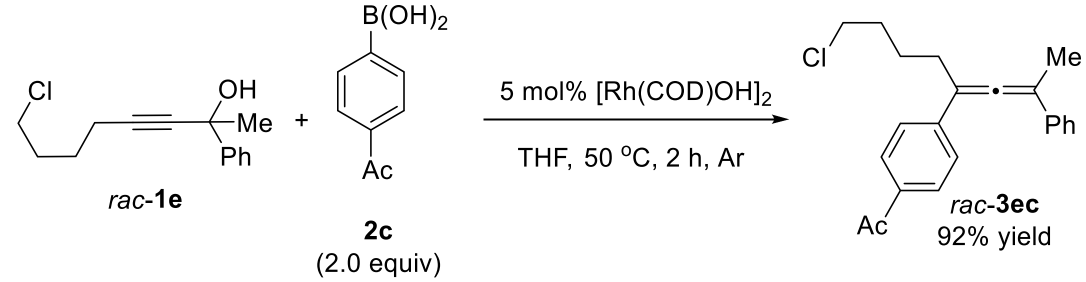

Following Typical Procedure I, the reaction of *rac*-**1e** (47.1 mg, 0.2 mmol), 4-acetylphenylboronic acid **2c** (64.8 mg, 0.4 mmol), $[Rh(COD)OH]_2$ (4.7 mg, 0.01 mmol) and THF (1 mL) afforded *rac*-**3ec** (61.8 mg, 92%) [eluent: petroleum ether / ethyl acetate = 50/1 (~200 mL), 20/1 (~210 mL)]: oil; **^{1}H NMR** (400 MHz, $CDCl_3$): δ = 7.90 (d, *J* = 8.8 Hz, 2 H, Ar-H), 7.49 (d, *J* = 8.4 Hz, 2 H, Ar-H), 7.42 (d, *J* = 7.6 Hz, 2 H, Ar-H), 7.33 (t, *J* = 7.6 Hz, 2 H, Ar-H), 7.23 (t, *J* = 7.2 Hz, 1 H, Ar-H), 3.52 (t, *J* = 6.6 Hz,

2 H, CH_2), 2.67-2.45 (m, 5 H, CH_2 and CH_3), 2.24 (s, 3 H, CH_3), 1.95-1.81 (m, 2 H, CH_2), 1.80-1.67 (m, 2 H, CH_2); **^{13}C NMR** (100 MHz, $CDCl_3$): δ = 206.7, 197.5, 141.8, 136.3, 135.4, 128.6, 128.5, 127.1, 126.0, 125.7, 106.9, 104.8, 44.7, 32.2, 29.2, 26.5, 25.0, 16.7; **IR** (neat): *v* = 2941, 1931, 1678, 1599, 1358, 1265, 1026 cm^{-1}; **MS** (70 eV, EI) *m/z* (%): 340 (M^+(^{37}Cl), 9.39), 338 (M^+(^{35}Cl), 27.50), 247 (100); **HRMS** calcd *m/z* for $C_{22}H_{23}{}^{35}ClO$ [M^+]: 338.1432, found: 338.1430.

(20) Preparation of 2,6-diphenyl-4-(3-chlorophenyl)-2,3-hexadiene (*rac*-3fq) (zwf-5-045)

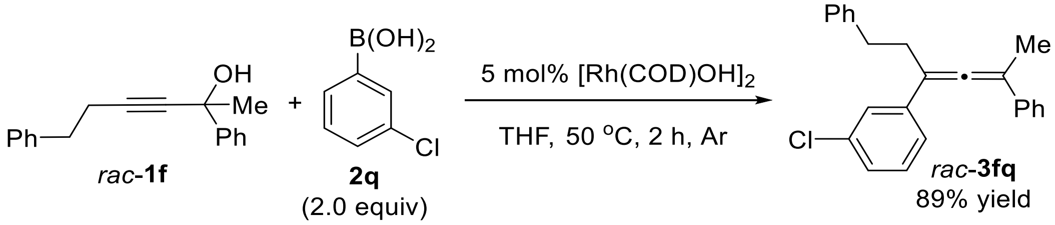

Following Typical Procedure I, the reaction of *rac*-**1f** (50.2 mg, 0.2 mmol), 3-chlorophenylboronic acid **2q** (62.5 mg, 0.4 mmol), $[Rh(COD)OH]_2$ (4.5 mg, 0.01 mmol) and THF (1 mL) afforded *rac*-**3fq** (61.7 mg, 89%) [eluent: petroleum ether / ethyl acetate = 50/1 (~150 mL)]: oil; **^{1}H NMR** (400 MHz, $CDCl_3$): δ = 7.48-7.06 (m, 14 H, Ar-H), 2.96-2.75 (m, 4 H, 2 x CH_2), 2.09 (s, 3 H, CH_3); **^{13}C NMR** (100 MHz, $CDCl_3$): δ = 205.8, 141.5, 138.9, 136.5, 134.5, 126.9, 128.5, 128.44, 128.35, 126.9, 126.8, 126.0, 125.9, 125.7, 124.2, 106.3, 104.8, 33.9, 31.8, 16.7; **IR** (neat): *v* = 3060, 3026, 2919, 2857, 1935, 1591, 1493, 1079, 1025 cm^{-1}; **MS** (70 eV, EI) *m/z* (%): 346 (M^+(^{37}Cl), 29.54), 344 (M(^{35}Cl), 82.22), 91 (100); **HRMS** calcd *m/z* for $C_{24}H_{21}{}^{35}Cl$ [M^+]: 344.1326, found: 344.1327.

(21) Preparation of 2-(thiophen-3-yl)-4-phenyl-2,3-octadiene (*rac*-3ga) (zwf-6-193)

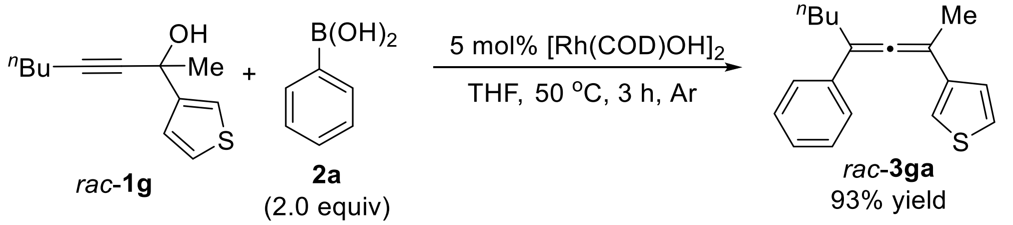

Following Typical Procedure I, the reaction of *rac*-**1g** (41.8 mg, 0.2 mmol),

phenylboronic acid **2a** (48.5 mg, 0.4 mmol), [Rh(COD)OH]$_2$ (4.6 mg, 0.01 mmol) and THF (1 mL) afforded *rac*-**3ga** (51.6 mg, 93%, 97% purity) [eluent: petroleum ether = 320 mL]: oil; **^{1}H NMR** (400 MHz, CDCl$_3$): δ = 7.40 (d, *J* = 8.0 Hz, 2 H, Ar-H), 7.28 (t, *J* = 7.6 Hz, 2 H, Ar-H), 7.21-7.14 (m, 2 H, Ar-H and one proton from thienyl), 7.12-7.05 (m, 2 H, two proton from thienyl), 2.59-2.43 (m, 2 H, CH$_2$), 2.16 (s, 3 H, CH$_3$), 1.61-1.48 (m, 2 H, CH$_2$), 1.47-1.34 (m, 2 H, CH$_2$), 0.91 (t, *J* = 7.2 Hz, 3 H, CH$_3$); **^{13}C NMR** (100 MHz, CDCl$_3$): δ = 205.3, 139.6, 137.1, 128.4, 126.7, 126.6, 126.2, 125.4, 118.9, 107.2, 100.0, 30.14, 30.07, 22.6, 17.4, 14.0; **IR** (neat): *v* = 2955, 2925, 2858, 1934, 1597, 1446, 1245, 1181, 1026 cm^{-1}; **MS** (70 eV, EI) *m/z* (%): 269 (M$^+$+1, 2.96), 268 (M$^+$, 14.23), 211 (100); **HRMS** calcd *m/z* for $C_{18}H_{20}S$ [M$^+$]: 268.1280, found: 268.1283.

(22) Preparation of 2-(2-methylphenyl)-4-(4-bromophenyl)-2,3-octadiene (*rac*-3hi) (zwf-7-007)

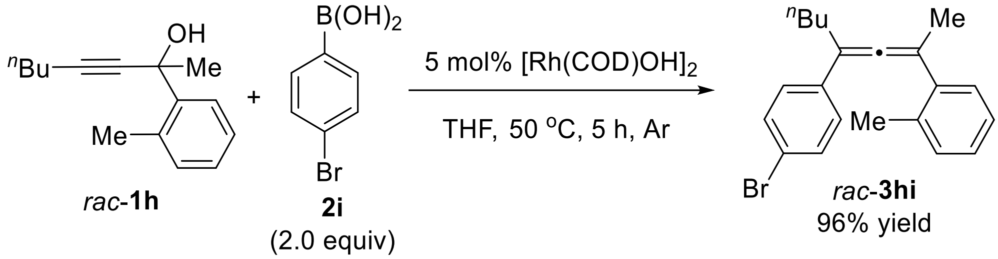

rac-**1h** **2i** (2.0 equiv) *rac*-**3hi** 96% yield

Following Typical Procedure I, the reaction of *rac*-**1h** (43.0 mg, 0.2 mmol), 4-bromophenylboronic acid **2i** (80.2 mg, 0.4 mmol), [Rh(COD)OH]$_2$ (4.5 mg, 0.01 mmol) and THF (1 mL) afforded *rac*-**3hi** (67.7 mg, 96%) [eluent: petroleum ether / ethyl acetate = 60/1 (~120 mL)]: oil; **^{1}H NMR** (400 MHz, CDCl$_3$): δ = 7.41 (d, *J* = 8.4 Hz, 2 H, Ar-H), 7.32-7.22 (m, 3 H, Ar-H), 7.21-7.08 (m, 3 H, Ar-H), 2.41 (t, *J* = 7.6 Hz, 2 H, CH$_2$), 2.31 (s, 3 H, CH$_3$), 2.15 (s, 3 H, CH$_3$), 1.59-1.50 (m, 2 H, CH$_2$), 1.44-1.32 (m, 2 H, CH$_2$), 0.92 (t, *J* = 7.2 Hz, 3 H, CH$_3$); **^{13}C NMR** (100 MHz, CDCl$_3$): δ = 204.0, 138.1, 136.8, 135.7, 131.3, 130.6, 127.9, 127.6, 126.9, 125.9, 120.2, 104.1, 103.3, 30.1, 30.0, 22.5, 20.8, 20.5, 14.0; **IR** (neat): *v* = 2954, 2925, 2858, 1943, 1460, 1105, 1043, 1007 cm^{-1}; **MS** (70 eV, EI) *m/z* (%): 356 (M$^+$(^{81}Br), 1.85), 354 (M$^+$(^{79}Br), 1.77), 218 (100); **HRMS** calcd *m/z* for $C_{21}H_{23}{}^{79}Br$ [M$^+$]: 354.0978, found: 354.0980.

(23) Preparation of 2-(3-methoxyphenyl)-4-(4-chlorophenyl)-2,3-octadiene (*rac*-3ih) (zwf-7-008)

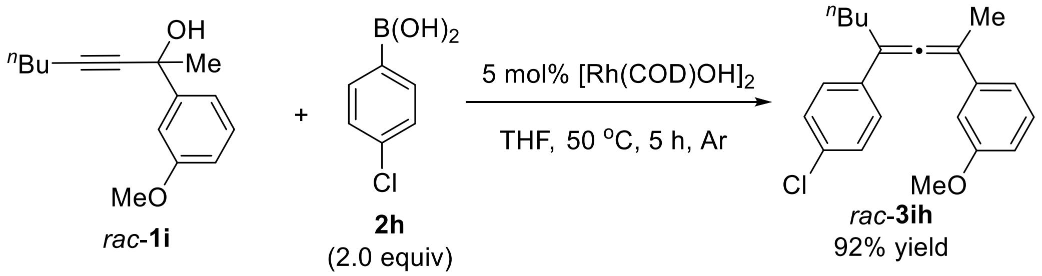

Following Typical Procedure I, the reaction of *rac*-**1i** (46.2 mg, 0.2 mmol), 4-chlorophenylboronic acid **2h** (62.8 mg, 0.4 mmol), [Rh(COD)OH]$_2$ (4.6 mg, 0.01 mmol) and THF (1 mL) afforded *rac*-**3ih** (59.9 mg, 92%) [eluent: petroleum ether / ethyl acetate = 60/1 (~120 mL)]: oil; **^{1}H NMR** (400 MHz, CDCl$_3$): δ = 7.32 (d, J = 8.8 Hz, 2 H, Ar-H), 7.28-7.16 (m, 3 H, Ar-H), 7.02 (d, J = 7.6 Hz, 1 H, Ar-H), 7.00-6.94 (m, 1 H, Ar-H), 6.77 (dd, J_1 = 8.0 Hz, J_2 = 2.0 Hz, 1 H, Ar-H), 3.77 (s, 3 H, OCH$_3$), 2.59-2.40 (m, 2 H, CH$_2$), 2.18 (s, 3 H, CH$_3$), 1.60-1.49 (m, 2 H, CH$_2$), 1.47-1.33 (m, 2 H, CH$_2$), 0.90 (t, J = 7.2 Hz, 3 H, CH$_3$); **^{13}C NMR** (100 MHz, CDCl$_3$): δ = 205.6, 159.8, 138.5, 135.5, 132.3, 129.3, 128.5, 127.3, 118.2, 111.9, 111.7, 107.0, 103.9, 55.2, 30.0, 29.9, 22.6, 16.8, 13.9; **IR** (neat): v = 2955, 2927, 2859, 1933, 1603, 1488, 1322, 1046, 1011 cm^{-1}; **MS** (70 eV, EI) *m/z* (%): 328 (M$^+$(^{37}Cl), 6.54), 326 (M$^+$(^{35}Cl), 18.32), 269 (100); **HRMS** calcd m/z for $C_{21}H_{23}{}^{35}ClO$[M$^+$]: 326.1432, found: 326.1435.

(24) Preparation of 2-(4-bromophenyl)-4-(4-formylphenyl)-2,3-octadiene (*rac*-3jb) (zwf-7-009)

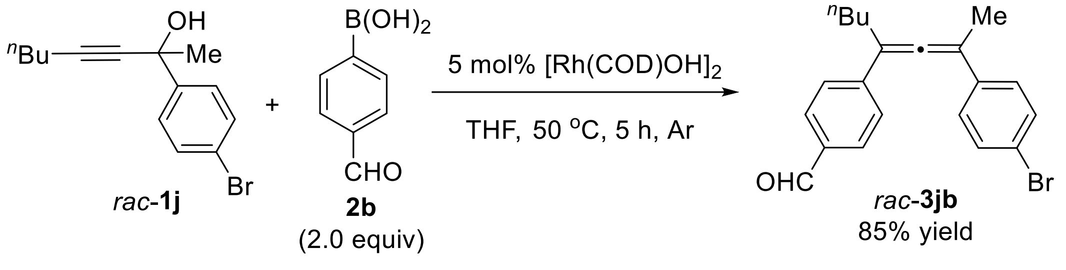

Following Typical Procedure I, the reaction of *rac*-**1j** (56.1 mg, 0.2 mmol), 4-formylphenylboronic acid **2b** (60.1 mg, 0.4 mmol), [Rh(COD)OH]$_2$ (4.5 mg, 0.01

mmol) and THF (1 mL) afforded *rac*-**3jb** (62.9 mg, 85%) [eluent: petroleum ether / ethyl acetate = 50/1 (~200 mL)]: oil; **^{1}H NMR** (400 MHz, $CDCl_3$): δ = 9.97 (s, 1 H, CHO), 7.81 (d, *J* = 8.0 Hz, 2 H, Ar-H), 7.54 (d, *J* = 8.4 Hz, 2 H, Ar-H), 7.44 (d, *J* = 8.4 Hz, 2 H, Ar-H), 7.27 (d, *J* = 8.4 Hz, 2 H, Ar-H), 2.57 (t, *J* = 7.6 Hz, 2 H, CH_2), 2.21 (s, 3 H, CH_3), 1.61-1.49 (m, 2 H, CH_2), 1.49-1.36 (m, 2 H, CH_2), 0.92 (t, *J* = 7.2 Hz, 3 H, CH_3); **^{13}C NMR** (100 MHz, $CDCl_3$): δ = 207.1, 191.6, 143.3, 135.4, 134.9, 131.6, 129.9, 127.3, 126.5, 120.9, 108.0, 103.8, 30.0, 29.7, 22.5, 16.6, 13.9; **IR** (neat): *v* = 2955, 2926, 2732, 1929, 1698, 1601, 1485, 1212, 1168, 1076, 1007 cm^{-1}; **MS** (70 eV, EI) *m/z* (%): 370 (M^+(^{81}Br), 6.27), 368 (M^+(^{79}Br), 6.24), 247 (100); **HRMS** calcd *m/z* for $C_{21}H_{21}{}^{79}BrO$ [M^+]: 368.0770, found: 368.0769.

(25) Preparation of 3-(2-methylphenyl)-5-(4-chlorophenyl)-3,4-octadiene (*rac*-3kh) (zwf-5-046)

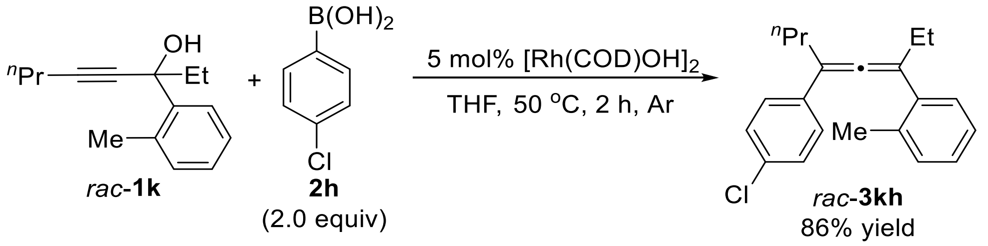

Following Typical Procedure I, the reaction of *rac*-**1k** (43.2 mg, 0.2 mmol), 4-chlorophenylboronic acid **2h** (62.4 mg, 0.4 mmol), $[Rh(COD)OH]_2$ (4.7 mg, 0.01 mmol) and THF (1 mL) afforded *rac*-**3kh** (53.5 mg, 86%) [eluent: petroleum ether / ethyl acetate = 50/1 (~150 mL)]: oil; **^{1}H NMR** (400 MHz, $CDCl_3$): δ = 7.35 (d, *J* = 8.4 Hz, 2 H, Ar-H), 7.30-7.20 (m, 3 H, Ar-H), 7.20-7.06 (m, 3 H, Ar-H), 2.52-2.34 (m, 4 H, 2 x CH_2), 2.30 (s, 3 H, CH_3), 1.59 (sextet, *J* = 7.4 Hz, 2 H, CH_2), 1.12 (t, *J* = 7.4 Hz, 3 H, CH_3), 0.98 (t, *J* = 7.2 Hz, 3 H, CH_3); **^{13}C NMR** (100 MHz, $CDCl_3$): δ = 202.4, 138.2, 136.3, 135.7, 132.0, 130.4, 128.3, 128.2, 127.3, 126.9, 125.7, 110.2, 105.9, 32.6, 27.5, 21.4, 20.6, 14.1, 12.6; **IR** (neat): *v* = 2962, 2930, 2895, 1946, 1484, 1455, 1089, 1007 cm^{-1}; **MS** (70 eV, EI) *m/z* (%): 312 (M^+(^{37}Cl), 0.76), 310 (M(^{35}Cl), 2.27), 267 (100); **HRMS** calcd *m/z* for $C_{21}H_{23}{}^{35}Cl$ [M^+]: 310.1483, found: 310.1485.

(26) Preparation of 2-phenyl-4-(3-(methoxycarbonyl)phenyl)-2,3-octadiene (*rac*-3ar) (wj-4-154)

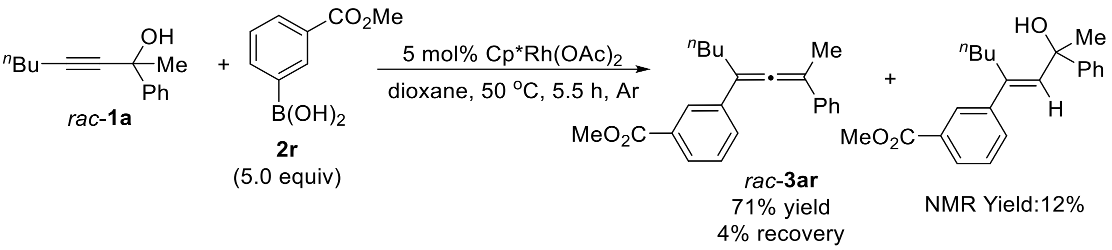

To an oven-dried Schlenk tube (25 mL) were added 3-(methoxycarbonyl)phenylboronic acid **2r** (74.1 mg, 0.4 mmol) and Cp*Rh(OAc)$_2$ (3.7 mg, 0.01 mmol). Then the flask was degassed and refilled with Ar for three times followed by the addition of *rac*-**1a** (40.5 mg, 0.2 mmol) and dioxane (1 mL). After being stirred at 50 °C for 1 h, **2r** (74.2 mg and 37.1 mg) was added; after being stirred for 2 h, **2r** (37.1 mg) was added. The resulting mixture was vigorously stirred at 50 °C for another 2.5 h as monitored by TLC, diluted with ethyl acetate (1 mL), filtered through a short column of silica gel (3 cm), eluted with ethyl acetate (10 mL), and concentrated. The residue was purified by chromatography on silica gel to afford *rac*-**3ar** (45.5 mg, 71%) [eluent: petroleum ether / ethyl ether = 50:1 (150 mL)]; oil; **^{1}H NMR** (400 MHz, CDCl$_3$): δ = 8.07 (t, J = 1.8 Hz, 1 H, Ar-H), 7.87 (dt, J_1 = 8.0 Hz, J_2 = 1.4 Hz, 1 H, Ar-H), 7.61 (dt, J_1 = 8.0 Hz, J_2 = 1.4 Hz, 1 H, Ar-H), 7.47-7.40 (m, 2 H, Ar-H), 7.38-7.28 (m, 3 H, Ar-H), 7.24-7.18 (m, 1 H, Ar-H), 3.91 (s, 3 H, CH$_3$), 2.66-2.51 (m, 2 H, CH$_2$), 2.23 (s, 3 H, CH$_3$), 1.59-1.51 (m, 2 H, CH$_2$), 1.48-1.38 (m, 2 H, CH$_2$), 0.91 (t, J = 7.4 Hz, 3 H, CH$_3$); **^{13}C NMR** (100 MHz, CDCl$_3$): δ = 205.6, 167.2, 137.6, 136.9, 130.9, 130.4, 128.41, 128.38, 127.8, 126.8, 126.6, 125.7, 107.3, 104.2, 52.1, 30.0, 29.9, 22.5, 16.8, 14.0; **IR** (neat): v = 2953, 2929, 1933, 1722, 1599, 1582, 1492, 1439, 1281, 1253, 1212, 1114, 1084, 1026 cm^{-1}; **MS** (70 eV, EI) *m/z* (%): 320 (M$^+$, 10.42), 263 (100); **HRMS** calcd for $C_{22}H_{24}O_2$ [M$^+$]: 320.1771, found: 320.1771.

1.3. Preparation of chiral allenes

Conditions A for exclusive *anti-β*-OH elimination

(1) Preparation of (*R*)-2,4-diphenyl-2,3-octadiene ((*R*)-3aa) (zwf-4-141 and zwf-6-

200-1)

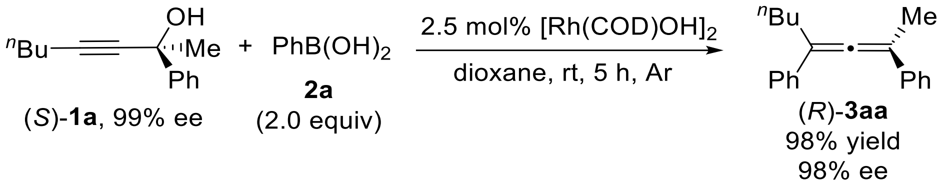

Typical Procedure II: To an oven-dried Schlenk tube (25 mL) was added phenylboronic acid **2a** (48.9 mg, 0.4 mmol), which was then transferred to a glovebox. After adding $[Rh(COD)OH]_2$ (2.4 mg, 0.005 mmol) in glovebox, it was transferred out of the glovebox. After replacing nitrogen with argon for three times by vacuum, (*S*)-**1a** (40.3 mg, 0.2 mmol, 99% ee) and freshly distilled dioxane (1 mL) were added. The resulting mixture was vigorously stirred at rt for 5 h as monitored by TLC, diluted with ethyl acetate (1 mL), filtered through a short column of silica gel (1 cm), eluted with ethyl acetate (5 mL), and concentrated. The residue was purified by chromatography on silica gel to afford (*R*)-**3aa** (51.4 mg, 98%) [eluent: petroleum ether / ethyl acetate = 60/1 (~120 mL)]: 98% ee (HPLC conditions: OJ-H column, hexane/*i*-PrOH = 99.5/0.5, 0.7 mL/min, λ = 214 nm, t_R (major) = 6.3 min, t_R (minor) = 8.0 min); $[\alpha]_D^{27}$ = -326.1 (*c* = 1.17, $CHCl_3$); oil; **^{1}H NMR** (400 MHz, $CDCl_3$): δ = 7.43 (t, *J* = 7.2 Hz, 4 H, Ar-H), 7.29 (m, 4 H, Ar-H), 7.23-7.12 (m, 2 H, Ar-H), 2.62-2.47 (m, 2 H, CH_2), 2.20 (s, 3 H, CH_3), 1.64-1.49 (m, 2 H, CH_2), 1.49-1.35 (m, 2 H, CH_2), 0.90 (t, *J* = 7.4 Hz, 3 H, CH_3); **^{13}C NMR** (100 MHz, $CDCl_3$): δ = 205.5, 137.2, 137.0, 128.41, 128.37, 126.7, 126.6, 126.0, 125.6, 107.8, 103.6, 30.1, 30.0, 22.6, 16.8, 14.0; **IR** (neat): ν = 3058, 2954, 2925, 2858, 1932, 1596, 1491, 1443, 1025 cm^{-1}; **MS** (70 eV, EI) *m/z* (%): 263 (M^++1, 1.21), 262 (M^+, 5.56), 205 (100); **HRMS** calcd for $C_{20}H_{22}$ [M^+]: 262.1716, found: 262.1725.

(2) Preparation of (*R*)-2-phenyl-4-(4-formylphenyl)-2,3-octadiene ((*R*)-3ab) (zwf-5-053 and zwf-7-034)

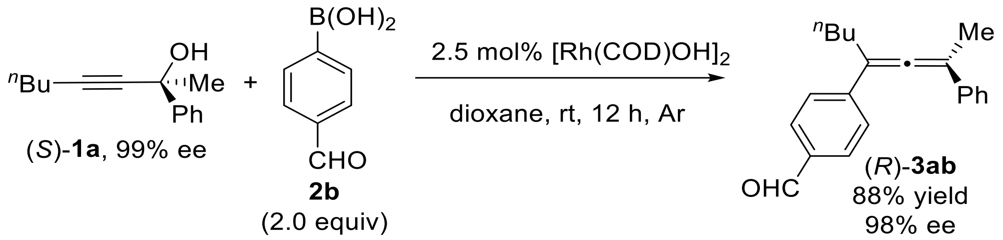

Following Typical Procedure II, (*S*)-**1a** (40.0 mg, 0.2 mmol, 99% ee), 4-formylphenylboronic acid **2b** (60.5 mg, 0.4 mmol), [Rh(COD)OH]$_2$ (2.3 mg, 0.005 mmol) and dioxane (1 mL) afforded (*R*)-**3ab** (50.3 mg, 88%) [eluent: petroleum ether / ethyl acetate = 60/1 (~240 mL)]: 98% ee (HPLC conditions: OD-H column, hexane/*i*-PrOH = 99.5/0.5, 1.0 mL/min, λ = 214 nm, t_R (minor) = 10.3 min, t_R (major) = 17.2 min); oil; **^{1}H NMR** (400 MHz, $CDCl_3$): δ = 9.96 (s, 1 H, CHO), 7.81 (d, *J* = 8.0 Hz, 2 H, Ar-H), 7.57 (d, *J* = 8.0 Hz, 2 H, Ar-H), 7.43 (d, *J* = 7.6 Hz, 2 H, Ar-H), 7.33 (t, *J* = 7.4 Hz, 2 H, Ar-H), 7.27-7.16 (m, 1 H, Ar-H), 2.64-2.50 (m, 2 H, CH_2), 2.24 (s, 3 H, CH_3), 1.63-1.51 (m, 2 H, CH_2), 1.50-1.34 (m, 2 H, CH_2), 0.92 (t, *J* = 7.2 Hz, 3 H, CH_3); **^{13}C NMR** (100 MHz, $CDCl_3$): δ = 207.2, 191.7, 143.7, 136.3, 134.7, 129.9, 128.5, 127.0, 126.4, 125.7, 107.5, 104.5, 30.0, 29.8, 22.5, 16.6, 13.9; **IR** (neat): *v* = 2956, 2927, 1930, 1698, 1599, 1212, 1168 cm^{-1}; **MS** (70 eV, EI) *m/z* (%): 291 (M^++1, 3.96), 290 (M^+, 15.61), 233 (100); **HRMS** calcd *m/z* for $C_{21}H_{22}O$ [M^+]: 290.1665, found: 290.1666.

(3) Preparation of (*R*)-2-phenyl-4-(4-acetylphenyl)-2,3-octadiene ((*R*)-3ac) (zwf-6-185 and zwf-7-038)

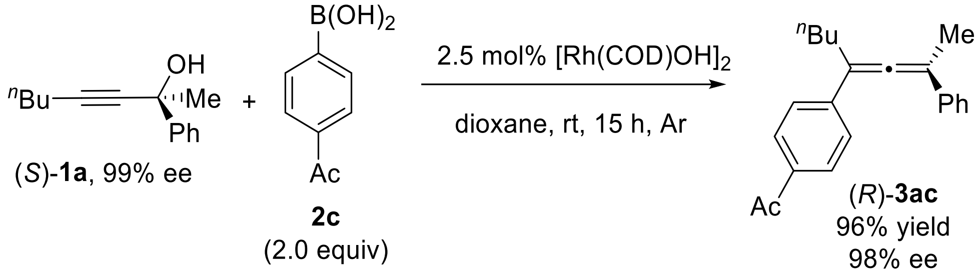

Following Typical Procedure II, the reaction of (*S*)-**1a** (40.4 mg, 0.2 mmol, 99% ee), 4-acetylphenylboronic acid **2c** (65.8 mg, 0.4 mmol), [Rh(COD)OH]$_2$ (2.4 mg, 0.005 mmol) and dioxane (1 mL) afforded (*R*)-**3ac** (58.7 mg, 96%) [eluent: petroleum ether / ethyl acetate = 60/1 (~250 mL)]: 98% ee (HPLC conditions: OD-H column, hexane/*i*-PrOH = 99.5/0.5, 1.0 mL/min, λ = 214 nm, t_R (minor) = 10.7 min, t_R (major) = 15.7

min); oil; ^{1}**H NMR** (400 MHz, $CDCl_3$): δ = 7.89 (d, *J* = 8.4 Hz, 2 H, Ar-H), 7.50 (d, *J* = 8.4 Hz, 2 H, Ar-H), 7.43 (d, *J* = 7.6 Hz, 2 H, Ar-H), 7.33 (t, *J* = 7.6 Hz, 2 H, Ar-H), 7.26-7.20 (m, 1 H, Ar-H), 2.64-2.48 (m, 5 H, CH_2 and CH_3), 2.23 (s, 3 H, CH_3), 1.62-1.50 (m, 2 H, CH_2), 1.48-1.37 (m, 2 H, CH_2), 0.92 (t, *J* = 7.4 Hz, 3 H, CH_3); ^{13}**C NMR** (100 MHz, $CDCl_3$): δ = 206.8, 197.6, 142.2, 136.6, 135.3, 128.6, 128.5, 127.0, 126.0, 125.7, 107.5, 104.3, 30.0, 29.8, 26.5, 22.5, 16.7, 13.9; **IR** (neat): *ν* = 2955, 2927, 2859, 1930, 1680, 1600, 1265, 1026 cm^{-1}; **MS** (70 eV, EI) *m/z* (%): 305 (M^++1, 7.14), 304 (M^+, 30.03), 247 (100); **HRMS** calcd *m/z* for $C_{22}H_{24}O$ [M^+]: 304.1822, found: 304.1821.

(4) Preparation of (*R*)-2-phenyl-4-(4-(methoxycarbonyl)phenyl)-2,3-octadiene ((*R*)-3ad) (zwf-5-054 and zwf-7-039)

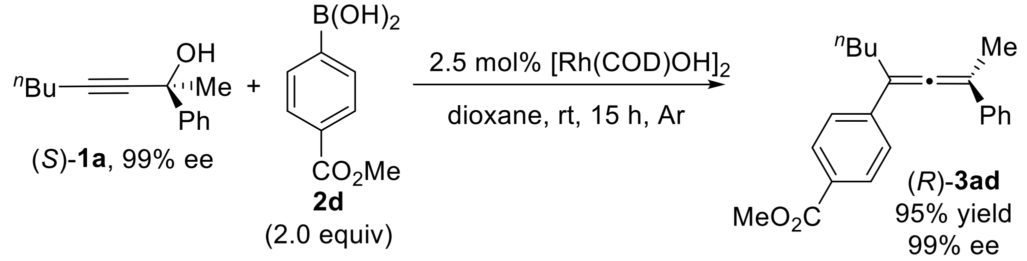

Following Typical Procedure II, (*S*)-**1a** (40.1 mg, 0.2 mmol, 99% ee), 4-(methoxycarbonyl)phenylboronic acid **2d** (71.6 mg, 0.4 mmol), $[Rh(COD)OH]_2$ (2.5 mg, 0.005 mmol) and dioxane (1 mL) afforded (*R*)-**3ad** (60.2 mg, 95%) [eluent: petroleum ether / ethyl acetate = 100/1 (~200 mL)]: 99% ee (HPLC conditions: OD-H column, hexane/*i*-PrOH = 99.5/0.5, 1.0 mL/min, λ = 214 nm, t_R (minor) = 6.6 min, t_R (major) = 9.3 min); $[\alpha]_D^{21}$ = -477.0 (*c* = 1.35, $CHCl_3$); white solid; m.p. 70.3-71.7 °C (petroleum ether/DCM); ^{1}**H NMR** (400 MHz, $CDCl_3$): δ = 7.96 (d, *J* = 8.4 Hz, 2 H, Ar-H), 7.54-7.38 (m, 4 H, Ar-H), 7.33 (t, *J* = 7.6 Hz, 2 H, Ar-H), 7.27-7.19 (m, 1 H, Ar-H), 3.90 (s, 3 H, OCH_3), 2.64-2.47 (m, 2 H, CH_2), 2.22 (s, 3 H, CH_3), 1.62-1.51 (m, 2 H, CH_2), 1.49-1.34 (m, 2 H, CH_2), 0.91 (t, *J* = 7.2 Hz, 3 H, CH_3); ^{13}**C NMR** (100 MHz, $CDCl_3$): δ = 206.6, 166.9, 142.0, 136.6, 129.7, 128.4, 128.1, 126.9, 125.8, 125.6, 107.5, 104.2, 51.9, 30.0, 29.8, 22.5, 16.7, 13.9; **IR** (neat): *ν* = 2956, 2924, 2862, 1929, 1714, 1603, 1437, 1272, 1107 cm^{-1}; **MS** (70 eV, EI) *m/z* (%): 321 (M^++1, 2.56), 320 (M^+, 9.61), 263 (100); Anal. Calcd. for $C_{22}H_{24}O_2$: C 82.46, H 7.55; found C 82.36, H 7.57.

(5) Preparation of (*R*)-2-phenyl-4-(4-nitrophenyl)-2,3-octadiene ((*R*)-3ae) (zwf-7-020)

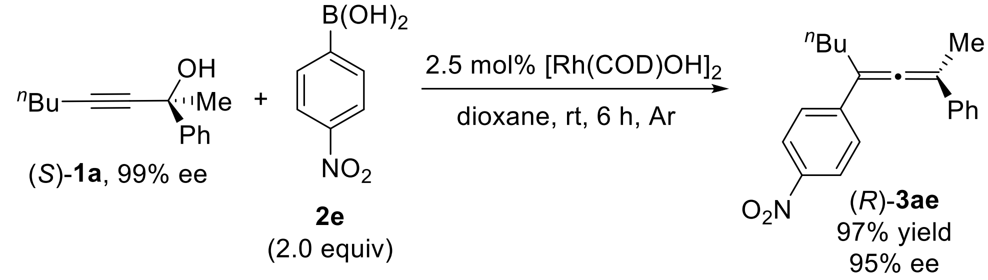

Following Typical Procedure II, the reaction of (*S*)-**1a** (40.3 mg, 0.2 mmol, 99% ee), 4-nitrophenylboronic acid **2e** (66.9 mg, 0.4 mmol), $[Rh(COD)OH]_2$ (2.5 mg, 0.005 mmol) and dioxane (1 mL) afforded (*R*)-**3ae** (59.7 mg, 97%) [eluent: petroleum ether / ethyl acetate = 50/1 (~200 mL)]: 95% ee (HPLC conditions: OD-H column, hexane/*i*-PrOH = 99.5/0.5, 1.0 mL/min, λ = 214 nm, t_R (minor) = 5.4 min, t_R (major) = 6.7 min); oil; **^{1}H NMR** (400 MHz, $CDCl_3$): δ = 8.14 (d, *J* = 8.4 Hz, 2 H, Ar-H), 7.53 (d, *J* = 8.8 Hz, 2 H, Ar-H), 7.42 (d, *J* = 7.6 Hz, 2 H, Ar-H), 7.34 (t, *J* = 7.6 Hz, 2 H, Ar-H), 7.24 (t, *J* = 7.2 Hz, 1 H, Ar-H), 2.65-2.49 (m, 2 H, CH_2), 2.24 (s, 3 H, CH_3), 1.64-1.50 (m, 2 H, CH_2), 1.50-1.35 (m, 2 H, CH_2), 0.92 (t, *J* = 7.2 Hz, 3 H, CH_3); **^{13}C NMR** (100 MHz, $CDCl_3$): δ = 207.4, 146.3, 144.3, 136.0, 128.6, 127.2, 126.5, 125.7, 123.7, 107.0, 104.9, 29.9, 29.8, 22.5, 16.6, 13.9; **IR** (neat): *v* = 2955, 2928, 2859, 1929, 1591, 1513, 1337, 1108, 1026 cm^{-1}; **MS** (70 eV, EI) *m/z* (%):308 (M^++1, 1.79), 307 (M^+, 8.13), 250 (100); **HRMS** calcd *m/z* for $C_{20}H_{21}NO_2$ [M^+]: 307.1567, found: 307.1571.

(6) Preparation of (*R*)-2-phenyl-4-(4-trifluoromethylphenyl)-2,3-octadiene ((*R*)-3af) (zwf-7-019)

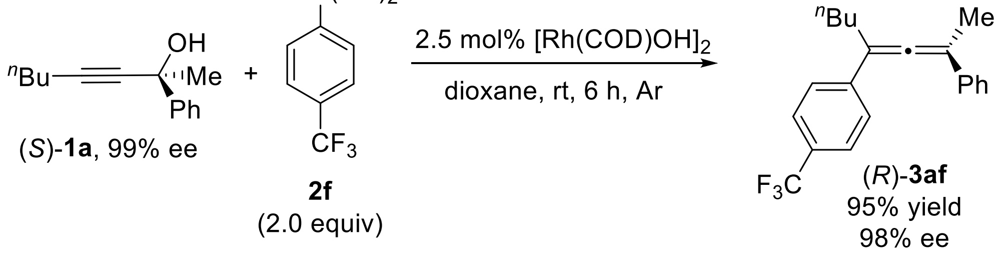

Following Typical Procedure II, the reaction of (*S*)-**1a** (40.1 mg, 0.2 mmol, 99% ee), 4-

trifluoromethylphenylboronic acid **2f** (77.5 mg, 0.4 mmol), $[Rh(COD)OH]_2$ (2.4 mg, 0.005 mmol) and dioxane (1 mL) afforded (*R*)-**3af** (62.0 mg, 95%) [eluent: petroleum ether / ethyl acetate = 50/1 (~200 mL)]: 98% ee (HPLC conditions: AD-H column, hexane, 0.5 mL/min, λ = 214 nm, t_R (major) = 3.6 min, t_R (minor) = 3.9 min); oil; **^{1}H NMR** (400 MHz, $CDCl_3$): δ = 7.58-7.46 (m, 4 H, Ar-H), 7.43 (d, *J* = 8.0 Hz, 2 H, Ar-H), 7.33 (t, *J* = 7.8 Hz, 2 H, Ar-H), 7.22 (t, *J* = 7.4 Hz, 1 H, Ar-H), 2.61-2.47 (m, 2 H, CH_2), 2.20 (s, 3 H, CH_3), 1.62-1.52 (m, 2 H, CH_2), 1.49-1.35 (m, 2 H, CH_2), 0.92 (t, *J* = 7.2 Hz, 3 H, CH_3); **^{13}C NMR** (100 MHz, $CDCl_3$): δ = 206.3, 141.0, 136.6, 128.6 (q, *J* = 32.1 Hz), 128.5, 127.0, 126.2, 125.7, 125.3 (q, *J* = 3.7 Hz), 124.4 (q, *J* = 270.1 Hz), 107.1, 104.4, 30.0, 29.9, 22.6, 16.7, 13.9; **^{19}F NMR** (376 MHz, $CDCl_3$): δ = -62.9; **IR** (neat): *v* = 2958, 2929, 2861, 1933, 1615, 1493, 1322, 1163, 1113, 1068 cm^{-1}; **MS** (70 eV, EI) *m/z* (%): 331 (M^++1, 1.79), 330 (M^+, 8.26), 273 (100); **HRMS** calcd *m/z* for $C_{21}H_{21}F_3$ [M^+]: 330.1590, found: 330.1592.

(7) Preparation of (*R*)-2-phenyl-4-(4-fluorophenyl)-2,3-octadiene ((*R*)-3ag) (zwf-6-172 and zwf-7-029)

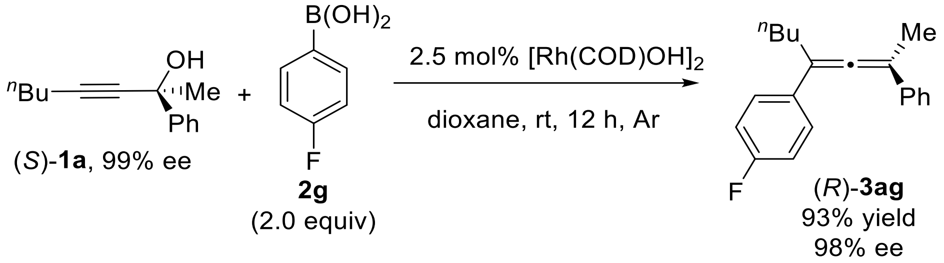

Following Typical Procedure II, the reaction of (*S*)-**1a** (39.7 mg, 0.2 mmol, 99% ee), 4-fluorophenylboronic acid **2g** (56.2 mg, 0.4 mmol), $[Rh(COD)OH]_2$ (2.2 mg, 0.005 mmol) and dioxane (1 mL) afforded (*R*)-**3ag** (51.1 mg, 93%) [eluent: petroleum ether / ethyl acetate = 60/1 (~120 mL)]: 98% ee (HPLC conditions: OJ-3 (3.0 mm I.D. *150mm) column, CO_2/MeOH = 95/5, 1.0 mL/min, λ = 214 nm, t_R (major) = 1.8 min, t_R (minor) = 2.4 min); oil; **^{1}H NMR** (400 MHz, $CDCl_3$): δ = 7.43 (d, *J* = 8.0 Hz, 2 H, Ar-H), 7.39-7.26 (m, 4 H, Ar-H), 7.20 (t, *J* = 7.2 Hz, 1 H, Ar-H), 6.98 (t, *J* = 8.8 Hz, 2 H, Ar-H), 2.59-2.43 (m, 2 H, CH_2), 2.20 (s, 3 H, CH_3), 1.61-1.50 (m, 2 H, CH_2), 1.47-1.34 (m, 2 H, CH_2), 0.91 (t, *J* = 7.4 Hz, 3 H, CH_3); **^{13}C NMR** (100 MHz, $CDCl_3$): δ =

205.2 (d, J = 1.5 Hz), 161.8 (d, J = 244.1 Hz), 137.1, 133.0 (d, J = 3.2 Hz), 128.4, 127.5 (d, J = 7.1 Hz), 126.7, 125.6, 115.2 (d, J = 21.4 Hz), 107.0, 103.8, 30.2, 30.0, 22.6, 16.8, 14.0; **^{19}F NMR** (376 MHz, $CDCl_3$): δ = -116.7; **IR** (neat): v = 2956, 2926, 2860, 1934, 1600, 1505, 1462, 1227, 1158, 1027 cm^{-1}; **MS** (70 eV, EI) m/z (%): 280 (M^+, 8.18), 223 (100); **HRMS** calcd m/z for $C_{20}H_{21}F$ [M^+]: 280.1622, found: 280.1623.

(8) Preparation of (*R*)-2-phenyl-4-(4-chlorophenyl)-2,3-octadiene ((*R*)-3ah) (zwf-6-182 and zwf-7-030)

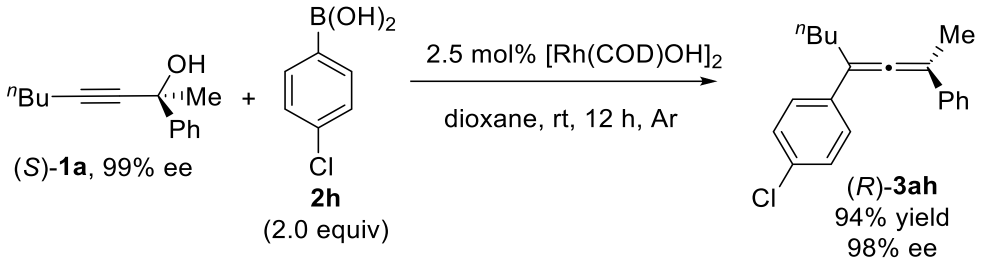

Following Typical Procedure II, the reaction of (*S*)-**1a** (40.2 mg, 0.2 mmol, 99% ee), 4-chlorophenylboronic acid **2h** (62.1 mg, 0.4 mmol), $[Rh(COD)OH]_2$ (2.4 mg, 0.005 mmol) and dioxane (1 mL) afforded (*R*)-**3ah** (55.4 mg, 94%) [eluent: petroleum ether / ethyl acetate = 60/1 (~120 mL)]: 98% ee (HPLC conditions: OJ-3 (3.0 mm I.D. *150mm) column, CO_2/MeOH = 95/5, 1.0 mL/min, λ = 214 nm, t_R (major) = 2.9 min, t_R (minor) = 3.9 min); oil; **^{1}H NMR** (400 MHz, $CDCl_3$): δ = 7.42 (d, J = 7.6 Hz, 2 H, Ar-H), 7.37-7.28 (m, 4 H, Ar-H), 7.28-7.17 (m, 3 H, Ar-H), 2.58-2.40 (m, 2 H, CH_2), 2.20 (s, 3 H, CH_3), 1.60-1.50 (m, 2 H, CH_2), 1.47-1.33 (m, 2 H, CH_2), 0.90 (t, J = 7.4 Hz, 3 H, CH_3); **^{13}C NMR** (100 MHz, $CDCl_3$): δ = 205.5, 136.9, 135.6, 132.3, 128.5, 128.4, 127.3, 126.8, 125.6, 107.0, 104.0, 30.0, 29.9, 22.6, 16.8, 13.9; **IR** (neat): v = 2956, 2927, 2859, 1934, 1597, 1490, 1092, 1011 cm^{-1}; **MS** (70 eV, EI) m/z (%): 298 (M^+(^{37}Cl), 2.99), 296 (M^+(^{35}Cl), 8.66), 239 (100); **HRMS** calcd m/z for $C_{20}H_{21}{}^{35}Cl$ [M^+]: 296.1326, found: 296.1334.

(9) Preparation of (*R*)-2-phenyl-4-(4-bromophenyl)-2,3-octadiene ((*R*)-3ai) (zwf-5-052 and zwf-7-031)

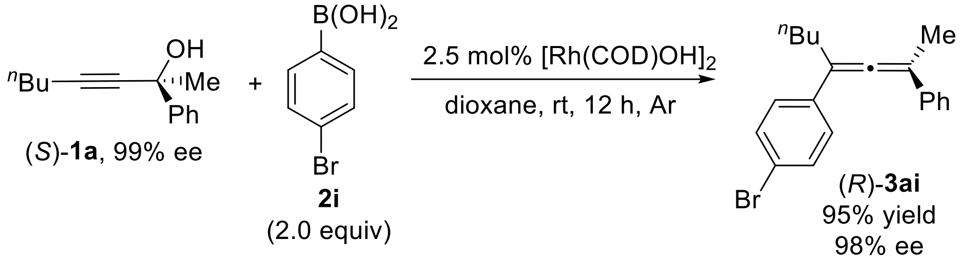

Following Typical Procedure II, (S)-**1a** (40.4 mg, 0.2 mmol, 99% ee), 4-bromophenylboronic acid **2i** (80.5 mg, 0.4 mmol), $[Rh(COD)OH]_2$ (2.5 mg, 0.005 mmol) and dioxane (1 mL) afforded (S)-**3ai** (64.7 mg, 95%) [eluent: petroleum ether / ethyl acetate = 60/1 (~120 mL)]: 98% ee (HPLC conditions: OD-H column, hexane, 0.5 mL/min, λ = 214 nm, t_R (minor) = 13.3 min, t_R (major) = 15.1 min); oil; **^{1}H NMR** (400 MHz, $CDCl_3$): δ = 7.47-7.37 (m, 4 H, Ar-H), 7.37-7.24 (m, 4 H, Ar-H), 7.24-7.16 (m, 1 H, Ar-H), 2.58-2.42 (m, 2 H, CH_2), 2.20 (s, 3 H, CH_3), 1.59-1.49 (m, 2 H, CH_2), 1.47-1.33 (m, 2 H, CH_2), 0.90 (t, J = 7.2 Hz, 3 H, CH_3); **^{13}C NMR** (100 MHz, $CDCl_3$): δ = 205.5, 136.8, 136.0, 131.4, 128.4, 127.6, 126.8, 125.6, 120.4, 107.1, 104.1, 30.0, 29.9, 22.6, 16.7, 14.0; **IR** (neat): v = 2958, 2924, 2856, 1930, 1487, 1071, 1007 cm^{-1}; **MS** (70 eV, EI) m/z (%): 342 (M^+(^{81}Br), 5.00), 340 (M^+(^{79}Br), 5.06), 204 (100); Anal. Calcd. for $C_{20}H_{21}Br$: C 70.39, H 6.20; found C 70.38, H 6.20.

(10) Preparation of (*R*)-2-phenyl-4-(4-methoxyphenyl)-2,3-octadiene ((*R*)-3aj) (zwf-6-186 and zwf-7-032)

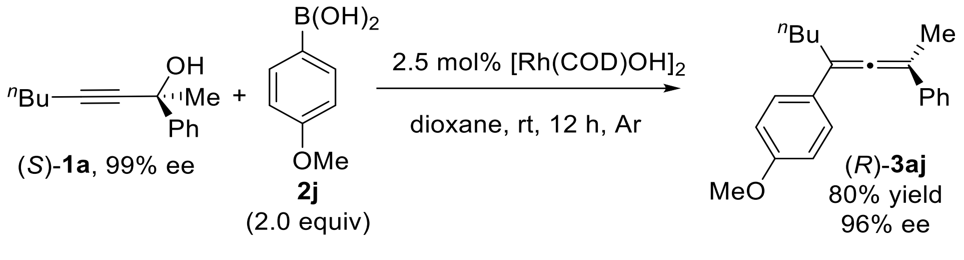

Following Typical Procedure II, the reaction of (S)-**1a** (39.2 mg, 0.2 mmol, 99% ee), 4-methoxyphenylboronic acid **2j** (60.6 mg, 0.4 mmol), $[Rh(COD)OH]_2$ (2.4 mg, 0.005 mmol) and dioxane (1 mL) afforded (R)-**3aj** (45.3 mg, 80%) [eluent: petroleum ether / ethyl acetate = 60/1 (~240 mL)]: 96% ee (HPLC conditions: OD-H column, hexane/i-PrOH = 99.5/0.5, 1.0 mL/min, λ = 214 nm, t_R (minor) = 4.8 min, t_R (major) = 5.2 min); oil; **^{1}H NMR** (400 MHz, $CDCl_3$): δ = 7.44 (d, J = 7.6 Hz, 2 H, Ar-H), 7.38-7.27 (m, 4

H, Ar-H), 7.23-7.14 (m, 1 H, Ar-H), 6.90-6.78 (m, 2 H, Ar-H), 3.79 (s, 3 H, OCH_3), 2.58-2.44 (m, 2 H, CH_2), 2.19 (s, 3 H, CH_3), 1.60-1.51 (m, 2 H, CH_2), 1.47-1.33 (m, 2 H, CH_2), 0.90 (t, J = 7.4 Hz, 3 H, CH_3); **^{13}C NMR** (100 MHz, $CDCl_3$): δ = 205.0, 158.5, 137.5, 129.3, 128.3, 127.1, 126.5, 125.6, 113.9, 107.3, 103.4, 55.3, 30.13, 30.10, 22.6, 16.9, 14.0; **IR** (neat): v = 2955, 2929, 2858, 1933, 1605, 1492, 1247, 1176, 1026 cm^{-1}; **MS** (70 eV, EI) *m/z* (%): 293 (M^++1, 4.18), 292 (M^+, 18.21), 235 (100); **HRMS** calcd *m/z* for $C_{21}H_{24}O$ [M^+]: 292.1822, found: 292.1823.

(11) Preparation of (*R*)-2-phenyl-4-(4-*tert*-butylphenyl)-2,3-octadiene ((*R*)-3ak) (zwf-6-183 and zwf-7-037)

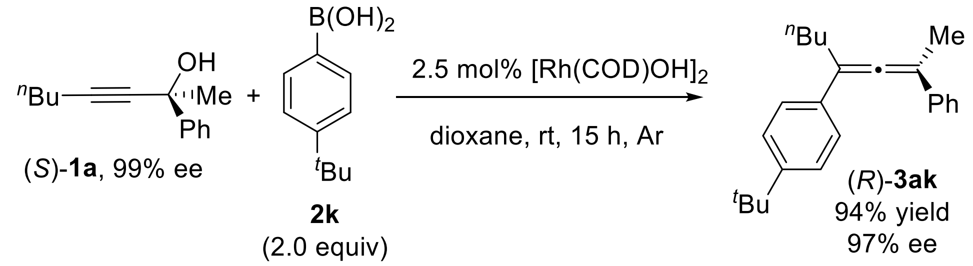

Following Typical Procedure II, the reaction of (*S*)-**1a** (40.2 mg, 0.2 mmol, 99% ee), 4-*tert*-butylphenylboronic acid **2k** (70.9 mg, 0.4 mmol), $[Rh(COD)OH]_2$ (2.1 mg, 0.005 mmol) and dioxane (1 mL) afforded (*R*)-**3ak** (59.3 mg, 94%) [eluent: petroleum ether / ethyl acetate = 60/1 (~120 mL)]: 97% ee (HPLC conditions: OJ-3 (3.0 mm I.D. *150mm) column, CO_2/MeOH = 98/2, 1.0 mL/min, λ = 254 nm, t_R (major) = 4.0 min, t_R (minor) = 5.7 min); oil; **^{1}H NMR** (400 MHz, $CDCl_3$): δ = 7.44 (d, J = 8.0 Hz, 2 H, Ar-H), 7.40-7.25 (m, 6 H, Ar-H), 7.22-7.15 (m, 1 H, Ar-H), 2.61-2.43 (m, 2 H, CH_2), 2.19 (s, 3 H, CH_3), 1.61-1.51 (m, 2 H, CH_2), 1.47-1.36 (m, 2 H, CH_2), 1.30 (s, 9 H, 3 x CH_3), 0.90 (t, J = 7.2 Hz, 3 H, CH_3); **^{13}C NMR** (100 MHz, $CDCl_3$): δ = 205.5, 149.7, 137.4, 134.0, 128.3, 126.5, 125.7, 125.6, 125.3, 107.5, 103.5, 34.4, 31.3, 30.2, 30.0, 22.7, 16.9, 14.0; **IR** (neat): v = 2957, 2928, 2868, 1935, 1492, 1462, 1363, 1268, 1026 cm^{-1}; **MS** (70 eV, EI) *m/z* (%): 318 (M^+, 1.51), 261 (100); **HRMS** calcd *m/z* for $C_{24}H_{30}$ [M^+]: 318.2342, found: 318.2345.

(12) Preparation of (*R*)-2-phenyl-4-(4-phenylphenyl)-2,3-octadiene ((*R*)-3al) (zwf-

6-184 and zwf-7-033)

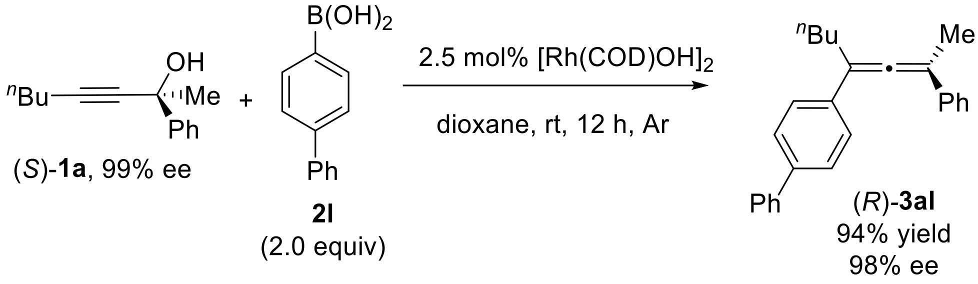

Following Typical Procedure II, the reaction of (*S*)-**1a** (40.3 mg, 0.2 mmol, 99% ee), 4-biphenylboronic acid **2l** (79.0 mg, 0.4 mmol), [Rh(COD)OH]$_2$ (2.2 mg, 0.005 mmol) and dioxane (1 mL) afforded (*R*)-**3al** (63.2 mg, 94%) [eluent: petroleum ether = 360 mL]: 98% ee (HPLC conditions: OD-H column, hexane/*i*-PrOH = 99.5/0.5, 1.0 mL/min, λ = 214 nm, t_R (minor) = 5.3 min, t_R (major) = 5.9 min); oil; **^{1}H NMR** (400 MHz, $CDCl_3$): δ = 7.67-7.37 (m, 10 H, Ar-H), 7.36-7.26 (m, 3 H, Ar-H), 7.21 (t, *J* = 7.4 Hz, 1 H, Ar-H), 2.66-2.49 (m, 2 H, CH_2), 2.23 (s, 3 H, CH_3), 1.68-1.52 (m, 2 H, CH_2), 1.50-1.34 (m, 2 H, CH_2), 0.92 (t, *J* = 7.4 Hz, 3 H, CH_3); **^{13}C NMR** (100 MHz, $CDCl_3$): δ = 205.8, 140.8, 139.5, 137.2, 136.0, 128.7, 128.4, 127.14, 127.13, 126.9, 126.7, 126.4, 125.7, 107.5, 103.7, 30.1, 30.0, 22.6, 16.9, 14.0; **IR** (neat): *ν* = 3057, 2959, 2925, 2867, 1932, 1597, 1464, 1065, 1029 cm^{-1}; **MS** (70 eV, EI) *m/z* (%): 339 (M^++1, 6.83), 338 (M^+, 21.09), 281 (100); **HRMS** calcd *m/z* for $C_{26}H_{26}$ [M^+]: 338.2029, found: 338.2031.

(13) Preparation of (*R*)-2-phenyl-4-(2-methylphenyl)-2,3-octadiene ((*R*)-3am) (zwf-5-057 and zwf-7-035)

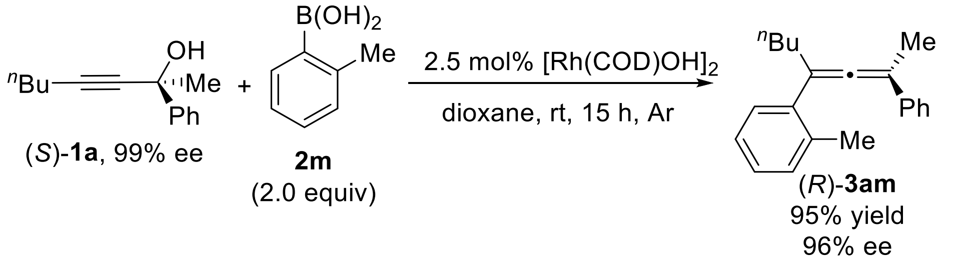

Following Typical Procedure II, the reaction of (*S*)-**1a** (40.0 mg, 0.2 mmol, 99% ee), 2-methylphenylboronic acid **2m** (54.2 mg, 0.4 mmol), [Rh(COD)OH]$_2$ (2.2 mg, 0.005 mmol) and dioxane (1 mL) afforded (*R*)-**3am** (52.2 mg, 95%) [eluent: petroleum ether / ethyl acetate = 50/1 (~100 mL)]: 96% ee (HPLC conditions: OJ-3 (3.0 mm I.D.

*150mm) column, CO_2/MeOH = 99/1, 1.0 mL/min, λ = 230 nm, t_R (major) = 4.0 min, t_R (minor) = 4.3 min); oil; **^{1}H NMR** (400 MHz, $CDCl_3$): δ = 7.42 (d, J = 7.2 Hz, 2 H, Ar-H), 7.36-7.23 (m, 3 H, Ar-H), 7.22-7.04 (m, 4 H, Ar-H), 2.57-2.26 (m, 5 H, CH_2 and CH_3), 2.15 (s, 3 H, CH_3), 1.58-1.31 (m, 4 H, 2 x CH_2), 0.89 (t, J = 7.2 Hz, 3 H, CH_3); **^{13}C NMR** (100 MHz, $CDCl_3$): δ = 203.3, 138.1, 137.8, 135.8, 130.4, 128.2, 128.0, 126.7, 126.3, 125.74, 125.71, 106.7, 100.7, 34.0, 30.1, 22.5, 20.7, 17.1, 14.0; **IR** (neat): v = 2956, 2927, 2858, 1943, 1490, 1459, 1027 cm^{-1}; **MS** (70 eV, EI) m/z (%): 276 (M^+, 3.62), 219 (100); **HRMS** calcd m/z for $C_{21}H_{24}$ [M^+]: 276.1873, found: 276.1870.

(14) Preparation of (*R*)-2-phenyl-4-(3-methylphenyl)-2,3-octadiene ((*R*)-3an) (zwf-6-169 and zwf-7-027)

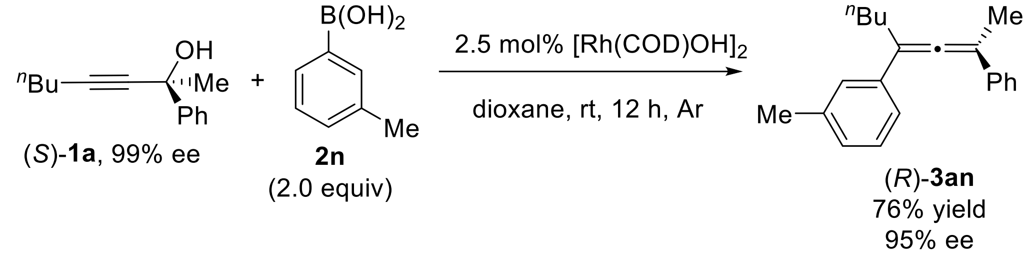

(*S*)-**1a**, 99% ee

2n
(2.0 equiv)

(*R*)-**3an**
76% yield
95% ee

Following Typical Procedure II, the reaction of (*S*)-**1a** (40.0 mg, 0.2 mmol, 99% ee), 3-methylphenylboronic acid **2n** (54.2 mg, 0.4 mmol), $[Rh(COD)OH]_2$ (2.2 mg, 0.005 mmol) and dioxane (1 mL) afforded (*R*)-**3an** (41.6 mg, 76%) [eluent: petroleum ether / ethyl acetate = 60/1 (~122 mL)]: 95% ee (HPLC conditions: OJ-H column, hexane/*i*-PrOH = 99.5/0.5, 0.7 mL/min, λ = 214 nm, t_R (major) = 6.1 min, t_R (minor) = 7.4 min); oil; **^{1}H NMR** (400 MHz, $CDCl_3$): δ = 7.44 (d, J = 7.6 Hz, 2 H, Ar-H), 7.31 (t, J = 7.6 Hz, 2 H, Ar-H), 7.26-7.12 (m, 4 H, Ar-H), 7.01 (d, J = 6.8 Hz, 1 H, Ar-H), 2.62-2.45 (m, 2 H, CH_2), 2.32 (s, 3 H, CH_3), 2.20 (s, 3 H, CH_3), 1.61-1.51 (m, 2 H, CH_2), 1.48-1.33 (m, 2 H, CH_2), 0.90 (t, J = 7.4 Hz, 3 H, CH_3); **^{13}C NMR** (100 MHz, $CDCl_3$): δ = 205.5, 137.9, 137.3, 137.0, 128.4, 128.3, 127.5, 126.7, 126.6, 125.6, 123.2, 107.8, 103.4, 30.1, 30.0, 22.6, 21.5, 16.8, 14.0; **IR** (neat): v = 2925, 2859, 1933, 1600, 1443, 1026 cm^{-1}; **MS** (70 eV, EI) m/z (%): 277 (M^++1, 2.36), 276 (M^+, 9.99), 219 (100); **HRMS** calcd m/z for $C_{21}H_{24}$ [M^+]: 276.1873, found: 276.1875.

(15) Preparation of (*R*)-2-phenyl-4-(4-methylphenyl)-2,3-octadiene ((*R*)-3ao) (zwf-6-170 and zwf-7-028)

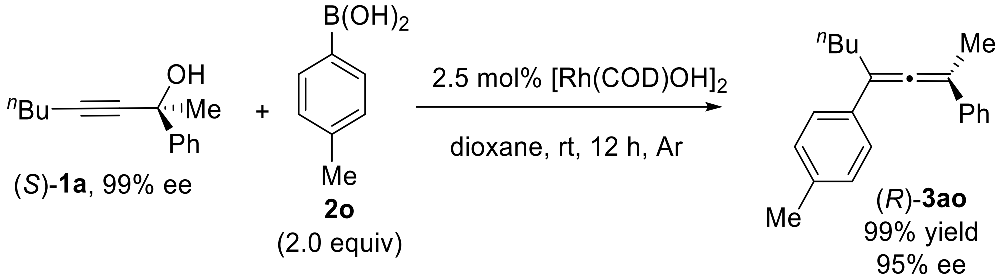

Following Typical Procedure II, the reaction of (*S*)-**1a** (40.2 mg, 0.2 mmol, 99% ee), 4-methylphenylboronic acid **2o** (54.6 mg, 0.4 mmol), $[Rh(COD)OH]_2$ (2.4 mg, 0.005 mmol) and dioxane (1 mL) afforded (*R*)-**3ao** (54.2 mg, 99%) [eluent: petroleum ether / ethyl acetate = 60/1 (~120 mL)]: 95% ee (HPLC conditions: OJ-3 (3.0 mm I.D. *150mm) column, CO_2/MeOH = 95/5, 1.0 mL/min, λ = 214 nm, t_R (major) = 2.0 min, t_R (minor) = 3.0 min); oil; **^{1}H NMR** (400 MHz, $CDCl_3$): δ = 7.43 (d, *J* = 8.0 Hz, 2 H, Ar-H), 7.30 (t, *J* = 8.0 Hz, 4 H, Ar-H), 7.22-7.16 (m, 1 H, Ar-H), 7.11 (d, *J* = 8.0 Hz, 2 H, Ar-H), 2.59-2.46 (m, 2 H, CH_2), 2.32 (s, 3 H, CH_3), 2.19 (s, 3 H, CH_3), 1.62-1.50 (m, 2 H, CH_2), 1.47-1.32 (m, 2 H, CH_2), 0.90 (t, *J* = 7.2 Hz, 3 H, CH_3); **^{13}C NMR** (100 MHz, $CDCl_3$): δ = 205.3, 137.4, 136.4, 134.0, 129.1, 128.4, 126.5, 126.0, 125.6, 107.6, 103.4, 30.1, 30.0, 22.6, 21.0, 16.9, 14.0; **IR** (neat): *v* = 2958, 2929, 2859, 1929, 1595, 1492, 1450, 1026 cm^{-1}; **MS** (70 eV, EI) *m/z* (%): 277 (M^++1, 1.91), 276 (M^+, 8.85), 219 (100); **HRMS** calcd *m/z* for $C_{21}H_{24}$ [M^+]: 276.1873, found: 276.1874.

(16) Preparation of (*R*)-2-phenyl-4-(1-naphthyl)-2,3-octadiene ((*R*)-3ap) (zwf-7-100)

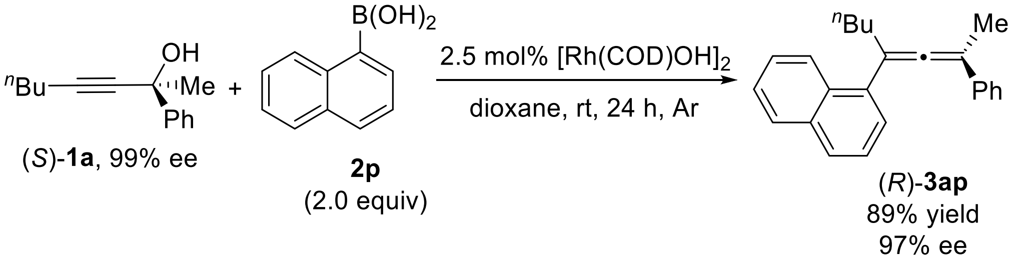

Following Typical Procedure II, the reaction of (*S*)-**1a** (39.9 mg, 0.2 mmol, 99% ee), 1-naphthaleneboronic acid **2p** (68.0 mg, 0.4 mmol), $[Rh(COD)OH]_2$ (2.4 mg, 0.005 mmol)

and dioxane (1 mL) afforded (*R*)-**3ap** (55.2 mg, 89%) [eluent: petroleum ether = 240 mL]: 97% ee (HPLC conditions: OJ-3 (3.0 mm I.D. *150mm) column, CO_2/MeOH = 99/1, 1.0 mL/min, λ = 210 nm, t_R (major) = 10.1 min, t_R (minor) = 11.2 min); oil; **^{1}H NMR** δ = 8.28-8.16 (m, 1 H, Ar-H), 7.87-7.76 (m, 1 H, Ar-H), 7.73 (d, *J* = 8.0 Hz, 1 H, Ar-H), 7.53-7.35 (m, 6 H, Ar-H), 7.31 (t, *J* = 7.6 Hz, 2 H, Ar-H), 7.17 (t, *J* = 7.2 Hz, 1 H, Ar-H), 2.68-2.46 (m, 2 H, CH_2), 2.20 (s, 3 H, CH_3), 1.63-1.50 (m, 2 H, CH_2), 1.47-1.33 (m, 2 H, CH_2), 0.89 (t, *J* = 7.4 Hz, 3 H, CH_3); **^{13}C NMR** (100 MHz, $CDCl_3$): δ = 203.8, 137.8, 136.9, 134.0, 131.4, 128.4, 128.3, 127.3, 126.4, 125.8, 125.7, 125.6, 125.5, 125.4, 106.1, 100.9, 34.8, 30.3, 22.5, 17.2, 14.0; **IR** (neat): *v* = 3057, 2955, 2926, 1944, 1593, 1493, 1443, 1065, 1026 cm^{-1}; **MS** (70 eV, EI) *m/z* (%): 313 (M^++1, 8.35), 312 (M^+, 33.40), 255 (100); **HRMS** calcd *m/z* for $C_{24}H_{24}$ [M^+]: 312.1873, found: 312.1876.

(17) Preparation of (*R*)-2-phenyl-4-(4-formylphenyl)-2,3-nonadiene ((*R*)-3cb) (zwf-7-090)

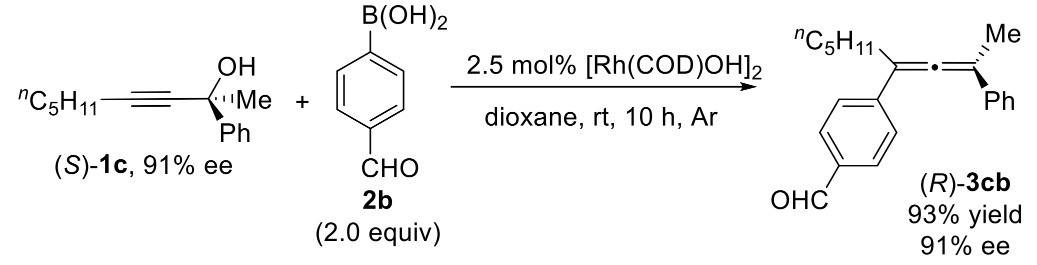

Following Typical Procedure II, the reaction of (*S*)-**1c** (43.0 mg, 0.2 mmol, 91% ee), 4-formylphenylboronic acid **2b** (59.7 mg, 0.4 mmol), $[Rh(COD)OH]_2$ (2.5 mg, 0.005 mmol) and dioxane (1 mL) afforded (*R*)-**3cb** (56.4 mg, 93%) [eluent: petroleum ether / ethyl acetate = 50/1 (~200 mL)]: 91% ee (HPLC conditions: OD-H column, hexane/*i*-PrOH = 99.5/0.5, 1.0 mL/min, λ = 214 nm, t_R (minor) = 9.7 min, t_R (major) = 16.1 min); oil; **^{1}H NMR** (400 MHz, $CDCl_3$): δ = 9.96 (s, 1 H, CHO), 7.81 (d, *J* = 8.4 Hz, 2 H, Ar-H), 7.56 (d, *J* = 8.4 Hz, 2 H, Ar-H), 7.43 (d, *J* = 8.0 Hz, 2 H, Ar-H), 7.33 (t, *J* = 7.8 Hz, 2 H, Ar-H), 7.23 (t, *J* = 7.2 Hz, 1 H, Ar-H), 2.65-2.48 (m, 2 H, CH_2), 2.23 (s, 3 H, CH_3), 1.65-1.51 (m, 2 H, CH_2), 1.43-1.20 (m, 4 H, 2 x CH_2), 0.86 (t, *J* = 7.0 Hz, 3 H, CH_3); **^{13}C NMR** (100 MHz, $CDCl_3$): δ = 207.2, 191.7, 143.7, 136.3, 134.7, 129.9, 128.5, 127.0, 126.4, 125.7, 107.5, 104.5, 31.6, 30.0, 27.5, 22.5, 16.6, 14.0; **IR** (neat): *v* = 2953, 2928,

2857, 1929, 1697, 1599, 1211, 1026 cm^{-1}; **MS** (70 eV, EI) *m/z* (%): 305 (M^++1, 3.90), 304 (M^+, 13.60), 248, 233 (100); **HRMS** calcd for m/z $C_{22}H_{24}O$ [M^+]: 304.1822, found: 304.1827.

(18) Preparation of (*R*)-2-phenyl-4-(4-chlorophenyl)-2,3-decadiene ((*R*)-3dh) (zwf-7-091)

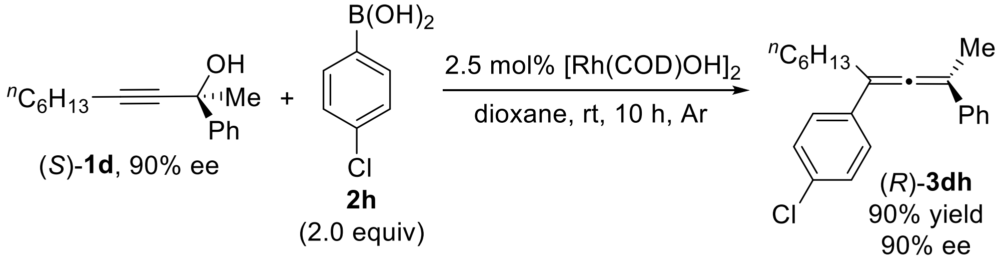

Following Typical Procedure II, the reaction of (*S*)-**1d** (46.3 mg, 0.2 mmol, 90% ee), 4-chlorophenylboronic acid **2h** (62.9 mg, 0.4 mmol), $[Rh(COD)OH]_2$ (2.5 mg, 0.005 mmol) and dioxane (1 mL) afforded (*R*)-**3dh** (58.6 mg, 90%) [eluent: petroleum ether / ethyl acetate = 50/1 (~200 mL)]: 90% ee (HPLC conditions: OD-H column, hexane, 0.5 mL/min, λ = 214 nm, t_R (minor) = 10.4 min, t_R (major) = 12.0 min); oil; **^{1}H NMR** (400 MHz, $CDCl_3$): δ = 7.42 (d, *J* = 8.0 Hz, 2 H, Ar-H), 7.37-7.28 (m, 4 H, Ar-H), 7.28-7.17 (m, 3 H, Ar-H), 2.57-2.40 (m, 2 H, CH_2), 2.20 (s, 3 H, CH_3), 1.61-1.51 (m, 2 H, CH_2), 1.44-1.33 (m, 2 H, CH_2), 1.32-1.18 (m, 4 H, CH_2), 0.85 (t, *J* = 7.0 Hz, 3 H, CH_3); **^{13}C NMR** (100 MHz, $CDCl_3$): δ = 205.5, 136.9, 135.6, 132.3, 128.5, 128.4, 127.3, 126.8, 125.6, 107.0, 104.0, 31.7, 30.2, 29.2, 27.8, 22.7, 16.8, 14.0; **IR** (neat): *ν* = 2953, 2926, 2857, 1933, 1597, 1489, 1092, 1011 cm^{-1}; **MS** (70 eV, EI) *m/z* (%): 326 (M^+(^{37}Cl), 2.03), 324 (M^+(^{35}Cl), 6.47), 239 (100); **HRMS** calcd *m/z* for $C_{22}H_{25}{}^{35}Cl$ [M^+]: 324.1639, found: 324.1642.

(19) Preparation of (*R*)-2,4-diphenyl-8-chloro-2,3-octadiene ((*R*)-3ea) (zwf-7-051)

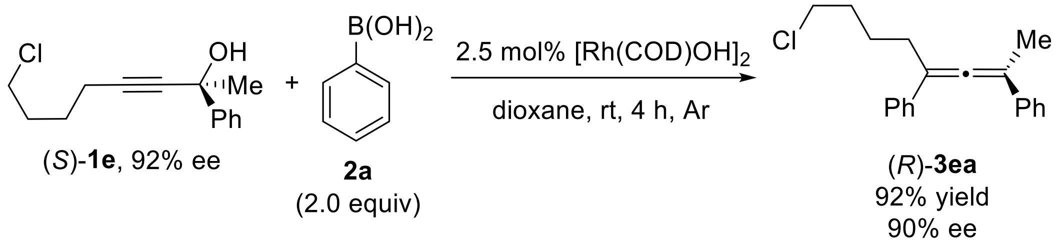

Following Typical Procedure II, the reaction of (*S*)-**1e** (47.2 mg, 0.2 mmol, 92% ee), phenylboronic acid **2a** (48.5 mg, 0.4 mmol), $[Rh(COD)OH]_2$ (2.4 mg, 0.005 mmol) and dioxane (1 mL) afforded (*R*)-**3ea** (54.2 mg, 92%) [eluent: petroleum ether / ethyl acetate = 60/1 (~120 mL)]: 90% ee (HPLC conditions: AD-H column, hexane/*i*-PrOH = 99.5/0.5, 0.5 mL/min, λ = 214 nm, t_R (major) = 9.2 min, t_R (minor) = 9.9 min); oil; **^{1}H NMR** (400 MHz, $CDCl_3$): δ = 7.49-7.36 (m, 4 H, Ar-H), 7.35-7.25 (m, 4 H, Ar-H), 7.24-7.17 (m, 2 H, Ar-H), 3.51 (t, *J* = 6.8 Hz, 2 H, CH_2), 2.67-2.49 (m, 2 H, CH_2), 2.21 (s, 3 H, CH_3), 1.93-1.81 (m, 2 H, CH_2), 1.78-1.64 (m, 2 H, CH_2); **^{13}C NMR** (100 MHz, $CDCl_3$): δ = 205.4, 137.0, 136.7, 128.5, 128.4, 126.84, 126.80, 126.0, 125.6, 107.2, 104.1, 44.8, 32.3, 29.4, 25.1, 16.9; **IR** (neat): *v* = 2941, 2863, 1933, 1596, 1445, 1065, 1026 cm^{-1}; **MS** (70 eV, EI) *m/z* (%): 298 (M^+(^{37}Cl), 2.32), 296 (M^+(^{35}Cl), 6.91), 205 (100); **HRMS** calcd *m/z* for $C_{20}H_{21}{}^{35}Cl$ [M^+]: 296.1326, found: 296.1325.

(20) Preparation of (*R*)-2-phenyl-4-(4-acetylphenyl)-8-chloro-2,3-octadiene ((*R*)-3ec) (zwf-7-093)

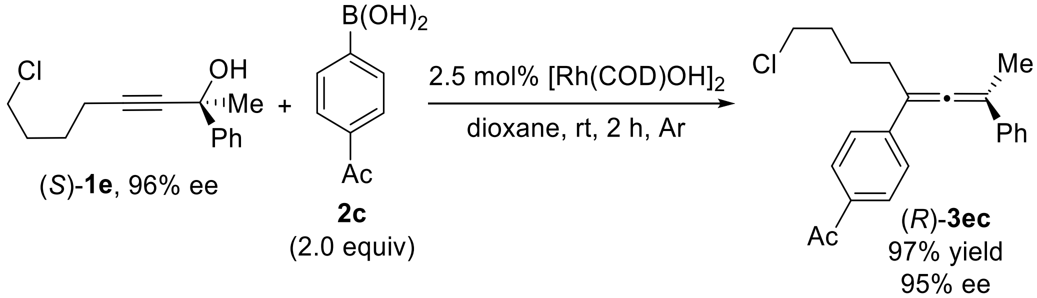

Following Typical Procedure II, the reaction of (*S*)-**1e** (47.0 mg, 0.2 mmol, 96% ee), 4-acetylphenylboronic acid **2c** (64.2 mg, 0.4 mmol), $[Rh(COD)OH]_2$ (2.4 mg, 0.005 mmol) and dioxane (1 mL) afforded (*R*)-**3ec** (65.1 mg, 97%) [eluent: petroleum ether / ethyl acetate = 50/1 (~200 mL), then 20/1 (~210 mL)]: 95% ee (HPLC conditions: OD-

H column, hexane/*i*-PrOH = 98/2, 1.0 mL/min, λ = 214 nm, t_R (minor) = 12.1 min, t_R (major) = 13.3 min); oil; **^{1}H NMR** (400 MHz, $CDCl_3$): δ = 7.90 (d, *J* = 8.4 Hz, 2 H, Ar-H), 7.49 (d, *J* = 8.4 Hz, 2 H, Ar-H), 7.42 (d, *J* = 7.6 Hz, 2 H, Ar-H), 7.33 (t, *J* = 7.6 Hz, 2 H, Ar-H), 7.23 (t, *J* = 7.2 Hz, 1 H, Ar-H), 3.52 (t, *J* = 6.6 Hz, 2 H, CH_2), 2.68-2.49 (m, 5 H, CH_2 and CH_3), 2.24 (s, 3 H, CH_3), 1.93-1.81 (m, 2 H, CH_2), 1.80-1.70 (m, 2 H, CH_2); **^{13}C NMR** (100 MHz, $CDCl_3$): δ = 206.6, 197.5, 141.8, 136.3, 135.4, 128.6, 128.5, 127.1, 126.0, 125.6, 106.8, 104.8, 44.7, 32.2, 29.2, 26.5, 25.0, 16.7; **IR** (neat): *v* = 2941, 1931, 1678, 1599, 1358, 1265, 1026 cm^{-1}; **MS** (70 eV, EI) *m/z* (%): 340 (M^+(^{37}Cl), 9.13), 338 (M^+(^{35}Cl), 25.42), 247 (100); **HRMS** calcd *m/z* for $C_{22}H_{23}{}^{35}ClO$ [M^+]: 338.1432, found: 338.1430.

(21) Preparation of (*R*)-2,6-diphenyl-4-(3-chlorophenyl)-2,3-hexadiene ((*R*)-3fq) (zwf-5-062 and zwf-7-052)

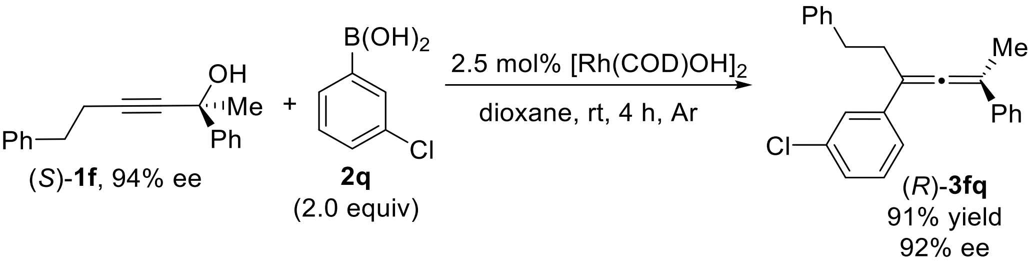

(*S*)-**1f**, 94% ee **2q** (2.0 equiv) (*R*)-**3fq** 91% yield 92% ee

Following Typical Procedure II, (*S*)-**1f** (50.4 mg, 0.2 mmol, 94% ee), 3-chlorophenylboronic acid **2q** (62.3 mg, 0.4 mmol), $[Rh(COD)OH]_2$ (2.3 mg, 0.005 mmol) and dioxane (1 mL) afforded (*R*)-**3fq** (63.5 mg, 91%) [eluent: petroleum ether / ethyl acetate = 60/1 (~120 mL)]: 92% ee (HPLC conditions: OD-H column, hexane/*i*-PrOH = 99.5/0.5, 1.0 mL/min, λ = 214 nm, t_R (major) = 7.0 min, t_R (minor) = 7.4 min); oil; **^{1}H NMR** (400 MHz, $CDCl_3$): δ = 7.55-7.00 (m, 14 H, Ar-H), 2.95-2.73 (m, 4 H, 2 x CH_2), 2.09 (s, 3 H, CH_3); **^{13}C NMR** (100 MHz, $CDCl_3$): δ = 205.8, 141.5, 138.9, 136.5, 134.5, 129.6, 128.5, 128.44, 128.35, 126.9, 126.8, 126.0, 125.9, 125.7, 124.2, 106.3, 104.8, 33.9, 31.8, 16.7; **IR** (neat): *v* = 3061, 3027, 2919, 2857, 1936, 1591, 1493, 1079, 1026 cm^{-1}; **MS** (70 eV, EI) *m/z* (%): 346 (M^+(^{37}Cl), 18.30), 344 (M(^{35}Cl), 51.47), 91 (100); **HRMS** calcd for *m/z* $C_{24}H_{21}{}^{35}Cl$ [M^+]: 344.1326, found: 344.1335.

(22) Preparation of (*R*)-2-(thiophen-3-yl)-4-phenyl-2,3-octadiene ((*R*)-3ga) (zwf-7-054)

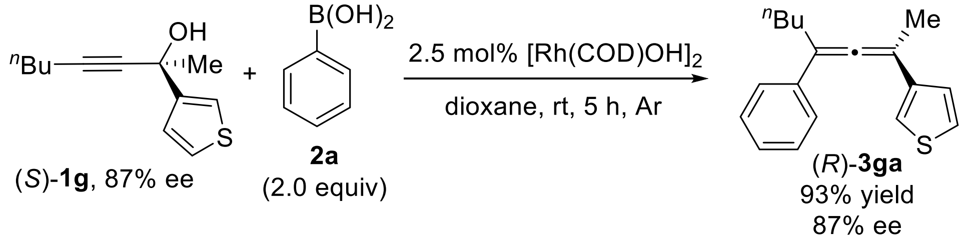

Following Typical Procedure II, the reaction of (*S*)-**1g** (40.8 mg, 0.2 mmol, 87% ee), phenylboronic acid **2a** (49.1 mg, 0.4 mmol), $[Rh(COD)OH]_2$ (2.4 mg, 0.005 mmol) and dioxane (1 mL) afforded (*R*)-**3ga** (48.7 mg, 93%) [eluent: petroleum ether (~260 mL)]: 87% ee (HPLC conditions: AD-H column, hexane/*i*-PrOH = 99.5/0.5, 0.5 mL/min, λ = 214 nm, t_R (major) = 7.7 min, t_R (minor) = 8.2 min); oil; **^{1}H NMR** (400 MHz, $CDCl_3$): δ = 7.41 (d, *J* = 7.6 Hz, 2 H, Ar-H), 7.30 (t, *J* = 7.6 Hz, 2 H, Ar-H), 7.24-7.16 (m, 2 H, Ar-H and one proton of thienyl), 7.14-7.02 (m, 2 H, two proton of thienyl), 2.52 (t, *J* = 7.6 Hz, 2 H, CH_2), 2.17 (s, 3 H, CH_3), 1.60-1.51 (m, 2 H, CH_2), 1.48-1.34 (m, 2 H, CH_2), 0.91 (t, *J* = 7.2 Hz, 3 H, CH_3); **^{13}C NMR** (100 MHz, $CDCl_3$): δ = 205.4, 139.6, 137.2, 128.4, 126.7, 126.2, 125.4, 118.9, 107.2, 100.0, 30.2, 30.1, 22.6, 17.4, 14.0; **IR** (neat): *v* = 2955, 2925, 2858, 1934, 1597, 1445, 1246, 1181, 1026 cm^{-1}; **MS** (70 eV, EI) *m/z* (%): 269 (M^++1, 2.62), 268 (M^+, 13.51), 211 (100); **HRMS** calcd *m/z* for $C_{18}H_{20}S$ [M^+]: 268.1280, found: 268.1283.

(23) Preparation of (*R*)-2-(2-methylphenyl)-4-(4-bromophenyl)-2,3-octadiene ((*R*)-3hi) (zwf-7-016)

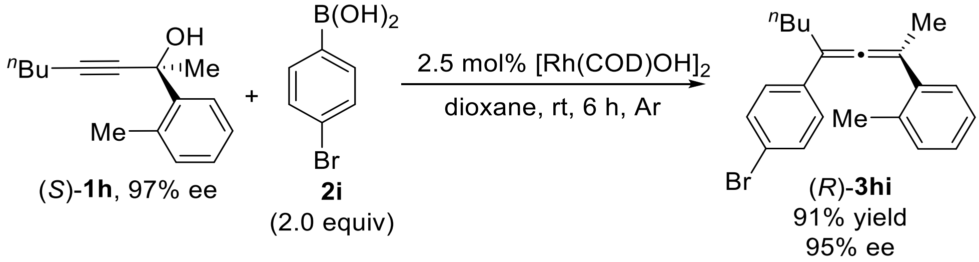

Following Typical Procedure II, the reaction of (*S*)-**1h** (43.0 mg, 0.2 mmol, 97% ee), 4-bromophenylboronic acid **2i** (80.5 mg, 0.4 mmol), $[Rh(COD)OH]_2$ (2.4 mg, 0.005

mmol) and dioxane (1 mL) afforded (*R*)-**3hi** (64.1 mg, 91%) [eluent: petroleum ether / ethyl acetate = 60/1 (~120 mL)]: 95% ee (HPLC conditions: AD-H column, hexane/*i*-PrOH = 99.5/0.5, 0.5 mL/min, λ = 214 nm, t_R (minor) = 6.4 min, t_R (major) = 7.2 min); oil; **^{1}H NMR** (400 MHz, $CDCl_3$): δ = 7.42 (d, *J* = 8.8 Hz, 2 H, Ar-H), 7.32-7.24 (m, 3 H, Ar-H), 7.22-7.08 (m, 3 H, Ar-H), 2.41 (t, *J* = 7.4 Hz, 2 H, CH_2), 2.31 (s, 3 H, CH_3), 2.15 (s, 3 H, CH_3), 1.58-1.50 (m, 2 H, CH_2), 1.45-1.33 (m, 2 H, CH_2), 0.92 (t, *J* = 7.2 Hz, 3 H, CH_3); **^{13}C NMR** (100 MHz, $CDCl_3$): δ = 203.9, 138.1, 136.8, 135.7, 131.3, 130.6, 127.9, 127.6, 126.9, 125.9, 120.2, 104.1, 103.3, 30.1, 30.0, 22.5, 20.9, 20.5, 14.0; **IR** (neat): *v* = 2954, 2925, 2858, 1943, 1486, 1460, 1105, 1043, 1007 cm^{-1}; **MS** (70 eV, EI) *m/z* (%): 356 (M^+(^{81}Br), 2.20), 354 (M^+(^{79}Br), 1.90), 218 (100); **HRMS** calcd m/z for $C_{21}H_{23}{}^{79}Br$ [M^+]: 354.0978, found: 354.0978.

(24) Preparation of (*R*)-2-(3-methoxyphenyl)-4-(4-chlorophenyl)-2,3-octadiene ((*R*)-3ih) (zwf-7-017)

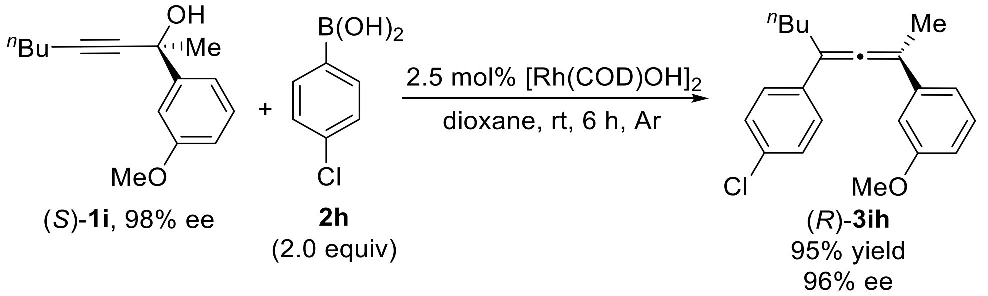

Following Typical Procedure II, the reaction of (*S*)-**1i** (46.1 mg, 0.2 mmol, 98% ee), 4-chlorophenylboronic acid **2h** (62.3 mg, 0.4 mmol), $[Rh(COD)OH]_2$ (2.3 mg, 0.005 mmol) and dioxane (1 mL) afforded (*R*)-**3ih** (61.9 mg, 95%) [eluent: petroleum ether / ethyl acetate = 60/1 (~120 mL)]: 96% ee (HPLC conditions: OD-H column, hexane/*i*-PrOH = 99.5/0.5, 1.0 mL/min, λ = 214 nm, t_R (minor) = 3.8 min, t_R (major) = 4.0 min); oil; **^{1}H NMR** (400 MHz, $CDCl_3$): δ = 7.33 (d, *J* = 8.4 Hz, 2 H, Ar-H), 7.28-7.18 (m, 3 H, Ar-H), 7.02 (d, *J* = 8.0 Hz, 1 H, Ar-H), 6.97 (s, 1 H, Ar-H), 6.77 (dd, J_1 = 8.0 Hz, J_2 = 2.0 Hz, 1 H, Ar-H), 3.78 (s, 3 H, OCH_3), 2.58-2.41 (m, 2 H, CH_2), 2.18 (s, 3 H, CH_3), 1.60-1.50 (m, 2 H, CH_2), 1.47-1.34 (m, 2 H, CH_2), 0.90 (t, *J* = 7.4 Hz, 3 H, CH_3); **^{13}C NMR** (100 MHz, $CDCl_3$): δ = 205.6, 159.8, 138.5, 135.5, 132.3, 129.3, 128.5, 127.3,

118.2, 111.9, 111.7, 107.0, 103.9, 55.2, 30.0, 29.9, 22.6, 16.8, 13.9; **IR** (neat): v = 2955, 2927, 2863, 1934, 1599, 1487, 1322, 1044, 1013 cm^{-1}; **MS** (70 eV, EI) *m/z* (%): 328 (M^+(^{37}Cl), 7.35), 326 (M^+(^{35}Cl), 19.20), 269 (100); **HRMS** calcd m/z for $C_{21}H_{23}{}^{35}ClO[M^+]$: 326.1432, found: 326.1434.

(25) Preparation of (*R*)-2-(4-bromophenyl)-4-(4-formylphenyl)-2,3-octadiene ((*R*)-3jb) (zwf-7-018)

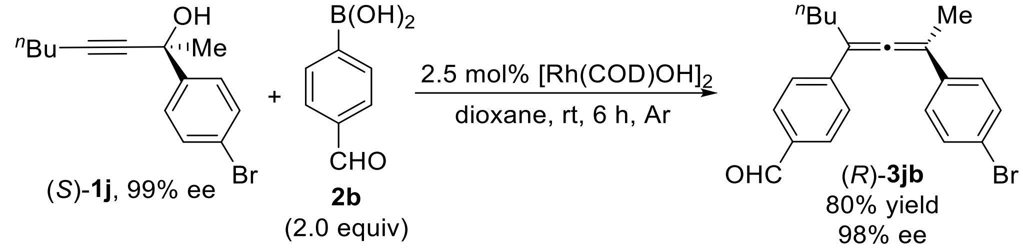

Following Typical Procedure II, the reaction of (*S*)-**1j** (56.2 mg, 0.2 mmol, 99% ee), 4-formylphenylboronic acid **2b** (60.3 mg, 0.4 mmol), $[Rh(COD)OH]_2$ (2.2 mg, 0.005 mmol) and dioxane (1 mL) afforded (*R*)-**3jb** (59.3 mg, 80%) [eluent: petroleum ether / ethyl acetate = 50/1 (~150 mL)]: 98% ee (HPLC conditions: OD-H column, hexane/*i*-PrOH = 99.5/0.5, 1.0 mL/min, λ = 214 nm, t_R (minor) = 5.8 min, t_R (major) = 6.4 min); oil; **^{1}H NMR** (400 MHz, $CDCl_3$): δ = 9.97 (s, 1 H, CHO), 7.81 (d, J = 8.4 Hz, 2 H, Ar-H), 7.54 (d, J = 8.4 Hz, 2 H, Ar-H), 7.44 (d, J = 8.4 Hz, 2 H, Ar-H), 7.28 (d, J = 8.8 Hz, 2 H, Ar-H), 2.57 (t, J = 7.4 Hz, 2 H, CH_2), 2.21 (s, 3 H, CH_3), 1.61-1.50 (m, 2 H, CH_2), 1.48-1.36 (m, 2 H, CH_2), 0.92 (t, J = 7.4 Hz, 3 H, CH_3); **^{13}C NMR** (100 MHz, $CDCl_3$): δ = 207.1, 191.7, 143.3, 135.4, 134.9, 131.6, 130.0, 127.3, 126.5, 120.9, 108.0, 103.8, 30.0, 29.8, 22.5, 16.6, 13.9; **IR** (neat): v = 2955, 2926, 2731, 1929, 1698, 1569, 1485, 1212, 1168, 1076, 1007 cm^{-1}; **MS** (70 eV, EI) *m/z* (%): 370 (M^+(^{81}Br), 4.83), 368 (M^+(^{79}Br), 5.46), 247 (100); **HRMS** calcd m/z for $C_{21}H_{21}{}^{79}BrO$ [M^+]: 368.0770, found: 368.0771.

(26) Preparation of (*R*)-3-(2-methylphenyl)-5-(4-chlorophenyl)-3,4-octadiene ((*R*)-3kh) (zwf-5-055 and zwf-7-053)

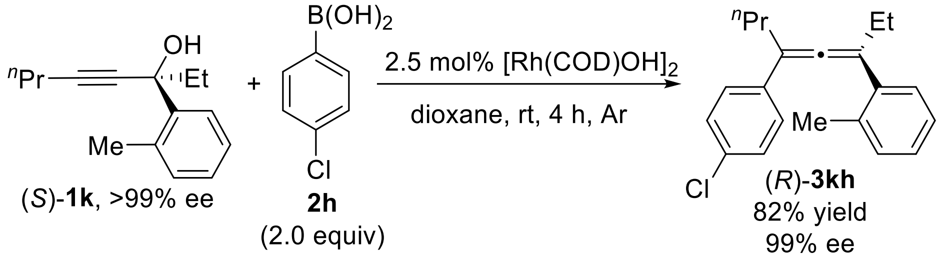

Following Typical Procedure II, the reaction of (*S*)-**1k** (43.0 mg, 0.2 mmol, >99% ee), 4-chlorophenylboronic acid **2h** (62.1 mg, 0.4 mmol), $[Rh(COD)OH]_2$ (2.4 mg, 0.005 mmol) and dioxane (1 mL) afforded (*R*)-**3kh** (50.4 mg, 82%) [eluent: petroleum ether / ethyl acetate = 60/1 (~120 mL)]: 99% ee (HPLC conditions: AD-H column, hexane/*i*-PrOH = 99.5/0.5, 0.5 mL/min, λ = 214 nm, t_R (minor) = 7.2 min, t_R (major) = 9.3 min); oil; **^{1}H NMR** (400 MHz, $CDCl_3$): δ = 7.36 (d, *J* = 8.0 Hz, 2 H, Ar-H), 7.31-7.21 (m, 3 H, Ar-H), 7.21-7.09 (m, 3 H, Ar-H), 2.49-2.36 (m, 4 H, 2 x CH_2), 2.30 (s, 3 H, CH_3), 1.67-1.52 (m, 2 H, CH_2), 1.12 (t, *J* = 7.4 Hz, 3 H, CH_3), 0.98 (t, *J* = 7.2 Hz, 3 H, CH_3); **^{13}C NMR** (100 MHz, $CDCl_3$): δ = 202.4, 138.2, 136.3, 135.7, 132.0, 130.4, 128.3, 128.2, 127.3, 126.9, 125.7, 110.2, 105.9, 32.6, 27.5, 21.4, 20.6, 14.1, 12.6; **IR** (neat): *v* = 2961, 2930, 2896, 1947, 1484, 1455, 1089, 1008 cm^{-1}; **MS** (70 eV, EI) *m/z* (%): 312 (M^+(^{37}Cl), 0.86), 310 (M(^{35}Cl), 2.55), 267 (100); **HRMS** calcd for *m/z* $C_{21}H_{23}{}^{35}Cl$ [M^+]: 310.1483, found: 310.1487.

Conditions B for exclusive *syn-β*-OH elimination

(1) Preparation of (*S*)-2,4-diphenyl-2,3-octadiene ((*S*)-3aa) (wj-4-107)

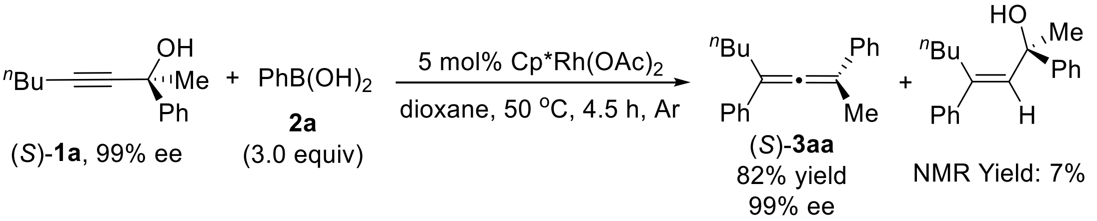

Typical Procedure III: To an oven-dried Schlenk tube (25 mL) were added phenylboronic acid **2a** (73.1 mg, 0.6 mmol) and $Cp^*Rh(OAc)_2$ (3.6 mg, 0.01 mmol). The flask was degassed and refilled with Ar for three times, and (*S*)-**1a** (40.6 mg, 0.2 mmol, 99% ee) and dioxane (1 mL) were added. The resulting mixture was vigorously stirred at 50 °C for 4.5 h as monitored by TLC, diluted with ethyl acetate (1 mL), filtered

through a short column of silica gel (3 cm), eluted with ethyl acetate (10 mL), and concentrated. The residue was purified by chromatography on silica gel to afford (*S*)-**3aa** (44.0 mg, 82%, purity: 98%) [eluent: petroleum ether (150 mL)]: 99% ee (HPLC conditions: OJ-H column, hexane/*i*-PrOH = 99.5/0.5, 0.5 mL/min, λ = 214 nm, t_R (minor) = 9.0 min, t_R (major) = 11.2 min); $[\alpha]_D^{22}$ = +339.7 (*c* = 1.56, $CHCl_3$); oil; **^{1}H NMR** (400 MHz, $CDCl_3$): δ = 7.48-7.39 (m, 4 H, Ar-H), 7.35-7.25 (m, 4 H, Ar-H), 7.24-7.15 (m, 2 H, Ar-H), 2.60-2.48 (m, 2 H, CH_2), 2.20 (s, 3 H, CH_3), 1.61-1.51 (m, 2 H, CH_2), 1.48-1.35 (m, 2 H, CH_2), 0.91 (t, *J* = 7.4 Hz, 3 H, CH_3); **^{13}C NMR** (100 MHz, $CDCl_3$): δ = 205.6, 137.2, 137.0, 128.41, 128.38, 126.7, 126.6, 126.1, 125.6, 107.8, 103.6, 30.1, 30.0, 22.6, 16.8, 14.0; **IR** (neat): *v* = 2953, 2926, 2858, 1936, 1869, 1798, 1597, 1492, 1462, 1444, 1369, 1103, 1065, 1025 cm^{-1}; **MS** (70 eV, EI) *m/z* (%): 262 (M^+, 4.27), 205 (100); **HRMS** calcd for $C_{20}H_{22}$ [M^+]: 262.1716, found: 262.1718.

(2) Preparation of (*S*)-2-phenyl-4-(4-fluorophenyl)-2,3-octadiene ((*S*)-3ag) (wj-4-158)

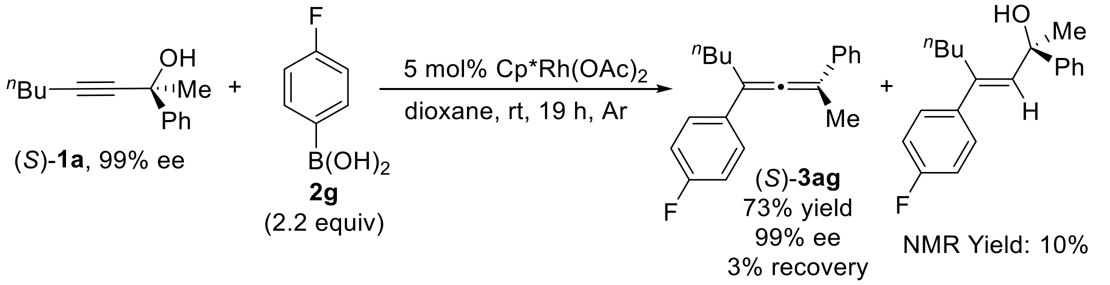

Following Typical Procedure III, the reaction of 4-fluorophenylboronic acid **2g** (61.6 mg, 0.44 mmol), Cp*Rh(OAc)$_2$ (3.6 mg, 0.01 mmol), and (*S*)-**1a** (40.4 mg, 0.2 mmol, 99% ee)/dioxane (1 mL) afforded (*S*)-**3ag** (41.1 mg, 73%) [eluent: petroleum ether (100 mL)]: 99% ee (HPLC conditions: OJ-3 (3.0 mm I.D. *150mm) column, CO_2/MeOH = 95/5, 1.0 mL/min, λ = 214 nm, t_R (minor) = 1.8 min, t_R (major) = 2.4 min); $[\alpha]_D^{25}$ = +277.7 (*c* = 1.09, $CHCl_3$); oil; **^{1}H NMR** (400 MHz, $CDCl_3$): δ = 7.48-7.40 (m, 2 H, Ar-H), 7.40-7.28 (m, 4 H, Ar-H), 7.25-7.18 (m, 1 H, Ar-H), 7.03-6.94 (m, 2 H, Ar-H), 2.58-2.44 (m, 2 H, CH_2), 2.20 (s, 3 H, CH_3), 1.61-1.50 (m, 2 H, CH_2), 1.48-1.35 (m, 2 H, CH_2), 0.91 (t, *J* = 7.2 Hz, 3 H, CH_3); **^{13}C NMR** (100 MHz, $CDCl_3$): δ = 205.2 (d, *J* = 2.4 Hz), 161.8 (d, *J* = 244.1 Hz), 137.1, 133.0 (d, *J* = 3.9 Hz), 128.4, 127.5 (d, *J* = 7.9

Hz), 126.7, 125.6, 115.2 (d, J = 21.4 Hz), 107.0, 103.8, 30.2, 30.0, 22.6, 16.8, 14.0; **^{19}F NMR** (376 MHz, $CDCl_3$): δ = -116.7; **IR** (neat): v = 2955, 2929, 2859, 1933, 1886, 1599, 1506, 1463, 1443, 1372, 1228, 1159, 1100, 1065, 1026 cm^{-1}; **MS** (70 eV, EI) *m/z* (%): 280 (M^+, 9.36), 141 (100); **HRMS** calcd *m/z* for $C_{20}H_{21}F$ [M^+]: 280.1622, found: 280.1624.

(3) Preparation of (*S*)-2-phenyl-4-(4-*tert*-butylphenyl)-2,3-octadiene ((*S*)-3ak) (wj-4-159)

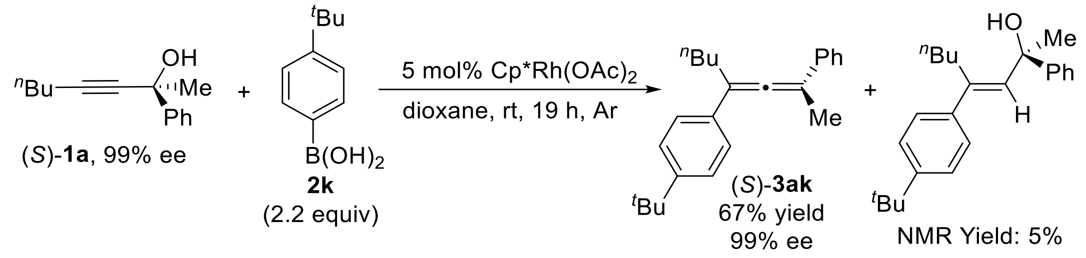

Following Typical Procedure III, the reaction of 4-*tert*-butylphenylboronic acid **2k** (78.3 mg, 0.44 mmol), Cp*Rh(OAc)$_2$ (3.7 mg, 0.01 mmol), and (*S*)-**1a** (40.5 mg, 0.2 mmol, 99% ee)/dioxane (1 mL) afforded (*S*)-**3ak** (43.0 mg, 67%) [eluent: petroleum ether (100 mL)]: 99% ee (HPLC conditions: OJ-3 (3.0 mm I.D. *150mm) column, CO_2/MeOH = 98/2, 1.0 mL/min, λ = 254 nm, t_R (minor) = 4.0 min, t_R (major) = 5.7 min); $[\alpha]_D^{25}$ = +356.1 (c = 0.94, $CHCl_3$); oil; **^{1}H NMR** (400 MHz, $CDCl_3$): δ = 7.48-7.42 (m, 2 H, Ar-H), 7.40-7.27 (m, 6 H, Ar-H), 7.23-7.16 (m, 1 H, Ar-H), 2.58-2.51 (m, 2 H, CH_2), 2.19 (s, 3 H, CH_3), 1.61-1.52 (m, 2 H, CH_2), 1.47-1.38 (m, 2 H, CH_2), 1.30 (s, 9 H, 3 x CH_3), 0.90 (t, J = 7.2 Hz, 3 H, CH_3); **^{13}C NMR** (100 MHz, $CDCl_3$): δ = 205.5, 149.7, 137.4, 134.0, 128.3, 126.5, 125.7, 125.6, 125.3, 107.5, 103.5, 34.4, 31.3, 30.1, 30.0, 22.6, 16.9, 14.0; **IR** (neat): v = 2958, 2923, 2856, 1934, 1884, 1596, 1513, 1492, 1463, 1362, 1269, 1113, 1066, 1027 cm^{-1}; **MS** (70 eV, EI) *m/z* (%): 318 (M^+, 1.37), 261 (100); **HRMS** calcd *m/z* for $C_{24}H_{30}$ [M^+]: 318.2342, found: 318.2346.

(4) Preparation of (*S*)-2-phenyl-4-(4-phenylphenyl)-2,3-octadiene ((*S*)-3al) (wj-4-163)

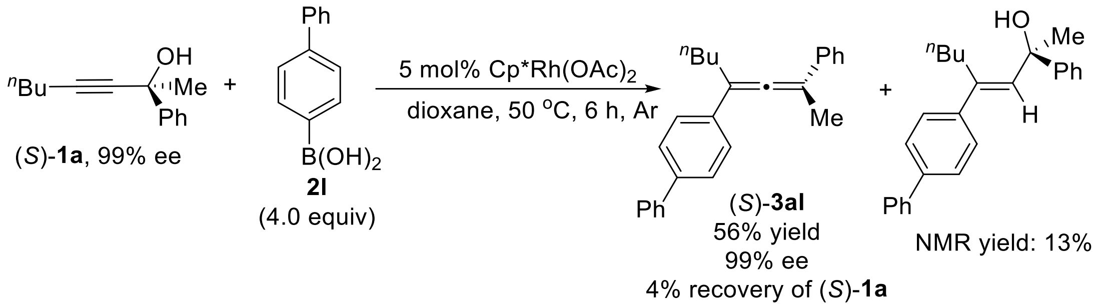

Following Typical Procedure III, the reaction of 4-biphenylboronic acid **2l** (158.7 mg, 0.8 mmol), Cp*Rh(OAc)$_2$ (3.6 mg, 0.01 mmol), and (*S*)-**1a** (40.4 mg, 0.2 mmol, 99% ee)/dioxane (1 mL) afforded (*S*)-**3al** (37.9 mg, 56%) [eluent: petroleum ether (300 mL)]: 99% ee (HPLC conditions: OD-H column, hexane/*i*-PrOH = 99.5/0.5, 1.0 mL/min, λ = 214 nm, t_R (major) = 5.1 min, t_R (minor) = 5.6 min); $[\alpha]_D^{30}$ = +476.5 (*c* = 1.07, CHCl$_3$); oil; **^{1}H NMR** (400 MHz, CDCl$_3$): δ = 7.62-7.38 (m, 10 H, Ar-H), 7.37-7.28 (m, 3 H, Ar-H), 7.21 (t, *J* = 7.2 Hz, 1 H, Ar-H), 2.64-2.52 (m, 2 H, CH$_2$), 2.23 (s, 3 H, CH$_3$), 1.59 (quint, *J* = 7.5 Hz, 2 H, CH$_2$), 1.49-1.38 (m, 2 H, CH$_2$), 0.92 (t, *J* = 7.4 Hz, 3 H, CH$_3$); **^{13}C NMR** (100 MHz, CDCl$_3$): δ = 205.8, 140.8, 139.5, 137.2, 136.0, 128.7, 128.4, 127.1, 126.9, 126.7, 126.4, 125.6, 107.5, 103.7, 30.1, 30.0, 22.6, 16.8, 14.0; **IR** (neat): *v* = 2959, 2925, 2857, 1924, 1597, 1522, 1489, 1462, 1263, 1117, 1065, 1025 cm^{-1}; **MS** (70 eV, EI) *m/z* (%): 339 (M$^+$+1, 3.40), 338 (M$^+$, 11.10), 281 (100); **HRMS** calcd *m/z* for $C_{26}H_{26}$ [M$^+$]: 338.2029, found: 338.2029.

(5) Preparation of (*S*)-2-phenyl-4-(3-(methoxycarbonyl)phenyl)-2,3-octadiene ((*S*)-3ar) (wj-4-164)

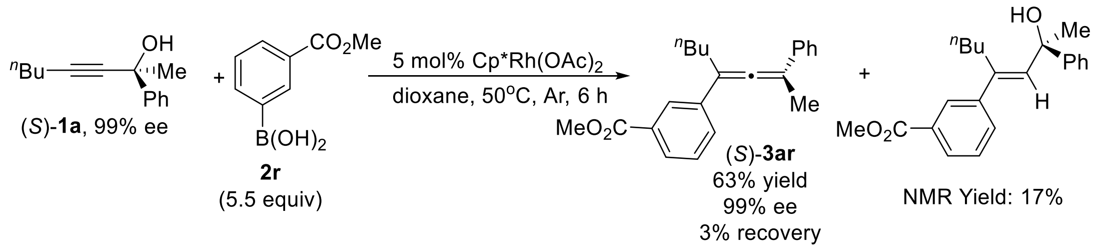

Following Typical Procedure III, the reaction of 3-(methoxycarbonyl)phenylboronic acid **2r** (74.2 mg, 0.4 mmol, and other 74.2 mg and 55.2 mg were sequentially added in 1 h and 3 h), Cp*Rh(OAc)$_2$ (3.6 mg, 0.01 mmol), and (*S*)-**1a** (40.5 mg, 0.2 mmol, 99% ee)/dioxane (1 mL) afforded (*S*)-**3ar** (40.6 mg, 63%) [eluent: petroleum ether /

ethyl ether = 50:1 (150 mL)]: 99% ee (HPLC conditions: IC column, hexane/*i*-PrOH = 99.5/0.5, 0.5 mL/min, λ = 214 nm, t_R (minor) = 16.1 min, t_R (major) = 17.2 min); $[\alpha]_D^{31}$ = +267.8 (*c* = 0.95, $CHCl_3$); oil; **^{1}H NMR** (400 MHz, $CDCl_3$): δ = 8.07 (t, *J* = 1.6 Hz, 1 H, Ar-H), 7.87 (dt, J_1 = 8.0 Hz, J_2 = 1.4 Hz, 1 H, Ar-H), 7.61 (dt, J_1 = 8.4 Hz, J_2 = 1.5 Hz, 1 H, Ar-H), 7.48-7.39 (m, 2 H, Ar-H), 7.38-7.28 (m, 3 H, Ar-H), 7.24-7.18 (m, 1 H, Ar-H), 3.91 (s, 3 H, CH_3), 2.65-2.50 (m, 2 H, CH_2), 2.23 (s, 3 H, CH_3), 1.62-1.51 (m, 2 H, CH_2), 1.49-1.37 (m, 2 H, CH_2), 0.91 (t, *J* = 7.4 Hz, 3 H, CH_3); **^{13}C NMR** (100 MHz, $CDCl_3$): δ = 205.6, 167.2, 137.6, 136.9, 130.9, 130.4, 128.41, 128.38, 127.8, 126.8, 126.6, 125.7, 107.2, 104.2, 52.1, 30.0, 29.9, 22.5, 16.8, 14.0; **IR** (neat): *ν* = 2957, 2925, 1925, 1717, 1598, 1493, 1462, 1441, 1279, 1252, 1210, 1171, 1111, 1027 cm^{-1}; **MS** (70 eV, EI) *m/z* (%): 320 (M^+, 11.02), 263 (100); **HRMS** calcd for $C_{22}H_{24}O_2$ [M^+]: 320.1771, found: 320.1771.

1.4. Preparation and characterization of the byproducts 4aa and 5aa

(1) Preparation of (*E*)-2,4-diphenyloct-3-en-2-ol (*rac*-4aa) (wj-6-110)[3]

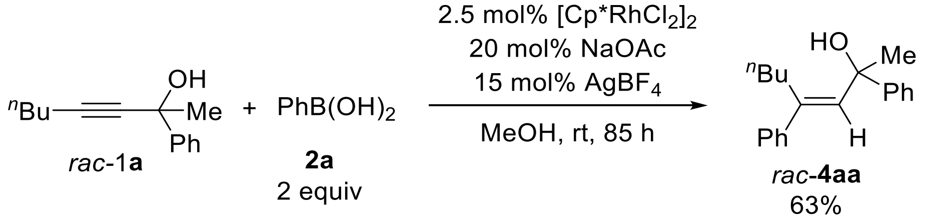

To a Schlenk flask was added phenylboronic acid **2a** (243.7 mg, 2.0 mmol), $[Cp^*RhCl_2]_2$ (15.5 mg, 0.025 mmol), NaOAc (16.4 mg, 0.2 mmol), then $AgBF_4$ (29.2 mg, 0.15 mmol) was added in the glovebox. After transferring out of the glovebox, *rac*-**1a** (202.4 mg, 1.0 mmol) and MeOH (5 mL) were added sequentially. The resulting mixture was stirred at room temperature for 85 h, filtered through a short column of silica gel (3 cm), eluted with ethyl acetate (20 mL), and concentrated. The residue was purified by chromatography on silica gel to afford *rac*-**4aa** (175.8 mg, 63%) [eluent: toluene / petroleum ether = 3:1 (400 mL)]; oil; **^{1}H NMR** (400 MHz, $CDCl_3$): δ = 7.54 (d, *J* = 7.6 Hz, 2 H, Ar-H), 7.38-7.18 (m, 8 H, Ar-H), 6.11 (s, 1 H, CH), 2.47-2.31 (m, 2 H, CH_2), 1.99 (s, 1 H, OH), 1.71 (s, 3 H, CH_3), 1.09-0.80 (m, 4 H, 2 x CH_2), 0.64 (t, *J* = 6.8 Hz, 3 H, CH_3); **^{13}C NMR** (100 MHz, $CDCl_3$): δ = 148.9, 145.3, 143.0, 135.7,

128.2, 128.1, 127.1, 126.7, 126.5, 125.2, 74.2, 34.0, 30.1, 29.9, 22.7, 13.7; **IR** (neat): *v* = 3447, 2957, 2247, 1951, 1887, 1809, 1492, 1445, 1322, 1063 cm^{-1}; **MS** (FI) *m/z* (%): 280 (M^+); **HRMS** (FI) calcd for $C_{20}H_{24}O$ [M^+]: 280.1822, found: 280.1825.

(2) Preparation of (*R*,*E*)-2,4-diphenyloct-3-en-2-ol ((*R,E*)-4aa) (zwf-7-011-2)

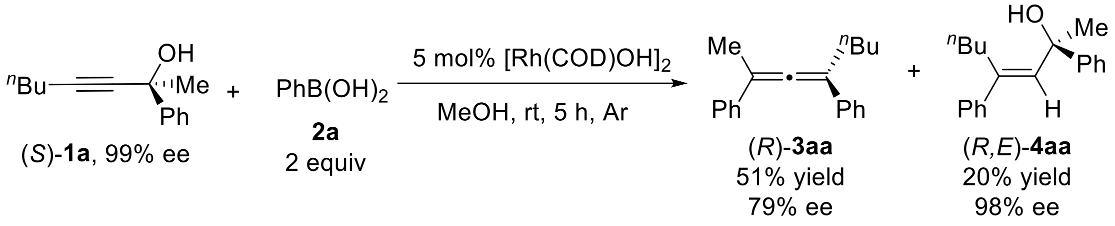

Following Typical Procedure I, the reaction of (*S*)-**1a** (101.3 mg, 0.5 mmol, 99% ee), phenylboronic acid **2a** (121.5 mg, 1.0 mmol), $[Rh(COD)OH]_2$ (11.5 mg, 0.025 mmol), and MeOH (2.5 mL) afforded (*R*,*E*)-**4aa** (28.5 mg, 20%) [eluent: petroleum ether / ethyl acetate = 60/1 (~710 mL)]: 98% ee (HPLC conditions: OD-H column, hexane/*i*-PrOH = 90/10, 1.0 mL/min, λ = 214 nm, t_R (major) = 4.8 min, t_R (minor) = 13.7 min; $[\alpha]_D^{23}$ = -53.1 (*c* = 0.95, $CHCl_3$); oil; **^{1}H NMR** (400 MHz, $CDCl_3$): δ = 7.54 (d, *J* = 7.6 Hz, 2 H, Ar-H), 7.37-7.19 (m, 8 H, Ar-H), 6.11 (s, 1 H, CH), 2.47-2.30 (m, 2 H, CH_2), 2.01 (s, 1 H, OH), 1.71 (s, 3 H, CH_3), 1.08-0.80 (m, 4 H, 2 x CH_2), 0.64 (t, *J* = 7.0 Hz, 3 H, CH_3); **^{13}C NMR** (100 MHz, $CDCl_3$): δ = 148.9, 145.2, 143.0, 135.7, 128.2, 128.1, 127.1, 126.6, 126.5, 125.2, 74.2, 34.0, 30.1, 29.9, 22.7, 13.7; **IR** (neat): *v* = 3464, 2958, 2863, 1491, 1446, 1161, 1063, 1028 cm^{-1}; **MS** (ESI) *m/z*: 263 (($M-OH)^+$); **HRMS** calcd for $C_{20}H_{23}$ [$(M-OH)^+$]: 263.1794, found: 263.1790.

(3) Preparation of (*Z*)-2,3-diphenyloct-3-en-2-ol (*rac-5aa*) (wj-4-067)

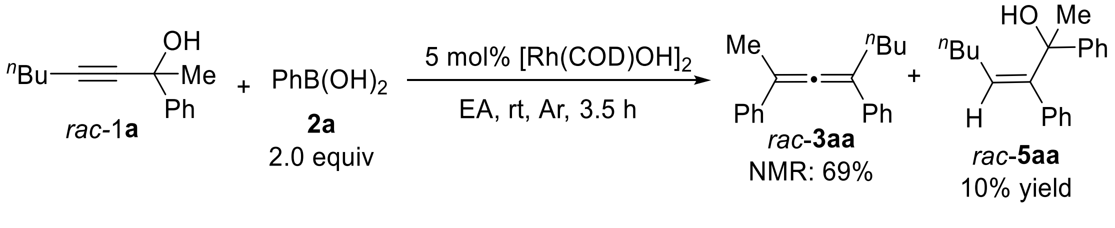

Following Typical Procedure I, the reaction of *rac*-**1a** (101.3 mg, 0.5 mmol), phenylboronic acid **2a** (121.8 mg, 1.0 mmol), $[Rh(COD)OH]_2$ (11.4 mg, 0.025 mmol), and ethyl acetate (2.5 mL) afforded *rac*-**5aa** (13.9 mg, 10%, purity: 97%) (the silica gel

was pretreated with 5% (v/v) Et_3N in petroleum ether, eluent: petroleum ether / ethyl acetate = 80/1 (500 mL)]; oil; **^{1}H NMR** (400 MHz, $CDCl_3$): δ = 7.63-7.54 (m, 2 H, Ar-H), 7.40-7.32 (m, 2 H, Ar-H), 7.32-7.18 (m, 6 H, Ar-H), 5.45 (t, J = 7.4 Hz, 1 H, CH), 2.04-1.87 (m, 3 H, OH and CH_2), 1.66 (s, 3 H, CH_3), 1.24-1.08 (m, 4 H, 2 x CH_2), 0.76 (t, J = 7.0 Hz, 3 H, CH_3); **^{13}C NMR** (100 MHz, $CDCl_3$): δ = 148.4, 147.0, 143.5, 134.2, 129.0, 128.2, 127.8, 126.9, 126.6, 125.7, 76.5, 31.6, 31.4, 29.2, 22.3, 13.8; **IR** (neat): *v* = 3441 (br), 2958, 2924, 1948, 1599, 1490, 1444, 1373, 1110, 1064, 1027 cm^{-1}; **MS** (ESI) *m/z*: 303 ($M+Na^+$); **HRMS** (ESI) calcd for $C_{20}H_{24}ONa$ [$M+Na^+$]: 303.1719, found: 303.1719.

(4) Preparation of (*S,Z*)-2,3-diphenyloct-3-en-2-ol ((*S,Z*)-5aa)(wj-4-053-5)

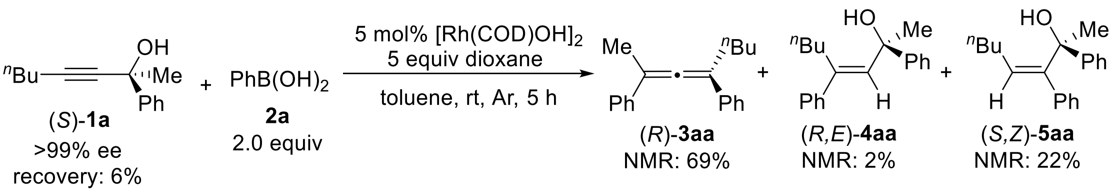

To an oven-dried Schlenk tube were added phenylboronic acid **2a** (48.8 mg, 0.4 mmol) and $[Rh(COD)OH]_2$ (4.6 mg, 0.01 mmol). The flask was degassed and refilled with Ar for three times, and (*S*)-**1a** (40.3 mg, 0.2 mmol, >99% ee)/toluene (1 mL) and dioxane (85.2 μL, d = 1.034 g/mL, 88.1 mg, 1.0 mmol) were added sequentially. The resulting mixture was vigorously stirred at room temperature for 5 h, diluted with ethyl acetate (1 mL), filtered through a short column of silica gel (3 cm), eluted with ethyl acetate (10 mL), and concentrated. (*S,Z*)-**5aa** was separated by preparative TLC (the silica gel was pretreated with 5% (v/v) Et_3N in petroleum ether)]: >99% ee (HPLC conditions: IC column, hexane/*i*-PrOH = 99/1, 0.5 mL/min, λ = 214 nm, t_R (major) = 11.4 min); oil; **^{1}H NMR** (400 MHz, $CDCl_3$): δ = 7.64-7.52 (m, 2 H, Ar-H), 7.41-7.32 (m, 2 H, Ar-H), 7.32-7.18 (m, 6 H, Ar-H), 5.45 (t, J = 7.4 Hz, 1 H, CH), 2.06-1.85 (m, 3 H, OH and CH_2), 1.67 (s, 3 H, CH_3), 1.24-1.08 (m, 4 H, 2 x CH_2), 0.76 (t, J = 7.0 Hz, 3 H, CH_3); **^{13}C NMR** (100 MHz, $CDCl_3$): δ = 148.4, 147.0, 143.5, 134.2, 129.0, 128.2, 127.8, 126.9, 126.6, 125.7, 76.5, 31.6, 31.4, 29.2, 22.3, 13.8; **IR** (neat): *v* = 3453 (br), 2956, 2923, 1948, 1599, 1490, 1444, 1372, 1310, 1064, 1028 cm^{-1}; **MS** (ESI) *m/z*: 303 ($M+Na^+$);

HRMS (ESI) calcd for $C_{20}H_{24}ONa$ [M+Na$^+$]: 303.1719, found: 303.1717.

1.5. Mechanistic studies

(1) Preparation of 2-phenyloct-3-yn-2-yl 3,4,5-trimethoxybenzoate (*rac*-1a') (zwf-6-114)

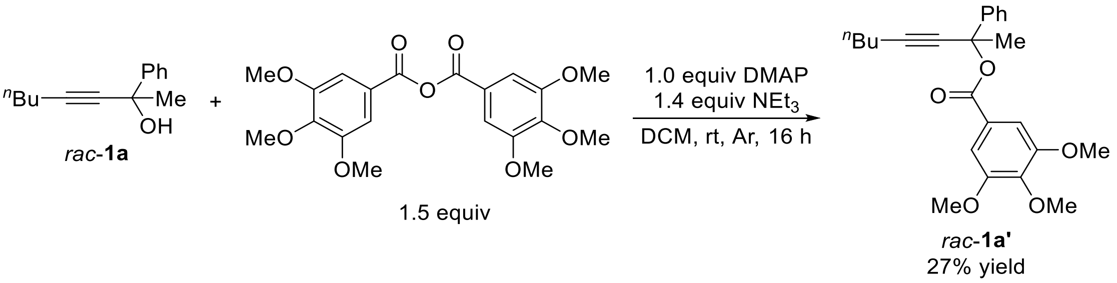

Typical Procedure IV: To a Schlenk tube (25 mL) were added DMAP (122.5 mg, 1.0 mmol), triethylamine (145.6 mg, 208 μL, d = 0.7 g/mL, 1.4 mmol), *rac*-**1a** (201.9 mg, 1.0 mmol), DCM (5 mL), and 3,4,5-trimethoxybenzoic anhydride (610.3 mg, 1.5 mmol) sequentially. The resulting mixture were vigorously stirred at room temperature for 16 h as monitored by TLC. The resulting mixture was quenched with 3 mL of H_2O and extracted with 3 x 5 mL of ethyl acetate. The combined organic layer was washed with 5 mL aqueous saturated solution of NaCl, dried over anhydrous Na_2SO_4, filtered, and concentrated under reduced pressure. The residue was purified by chromatography on silica gel to afford *rac*-**1a'** (105.9 mg, 27%) [eluent: petroleum ether / ethyl ether / dichloromethane = 30/1/1 (~640 mL), then 10/1/1 (~360 mL)]: solid; m.p. 58.0-58.6 °C (petroleum ether/ethyl acetate); **^{1}H NMR** (400 MHz, $CDCl_3$): δ = 7.64 (d, J = 7.6 Hz, 2 H, Ar-H), 7.39-7.23 (m, 5 H, Ar-H), 3.95-3.81 (m, 9 H, 3 x OCH_3), 2.34 (t, J = 7.0 Hz, 2 H, CH_2), 2.01 (s, 3 H, CH_3), 1.64-1.52 (m, 2 H, CH_2), 1.52-1.39 (m, 2 H, CH_2), 0.93 (t, J = 7.2 Hz, 3 H, CH_3); **^{13}C NMR** (100 MHz, $CDCl_3$): δ = 163.7, 152.8, 143.2, 142.1, 128.2, 127.6, 125.9, 124.8, 106.9, 88.4, 79.4, 76.8, 60.8, 56.1, 32.5, 30.5, 21.9, 18.5, 13.5; **IR** (neat): v = 2957, 2942, 2251, 1711, 1585, 1449, 1380, 1226, 1124, 1059 cm^{-1}; **MS** (70 eV, EI) m/z (%): 397 (M$^+$+1, 2.49), 396 (M$^+$, 5.81), 212 (100); Anal. Calcd. for $C_{24}H_{28}O_5$: C 72.71, H 7.12; found C 72.54, H 7.27.

(2) Preparation of (*S*)-2-phenyloct-3-yn-2-yl 3,4,5-trimethoxybenzoate ((*S*)-1a') (zwf-6-123-2)

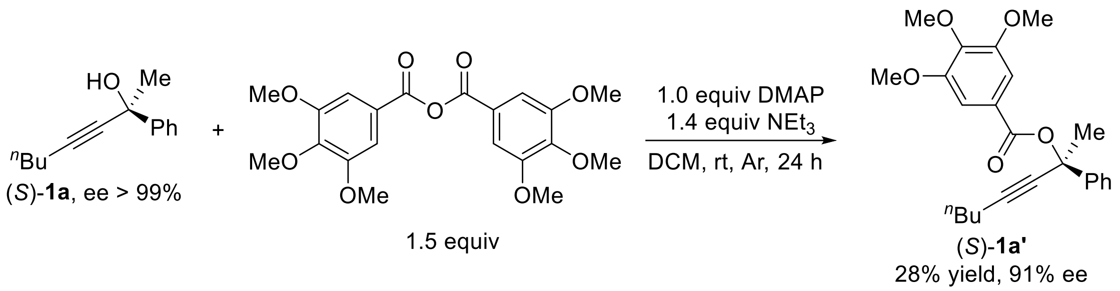

Following Typical Procedure IV, the reaction of DMAP (122.3 mg, 1.0 mmol), triethylamine (145.6 mg, 208 μL, d = 0.7 g/mL, 1.4 mmol), (*S*)-**1a** (201.5 mg, >99% ee, 1.0 mmol), DCM (5 mL), and 3,4,5-trimethoxybenzoic anhydride (610.0 mg, 1.5 mmol) afforded (*S*)-**1a'** (109.0 mg, 28%) [eluent: petroleum ether / ethyl ether / dichloromethane = 30/1/1 (~320 mL), then petroleum ether / ethyl acetate 10/1 (~330 mL)]: 91% ee (HPLC conditions: AD-H column, hexane/*i*-PrOH = 98/2, 1.0 mL/min, λ = 214 nm, t_R (major) = 8.6 min, t_R (minor) = 13.4 min); solid; m.p. 60.0-60.4 °C (petroleum ether / ethyl acetate); **^{1}H NMR** (400 MHz, $CDCl_3$): δ = 7.64 (d, *J* = 7.2 Hz, 2 H, Ar-H), 7.40-7.21 (m, 5 H, Ar-H), 3.96-3.81 (m, 9 H, 3 x OCH_3), 2.34 (t, *J* = 7.0 Hz, 2 H, CH_2), 2.01 (s, 3 H, CH_3), 1.63-1.52 (m, 2 H, CH_2), 1.52-1.37 (m, 2 H, CH_2), 0.93 (t, *J* = 7.2 Hz, 3 H, CH_3); **^{13}C NMR** (100 MHz, $CDCl_3$): δ = 163.7, 152.8, 143.2, 142.1, 128.2, 127.6, 125.9, 124.9, 106.9, 88.4, 79.4, 76.8, 60.8, 56.1, 32.5, 30.5, 21.9, 18.5, 13.5; **IR** (neat): *ν* = 2957, 2942, 2251, 1710, 1585, 1449, 1380, 1225, 1123, 1058 cm^{-1}; **MS** (70 eV, EI) *m/z* (%): 396 (M^+, 8.34), 212 (100); Anal. Calcd. for $C_{24}H_{28}O_5$: C 72.71, H 7.12; found C 72.49, H 7.04.

(3) Preparation of 1,3-diphenyl-1-(4-formylphenyl)-1,2-butadiene (*rac*-3bb) and (*E*)-2-phenyl-4-(4-formylphenyl)-oct-3-en-2-ol ((*E*)-4bb) (zwf-7-057)

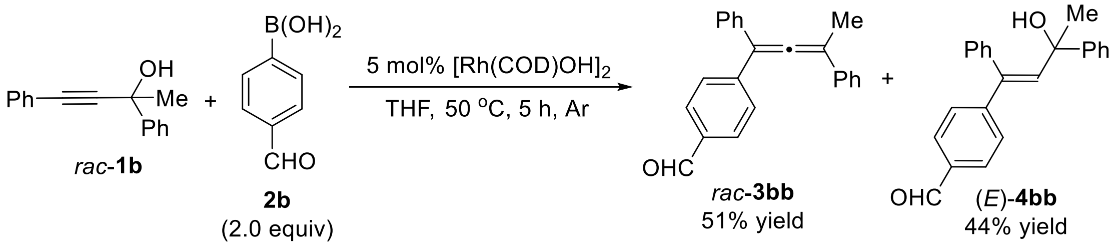

Following Typical Procedure I, the reaction of *rac*-**1b** (44.3 mg, 0.2 mmol), 4-formylphenylboronic acid **2b** (60.2 mg, 0.4 mmol), $[Rh(COD)OH]_2$ (4.4 mg, 0.01

mmol) and THF (1 mL). The reaction afforded a mixture of *rac*-**3bb** (54% NMR yield), and (*E*)-**4bb** (49% NMR yield), which were determined by ^{1}H NMR analysis of the crude product with CH_2Br_2 (14 μL) as the internal standard. The residue was purified by chromatography on silica gel to afford the pure product *rac*-**3bb** (31.3 mg, 51%) and (*E*)-**4bb** (28.6 mg, 44%) [eluent: petroleum ether / ethyl acetate = 50/1 (~300 mL), 15/1 (~320 mL)].

rac-**3bb**: oil; **^{1}H NMR** (400 MHz, $CDCl_3$): δ = 10.00 (s, 1 H, CHO), 7.84 (d, *J* = 8.4 Hz, 2 H, Ar-H), 7.55 (d, *J* = 8.4 Hz, 2 H, Ar-H), 7.49 (d, *J* = 8.0 Hz, 2 H, Ar-H), 7.42-7.27 (m, 7 H, Ar-H), 7.27-7.22 (m, 1 H, Ar-H), 2.32 (s, 3 H, CH_3); **^{13}C NMR** (100 MHz, $CDCl_3$): δ = 208.6, 191.7, 143.6, 136.1, 135.7, 135.2, 129.9, 128.8, 128.64, 128.63, 128.5, 127.7, 127.4, 125.8, 111.3, 104.6, 16.8; **IR** (neat): *v* = 3025, 2825, 1927, 1698, 1568, 1460, 1210, 1165 cm^{-1}; **MS** (70 eV, EI) *m/z* (%): 311 (M^++1, 1.05), 310 (M^+, 3.98), 84 (100); **HRMS** calcd for *m/z* $C_{23}H_{18}O$ [M^+]: 310.1352, found: 310.1356.

(*E*)-**4bb**: oil; **^{1}H NMR** (400 MHz, $CDCl_3$): δ = 9.98 (s, 1 H, CHO), 7.78 (d, *J* = 8.4 Hz, 2 H, Ar-H), 7.45 (d, *J* = 7.6 Hz, 2 H, Ar-H), 7.39 (d, *J* = 8.0 Hz, 2 H, Ar-H), 7.33-7.13 (m, 8 H, Ar-H), 6.73 (s, 1 H, =CH), 2.25 (s, 1 H, OH), 1.58 (s, 3 H, CH_3); **^{13}C NMR** (100 MHz, $CDCl_3$): δ = 191.9, 149.8, 148.4, 147.0, 137.4, 134.9, 132.4, 129.8, 129.1, 128.4, 128.2, 128.1, 127.3, 127.0, 126.0, 77.5, 31.1; **IR** (neat): *v* = 3481, 3022, 2830, 1697, 1601, 1445, 1170, 1067 cm^{-1}; **MS** (70 eV, EI) *m/z* (%): 329 (M^++1, 1.05), 328 (M^+, 4.18), 208 (100); **HRMS** calcd for *m/z* $C_{23}H_{20}O_2$ [M^+]: 328.1458, found: 328.1457.

(4) Preparation of (*R*)-1,3-diphenyl-1-(4-formylphenyl)-1,2-butadiene ((*R*)-3bb) and (*R*, *E*)-2-phenyl-4-(4-formylphenyl)-oct-3-en-2-ol ((*R*, *E*)-4bb) (zwf-7-064)

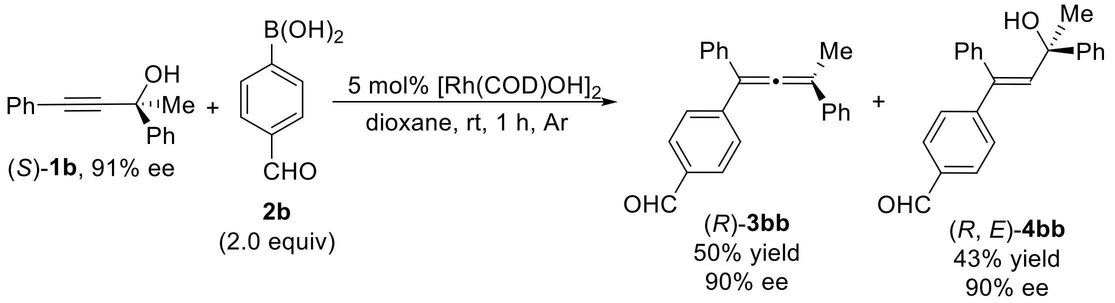

Following Typical Procedure II, the reaction of (*S*)-**1b** (44.2 mg, 0.2 mmol, 91% ee), 4-formylphenylboronic acid **2b** (60.5 mg, 0.4 mmol), $[Rh(COD)OH]_2$ (4.7 mg, 0.01

mmol) and dioxane (1 mL) afforded a mixture of (*R*)-**3bb** (52% NMR yield), and (*R*, *E*)-**4bb** (47% NMR yield), which were determined by ^{1}H NMR analysis of the crude product with CH_2Br_2 (14 μL) as the internal standard. The residue was purified by chromatography on silica gel to afford the pure product (*R*)-**3bb** (30.6 mg, 50%) and (*R*, *E*)-**4bb** (28.4 mg, 43%) [eluent: petroleum ether / ethyl acetate = 50/1 (~300 mL), 15/1 (~320 mL)].

(*R*)-**3bb**: 90% ee (HPLC conditions: OD-H column, hexane/*i*-PrOH = 98/2, 1.0 mL/min, λ = 214 nm, t_R (minor) = 7.8 min, t_R (major) = 12.4 min); oil; **^{1}H NMR** (400 MHz, $CDCl_3$): δ = 9.99 (s, 1 H, CHO), 7.84 (d, *J* = 8.4 Hz, 2 H, Ar-H), 7.55 (d, *J* = 8.4 Hz, 2 H, Ar-H), 7.49 (d, *J* = 7.6 Hz, 2 H, Ar-H), 7.43-7.28 (m, 7 H, Ar-H), 7.28-7.22 (m, 1 H, Ar-H), 2.32 (s, 3 H, CH_3); **^{13}C NMR** (100 MHz, $CDCl_3$): δ = 208.6, 191.7, 143.6, 136.1, 135.7, 135.3, 129.9, 128.8, 128.65, 128.63, 128.5, 127.7, 127.4, 125.8, 111.3, 104.6, 16.8; **IR** (neat): *v* = 3026, 2824, 1928, 1698, 1568, 1418, 1210, 1168 cm^{-1}; **MS** (70 eV, EI) *m/z* (%): 311 (M^++1, 25.44), 310 (M^+, 100), 310 (100); **HRMS** calcd for *m/z* $C_{23}H_{18}O$ [M^+]: 310.1352, found: 310.1356.

(*R*, *E*)-**4bb**: 90% ee (HPLC conditions: AD-H column, hexane/*i*-PrOH = 90/10, 1.0 mL/min, λ = 214 nm, t_R (minor) = 17.2 min, t_R (major) = 19.6 min); oil; **^{1}H NMR** (400 MHz, $CDCl_3$): δ = 9.98 (s, 1 H, CHO), 7.78 (d, *J* = 8.0 Hz, 2 H, Ar-H), 7.45 (d, *J* = 7.2 Hz, 2 H, Ar-H), 7.39 (d, *J* = 8.4 Hz, 2 H, Ar-H), 7.32-7.15 (m, 8 H, Ar-H), 6.73 (s, 1 H, =CH), 2.25 (s, 1 H, OH), 1.58 (s, 3 H, CH_3); **^{13}C NMR** (100 MHz, $CDCl_3$): δ = 191.9, 149.8, 148.4, 147.0, 137.4, 134.9, 132.4, 129.8, 129.1, 128.4, 128.2, 128.1, 127.3, 127.0, 126.0, 77.5, 31.1; **IR** (neat): *v* = 3469, 3024, 2831, 1697, 1601, 1445, 1170, 1068 cm^{-1}; **MS** (70 eV, EI) *m/z* (%): 329 (M^++1, 1.02), 328 (M^+, 3.87), 208 (100); **HRMS** calcd for *m/z* $C_{23}H_{20}O_2$ [M^+]: 328.1458, found: 328.1458.

(5) Preparation of (*S*)-*N*-methoxy-2-(2-phenyl-2,3-octadien-4-yl)benzamide ((*S*)-16) (zwf-5-083)

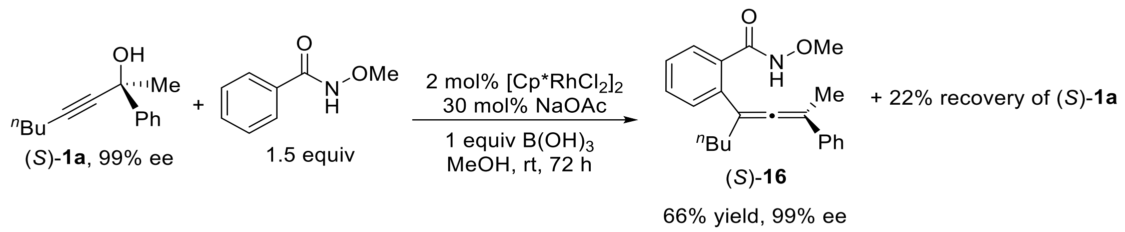

To a Schlenk tube were added *N*-methoxybenzamide (45.9 mg, 0.3 mmol), [Cp*$RhCl_2$]$_2$ (2.4 mg, 0.004 mmol), NaOAc (5.0 mg, 0.06 mmol), $B(OH)_3$ (12.5 mg, 0.2 mmol), (*S*)-**1a** (40.3 mg, 0.2 mmol, 99% ee), and MeOH (1.2 mL) sequentially at room temperature. The reaction was stirred for 72 h at room temperature. After filtration through a short column of silica gel (eluent: ethyl acetate 20 mL) and evaporation under reduced pressure, the crude product was purified by flash column chromatography on silica gel to afford (*S*)-**16**[1] (43.9 mg, 66%) as an oil [eluent: petroleum ether/ethyl acetate = 10/1 (220 mL), 7/1 (160 mL), 5/1 (180 mL)]: 99% ee (HPLC conditions: IC column, hexane/*i*-PrOH = 90/10, 1.0 mL/min, λ = 214 nm, t_R (major) = 10.6 min, t_R (minor) = 11.6 min); **^{1}H NMR** (400 MHz, $CDCl_3$): δ = 8.64 (br, 1 H, NH), 7.52 (d, *J* = 7.2 Hz, 1 H, Ar-H), 7.44-7.36 (m, 3 H, Ar-H), 7.36-7.24 (m, 4 H, Ar-H), 7.21 (t, *J* = 7.2 Hz, 1 H, Ar-H), 3.84 (s, 3 H, OCH_3), 2.55-2.33 (m, 2 H, CH_2), 2.19 (s, 3 H, CH_3), 1.54-1.43 (m, 2 H, CH_2), 1.43-1.32 (m, 2 H, CH_2), 0.88 (t, *J* = 7.2 Hz, 3 H, CH_3); **^{13}C NMR** (100 MHz, CDCl3): δ = 203.8, 167.6, 137.6, 137.2, 131.6, 130.7, 129.4, 128.8, 128.4, 127.1, 126.8, 125.7, 107.5, 102.6, 64.3, 33.8, 30.0, 22.3, 16.8, 13.9.

(6) Investigation on the effect of H_2O by using dried starting materials

Detail experimental procedure for removing moisture

To investigate the effect of water, the starting materials and 1,4-dioxane had been carefully dried according to the following procedure: (*S*)-**1a** (600 mg) was dissolved in 5 mL of anhydrous benzene and evaporated under vacuum for three times to remove moisture via azeotropic distillation and then transferred into the glovebox and opened to further remove the trace amount of H_2O for two days before use. Phenylboronic acid **2a** was recrystallized from ethyl acetate and transferred into the glovebox and opened to further remove the trace amount of H_2O for one day before use. 1,4-Dioxane was distilled from sodium wire with benzophenone as the indicator, further treated with

LiAlH4, distilled, and transferred into the glovebox for direct use.

Then following Typical Procedure II, the reactions of (*S*)-**1a** (0.2 mmol, 96% ee), phenylboronic acid **2a** (0.4 mmol) or triphenylboroxin (0.13 mmol), [Rh(COD)OH]2 (0.005 mmol) with different amounts of additives in dioxane (1 mL) at rt were conducted to collect the data.

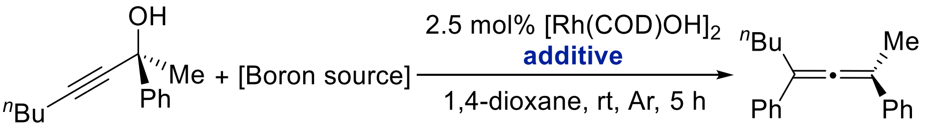

(*S*)-**1a**, 96% ee **2a** (2.0 equiv) (*R*)-**3aa**

entry	[Boron source]	additive	NMR yield, ee, es of (*R*)-**3aa** (%)	recovery of (*S*)-**1a** (%)
1	$PhB(OH)_2$	1.0 equiv H_2O	96, 96, >99	0
2	$PhB(OH)_2$	0.5 equiv H_2O	95, 96, >99	0
3	$PhB(OH)_2$	0.2 equiv H_2O	93, 96, >99	0
4	$PhB(OH)_2$	*none*	93, 93, 97	0
5	$(PhBO)_3$	*none*	89, 93, 97	0
6	$PhB(OH)_2$	20 mg 4Å MS	54, 85, 89	45
7	$PhB(OH)_2$	40 mg 4Å MS	49, 78, 81	49

(7) Study on the effect of ether

Following Typical Procedure II, the reactions of (*S*)-**1a** (0.2 mmol, >99% ee), phenylboronic acid **2a** (0.4 mmol), and [Rh(COD)OH]2 (0.005 mmol) with different amounts of 1,4-dioxane in toluene (1 mL) at rt were conducted to collect the data.

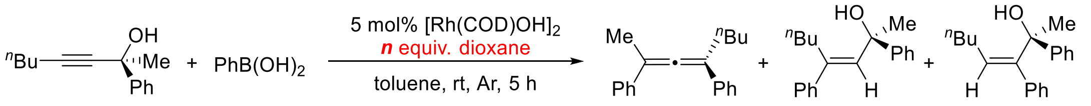

(*S*)-**1a**, >99% ee **2a** (2.0 equiv.) (*R*)-**3aa** (*R*)-**4aa** (*S*)-**5aa**

entry	*n*	yield, ee of (*R*)-**3aa** (%)	recovery of (*S*)-**1a** (%)	yield of (*R*)-**4aa** (%)	yield of (*S*)-**5aa** (%)
1	0	19, 92	57	1	9
2	0.1	52, 95	21	2	16
3	0.5	55, 95	22	2	18
4	1.0	58, 95	19	2	18
5	3.0	58, 97	15	2	20
6	5.0	59, 97	6	2	22

1.6. Detail studies on absolute configuration determination

Recently, Dou and coworkers reported a Rh-catalyzed *syn* β-OH elimination reaction of chiral propargylic alcohol with triphenylboroxin to chiral allene.[4] They had assigned the absolute configuration of their chiral allene product to *S* based on the ***Lowe-Brewster rule*** analysis.

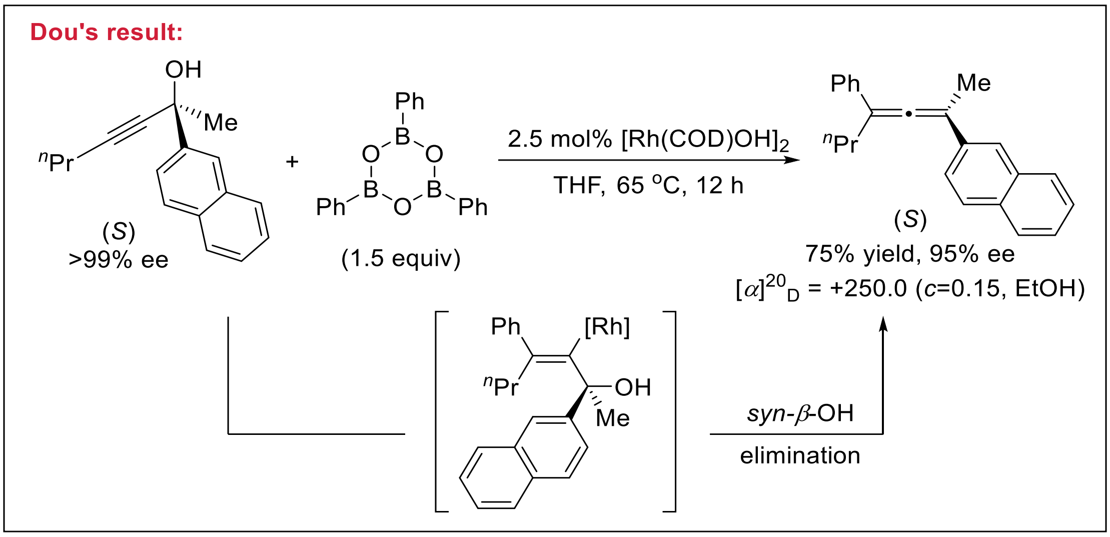

The absolute configuration of a chiral allene is related to the sign of its optical rotation at the sodium D-line by the rule proposed by Lowe and developed by Brewster.[5,6] The ***Lowe-Brewster rule*** states that if the most polarizable substituent (R^1) of a chiral allene is aligned uppermost along a vertical axis, and the more polarizable (R^3) of the two rear substituents in the horizontal plane is disposed on the right-hand side, a clockwise screw pattern of polarizability obtains, and the isomer is dextrorotatory (+) (Supplementary Figure 1). Conversely, if the more polarizable of the two rear substituents in the horizontal plane is disposed to the left, the allene is laevorotatory (-).

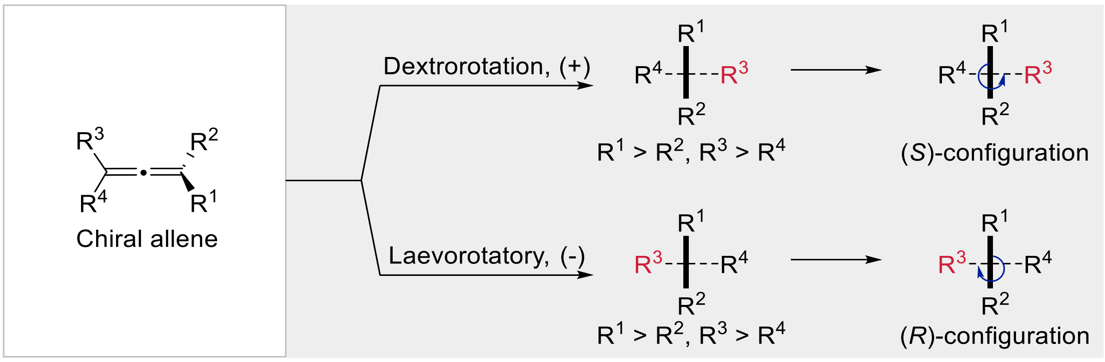

Supplementary Figure 1: The Lowe-Brewster rule.

We first confirmed the absolute configuration of our chiral propargylic alcohols. The esterification reaction of the chiral tertiary propargylic alcohol (*S*)-**1a** with 3,4,5-trimethoxybenzoic anhydride yielded the (*S*)-**1a'** in 28% yield with 91% ee. The absolute configuration of (*S*)-**1a'** was firmly established by the single crystal X-ray crystallography. In this transformation the stereocenter of chiral tertiary propargylic alcohol (*S*)-**1a** was remained.

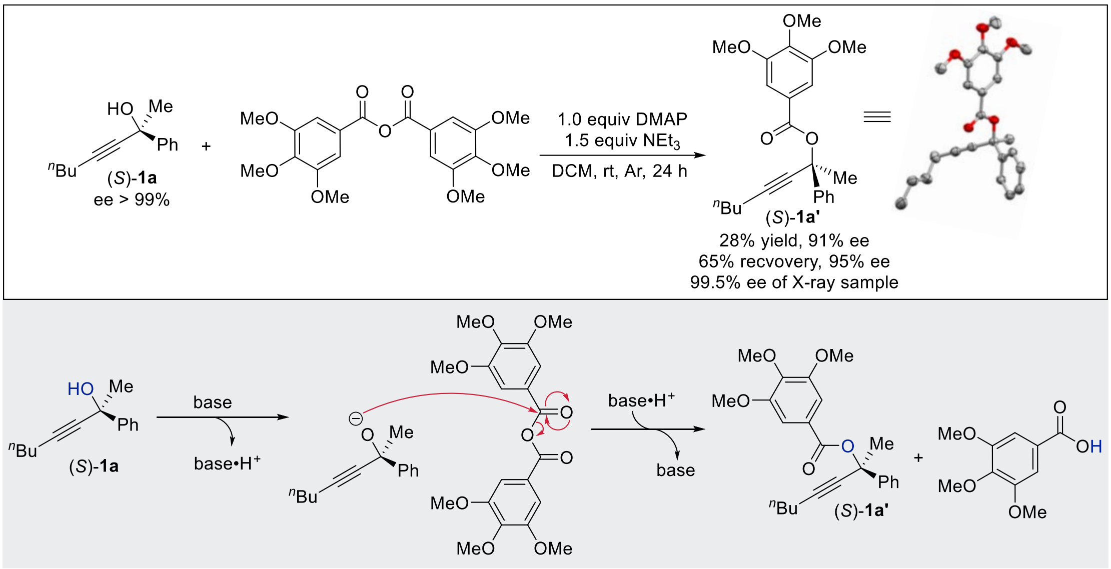

On the other hand, Nakajima and coworkers determined the absolute configuration of chiral tertiary propargylic alcohols by transferred them to a series of unambiguous chiral esters.[7,8]

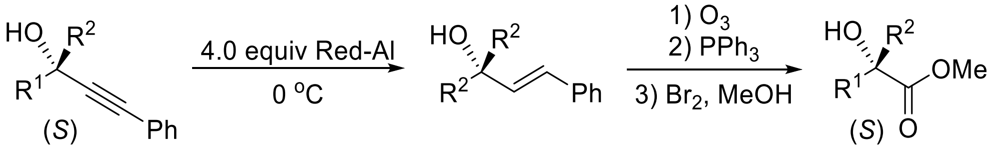

R^1 = Ph, R^2 = Et; R^1 = 4-$CH_3C_6H_4$, R^2 = Me; R^1 = 4-$CF_3C_6H_4$, R^2 = Me;
R^1 = 2-Naphthyl, R^2 = Me; R^1 = $PhCH_2CH_2$, R^2 = Me;

Nakajima and coworkers reported the chiral propargylic alcohol (*S*)-**1a** with 90% ee (HPLC conditions: Daicel Chiralcel OD-H; eluent, hexane/*i*-PrOH = 200/1; flow rate: 1.0 mL/min, 254 nm, t_R (minor) = 20.7 min, t_R (major) = 22.5 min).

File Name k4085r-0%ee.crm

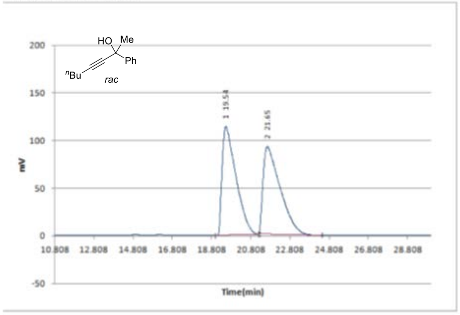

comment OD-H_Hex/IPA=200/1_flow=1.0 mL/min_UL

Date 2013/6/3 12:20

No.	Rt	Peak Name	Area	Area(%)	Height	Amount	NTP	Tf	Resolution
1	19.54		5575289	50.1966	113604	-----	3278.4	2.315	1.391
2	21.65		5531623	49.8034	92003	-----	2678.4	2.588	-----
			11106911	100	205607				

File Name k4085b-90%ee.crm

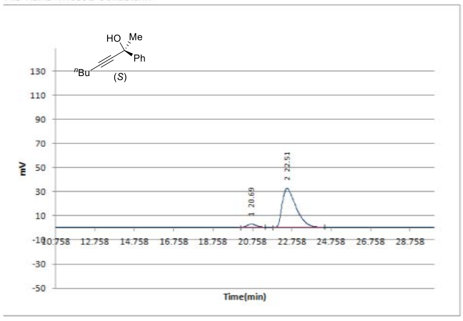

comment OD-H_Hex/IPA=200/1_flow=1.0 mL/min_UL

Date 2013/6/3 11:40

No.	Rt	Peak Name	Area	Area(%)	Height	Amount	NTP	Tf	Resolution
1	20.69		80604	4.9267	2439	-----	8762.6	1.144	1.65
2	22.51		1555458	95.0733	32250	-----	4672.5	1.765	-----
			1636062	100	34689				

We prepared the chiral propargylic alcohol (*S*)-**1a** with >99% ee (HPLC conditions: Daicel Chiralcel OD-H; eluent, hexane/*i*-PrOH = 200/1; flow rate: 1.0 mL/min, 214 nm, t_R (minor) = 19.0 min, t_R (major) = 20.8 min).

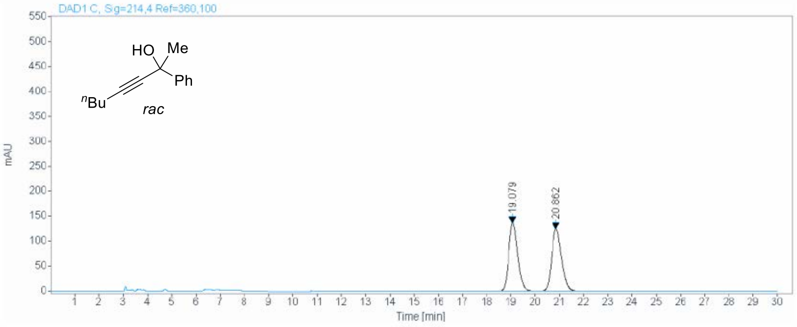

Signal: DAD1 C, Sig=214,4 Ref=360,100

RT [min]	Width [min]	Height	Area	Area%
19.079	0.3970	136.6629	3502.5771	50.1025
20.862	0.4385	123.8741	3488.2429	49.8975
		Sum	6990.8201	100.0000

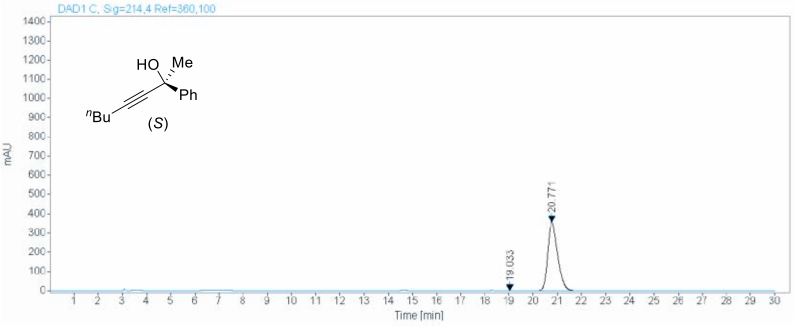

Signal: DAD1 C, Sig=214,4 Ref=360,100

RT [min]	Width [min]	Height	Area	Area%
19.033	0.3564	0.4432	9.4785	0.0927
20.771	0.4424	356.3593	10215.3369	99.9073
		Sum	10224.8154	100.0000

Based on these two solid evidences, the absolute configuration of chiral tertiary propargylic alcohols were determined to be *S*.

Then, we prepared Dou's same substrate (*S*)-**1l** (99% ee)[9] and tried the reaction of (*S*)-**1l** with **2a** under our optimal reaction conditions, resluting in the formation of (*R*)-**3la** in 82% yield with 96% ee (Supplementary Figure 2, eq. (2)). We also tried the same reaction under the reaction conditions reported by Dou et al. and found the same product (*R*)-**3la** was formed in 83% yield with 95% ee (Supplementary Figure 2, eq. (3)).

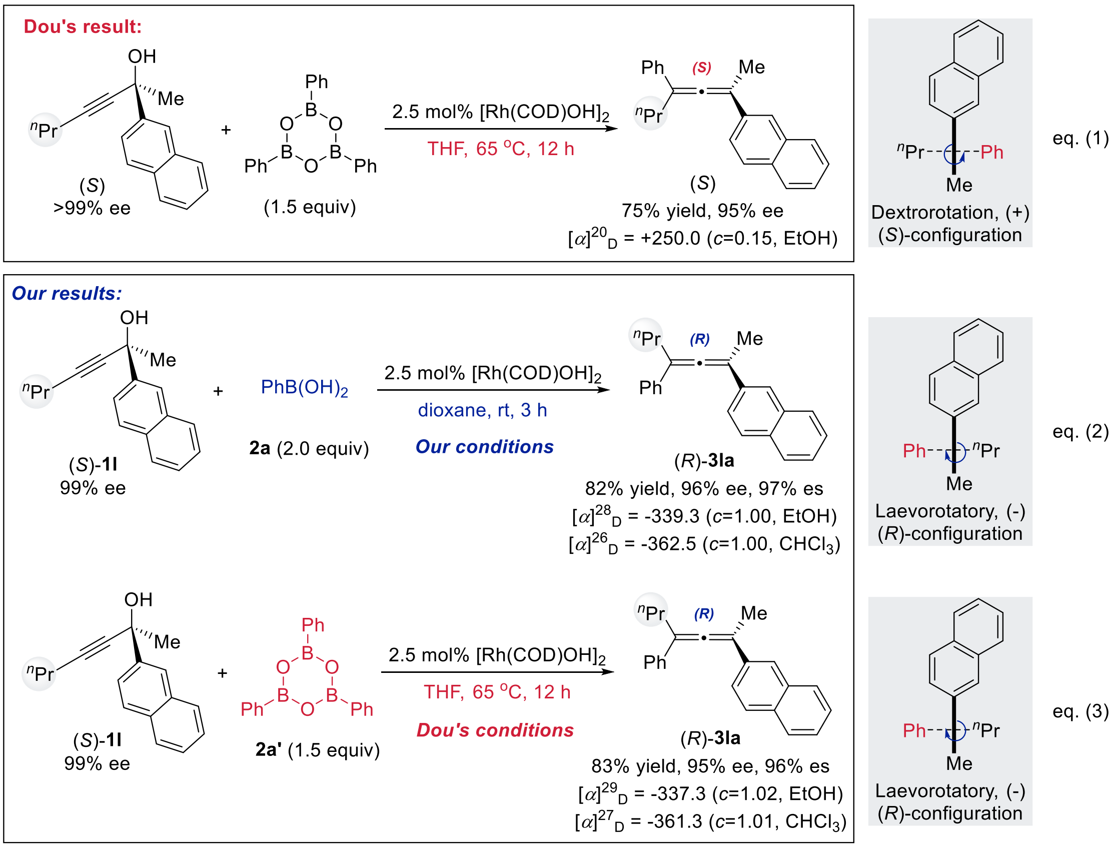

Supplementary Figure 2: The absolute configuration determination via Lowe-Brewster rule.

Furthermore, we also found that when the boronic acid **2d** or boroxin **2d'** was submitted to Dou's optimal conditions, (*R*)-**3ad** was unambiguously obtained in good yield with high ee (Supplementary Figure 3, eqs. (2) and (3)).

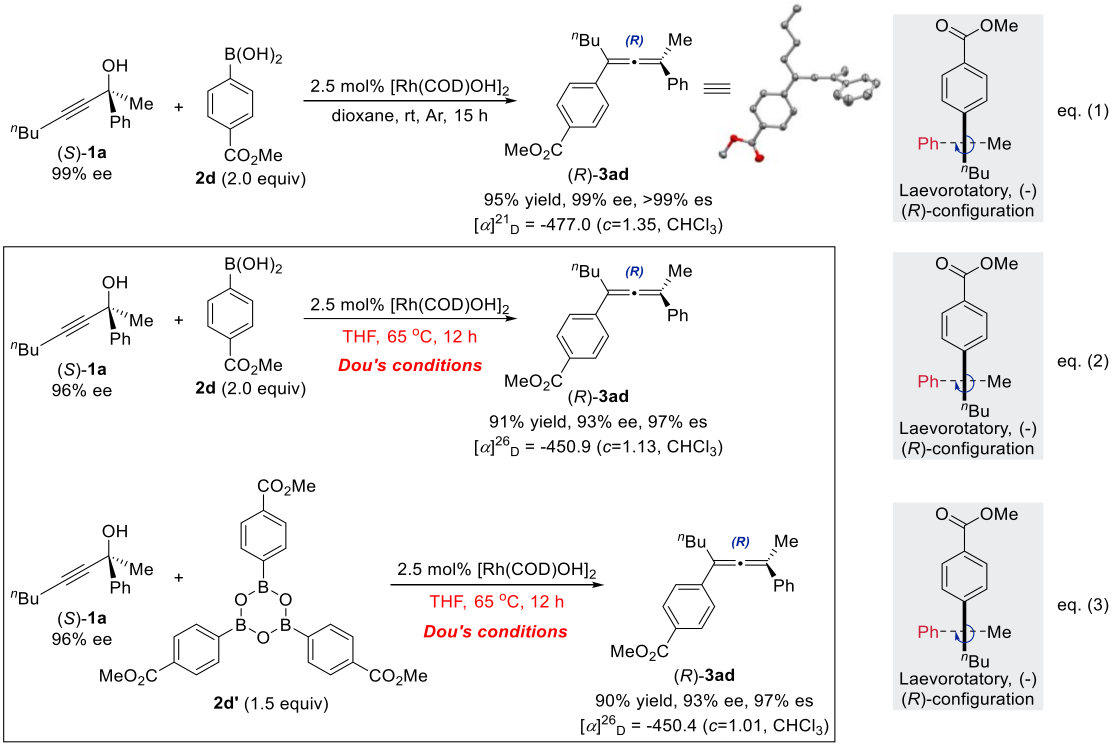

Supplementary Figure 3: The absolute configuration determination of (*R*)-3ad via the single crystal X-ray crystallography.

To double check the stereochemical outcome of this transformation, we tried to demonstrate the stereochemistry by using propargylic alcohol (*S*)-**1j** containing the heavy atom of bromine, as the substrate. The reaction of (*S*)-**1j** and **2d** under the catalysis of $[Rh(COD)OH]_2$ afforded (*R*)-**3jd** in 81% yield with >99% ee, and the absolute configuration of (*R*)-**3jd** was confirmed as *R* [Flack parameter = 0.068(9)] via the single crystal X-ray crystallography.

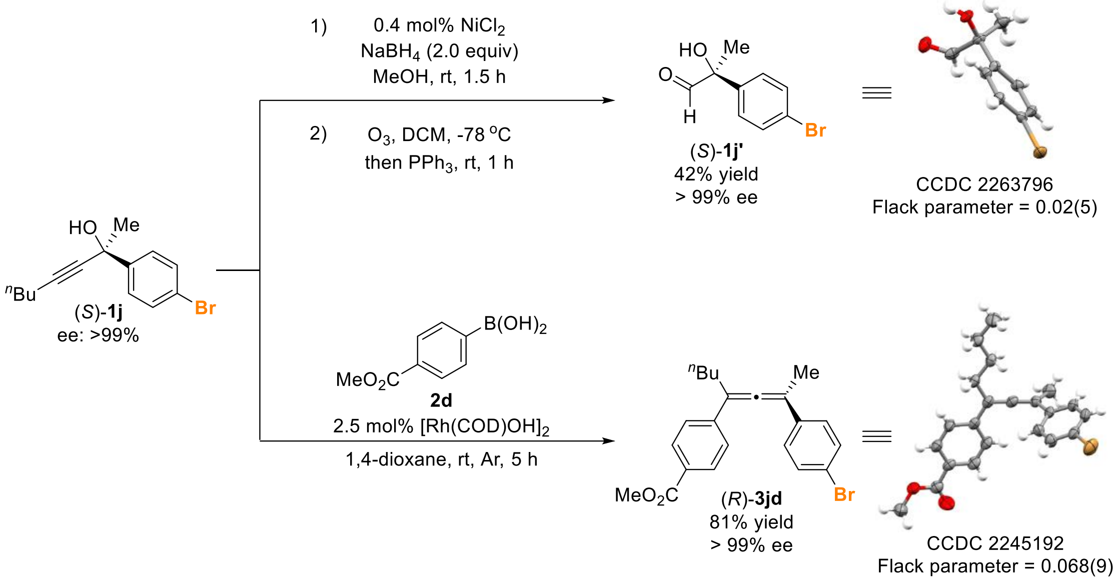

These results confirmed that the Rh-catalyzed coupling reaction of chiral tertiary propargylic alcohols with both aryl boronic acids and triphenylborixins proceeded an exclusive *anti-β*-OH elimination process rather than *syn-β*-OH elimination reported by us for C-H bond activation-based Rh-catalyzed direct reactions of optically active tertiary propargylic alcohols with *N*-methoxybenzamides.[10,11]

(1) Preparation of 2-(2-naphthyl)-4-phenyl-2,3-heptadiene (*rac*-3la) (zwf-8-074)

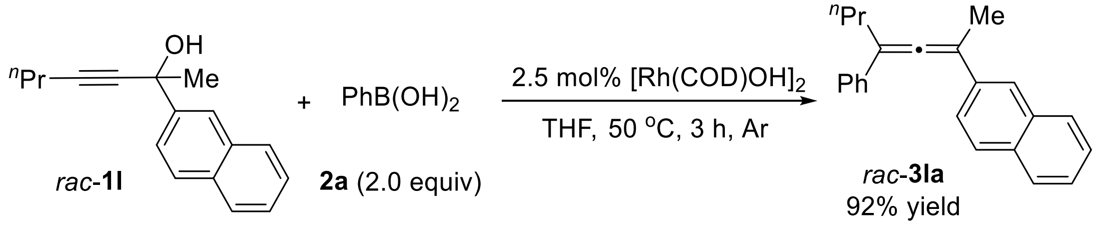

Following Typical Procedure I, the reaction of *rac*-**1l** (47.4 mg, 0.2 mmol), phenylboronic acid **2a** (48.1 mg, 0.4 mmol), $[Rh(COD)OH]_2$ (2.2 mg, 0.005 mmol) and THF (1 mL) afforded *rac*-**3la** (54.6 mg, 92%) [eluent: petroleum ether / ethyl acetate = 80/1 (~160 mL)]: oil; ^{1}**H NMR** (400 MHz, $CDCl_3$): δ = 7.85-7.73 (m, 3 H, Ar-H), 7.70 (d, J = 8.8 Hz, 1 H, Ar-H), 7.62 (dd, J_1 = 8.4 Hz, J_2 = 1.6 Hz, 1 H, Ar-H), 7.50-7.36 (m, 4 H, Ar-H), 7.31 (t, J = 7.6 Hz, 2 H, Ar-H), 7.24-7.16 (m, 1 H, Ar-H), 2.56 (t, J = 7.6 Hz, 2 H, CH_2), 2.32 (s, 3 H, CH_3), 1.71-1.56 (m, 2 H, CH_2), 1.01 (t, J = 7.4 Hz, 3 H, CH_3); ^{13}**C NMR** (100 MHz, $CDCl_3$): δ = 206.4, 137.0, 134.7, 133.7, 132.4, 128.5, 128.0,

127.7, 127.5, 126.8, 126.1, 125.6, 125.0, 123.3, 107.9, 104.0, 32.4, 21.2, 16.9, 14.1; **IR** (neat): v = 3055, 2957, 2927, 2867, 1931, 1598, 1497, 1452, 1099, 1064 cm^{-1}; **MS** (70 eV, EI) *m/z* (%): 299 (M^++1, 10.86), 298 (M^+, 47.91), 255 (100); **HRMS** calcd *m/z* for $C_{23}H_{22}$ [M^+]: 298.1716, found: 298.1716.

(2) Preparation of (*R*)-2-(2-naphthyl)-4-phenyl-2,3-heptadiene ((*R*)-3la) (zwf-8-077 and zwf-8-083)

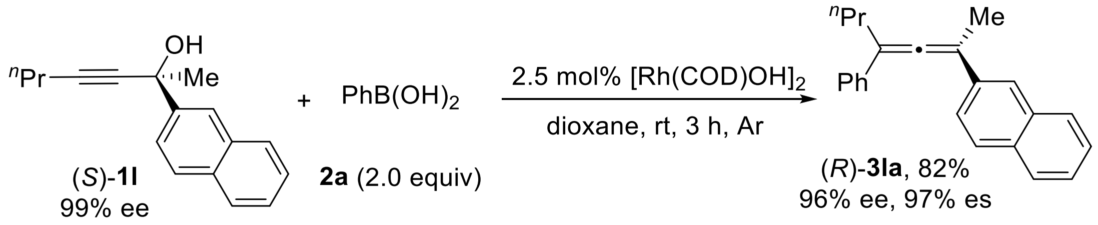

Following Typical Procedure II, the reaction of (*S*)-**1l** (47.7 mg, 0.2 mmol, 99% ee), phenylboronic acid **2a** (48.2 mg, 0.4 mmol), $[Rh(COD)OH]_2$ (2.2 mg, 0.005 mmol) and dioxane (1 mL) afforded (*R*)-**3la** (48.7 mg, 82%) [eluent: petroleum ether / ethyl acetate = 80/1 (~160 mL)]: oil; 96% ee (HPLC conditions: AD-H column, hexane/*i*-PrOH = 99.5/0.5, 0.5 mL/min, λ = 214 nm, t_R (major) = 10.2 min, t_R (minor) = 11.3 min); $[\alpha]_D^{28}$ = -339.3 (c = 1.00, EtOH); $[\alpha]_D^{26}$ = -362.5 (c = 1.00, $CHCl_3$); oil; **^{1}H NMR** (400 MHz, $CDCl_3$): δ = 7.89-7.73 (m, 3 H, Ar-H), 7.71 (d, J = 8.4 Hz, 1 H, Ar-H), 7.62 (d, J = 8.4 Hz, 1 H, Ar-H), 7.52-7.36 (m, 4 H, Ar-H), 7.31 (t, J = 7.4 Hz, 2 H, Ar-H), 7.24-7.16 (m, 1 H, Ar-H), 2.57 (t, J = 7.2 Hz, 2 H, CH_2), 2.32 (s, 3 H, CH_3), 1.73-1.53 (m, 2 H, CH_2), 1.01 (t, J = 7.2 Hz, 3 H, CH_3); **^{13}C NMR** (100 MHz, $CDCl_3$): δ = 206.4, 137.0, 134.7, 133.7, 132.4, 128.5, 128.0, 127.7, 127.5, 126.7, 126.1, 125.6, 125.0, 123.3, 107.9, 104.0, 32.4, 21.2, 16.9, 14.1; **IR** (neat): v = 3055, 2957, 2923, 2874, 1927, 1598, 1496, 1451, 1097, 1065 cm^{-1}; **MS** (70 eV, EI) *m/z* (%): 299 (M^++1, 11.75), 298 (M^+, 48.45), 255 (100); **HRMS** calcd *m/z* for $C_{23}H_{22}$ [M^+]: 298.1716, found: 298.1716.

(3) Preparation of (*R*)-2-(2-naphthyl)-4-phenyl-2,3-heptadiene ((*R*)-3la) (zwf-8-078 and zwf-8-084)

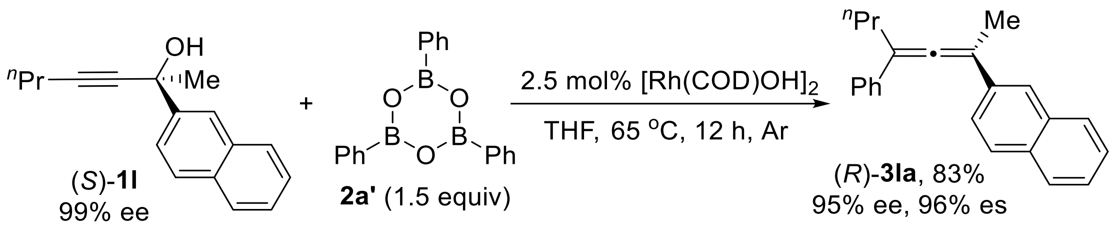

Typical Procedure V: To an oven-dried Schlenk tube (25 mL) was added boroxin **2a'** (31.1 mg, 0.1 mmol), which was then transferred to a glovebox. After adding $[Rh(COD)OH]_2$ (2.3 mg, 0.005 mmol) in glovebox, it was transferred out of the glovebox. After replacing nitrogen with argon for three times at rt by vacuum, (*S*)-**1l** (47.6 mg, 0.2 mmol, 99% ee), and freshly distilled THF (1 mL) were added. The resulting mixture was vigorously stirred at 65 °C for 12 h as monitored by TLC, diluted with ethyl acetate (1 mL), filtered through a short column of silica gel (1 cm), eluted with ethyl acetate (5 mL), and concentrated. The residue was purified by chromatography on silica gel to afford (*R*)-**3la** (49.5 mg, 83%) [eluent: petroleum ether / ethyl acetate = 80/1 (~160 mL)]: oil; 95% ee (HPLC conditions: AD-H column, hexane/*i*-PrOH = 99.5/0.5, 0.5 mL/min, λ = 214 nm, t_R (major) = 9.9 min, t_R (minor) = 10.8 min); $[\alpha]_D^{29}$ = -337.3 (*c* = 1.02, EtOH); $[\alpha]_D^{27}$ = -361.3 (*c* = 1.01, $CHCl_3$); oil; **^{1}H NMR** (400 MHz, $CDCl_3$): δ = 7.87-7.73 (m, 3 H, Ar-H), 7.71 (d, *J* = 8.8 Hz, 1 H, Ar-H), 7.62 (d, *J* = 8.8 Hz, 1 H, Ar-H), 7.49-7.37 (m, 4 H, Ar-H), 7.31 (t, *J* = 7.8 Hz, 2 H, Ar-H), 7.23-7.15 (m, 1 H, Ar-H), 2.57 (t, *J* = 7.4 Hz, 2 H, CH_2), 2.32 (s, 3 H, CH_3), 1.72-1.53 (m, 2 H, CH_2), 1.01 (t, *J* = 7.2 Hz, 3 H, CH_3); **^{13}C NMR** (100 MHz, $CDCl_3$): δ = 206.4, 137.0, 134.7, 133.7, 132.4, 128.5, 128.0, 127.7, 127.5, 126.7, 126.1, 125.6, 125.0, 123.3, 107.9, 104.0, 32.4, 21.2, 16.9, 14.1.

(4) Preparation of (*R*)-2-phenyl-4-(4-(methoxycarbonyl)phenyl)-2,3-octadiene ((*R*)-3ad) (zwf-8-071)

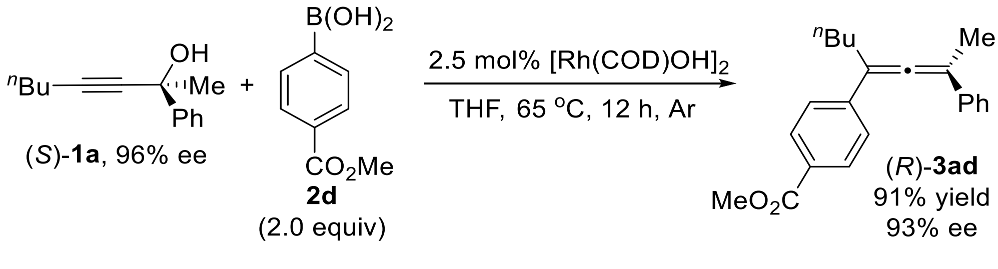

Following Typical Procedure V, (*S*)-**1a** (40.3 mg, 0.2 mmol, 96% ee), 4-(methoxycarbonyl)phenylboronic acid **2d** (71.8 mg, 0.4 mmol), $[Rh(COD)OH]_2$ (2.3 mg, 0.005 mmol) and THF (1 mL) afforded (*R*)-**3ad** (58.4 mg, 91%) [eluent: petroleum ether / ethyl acetate = 100/1 (~200 mL)]: 93% ee (HPLC conditions: OD-H column, hexane/*i*-PrOH = 99.5/0.5, 1.0 mL/min, λ = 214 nm, t_R (minor) = 6.1 min, t_R (major) = 9.2 min); $[\alpha]_D^{26}$ = -450.9 (*c* = 1.13, $CHCl_3$); **^{1}H NMR** (400 MHz, $CDCl_3$): δ = 7.96 (d, *J* = 8.4 Hz, 2 H, Ar-H), 7.53-7.38 (m, 4 H, Ar-H), 7.33 (t, *J* = 7.8 Hz, 2 H, Ar-H), 7.25-7.18 (m, 1 H, Ar-H), 3.89 (s, 3 H, OCH_3), 2.62-2.46 (m, 2 H, CH_2), 2.22 (s, 3 H, CH_3), 1.60-1.50 (m, 2 H, CH_2), 1.48-1.34 (m, 2 H, CH_2), 0.91 (t, *J* = 7.4 Hz, 3 H, CH_3); **^{13}C NMR** (100 MHz, $CDCl_3$): δ = 206.6, 167.0, 142.0, 136.6, 129.7, 128.5, 128.2, 126.9, 125.9, 125.7, 107.5, 104.2, 52.0, 30.0, 29.8, 22.5, 16.7, 13.9.

(5) Preparation of (*R*)-2-phenyl-4-(4-(methoxycarbonyl)phenyl)-2,3-octadiene ((*R*)-3ad) (zwf-8-072)

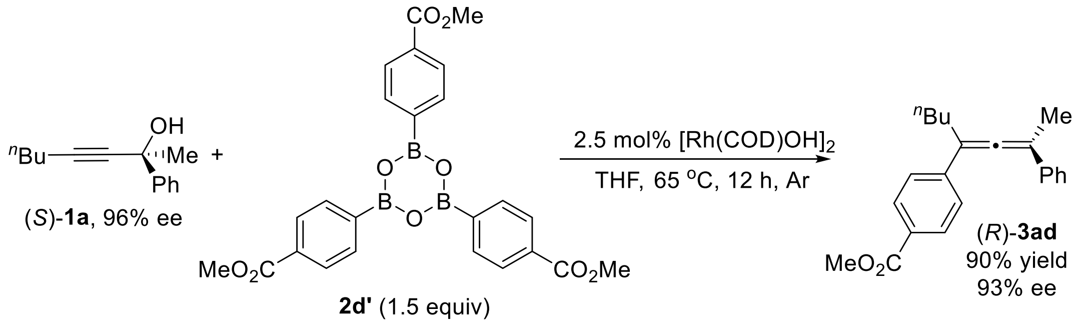

Following Typical Procedure V, (*S*)-**1a** (40.5 mg, 0.2 mmol, 96% ee), boroxin **2d'**[12] (48.8 mg, 0.1 mmol), $[Rh(COD)OH]_2$ (2.4 mg, 0.005 mmol) and THF (1 mL) afforded (*R*)-**3ad** (57.8 mg, 90%) [eluent: petroleum ether / ethyl acetate = 100/1 (~200 mL)]: 93% ee (HPLC conditions: OD-H column, hexane/*i*-PrOH = 99.5/0.5, 1.0 mL/min, λ = 214 nm, t_R (minor) = 5.9 min, t_R (major) = 8.9 min); $[\alpha]_D^{26}$ = -450.4 (*c* = 1.01, $CHCl_3$); **^{1}H NMR** (400 MHz, $CDCl_3$): δ = 7.96 (d, *J* = 8.8 Hz, 2 H, Ar-H), 7.53-7.37 (m, 4 H, Ar-H), 7.33 (t, *J* = 7.6 Hz, 2 H, Ar-H), 7.26-7.16 (m, 1 H, Ar-H), 3.89 (s, 3 H, OCH_3), 2.62-2.48 (m, 2 H, CH_2), 2.22 (s, 3 H, CH_3), 1.61-1.51 (m, 2 H, CH_2), 1.48-1.35 (m, 2 H, CH_2), 0.91 (t, *J* = 7.4 Hz, 3 H, CH_3); **^{13}C NMR** (100 MHz, $CDCl_3$): δ = 206.6, 167.0, 142.0, 136.6, 129.7, 128.5, 128.1, 126.9, 125.8, 125.7, 107.5, 104.2, 52.0, 30.0, 29.8,

22.6, 16.7, 13.9.

(6) Synthesis of 2-(4-bromophenyl)-4-(4-(methoxycarbonyl)phenyl)-2,3-octadiene (*rac*-3jd)(wj-7-137)

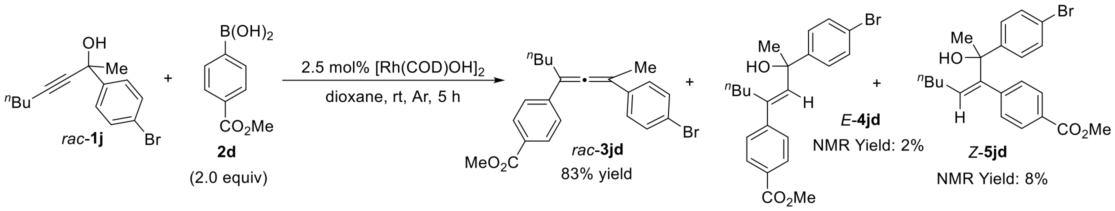

Following Typical Procedure II, the reaction of *rac*-**1j** (56.1 mg, 0.2 mmol), 4-(methoxycarbonyl)phenylboronic acid **2d** (72.1 mg, 0.4 mmol), [Rh(COD)OH]$_2$ (2.3 mg, 0.005 mmol), and dioxane (1 mL) afforded *rac*-**3jd** (65.8 mg, 83%) [eluent: petroleum ether / ethyl acetate = 150:1 (450 mL)]; solid; m.p. 89.8-90.7 ºC (DCM/MeOH); **^{1}H NMR** (400 MHz, $CDCl_3$): δ = 7.97 (d, *J* = 8.4 Hz, 2 H, Ar-H), 7.52-7.37 (m, 4 H, Ar-H), 7.27 (d, *J* = 8.4 Hz, 2 H, Ar-H), 3.90 (s, 3 H, CH_3), 2.55 (t, *J* = 7.4 Hz, 2 H, CH_2), 2.19 (s, 3 H, CH_3), 1.60-1.48 (m, 2 H, CH_2), 1.48-1.34 (m, 2 H, CH_2), 0.91 (t, *J* = 7.4 Hz, 3 H, CH_3); **^{13}C NMR** (100 MHz, $CDCl_3$): δ = 206.6, 166.9, 141.6, 135.7, 131.5, 129.8, 128.4, 127.2, 125.9, 120.8, 107.9, 103.5, 52.0, 30.0, 29.8, 22.5, 16.6, 13.9; **IR** (neat): *v* = 2924, 1929, 1719, 1604, 1488, 1435, 1271, 1175, 1101, 1080, 1009 cm^{-1}; **MS** (70 eV, EI) *m/z* (%): 400 [M$^+$(^{81}Br), 3.89], 398 [M$^+$(^{79}Br), 3.91], 277 (100); **HRMS** (EI) calcd for $C_{22}H_{23}{}^{79}BrO_2$ [M$^+$]: 398.0876, found: 398.0877.

(7) Synthesis of (*R*)-2-(4-bromophenyl)-4-(4-(methoxycarbonyl)phenyl)-2,3-octadiene ((*R*)-3jd)(wj-7-139)

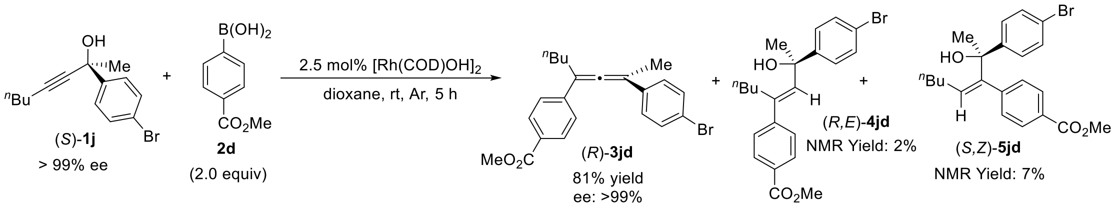

Following Typical Procedure II, the reaction of (*S*)-**1j** (84.1 mg, 0.3 mmol, >99% ee), 4-(methoxycarbonyl)phenylboronic acid **2d** (108.1 mg, 0.6 mmol), [Rh(COD)OH]$_2$ (3.4 mg, 0.0075 mmol), and dioxane (1.5 mL) afforded (*R*)-**3jd** (96.4 mg, 81%) [eluent:

petroleum ether / ethyl acetate = 150:1 (450 mL)]: >99% ee (HPLC conditions: AD-H column, hexane/*i*-PrOH = 98/2, 1.0 mL/min, λ = 214 nm, t_R (major) = 5.7 min); $[\alpha]_D^{30}$ = -498.0 (*c* = 0.93, $CHCl_3$); solid; m.p. 67.3-67.7 °C (DCM/MeOH); **^{1}H NMR** (400 MHz, $CDCl_3$): δ = 7.97 (d, *J* = 8.0 Hz, 2 H, Ar-H), 7.52-7.37 (m, 4 H, Ar-H), 7.28 (d, *J* = 8.4 Hz, 2 H, Ar-H), 3.90 (s, 3 H, CH_3), 2.55 (t, *J* = 7.4 Hz, 2 H, CH_2), 2.20 (s, 3 H, CH_3), 1.59-1.48 (m, 2 H, CH_2), 1.48-1.35 (m, 2 H, CH_2), 0.91 (t, *J* = 7.2 Hz, 3 H, CH_3); **^{13}C NMR** (100 MHz, $CDCl_3$): δ = 206.6, 166.9, 141.6, 135.7, 131.5, 129.8, 128.4, 127.2, 125.9, 120.8, 107.9, 103.5, 52.0, 30.0, 29.8, 22.5, 16.6, 13.9; **IR** (neat): *v* = 2951, 1931, 1720, 1603, 1482, 1434, 1273, 1179, 1100, 1073, 1006 cm^{-1}; **MS** (70 eV, EI) *m/z* (%): 400 [M^+(^{81}Br), 4.14], 398 [M^+(^{79}Br), 4.18], 277 (100); **HRMS** (EI) calcd for $C_{22}H_{23}{}^{79}BrO_2$ [M^+]: 398.0876, found: 398.0875.

(8) Synthesis of 2-(4-bromophenyl)-2-hydroxypropanal (*rac*-1j')(wj-8-036)

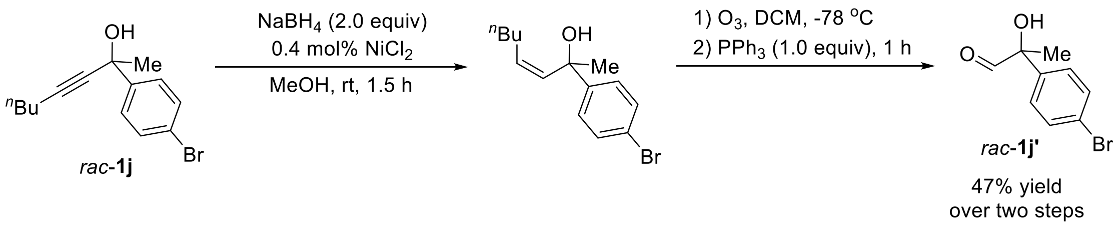

Step 1[13]: To an oven-dried Schlenk tube was added $NiCl_2$ (0.6 mg, 0.004 mmol). After replacing the air with argon for three times, *rac*-**1j** (281.4 mg, 1.0 mmol) and MeOH (8 mL) were added and the resulting mixture was vigorously stirred for 10 min at room temperature. Then $NaBH_4$ (75.7 mg, 2.0 mmol) was added and the resulting mixture was stirred at room temperature for 1.5 h as monitored by TLC, filtered through a short column of celite (3 cm) eluted with ethyl acetate (20 mL), concentrated, and directly used in the next step without further purification.

Step 2[7]: All the above prepared allyl alcohol was dissolved in DCM (40 mL) and the solution was cooled to -78 °C, then oxone was bubbled until the blue color persisted. Oxygen was then bubbled until the blue color disappeared, and triphenylphosphine (262.5 mg, 1.0 mmol) was added. The resulting mixture was stirred at room temperature for 1 h and concentrated. The resulting residue was purified by column chromatography on silica gel to afford *rac*-**1j'** (108.4 mg, 47%) [eluent: petroleum ether / ethyl acetate

= 8/1 (350 mL)]: solid; m.p. 51.7-52.8 °C (petroleum ether/DCM); **^{1}H NMR** (400 MHz, $CDCl_3$): δ = 9.52 (s, 1 H, CHO), 7.53 (d, *J* = 8.4 Hz, 2 H, Ar-H), 7.34 (d, *J* = 8.4 Hz, 2 H, Ar-H), 3.85 (s, 1 H, OH), 1.68 (s, 3 H, CH_3); **^{13}C NMR** (100 MHz, $CDCl_3$): δ = 199.2, 138.3, 132.0, 127.5, 122.5, 78.8, 23.7; **IR** (neat): *ν* = 3445, 2986, 1731, 1717, 1486, 1395, 1328, 1220, 1091, 1071, 1007 cm^{-1}; **MS** (ESI) *m/z*: 211 [M(^{79}Br)-H_2O+H^+]; **HRMS** calcd *m/z* for $C_9H_8O^{79}Br$ [M(^{79}Br)-H_2O+H^+]: 210.9753, found: 210.9754.

(9) Synthesis of (*S*)-2-(4-bromophenyl)-2-hydroxypropanal ((*S*)-1j') (wj-8-040)

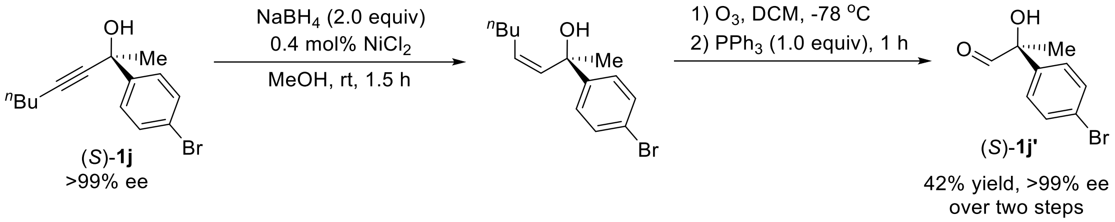

(*S*)-**1j**
>99% ee

(*S*)-**1j'**
42% yield, >99% ee
over two steps

Step 1[13]: To an oven-dried Schlenk tube was added $NiCl_2$ (0.6 mg, 0.004 mmol). After replacing the air with argon for three times, (*S*)-**1j** (280.8 mg, 1.0 mmol, >99% ee) and MeOH (8 mL) were added and the resulting mixture was vigorously stirred for 10 min at room temperature. Then $NaBH_4$ (76.0 mg, 2.0 mmol) was added and the resulting mixture was stirred at room temperature for 1.5 h as monitored by TLC, filtered through a short column of celite (3 cm) eluted with ethyl acetate (20 mL), concentrated, and directly used in the next step without further purification.

Step 2[7]: All the above prepared allyl alcohol was dissolved in DCM (40 mL) and the solution was cooled to -78 °C, then oxone was bubbled until the blue color persisted. Oxygen was then bubbled until the blue color disappeared, and triphenylphosphine (262.5 mg, 1.0 mmol) was added. The resulting mixture was stirred at room temperature for 1 h, concentrated, and purified by column chromatography on silica gel to afford (*S*)-**1j'** (95.0 mg, 42%) [eluent: petroleum ether / ethyl acetate = 8/1 (350 mL)]: >99% ee (HPLC conditions: OJ-H column, hexane/*i*-PrOH = 95/5, 1.0 mL/min, λ = 214 nm, t_R (major) = 16.1 min); $[\alpha]_D^{28}$ = +199.8 (*c* = 0.47, $CHCl_3$): solid; m.p. 78.8-79.3 °C (petroleum ether/DCM); **^{1}H NMR** (400 MHz, $CDCl_3$): δ = 9.53 (s, 1 H, CHO), 7.53 (d, *J* = 8.8 Hz, 2 H, Ar-H), 7.35 (d, *J* = 8.8 Hz, 2 H, Ar-H), 3.83 (s, 1 H, OH), 1.68 (s, 3 H, CH_3); **^{13}C NMR** (100 MHz, $CDCl_3$): δ = 199.2, 138.3, 132.0, 127.5, 122.5, 78.8, 23.7;

IR (neat): *ν* = 3445, 2986, 1731, 1717, 1486, 1395, 1328, 1220, 1091, 1071, 1007 cm^{-1}; **MS** (ESI) *m/z*: 211 [M(^{79}Br)-H_2O+H^+]; **HRMS** calcd *m/z* for $C_9H_8O^{79}Br$ [M(^{79}Br)-H_2O+H^+]: 210.9753, found: 210.9753.

1.7. Synthetic application

(1) Preparation of 6 (zwf-7-200)

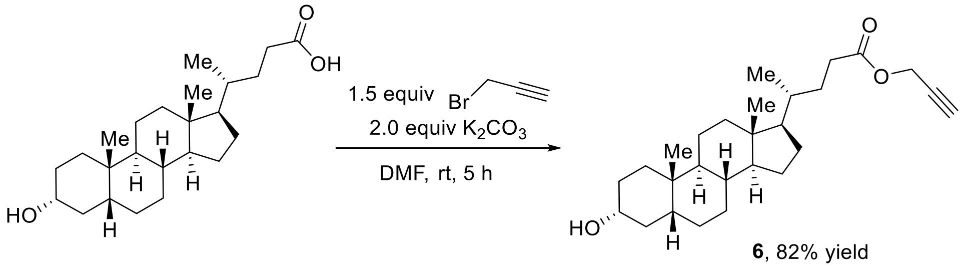

6, 82% yield

To a solution of lithocolic acid (754.1 mg, 2.0 mmol) and K_2CO_3 (553.3 mg, 4.0 mmol, 2.0 equiv) in DMF (10 ml) was added propargyl bromide (320 μl, d = 1.38 g/mL, 3.0 mmol, 1.5 equiv, 80% in toluene) at room temperature. After stirring at rt for 5 h, the reaction was quenched with H_2O (10 ml) and the resulting mixture was extracted with ethyl acetate (10 ml x 3). The organic layer was washed with brine (10 ml), dried over anhydrous Na_2SO_4, and concentrated in reduced pressure. The crude product was purified by chromatography on silica gel to afford **6** (680.7 mg, 82%) [eluent: petroleum ether / ethyl acetate = 5/1 (~540 mL)]: oil; **^{1}H NMR** (400 MHz, $CDCl_3$): δ = 4.67 (d, *J* = 2.4 Hz, 2 H, CH_2), 3.69-3.54 (m, 1 H, CH), 2.47 (t, *J* = 2.4 Hz, 1 H), 2.45-2.34 (m, 1 H), 2.32-2.21 (m, 1 H), 2.01-1.93 (m, 1 H), 1.90-1.47 (m, 9 H), 1.45-0.87 (m, 23 H), 0.64 (s, 3 H, CH_3); **^{13}C NMR** (100 MHz, $CDCl_3$): δ = 173.4, 77.8, 74.7, 71.7, 56.4, 55.9, 51.7, 42.7, 42.0, 40.4, 40.1, 36.4, 35.8, 35.3, 35.2, 34.5, 30.9, 30.8, 30.5, 28.1, 27.1, 26.4, 24.1, 23.3, 20.8, 18.2, 12.0; **IR** (neat): *ν* = 3309 (br), 2928, 2863, 2129, 1742, 1448, 1158, 1014 cm^{-1}; **MS** (ESI) *m/z*: 437 (M+Na^+), 397 (M-H_2O+H^+); **HRMS** calcd for $C_{27}H_{42}O_3Na$ [M+Na^+]: 437.3026, found: 437.3016.

(2) Preparation of 8 (zwf-7-182)

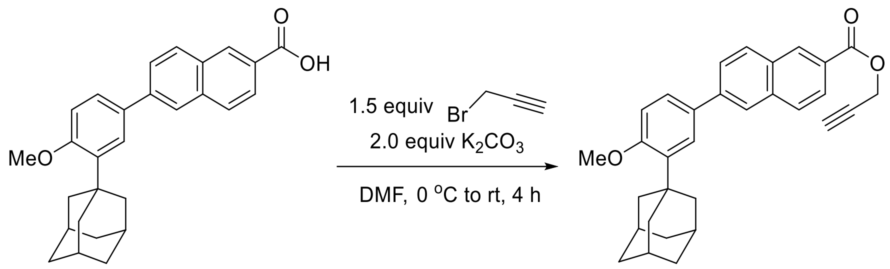

8, 84% yield

To a suspension of adapalene (1224.2 mg, 3.0 mmol) and K_2CO_3 (830.4 mg, 6.0 mmol, 2.0 equiv) in DMF (10 ml) was added propargyl bromide (480 μl, d = 1.38 g/mL, 4.5 mmol, 1.5 equiv, 80% in toluene) at 0 °C. After stirring at rt for 4 h, the reaction was quenched with H_2O (10 ml) and the resulting mixture was extracted with ethyl acetate (10 ml x 3). The organic layer was washed with brine (20 ml), dried over anhydrous Na_2SO_4, and concentrated in reduced pressure. The crude product was recrystallized by petroleum ether and DCM (v/v = 8 mL/8 mL) to afford **8** (1.1257 g, 84%): solid; m.p. 174.0-174.9 °C (petroleum ether/dichloromethane); **^{1}H NMR** (400 MHz, $CDCl_3$): δ = 8.63 (s, 1 H, Ar-H), 8.08 (dd, J_1 = 8.6 Hz, J_2 = 1.4 Hz, 1 H, Ar-H), 8.03-7.94 (m, 2 H, Ar-H), 7.91 (d, J = 8.8 Hz, 1 H, Ar-H), 7.79 (dd, J_1 = 8.4 Hz, J_2 = 1.6 Hz, 1 H, Ar-H), 7.60 (d, J = 2.4 Hz, 1 H, Ar-H), 7.53 (dd, J_1 = 8.4 Hz, J_2 = 2.0 Hz, 1 H, Ar-H), 6.98 (d, J = 8.4 Hz, 1 H, Ar-H), 4.99 (d, J = 2.8 Hz, 2 H, CH_2), 3.89 (s, 3 H, OCH_3), 2.55 (t, J = 2.2 Hz, 1 H, CH), 2.26-2.14 (m, 6 H, 3 x CH_2), 2.14-2.04 (m, 3 H, 3 x CH), 1.88-1.72 (m, 6 H, 3 x CH_2); **^{13}C NMR** (100 MHz, $CDCl_3$): δ = 166.0, 158.9, 141.6, 139.0, 136.1, 132.4, 131.2, 131.1, 129.7, 128.3, 126.5, 126.1, 125.9, 125.7, 125.5, 124.7, 112.1, 77.8, 75.0, 55.1, 52.5, 40.6, 37.2, 37.1, 29.1; **IR** (neat): v = 2903, 2843, 2361, 1709, 1448, 1218, 1089 cm^{-1}; **MS** (70 eV, EI) *m/z* (%): 451 ($M+1^+$, 33.38), 450 (M^+, 100.00); Anal. Calcd. for $C_{31}H_{30}O_3$: C 82.64, H 6.71; found C 82.34, H 6.61.

(3) Preparation of 9 (zwf-7-188)[14]

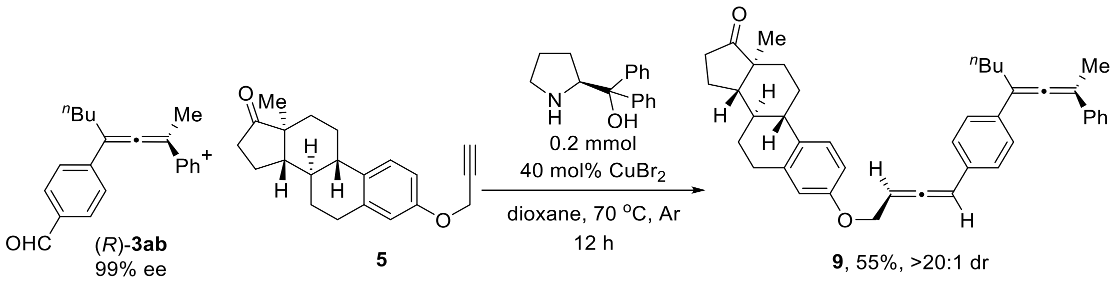

Typical Procedure VI: To an oven-dried Schlenk tube with a rubber plug were added $CuBr_2$ (18.1 mg, 0.08 mmol) and (*S*)-diphenylprolinol (50.7 mg, 0.2 mmol) under argon atmosphere. Then (*R*)-**3ab** (87.0 mg, 0.3 mmol, 99% ee)/dioxane (0.5 mL) and alkyne **5** (92.6 mg, 0.3 mmol)/dioxane (0.5 mL) were added sequentially. The reaction mixture was then stirred in an oil bath preheated at 70 °C for 12 h as monitored by TLC. The resulting mixture was diluted with ethyl acetate (1 mL), filtered through a short column of silica gel (1 cm), eluted with ethyl acetate (5 mL), and concentrated, which was purified by chromatography on silica gel to afford **9** (64.3 mg, 55%) [eluent: petroleum ether / ethyl acetate = 15/1 (~320 mL)]: oil; **^{1}H NMR** (400 MHz, $CDCl_3$): δ = 7.42 (d, J = 7.2 Hz, 2 H, Ar-H), 7.39-7.27 (m, 4 H, Ar-H), 7.23-7.14 (m, 4 H, Ar-H), 7.42 (dd, J_1 = 8.4 Hz, J_2 = 2.8 Hz, 1 H, Ar-H), 6.66 (d, J = 2.8 Hz, 1 H, Ar-H), 6.27 (dt, J_1 = 6.0 Hz, J_2 = 2.4 Hz, 1 H, =CH), 5.76 (q, J = 6.7 Hz, 1 H, =CH), 4.64 (dd, J_1 = 6.4 Hz, J_2 = 2.2 Hz, 2 H, CH_2), 2.95-2.70 (m, 2 H, CH_2), 2.60-2.42 (m, 3 H), 2.42-2.34 (m, 1 H), 2.29-1.88 (m, 8 H), 1.66-1.34 (m, 10 H), 0.95-0.83 (m, 6 H, 2 x CH_3); **^{13}C NMR** (100 MHz, $CDCl_3$): δ = 220.8, 206.5, 205.7, 156.1, 137.7, 137.1, 135.9, 132.3, 131.8, 128.4, 127.0, 126.7, 126.3, 126.2, 125.6, 115.0, 112.8, 107.6, 103.7, 95.9, 91.9, 65.7, 50.3, 47.9, 43.9, 38.3, 35.8, 31.5, 30.0, 29.9, 29.6, 26.5, 25.8, 22.6, 21.5, 16.8, 14.0, 13.8; **IR** (neat): v = 2927, 2859, 1951, 1737, 1607, 1494, 1229, 1024 cm^{-1}; **MS** (ESI) *m/z*: 583 ($M+H^+$); **HRMS** calcd for $C_{42}H_{47}O_2$ [$M+H^+$]: 583.3571, found: 583.3573.

(4) Preparation of 10 (zwf-8-001)[14]

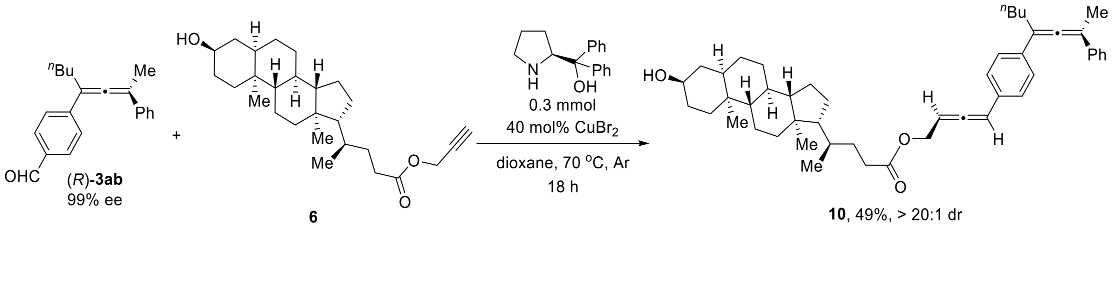

Following Typical Procedure VI, the reaction of (*S*)-diphenylprolinol (75.8 mg, 0.3 mmol), $CuBr_2$ (18.2 mg, 0.08 mmol), (*R*)-**3ab** (87.3 mg, 0.3 mmol, 99% ee), alkyne **6** (82.6 mg, 0.2 mmol), and dioxane (1 mL) afforded **10** (70.3 mg, 49%, 97% purity) [eluent: petroleum ether / ethyl acetate = 10/1 (~660 mL)]: oil; **^{1}H NMR** (400 MHz, $CDCl_3$): δ = 7.43 (d, J = 7.6 Hz, 2 H, Ar-H), 7.36 (d, J = 8.4 Hz, 2 H, Ar-H), 7.31 (t, J = 7.8 Hz, 2 H, Ar-H), 7.24-7.18 (m, 3 H, Ar-H), 6.27 (dt, J_1 = 6.4 Hz, J_2 = 2.6 Hz, 1 H, =CH), 5.69 (q, J = 6.4 Hz, 1 H, =CH), 4.73-4.47 (m, 2 H, CH_2), 3.71-3.53 (m, 1 H, CH), 2.53 (t, J = 7.4 Hz, 2 H, CH_2), 2.41-2.31 (m, 1 H), 2.28-2.16 (m, 4 H), 1.98-0.85 (m, 40 H), 0.62 (s, 3 H, CH_3); **^{13}C NMR** (100 MHz, $CDCl_3$): δ = 206.3, 205.8, 173.9, 137.1, 136.0, 131.7, 128.4, 127.0, 126.6, 126.3, 125.6, 107.6, 103.7, 96.4, 91.3, 71.8, 61.5, 56.4, 55.9, 42.7, 42.1, 40.4, 40.1, 36.4, 35.8, 35.31, 35.29, 34.5, 31.2, 30.9, 30.5, 30.1, 29.9, 28.1, 27.2, 26.4, 24.2, 23.3, 22.6, 20.8, 18.2, 16.8, 14.0, 12.0; **IR** (neat): ν = 3370 (br), 2927, 2862, 1952, 1736, 1446, 1264, 1160, 1015 cm^{-1}; **MS** (ESI) *m/z*: 690 ($M+H^+$); **HRMS** calcd for $C_{48}H_{65}O_3$ [$M+H^+$]: 689.4928, found: 689.4928.

(5) Preparation of 11 (zwf-7-189)[14]

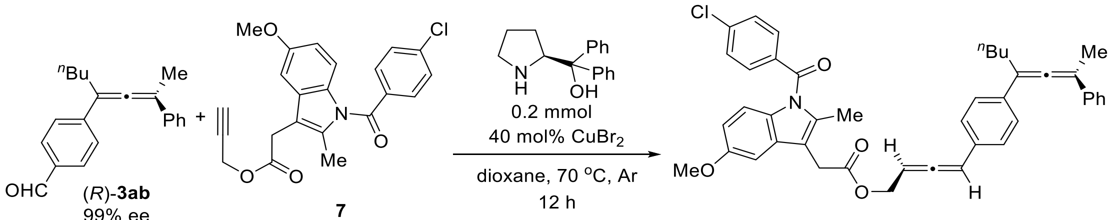

Following Typical Procedure VI, the reaction of (*S*)-diphenylprolinol (50.6 mg, 0.2 mmol), $CuBr_2$ (18.2 mg, 0.08 mmol), (*R*)-**3ab** (86.9 mg, 0.3 mmol, 99% ee), alkyne **7** (109.7 mg, 0.3 mmol), and dioxane (1 mL) afforded **11** (90.9 mg, 68%) [eluent: petroleum ether / ethyl acetate = 15/1 (~320 mL)]: oil; **^{1}H NMR** (400 MHz, $CDCl_3$): δ = 7.61 (d, J = 8.4 Hz, 2 H, Ar-H), 7.42 (d, J = 8.4 Hz, 4 H, Ar-H), 7.36-7.27 (m, 4 H, Ar-H), 7.24-7.10 (m, 3 H, Ar-H), 6.96 (d, J = 2.0 Hz, 1 H, Ar-H), 6.85 (d, J = 9.2 Hz, 1 H, Ar-H), 6.63 (dd, J_1 = 8.8 Hz, J_2 = 2.2 Hz, 1 H, Ar-H), 6.20 (dt, J_1 = 6.4 Hz, J_2 = 3.0 Hz, 1 H, =CH), 5.67 (q, J = 6.3 Hz, 1 H, =CH), 4.84-4.52 (m, 2 H, CH_2), 3.79 (s, 3 H, OCH_3), 3.67 (s, 2 H, CH_2), 2.51 (t, J = 7.4 Hz, 2 H, CH_2), 2.34 (s, 3 H, CH_3), 2.19 (s, 3 H, CH_3), 1.60-1.48 (m, 2 H, CH_2), 1.48-1.34 (m, 2 H, CH_2), 0.90 (t, J = 7.0 Hz, 3 H,

CH_3); **^{13}C NMR** (100 MHz, $CDCl_3$): δ = 206.4, 205.8, 170.4, 168.1, 156.0, 139.1, 137.1, 136.1, 135.9, 133.9, 131.5, 131.1, 130.7, 130.6, 129.0, 128.4, 126.9, 126.6, 126.3, 125.6, 114.9, 112.4, 111.6, 107.6, 103.7, 101.3, 96.5, 90.9, 62.2, 55.6, 30.3, 30.0, 29.8, 22.6, 16.8, 13.9, 13.3; **IR** (neat): *ν* = 2955, 2929, 2858, 1953, 1736, 1682, 1596, 1478, 1315, 1290, 1222, 1141,1066 cm^{-1}; **MS** (ESI) *m/z*: 670 [M(^{35}Cl)+H^+]; **HRMS** calcd for $C_{43}H_{41}{}^{35}ClNO_4$ [M+H^+]: 670.2719, found: 670.2721.

(6) Preparation of 12 (zwf-7-190)[14]

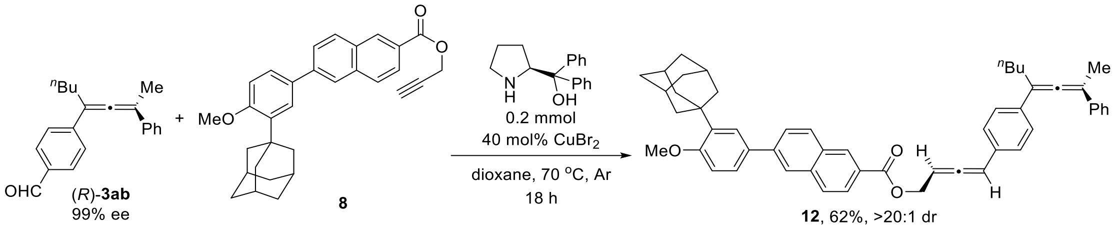

Following Typical Procedure VI, the reaction of (*S*)-diphenylprolinol (50.7 mg, 0.2 mmol), $CuBr_2$ (18.4 mg, 0.08 mmol), (*R*)-**3ab** (87.0 mg, 0.3 mmol, 99% ee), and alkyne **8** (135.4 mg, 0.3 mmol) in dioxane (1 mL) afforded **12** (89.7 mg, 62%) [eluent: petroleum ether / ethyl acetate = 80/1 (~400 mL)]: oil; **^{1}H NMR** (400 MHz, $CDCl_3$): δ = 8.59 (s, 1 H, Ar-H), 8.06 (dd, J_1 = 8.8 Hz, J_2 = 1.6 Hz, 1 H, Ar-H), 7.99 (s, 1 H, Ar-H), 7.90 (dd, J_1 = 14.0 Hz, J_2 = 8.0 Hz, 2 H, Ar-H), 7.76 (dd, J_1 = 8.8 Hz, J_2 = 1.8 Hz, 1 H, Ar-H), 7.59 (d, *J* = 2.0 Hz, 1 H, Ar-H), 7.53 (dd, J_1 = 8.0 Hz, J_2 = 2.2 Hz, 1 H, Ar-H), 7.47-7.40 (m, 2 H, Ar-H), 7.38 (d, *J* = 8.4 Hz, 2 H, Ar-H), 7.35-7.24 (m, 4 H, Ar-H), 7.19 (t, *J* = 7.4 Hz, 1 H, Ar-H), 6.98 (d, *J* = 8.4 Hz, 1 H, Ar-H), 6.34 (dt, J_1 = 6.4 Hz, J_2 = 2.4 Hz, 1 H, =CH), 5.89 (q, *J* = 6.5 Hz, 1 H, =CH), 5.90-4.85 (m, 2 H, CH_2), 3.89 (s, 3 H, OCH_3), 2.61-2.45 (m, 2 H), 2.27-2.13 (m, 9 H), 2.13-2.05 (m, 3 H), 1.88-1.70 (m, 6 H), 1.61-1.51 (m, 2 H, CH_2), 1.47-1.35 (m, 2 H, CH_2), 0.90 (t, *J* = 7.4 Hz, 3 H, CH_3); **^{13}C NMR** (100 MHz, $CDCl_3$): δ = 206.8, 205.8, 166.5, 158.9, 141.4, 139.0, 137.1, 136.1, 136.0, 132.5, 131.7, 131.2, 131.0, 129.7, 128.4, 128.2, 127.1, 126.8, 126.6, 126.41, 126.35, 125.9, 125.7, 125.61, 125.58, 124.7, 112.1, 107.6, 103.7, 96.5, 91.2, 62.5, 55.1, 40.6, 37.2, 37.1, 30.1, 29.9, 29.1, 22.6, 16.8, 14.0; **IR** (neat): *ν* = 2902, 2848, 1952, 1715, 1628, 1442, 1276, 1179, 1138, 1090 cm^{-1}; **MS** (ESI) *m/z*: 725 [M+H^+]; **HRMS** calcd *m/z* for $C_{52}H_{53}O_3$ [M+H^+]: 725.3989, found: 725.3991.

(7) Preparation of 2-phenyl-4-(4-(1-oxo-3-(trimethylsilyl)prop-2-ynylphenyl))-2,3-octadiene (*rac*-13) (zwf-8-009 and zwf-8-010)[15]

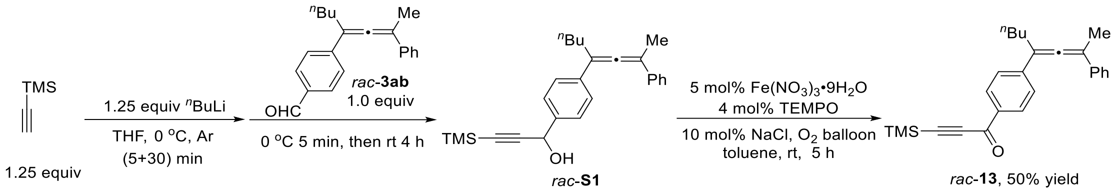

To an oven-dried Schlenk tube (25 mL) were added trimethylsilylacetylene (35 μL, d = 0.695 g/mL, 24.3 mg, 0.25 mmol) and THF (1 mL). Then a solution of ^{n}BuLi (0.1 mL, 2.5 M in hexane, 0.25 mmol) was added dropwise over 5 min at 0 °C under argon. After being stirred at 0 °C for 30 min, a solution of *rac*-**3ab** (58.0 mg, 0.2 mmol) in THF (1 mL) was added dropwise over 5 min. The resulting mixture then stirred at rt for 4 h as monitored by TLC and quenched with H_2O (2 mL). After extraction with ethyl acetate (3 mL x 3), the combined organic layer was washed with brine (5 mL) and dried over anhydrous Na_2SO_4. Filtration and concentration under reduced pressure afforded crude *rac*-**S1** as an oil, which was used in the next step without further purification.

To an oven-dried Schlenk tube (25 mL) containing the whole *rac*-**S1** prepared above were added toluene (1 mL), NaCl (1.0 mg, 0.02 mmol), $Fe(NO_3)_3 \cdot 9H_2O$ (4.6 mg, 0.01 mmol), and TEMPO (1.2 mg, 0.008 mmol). Then the flask was degassed and refilled with O_2 by a balloon of O_2 for three times. The resulting mixture was stirred under the atmosphere of O_2 from a balloon at rt until the reaction was complete as monitored by TLC. The residue was diluted with ethyl acetate (1 mL), filtered through a short column of silica gel (1 cm), eluted with ethyl acetate (5 mL), and concentrated, which was purified by chromatography on silica gel to afford *rac*-**13** (38.6 mg, 50%) [eluent: petroleum ether / ethyl acetate = 100/1 (~300 mL)]: oil; **^{1}H NMR** (400 MHz, $CDCl_3$): δ = 8.08 (d, *J* = 8.4 Hz, 2 H, Ar-H), 7.52 (d, *J* = 8.8 Hz, 2 H, Ar-H), 7.48-7.39 (m, 2 H, Ar-H), 7.34 (t, *J* = 7.6 Hz, 2 H, Ar-H), 7.27-7.21 (m, 1 H, Ar-H), 2.64-2.51 (m, 2 H, CH_2), 2.23 (s, 3 H, CH_3), 1.62-1.52 (m, 2 H, CH_2), 1.49-1.38 (m, 2 H, CH_2), 0.92 (t, *J* = 7.4 Hz, 3 H, CH_3), 0.30 (s, 9 H, 3 x CH_3); **^{13}C NMR** (100 MHz, $CDCl_3$): δ = 207.2, 177.0, 143.4, 136.3, 134.8, 129.9, 128.5, 127.0, 126.0, 125.7, 107.6, 104.4, 101.0, 100.1,

30.0, 29.8, 22.5, 16.6, 13.9, -0.7; **IR** (neat): v = 2957, 2927, 2859, 2153, 1930, 1641, 1596, 1252, 1010 cm^{-1}; **MS** (70 eV, EI) *m/z* (%): 387 (M^++1, 7.73), 386 (M^+, 22.88), 125 (100); **HRMS** calcd *m/z* for $C_{26}H_{30}OSi$ [M^+]: 386.2060, found: 386.2059.

(8) Preparation of (*R*)-2-phenyl-4-(4-(1-oxo-3-(trimethylsilyl)prop-2-ynylphenyl))-2,3-octadiene (*R*)-13 (zwf-8-020 and zwf-8-024)[15]

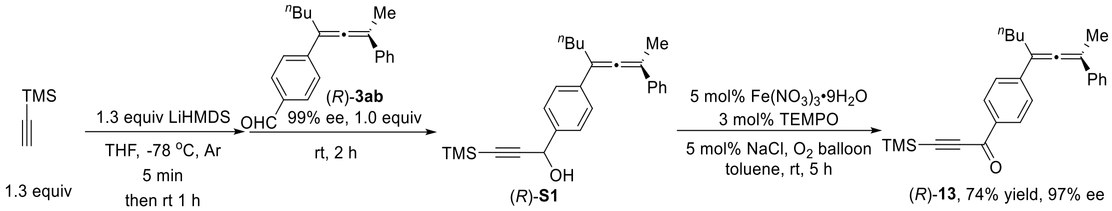

To an oven-dried Schlenk tube (25 mL) were added trimethylsilylacetylene (40 μL, d = 0.695 g/mL, 27.8 mg, 0.28 mmol) and THF (1 mL). Then a solution of LiHMDS (0.2 mL, 1.3 M in hexane, 0.26 mmol) was added dropwise over 5 min at -78 °C under argon. The reaction was warmed up to room temperature. After being stirred at rt for 1 h, a solution of (*R*)-**3ab** (58.2 mg, 0.2 mmol, 99% ee) in THF (1 mL) was added. The resulting mixture then stirred at rt for 2 h as monitored by TLC and quenched with H_2O (2 mL). After extraction with ethyl acetate (3 mL x 3), the combined organic layer was washed with brine (5 mL) and dried over anhydrous Na_2SO_4. Filtration and concentration under reduced pressure afforded crude (*R*)-**S1** as an oil, which was used in the next step without further purification.

To an oven-dried Schlenk tube (25 mL) containing the whole (*R*)-**S1** prepared above were added toluene (1 mL), NaCl (0.8 mg, 0.01 mmol), $Fe(NO_3)_3·9H_2O$ (4.0 mg, 0.01 mmol), and TEMPO (1.1 mg, 0.007 mmol). Then the flask was degassed and refilled with O_2 by a balloon of O_2 for three times. The resulting mixture was stirred under the atmosphere of O_2 from a balloon at rt until the reaction was complete as monitored by TLC. The residue was diluted with ethyl acetate (1 mL), filtered through a short column of silica gel (1 cm), eluted with ethyl acetate (5 mL), and concentrated, which was purified by chromatography on silica gel to afford (*R*)-**13** (57.4 mg, 74%) [eluent: petroleum ether / ethyl acetate = 100/1 (~300 mL)]: 97% ee (HPLC conditions: OD-H column, hexane/*i*-PrOH = 99.5/0.5, 1.0 mL/min, λ = 214 nm, t_R (minor) = 5.2 min, t_R

(major) = 9.4 min); oil; **^{1}H NMR** (400 MHz, $CDCl_3$): δ = 8.08 (d, J = 8.4 Hz, 2 H, Ar-H), 7.53 (d, J = 8.4 Hz, 2 H, Ar-H), 7.43 (d, J = 7.2 Hz, 2 H, Ar-H), 7.34 (t, J = 7.8 Hz, 2 H, Ar-H), 7.26-7.20 (m, 1 H, Ar-H), 2.63-2.51 (m, 2 H, CH_2), 2.23 (s, 3 H, CH_3), 1.63-1.53 (m, 2 H, CH_2), 1.50-1.38 (m, 2 H, CH_2), 0.92 (t, J = 7.2 Hz, 3 H, CH_3), 0.31 (s, 9 H, 3 x CH_3); **^{13}C NMR** (100 MHz, $CDCl_3$): δ = 207.3, 177.1, 143.4, 136.4, 134.8, 129.9, 128.5, 127.0, 126.0, 125.7, 107.6, 104.4, 101.0, 100.1, 30.0, 29.8, 22.5, 16.6, 13.9, -0.7; **IR** (neat): v = 2957, 2928, 2859, 2153, 1930, 1641, 1596, 1252, 1010 cm^{-1}; **MS** (70 eV, EI) *m/z* (%): 387 (M^++1, 7.81), 386 (M^+, 22.71), 125 (100); **HRMS** calcd *m/z* for $C_{26}H_{30}OSi$ [M^+]: 386.2060, found: 386.2064.

(9) Preparation of 2-phenyl-4-(4-hydroxymethylphenyl)-2,3-octadiene (*rac*-14) (zwf-8-014)

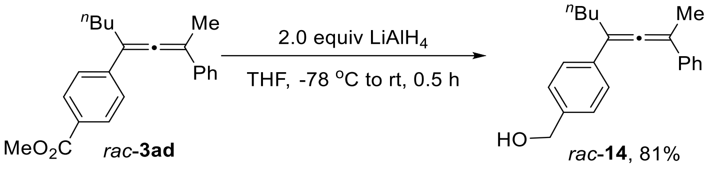

Typical Procedure VII: To an oven-dried Schlenk tube (25 mL) were added $LiAlH_4$ (15.0 mg, 0.4 mmol) and THF (0.5 mL) sequentially. The reaction mixture was cooled down to -78 °C and a solution of *rac*-**3ad** (64.2 mg, 0.2 mmol) in THF (0.5 mL) was added to the tube. The resulting mixture then stirred at rt for 0.5 h as monitored by TLC and quenched with H_2O (2 mL) at -78 °C. After extraction with ethyl acetate (5 mL x 3), the combined organic layer was washed with brine (10 mL), dried over anhydrous Na_2SO_4, and concentrated in reduced pressure. The crude product was purified by chromatography on silica gel to afford *rac*-**14** (47.4 mg, 81%) [eluent: petroleum ether / ethyl acetate = 5/1 (~360 mL)]: oil; **^{1}H NMR** (400 MHz, $CDCl_3$): δ = 7.50-7.37 (m, 4 H, Ar-H), 7.37-7.27 (m, 4 H, Ar-H), 7.24-7.17 (m, 1 H, Ar-H), 4.66 (s, 2 H, CH_2), 2.63-2.45 (m, 2 H, CH_2), 2.21 (s, 3 H, CH_3), 1.70-1.51 (m, 3 H, OH and CH_2), 1.48-1.35 (m, 2 H, CH_2), 0.91 (t, J = 7.4 Hz, 3 H, CH_3); **^{13}C NMR** (100 MHz, $CDCl_3$): δ = 205.6, 139.3, 137.1, 136.5, 128.4, 127.2, 126.6, 126.2, 125.6, 107.5, 103.6, 65.1, 30.1, 30.0, 22.6, 16.8, 14.0; **IR** (neat): v = 3334 (br), 2955, 2926, 2859, 1932, 1510, 1492, 1026,

1014 cm^{-1}; **MS** (70 eV, EI) *m/z* (%): 292 (M$^+$, 3.22), 205 (100); **HRMS** calcd *m/z* for $C_{21}H_{24}O$ [M$^+$]: 292.1822, found: 292.1827.

(10) Preparation of (*R*)-2-phenyl-4-(4-hydroxymethylphenyl)-2,3-octadiene (*R*)-14 (zwf-8-016)

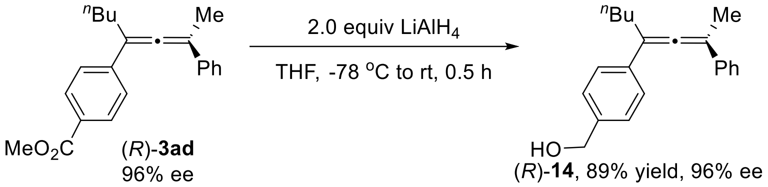

Following Typical Procedure VII, the reaction of $LiAlH_4$ (15.3 mg, 0.4 mmol) and (*R*)-**3ad** (64.0 mg, 0.2 mmol, 96% ee) in THF (1.0 mL) afforded (*R*)-**14** (52.2 mg, 89%) [eluent: petroleum ether / ethyl acetate = 10/1 (~330 mL)]: 96% ee (HPLC conditions: OD-H column, hexane/*i*-PrOH = 98/2, 1.0 mL/min, λ = 214 nm, t_R (minor) = 15.6 min, t_R (major) = 18.0 min); oil; **^{1}H NMR** (400 MHz, $CDCl_3$): δ = 7.51-7.37 (m, 4 H, Ar-H), 7.37-7.25 (m, 4 H, Ar-H), 7.23-7.16 (m, 1 H, Ar-H), 4.65 (s, 2 H, CH_2), 2.63-2.47 (m, 2 H, CH_2), 2.20 (s, 3 H, CH_3), 1.71 (brs, 1 H, OH), 1.61-1.50 (m, 2 H, CH_2), 1.48-1.35 (m, 2 H, CH_2), 0.91 (t, J = 7.4 Hz, 3 H, CH_3); **^{13}C NMR** (100 MHz, $CDCl_3$): δ = 205.6, 139.3, 137.2, 136.5, 128.4, 127.2, 126.7, 126.2, 125.6, 107.5, 103.6, 65.1, 30.1, 30.0, 22.6, 16.8, 14.0; **IR** (neat): *v* = 3367 (br), 2957, 2928, 2858, 1928, 1510, 1493, 1027, 1015 cm^{-1}; **MS** (70 eV, EI) *m/z* (%): 292 (M$^+$, 4.05), 205 (100); **HRMS** calcd *m/z* for $C_{21}H_{24}O$ [M$^+$]: 292.1822, found: 292.1822.

(11) Preparation of 2-phenyl-4-((4-formaylphenyl)phenyl)-2,3-octadiene (*rac*-15) (zwf-8-012)[16]

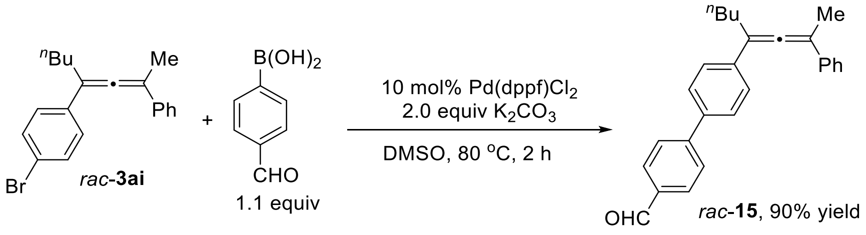

Typical Procedure VIII: To an oven-dried Schlenk tube (25 mL) were added $Pd(dppf)Cl_2$ (14.7 mg, 0.02 mmol), 4-formylboronic acid (33.2 mg, 0.22 mmol), and K_2CO_3 (55.8 mg, 0.4 mmol) sequentially. Then the flask was degassed and refilled with

argon. A solution of *rac*-**3ai** (68.4 mg, 0.2 mmol) in DMSO (2 mL) was added under argon. The resulting mixture was stirred at 80 °C under argon for 2 h, cooled down to the room temperature, and quenched with H_2O (2 mL). After extraction with ethyl acetate (5 mL x 3), the combined organic layer was washed with brine (10 mL), dried over anhydrous Na_2SO_4, and concentrated in reduced pressure. The crude product was purified by chromatography on silica gel to afford *rac*-**15** (65.8 mg, 90%) [eluent: petroleum ether / ethyl acetate = 10/1 (~330 mL)]: oil; **^{1}H NMR** (400 MHz, $CDCl_3$): δ = 10.02 (s, 1 H, CHO), 7.92 (d, *J* = 8.0 Hz, 2 H, Ar-H), 7.73 (d, *J* = 8.0 Hz, 2 H, Ar-H), 7.58 (d, *J* = 8.4 Hz, 2 H, Ar-H), 7.53 (d, *J* = 8.0 Hz, 2 H, Ar-H), 7.46 (d, *J* = 8.0 Hz, 2 H, Ar-H), 7.33 (t, *J* = 7.6 Hz, 2 H, Ar-H), 7.26-7.18 (m, 1 H, Ar-H), 2.65-2.52 (m, 2 H, CH_2), 2.23 (s, 3 H, CH_3), 1.66-1.53 (m, 2 H, CH_2), 1.50-1.38 (m, 2 H, CH_2), 0.93 (t, *J* = 7.2 Hz, 3 H, CH_3); **^{13}C NMR** (100 MHz, $CDCl_3$): δ = 206.1, 191.8, 146.7, 137.8, 137.4, 136.9, 135.0, 130.3, 128.4, 127.33, 127.32, 126.8, 126.6, 125.6, 107.4, 104.0, 30.1, 29.9, 22.6, 16.8, 14.0; **IR** (neat): *v* = 3028, 2955, 2927, 2858, 1930, 1701, 1603, 1463, 1169 cm^{-1}; **MS** (70 eV, EI) *m/z* (%): 367 (M^++1, 5.25), 366 (M^+, 18.22), 309 (100); **HRMS** calcd *m/z* for $C_{27}H_{26}O$ [M^+]: 366.1978, found: 366.1978.

(12) Preparation of (*R*)-2-phenyl-4-((4-formaylphenyl)-phenyl)-2,3-octadiene (*R*)-15 (zwf-8-015)[16]

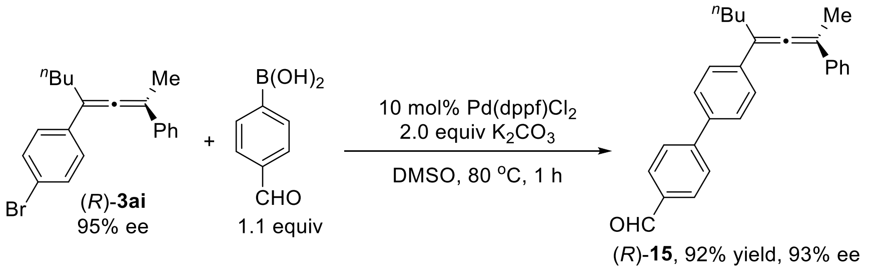

Following Typical Procedure VIII, the reaction of (*R*)-**3ai** (67.8 mg, 0.2 mmol, 95% ee), $Pd(dppf)Cl_2$ (14.7 mg, 0.02 mmol), 4-formylboronic acid (33.1 mg, 0.22 mmol), and K_2CO_3 (55.6 mg, 0.4 mmol), and DMSO (2 mL) afforded (*R*)-**15** (66.7 mg, 92%) [eluent: petroleum ether / ethyl acetate = 15/1 (~320 mL)]: 93% ee (HPLC conditions: AD-3 (3.0 mm I.D. *150mm) column, CO_2/*i*-PrOH = 90/10, 1.0 mL/min, λ = 254 nm, t_R (minor) = 13.8 min, t_R (major) = 16.5 min); oil; **^{1}H NMR** (400 MHz, $CDCl_3$): δ = 10.02

(s, 1 H, CHO), 7.92 (d, J = 8.0 Hz, 2 H, Ar-H), 7.73 (d, J = 8.0 Hz, 2 H, Ar-H), 7.58 (d, J = 8.4 Hz, 2 H, Ar-H), 7.53 (d, J = 8.0 Hz, 2 H, Ar-H), 7.46 (d, J = 8.0 Hz, 2 H, Ar-H), 7.33 (t, J = 7.6 Hz, 2 H, Ar-H), 7.22 (t, J = 7.2 Hz, 1 H, Ar-H), 2.58 (t, J = 7.6 Hz, 2 H, CH_2), 2.24 (s, 3 H, CH_3), 1.65-1.52 (m, 2 H, CH_2), 1.51-1.37 (m, 2 H, CH_2), 0.93 (t, J = 7.2 Hz, 3 H, CH_3); **^{13}C NMR** (100 MHz, $CDCl_3$): δ = 206.0, 191.8, 146.7, 137.8, 137.4, 136.9, 135.0, 130.3, 128.4, 127.33, 127.31, 126.8, 126.6, 125.6, 107.4, 104.0, 30.1, 29.9, 22.6, 16.8, 14.0; **IR** (neat): v = 3028, 2955, 2927, 2858, 1929, 1699, 1602, 1463, 1169 cm^{-1}; **MS** (70 eV, EI) m/z (%): 367 (M^++1, 6.09), 366 (M^+, 19.02), 309 (100); **HRMS** calcd m/z for $C_{27}H_{26}O$ [M^+]: 366.1978, found: 366.1978.

(13) 1 mmol scale preparation of (*R*)-2,4-diphenyl-2,3-octadiene ((*R*)-3aa) (zwf-7-049)

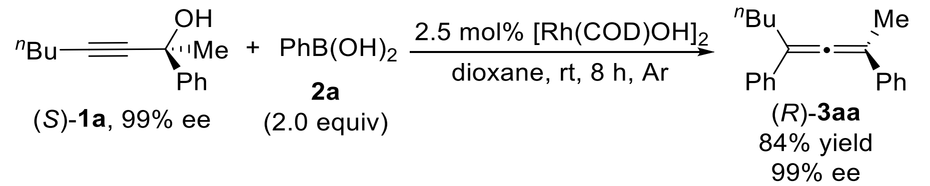

To an oven-dried Schlenk tube (25 mL) was added phenylboronic acid **2a** (243.7 mg, 2.0 mmol), which was then transferred to a glovebox. After adding $[Rh(COD)OH]_2$ (11.7 mg, 0.025 mmol) in glovebox, it was transferred out of the glovebox. After replacing nitrogen with argon for three times by vacuum, (*S*)-**1a** (201.4 mg, 1.0 mmol, 99% ee) and freshly distilled dioxane (5 mL) were added. The resulting mixture was vigorously stirred at room temperature for 8 h as monitored by TLC. The residue was diluted with ethyl acetate (5 mL), filtered through a short column of silica gel (3 cm), eluted with ethyl acetate (20 mL), and concentrated, which was purified by chromatography on silica gel to afford (*R*)-**3aa** (219.3 mg, 84%) [eluent: petroleum ether / ethyl acetate = 100/1 (~200 mL)]: 99% ee (HPLC conditions: OJ-H column, hexane/*i*-PrOH = 200/1, 0.7 mL/min, λ = 214 nm, t_R (major) = 6.3 min, t_R (minor) = 7.9 min); oil; **^{1}H NMR** (400 MHz, $CDCl_3$): δ = 7.43 (t, J = 7.6 Hz, 4 H, Ar-H), 7.36-7.26 (m, 4 H, Ar-H), 7.23-7.16 (m, 2 H, Ar-H), 2.61-2.47 (m, 2 H, CH_2), 2.20 (s, 3 H, CH_3), 1.62-1.54 (m, 2 H, CH_2), 1.48-1.36 (m, 2 H, CH_2), 0.91 (t, J = 7.2 Hz, 3 H, CH_3); **^{13}C**

NMR (100 MHz, $CDCl_3$): δ = 205.6, 137.2, 137.0, 128.40, 128.37, 126.7, 126.6, 126.1, 125.6, 107.8, 103.6, 30.1, 30.0, 22.6, 16.8, 14.0.

(14) Gram-scale synthesis of (*R*)-2-phenyl-4-(4-formylphenyl)-2,3-octadiene ((*R*)-3ak) (zwf-8-026)

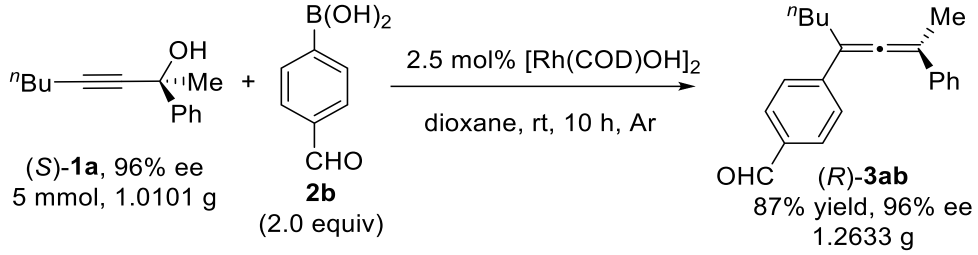

To an oven-dried Schlenk flask (100 mL) was added 4-formylphenylboronic acid **2c** (1.4913 g, 10 mmol), which was then transferred to a glovebox. After adding $[Rh(COD)OH]_2$ (57.3 mg, 0.125 mmol) in glovebox, it was transferred out of the glovebox. After replacing nitrogen with argon for three times by vacuum, (*S*)-**1a** (1.0101 g, 5.0 mmol, 96% ee) and freshly distilled dioxane (25 mL) were added. The resulting mixture was vigorously stirred at room temperature for 10 h as monitored by TLC. The residue was diluted with ethyl acetate (10 mL), filtered through a short column of silica gel (3 cm), eluted with ethyl acetate (50 mL), and concentrated, which was purified by chromatography on silica gel to afford (*R*)-**3ab** (1.2633 g, 87%) [eluent: petroleum ether / ethyl acetate = 100/1 (~500 mL)]: 96% ee (HPLC conditions: OD-H column, hexane/*i*-PrOH = 99.5/0.5, 1.0 mL/min, λ = 214 nm, t_R (major) = 10.2 min, t_R (minor) = 17.3 min); oil; **^{1}H NMR** (400 MHz, $CDCl_3$): δ = 9.97 (s, 1 H, CHO), 7.81 (d, J = 8.4 Hz, 2 H, Ar-H), 7.57 (d, J = 8.4 Hz, 2 H, Ar-H), 7.43 (d, J = 7.6 Hz, 2 H, Ar-H), 7.34 (t, J = 7.8 Hz, 2 H, Ar-H), 7.27-7.20 (m, 1 H, Ar-H), 2.64-2.50 (m, 2 H, CH_2), 2.24 (s, 3 H, CH_3), 1.64-1.52 (m, 2 H, CH_2), 1.50-1.35 (m, 2 H, CH_2), 0.92 (t, J = 7.2 Hz, 3 H, CH_3); **^{13}C NMR** (100 MHz, $CDCl_3$): δ = 207.2, 191.7, 143.8, 136.4, 134.7, 129.9, 128.5, 127.1, 126.4, 125.7, 107.6, 104.5, 30.0, 29.8, 22.5, 16.7, 13.9.

2. Supplementary DFT calculational details

Computational method.

All calculations were performed with the Gaussian 09 program.[17] Geometries have been fully optimized with the density functional theory of B3LYP method,[18] including Grimme's D3 dispersion corrections (B3LYP-D3 with Becke-Johnson damping).[19] The LANL2DZ[20] basis set in conjunction with the LANL2DZ pseudopotential was used for Rh atom, while the 6-31G(d,p)[21] basis set was used for carbon, hydrogen, nitrogen, oxygen and boron atoms. Harmonic vibration frequency calculations were conducted at the same level of theory to verify the stationary points to be minima (no imaginary frequency) or saddle points (one imaginary frequency). Intrinsic reaction coordinate (IRC)[22] calculations were performed to confirm the connection of the transition structures with their corresponding reactants and products. The solvent effects were determined by single-point calculations of the gas-phase stationary points at M06[23]/SDD[24]-6-311++G(d,p) level by using IEFPCM[25] solvation model. The reported energies are the solution-phase Gibbs free energies (ΔG_{sol}) in 1,4-dioxane (ε = 2.37) or in methanol (ε = 32.61) according to the experimental conditions.

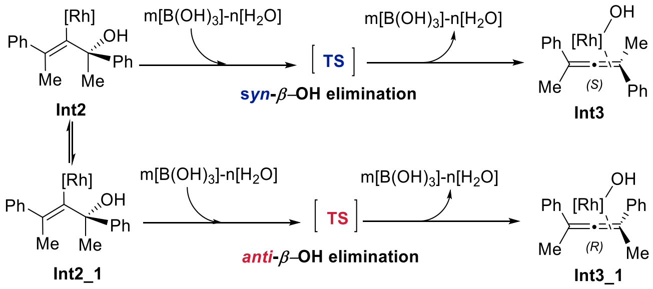

Supplementary Figure 4. The transition states for boric acid or/and water assisted Rh(I)-β-OH elimination of Int2

The transition states for Rh(I)-β-OH elimination of **Int2** (Fig. 4a and Supplementary Figure 4) assisted by boric acid or/and water are illlustrated in Supplementary Figure 5 (See Supplementary Figure 6 for the 3D-structures of these TSs). When one molecule of boric acid [1B(OH)3] is taken into consideration, a concerted six-membered cyclic transition structure **TS2_a** is obtained for β-OH elimination, which requires an energy

barrier of 29.2 kcal/mol. Similarly, **TS2_b** is located as the transition structure for one molecule of water [1H_2O] assisted process, with an energy barrier of 31.8 kcal/mol. However, both transition structures (**TS2_a** and **TS_b**) still proceed *syn*-elimination and feature even higher free energy barriers than *syn*-**TS2** (19.5 kcal/mol, Fig. 4a). Then two molecules of boric acid [2$B(OH)_3$] or water [2H_2O] are proposed as the hydroxy shuttle to assist the *β*-OH elimination step. Different from the one molecule-assisted process, the *β*-OH elimination with the help of [2$B(OH)_3$] or [2H_2O] could be not only the *syn*-process (**TS2_c**/**TS2_d**, Supplementary Figure 5) but also the *anti*-process (**TS2_o/TS2_p**, Supplementary Figure 5). Although these four eight-membered cyclic TSs are still unfavorable as compared with *syn*-**TS2**, the TSs for *anti*-process **TS2_o**/**TS2_p** are more stable than their corresponding *syn*-TSs (**TS2_c**/**TS2_d**). Furthermore, when three molecules of boric acid [3$B(OH)_3$] or water [3H_2O] participated *β*-OH elimination *via* ten-membered cyclic TSs are considered**,** no transition structures are obtained for [3$B(OH)_3$] or [3H_2O] participated *anti*-process. The ten-membered *syn*-TSs (**TS2_g**/**TS2_h**, Supplementary Figure 5) feature much higher free energies than *syn*-**TS2** (19.5 kcal/mol, Fig. 4a). Thus, neither boric acid nor water could facilitate the *β*-OH elimination process alone.

The possibility of the simultaneous participation of boric acid and water is also taken into consideration. Two *syn*-TSs (**TS2_e**/**TS2_f**, Supplementary Figure 5) and two *anti*-TSs (**TS2_q/TS2_r**, Supplementary Figure 5) are obtained for *β*-OH elimination assisted by one molecule of boric acid with one molecule of water [1$B(OH)_3$]-[1H_2O]. However, these four eight-membered cyclic TSs are still less stable than *syn*-**TS2** (19.5 kcal/mol, Fig. 4a).

Subsequently, the ten-membered cyclic TSs with simultaneous participation of two molecules of boric acid with one molecule of water [2$B(OH)_3$]-[1H_2O] or one molecule of boric acid with two molecules of water [1$B(OH)_3$]-[2H_2O] are investigated. Six *syn*-TSs (**TS2_i-n**, Supplementary Figure 5) and six *anti*-TSs (**TS2_u-z**, Supplementary Figure 5) associated with different rankings of the assisting species, are obtained for *β*-OH elimination. The increase of one more assisting molecule of $B(OH)_3$ or H_2O further increases the stability of ten-membered ring TSs (except **TS2_j/k/z**), making them

more kinetically favorable than the eight-membered ring TSs. Notably, the energetic data shows an obvious preference of *anti-β*-OH elimination over the *syn*-process.

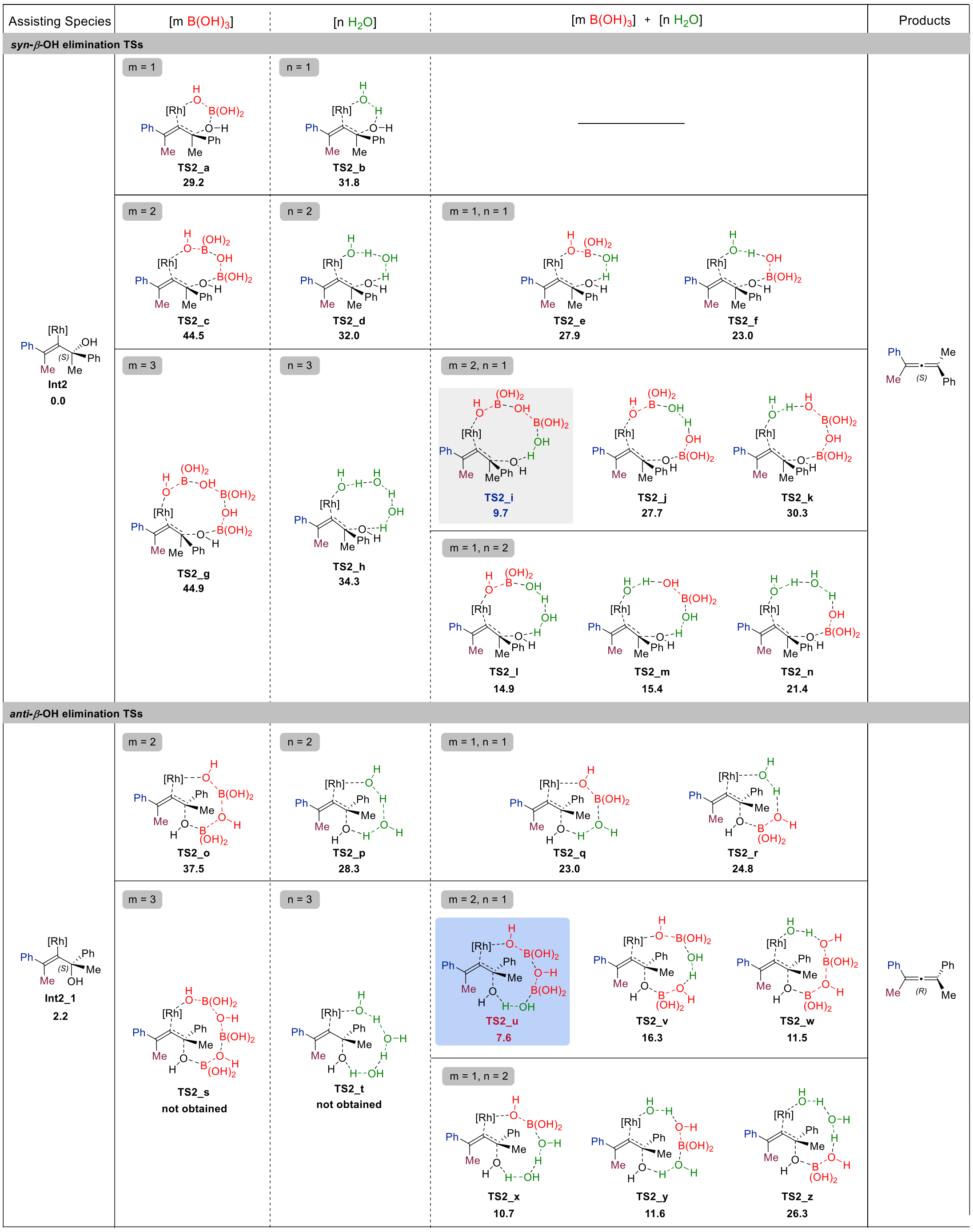

Supplementary Figure 5. The transition states and their corresponding activation energies for boric acid or/and water assisted Rh(I)-*β*-OH elimination of **Int2**. (Free energies are given in kcal/mol.)

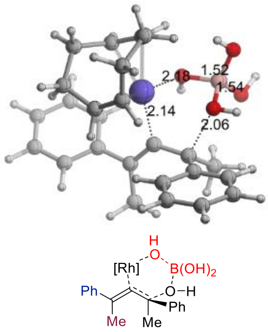

TS2_a

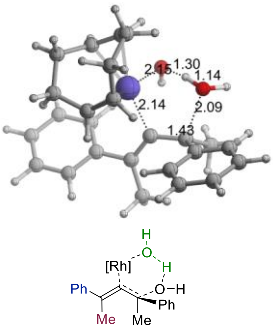

TS2_b

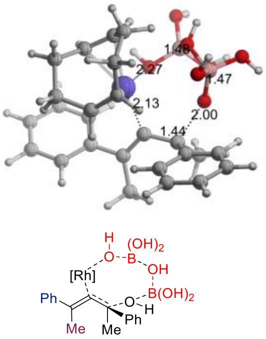

TS2_c

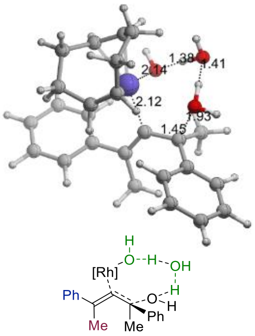

TS2_d

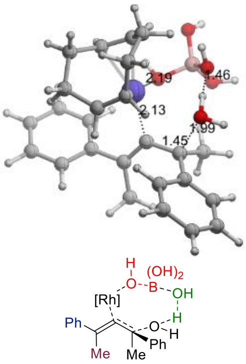

TS2_e

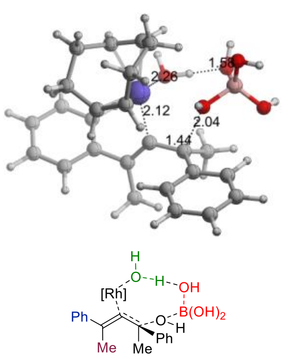

TS2_f

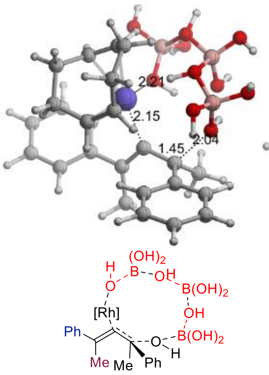

TS2_g

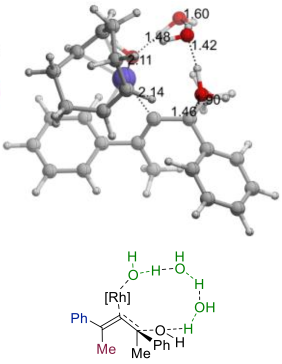

TS2_h

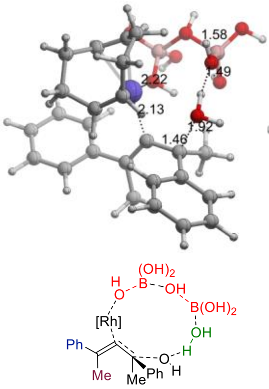

TS2_i

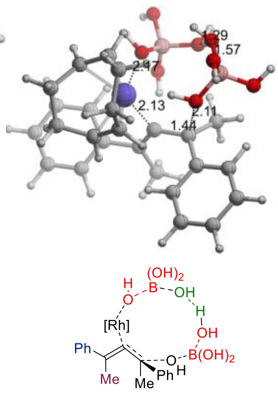

TS2_j

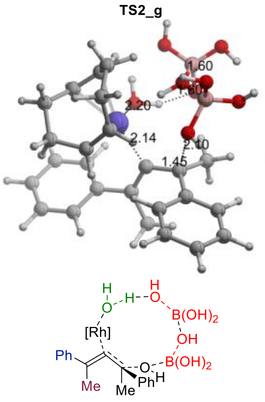

TS2_k

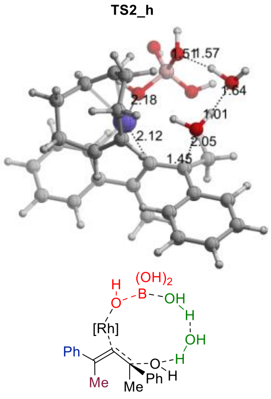

TS2_l

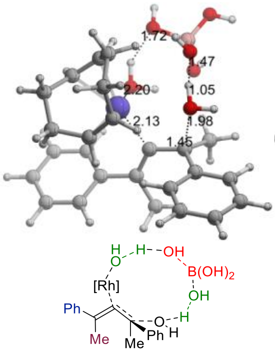

TS2_m

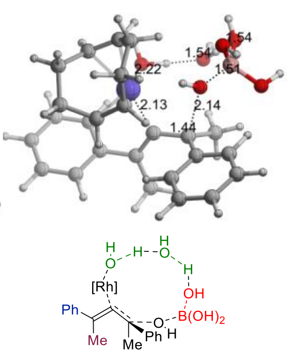

TS2_n

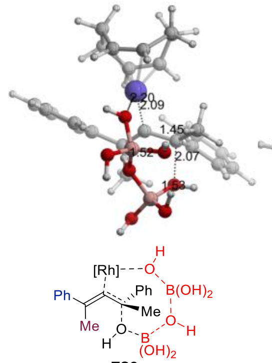

TS2_o

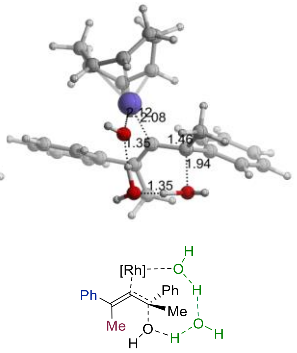

TS2_p

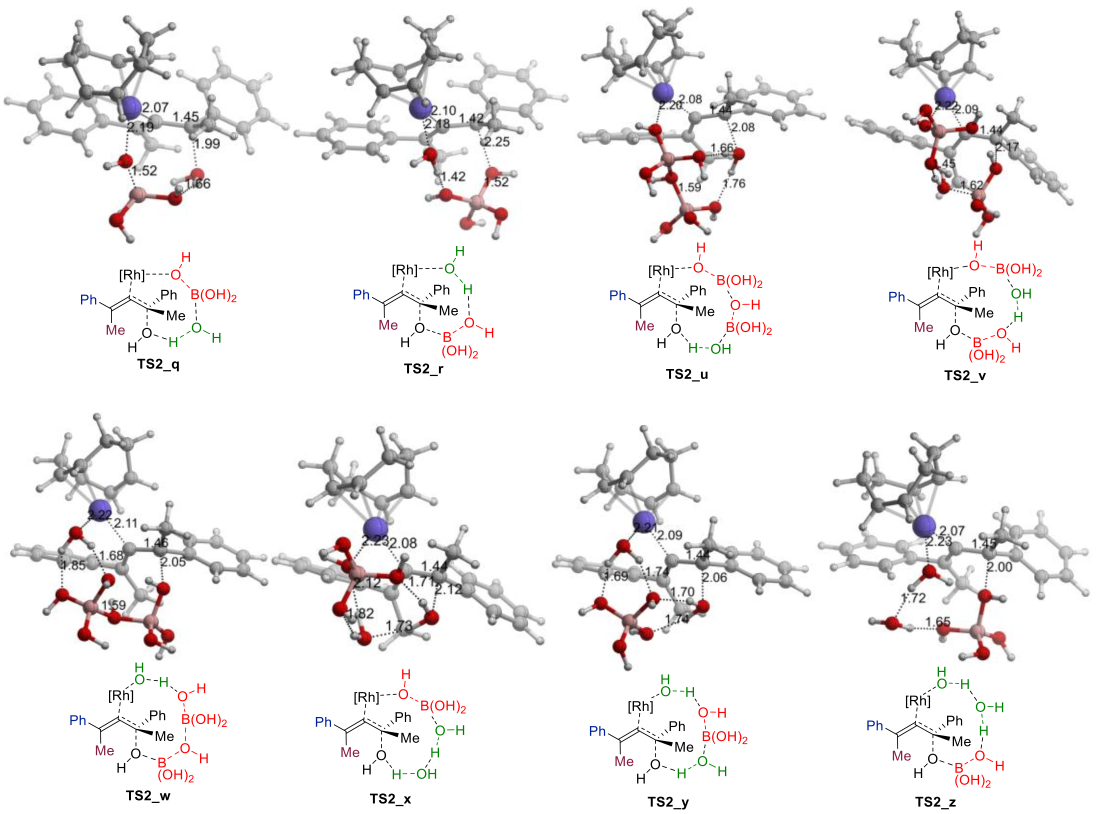

Supplementary Figure 6. 3D-structures for the transition states shown in Supplementary Figure 5. Bond lengths are given in angstroms.

Based on the single point energy calculations on the allene fragment in the two transition states, there is an energy difference of about 1.7 kcal/mol caused by the smaller C^1, C^2 and C^3 bond angle in *syn*-**TS2_i** (Supplementary Figure 7).

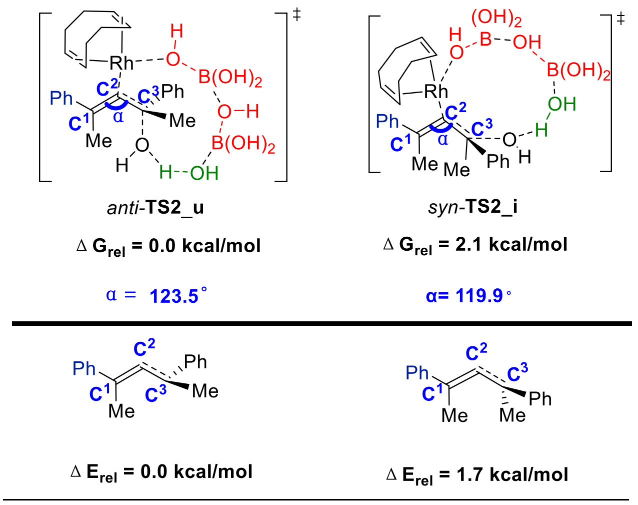

Supplementary Figure 7. Single point energy calculations on the allene fragment in the two TSs (*syn*-**TS2_i** and *anti*-**TS2_u**) at B3LYP-D3(BJ)/LANL2DZ, 6-31G(d,p) level.

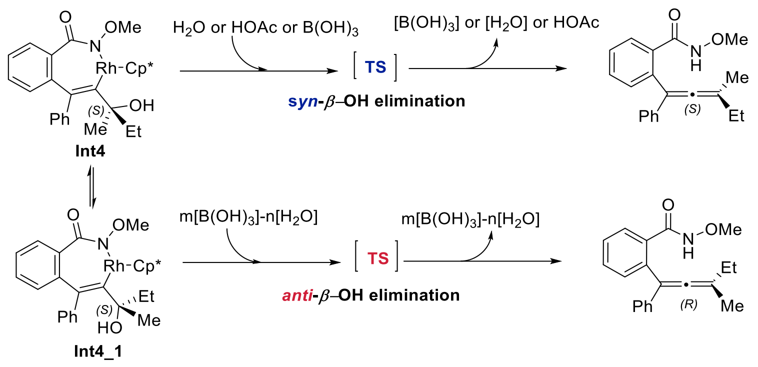

Supplementary Figure 8. The transition states for Rh(III)-*β*-OH elimination of Int4 assisted by water, acetic acid, or boric acid

The transition states for Rh(III)-*β*-OH elimination of **Int4** (Fig. 5b and Supplementary Figure 8) assisted by water, acetic acid, or boric acid are illlustrated in Supplementary Figure 9 (See Supplementary Figure 10 for the 3D-structures of these TSs). The transition states **TS3_a-c** are located as the transition structures each with one molecule of water [1H_2O], one molecule of HOAc [1HOAc], and one molecule of $B(OH)_3$ [1$B(OH)_3$], respectively. However, these TSs are all calculated to be unfavorable as compared with **TS3** (14.4 kcal/mol), due to the steric effect caused by Cp* ligand on the Rh(III) center with the assisting molecules (H_2O in **TS3_a**, HOAc in **TS3_b** and $B(OH)_3$ in **TS3_c**). Moreover, selected *anti-β*-OH elimination transition structures (**TS3_d-g**) are investigated: For the eight-membered cyclic transition structures **TS3_d-f**, the energy barriers are all calculated to be much higher (about 60 kcal/mol), due to the unfavorable steric effect between the Cp* ligand and the assisting molecules. Notably, in order to avoid the steric hinderance, Cp* ligand is bonded with the Rh(III) center through the η^1-coordination mode instead of the normal η^5-coordination in the structures of **TS3_d-f** (See Supplementary Figure 9 for the 3D-structures of these TSs). Even the [1H_2O]-[2$B(OH)_3$] participated ten-membered transition structure **TS3_g** features an energy barrier of 38.1 kcal/mol, which is much more unstable than **TS3**.

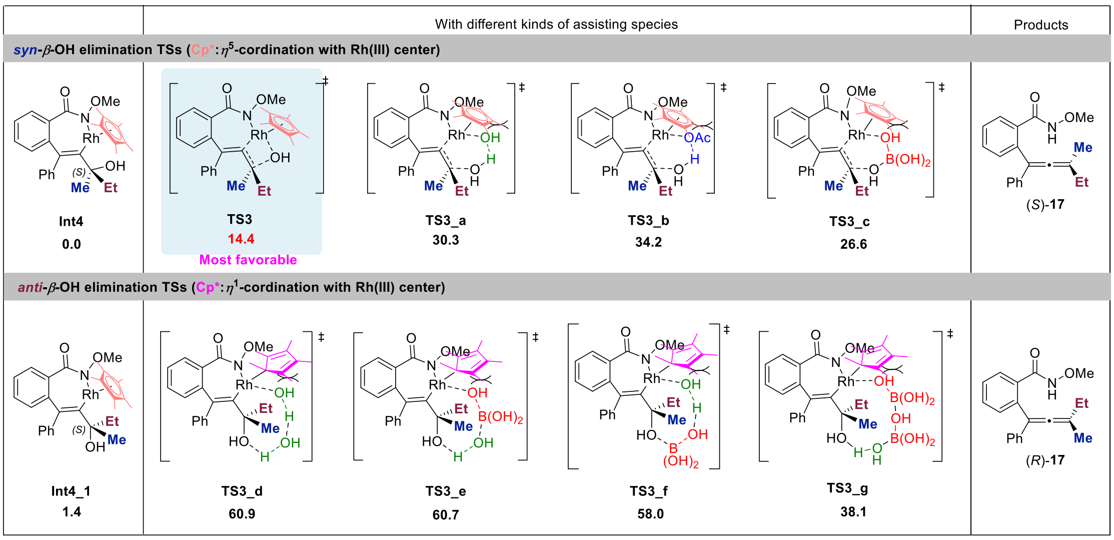

Supplementary Figure 9. The transition states and their corresponding activation energies for Rh(III)-*β*-OH elimination process of rhodacyclic intermediate **Int4**. (Free energies are given in kcal/mol.)

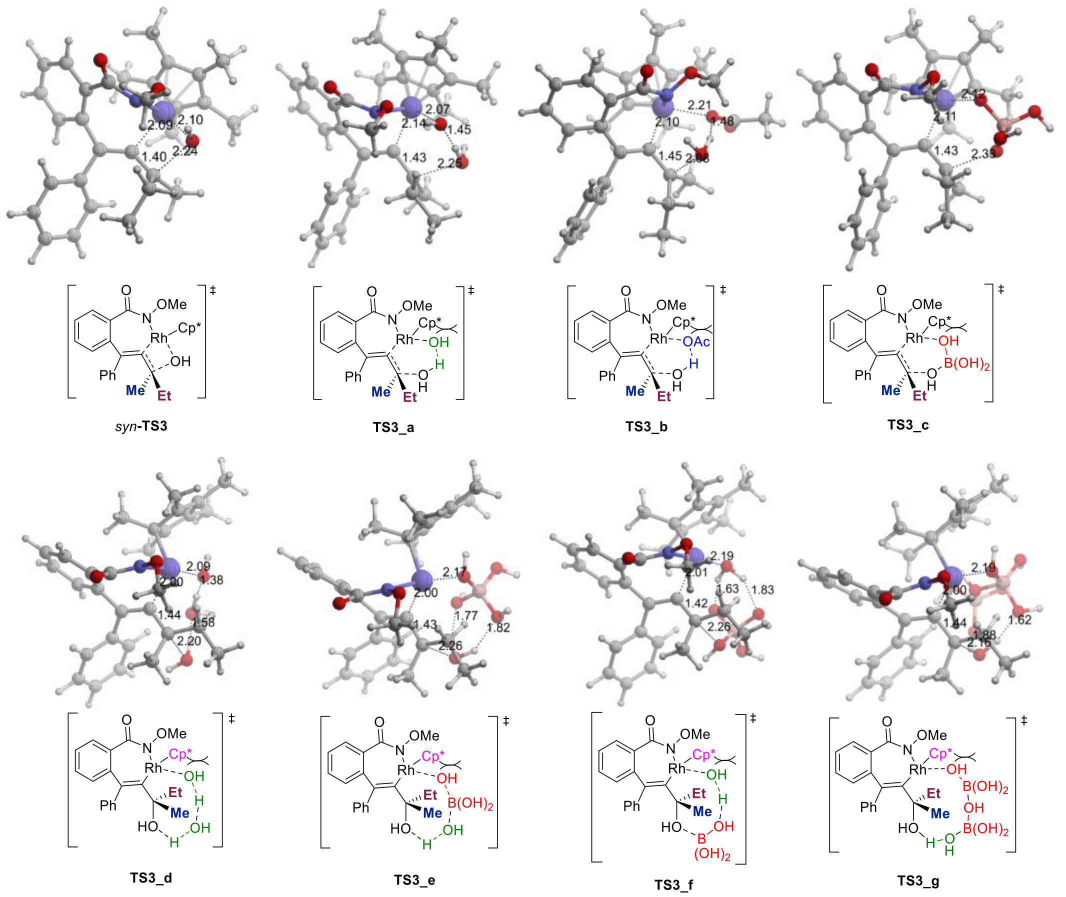

Supplementary Figure 10. 3D-structures for the transition states showed in Supplementary Figure 9. Bond lengths are given in angstroms. (Cp*:η^5-cordination with Rh(III) center; Cp*:η^1-cordination with Rh(III) center.)

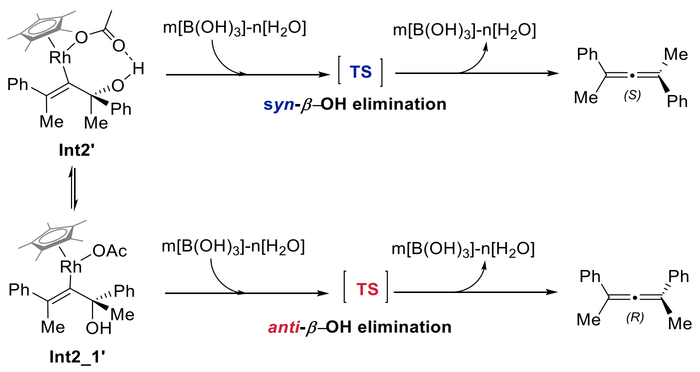

Supplementary Figure 11 The transition states for Rh(III)-*β*-OH elimination of Int2' assisted by boric acid or/and water

The transition states for Rh(III)-*β*-OH elimination of **Int2'** (Fig. 6a and Supplementary Figure 11) assisted by boric acid or/and water are illlustrated in Supplementary Figure 12 (See Supplementary Figure 13 for the 3D-structures of these TSs). The *syn*-*β*-OH elimination step would afford (*S*)-**3aa** *via* **TS4** requiring a free energy barrier of 26.4 kcal/mol. When the assisting species (water or/and boric acid) are taken into consideration, the *syn*-*β*-OH elimination TSs (**TS4_a-b**) and *anti*-*β*-OH elimination TSs (**TS4_c-e**) are all less favorable as compared with **TS4**, due to the hinderance between Cp* ligand on the Rh(III) center and the assisting molecules (Supplementary Figure 12).

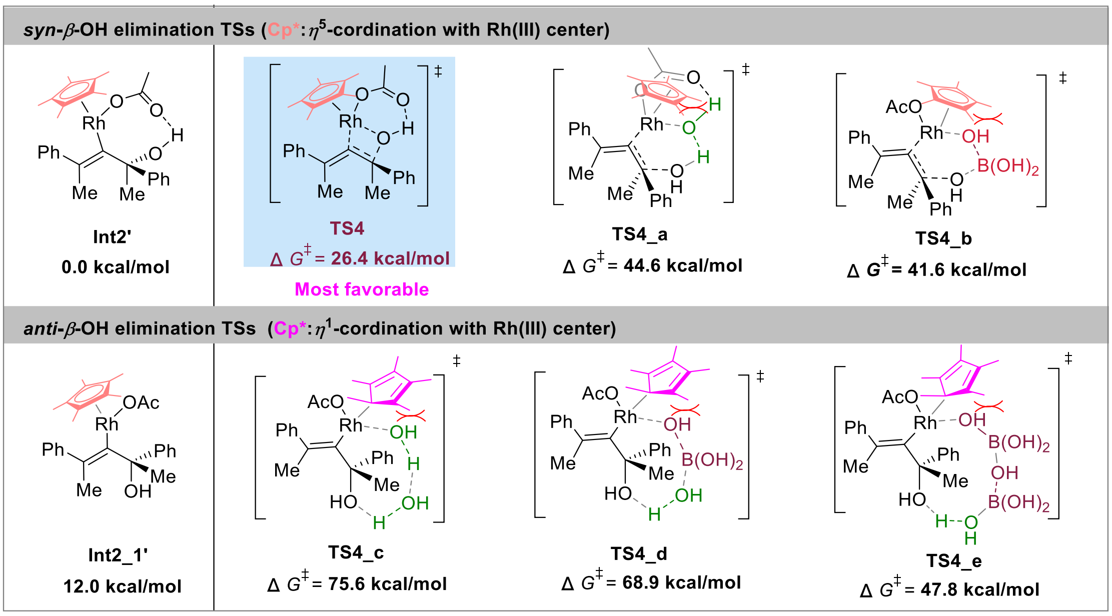

Supplementary Figure 12. The transition states and their corresponding activation energies for boric acid or/and water assisted Rh(III)-*β*-OH elimination process of **Int2'**. (Free energies are given in kcal/mol.)

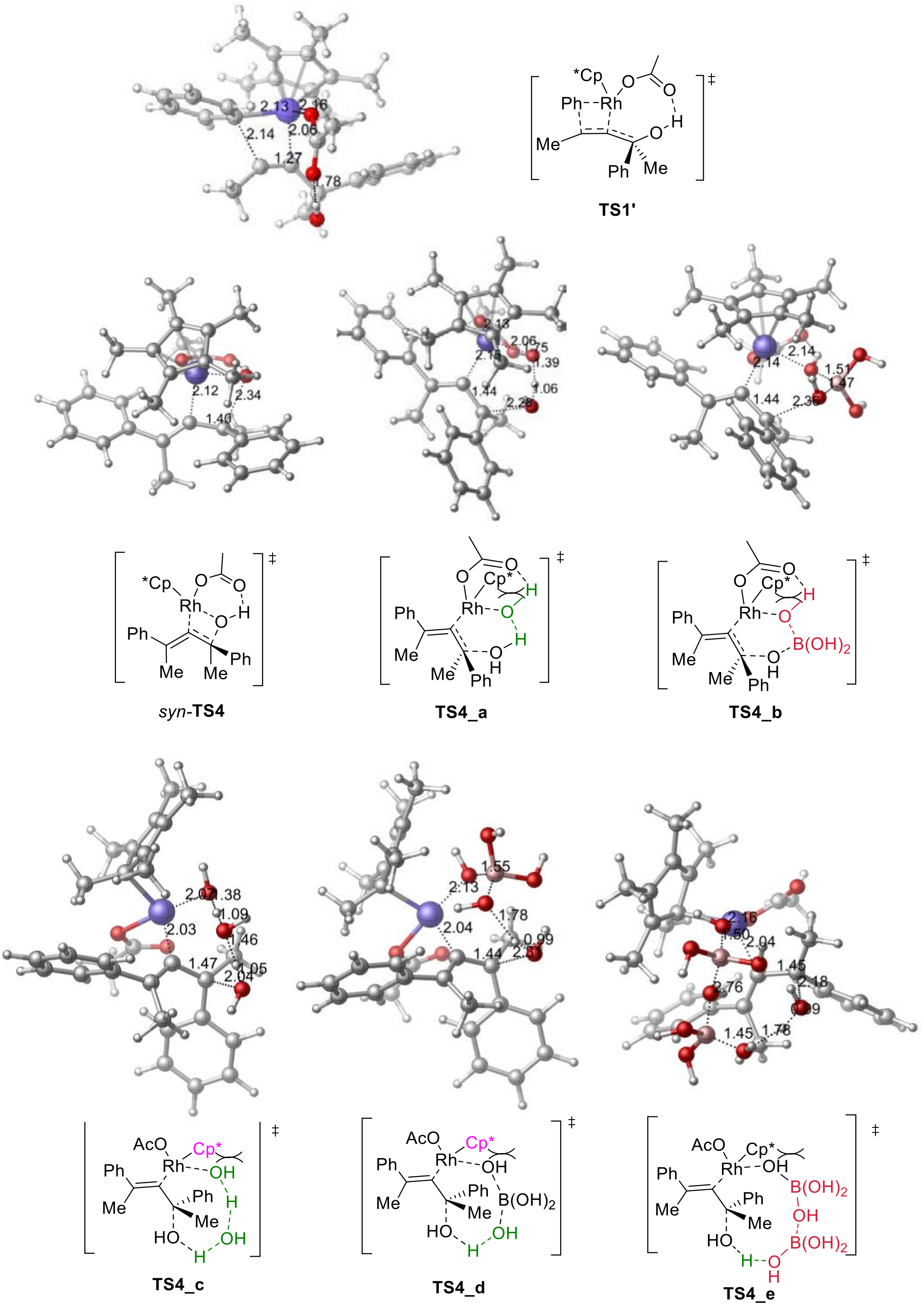

Supplementary Figure 13. 3D-structures for the transition states shown in Fig. 6a and Supplementary Figure 12. Bond lengths are given in angstroms. (Cp*:η^5-cordination with Rh(III) center; Cp*:η^1-cordination with Rh(III) center.)

The single point energy calculations on the allene fragments in the two transition states reveal that the smaller C1, C2 and C3 bond angle in *syn*-**TS4_b** destabilizes the structure by about 14.0 kcal/mol as compared to *syn*-**TS4** (Supplementary Figure 14).

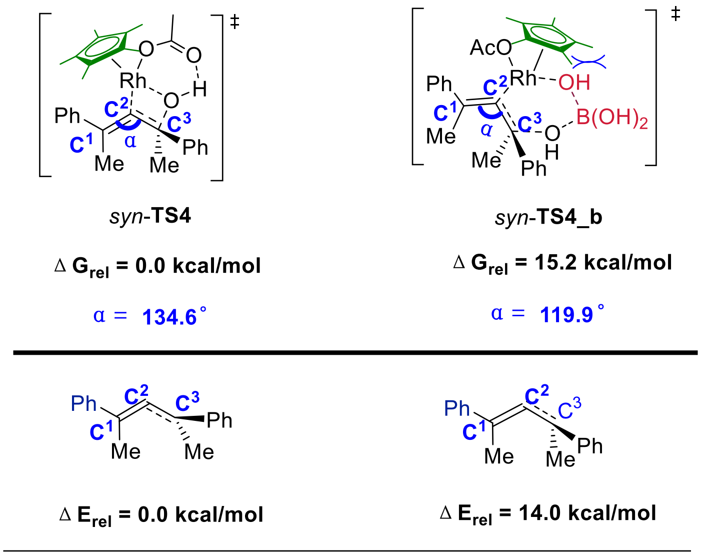

Supplementary Figure 14. Single point energy calculations on the allene fragment in the two TSs (*syn*-**TS4** and *anti*-**TS4_b**) at B3LYP-D3(BJ)/LANL2DZ, 6-31G(d,p) level.

We had also conducted the related DFT calculations on the TSs for *anti-β*-OH elimination without assisting species in both $[Cp^*RhCl_2]_2$- and $[Rh(COD)OH]_2$-catalyzed reaction systems. Unfortunately, our attempts to locate the *anti-β*-OH elimination transition states without any assisting species proved to be unsuccessful. All the efforts to locate any TSs for the *anti-β*-OH elimination converged towards its precursor **Int2** (or **Int2'**), probably due to the leaving hydroxyl anion in the system. The relaxed potential energy surface scan (PES Scan) for the direct *anti-β*-OH elimination was conducted on **int2_1'**, taking the C^3-O and C^2-Rh bond lengths as two variables (Supplementary Figure 15). As can be seen from the potential energy surface in Supplementary Figure 15, the total energy consistently increases with the elongation of the C^3-O and C^2-Rh bond lengths. And no saddle point has been found on the potential energy surface. Hence, we believed that the participation of the assisting species in constructing cyclic transition states is critical for the *anti-β*-OH elimination pathway.

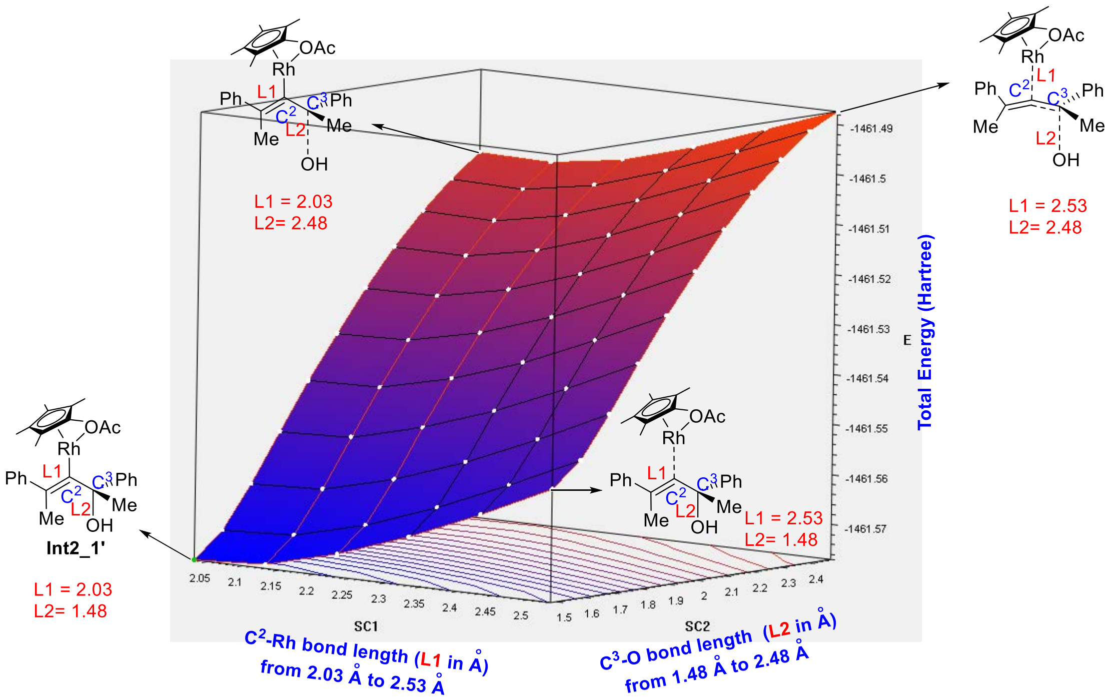

Supplementary Figure 15. Relaxed PES scan for the direct *anti-β*-OH elimination on **int2_1'** at B3LYP-D3(BJ)/LANL2DZ, 6-31G (d,p) level.

Energies of Intermediates and transition states

Supplementary Table 1. Electronic energies (E_{elec}), Gibbs free energies (G_{298}), thermal correction to Gibbs free energy ($cor\ G_{gas}$), solvation energies (E_{sol}), solvation free energies (G_{sol}) in 1,4-dioxane (ε = 2.2099) for all stationary points of the process.

species	E_{elec} (a.u.)	G_{298} (a.u.)	$cor\ G_{gas}$ (a.u.)	E_{sol} (a.u.)	G_{sol} (a.u.)
**Int1**	-1154.953869	-1154.538339	0.415529	-1155.297127	-1154.881598
**TS1**	-1154.942109	-1154.524334	0.417775	-1155.289883	-1154.872108
**Int2**	-1154.99762	-1154.576922	0.420698	-1155.337712	-1154.917014
syn-**TS2**	-1154.9648	-1154.546919	0.417881	-1155.303855	-1154.885974
**Int3**	-1154.994979	-1154.576989	0.41799	-1155.333394	-1154.915404
**B(OH)3**	-252.4980117	-252.475292	0.02272	-252.4674192	-252.4446992
**Re_a**	-1407.518539	-1407.052521	0.466018	-1407.816027	-1407.350009
**TS2_a**	-1407.481155	-1407.01609	0.465065	-1407.780278	-1407.315213
**H2O**	-76.4203103	-76.416585	0.003726	-76.421341	-76.41761500
**Re_b**	-1231.438912	-1230.992619	0.446293	-1231.767774	-1231.321481
**TS2_b**	-1231.400203	-1230.959573	0.44063	-1231.724648	-1231.284018
**2B(OH)3**	-505.0152331	-504.952234	0.062999	-504.9452137	-504.8822147
**Re_c**	-1660.01352	-1659.495113	0.518408	-1660.260733	-1659.742325
**TS2_c**	-1659.988941	-1659.474807	0.514134	-1660.242466	-1659.728332

**2H2O**	-152.8537685	-152.832972	0.020797	-152.8500387	-152.8292417
**Re_d**	-1307.887865	-1307.417777	0.470087	-1308.204375	-1307.734288
**TS2_d**	-1307.848522	-1307.383718	0.464804	-1308.160129	-1307.695325
**$B(OH)_3$-H_2O**	-328.9296658	-328.887521	0.042145	-328.8919009	-328.8497559
**Re_e**	-1483.945654	-1483.454929	0.490725	-1484.23053	-1483.739805
**TS2_e**	-1483.925273	-1483.436568	0.488705	-1484.211089	-1483.722384
**Re_f**	-1483.956784	-1483.469397	0.487388	-1484.246776	-1483.759388
**TS2_f**	-1483.932912	-1483.444464	0.488449	-1484.218577	-1483.730128
**3$B(OH)_3$**	-757.5361294	-757.425802	0.110328	-757.4201357	-757.3098077
**Re_g**	-1912.526147	-1911.96214	0.564007	-1912.729875	-1912.165868
**TS2_g**	-1912.510963	-1911.948313	0.562649	-1912.717893	-1912.155244
**3H2O**	-229.3043956	-229.258251	0.046145	-229.2869936	-229.2408486
**Re_h**	-1384.333673	-1383.841236	0.492437	-1384.641051	-1384.148614
**TS2_h**	-1384.291598	-1383.802317	0.48928	-1384.592483	-1384.103203
**2$B(OH)_3$-H_2O**	-581.4211956	-581.331086	0.090109	-581.3368744	-581.2467654
**Re_i**	-1736.460776	-1735.921484	0.539292	-1736.701677	-1736.162385
**TS2_i**	-1736.440339	-1735.904271	0.536068	-1736.684323	-1736.148255
**Re_j**	-1736.483741	-1735.95068	0.533061	-1736.728425	-1736.195364
**TS2_j**	-1736.442261	-1735.908844	0.533417	-1736.684628	-1736.151211
**Re_k**	-1736.479276	-1735.947582	0.531694	-1736.730058	-1736.198364
**TS2_k**	-1736.444187	-1735.907043	0.537144	-1736.687267	-1736.150123
**$B(OH)_3$-2H_2O**	-405.3643467	-405.296577	0.067769	-405.3121748	-405.2444058
**Re_l**	-1560.391561	-1559.880098	0.511462	-1560.666264	-1560.154802
**TS2_l**	-1560.373921	-1559.863086	0.510836	-1560.64857	-1560.137734
**Re_m**	-1560.399018	-1559.882131	0.516887	-1560.673564	-1560.156677
**TS2_m**	-1560.374549	-1559.861607	0.512942	-1560.649792	-1560.13685
**Re_n**	-1560.404154	-1559.892923	0.511232	-1560.686345	-1560.175113
**TS2_n**	-1560.373961	-1559.864876	0.509085	-1560.650075	-1560.14099
**Re_o**	-1660.023509	-1659.50927	0.514239	-1660.278226	-1659.763987
**TS2_o**	-1659.994917	-1659.483872	0.511045	-1660.250452	-1659.739407
**Re_p**	-1307.891371	-1307.422352	0.469019	-1308.209126	-1307.740107
**TS2_p**	-1307.852977	-1307.389052	0.463925	-1308.165103	-1307.701178
**Re_q**	-1483.9558	-1483.467878	0.487922	-1484.24807	-1483.760148
**TS2_q**	-1483.934652	-1483.444715	0.489936	-1484.220025	-1483.730089
**Re_r**	-1483.953665	-1483.466411	0.487254	-1484.246128	-1483.758874
**TS2_r**	-1483.924507	-1483.436397	0.48811	-1484.215438	-1483.727328
**Re_u**	-1736.471539	-1735.931947	0.539592	-1736.710992	-1736.171400
**TS2_u**	-1736.453519	-1735.914816	0.538702	-1736.69792	-1736.159218
**Re_v**	-1736.469543	-1735.931052	0.53849	-1736.715029	-1736.176539
**TS2_v**	-1736.445646	-1735.909158	0.536488	-1736.687061	-1736.150573
**Re_w**	-1736.461727	-1735.923672	0.538055	-1736.706249	-1736.168194
**TS2_w**	-1736.449239	-1735.908747	0.540491	-1736.69032	-1736.149829
**Re_x**	-1560.399017	-1559.885088	0.513929	-1560.670976	-1560.157047

**TS2_x**	-1560.38242	-1559.869308	0.513111	-1560.657407	-1560.144296
**Re_y**	-1560.401641	-1559.885807	0.515834	-1560.675186	-1560.159352
**TS2_y**	-1560.385212	-1559.869787	0.515425	-1560.658395	-1560.14297
**Re_z**	-1560.410675	-1559.793638	0.513411	-1560.689599	-1560.176188
**TS2_z**	-1560.370297	-1559.85753	0.512767	-1560.647017	-1560.13425
**Int4**	-1554.98058	-1554.453645	0.526936	-1555.143922	-1554.616986
**Int4_b**	-1554.959754	-1554.433407	0.526347	-1555.141139	-1554.614792
syn-**TS3**	-1554.944339	-1554.419612	0.524727	-1555.11881	-1554.594083
**TS3_a**	-1631.371945	-1630.823601	0.548603	-1631.534956	-1630.986353
**HOAc**	-229.0984854	-229.063789	0.034697	-229.0379576	-229.0032606
**TS3_b**	-1784.052856	-1783.468514	0.583613	-1784.149426	-1783.565813
**TS3_c**	-1807.449414	-1806.87726	0.572154	-1807.591404	-1807.01925
**TS3_d**	-1707.704794	-1707.133547	0.571248	-1707.918662	-1707.347414
**TS3_e**	-1883.860507	-1883.265800	0.594707	-1883.967583	-1883.372876
**TS3_f**	-1883.859368	-1883.264755	0.594614	-1883.969359	-1883.374745
**TS3_g**	-2136.385058	-2135.740763	0.644295	-2136.44733	-2135.803035
**Int1'**	-1461.535193	-1461.032778	0.502415	-1461.755045	-1461.25263
**TS1'**	-1461.514248	-1461.013493	0.500756	-1461.734657	-1461.233901
**Int2'**	-1461.60505	-1461.10042	0.504624	-1461.811265	-1461.306641
**Int2_b'**	-1461.57753	-1461.07385	0.503684	-1461.791127	-1461.287443
syn-**TS4**	-1461.555689	-1461.054518	0.501171	-1461.765736	-1461.264565
**TS4_a**	-1714.06191	-1713.51333	0.548584	-1714.23357	-1713.684986
**TS4_b**	-1537.98066	-1537.45679	0.523867	-1538.176973	-1537.653106
**TS4_c**	-1614.39287	-1613.84785	0.545028	-1614.560458	-1614.01543
**TS4_d**	-1790.47467	-1789.90363	0.571041	-1790.617718	-1790.046677
**TS4_e**	-2042.99944	-2042.38001	0.619423	-2043.096605	-2042.477182

3. Supplementary ^{1}H and ^{13}C NMR and HPLC spectra

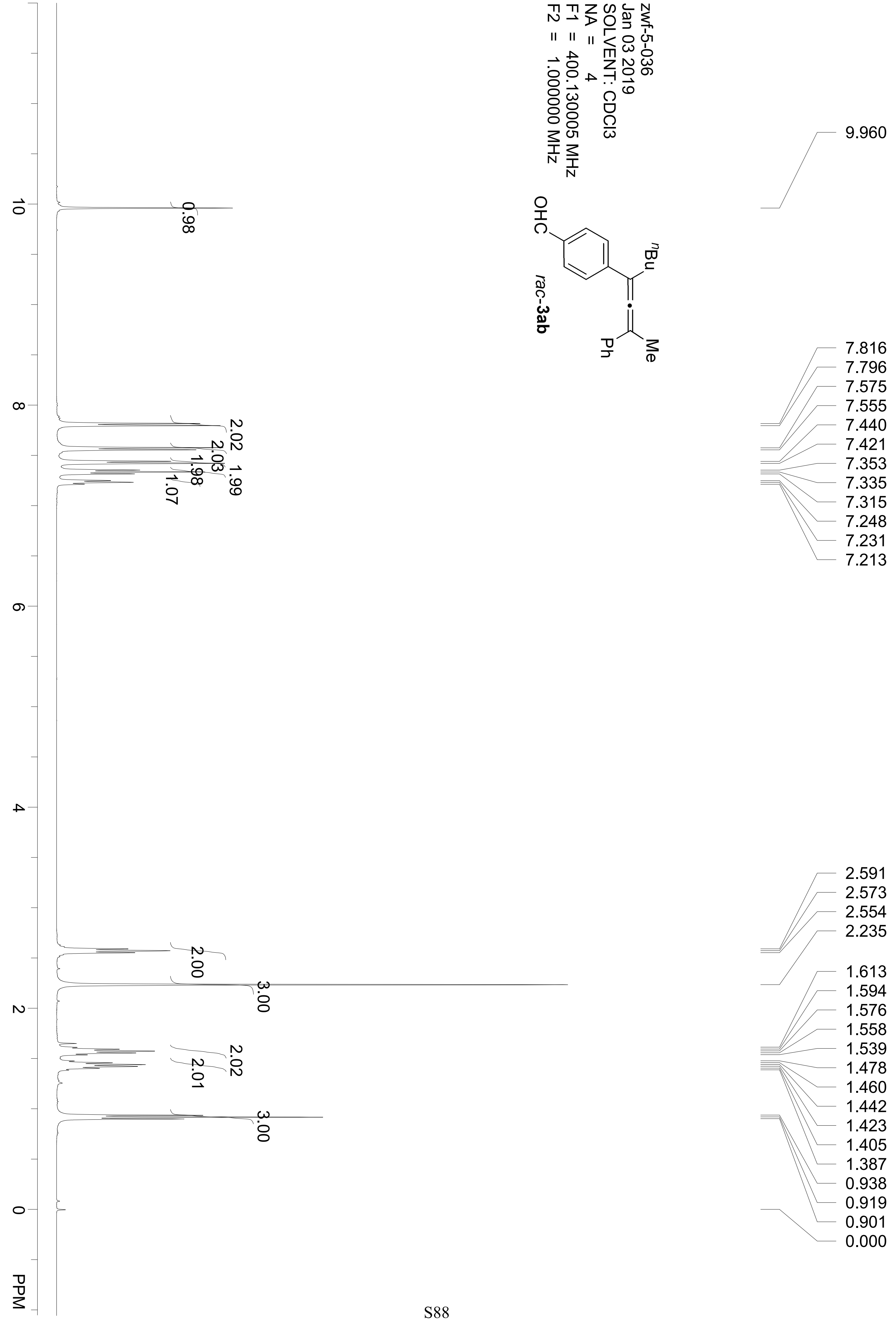

Supplementary Figure 16. ^{1}H NMR (400 MHz, $CDCl_3$) spectrum for *rac*-**3ab**

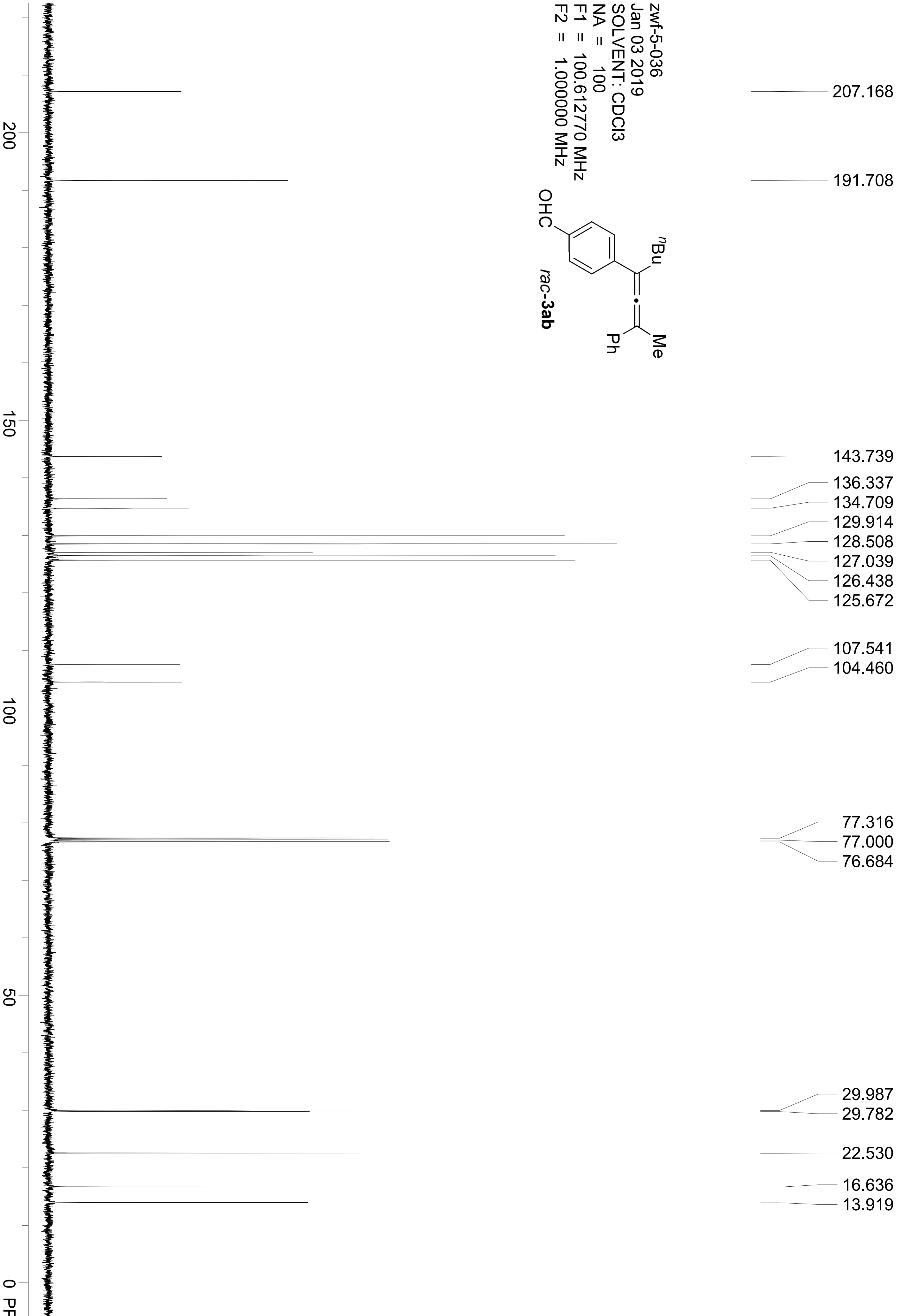

Supplementary Figure 17. ^{13}C NMR (100 MHz, CDCl$_3$) spectrum for *rac*-**3ab**

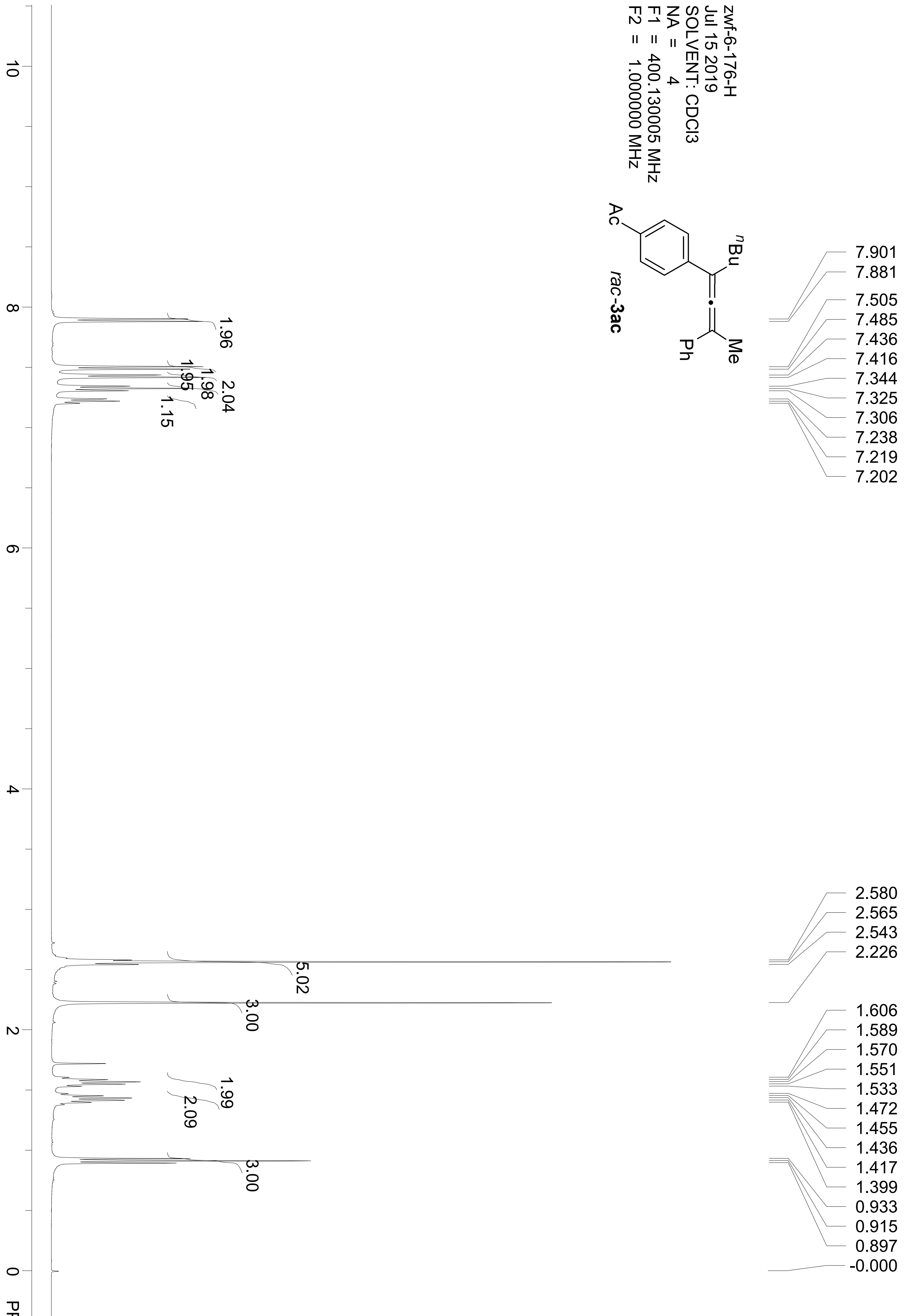

Supplementary Figure 18. ^{1}H NMR (400 MHz, $CDCl_3$) spectrum for *rac*-**3ac**

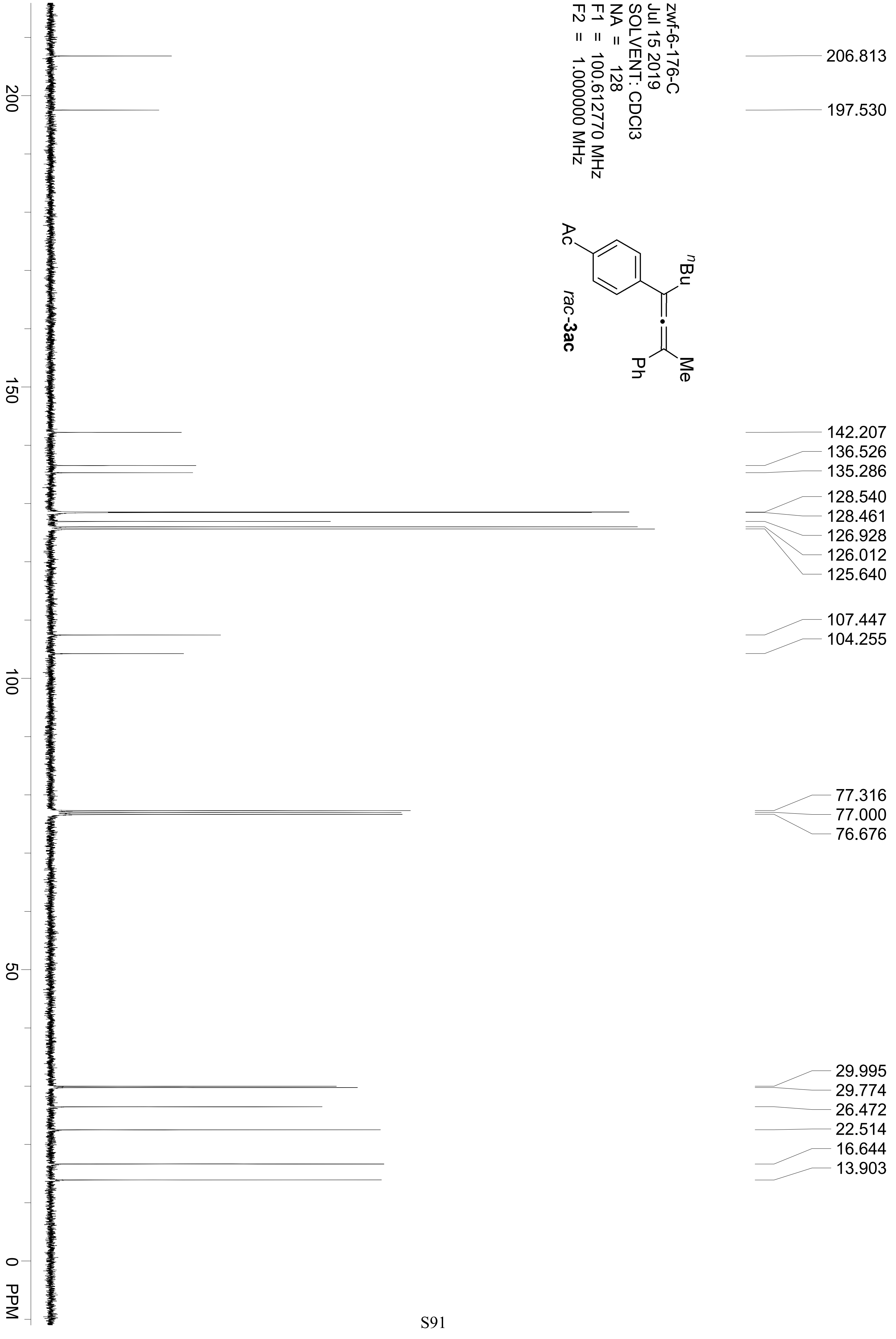

Supplementary Figure 19. ^{13}C NMR (100 MHz, $CDCl_3$) spectrum for *rac*-**3ac**

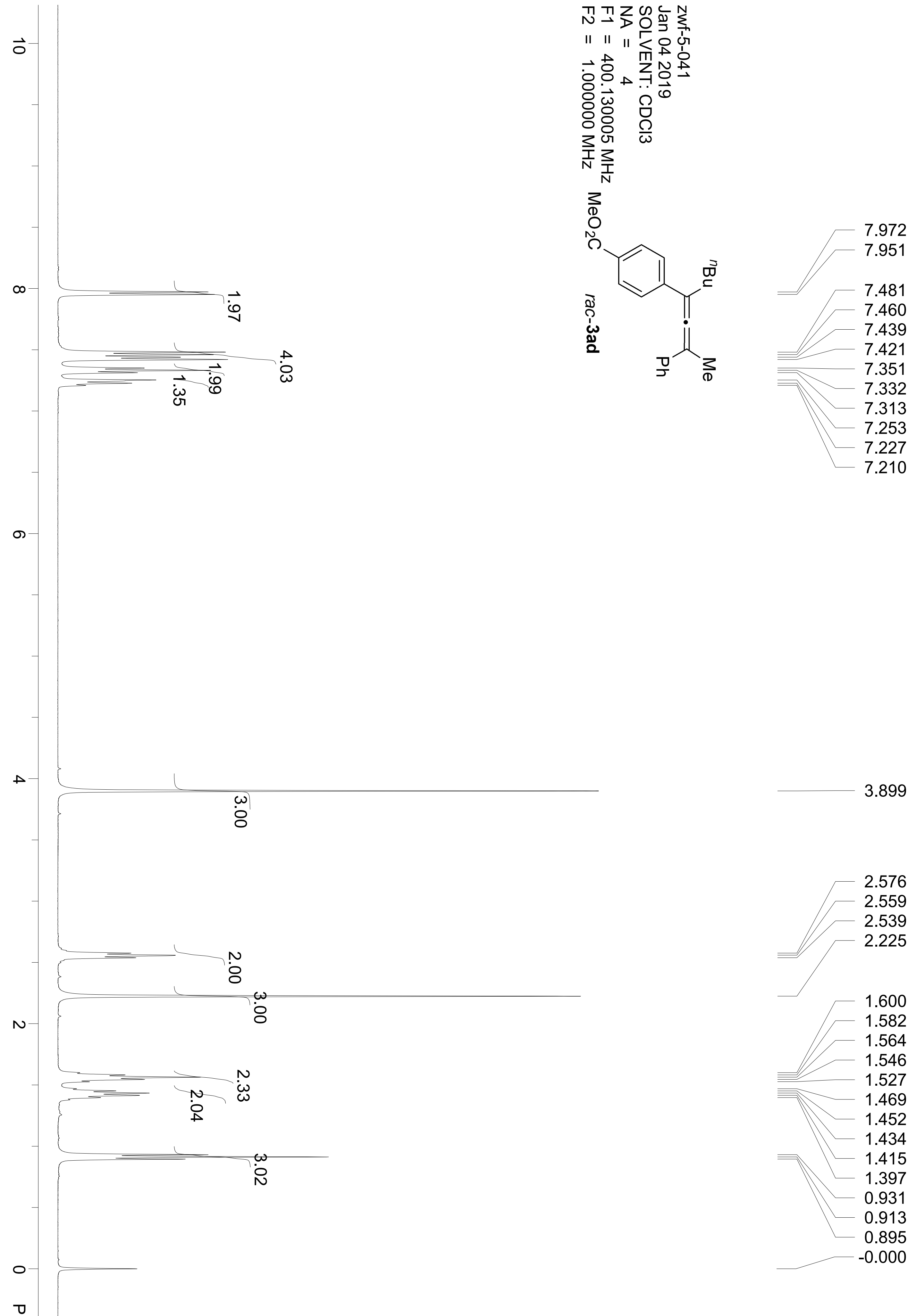

Supplementary Figure 20. ^{1}H NMR (400 MHz, $CDCl_3$) spectrum for *rac*-**3ad**

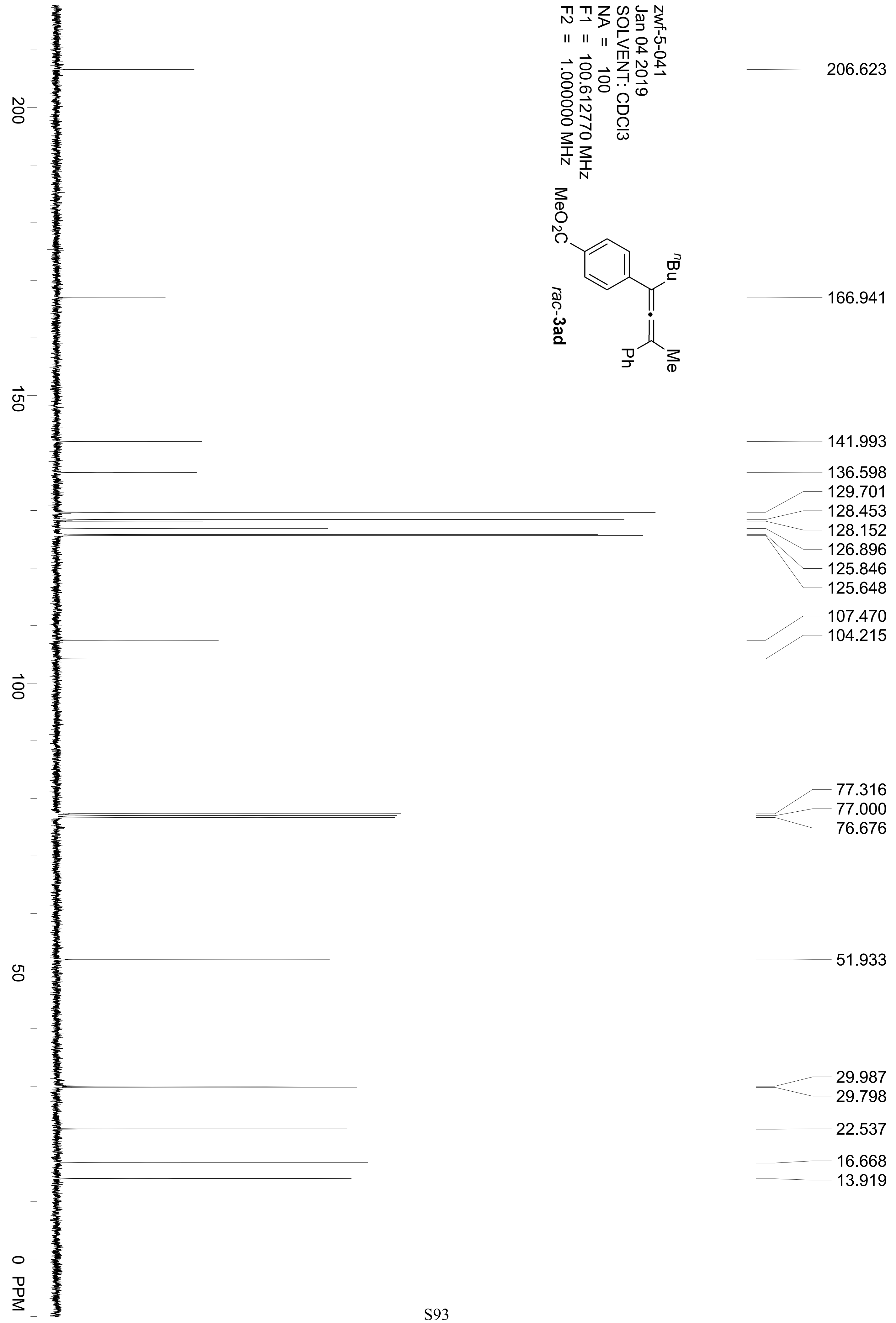

Supplementary Figure 21. ^{13}C NMR (100 MHz, $CDCl_3$) spectrum for *rac*-**3ad**

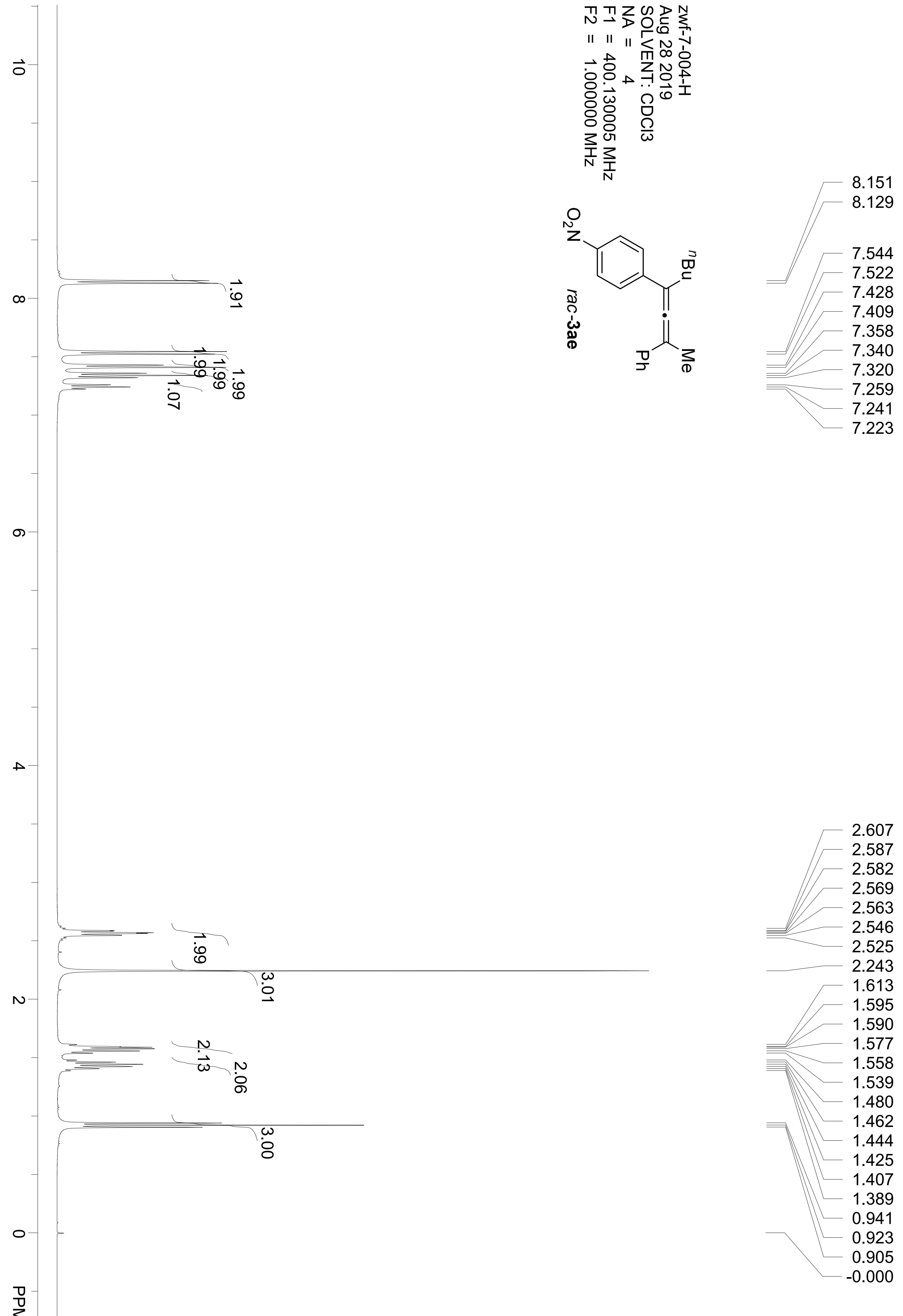

Supplementary Figure 22. ^{1}H NMR (400 MHz, $CDCl_3$) spectrum for *rac*-**3ae**

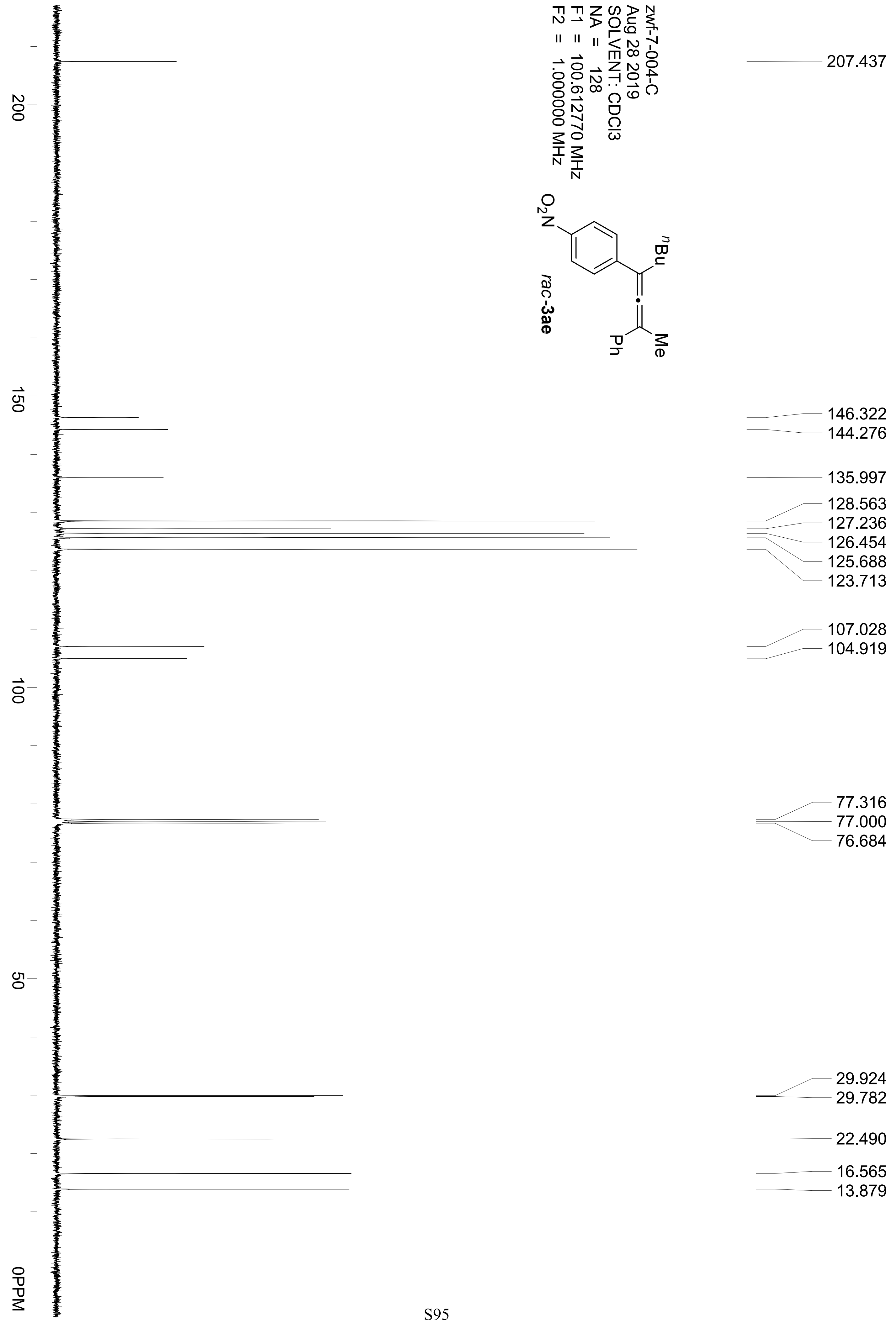

Supplementary Figure 23. ^{13}C NMR (100 MHz, $CDCl_3$) spectrum for *rac*-**3ae**

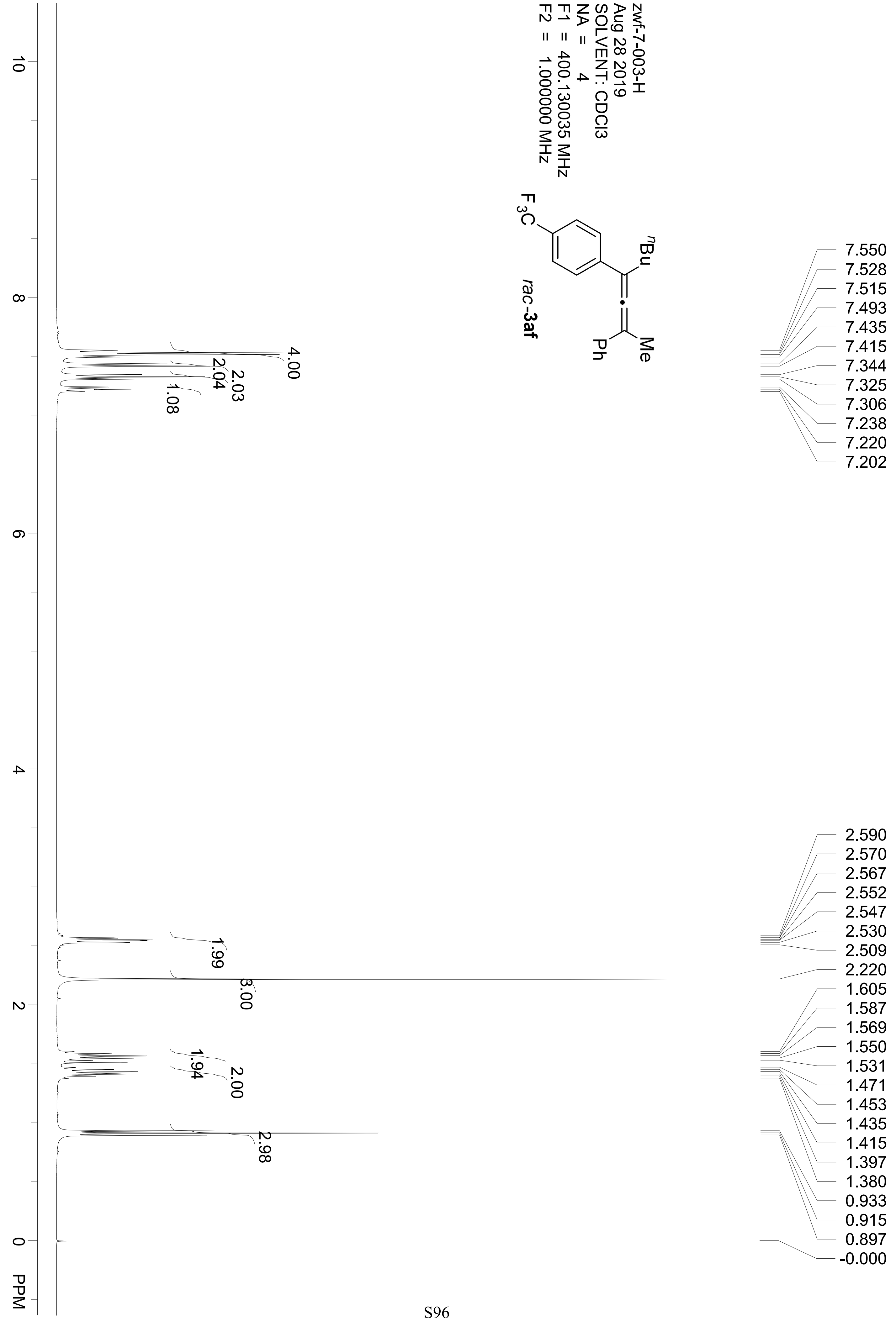

Supplementary Figure 24. ^{1}H NMR (400 MHz, $CDCl_3$) spectrum for *rac*-**3af**

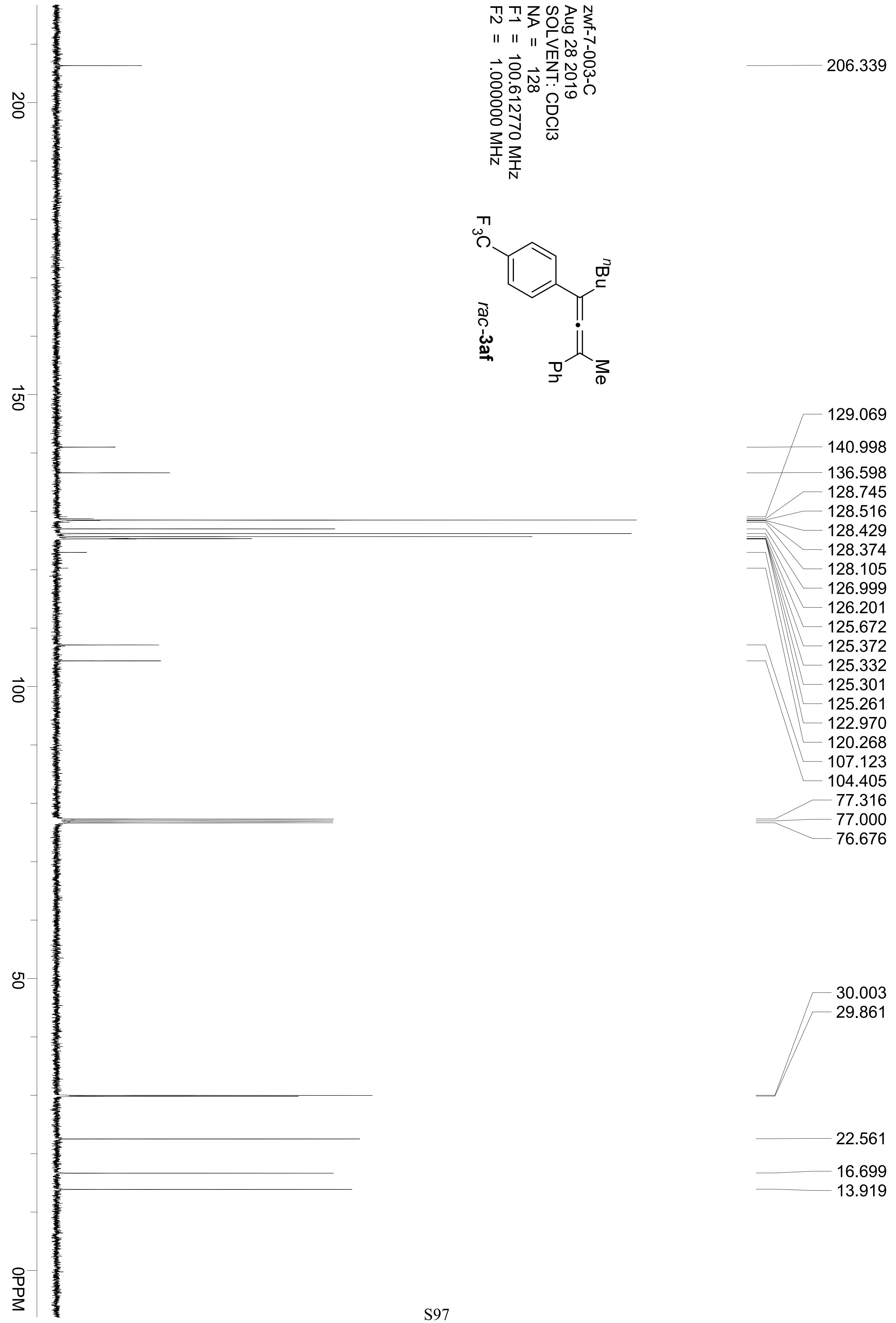

Supplementary Figure 25. ^{13}C NMR (100 MHz, CDCl$_3$) spectrum for *rac*-**3af**

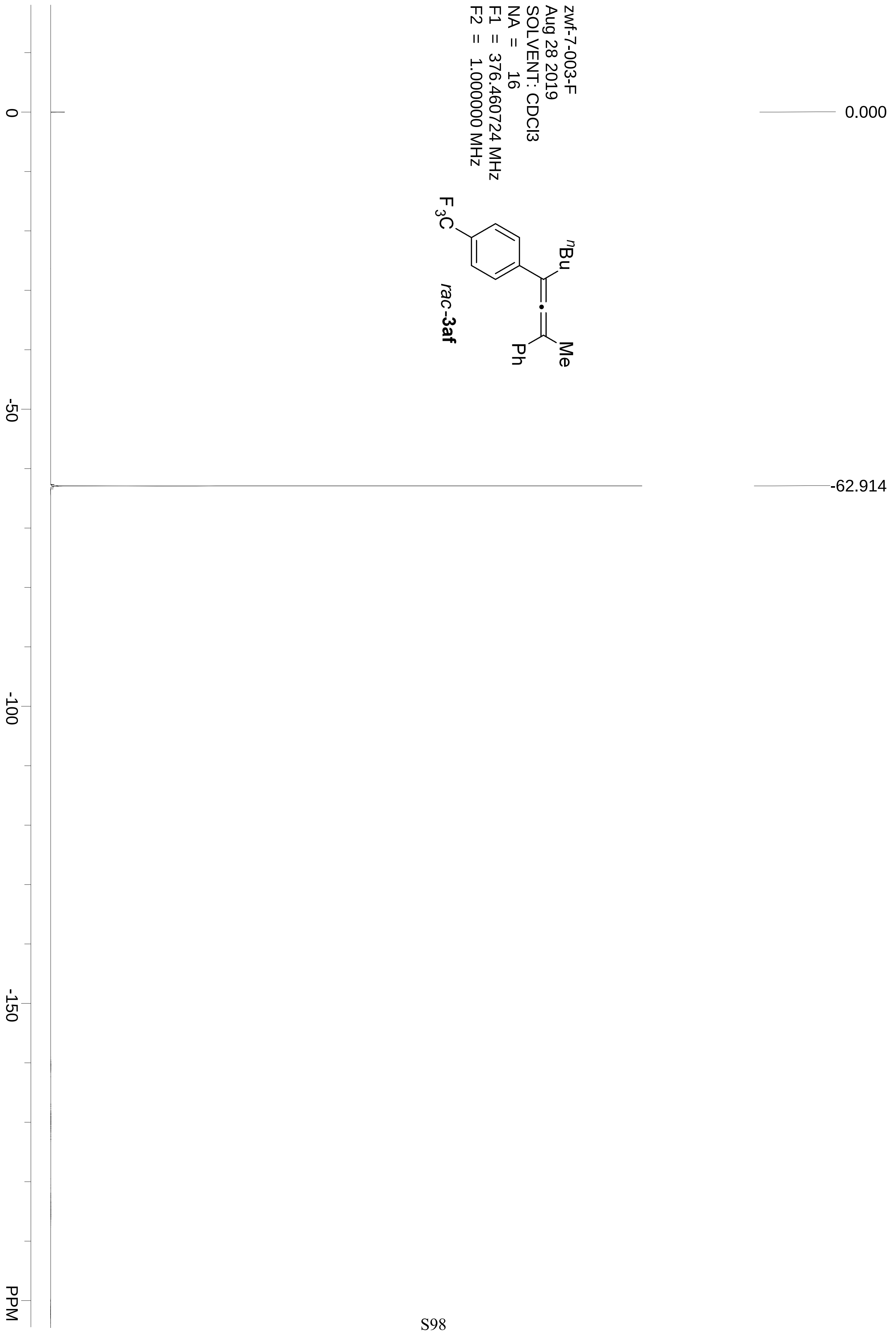

Supplementary Figure 26. ^{19}F NMR (376 MHz, $CDCl_3$) spectrum for *rac*-**3af**

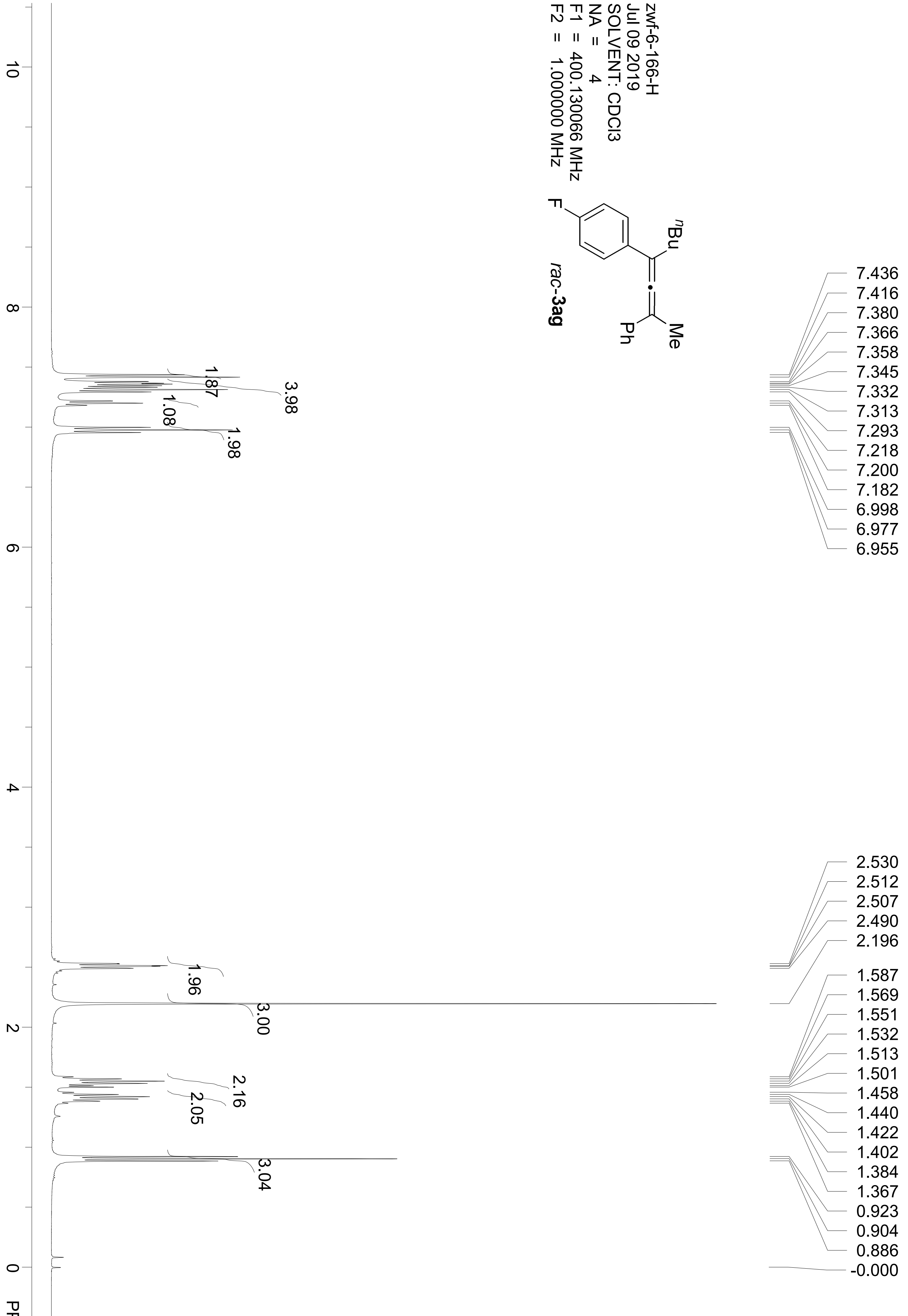

Supplementary Figure 27. ^{1}H NMR (400 MHz, $CDCl_3$) spectrum for *rac*-**3ag**

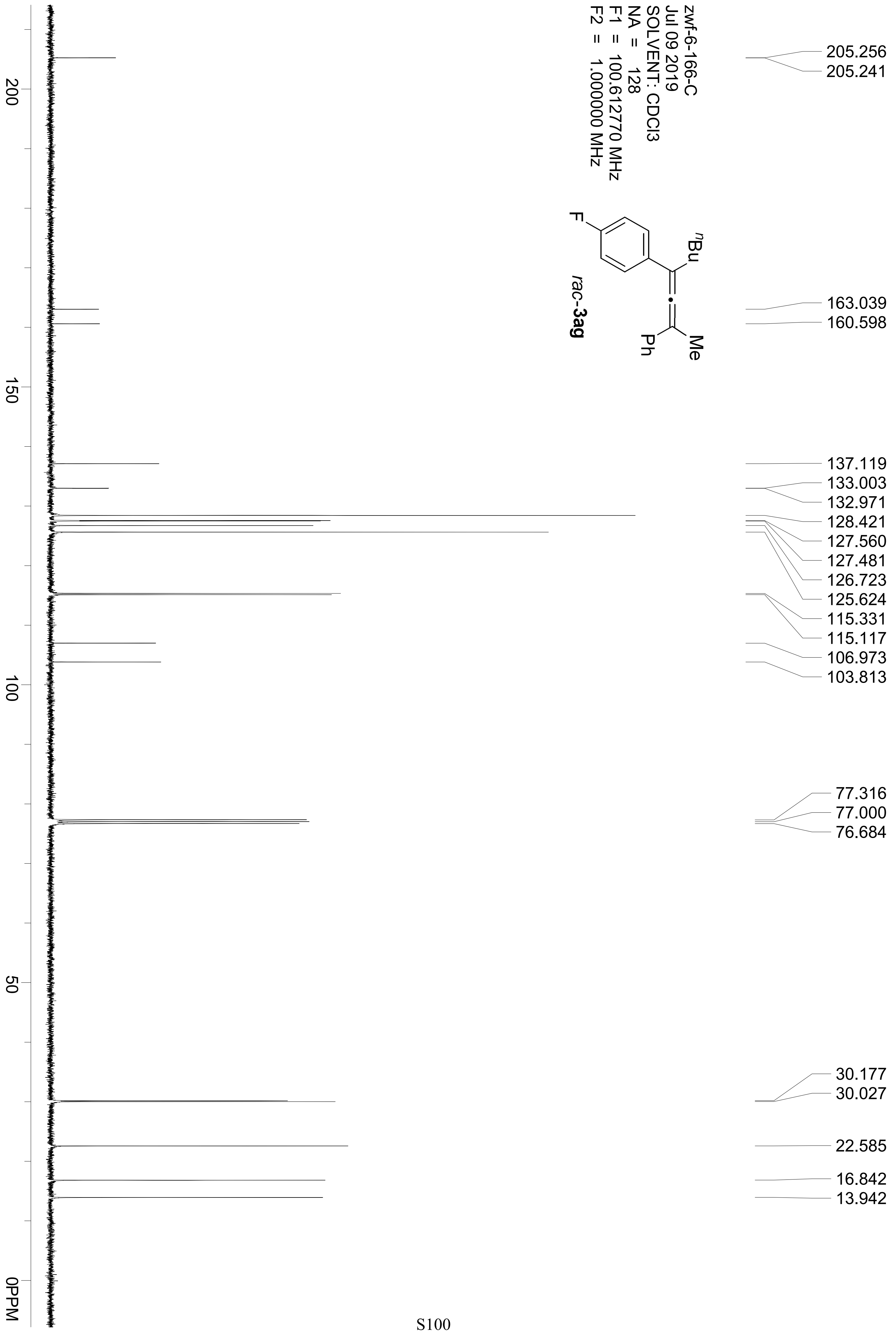

Supplementary Figure 28. ^{13}C NMR (100 MHz, $CDCl_3$) spectrum for *rac*-**3ag**

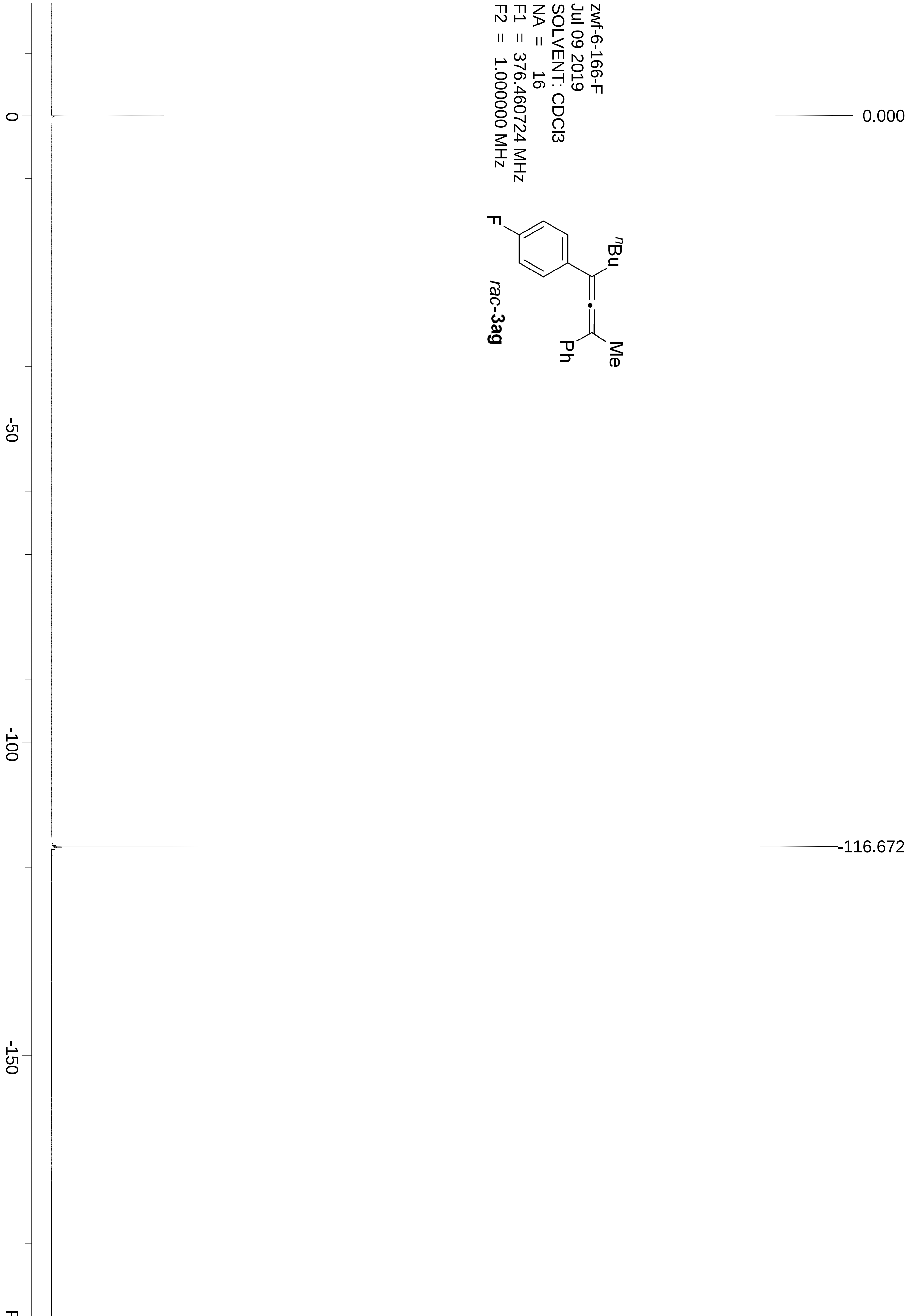

Supplementary Figure 29. ^{19}F NMR (376 MHz, $CDCl_3$) spectrum for *rac*-**3ag**

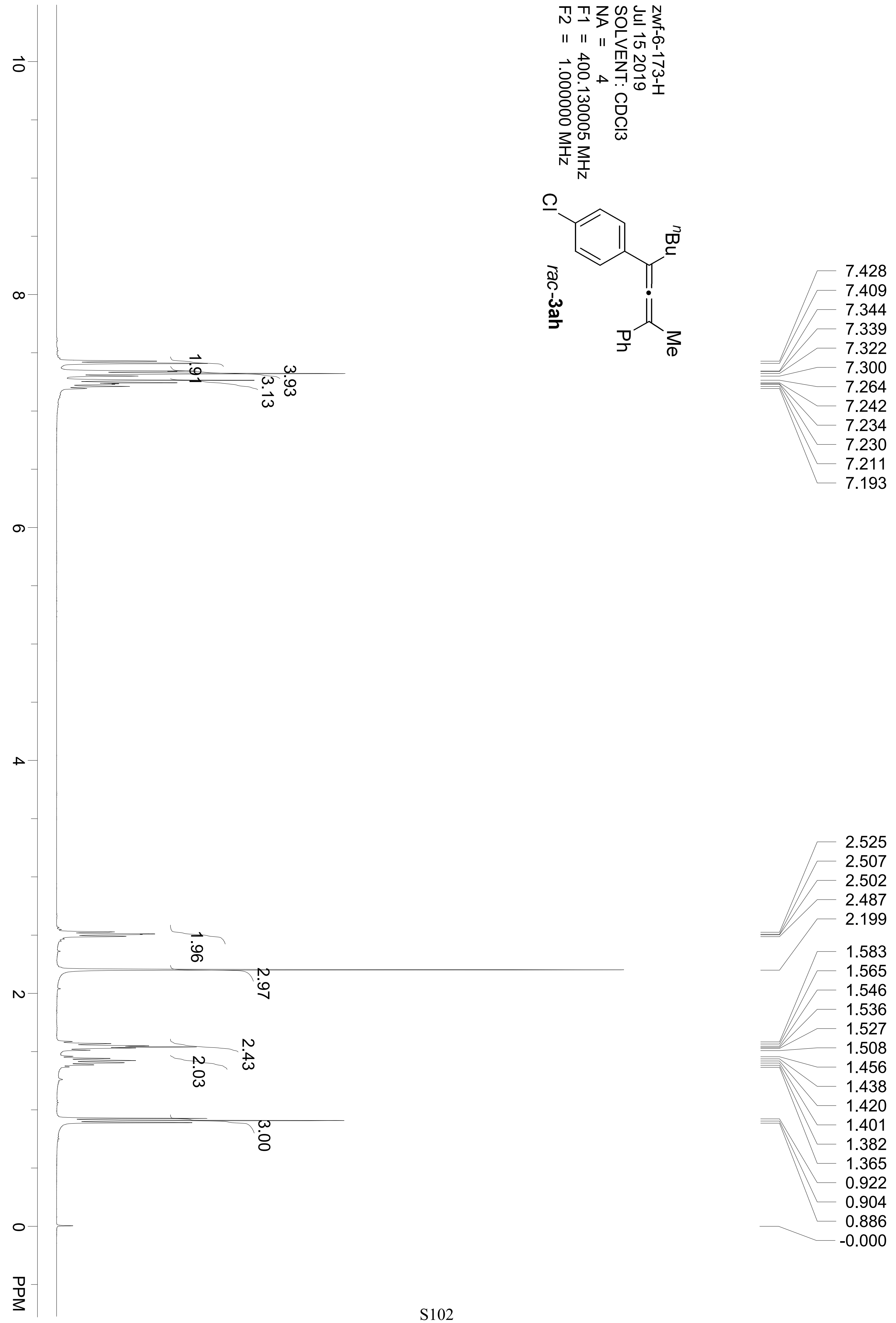

Supplementary Figure 30. ^{1}H NMR (400 MHz, $CDCl_3$) spectrum for *rac*-**3ah**

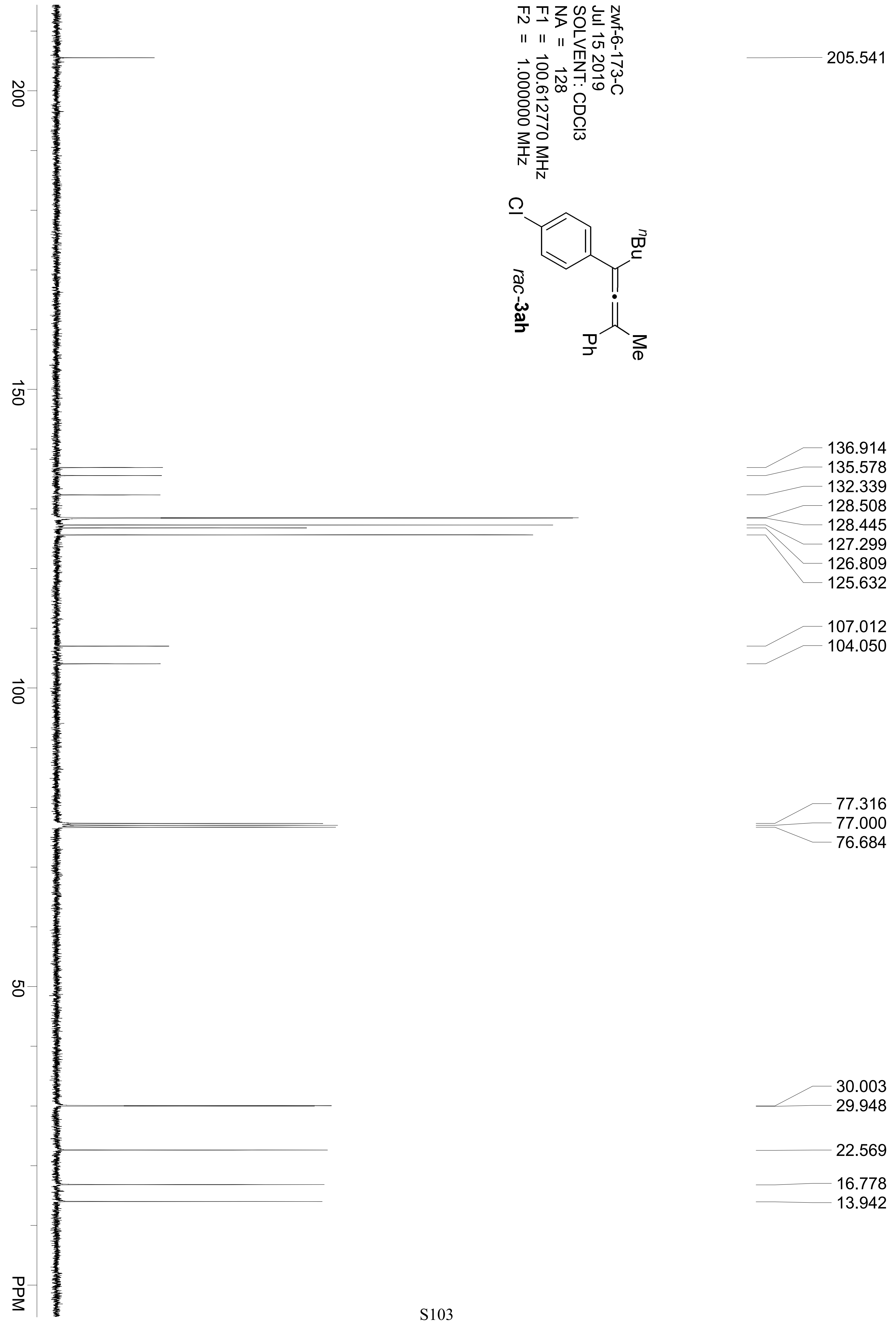

Supplementary Figure 31. ^{13}C NMR (100 MHz, $CDCl_3$) spectrum for *rac*-**3ah**

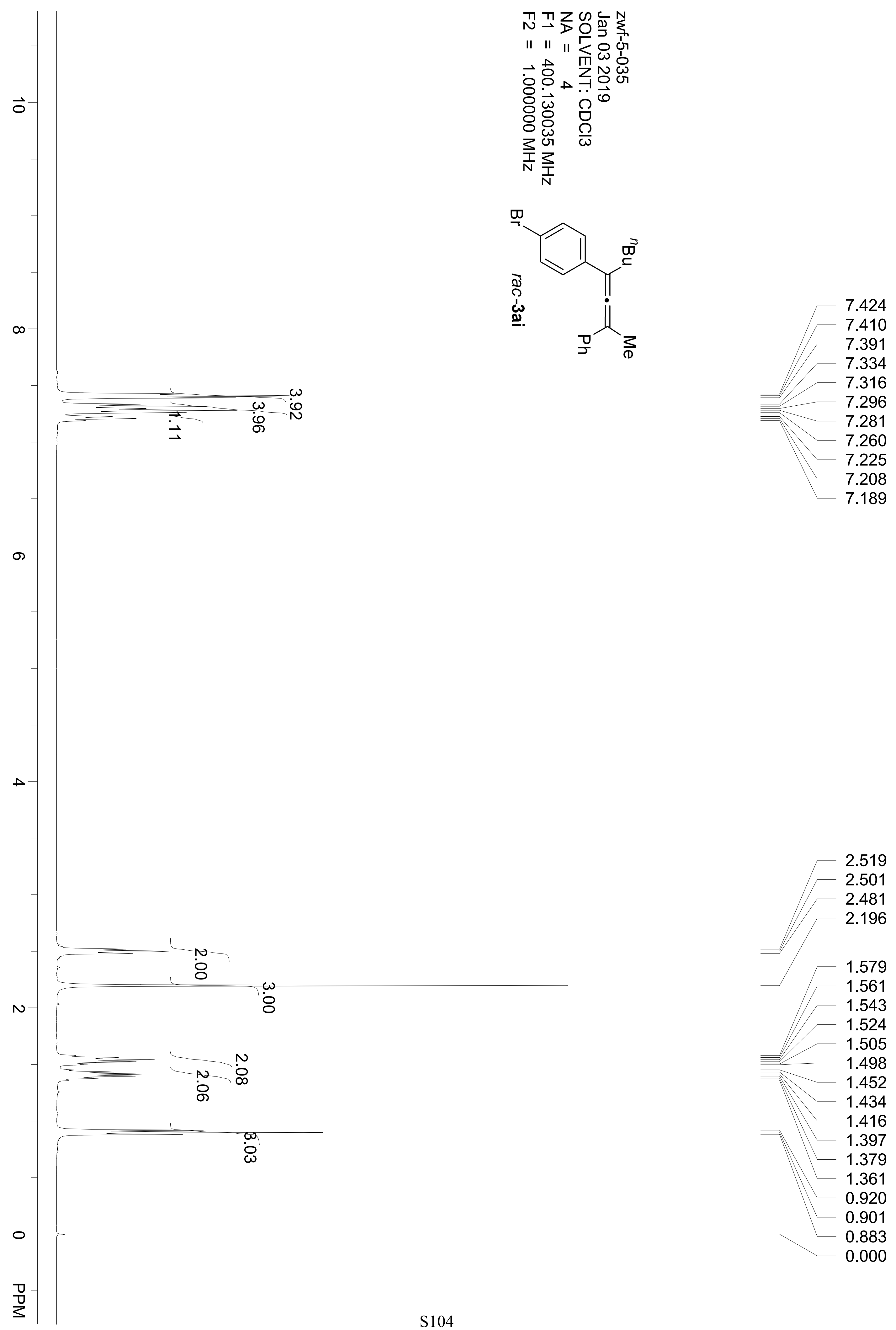

Supplementary Figure 32. ^{1}H NMR (400 MHz, $CDCl_3$) spectrum for *rac*-**3ai**

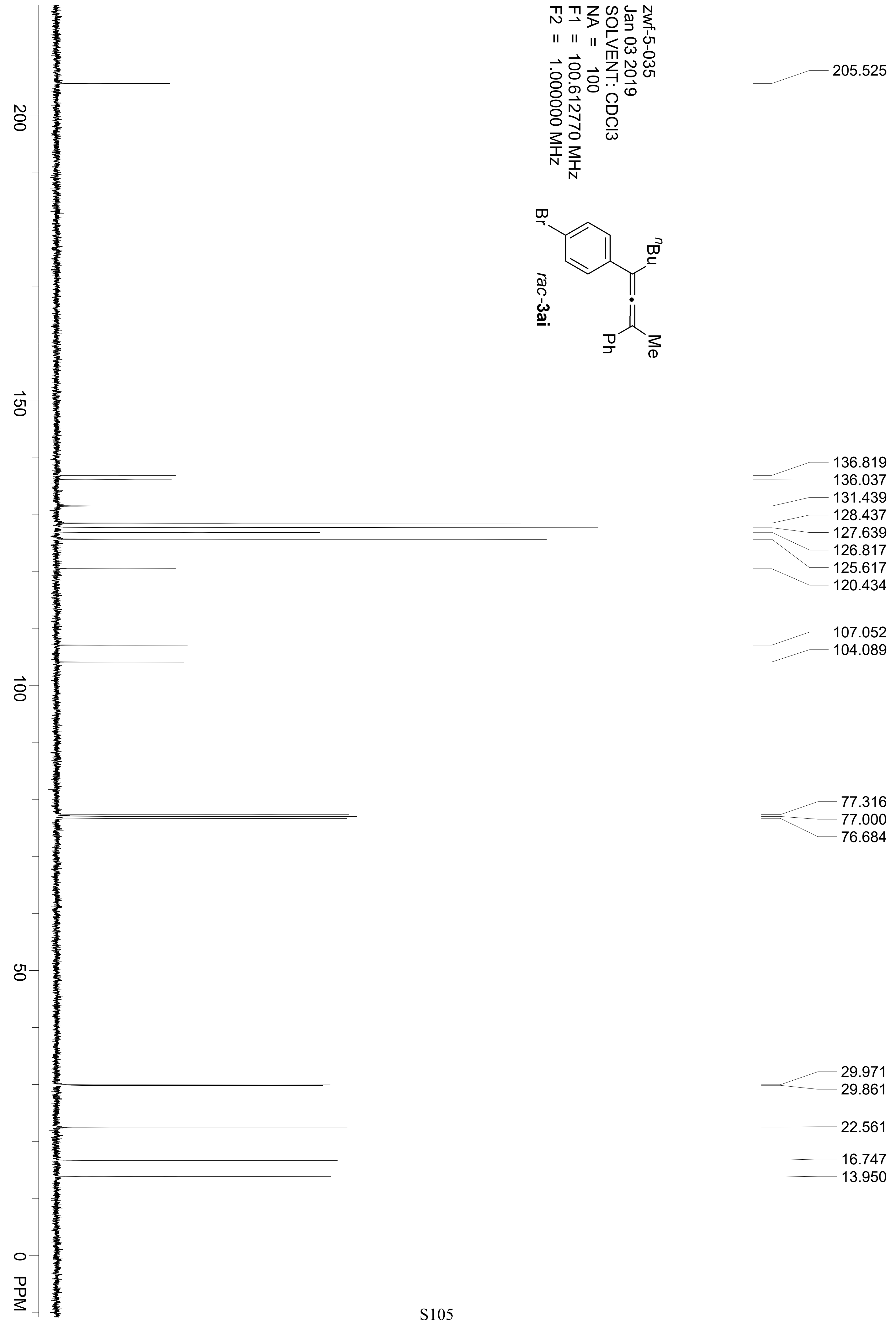

Supplementary Figure 33. ^{13}C NMR (100 MHz, $CDCl_3$) spectrum for *rac*-**3ai**

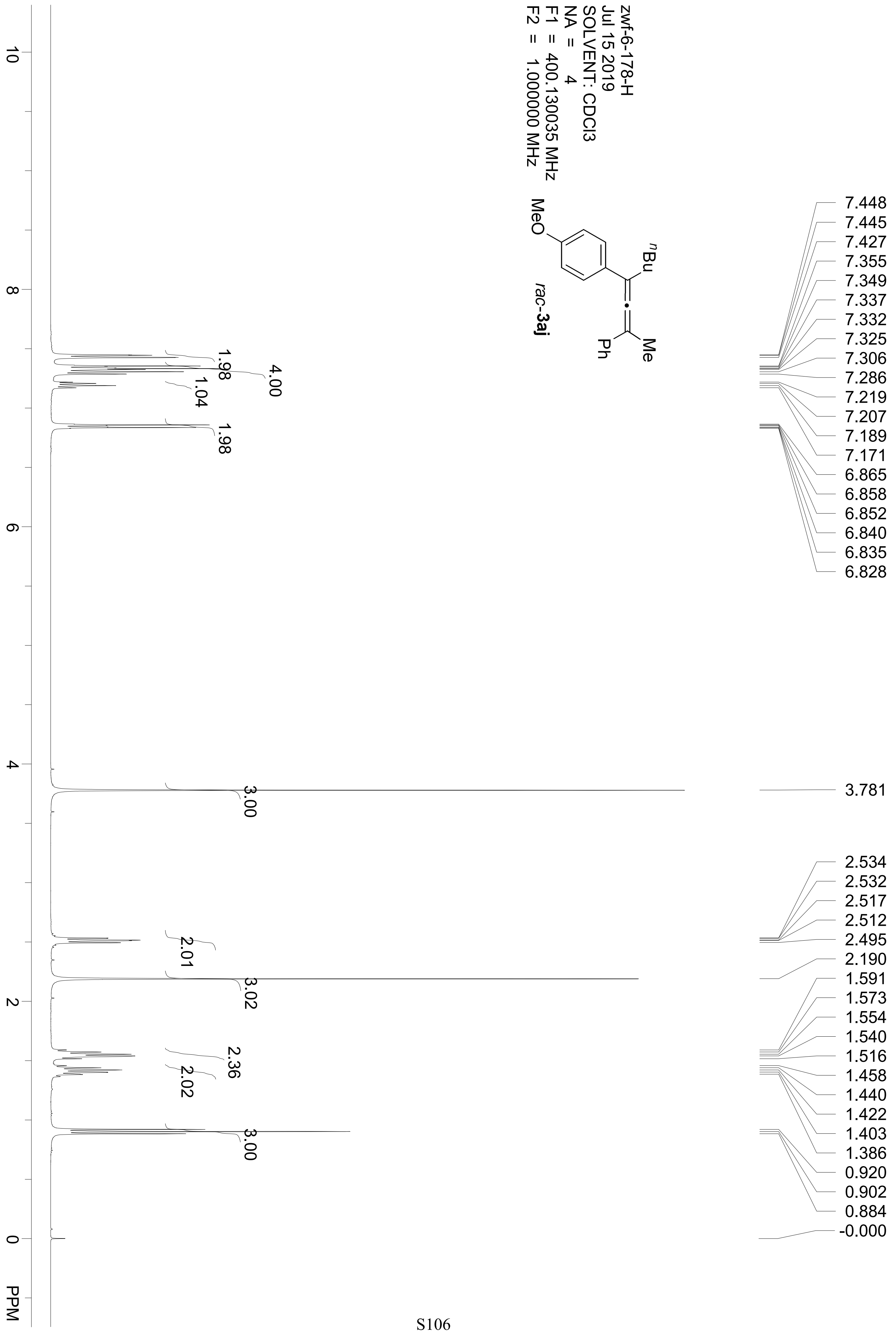

Supplementary Figure 34. ^{1}H NMR (400 MHz, $CDCl_3$) spectrum for *rac*-**3aj**

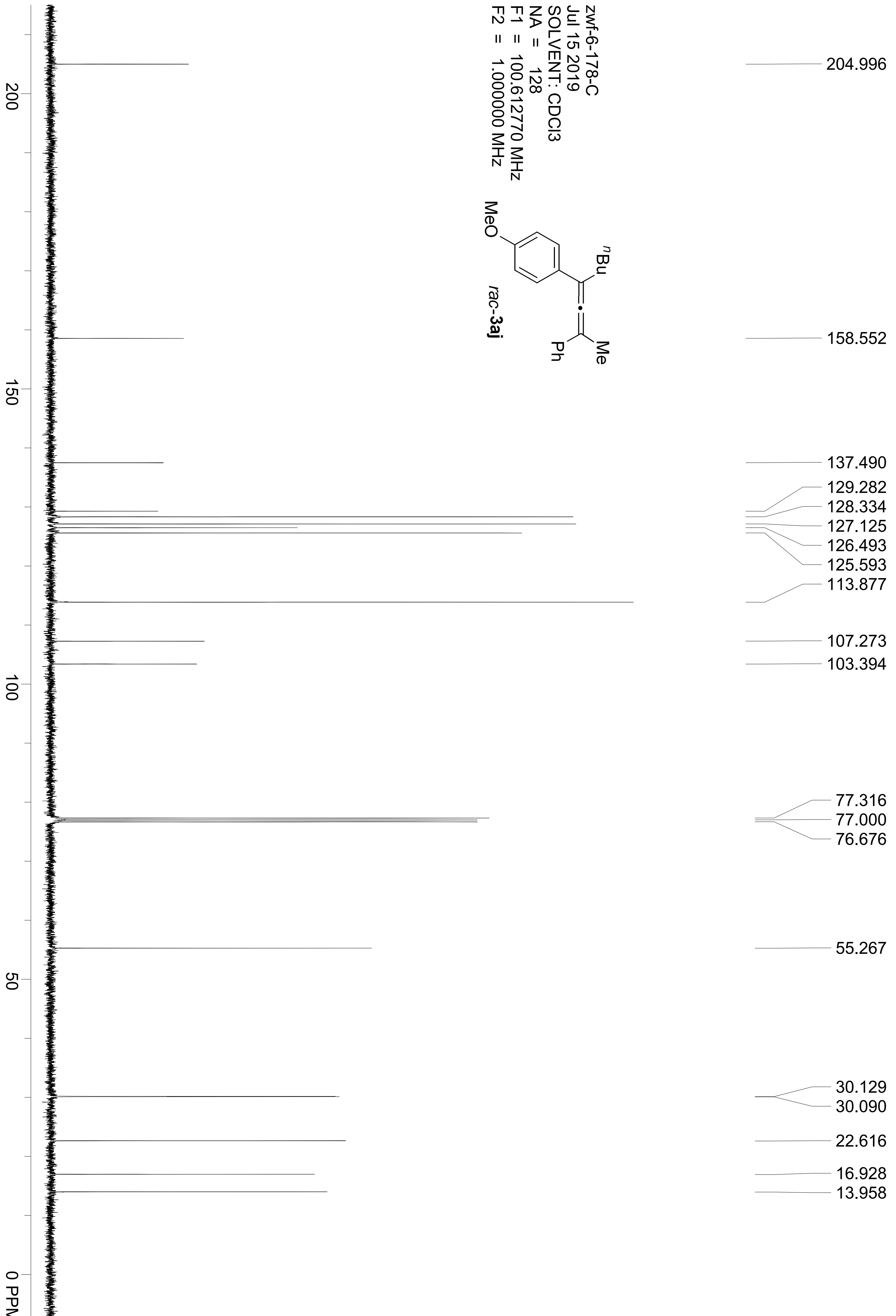

Supplementary Figure 35. ^{13}C NMR (100 MHz, $CDCl_3$) spectrum for *rac*-**3aj**

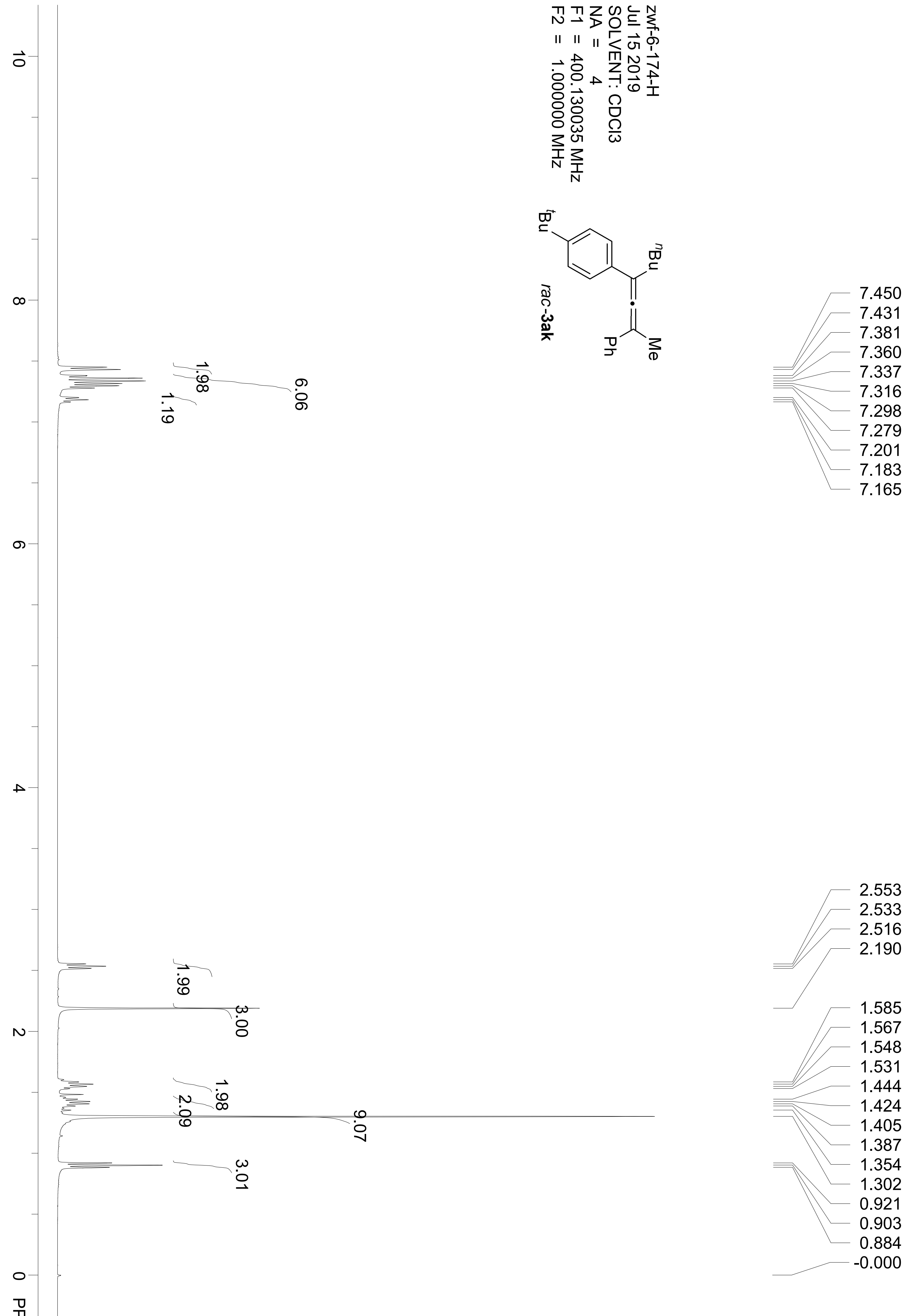

Supplementary Figure 36. ^{1}H NMR (400 MHz, $CDCl_3$) spectrum for *rac*-**3ak**

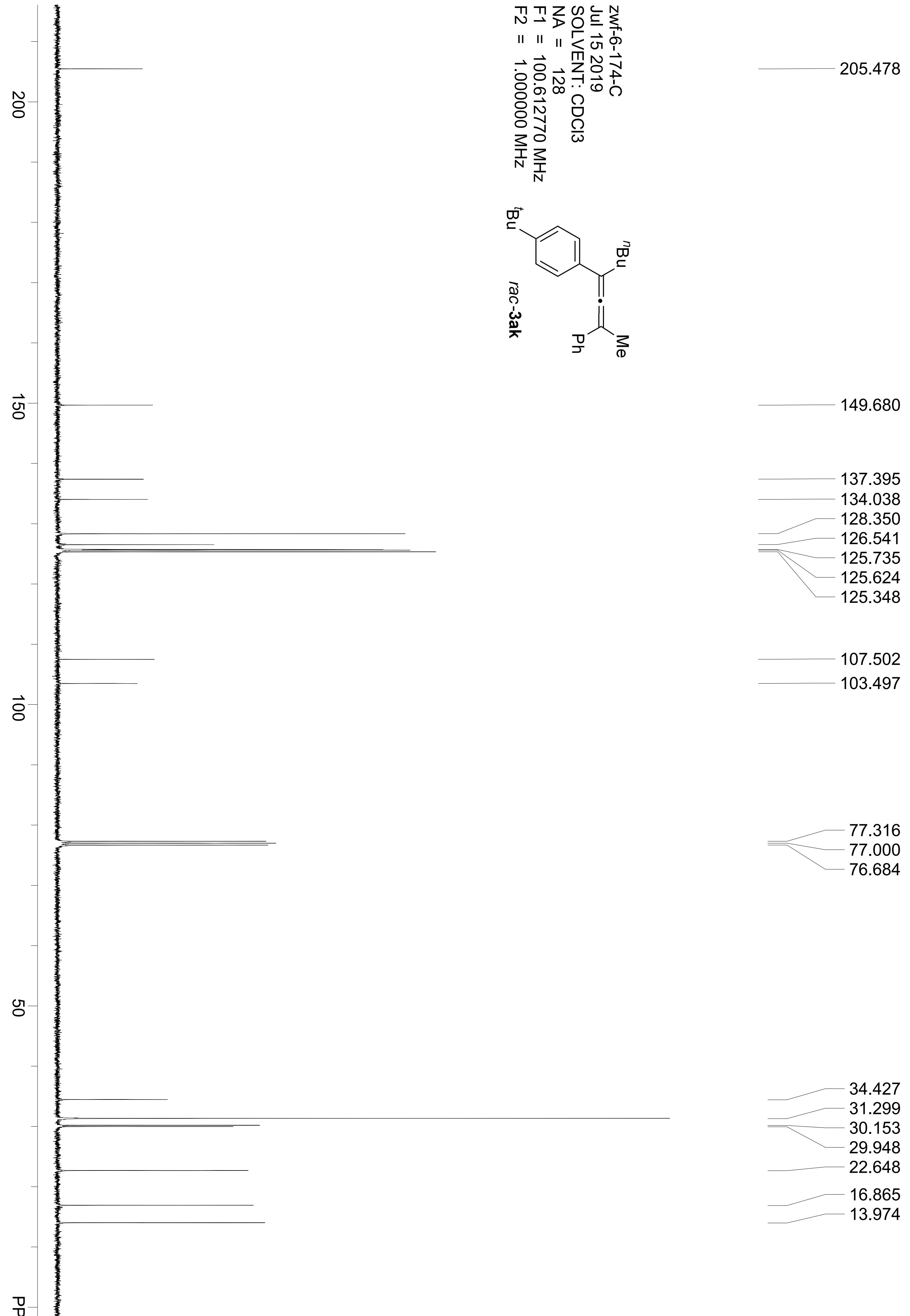

Supplementary Figure 37. ^{13}C NMR (100 MHz, $CDCl_3$) spectrum for *rac*-**3ak**

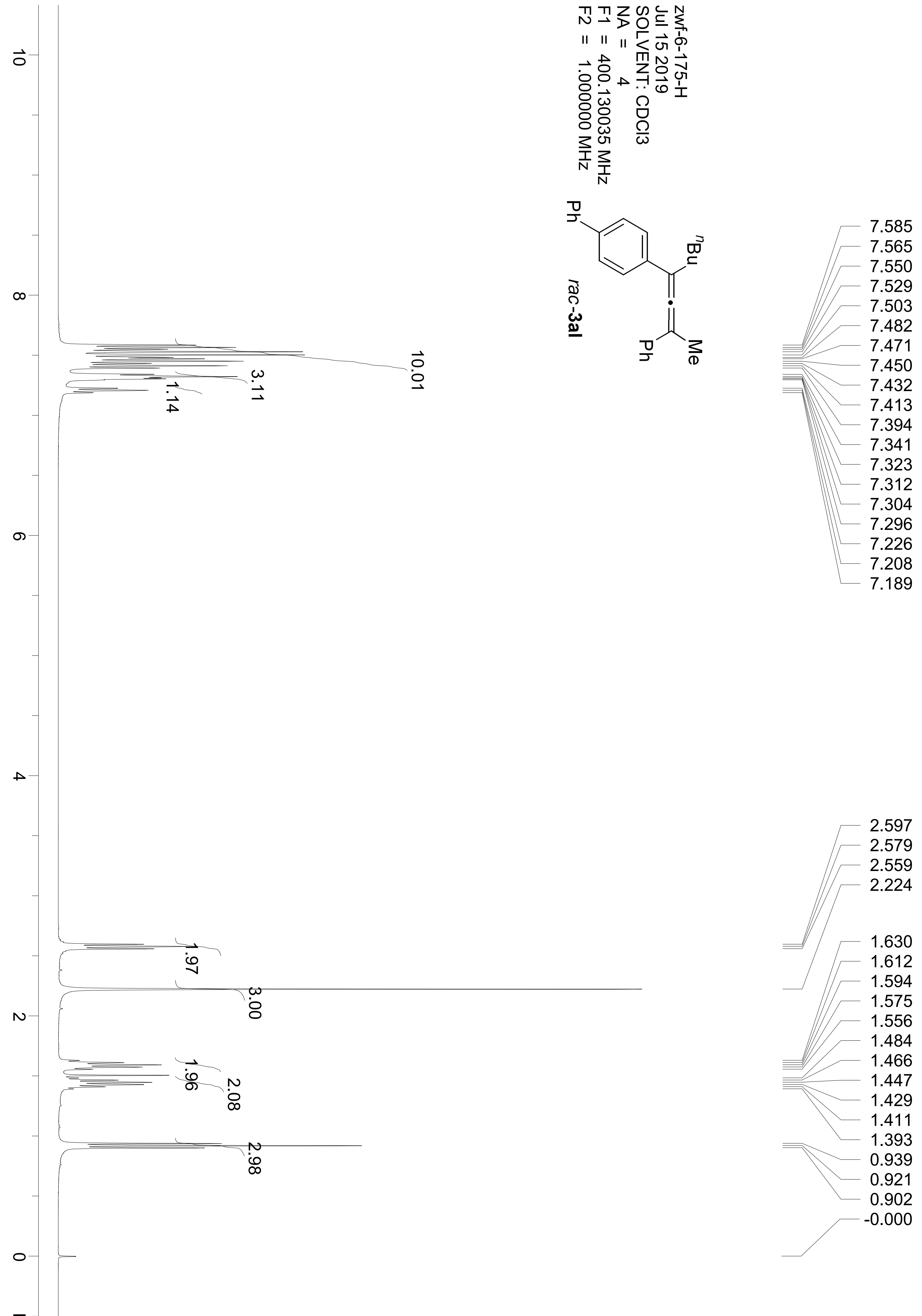

Supplementary Figure 38. ^{1}H NMR (400 MHz, $CDCl_3$) spectrum for *rac*-**3al**

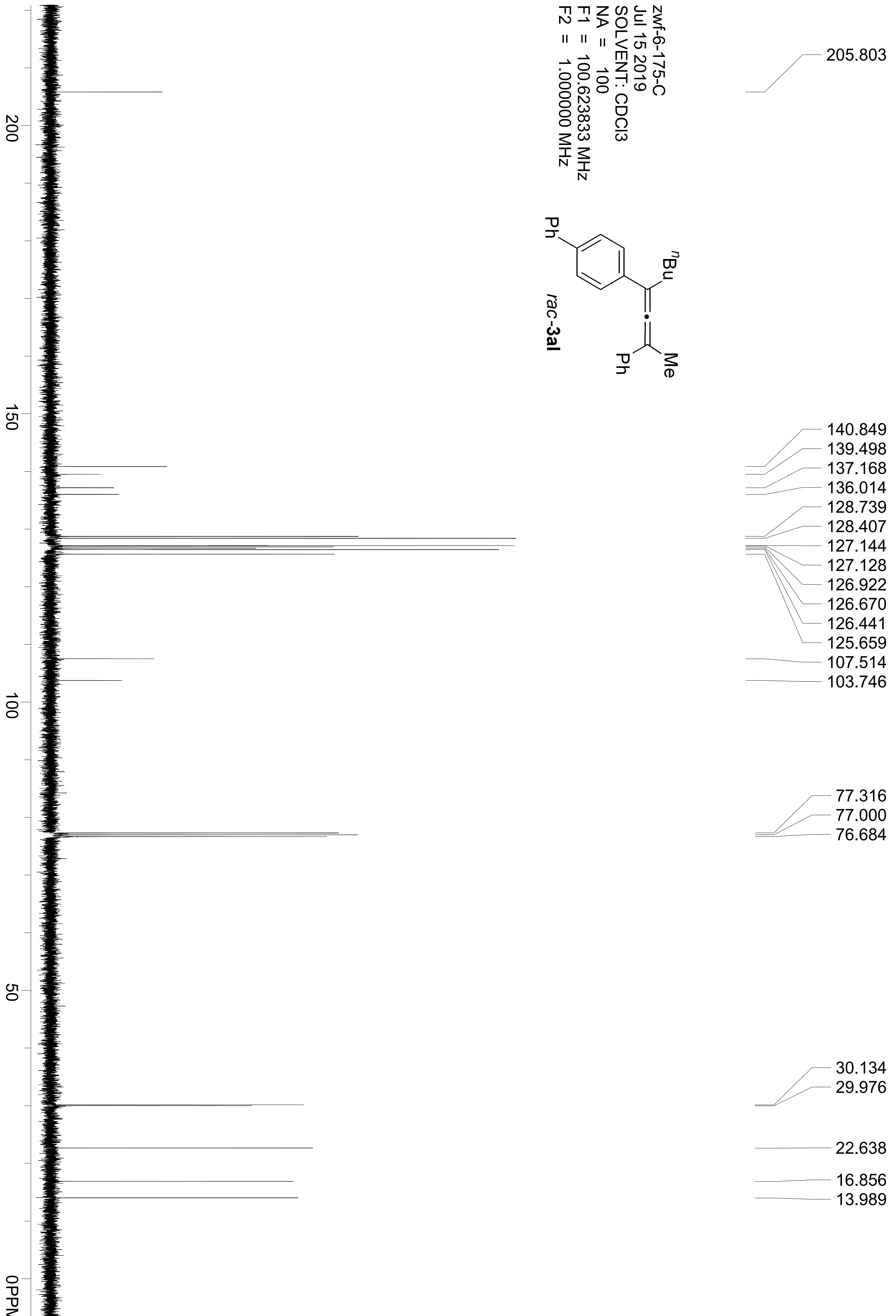

Supplementary Figure 39. ^{13}C NMR (100 MHz, $CDCl_3$) spectrum for *rac*-**3al**

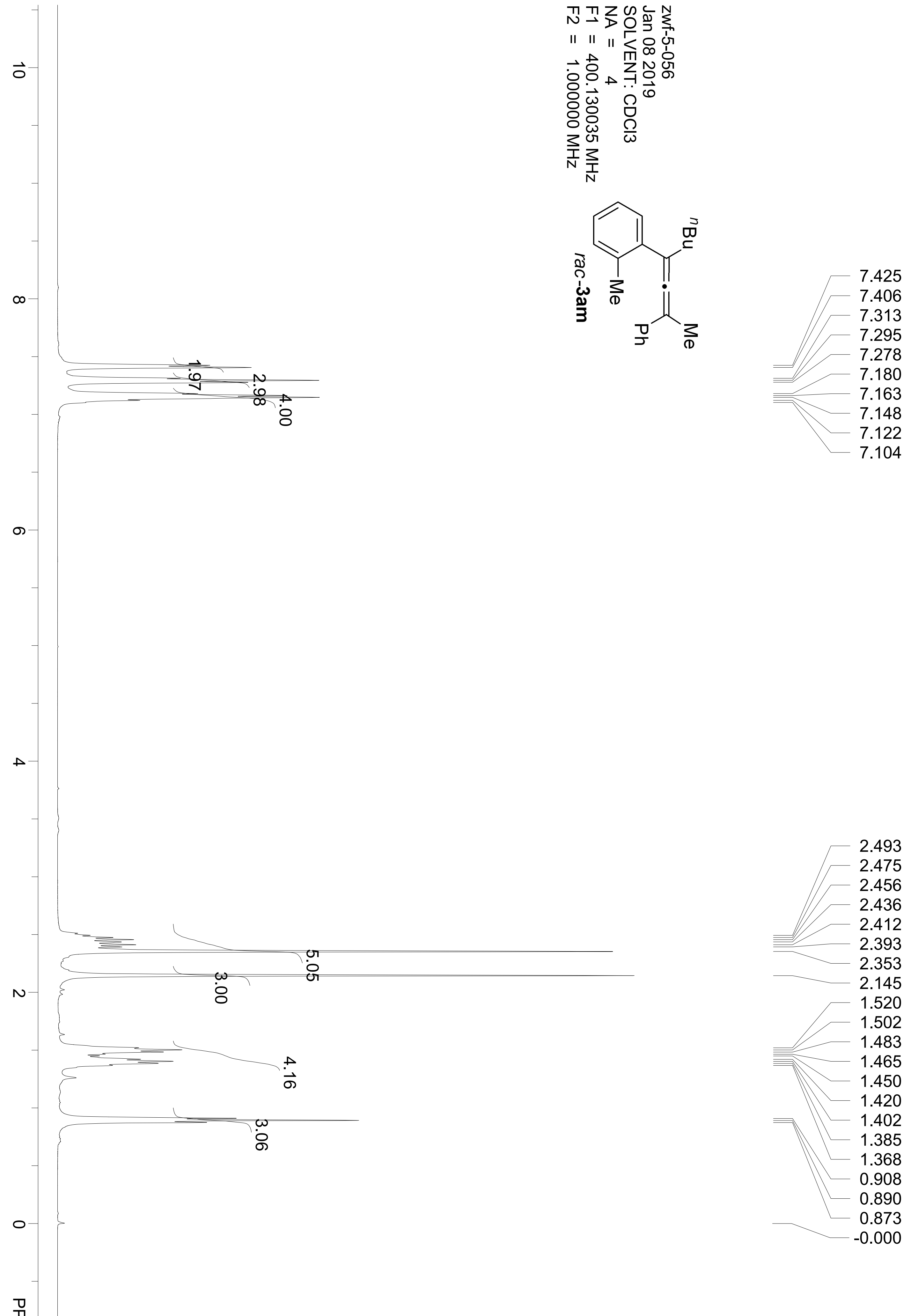

Supplementary Figure 40. ^{1}H NMR (400 MHz, $CDCl_3$) spectrum for *rac*-**3am**

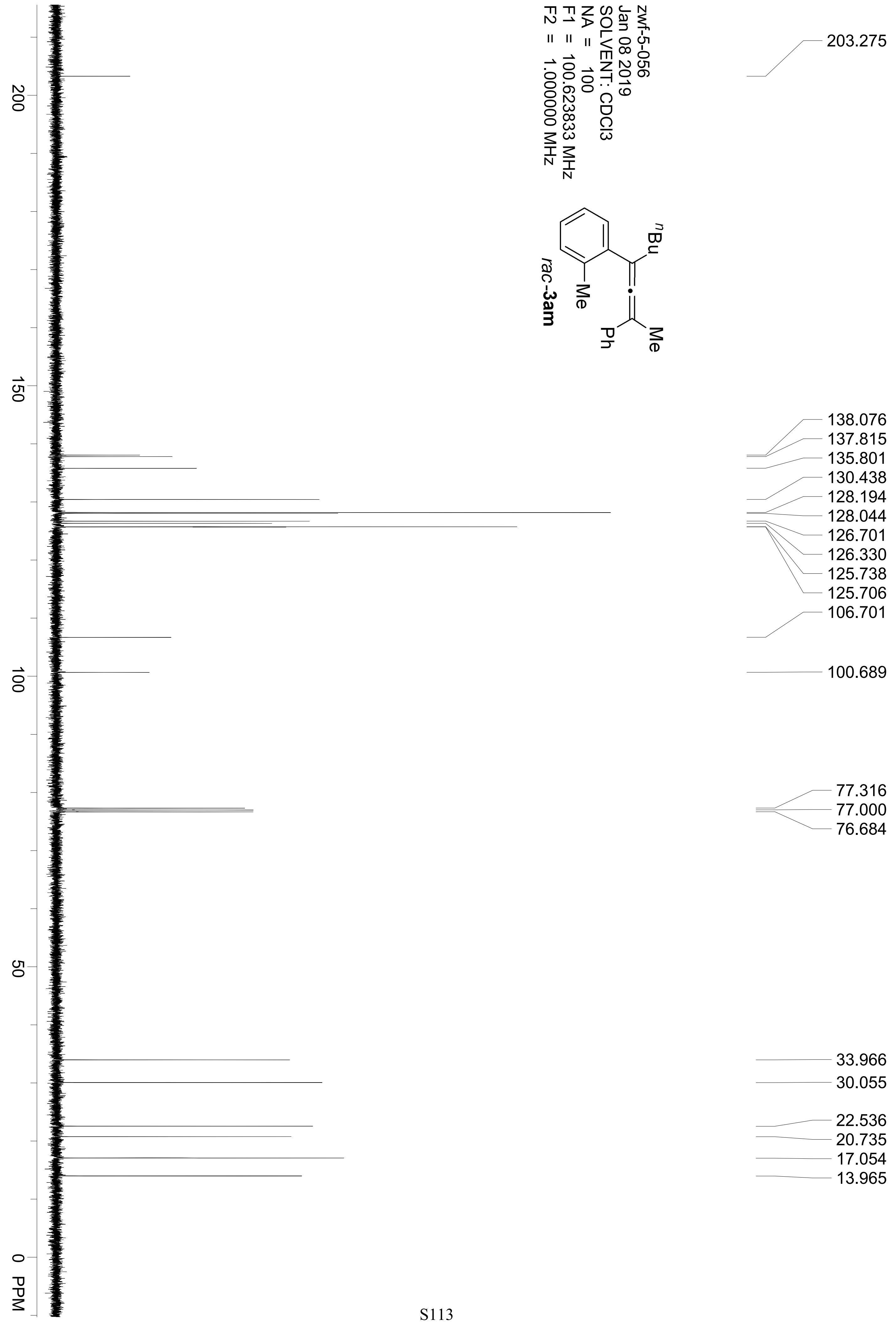

Supplementary Figure 41. ^{13}C NMR (100 MHz, $CDCl_3$) spectrum for *rac*-**3am**

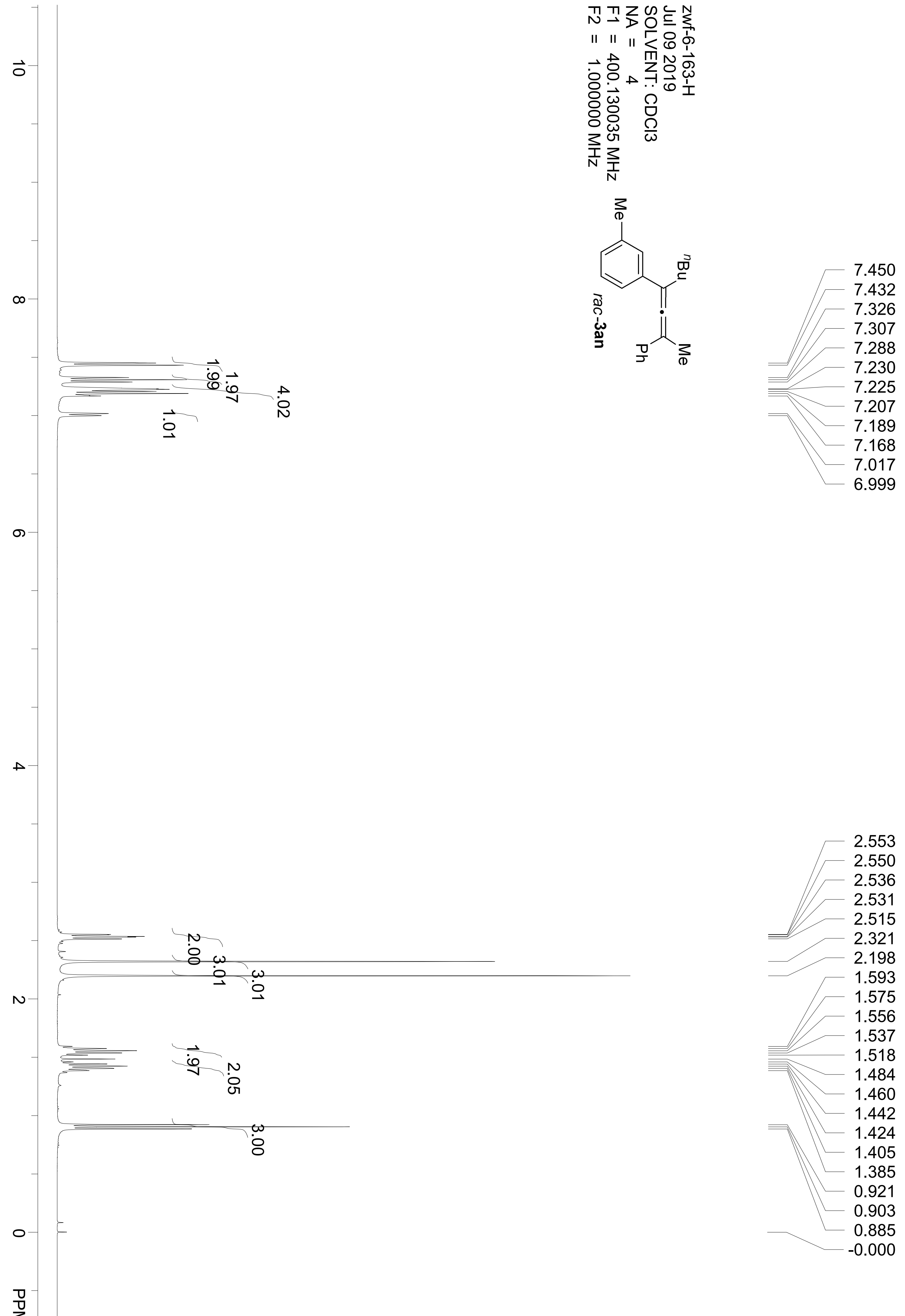

Supplementary Figure 42. ^{1}H NMR (400 MHz, $CDCl_3$) spectrum for *rac*-**3an**

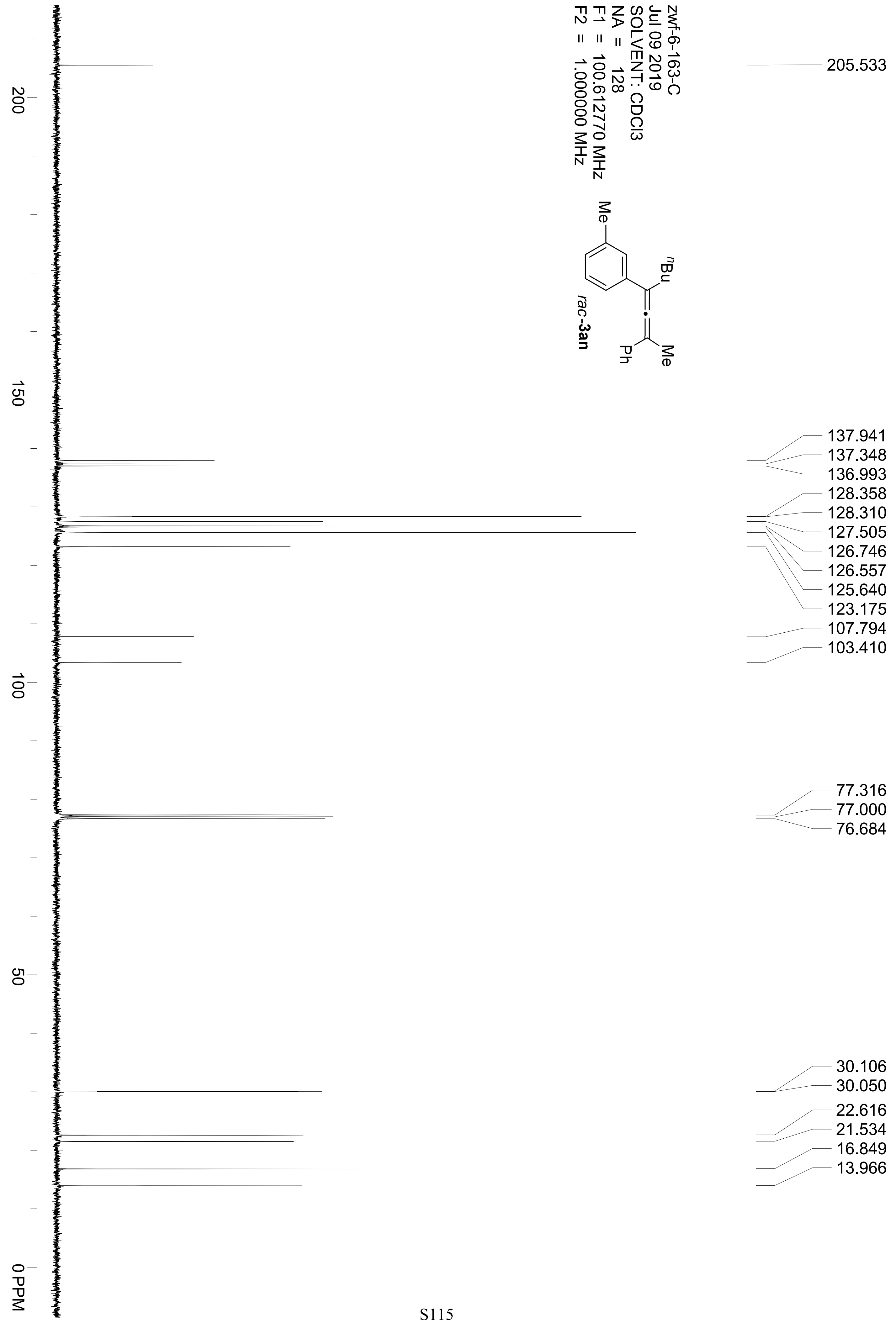

Supplementary Figure 43. ^{13}C NMR (100 MHz, $CDCl_3$) spectrum for *rac*-**3an**

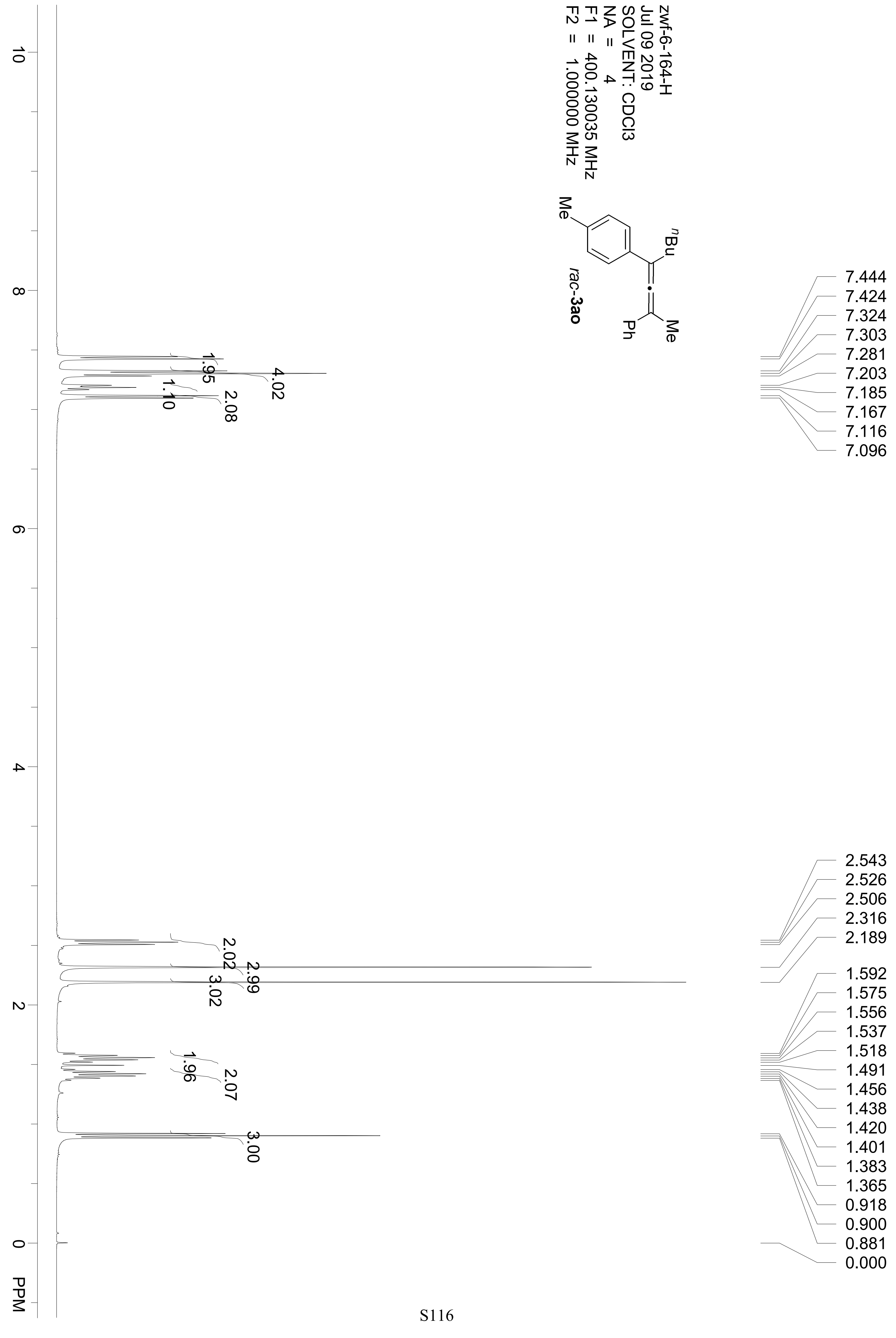

Supplementary Figure 44. ^{1}H NMR (400 MHz, $CDCl_3$) spectrum for *rac*-**3ao**

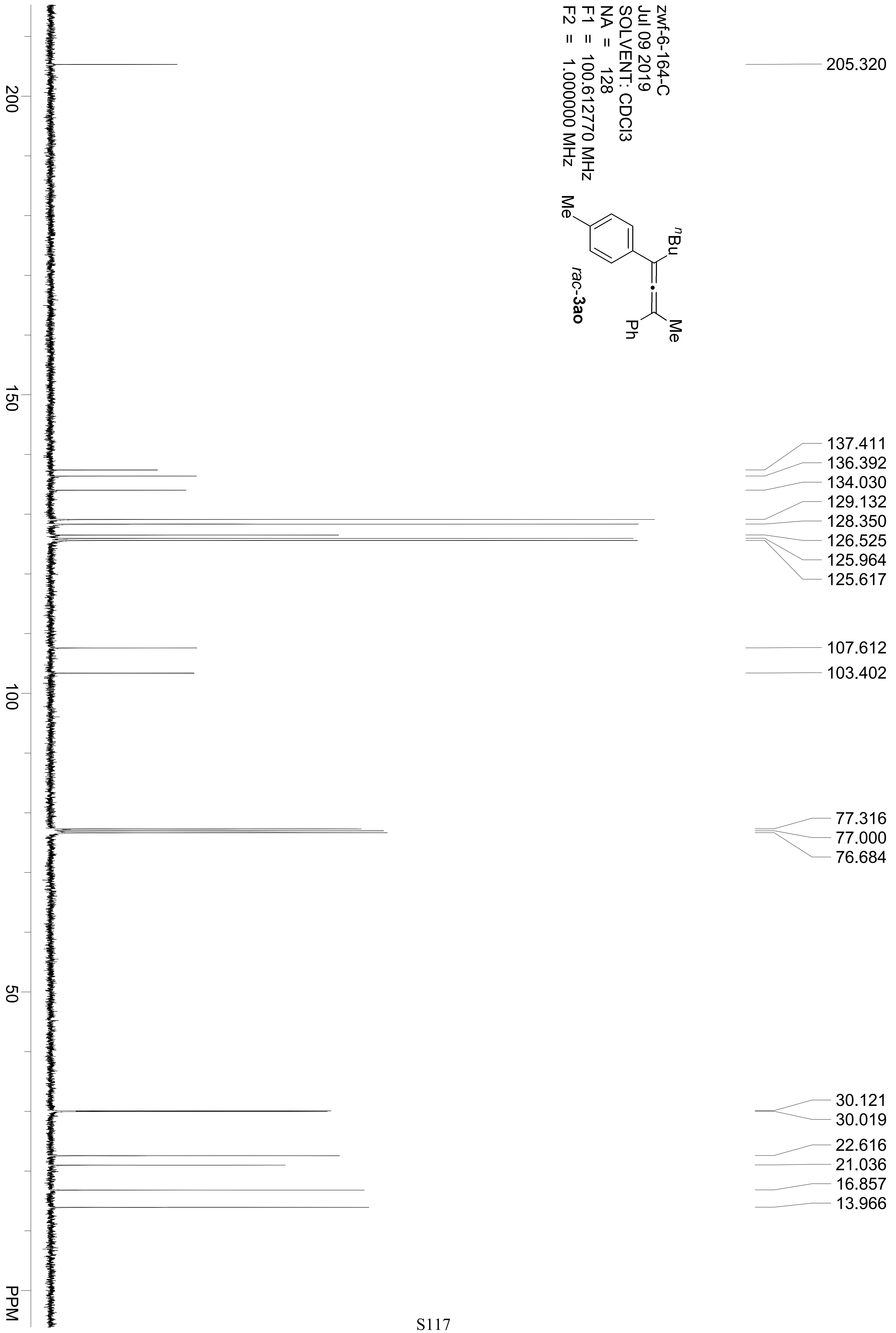

Supplementary Figure 45. ^{13}C NMR (100 MHz, $CDCl_3$) spectrum for *rac*-**3ao**

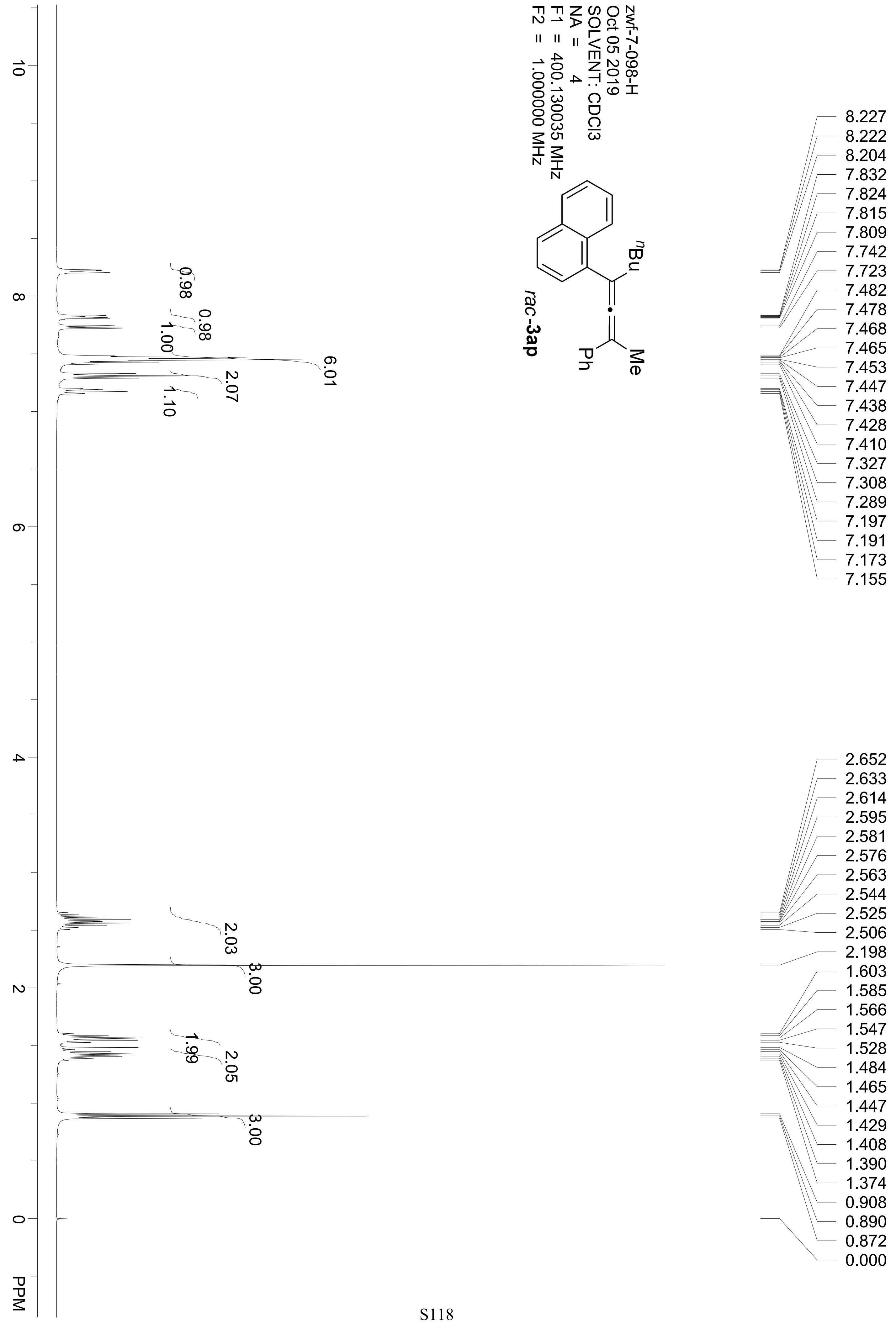

Supplementary Figure 46. ^{1}H NMR (400 MHz, $CDCl_3$) spectrum for *rac*-**3ap**

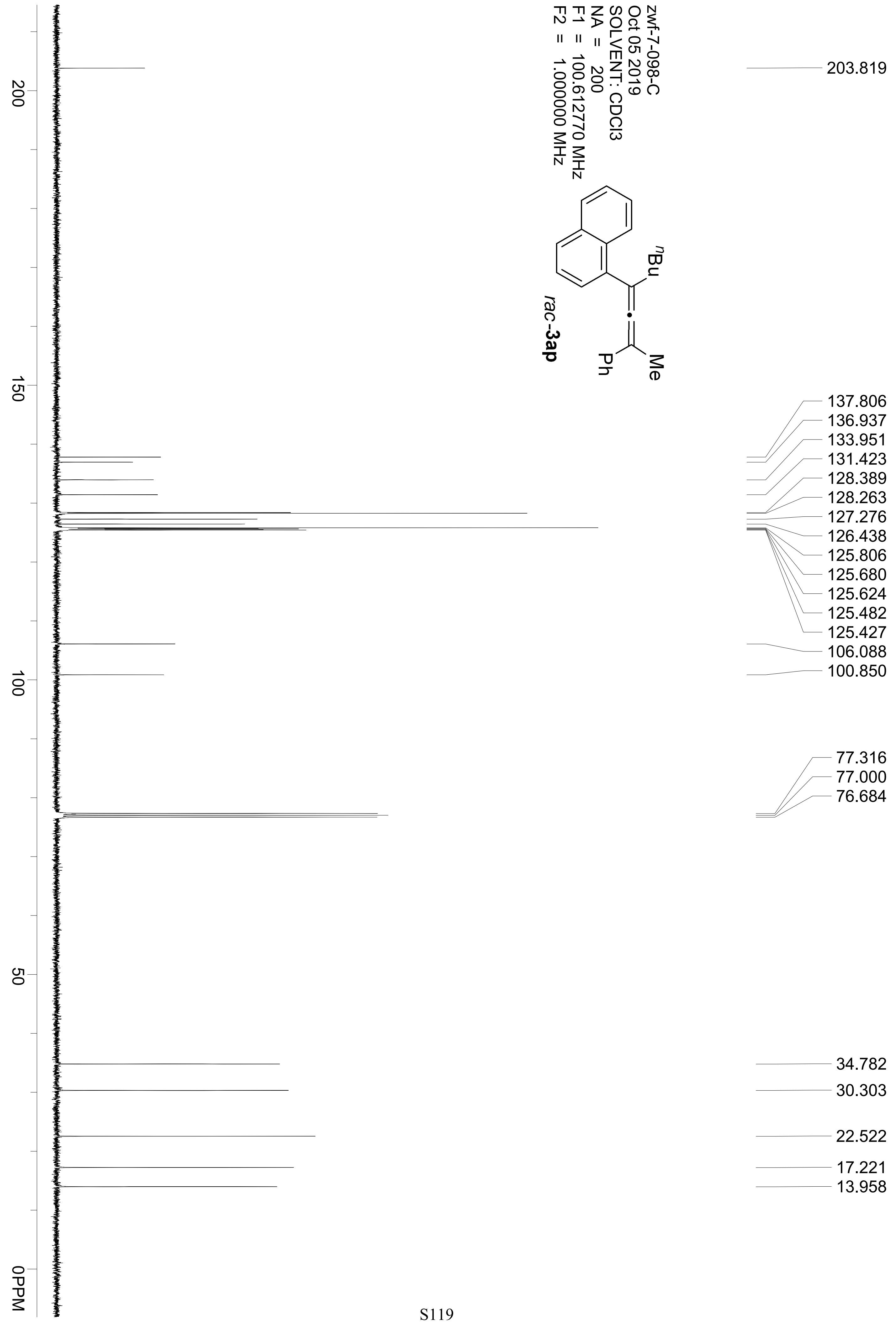

Supplementary Figure 47. ^{13}C NMR (100 MHz, $CDCl_3$) spectrum for *rac*-**3ap**

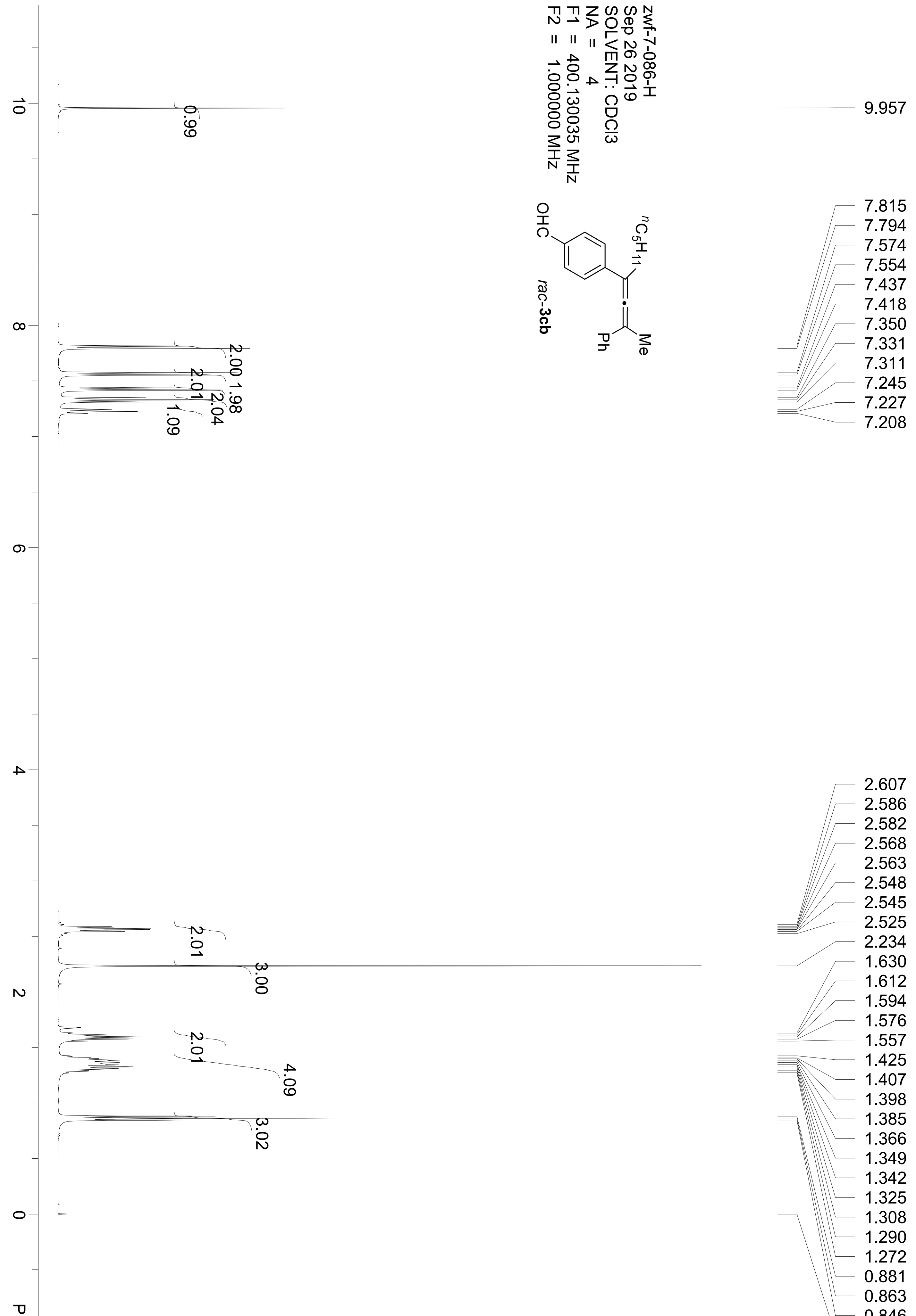

Supplementary Figure 48. ^{1}H NMR (400 MHz, $CDCl_3$) spectrum for *rac*-**3cb**

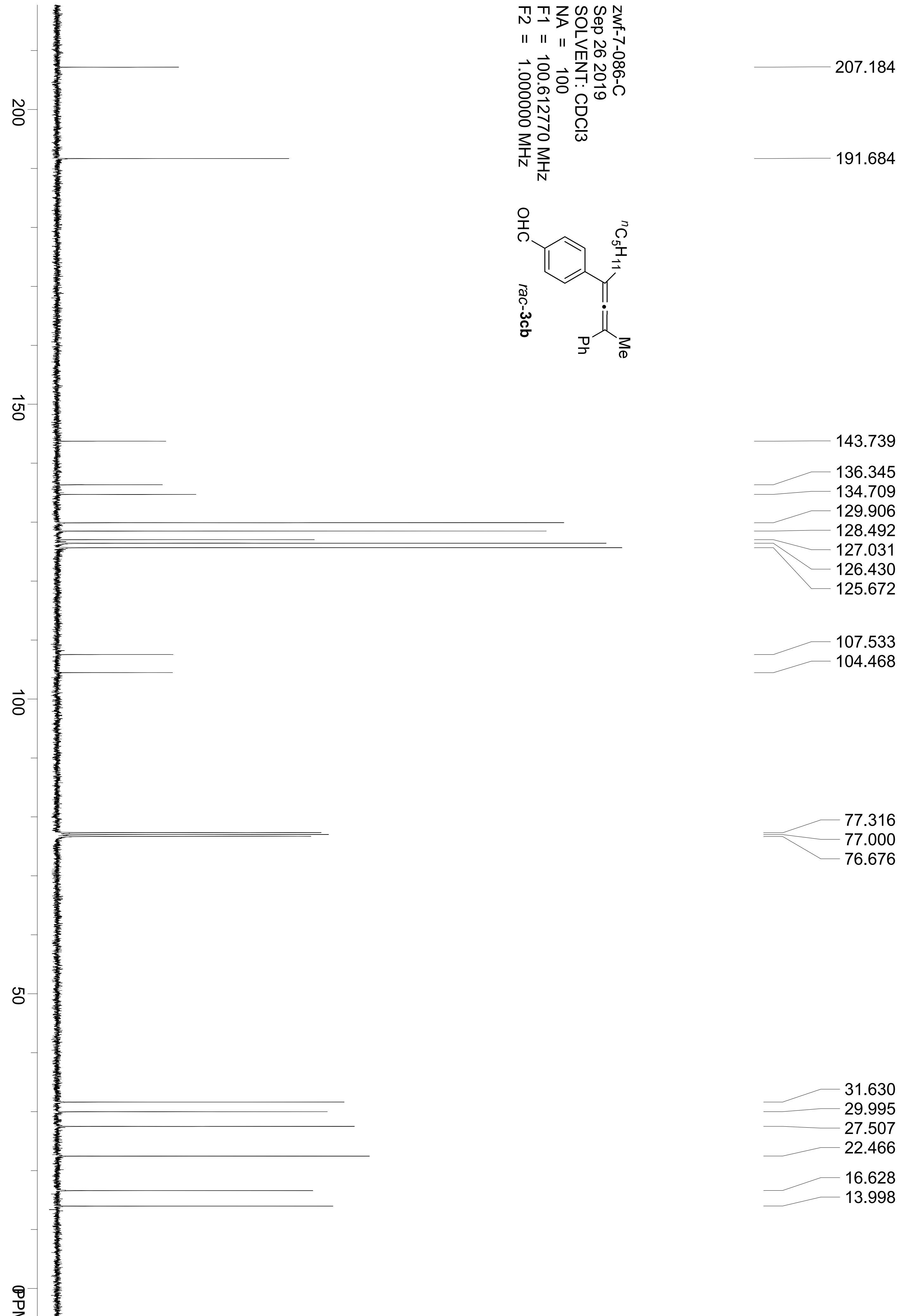

Supplementary Figure 49. ^{13}C NMR (100 MHz, $CDCl_3$) spectrum for *rac*-**3cb**

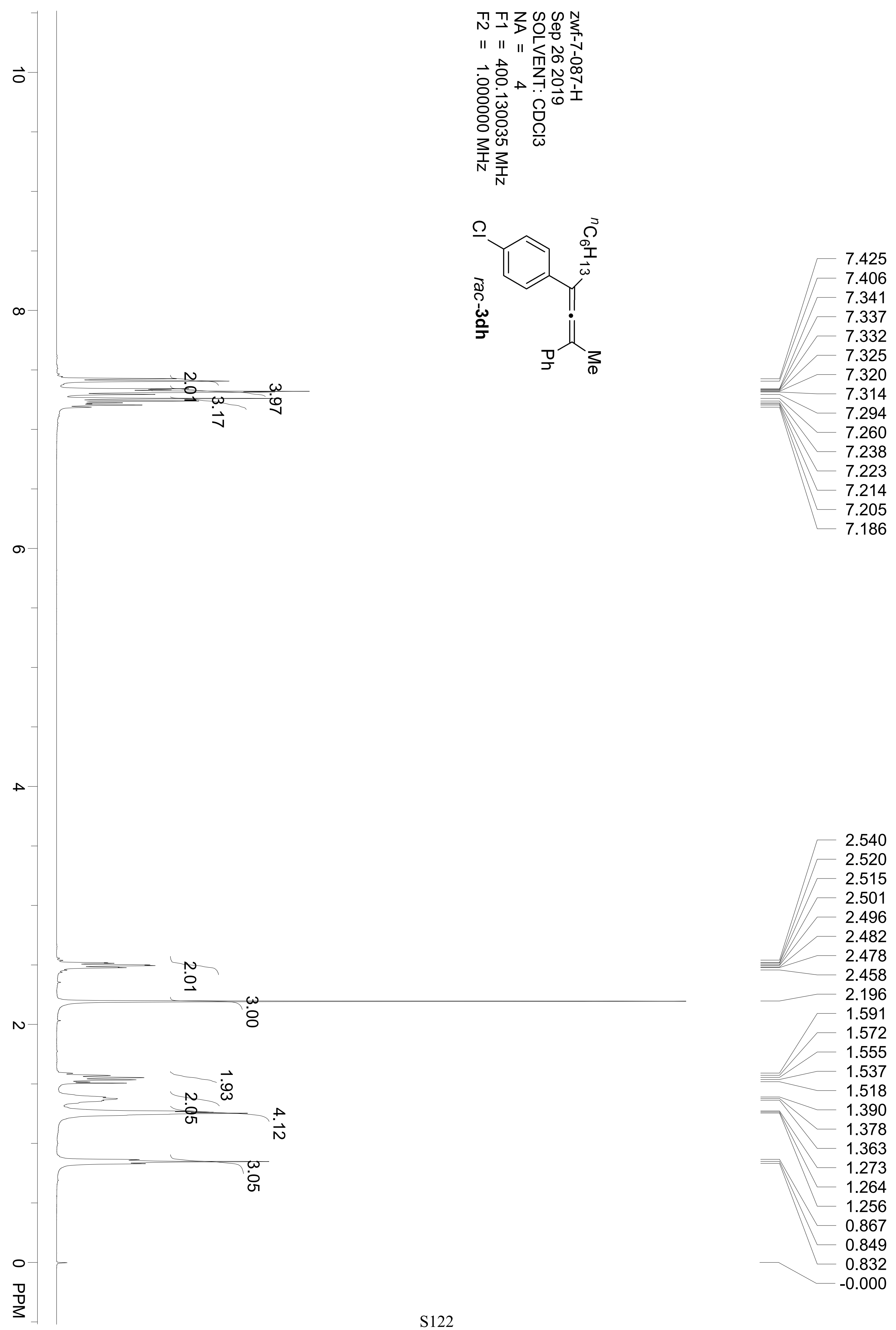

Supplementary Figure 50. ^{1}H NMR (400 MHz, $CDCl_3$) spectrum for *rac*-**3dh**

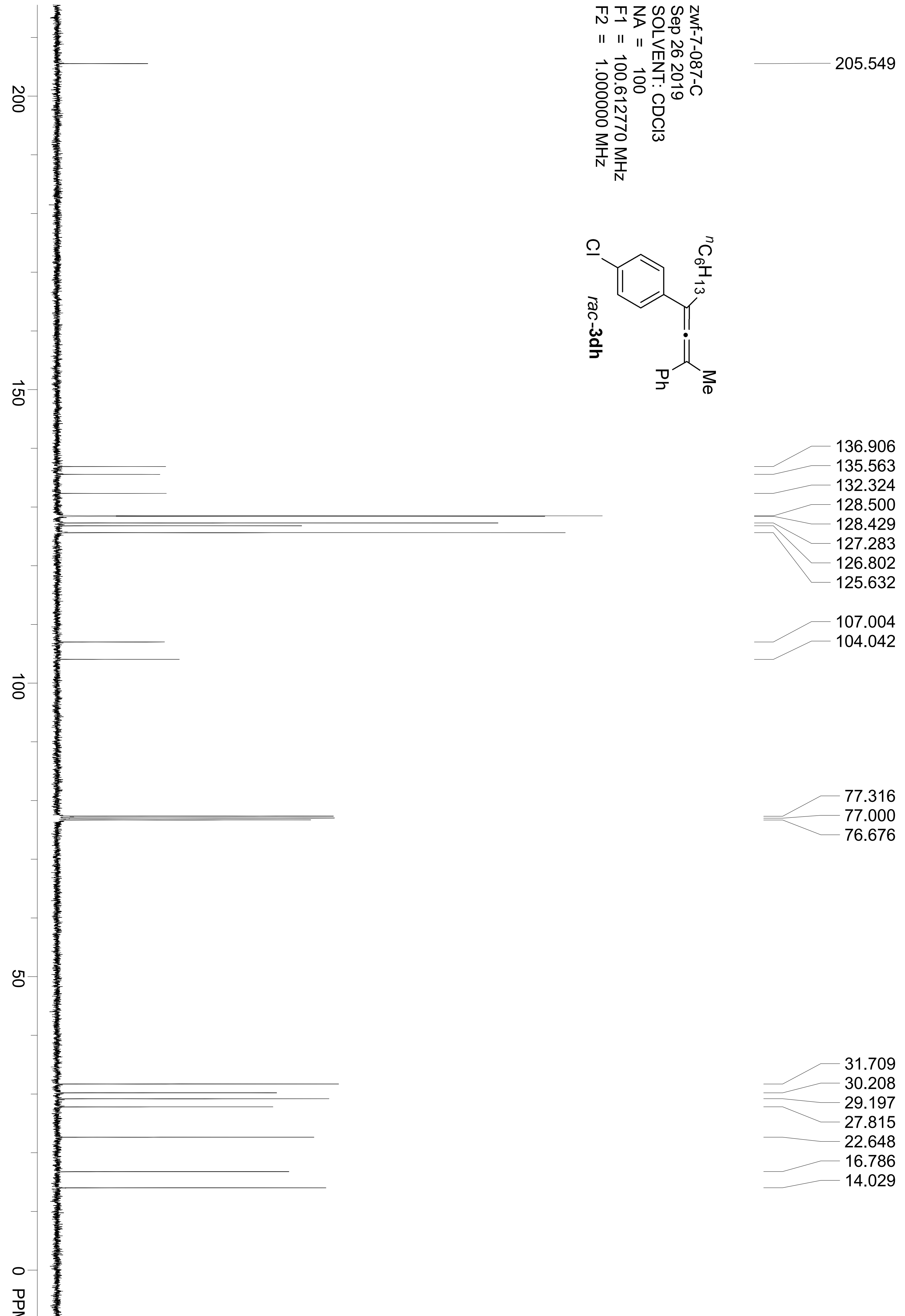

Supplementary Figure 51. ^{13}C NMR (100 MHz, $CDCl_3$) spectrum for *rac*-**3dh**

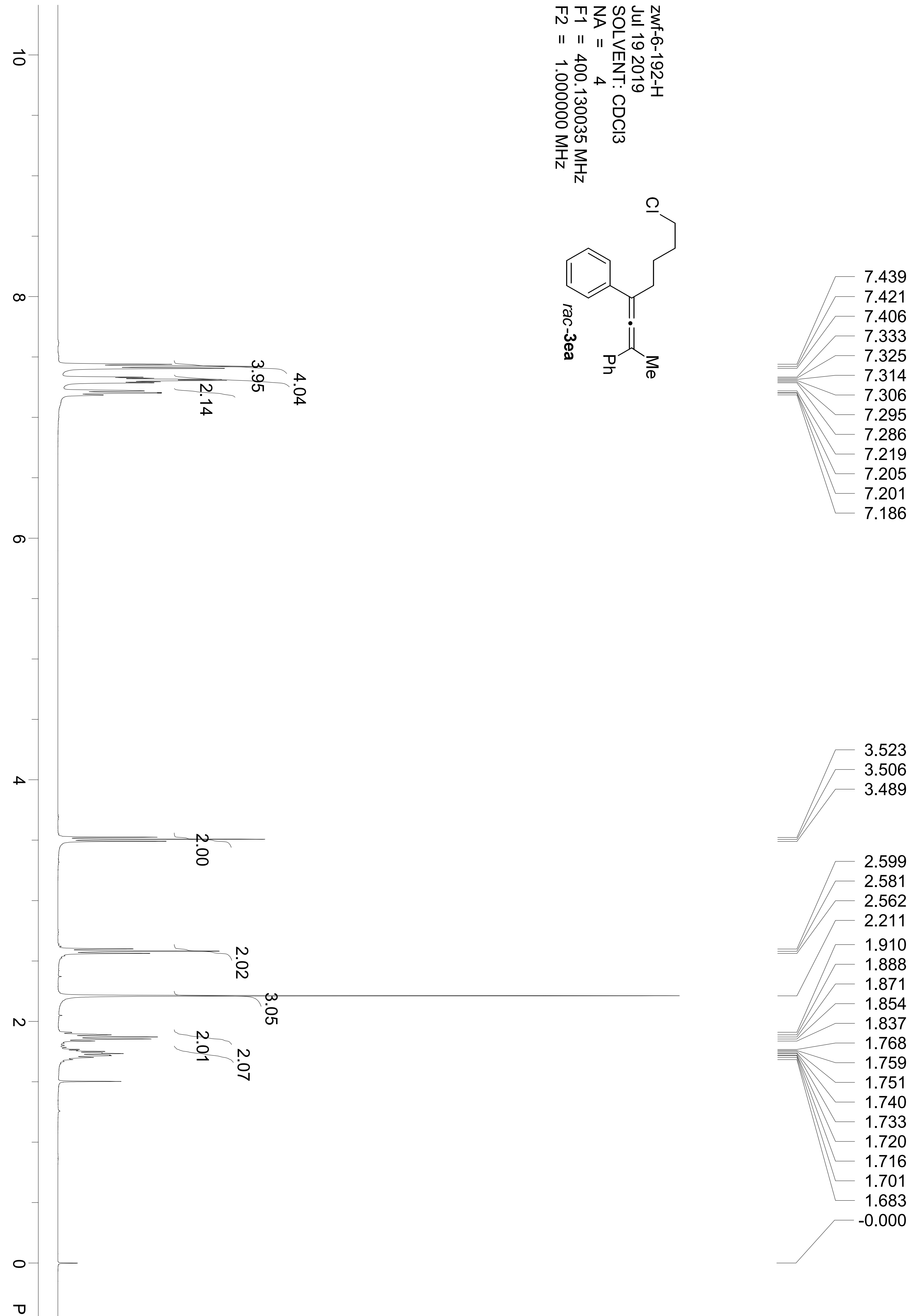

Supplementary Figure 52. ^{1}H NMR (400 MHz, $CDCl_3$) spectrum for *rac*-**3ea**

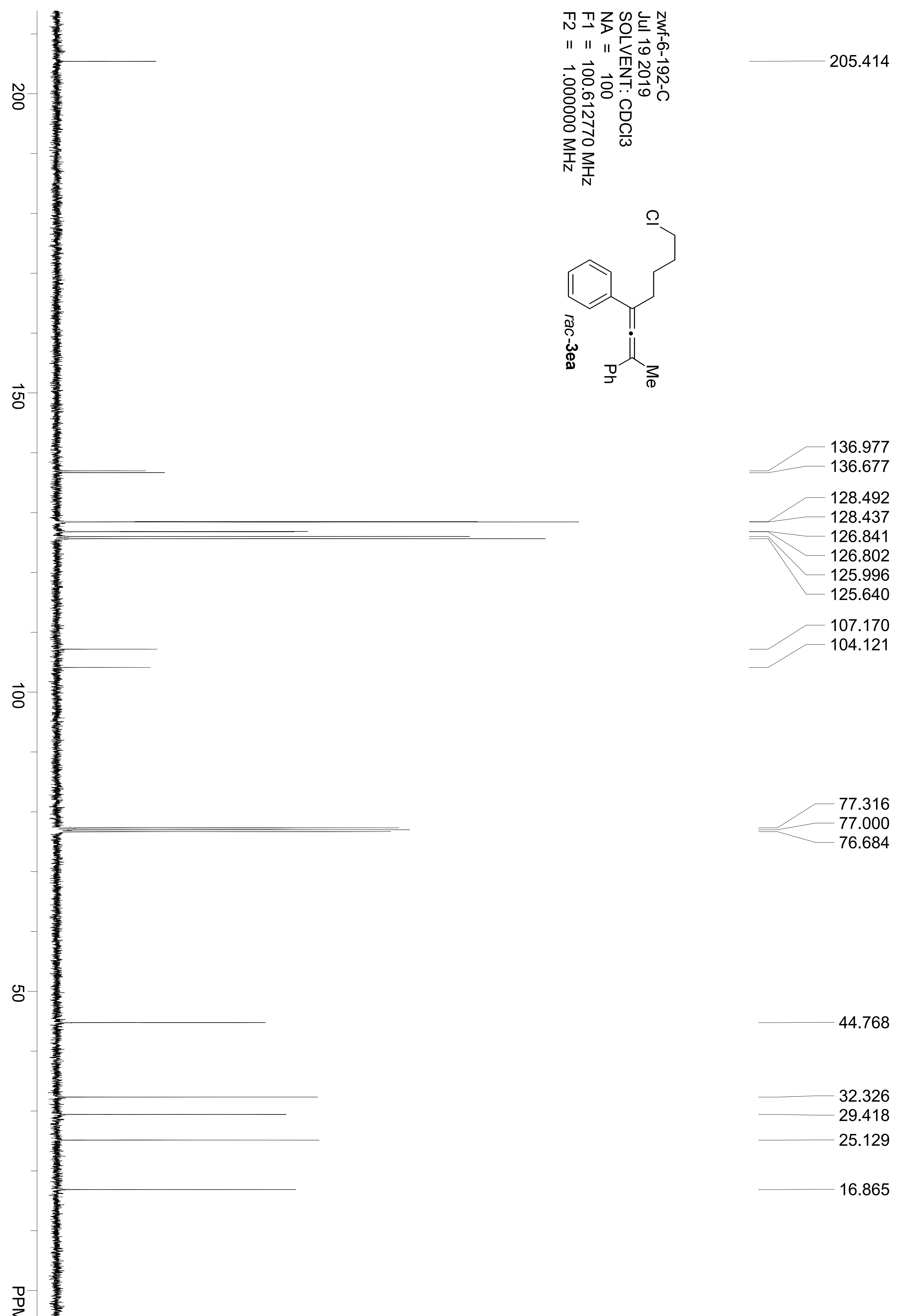

Supplementary Figure 53. ^{13}C NMR (100 MHz, $CDCl_3$) spectrum for *rac*-**3ea**

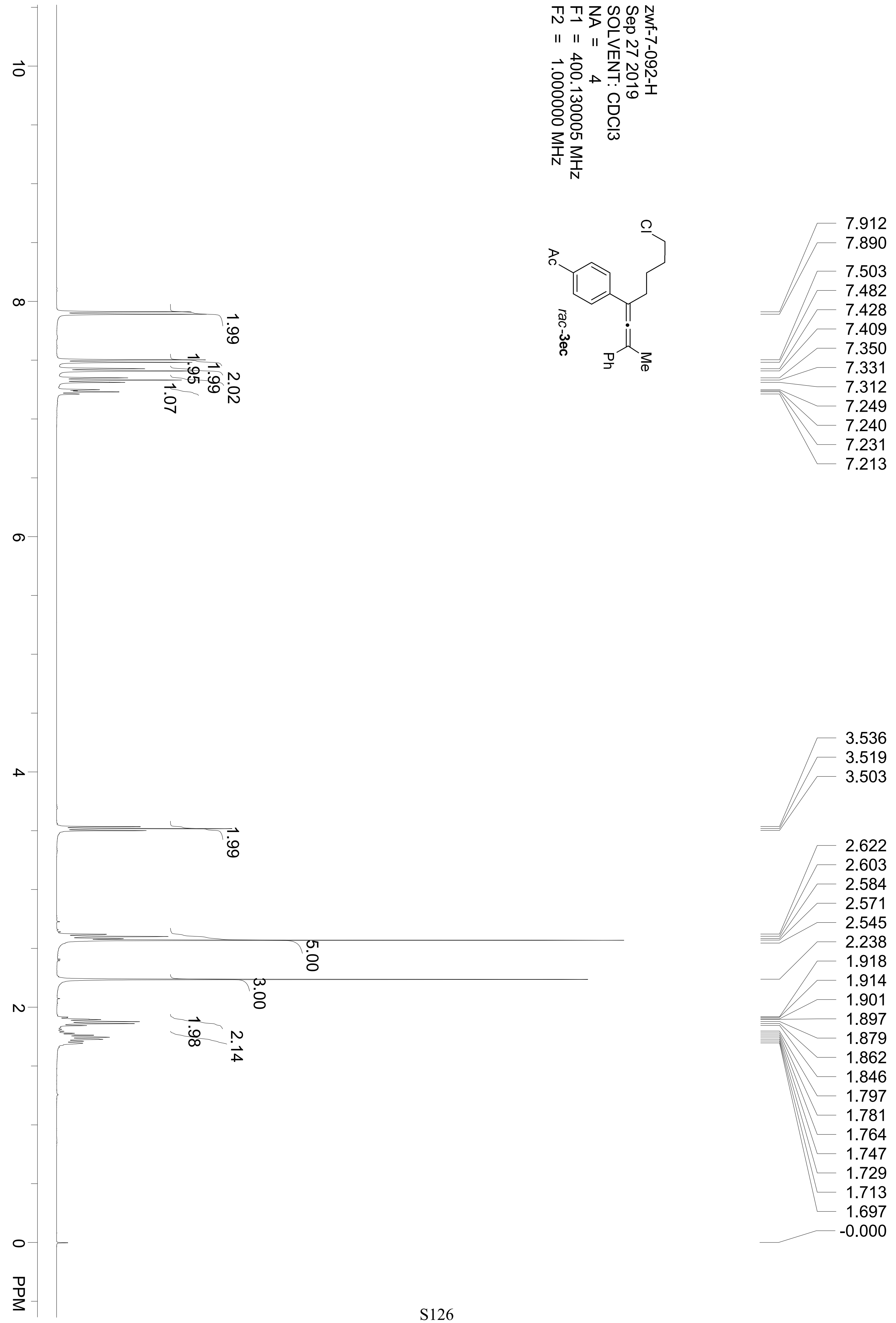

Supplementary Figure 54. ^{1}H NMR (400 MHz, $CDCl_3$) spectrum for *rac*-**3ec**

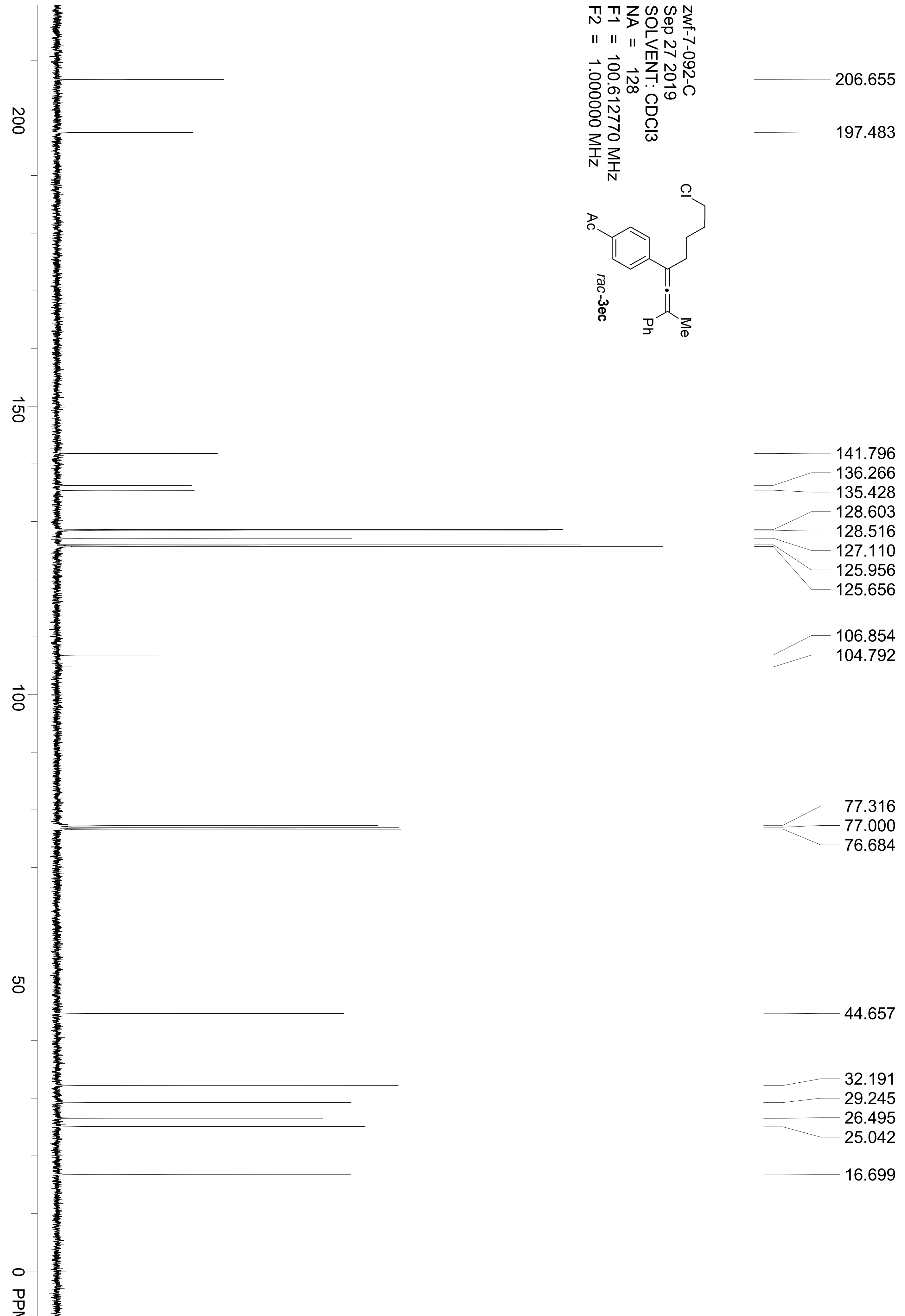

Supplementary Figure 55. ^{13}C NMR (100 MHz, $CDCl_3$) spectrum for *rac*-**3ec**

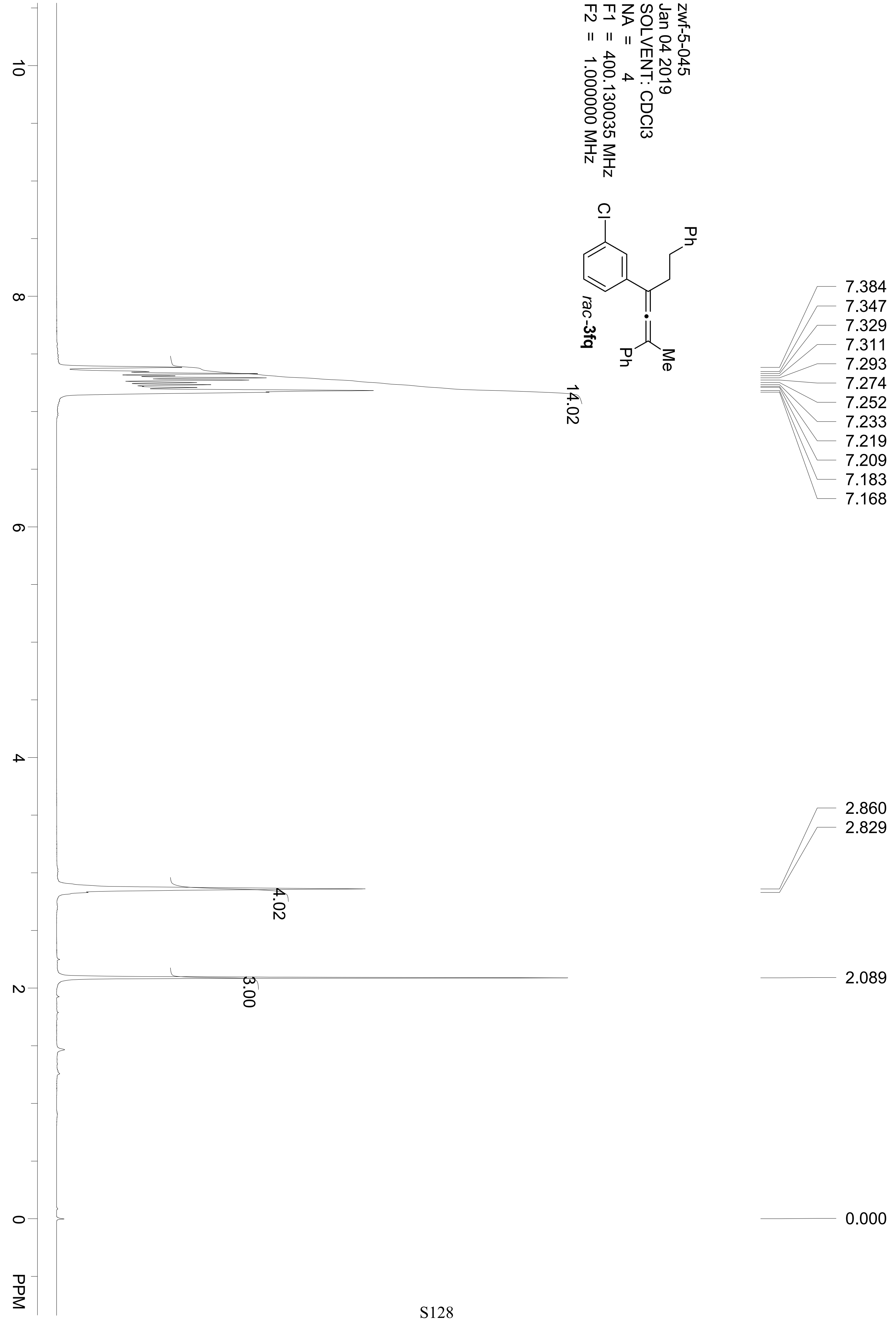

Supplementary Figure 56. ^{1}H NMR (400 MHz, $CDCl_3$) spectrum for *rac*-**3fq**

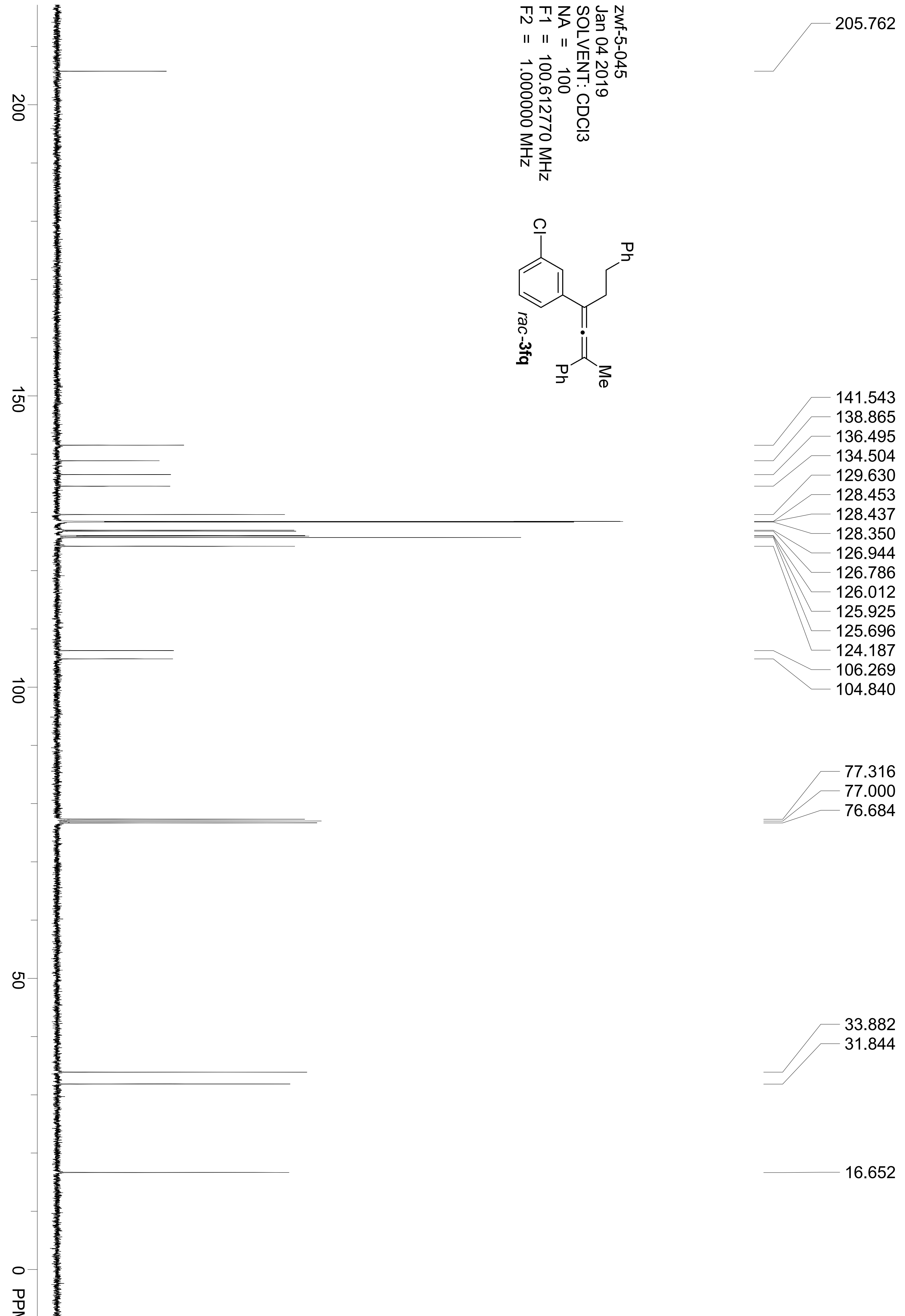

Supplementary Figure 57. ^{13}C NMR (100 MHz, $CDCl_3$) spectrum for *rac*-**3fq**

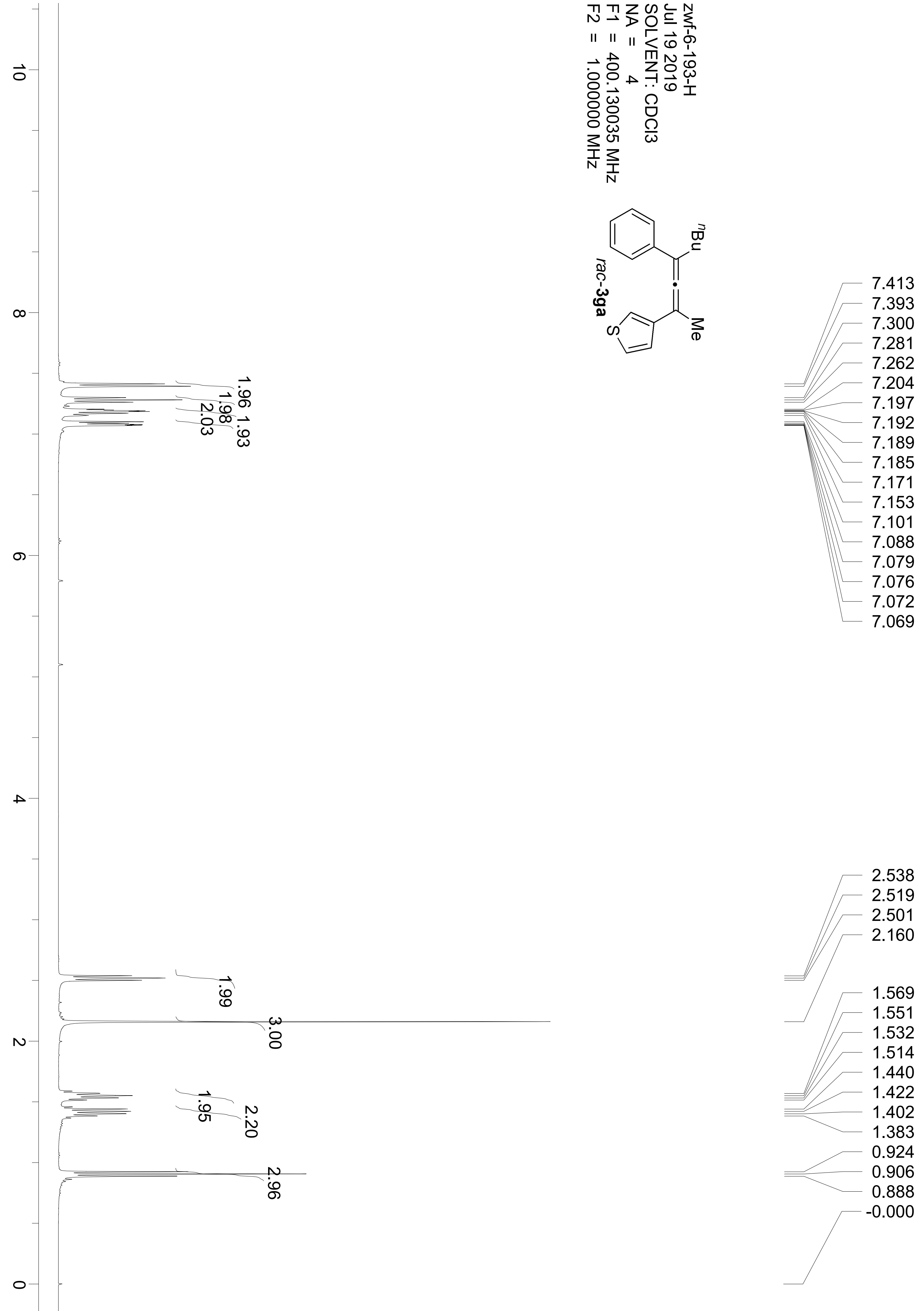

Supplementary Figure 58. ^{1}H NMR (400 MHz, $CDCl_3$) spectrum for *rac*-**3ga**

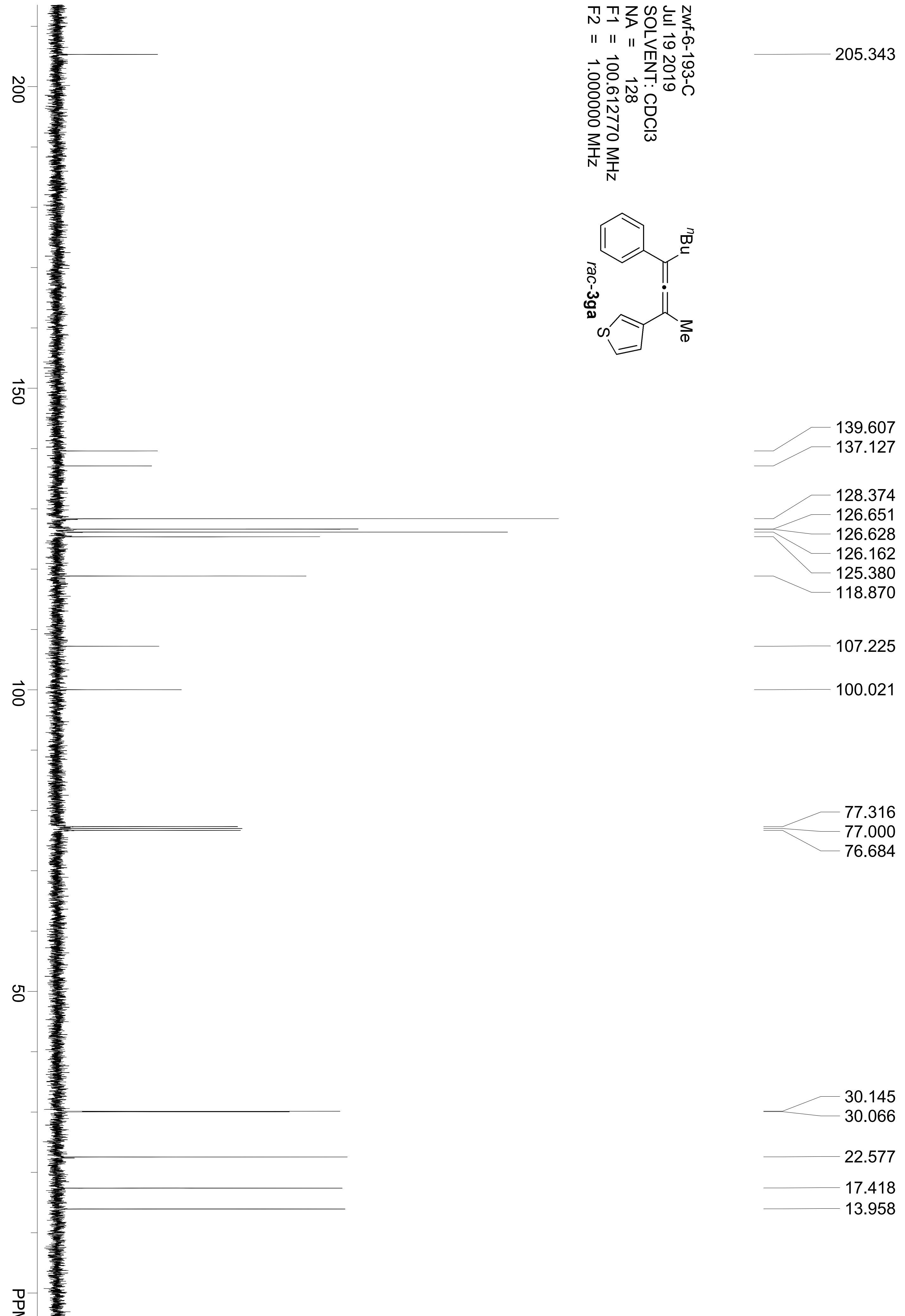

Supplementary Figure 59. ^{13}C NMR (100 MHz, $CDCl_3$) spectrum for *rac*-**3ga**

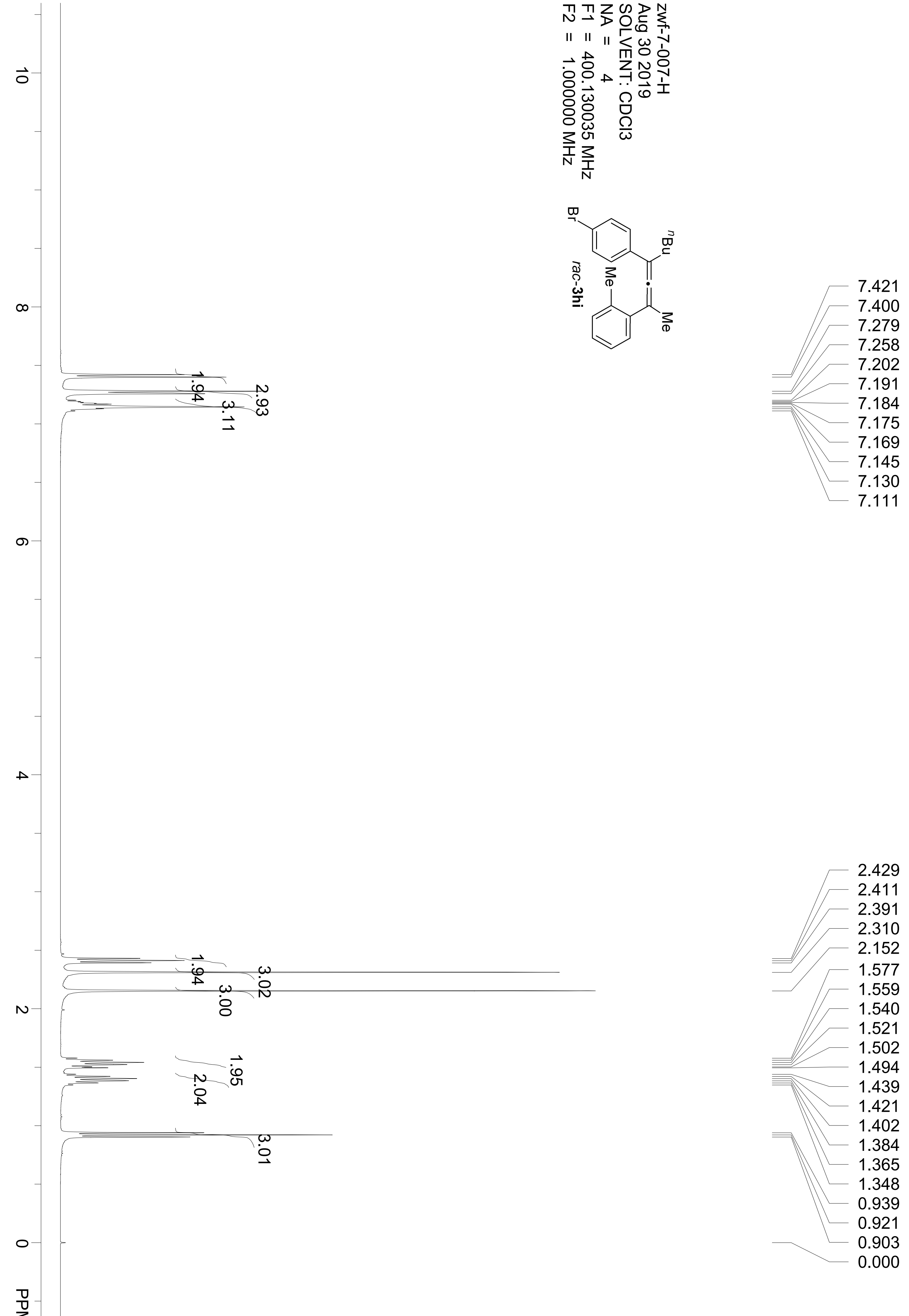

Supplementary Figure 60. ^{1}H NMR (400 MHz, $CDCl_3$) spectrum for *rac*-**3hi**

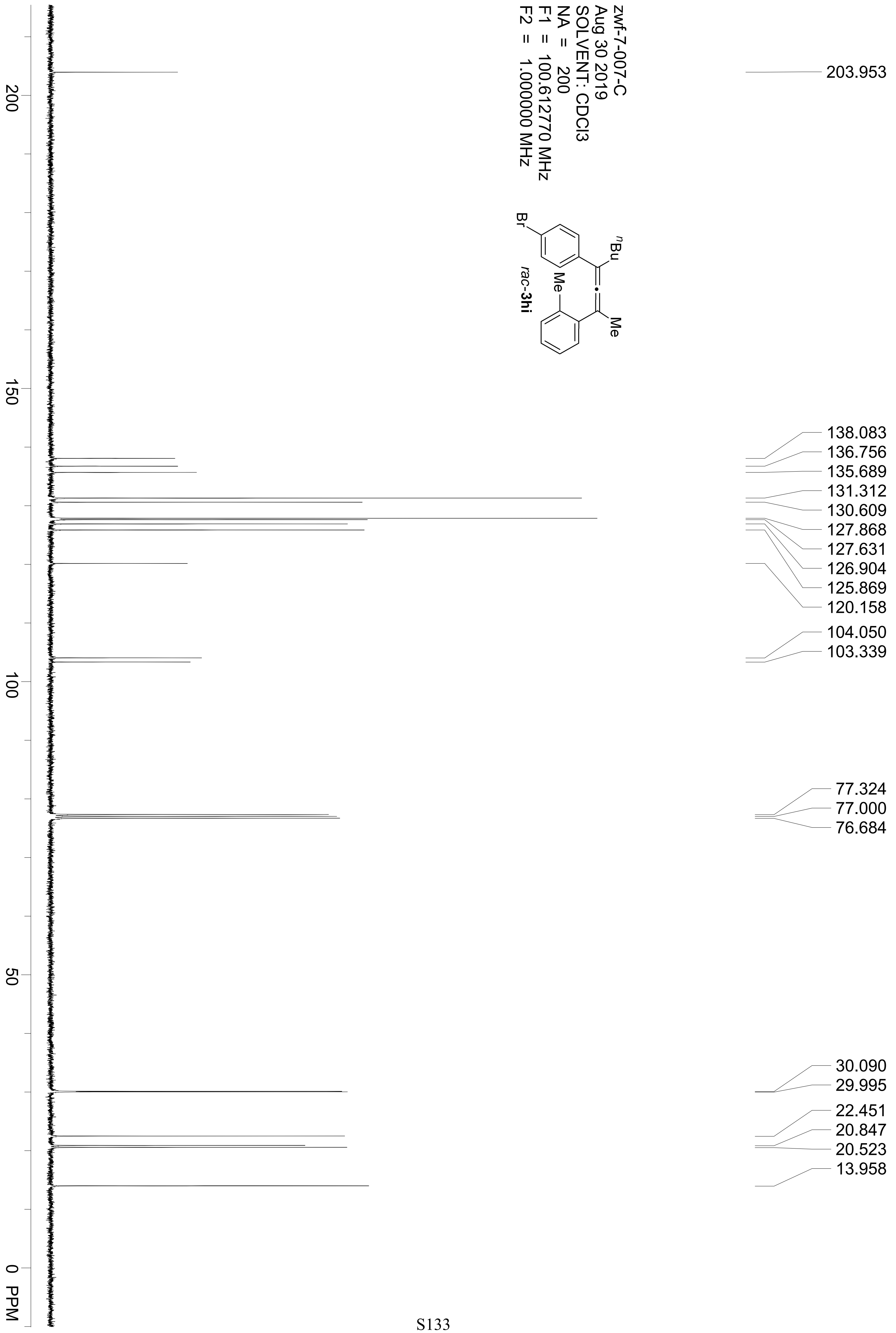

Supplementary Figure 61. ^{13}C NMR (100 MHz, $CDCl_3$) spectrum for *rac*-**3hi**

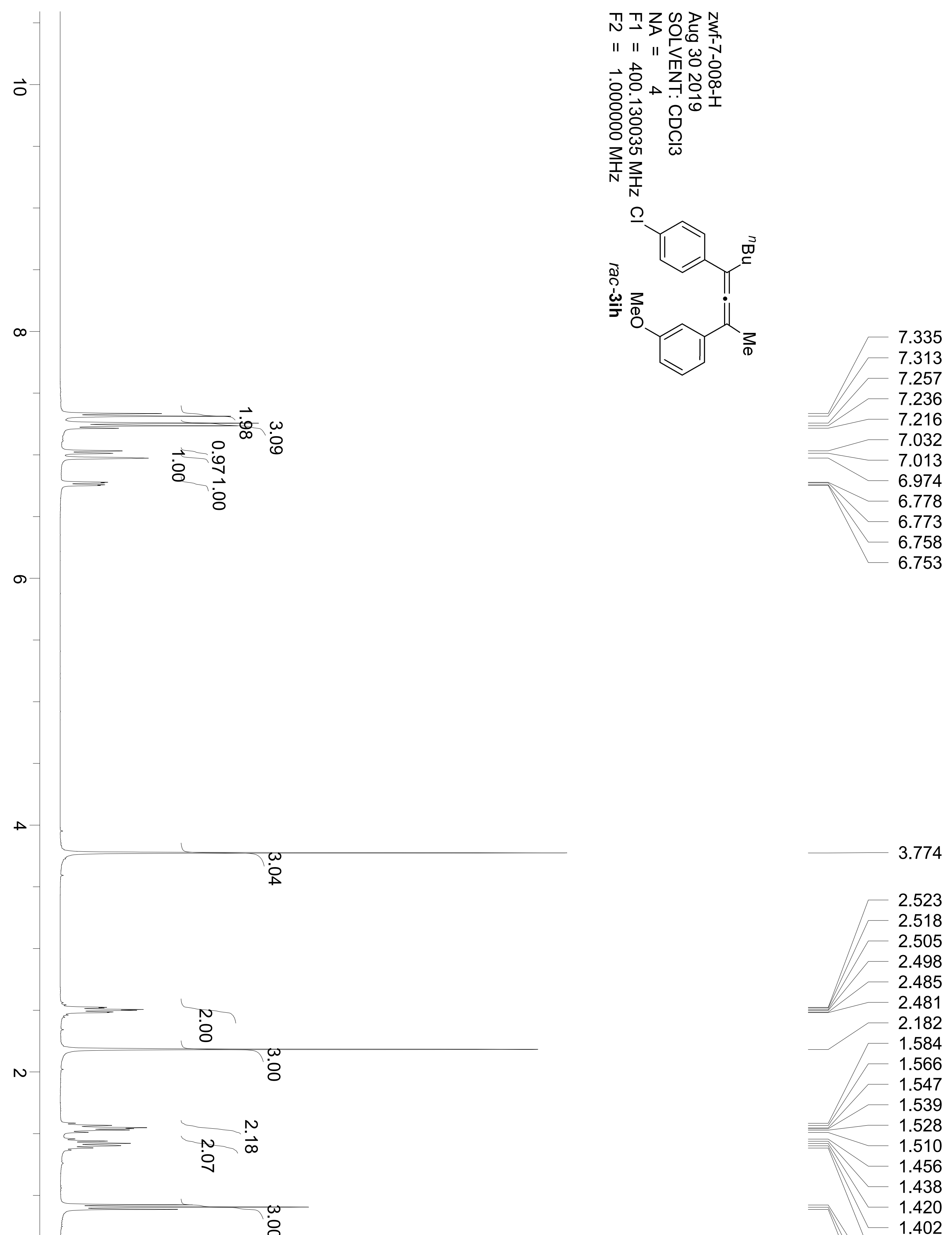

Supplementary Figure 62. ^{1}H NMR (400 MHz, $CDCl_3$) spectrum for *rac*-**3ih**

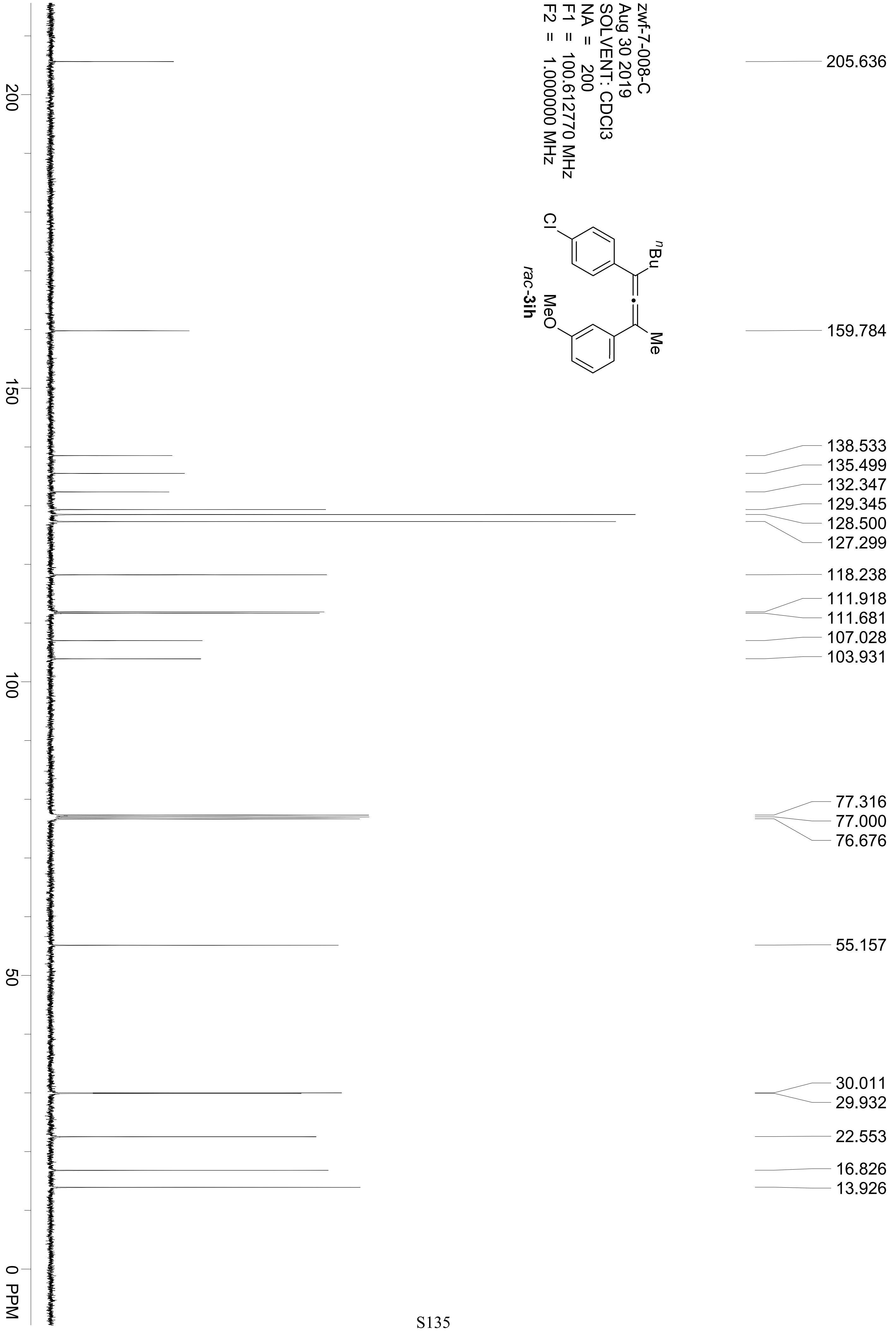

Supplementary Figure 63. ^{13}C NMR (100 MHz, $CDCl_3$) spectrum for *rac*-**3ih**

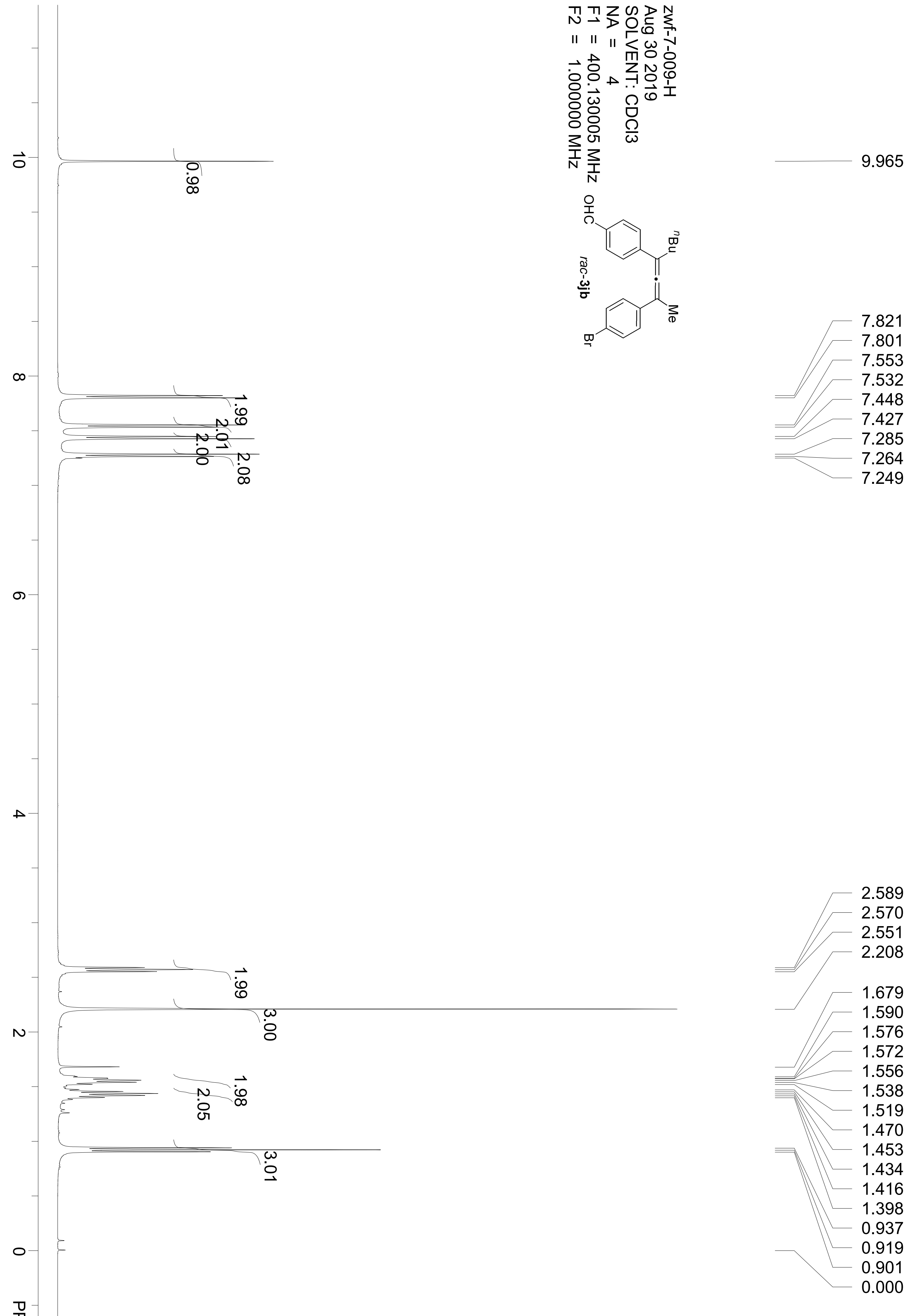

Supplementary Figure 64. ^{1}H NMR (400 MHz, $CDCl_3$) spectrum for *rac*-**3jb**

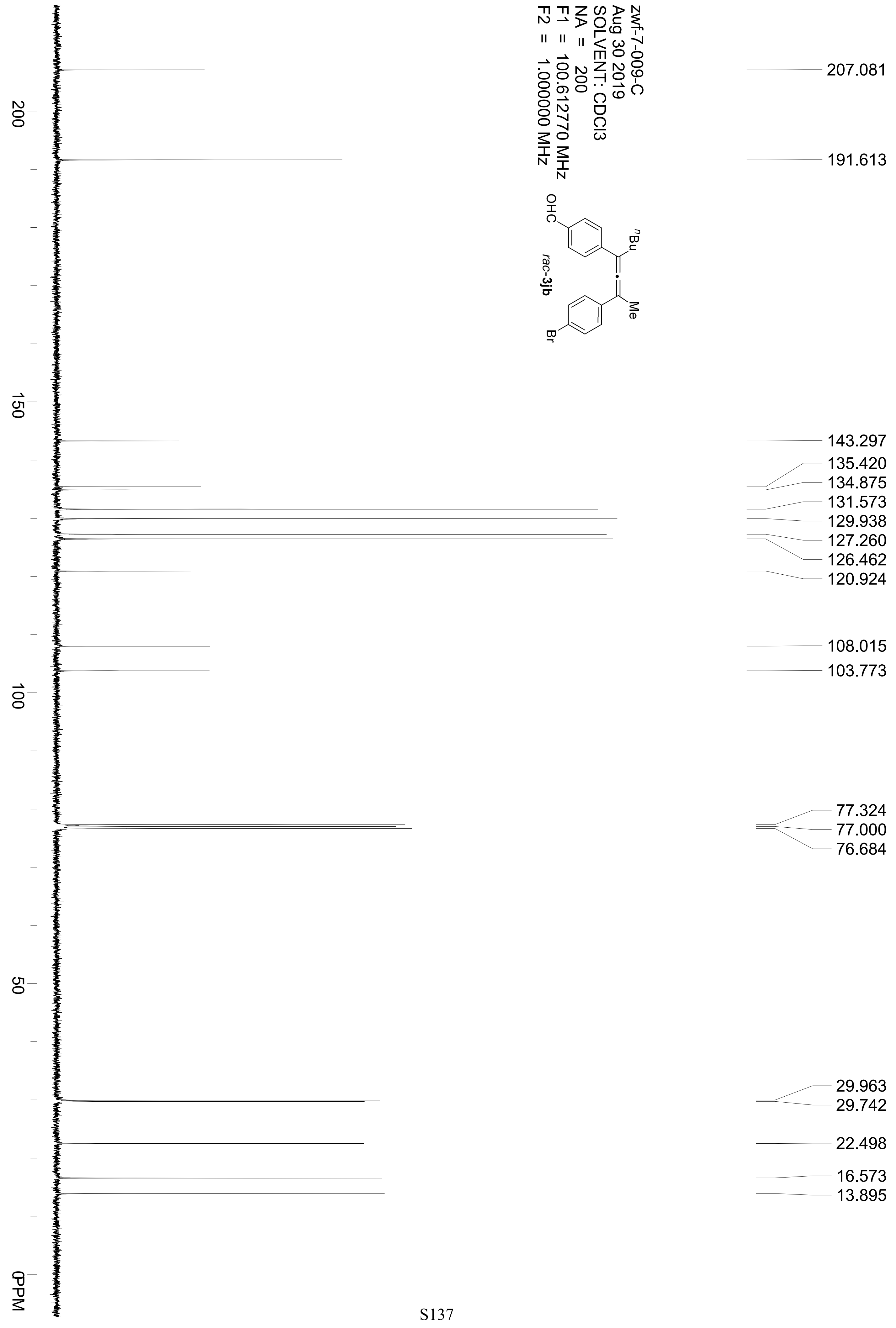

Supplementary Figure 65. ^{13}C NMR (100 MHz, $CDCl_3$) spectrum for *rac*-**3jb**

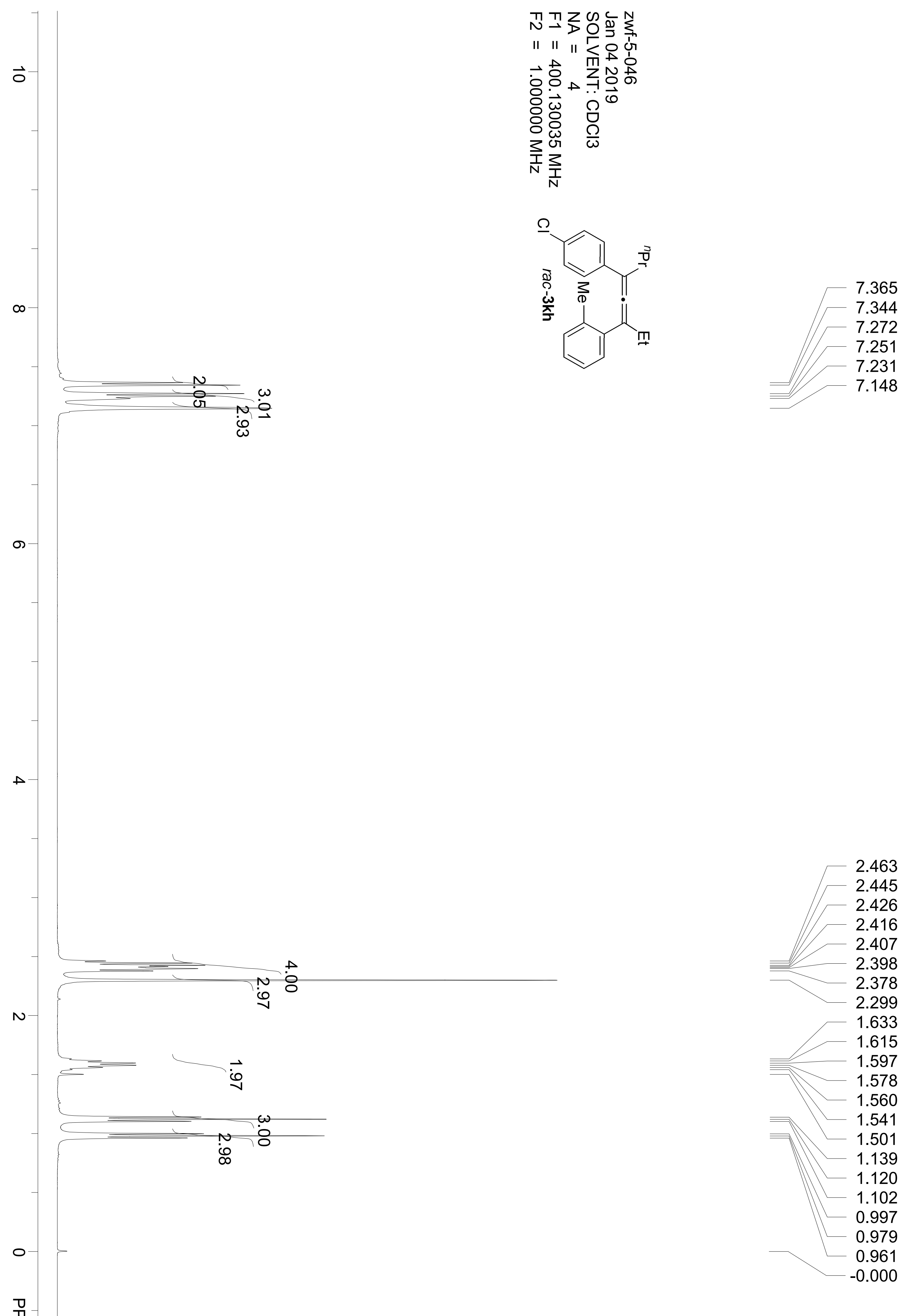

Supplementary Figure 66. ^{1}H NMR (400 MHz, $CDCl_3$) spectrum for *rac*-**3kh**

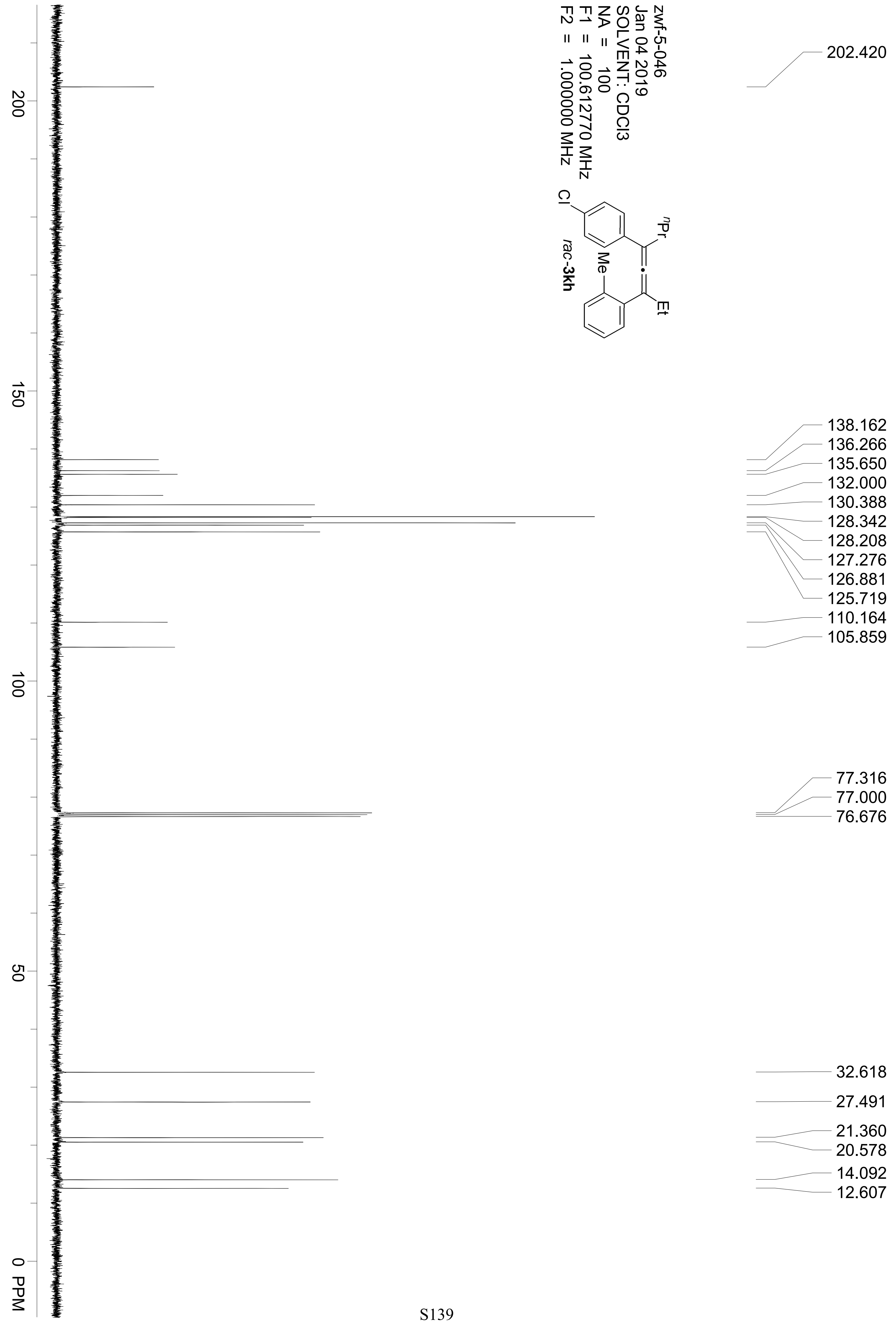

Supplementary Figure 67. ^{13}C NMR (100 MHz, $CDCl_3$) spectrum for *rac*-**3kh**

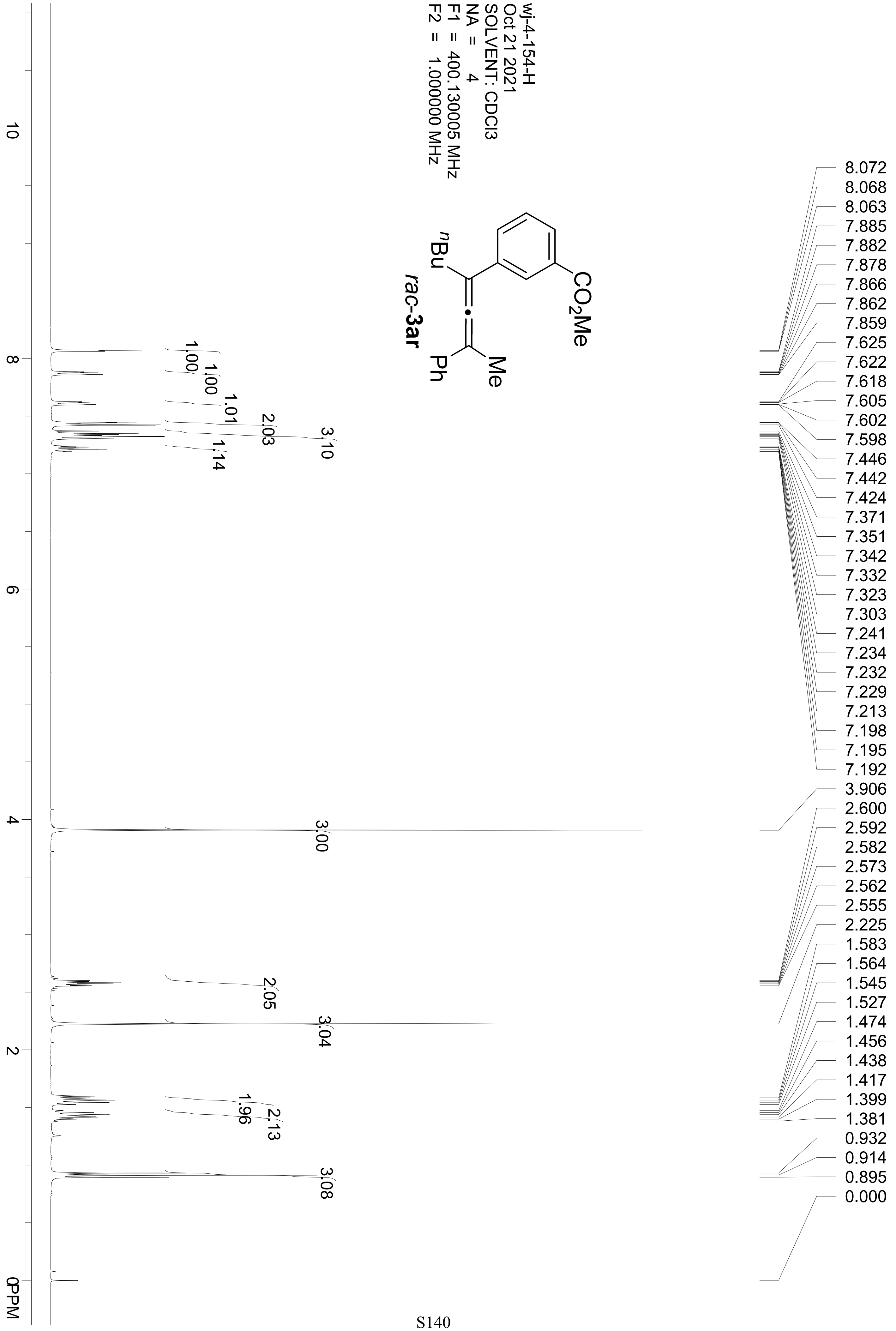

Supplementary Figure 68. ^{1}H NMR (400 MHz, $CDCl_3$) spectrum for *rac*-**3ar**

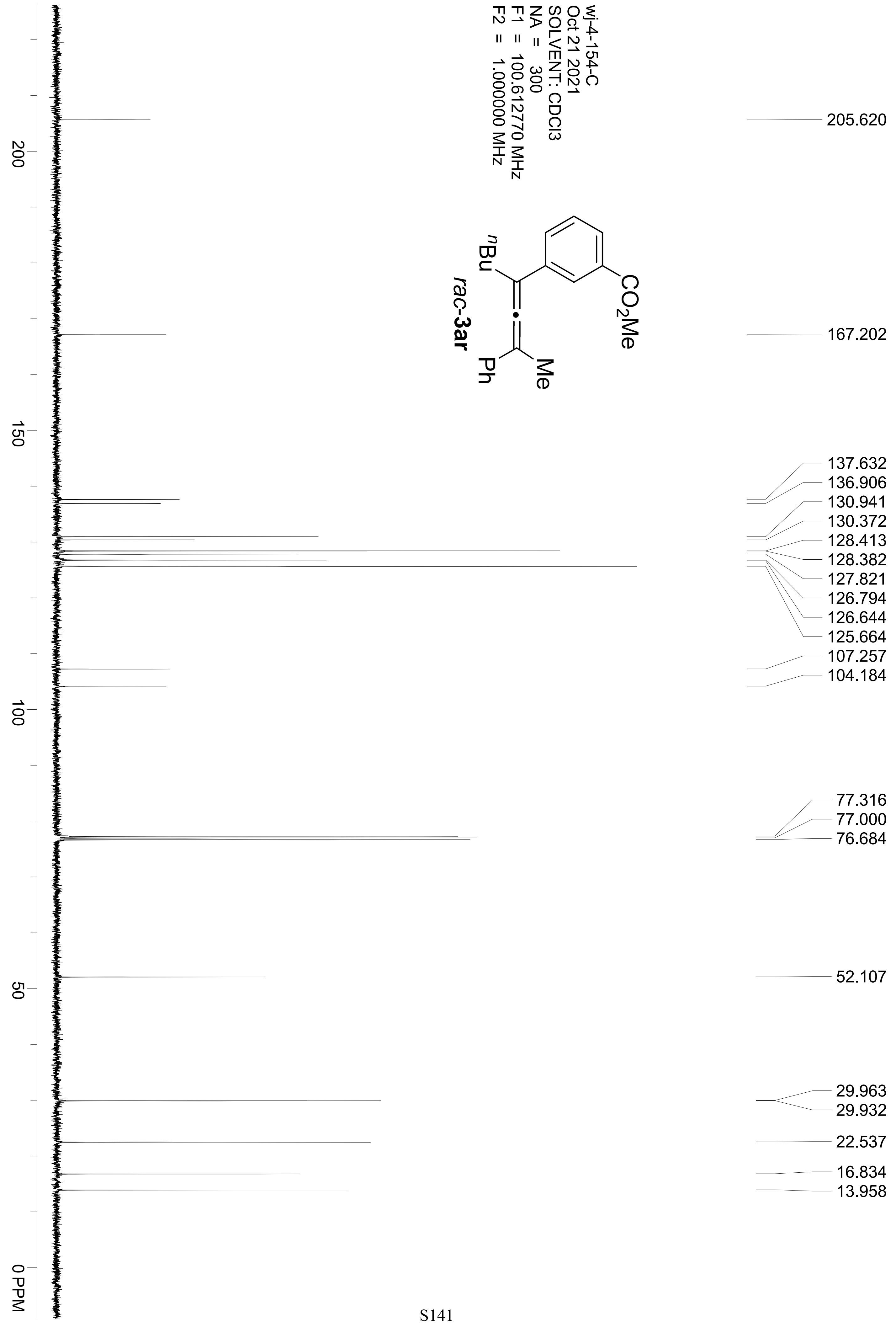

Supplementary Figure 69. ^{13}C NMR (100 MHz, $CDCl_3$) spectrum for *rac*-**3ar**

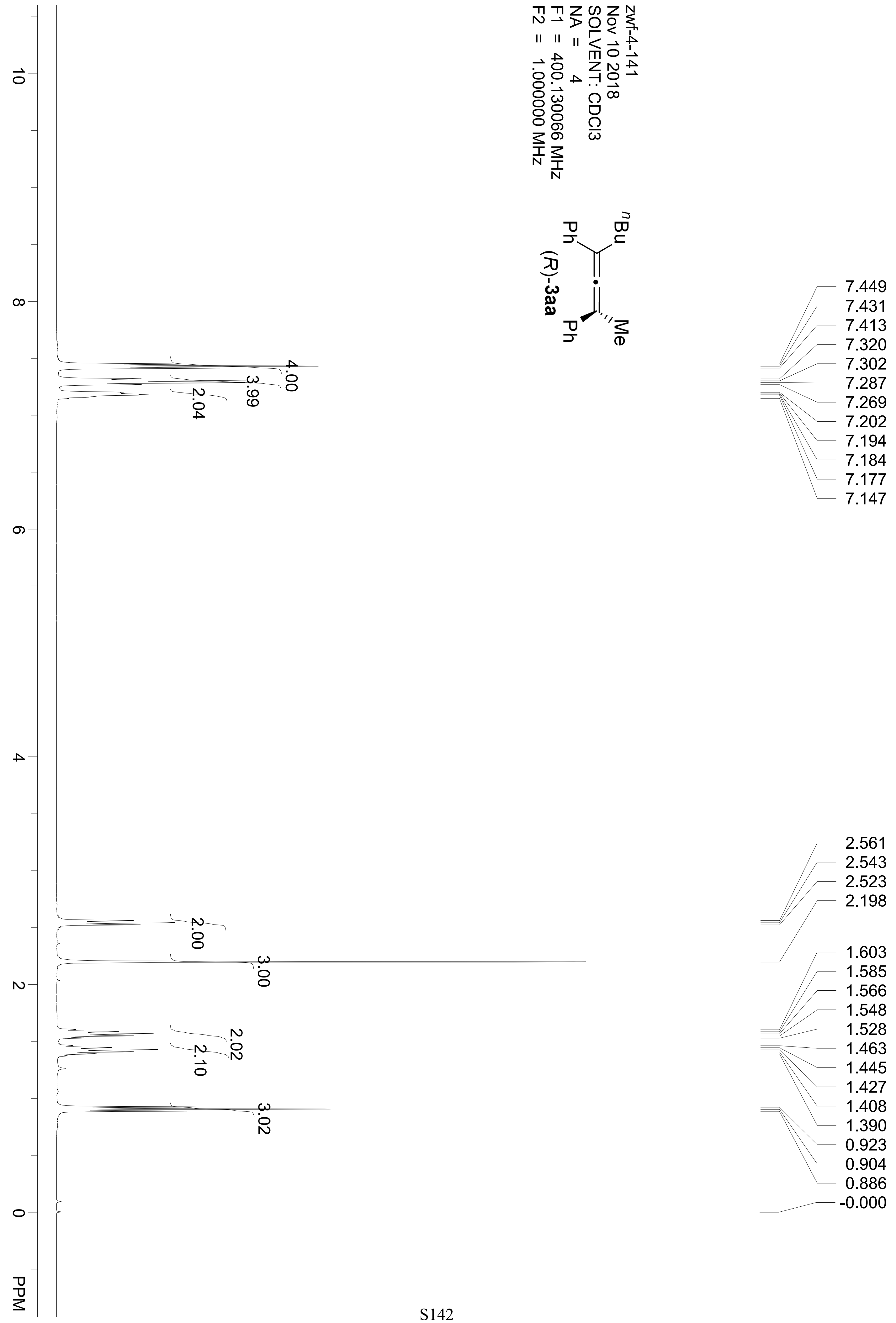

Supplementary Figure 70. ^{1}H NMR (400 MHz, $CDCl_3$) spectrum for (*R*)-**3aa**

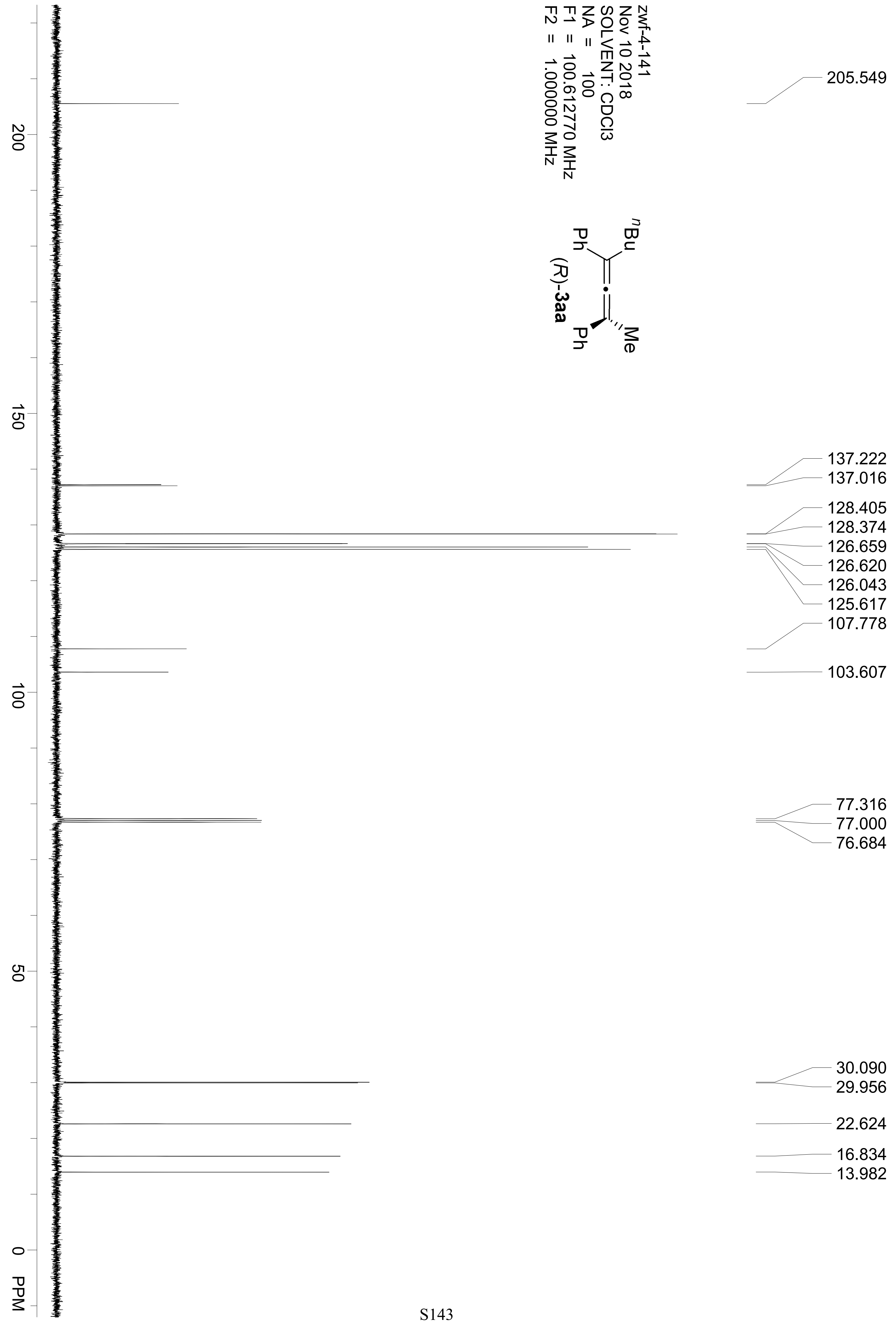

Supplementary Figure 71. ^{13}C NMR (100 MHz, $CDCl_3$) spectrum for (*R*)-**3aa**

Area Percent Report

Agilent Technologies

sample zwf-6-200-1-OJ-H-200-1-0.7-214

Data file: C:\Users\Public\Documents\ChemStation\1\Data\WJ_LC 2019-07-24 08-34-00\035-P1-C1-zwf-6-200-1.D

Acquisition Data:

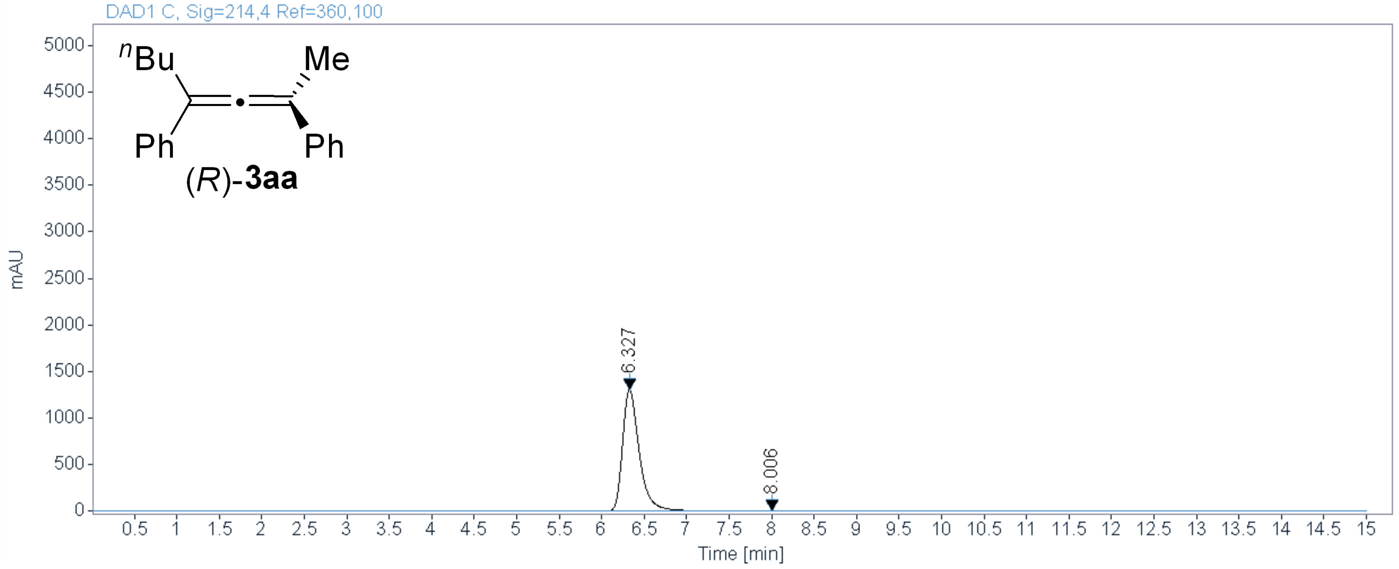

Signal: DAD1 C, Sig=214,4 Ref=360,100

RT [min]	Width [min]	Height	Area	Area%
6.327	0.2083	1308.0725	17968.8516	98.8580
8.006	0.4198	8.2422	207.5810	1.1420
		Sum	18176.4326	100.0000

Supplementary Figure 72. HPLC spectrum for (*R*)-**3aa**

Area Percent Report

Agilent Technologies

sample zwf-6-200-1-rac-OJ-H-200-1-0.7-214

Data file: C:\Users\Public\Documents\ChemStation\1\Data\WJ_LC 2019-07-24 08-34-00\037-P1-C3-zwf-6-200-1-rac.D

Acquisition Data:

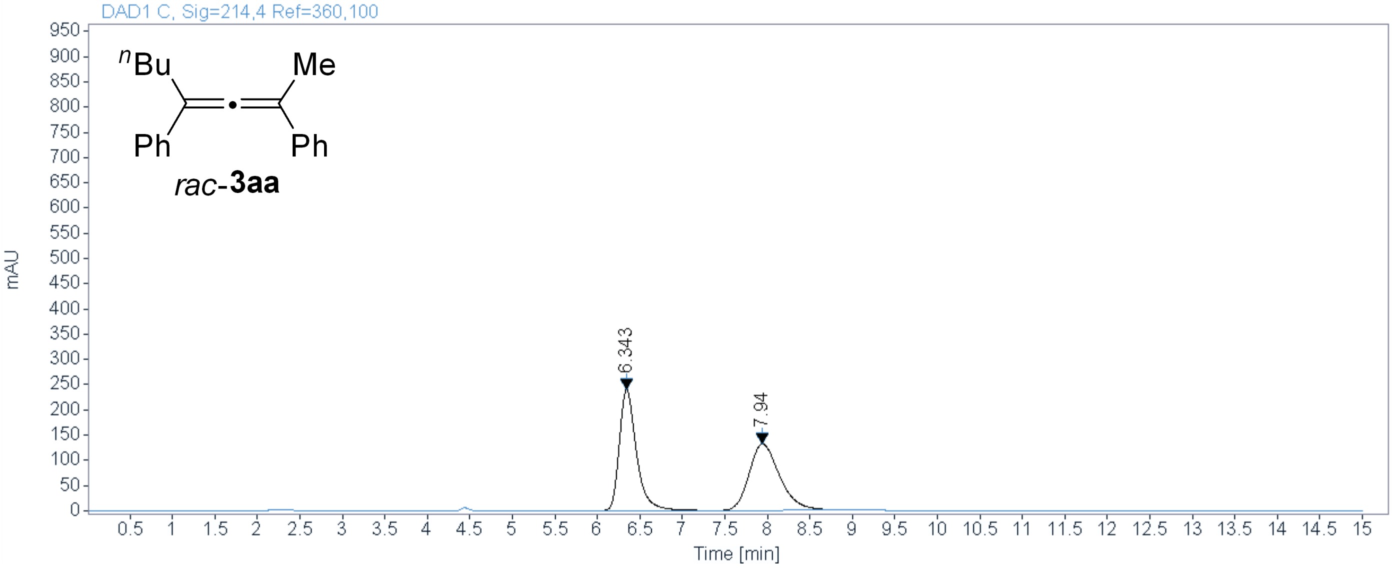

Signal: DAD1 C, Sig=214,4 Ref=360,100

RT [min]	Width [min]	Height	Area	Area%
6.343	0.2117	240.3114	3371.5481	50.4410
7.940	0.3879	131.5074	3312.5999	49.5590
		Sum	6684.1479	100.0000

Supplementary Figure 73. HPLC spectrum for *rac*-**3aa**

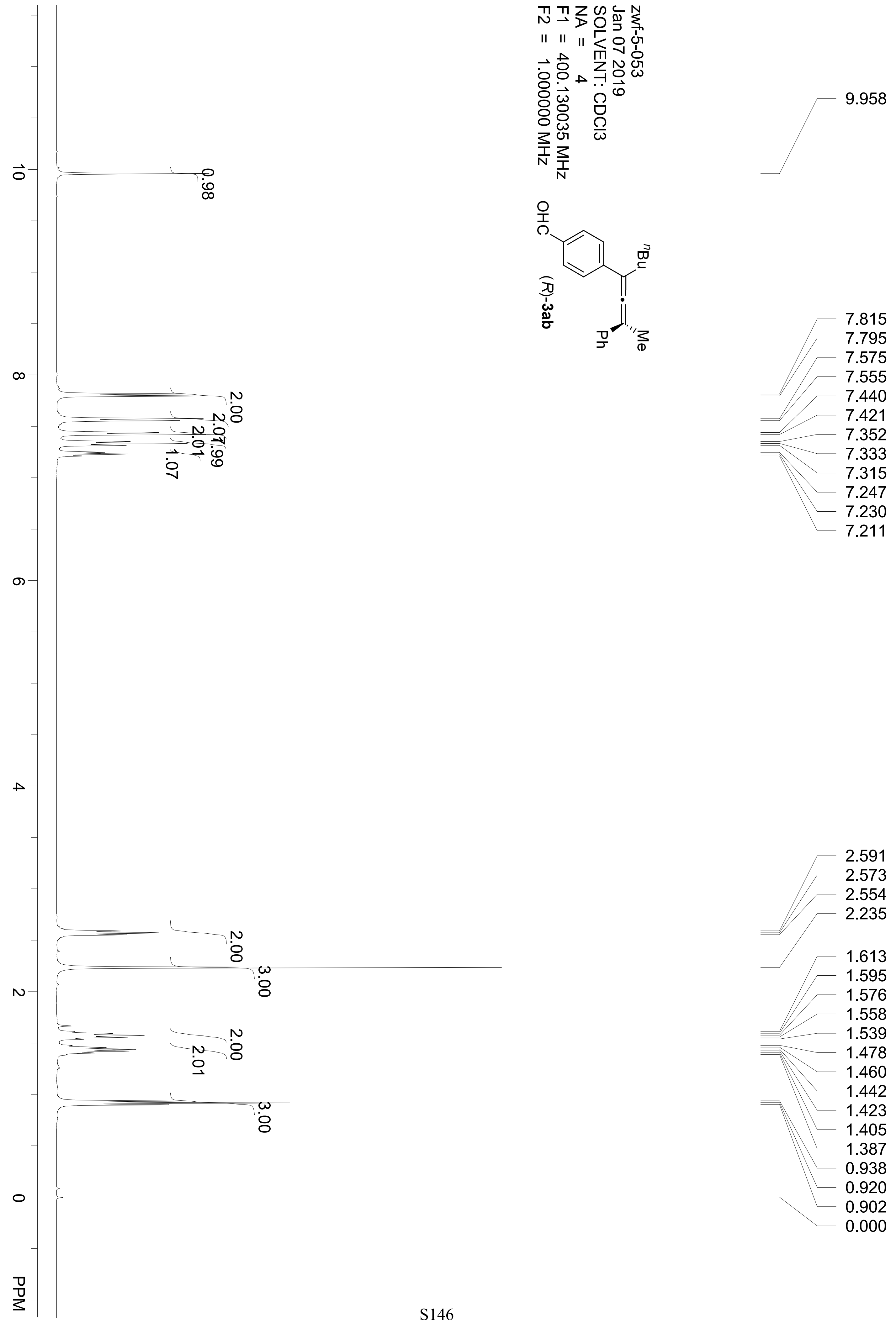

Supplementary Figure 74. ^{1}H NMR (400 MHz, $CDCl_3$) spectrum for (*R*)-**3ab**

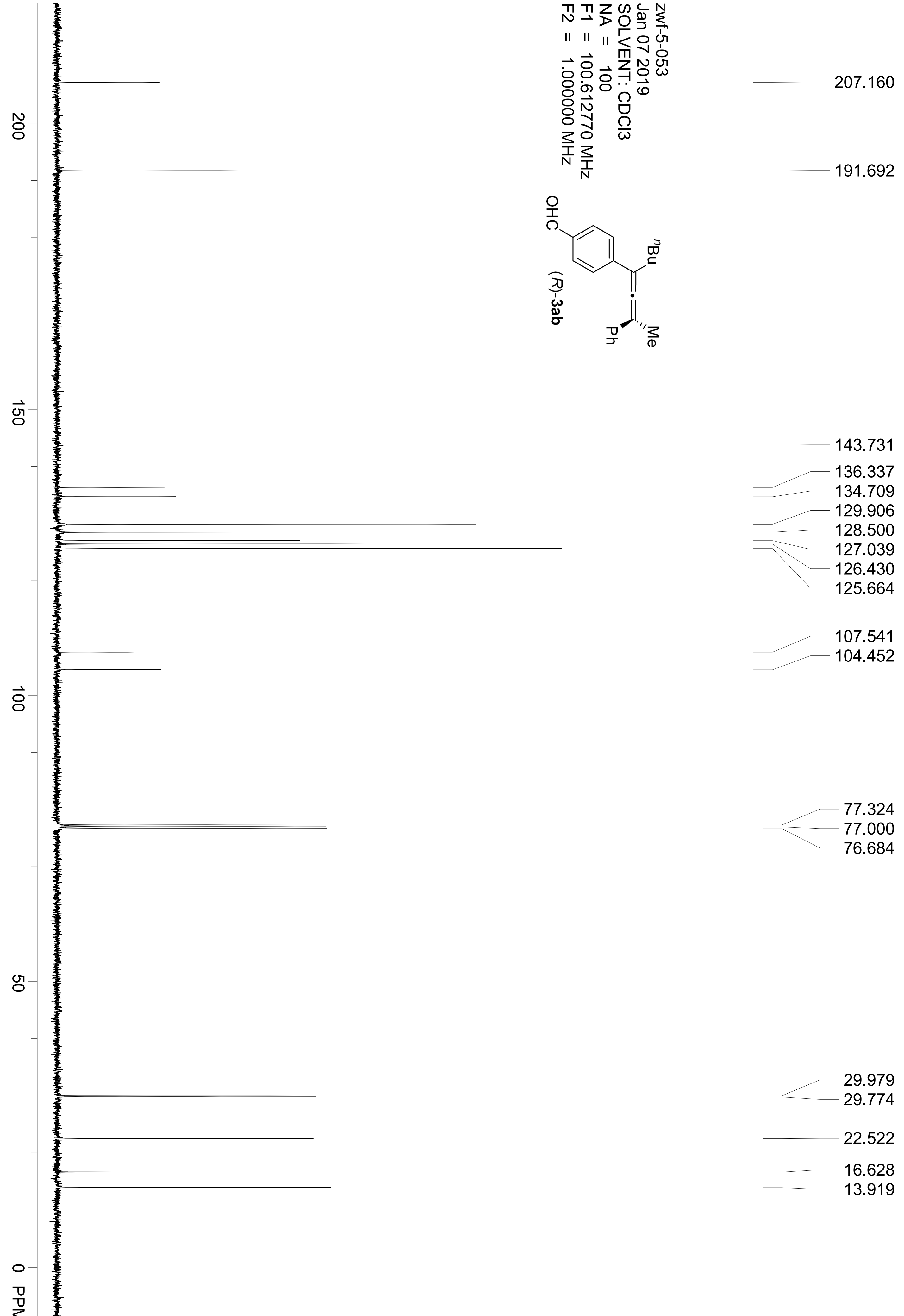

Supplementary Figure 75. ^{13}C NMR (100 MHz, $CDCl_3$) spectrum for (*R*)-**3ab**

Area Percent Report

Agilent Technologies

sample zwf-7-034-OD-H-99.5-0.5-1.0-214

Data file: C:\Users\Public\Documents\ChemStation\1\Data\zwf-allenioc acid_LC 2019-09-07 14-54-21\027-P1-B5-zwf-7-034.D

Acquisition Data:

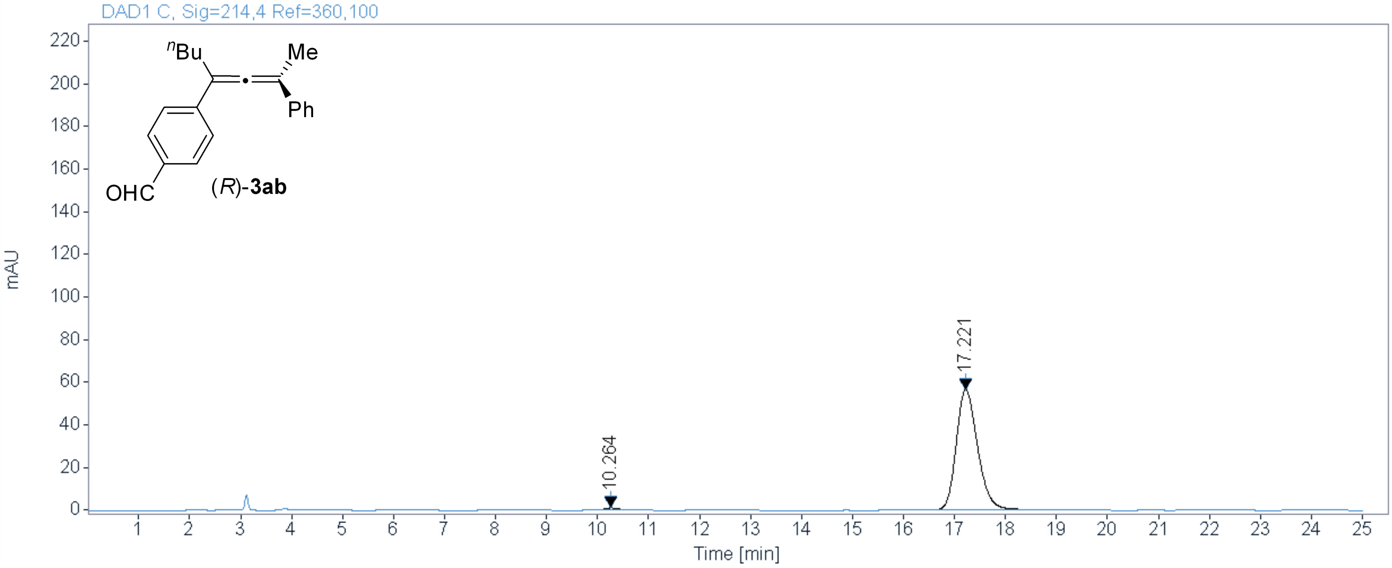

Signal: DAD1 C, Sig=214,4 Ref=360,100

RT [min]	Width [min]	Height	Area	Area%
10.264	0.2567	0.8517	13.1174	0.8023
17.221	0.4435	56.3983	1621.8859	99.1977
		Sum	1635.0033	100.0000

Supplementary Figure 76. HPLC spectrum for (*R*)-**3ab**

Area Percent Report

Agilent Technologies

sample zwf-7-034-rac-OD-H-99.5-0.5-1.0-214

Data file: C:\Users\Public\Documents\ChemStation\1\Data\zwf-allenioc acid_LC 2019-09-07 14-54-21\028-P1-B6-zwf-7-034-rac.D

Acquisition Data:

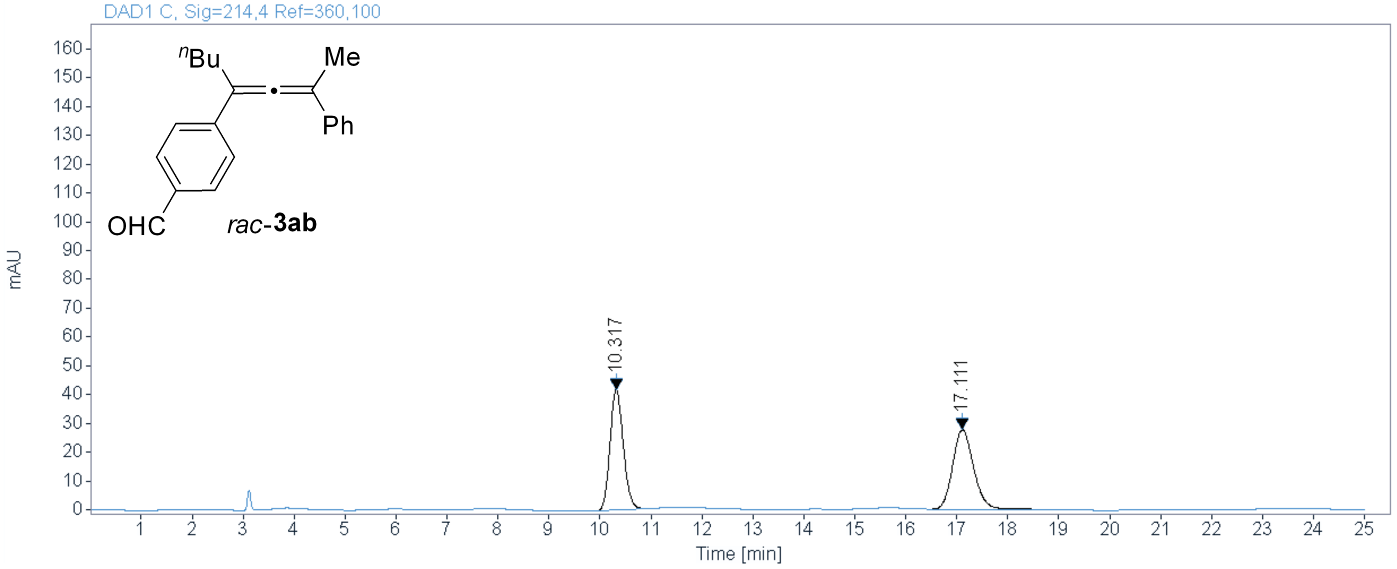

Signal: DAD1 C, Sig=214,4 Ref=360,100

RT [min]	Width [min]	Height	Area	Area%
10.317	0.2829	42.0488	767.4048	49.1709
17.111	0.4448	27.6419	793.2841	50.8291
		Sum	1560.6890	100.0000

Supplementary Figure 77. HPLC spectrum for *rac*-**3ab**

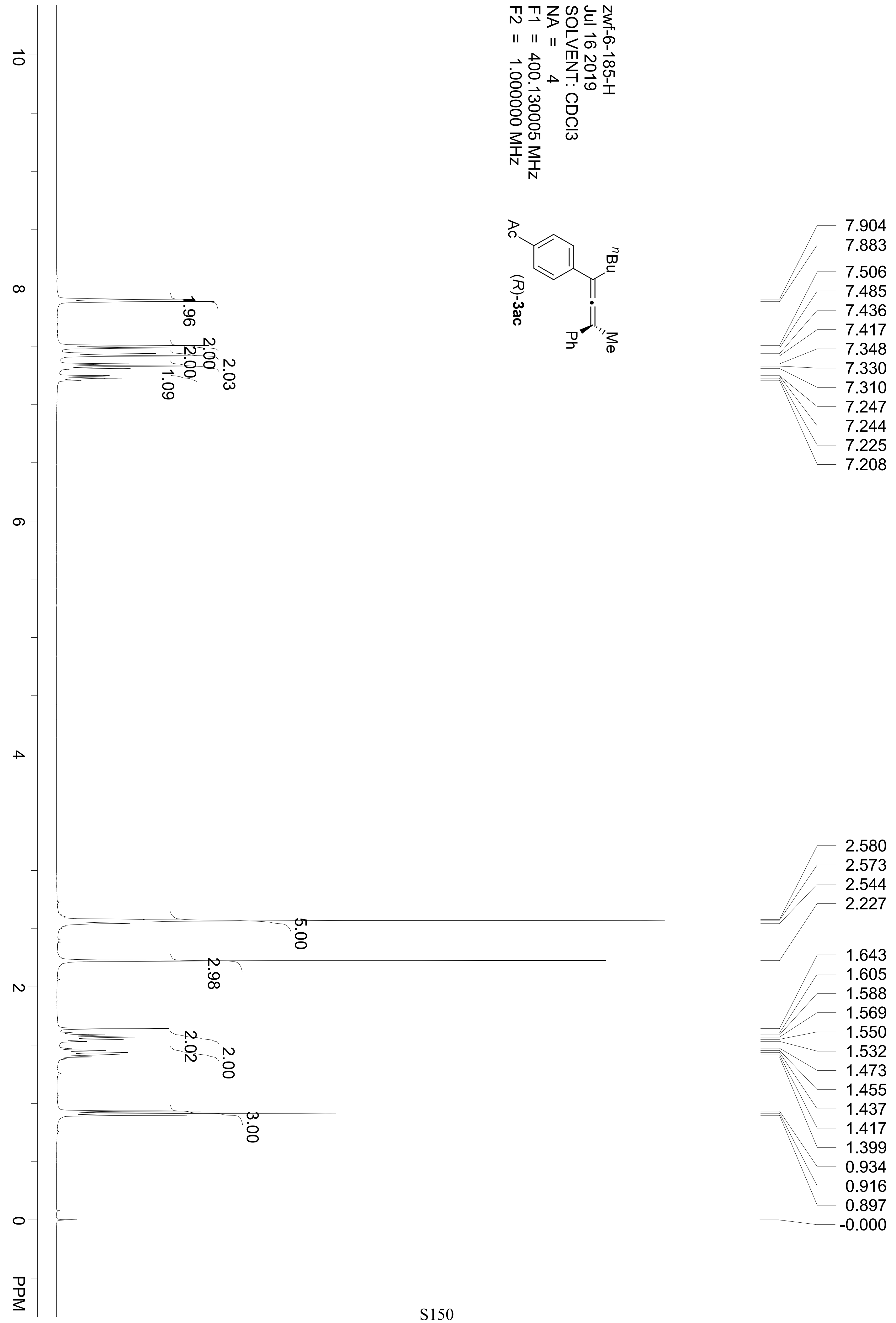

Supplementary Figure 78. ^{1}H NMR (400 MHz, $CDCl_3$) spectrum for (*R*)-**3ac**

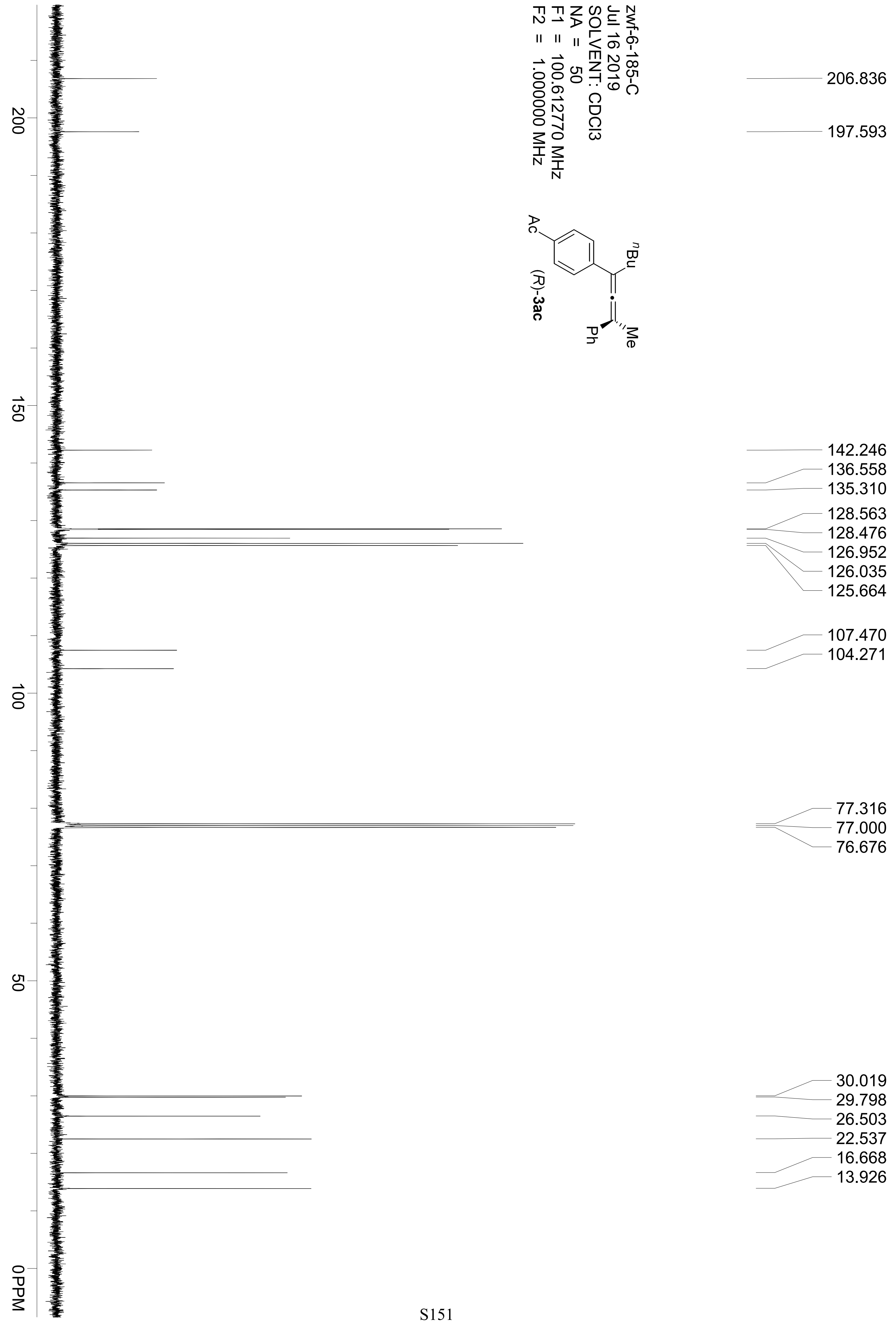

Supplementary Figure 79. ^{13}C NMR (100 MHz, $CDCl_3$) spectrum for (*R*)-**3ac**

Area Percent Report

Agilent Technologies

sample zwf-7-038-OD-H-99.5-0.5-1.0-214

Data file: C:\Users\Public\Documents\ChemStation\1\Data\zwf-allenioc acid_LC 2019-09-09 16-35-10\004-P1-C1-zwf-7-038.D

Acquisition Data:

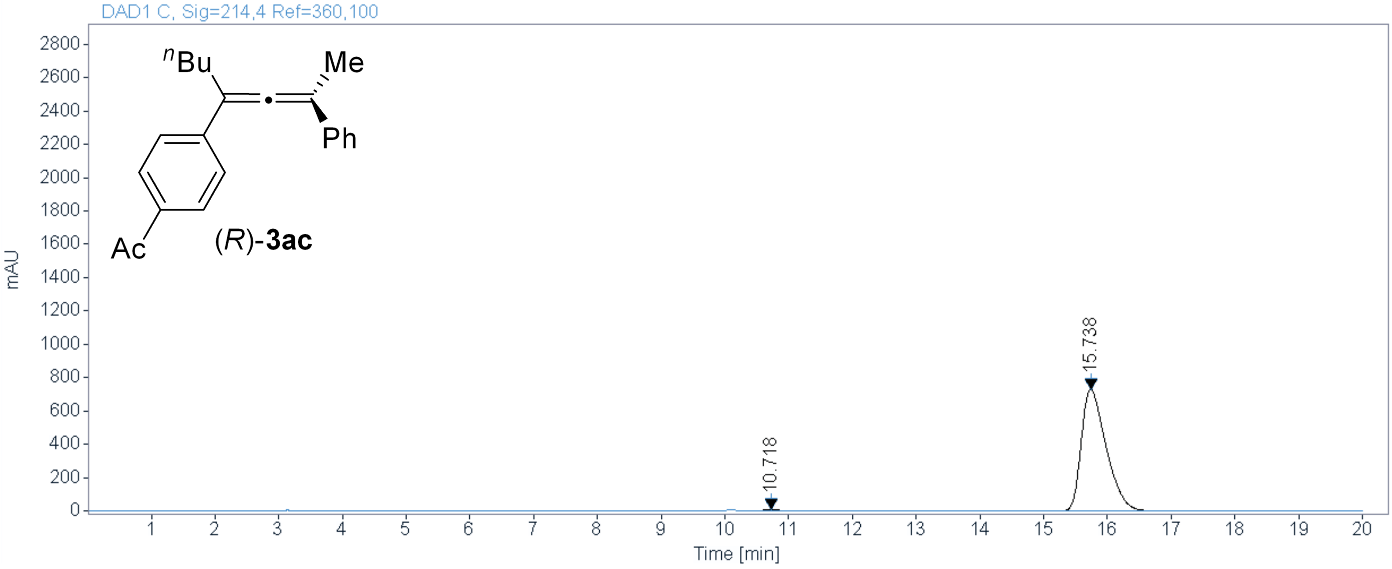

Signal: DAD1 C, Sig=214,4 Ref=360,100

RT [min]	Width [min]	Height	Area	Area%
10.718	0.2522	9.7484	158.9278	0.7934
15.738	0.4174	730.4096	19873.1875	99.2066
		Sum	20032.1153	100.0000

Supplementary Figure 80. HPLC spectrum for (*R*)-**3ac**

Area Percent Report

sample zwf-7-038-rac-OD-H-99.5-0.5-1.0-214

Data file: C:\Users\Public\Documents\ChemStation\1\Data\zwf-allenioc acid_LC 2019-09-09 16-35-10\003-P1-C2-zwf-7-038-rac.D

Acquisition Data:

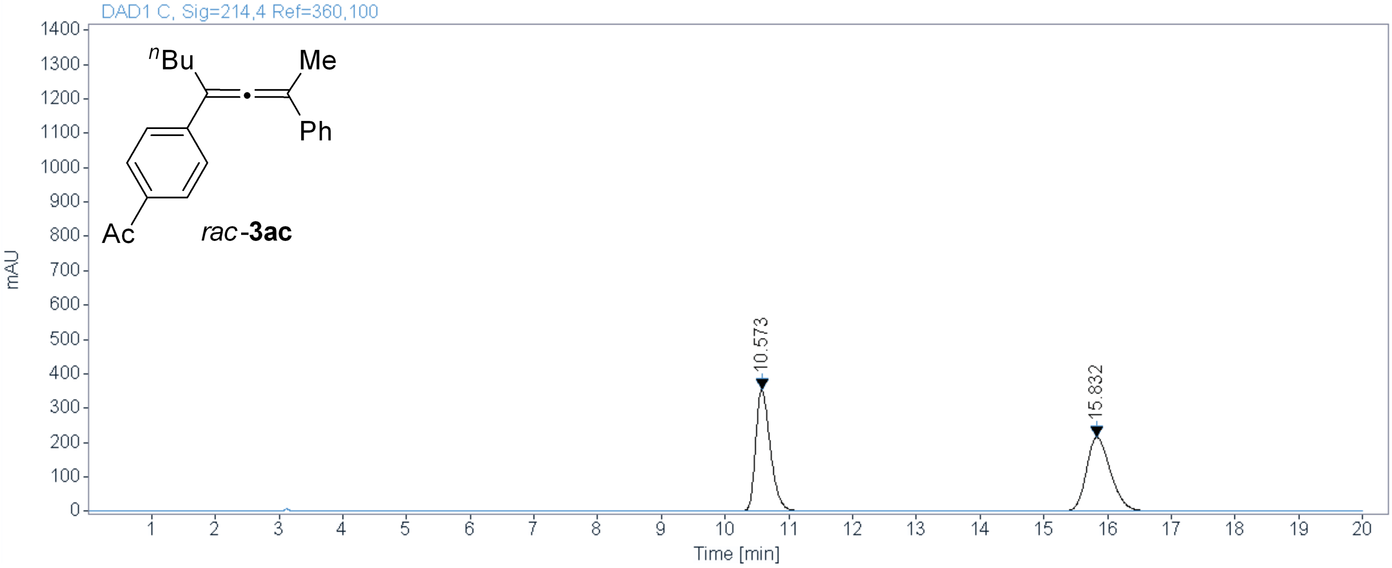

Signal: DAD1 C, Sig=214,4 Ref=360,100

RT [min]	Width [min]	Height	Area	Area%
10.573	0.2429	354.6315	5651.0142	49.9723
15.832	0.4066	215.2722	5657.2842	50.0277
		Sum	11308.2983	100.0000

Supplementary Figure 81. HPLC spectrum for *rac*-**3ac**

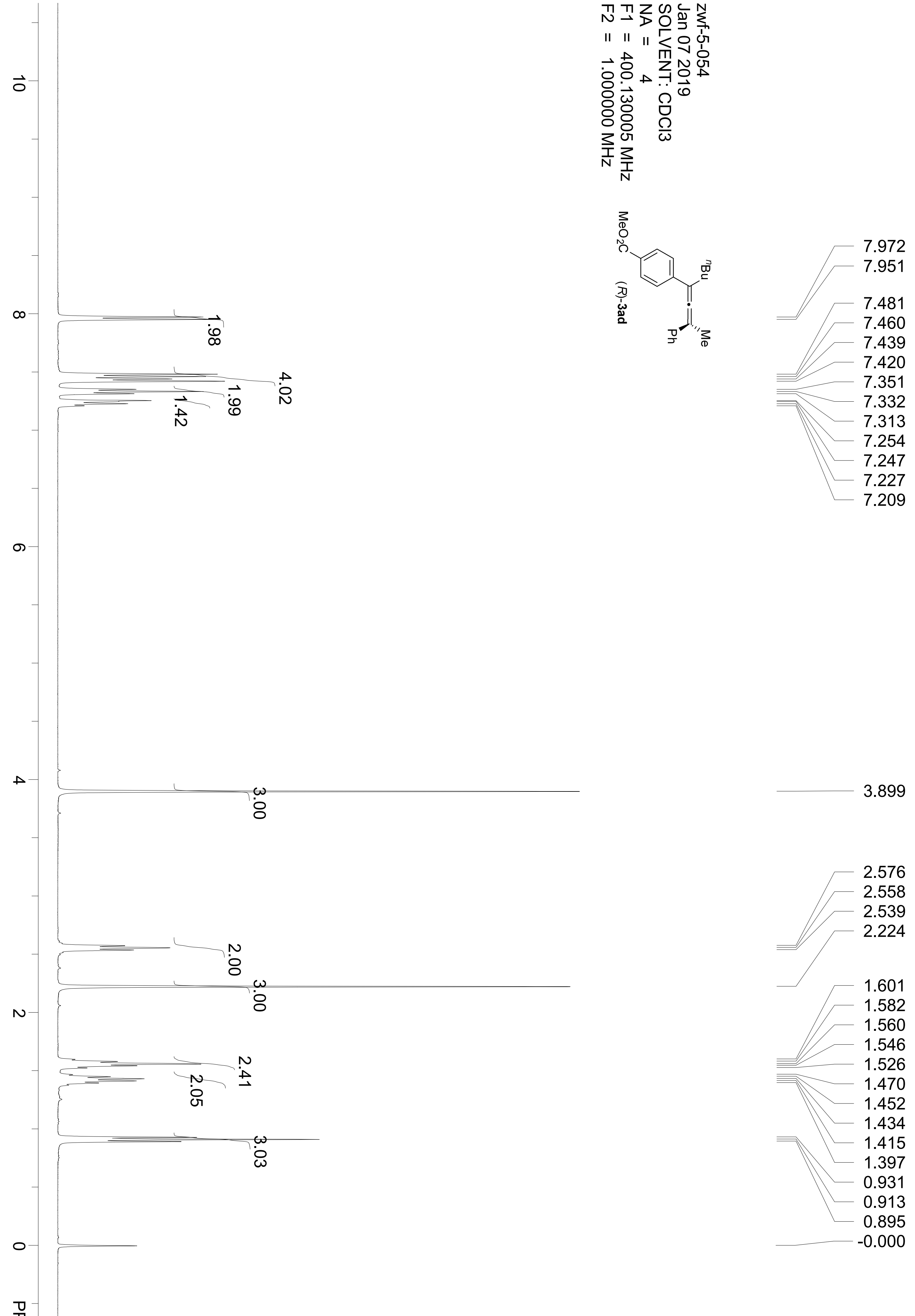

Supplementary Figure 82. ^{1}H NMR (400 MHz, $CDCl_3$) spectrum for (*R*)-**3ad**

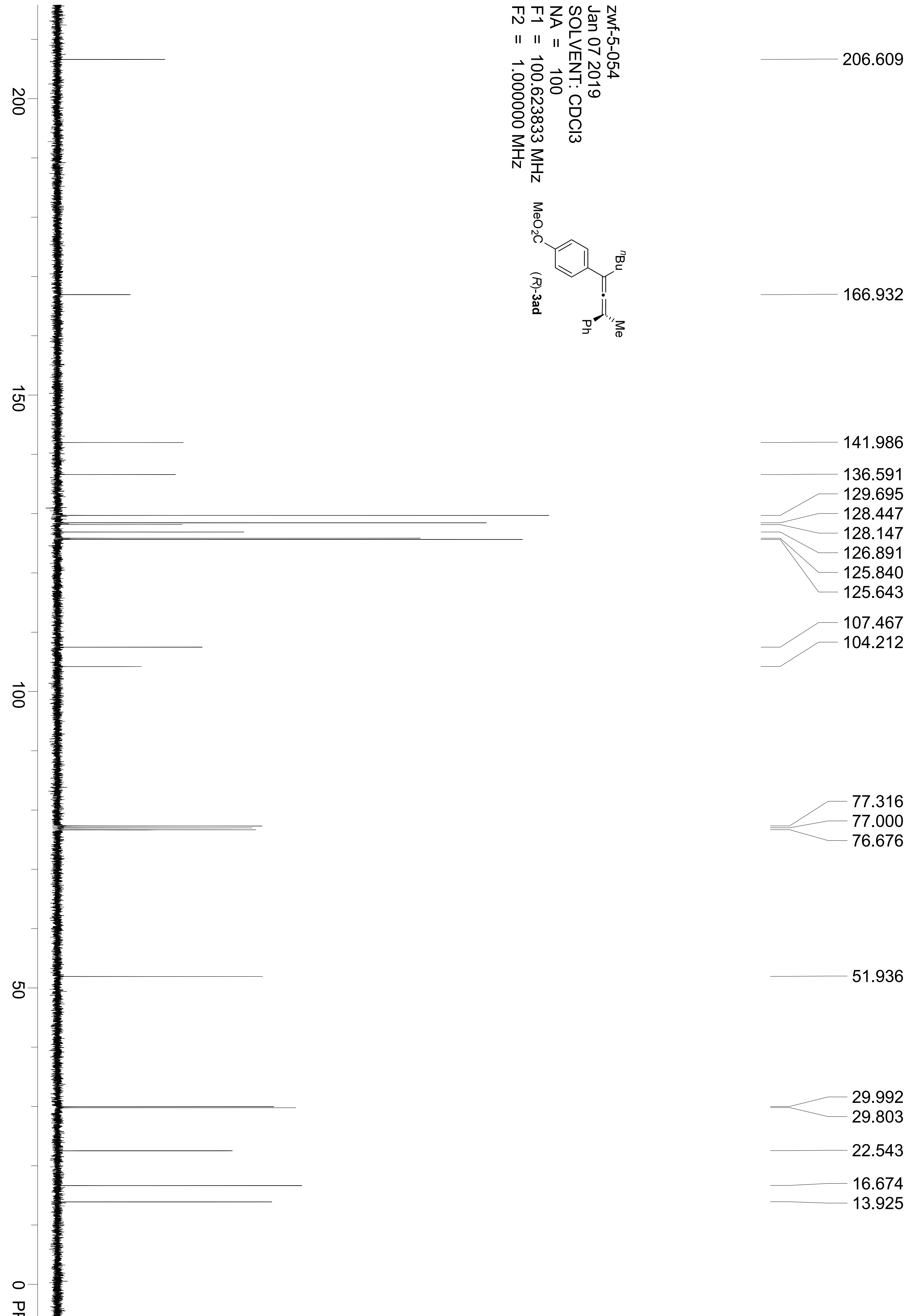

Supplementary Figure 83. ^{13}C NMR (100 MHz, $CDCl_3$) spectrum for (*R*)-**3ad**

Area Percent Report

Agilent Technologies

sample zwf-7-039-OD-H-99.5-0.5-1.0-214

Data file: C:\Users\Public\Documents\ChemStation\1\Data\zwf-allenioc acid_LC 2019-09-09 16-35-10\005-P1-C3-zwf-7-039.D

Acquisition Data:

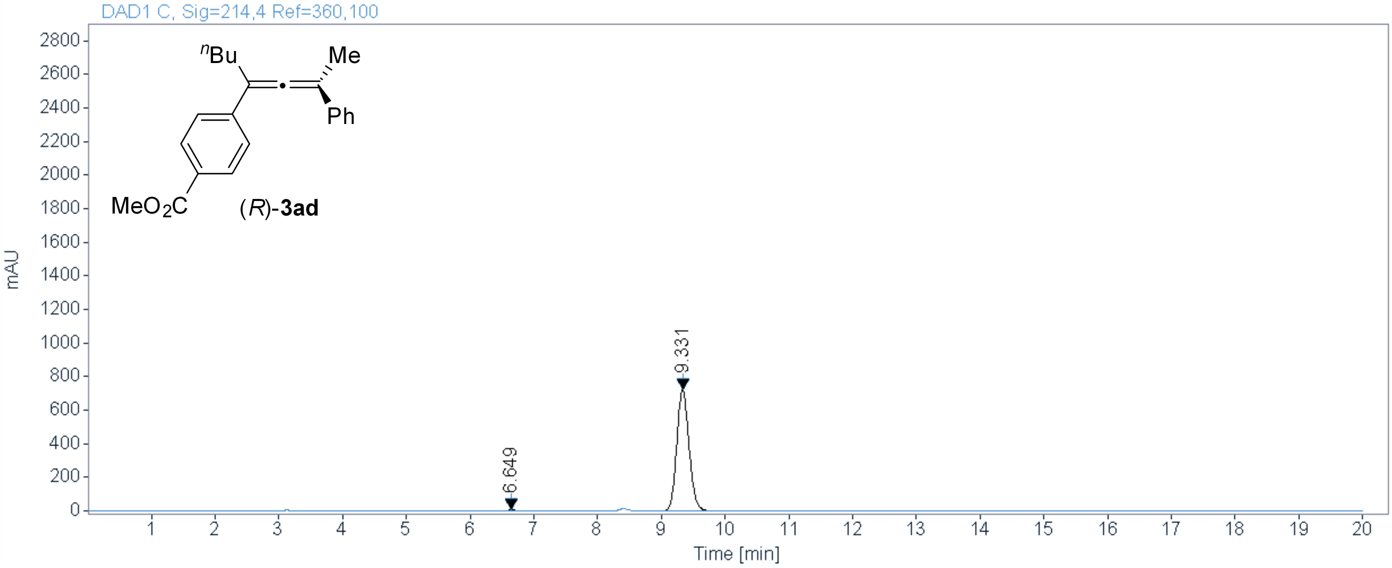

Signal: DAD1 C, Sig=214,4 Ref=360,100

RT [min]	Width [min]	Height	Area	Area%
6.649	0.1414	6.8024	61.4261	0.6248
9.331	0.2072	725.1760	9769.2666	99.3752
		Sum	9830.6927	100.0000

Supplementary Figure 84. HPLC spectrum for (*R*)-**3ad**

Area Percent Report

sample zwf-7-039-rac-OD-H-99.5-0.5-1.0-214

Data file: C:\Users\Public\Documents\ChemStation\1\Data\zwf-allenioc acid_LC 2019-09-09 16-35-10\006-P1-C4-zwf-7-039-rac.D

Acquisition Data:

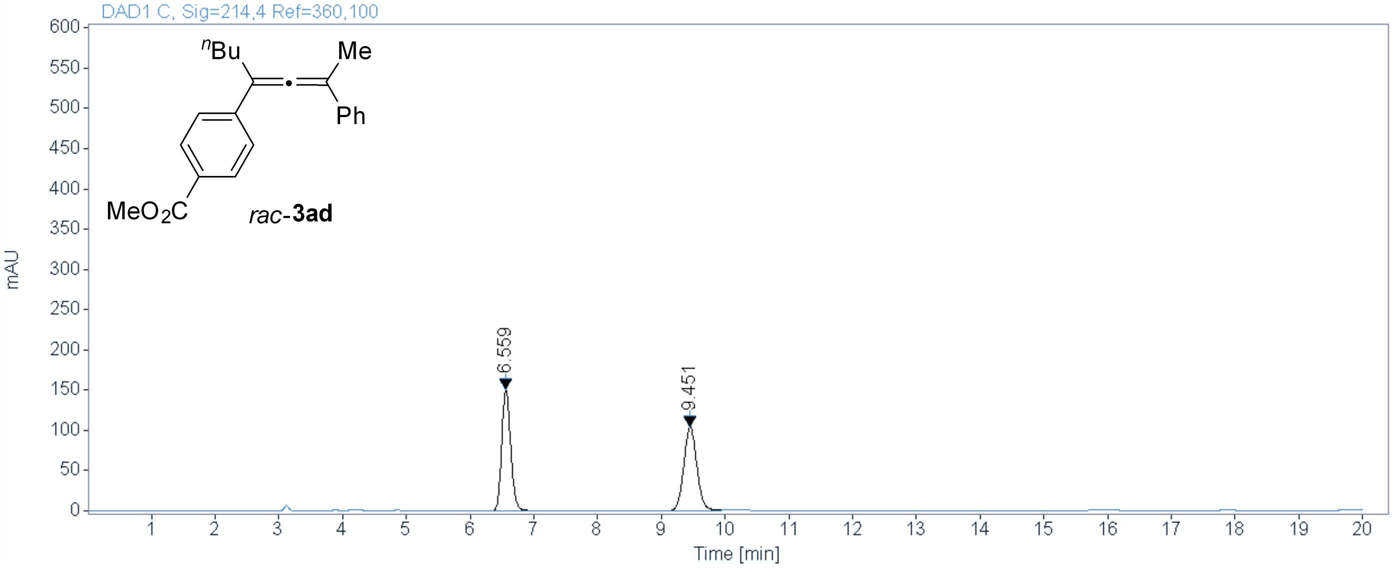

Signal: DAD1 C, Sig=214,4 Ref=360,100

RT [min]	Width [min]	Height	Area	Area%
6.559	0.1502	150.9416	1477.9921	49.6736
9.451	0.2388	104.5148	1497.4130	50.3264
		Sum	2975.4050	100.0000

Supplementary Figure 85. HPLC spectrum for *rac*-**3ad**

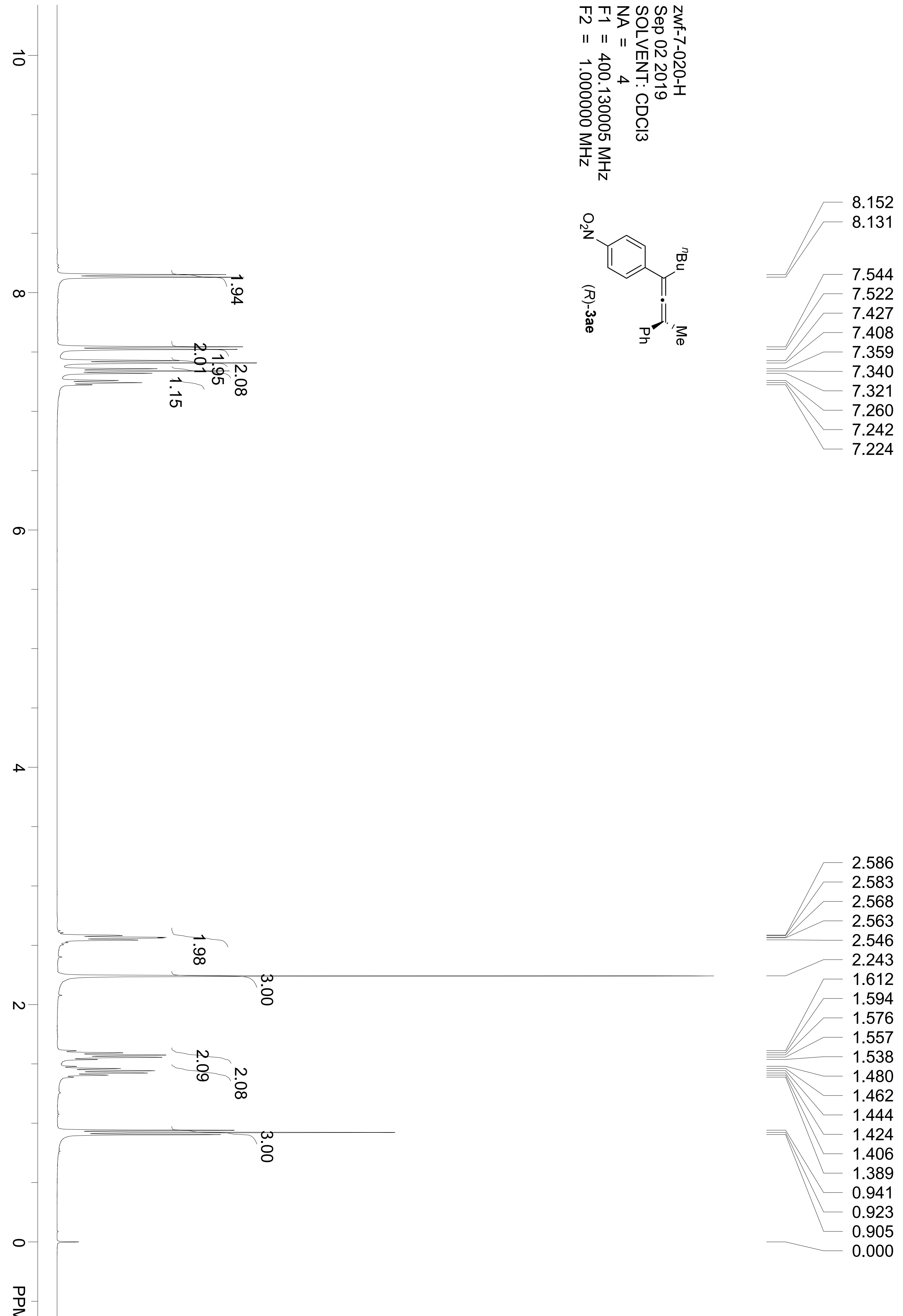

Supplementary Figure 86. ^{1}H NMR (400 MHz, $CDCl_3$) spectrum for (*R*)-**3ae**

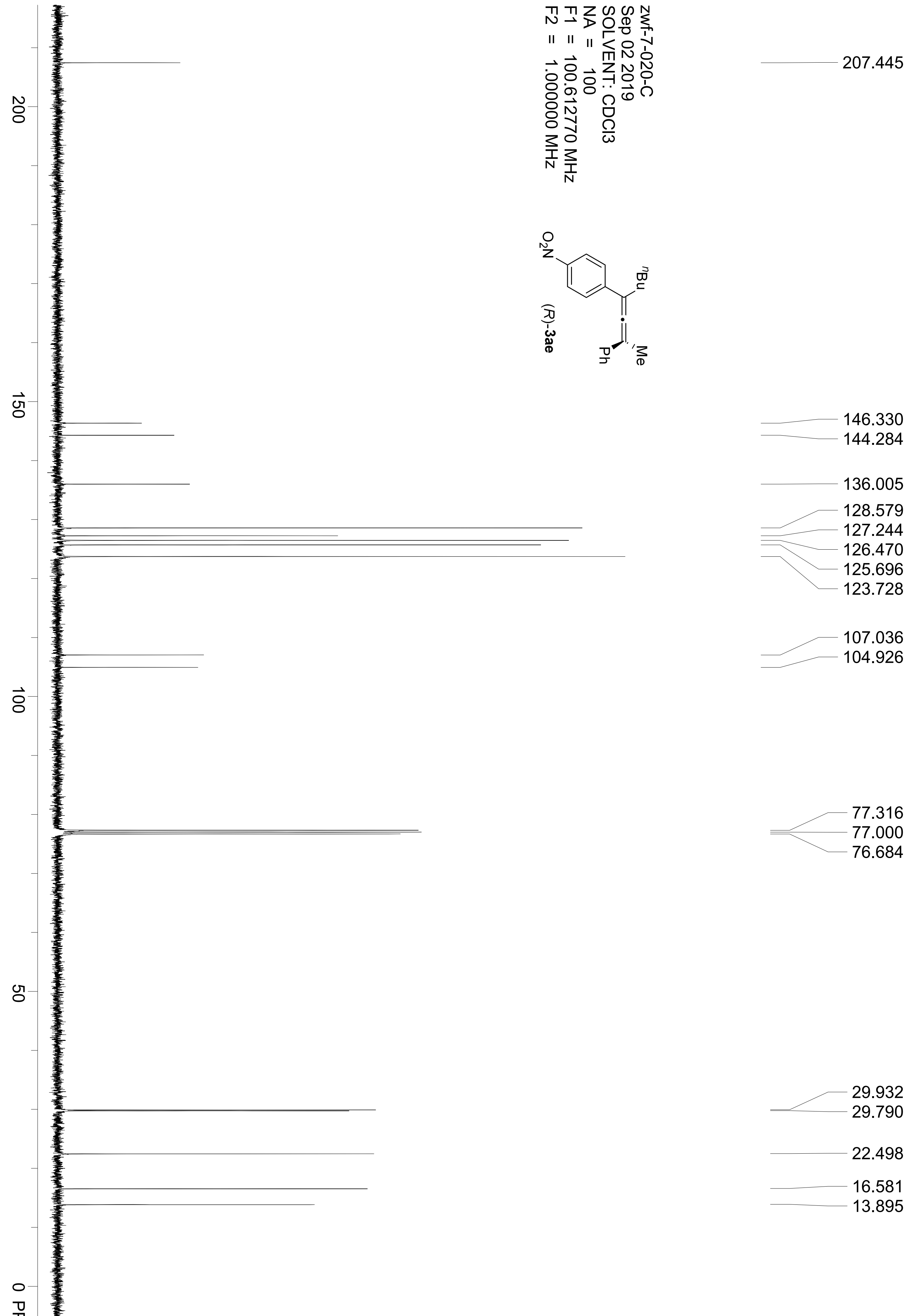

Supplementary Figure 87. ^{13}C NMR (100 MHz, $CDCl_3$) spectrum for (*R*)-**3ae**

Area Percent Report

Agilent Technologies

sample zwf-7-020-OD-H-99.5-0.5-1.0-214

Data file: C:\Users\Public\Documents\ChemStation\1\Data\wgl 2019-09-03 07-35-18\038-P1-C1-zwf-7-020.D

Acquisition Data:

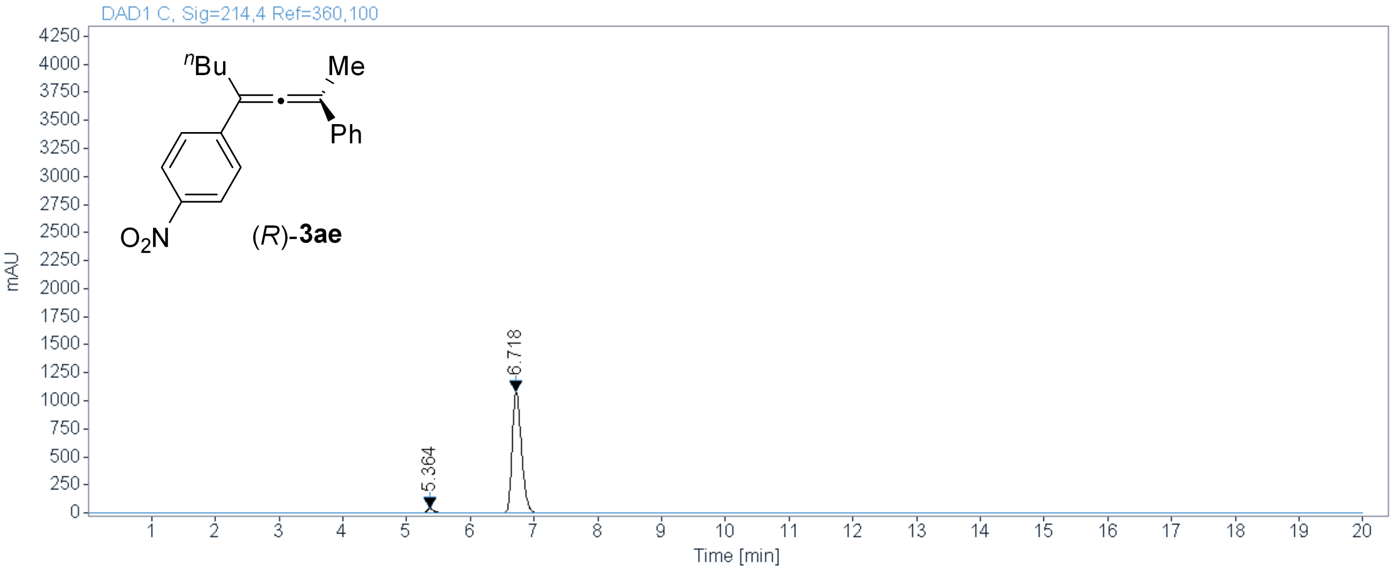

Signal: DAD1 C, Sig=214,4 Ref=360,100

RT [min]	Width [min]	Height	Area	Area%
5.364	0.1250	37.3975	280.4866	2.4706
6.718	0.1569	1086.2568	11072.6758	97.5294
		Sum	11353.1624	100.0000

Supplementary Figure 88. HPLC spectrum for (*R*)-**3ae**

Area Percent Report

Agilent Technologies

sample zwf-7-020-rac-OD-H-99.5-0.5-1.0-214

Data file: C:\Users\Public\Documents\ChemStation\1\Data\wgl 2019-09-03 07-35-18\039-P1-C2-zwf-7-020-rac.D

Acquisition Data:

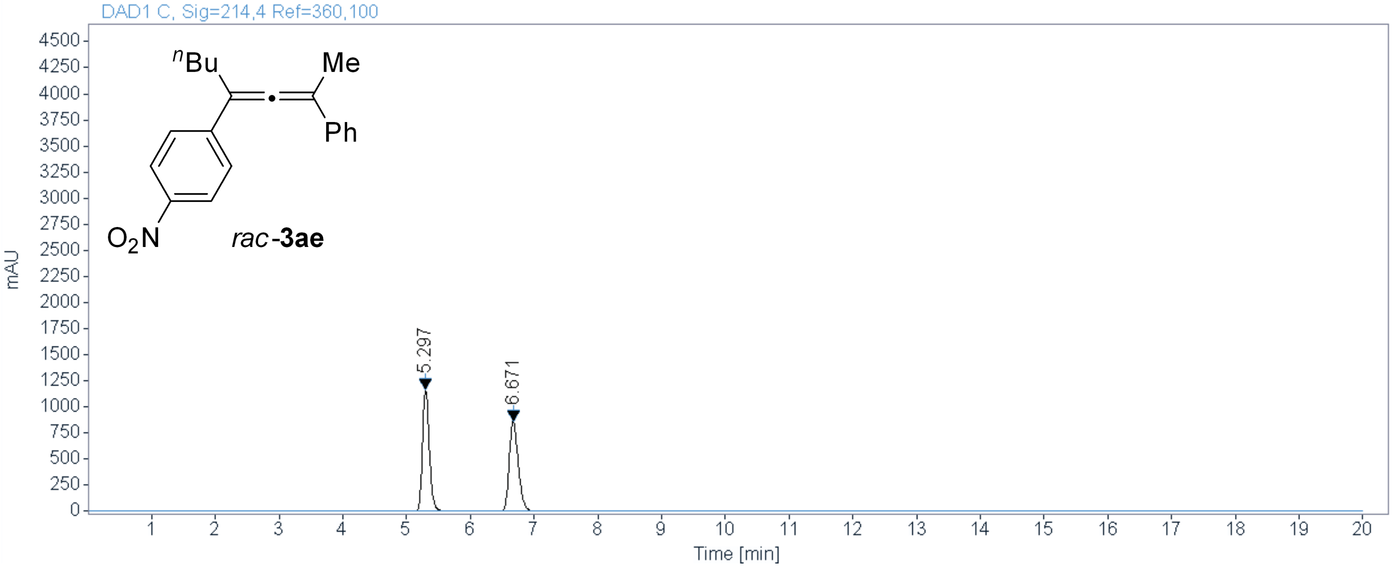

Signal: DAD1 C, Sig=214,4 Ref=360,100

RT [min]	Width [min]	Height	Area	Area%
5.297	0.1137	1170.5717	8622.6211	50.2488
6.671	0.1537	860.6385	8537.2246	49.7512
		Sum	17159.8457	100.0000

Supplementary Figure 89. HPLC spectrum for *rac*-**3ae**

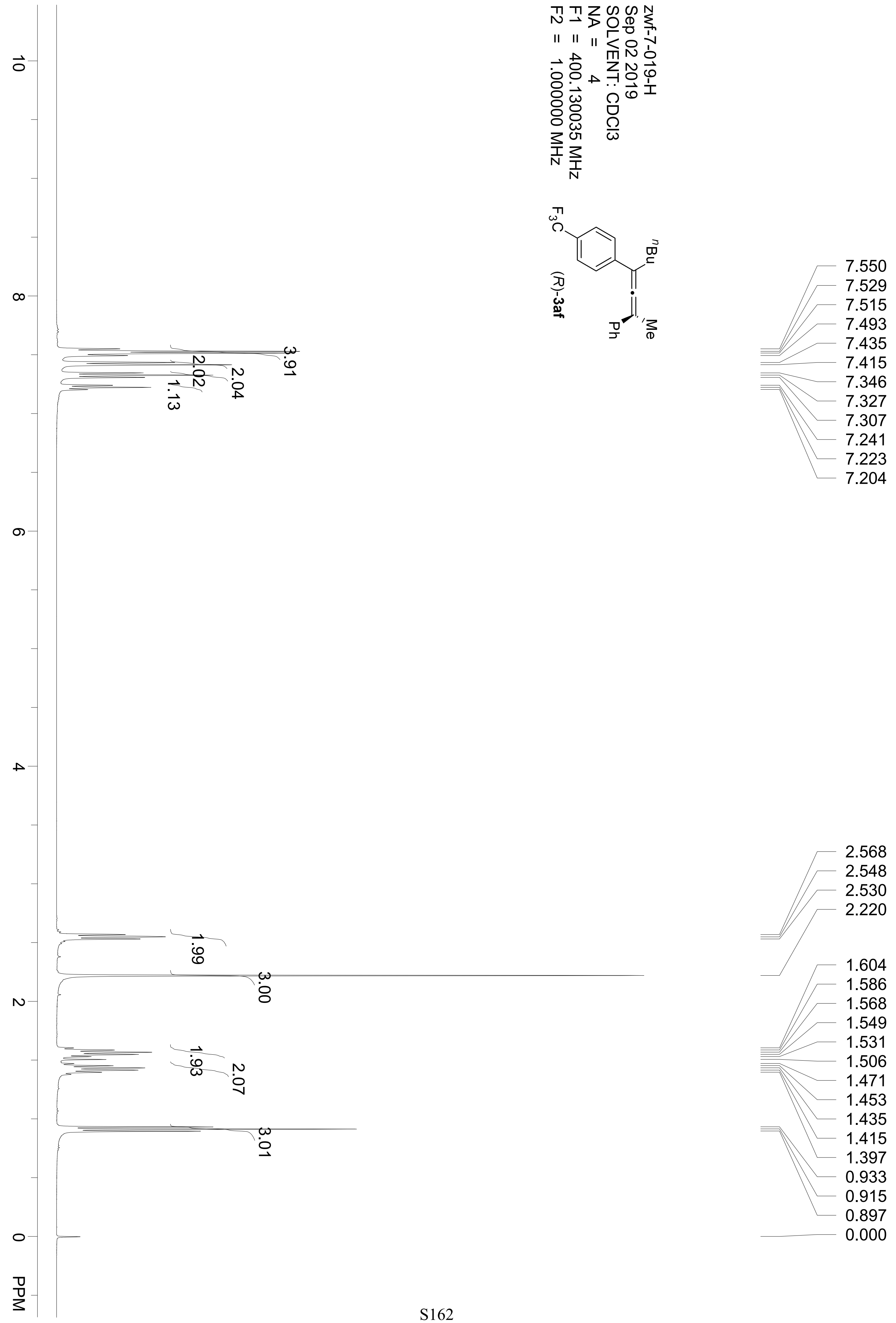

Supplementary Figure 90. ^{1}H NMR (400 MHz, $CDCl_3$) spectrum for (*R*)-**3af**

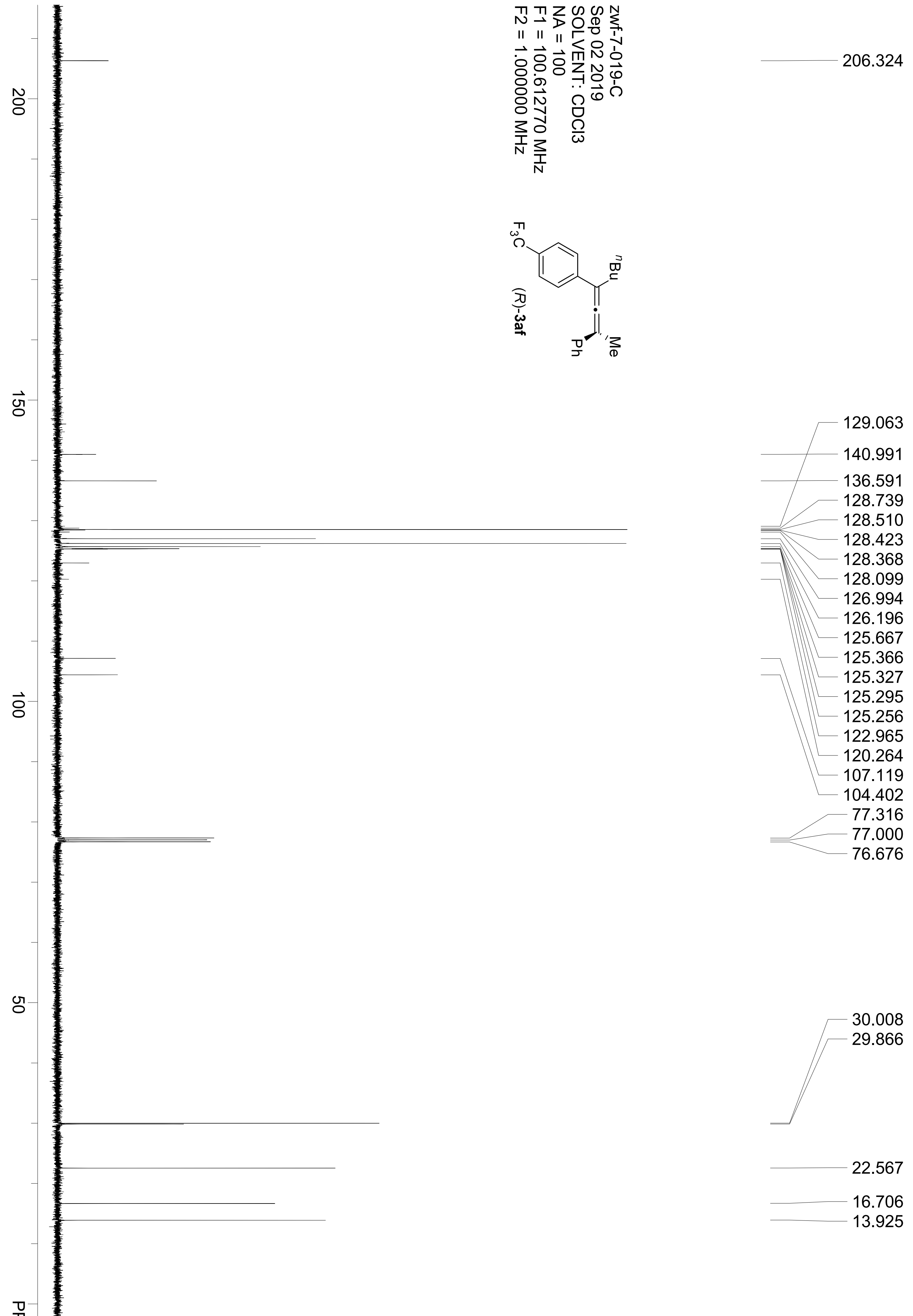

Supplementary Figure 91. ^{13}C NMR (100 MHz, $CDCl_3$) spectrum for (*R*)-**3af**

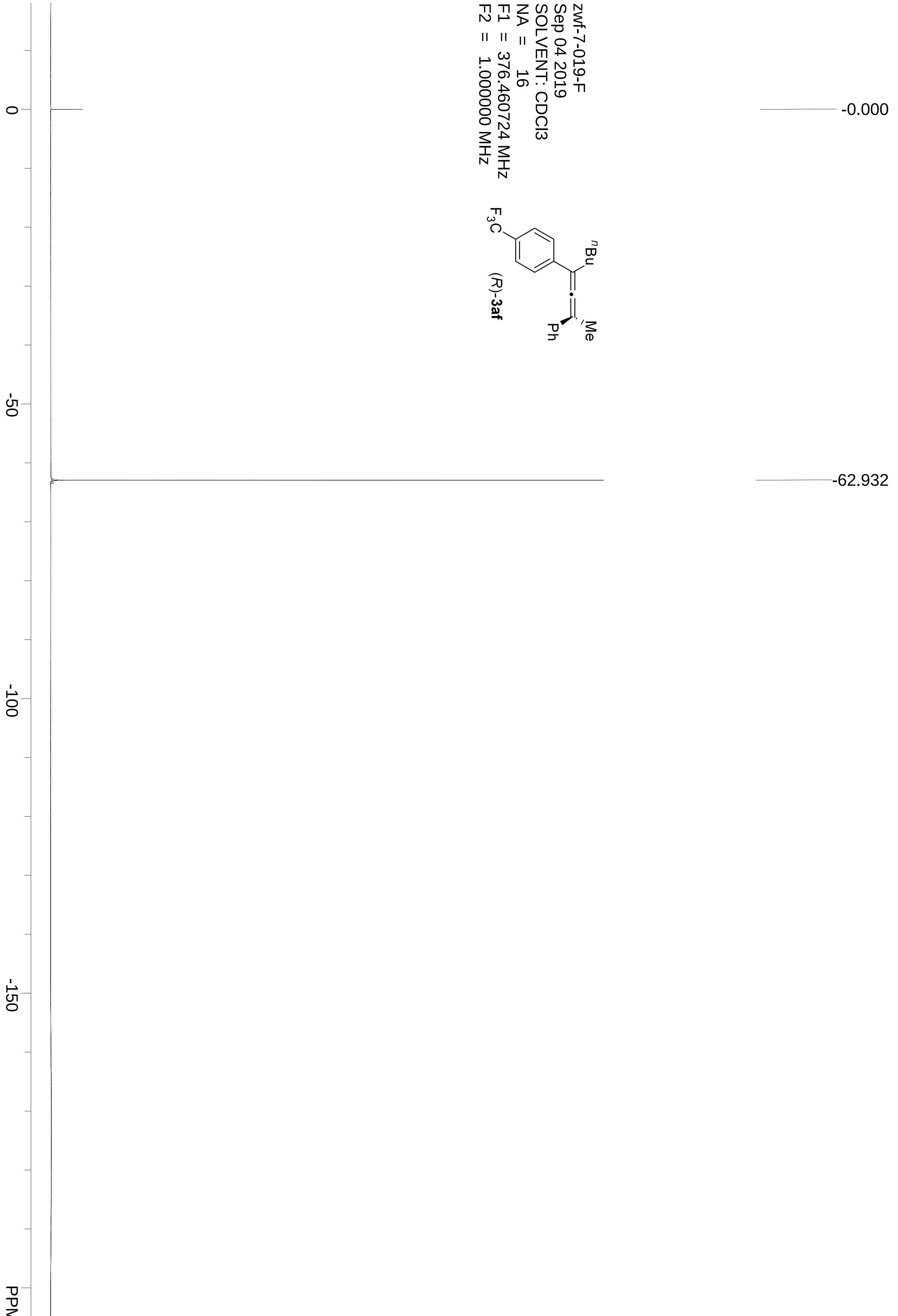

Supplementary Figure 92. ^{19}F NMR (376 MHz, $CDCl_3$) spectrum for (*R*)-**3af**

Area Percent Report

Agilent Technologies

sample zwf-7-019-AD-H-100-0-0.5-214

Data file: C:\Users\Public\Documents\ChemStation\1\Data\DEF_LC 2019-09-04 09-57-05\026-P1-C5-zwf-7-019.D

Acquisition Data:

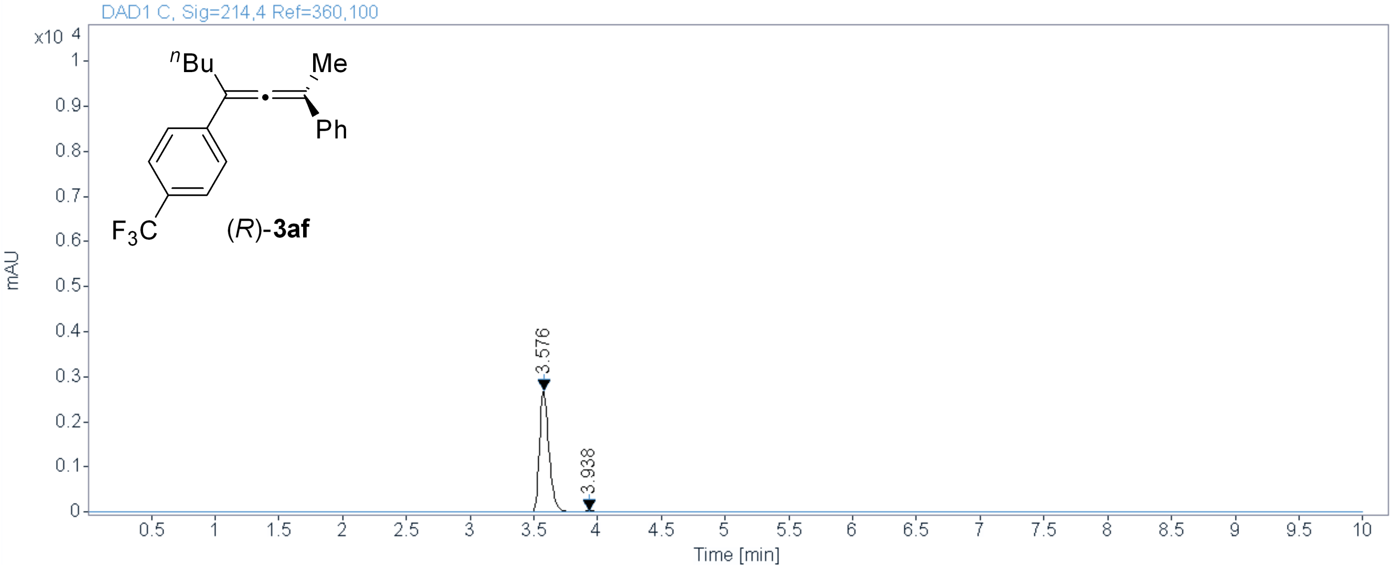

Signal: DAD1 C, Sig=214,4 Ref=360,100

RT [min]	Width [min]	Height	Area	Area%
3.576	0.0861	2709.9475	14006.0303	98.8433
3.938	0.0897	30.4626	163.9066	1.1567
		Sum	14169.9369	100.0000

Supplementary Figure 93. HPLC spectrum for (*R*)-**3af**

Area Percent Report

Agilent Technologies

sample zwf-7-019-rac-AD-H-100-0-0.5-214

Data file: C:\Users\Public\Documents\ChemStation\1\Data\DEF_LC 2019-09-04 09-57-05\027-P1-C6-zwf-7-019-rac.D

Acquisition Data:

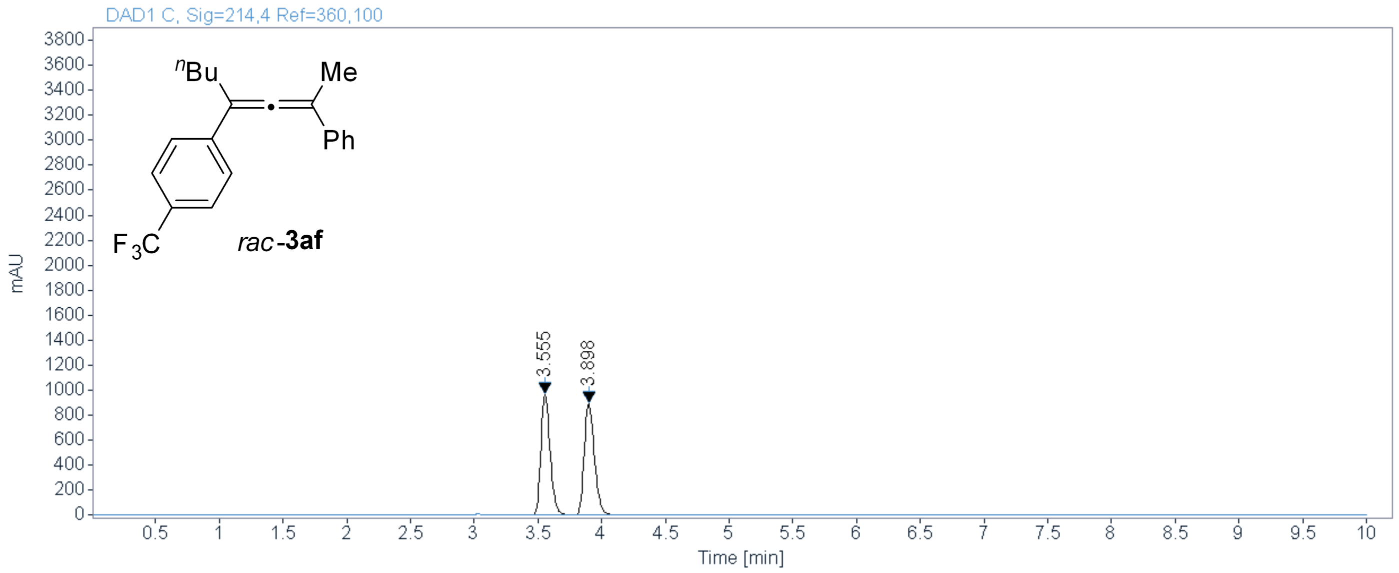

Signal: DAD1 C, Sig=214,4 Ref=360,100

RT [min]	Width [min]	Height	Area	Area%
3.555	0.0782	979.2198	4908.4673	50.0393
3.898	0.0837	897.4544	4900.7495	49.9607
		Sum	9809.2168	100.0000

Supplementary Figure 94. HPLC spectrum for *rac*-**3af**

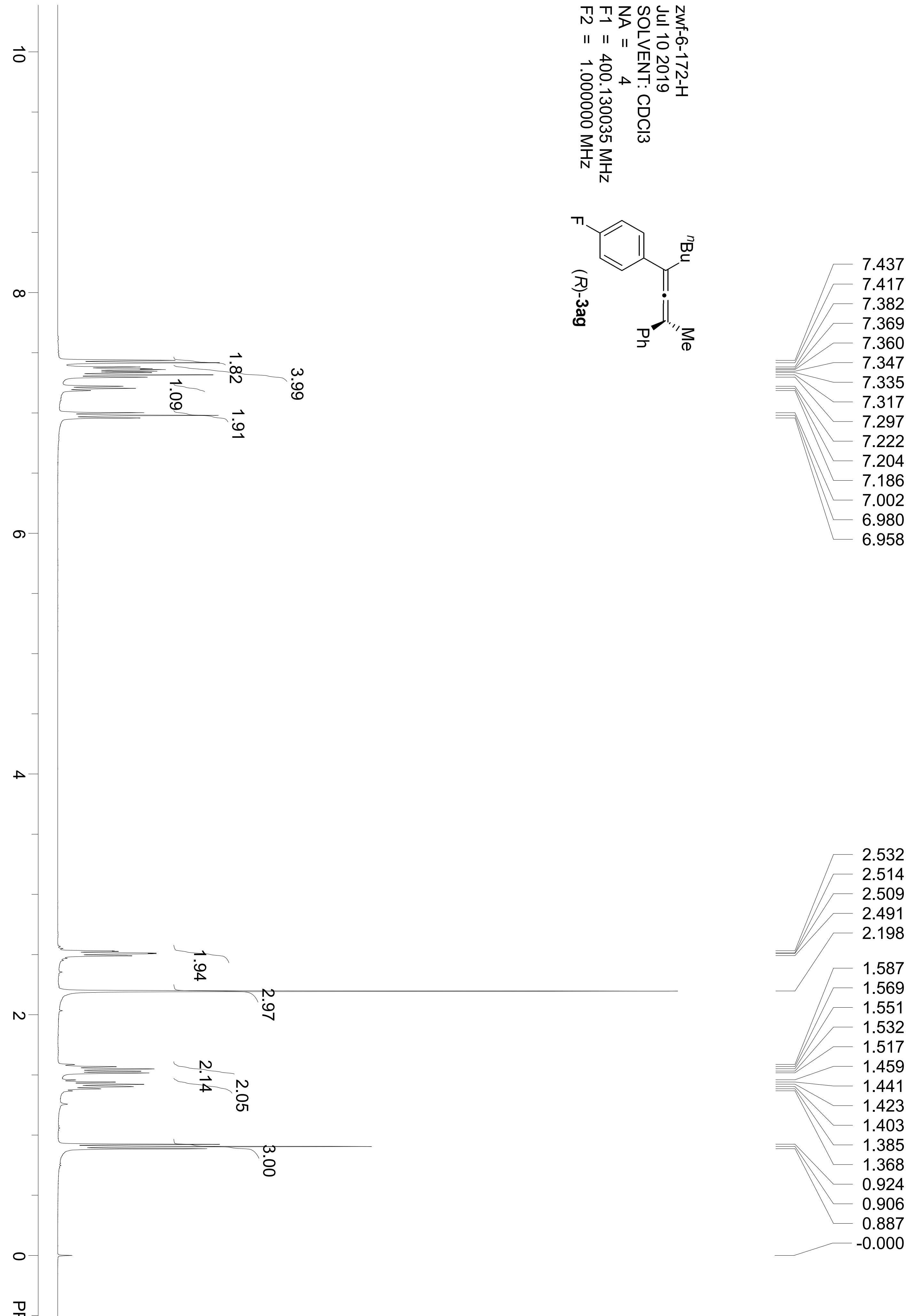

Supplementary Figure 95. ^{1}H NMR (400 MHz, $CDCl_3$) spectrum for (*R*)-**3ag**

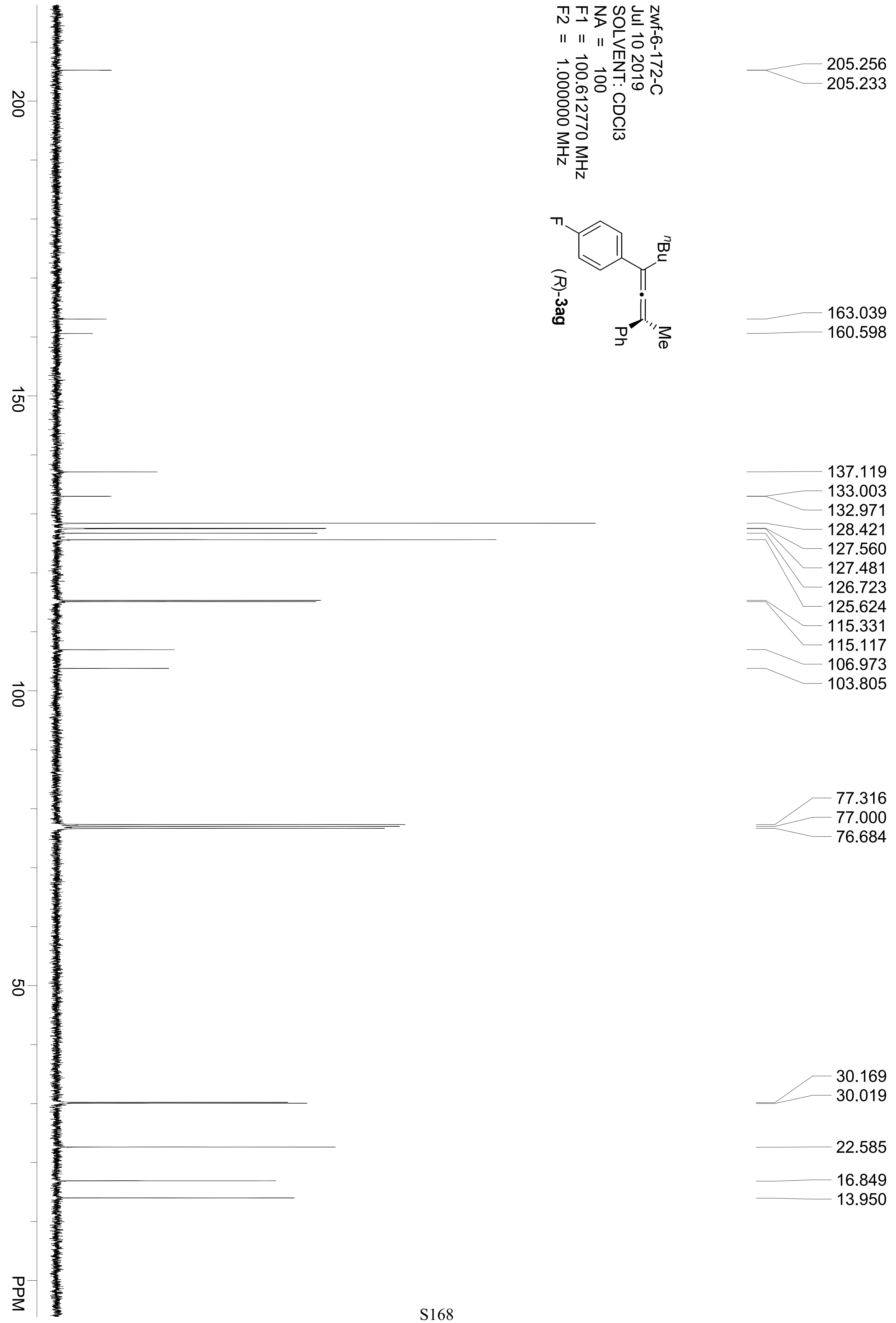

Supplementary Figure 96. ^{13}C NMR (100 MHz, $CDCl_3$) spectrum for (*R*)-**3ag**

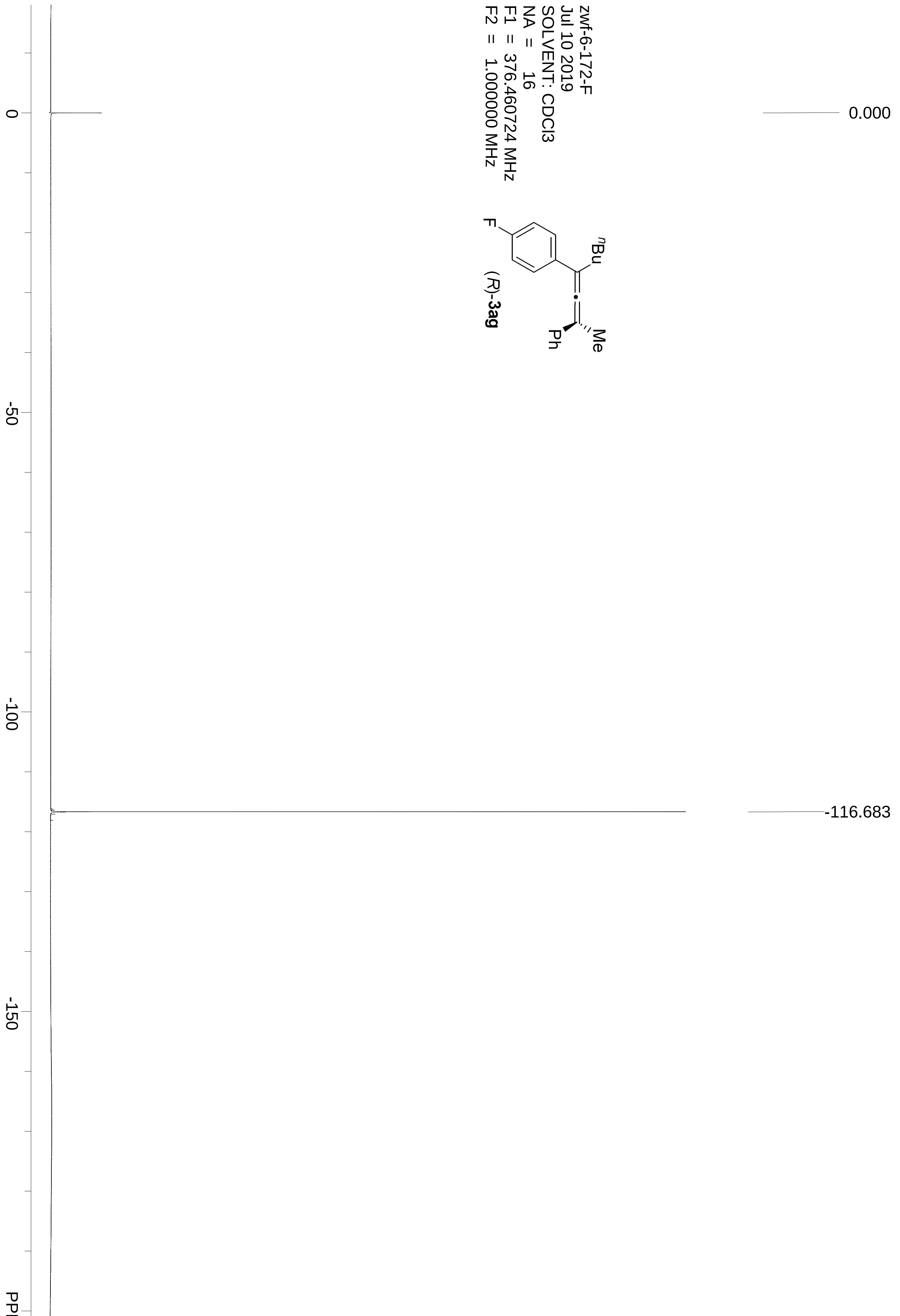

Supplementary Figure 97. ^{19}F NMR (376 MHz, $CDCl_3$) spectrum for (*R*)-**3ag**

Empower™ 3 SOFTWARE

Default Individual Report

SAMPLE INFORMATION

Sample Name:	zwf-7-029	Acquired By:	System
Sample Type:	Unknown	Sample Set Name:	
Vial:	1:F,7	Acq. Method Set:	upc_pda_2019m
Injection #:	1	Processing Method	TEST
Injection Volume:	2.00 ul	Channel Name:	PDA Ch2 214nm@4.8nm
Run Time:	35.0 Minutes	Proc. Chnl. Descr.:	PDA Ch2 214nm@4.8nm
Date Acquired:	9/11/2019 10:39:47 AM CST		
Date Processed:	9/12/2019 1:24:05 PM CST		

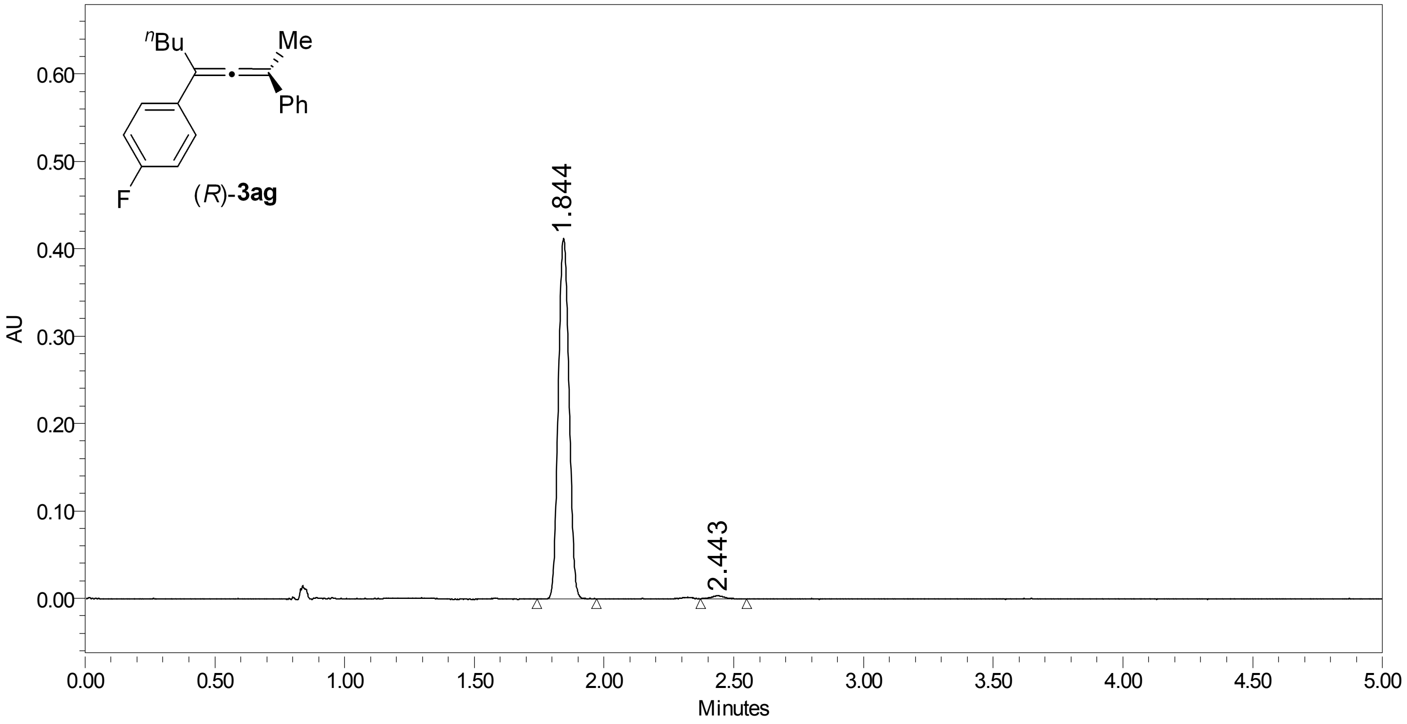

	RT	Peak Type	Height	Width (sec)	Area	% Area
1	1.844	Unknown	412415	13.800	1180860	98.87
2	2.443	Unknown	3585	10.650	13545	1.13

Reported by User: System
Report Method: Default Individual Report
Report Method II 17264
Page: 1 of 1

Project Name: TEST
Date Printed:
9/12/2019
1:41:40 PM PRC

Supplementary Figure 98. SFC spectrum for (*R*)-**3ag**

Default Individual Report

SAMPLE INFORMATION

Sample Name:	zwf-6-166	Acquired By:	System
Sample Type:	Unknown	Sample Set Name:	
Vial:	1:F,6	Acq. Method Set:	upc_pda_2019m
Injection #:	1	Processing Method	TEST
Injection Volume:	2.00 ul	Channel Name:	PDA Ch2 214nm@4.8nm
Run Time:	35.0 Minutes	Proc. Chnl. Descr.:	PDA Ch2 214nm@4.8nm
Date Acquired:	9/11/2019 10:23:18 AM CST		
Date Processed:	9/12/2019 1:23:08 PM CST		

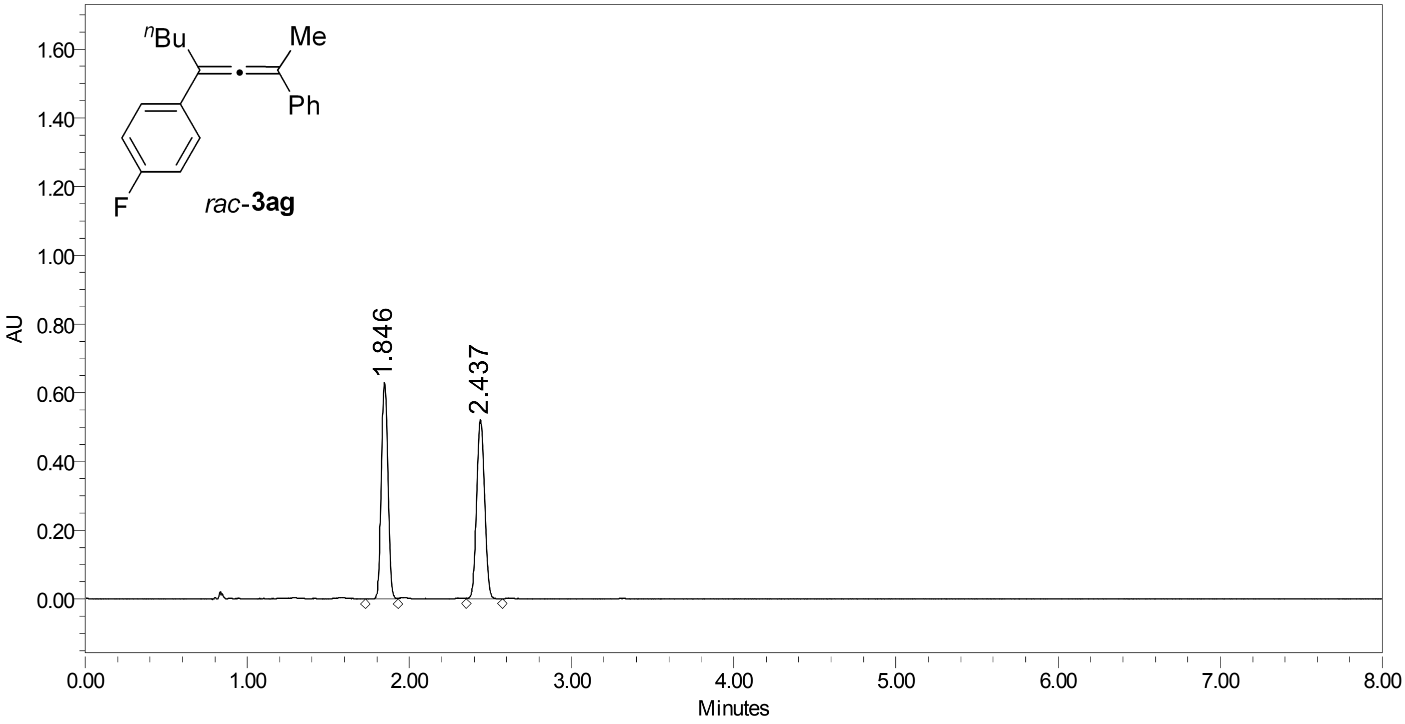

	RT	Peak Type	Height	Width (sec)	Area	% Area
1	1.846	Unknown	629441	12.050	1808223	49.93
2	2.437	Unknown	522658	13.400	1813286	50.07

Supplementary Figure 99. SFC spectrum for *rac*-**3ag**

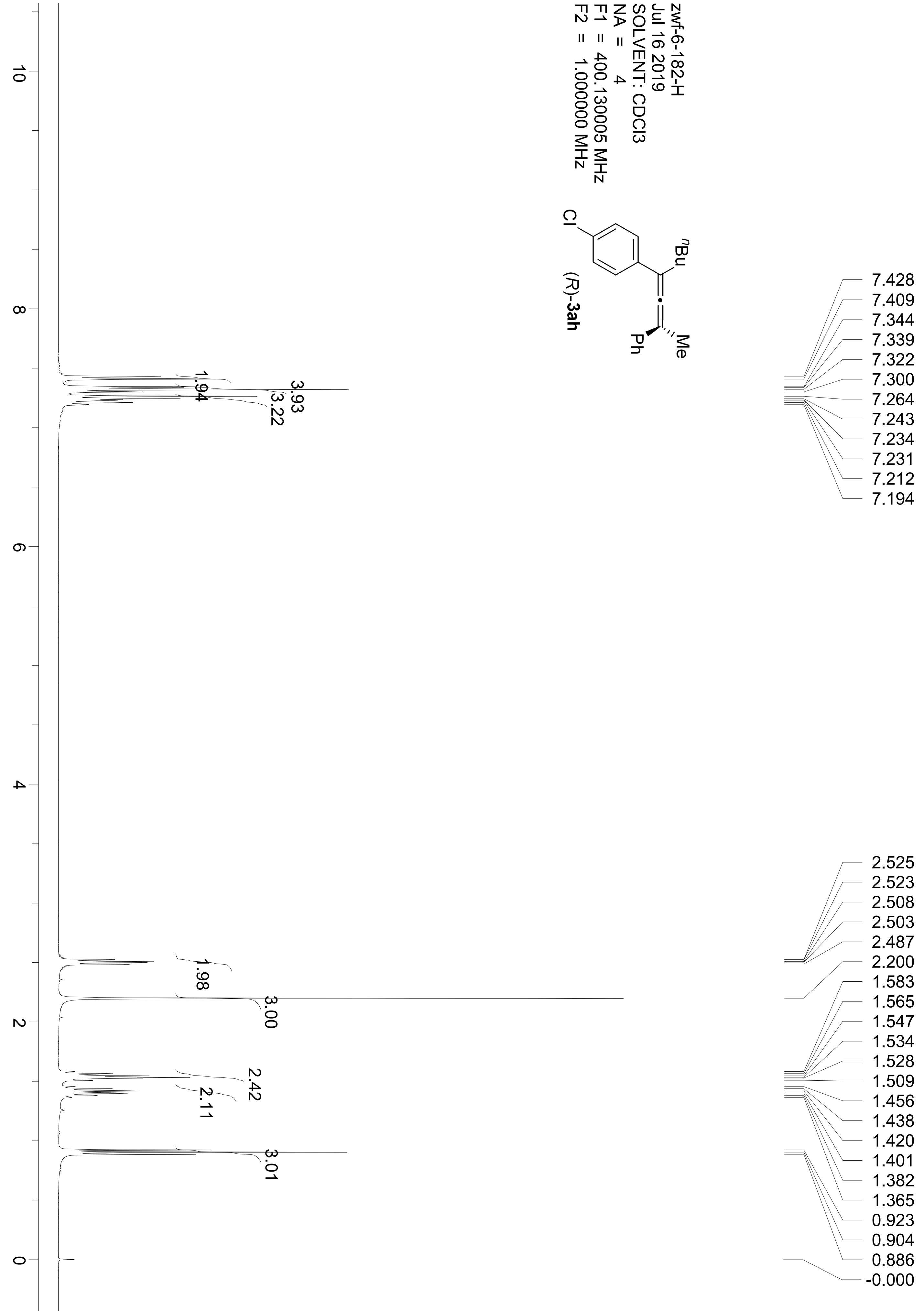

Supplementary Figure 100. ^{1}H NMR (400 MHz, $CDCl_3$) spectrum for (*R*)-**3ah**

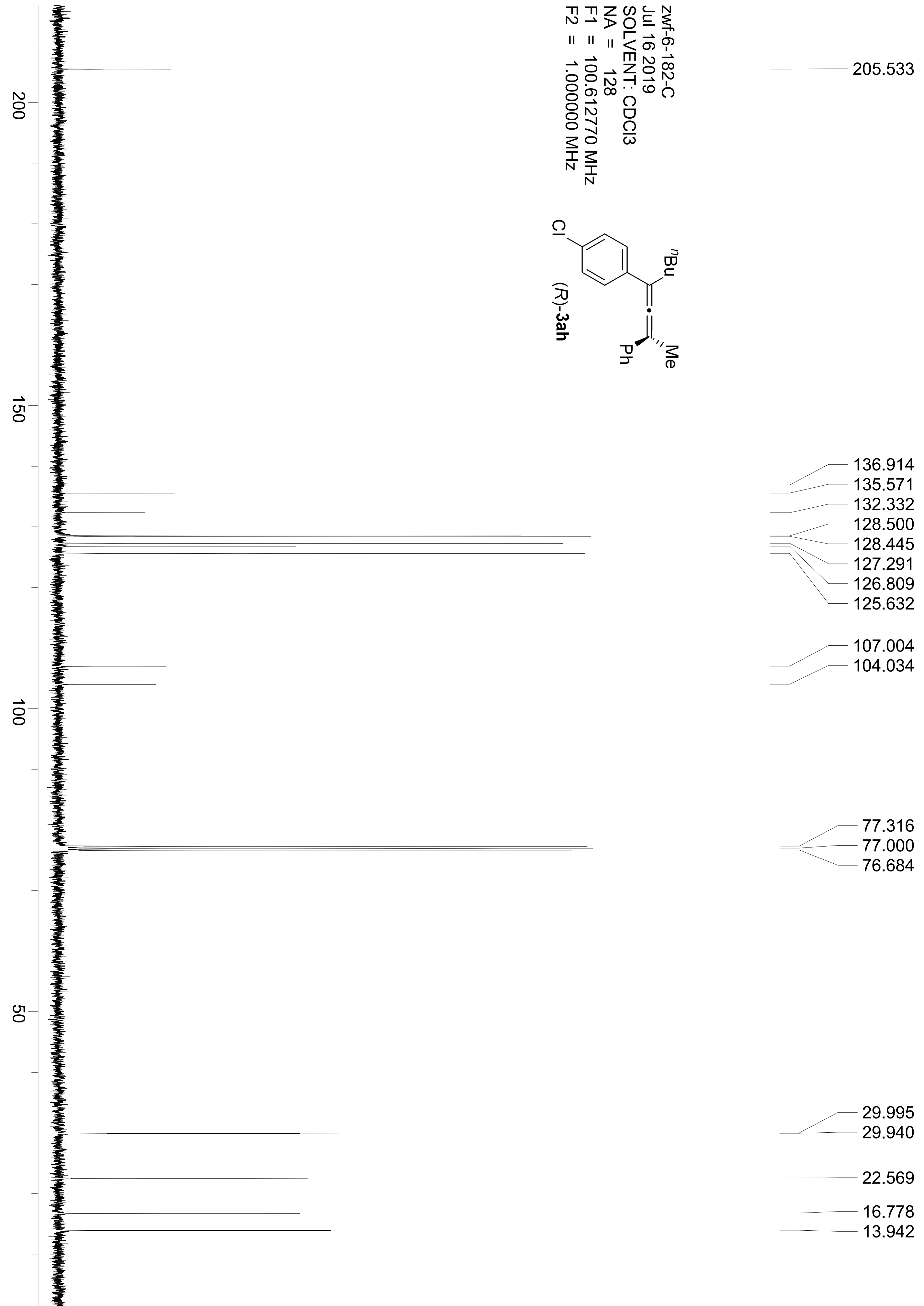

Supplementary Figure 101. ^{13}C NMR (100 MHz, $CDCl_3$) spectrum for (*R*)-**3ah**

Empower™ 3 SOFTWARE

Default Individual Report

SAMPLE INFORMATION

Sample Name:	zwf-7-030	Acquired By:	System
Sample Type:	Unknown	Sample Set Name:	
Vial:	1:F,5	Acq. Method Set:	upc_pda_2019m
Injection #:	1	Processing Method	TEST
Injection Volume:	2.00 ul	Channel Name:	PDA Ch2 214nm@4.8nm
Run Time:	35.0 Minutes	Proc. Chnl. Descr.:	PDA Ch2 214nm@4.8nm
Date Acquired:	9/11/2019 10:10:50 AM CST		
Date Processed:	9/12/2019 1:22:21 PM CST		

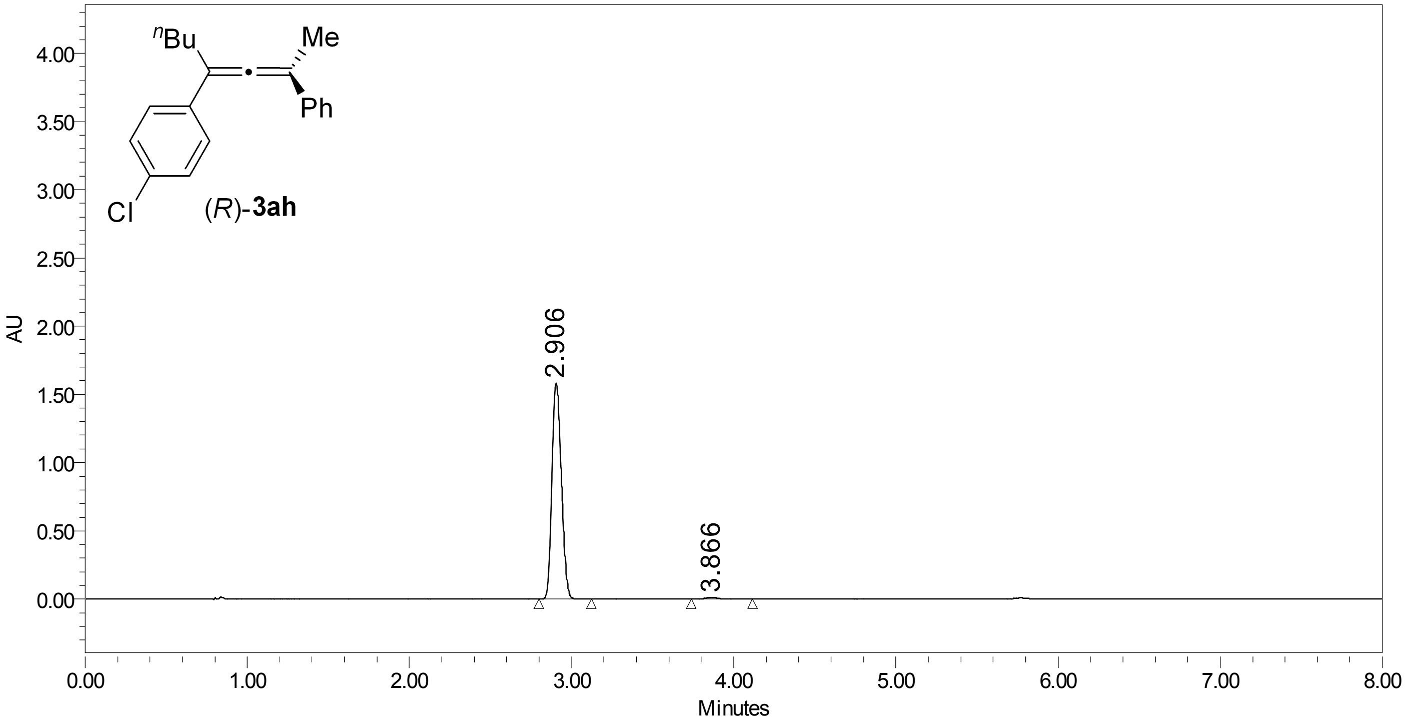

	RT	Peak Type	Height	Width (sec)	Area	% Area
1	2.906	Unknown	1583845	19.450	6367196	99.03
2	3.866	Unknown	11367	22.750	62346	0.97

Reported by User: System
Report Method: Default Individual Report
Report Method II 17262
Page: 1 of 1

Project Name: TEST
Date Printed:
9/12/2019
1:40:24 PM PRC

Supplementary Figure 102. SFC spectrum for (*R*)-**3ah**

Default Individual Report

SAMPLE INFORMATION

Sample Name:	zwf-6-173-rac	Acquired By:	System
Sample Type:	Unknown	Sample Set Name:	
Vial:	1:F,4	Acq. Method Set:	upc_pda_2019m
Injection #:	1	Processing Method	TEST
Injection Volume:	2.00 ul	Channel Name:	PDA Ch2 214nm@4.8nm
Run Time:	35.0 Minutes	Proc. Chnl. Descr.:	PDA Ch2 214nm@4.8nm
Date Acquired:	9/11/2019 10:00:31 AM CST		
Date Processed:	9/12/2019 1:21:10 PM CST		

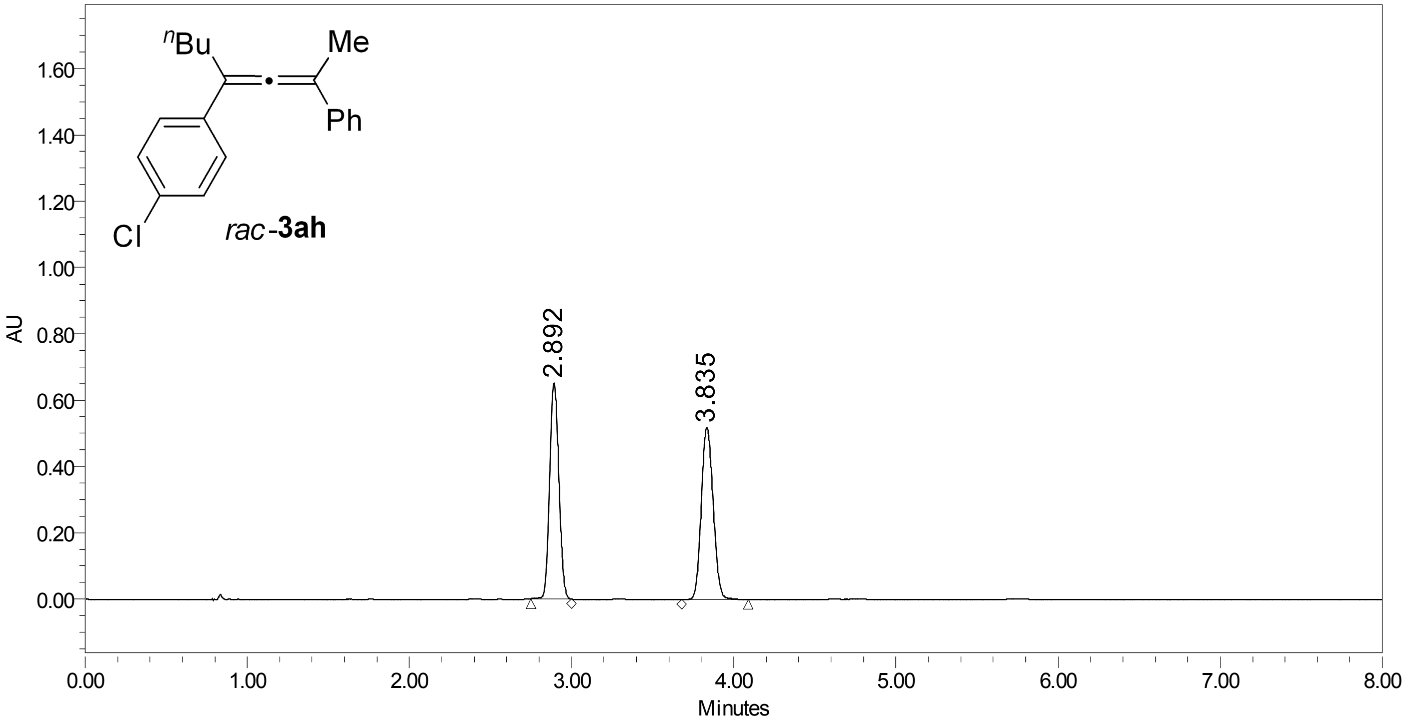

	RT	Peak Type	Height	Width (sec)	Area	% Area
1	2.892	Unknown	651074	15.000	2572779	49.88
2	3.835	Unknown	517549	24.550	2585303	50.12

Supplementary Figure 103. SFC spectrum for *rac*-**3ah**

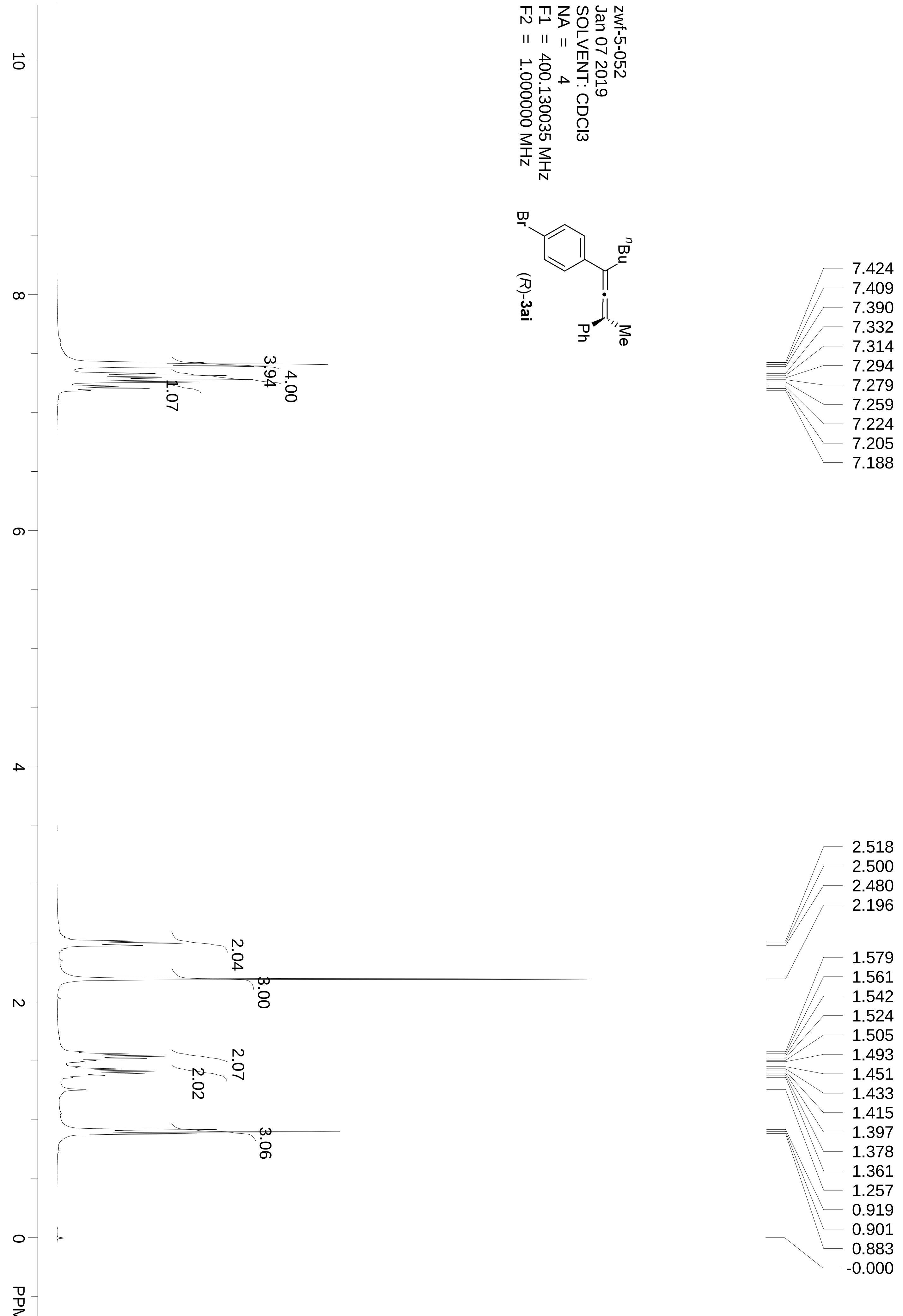

Supplementary Figure 104. ^{1}H NMR (400 MHz, $CDCl_3$) spectrum for (*R*)-**3ai**

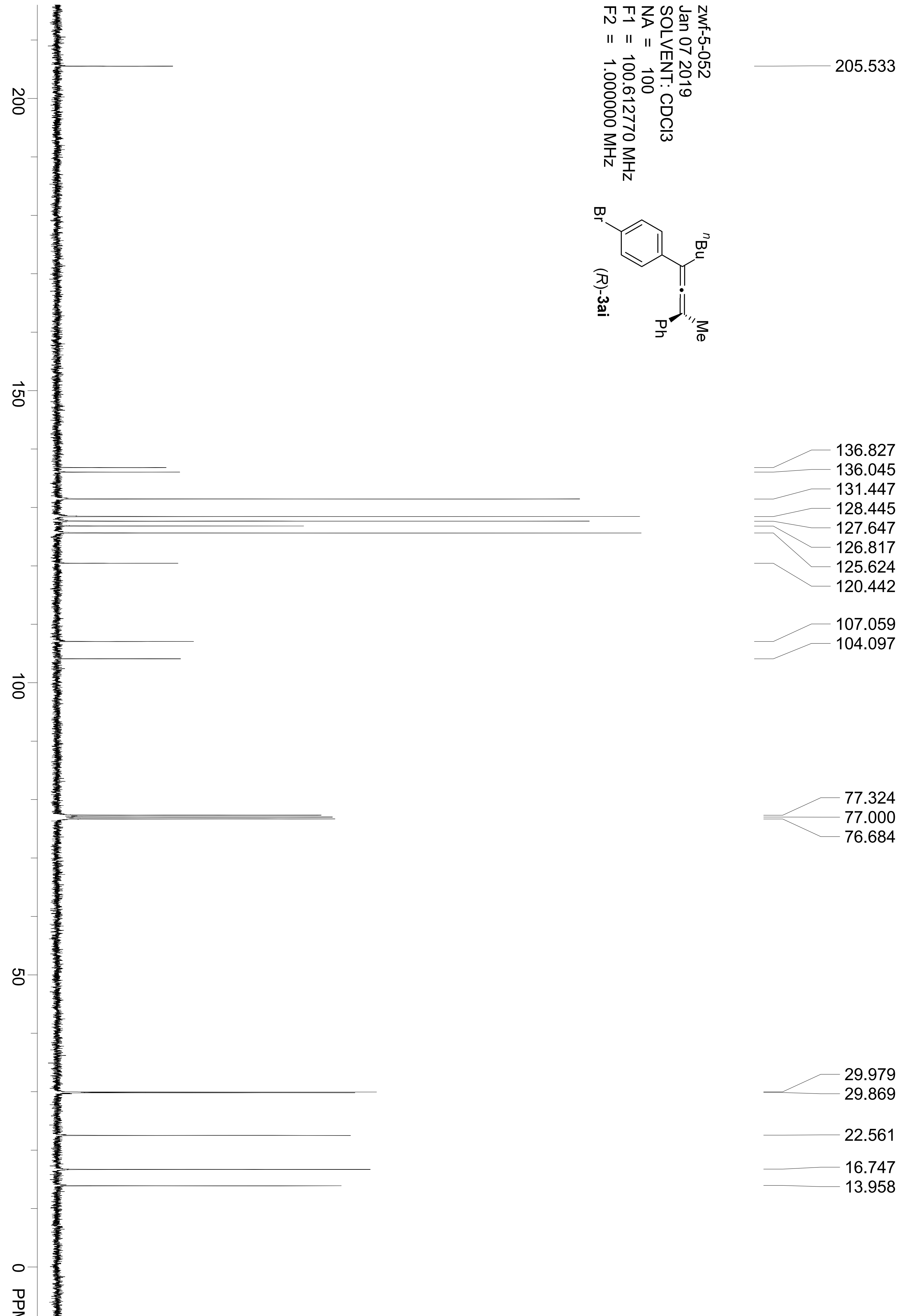

Supplementary Figure 105. ^{13}C NMR (100 MHz, $CDCl_3$) spectrum for (*R*)-**3ai**

Area Percent Report

Agilent Technologies

sample zwf-7-031-OD-H-100-0-0.5-214

Data file: C:\Users\Public\Documents\ChemStation\1\Data\zwf-allenioc acid_LC 2019-09-08 15-29-17\004-P1-C9-zwf-7-031.D

Acquisition Data:

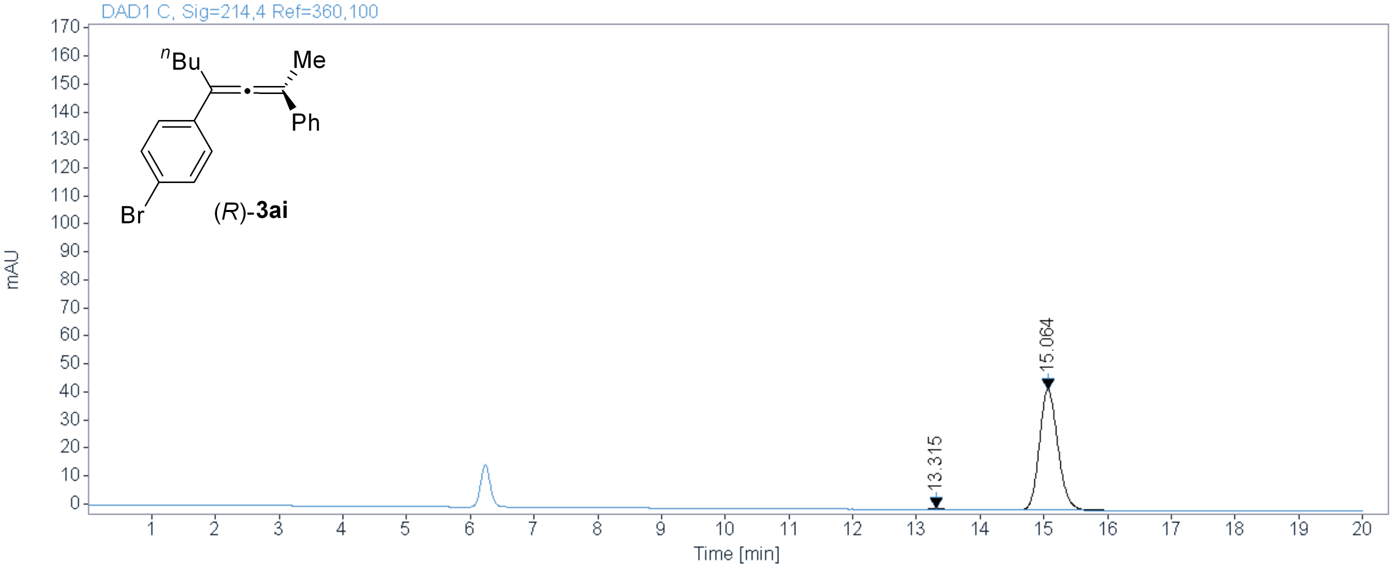

Signal: DAD1 C, Sig=214,4 Ref=360,100

RT [min]	Width [min]	Height	Area	Area%
13.315	0.2941	0.4088	7.2136	0.8213
15.064	0.3112	43.2100	871.0535	99.1787
		Sum	878.2672	100.0000

Supplementary Figure 106. HPLC spectrumfor (*R*)-**3ai**

Area Percent Report

sample zwf-7-031-rac-OD-H-100-0-0.5-214

Data file: C:\Users\Public\Documents\ChemStation\1\Data\zwf-allenioc acid_LC 2019-09-08 15-29-17\005-P1-C10-zwf-7-031-rac.D

Acquisition Data:

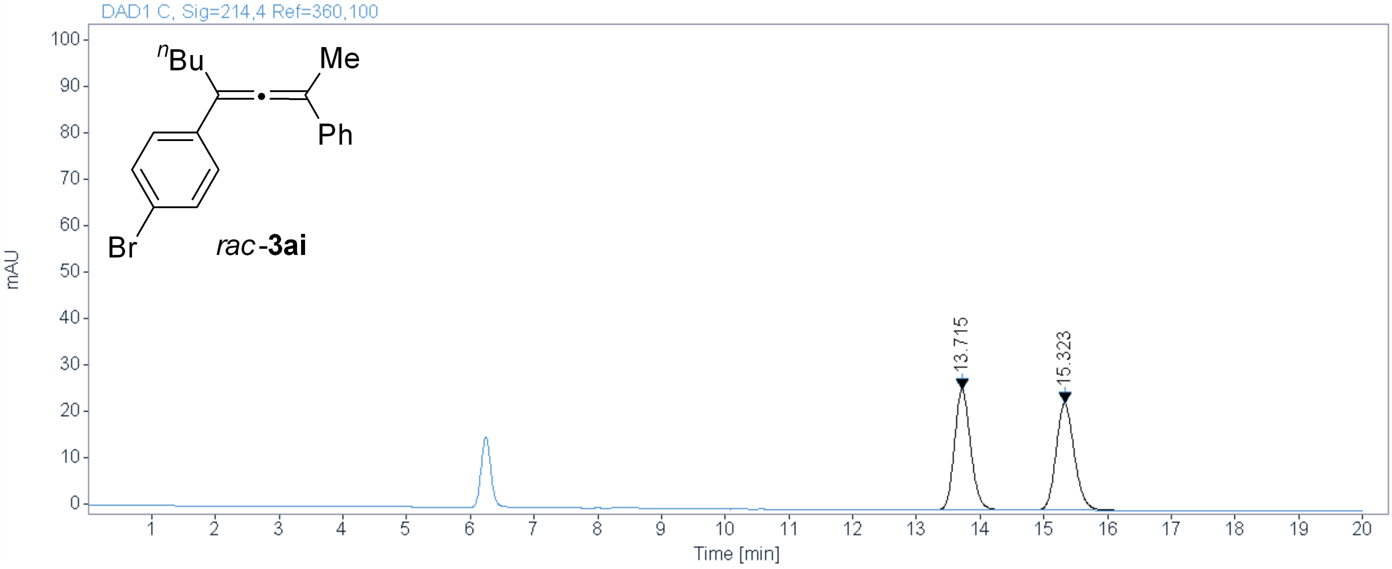

Signal: DAD1 C, Sig=214,4 Ref=360,100

RT [min]	Width [min]	Height	Area	Area%
13.715	0.2722	25.9736	459.0972	49.7412
15.323	0.3107	23.0541	463.8752	50.2588
		Sum	922.9725	100.0000

Supplementary Figure 107. HPLC spectrum for *rac*-**3ai**

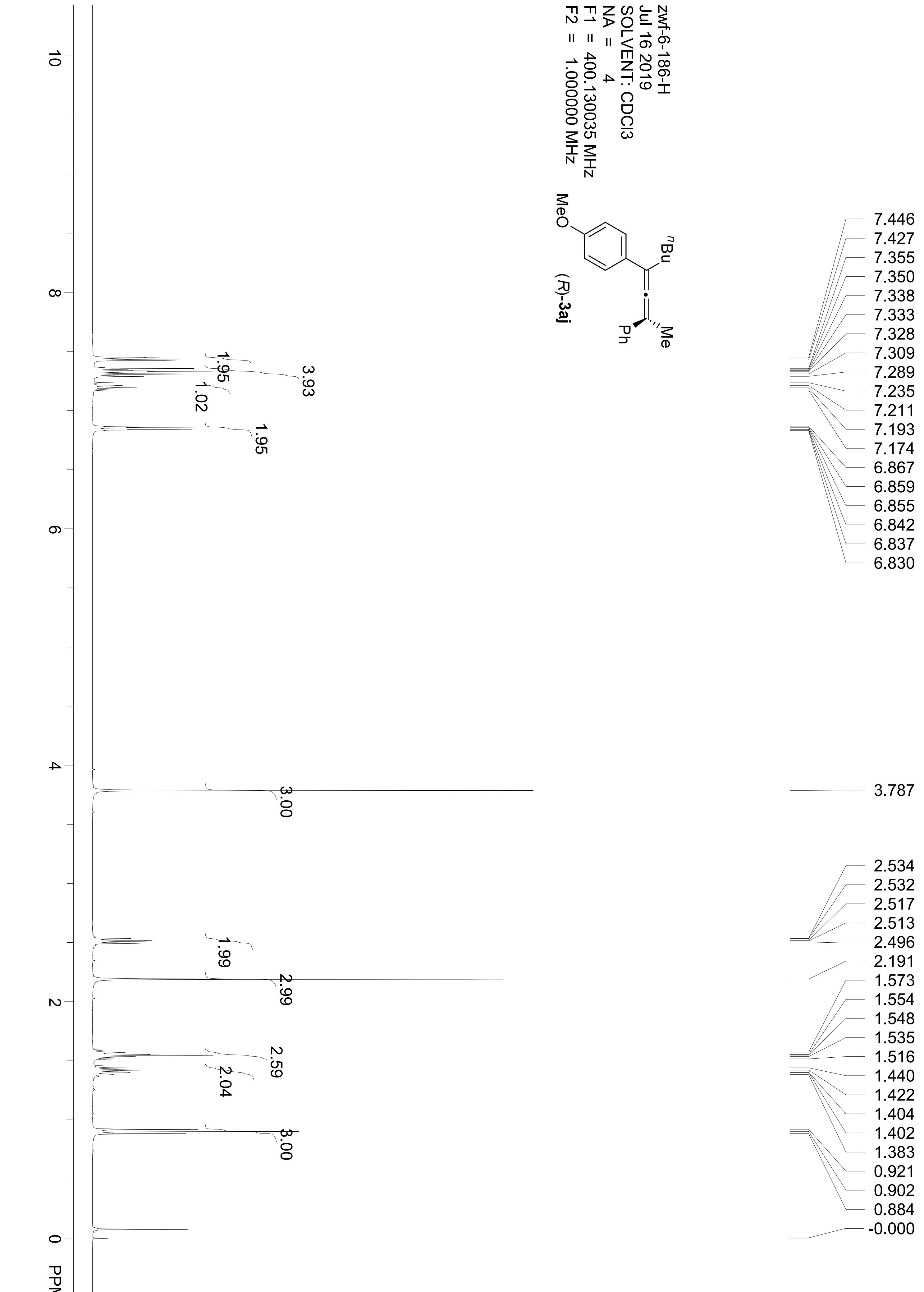

Supplementary Figure 108. ^{1}H NMR (400 MHz, $CDCl_3$) spectrum for (*R*)-**3aj**

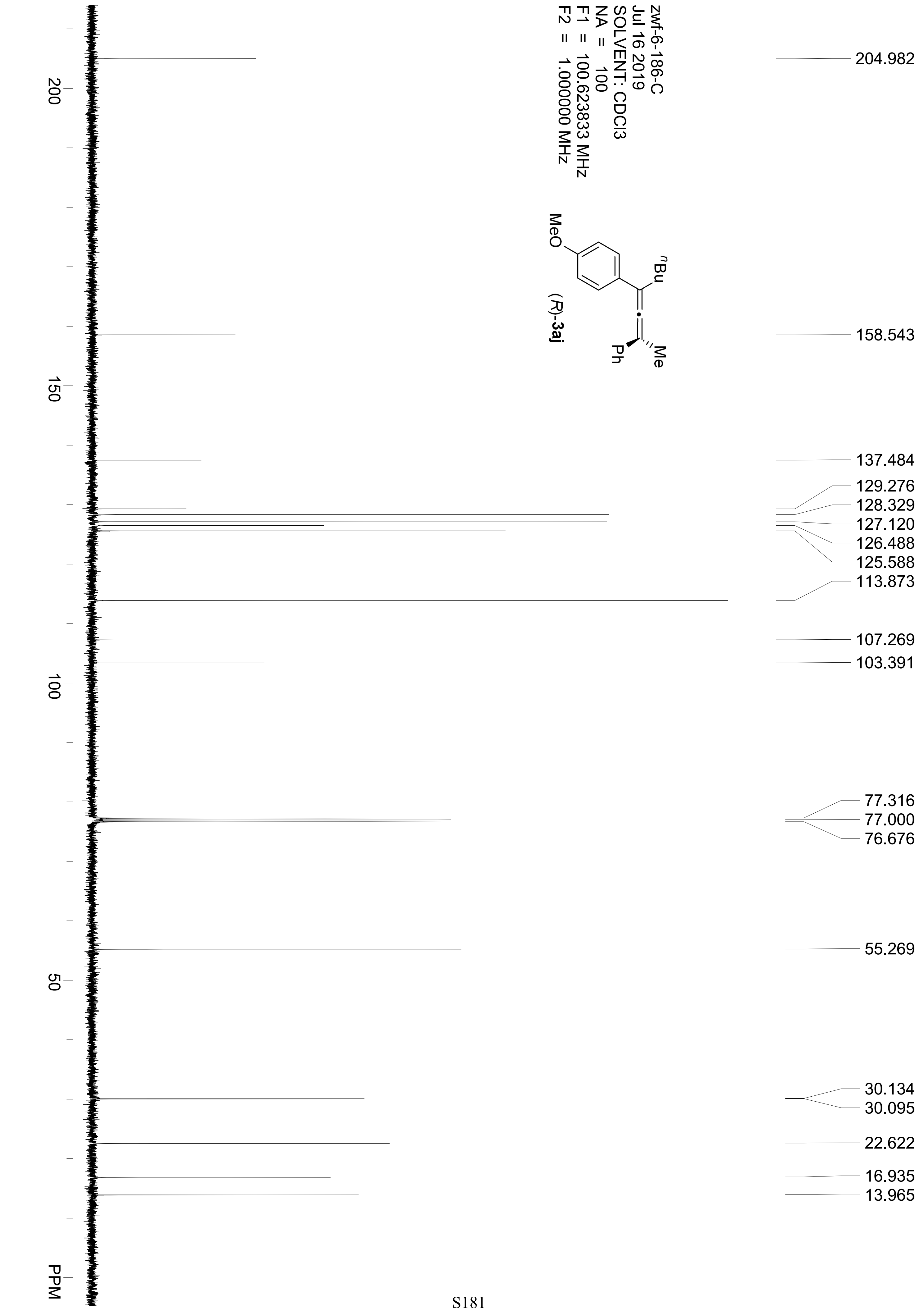

Supplementary Figure 109. ^{13}C NMR (100 MHz, $CDCl_3$) spectrum for (*R*)-**3aj**

Area Percent Report

sample zwf-7-032-OD-H-99.5-0.5-1.0-214

Data file: C:\Users\Public\Documents\ChemStation\1\Data\zwf-allenioc acid_LC 2019-09-07 14-54-21\023-P1-B1-zwf-7-032.D

Acquisition Data:

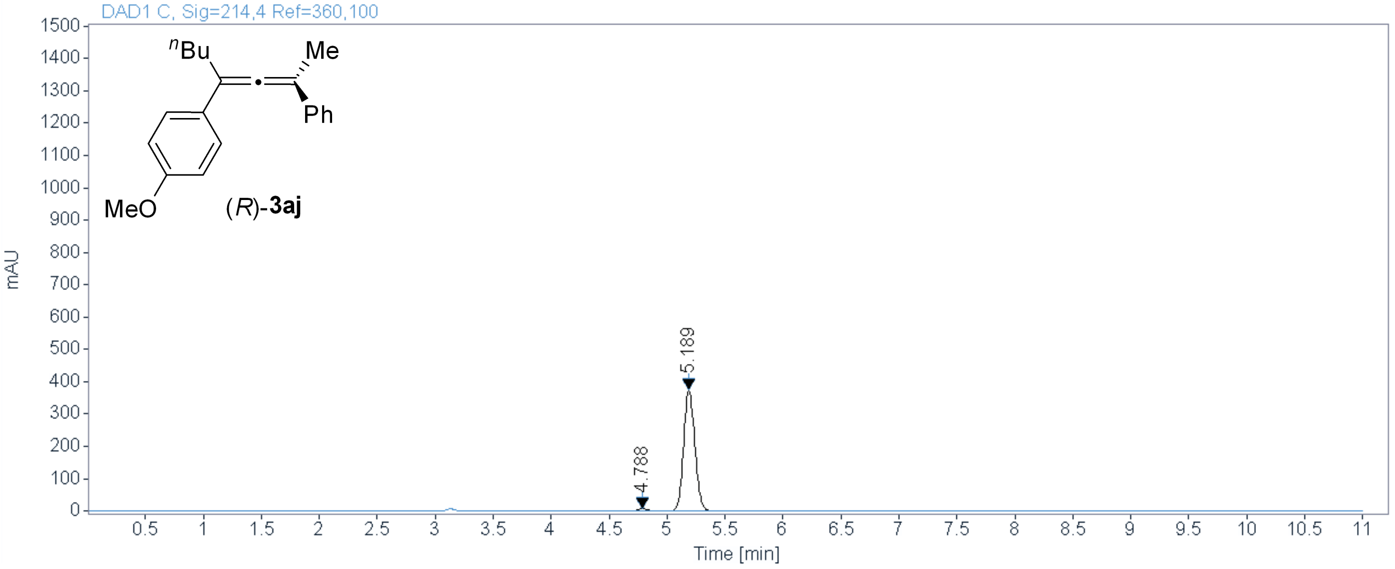

Signal: DAD1 C, Sig=214,4 Ref=360,100

RT [min]	Width [min]	Height	Area	Area%
4.788	0.0955	7.6677	46.9933	1.8103
5.189	0.1048	377.4749	2548.8826	98.1897
		Sum	2595.8759	100.0000

Supplementary Figure 110. HPLC spectrum for (*R*)-**3aj**

Area Percent Report

Agilent Technologies

sample zwf-7-032-rac-OD-H-99.5-0.5-1.0-214

Data file: C:\Users\Public\Documents\ChemStation\1\Data\zwf-allenioc acid_LC 2019-09-07 14-54-21\024-P1-B2-zwf-7-032-rac.D

Acquisition Data:

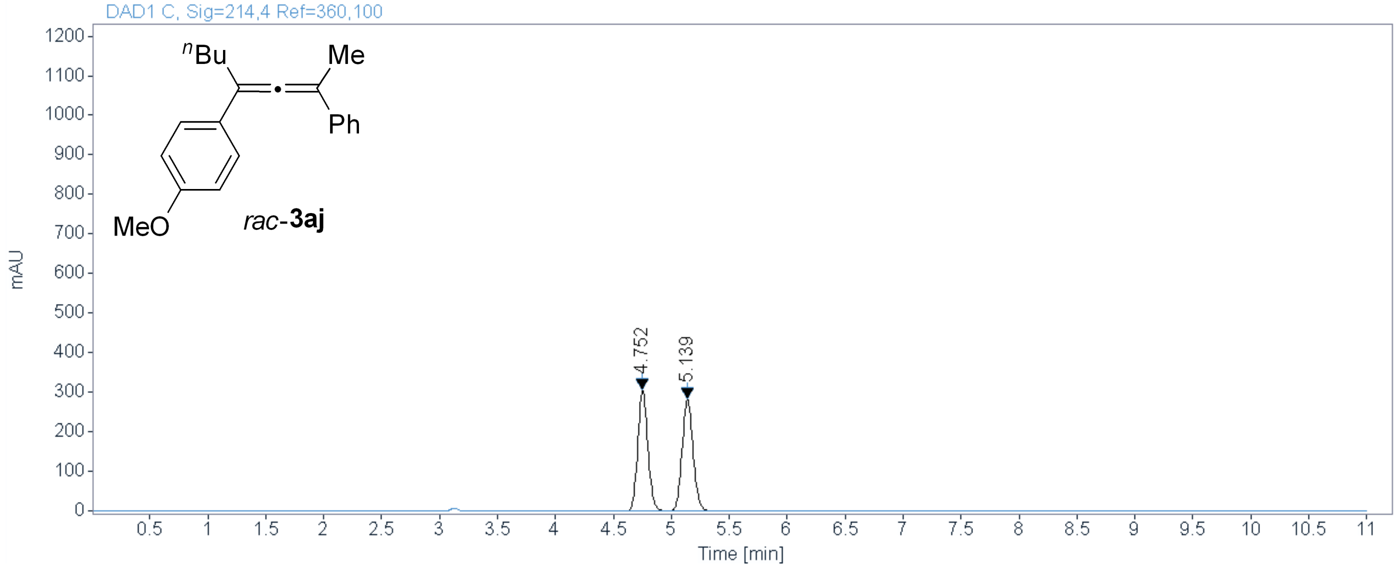

Signal: DAD1 C, Sig=214,4 Ref=360,100

RT [min]	Width [min]	Height	Area	Area%
4.752	0.0930	307.8929	1873.4294	49.9742
5.139	0.1030	284.0324	1875.3632	50.0258
		Sum	3748.7926	100.0000

Supplementary Figure 111. HPLC spectrum for *rac*-**3aj**

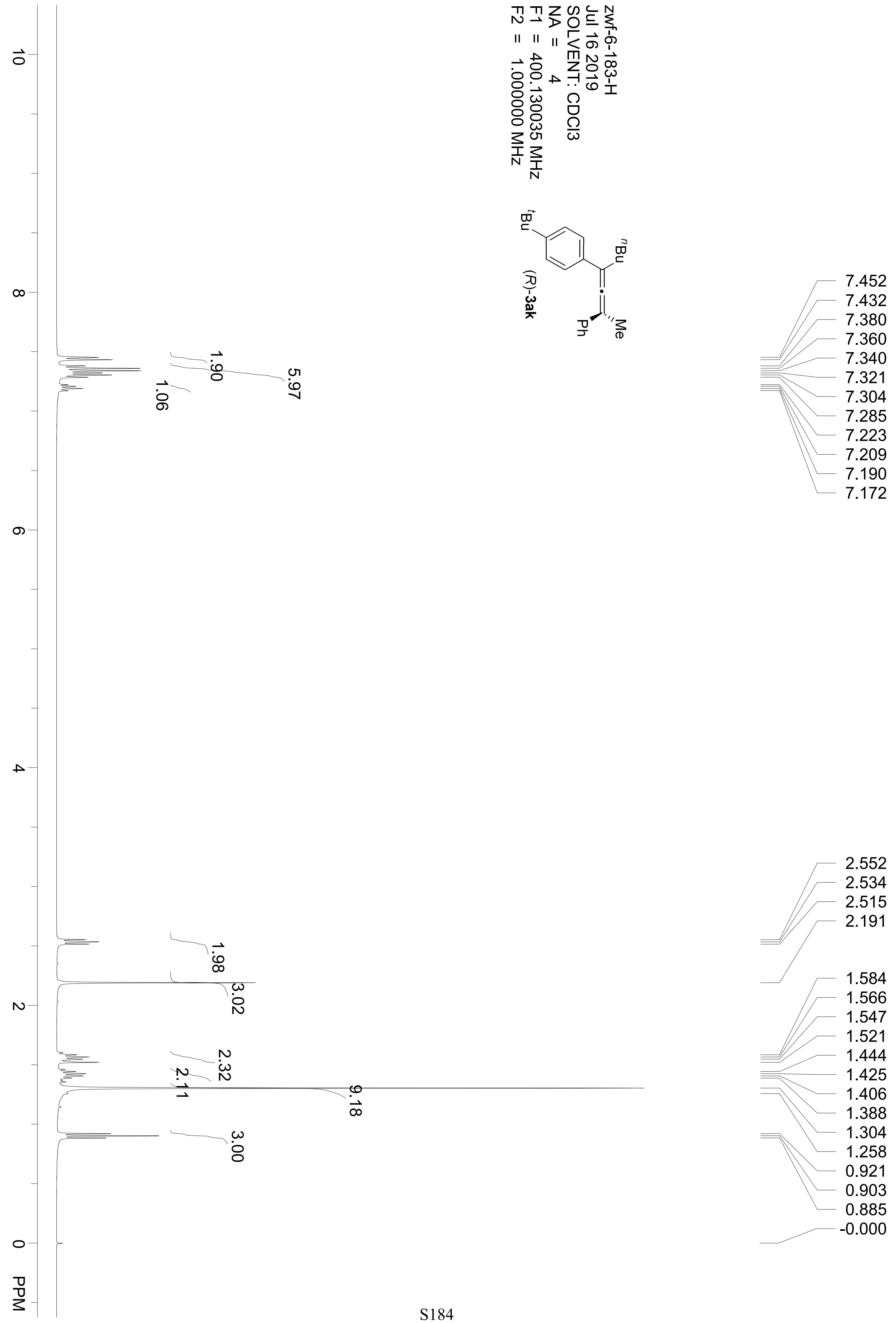

Supplementary Figure 112. ^{1}H NMR (400 MHz, $CDCl_3$) spectrum for (*R*)-**3ak**

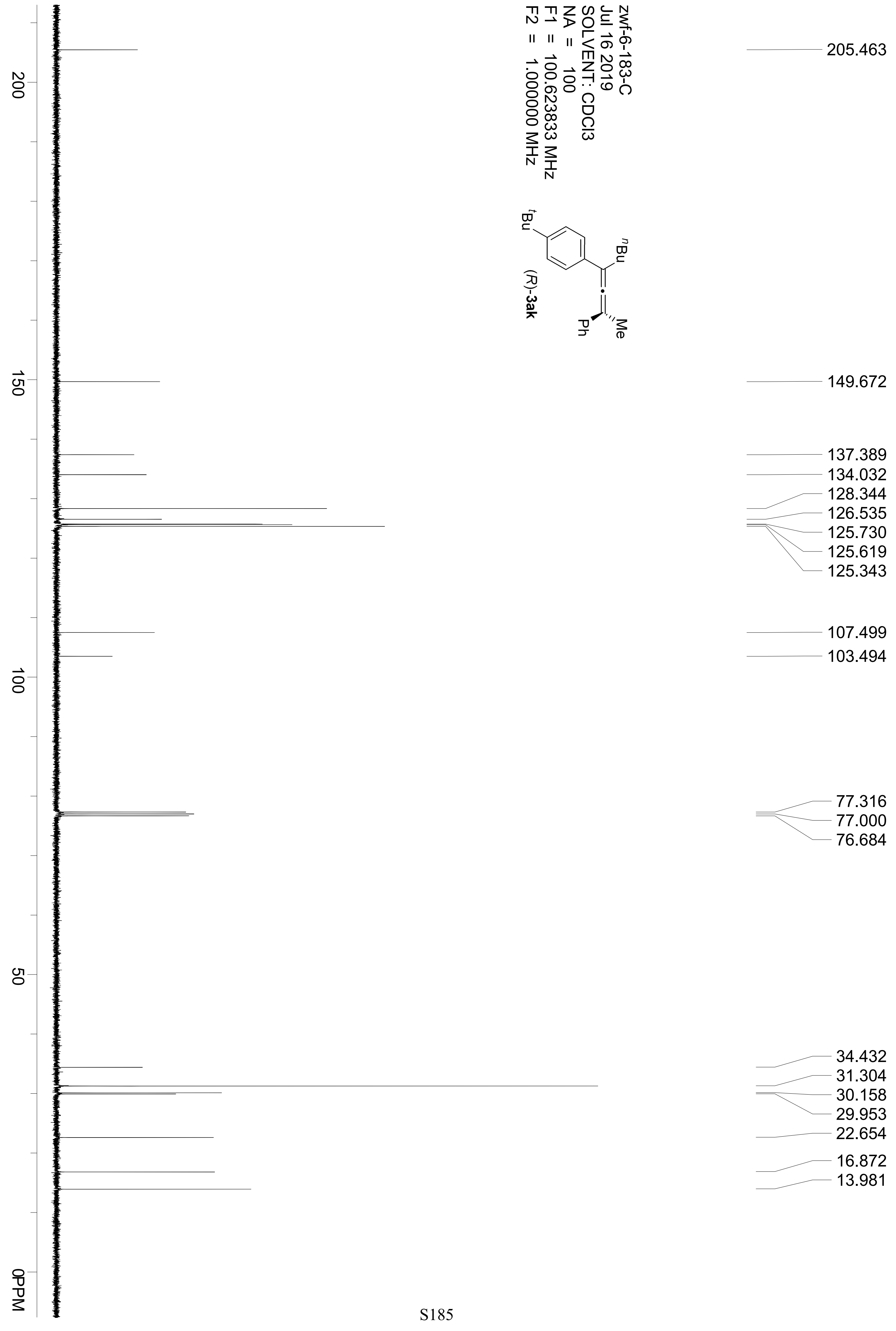

Supplementary Figure 113. ^{13}C NMR (100 MHz, $CDCl_3$) spectrum for (*R*)-**3ak**

Empower™ 3 SOFTWARE

Default Individual Report

SAMPLE INFORMATION

Sample Name:	zwf-7-037	Acquired By:	System
Sample Type:	Unknown	Sample Set Name:	
Vial:	1:E,1	Acq. Method Set:	upc_pda_2019m
Injection #:	1	Processing Method	TEST
Injection Volume:	2.00 ul	Channel Name:	PDA Ch1 254nm@4.8nm
Run Time:	35.0 Minutes	Proc. Chnl. Descr.:	PDA Ch1 254nm@4.8nm
Date Acquired:	9/11/2019 1:46:59 PM CST		
Date Processed:	9/12/2019 1:48:39 PM CST		

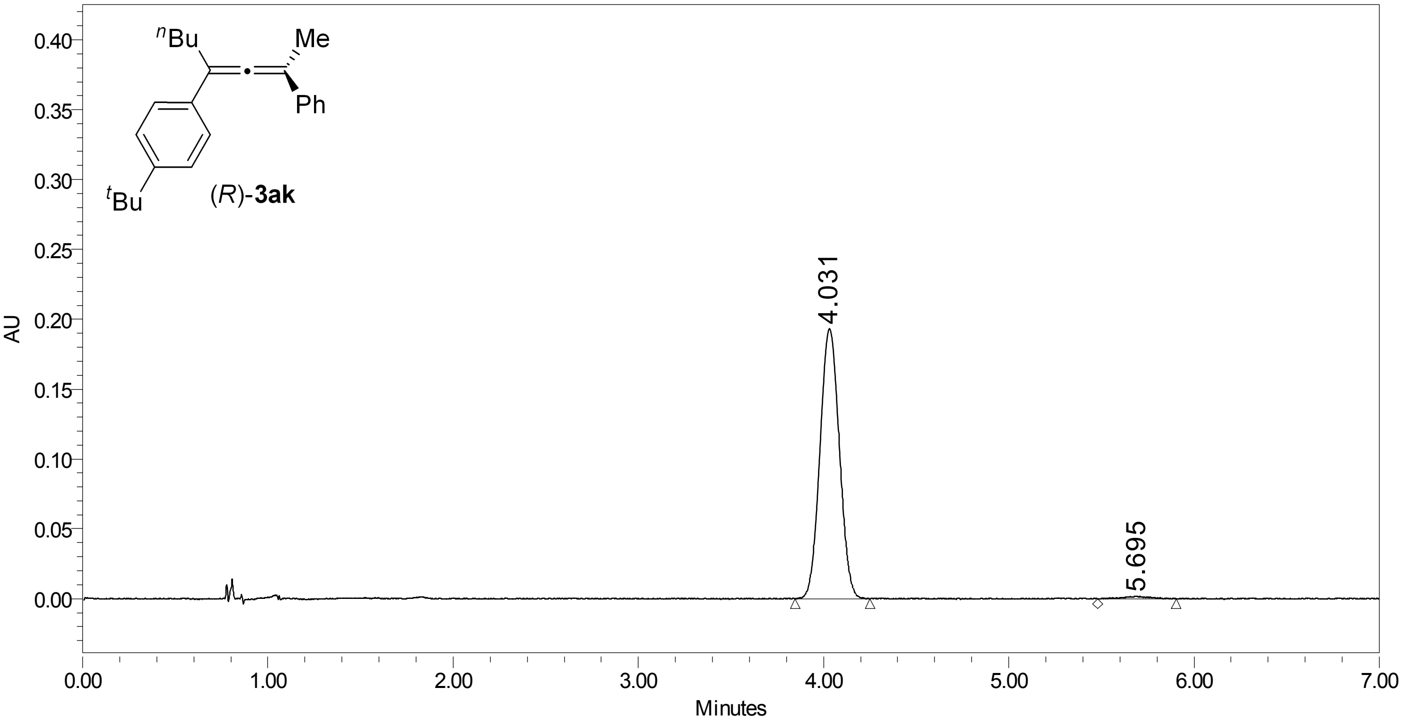

	RT	Peak Type	Height	Width (sec)	Area	% Area
1	4.031	Unknown	193418	24.400	1415920	98.39
2	5.695	Unknown	1983	25.450	23127	1.61

Reported by User: System
Report Method: Default Individual Report
Report Method ID 17266
Page: 1 of 1

Project Name: TEST
Date Printed:
9/12/2019
1:49:37 PM PRC

Supplementary Figure 114. SFC spectrum for (*R*)-**3ak**

Default Individual Report

SAMPLE INFORMATION

Sample Name:	zwf-6-174-rac	Acquired By:	System
Sample Type:	Unknown	Sample Set Name:	
Vial:	1:F,8	Acq. Method Set:	upc_pda_2019m
Injection #:	2	Processing Method	TEST
Injection Volume:	2.00 ul	Channel Name:	PDA Ch1 254nm@4.8nm
Run Time:	35.0 Minutes	Proc. Chnl. Descr.:	PDA Ch1 254nm@4.8nm
Date Acquired:	9/11/2019 1:31:16 PM CST		
Date Processed:	9/12/2019 1:48:14 PM CST		

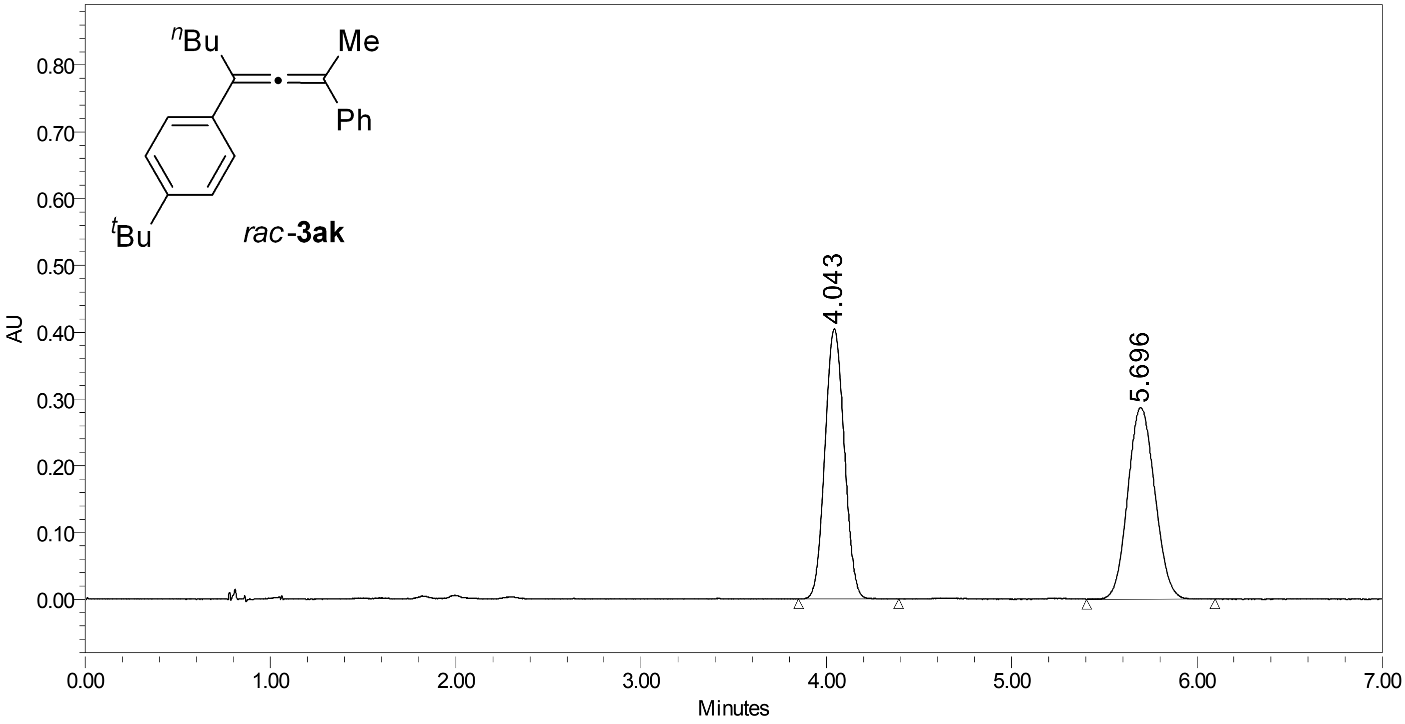

	RT	Peak Type	Height	Width (sec)	Area	% Area
1	4.043	Unknown	404684	32.400	2956585	50.44
2	5.696	Unknown	287013	41.600	2904480	49.56

Supplementary Figure 115. SFC spectrum for *rac*-**3ak**

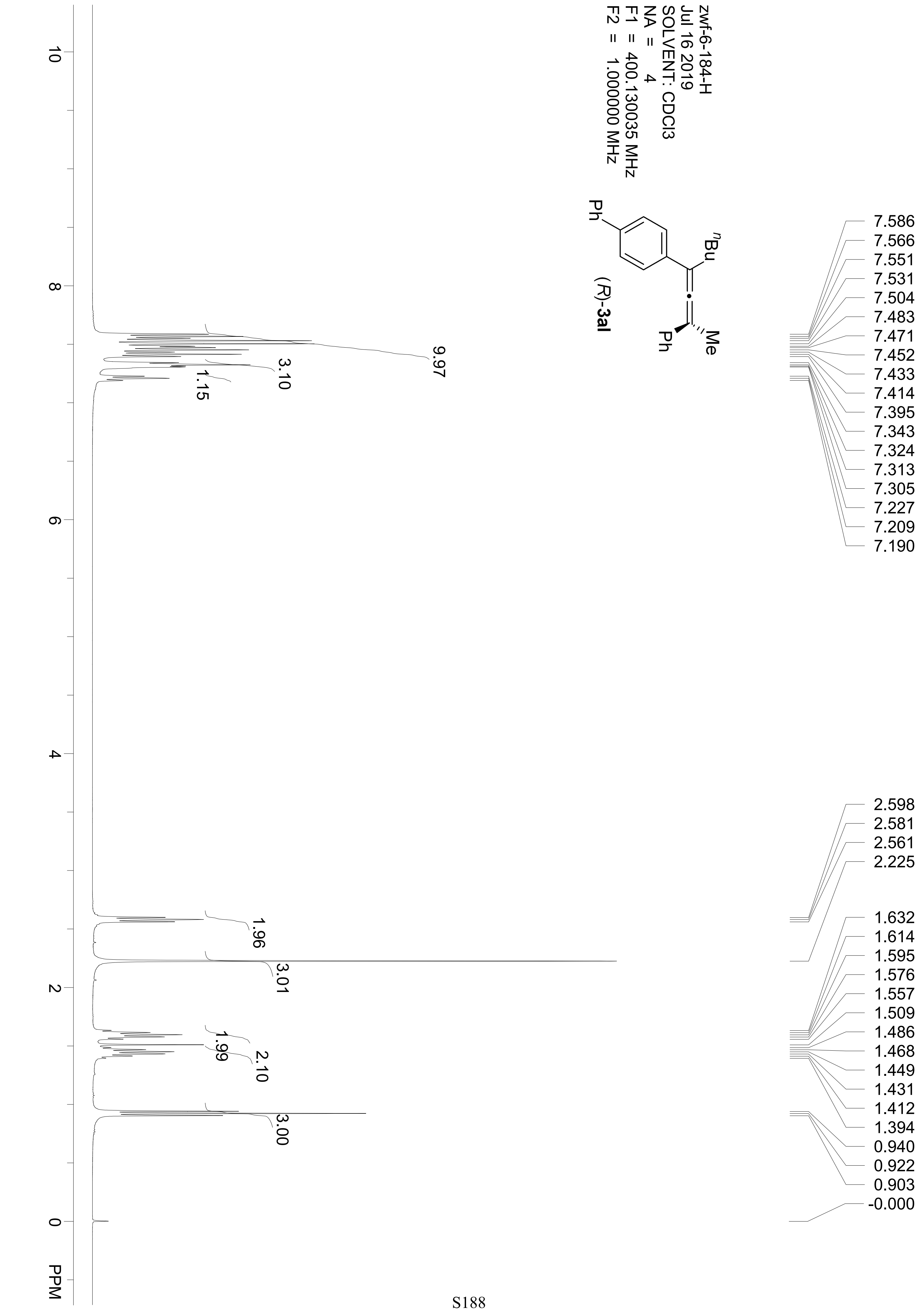

Supplementary Figure 116. ^{1}H NMR (400 MHz, $CDCl_3$) spectrum for (*R*)-**3al**

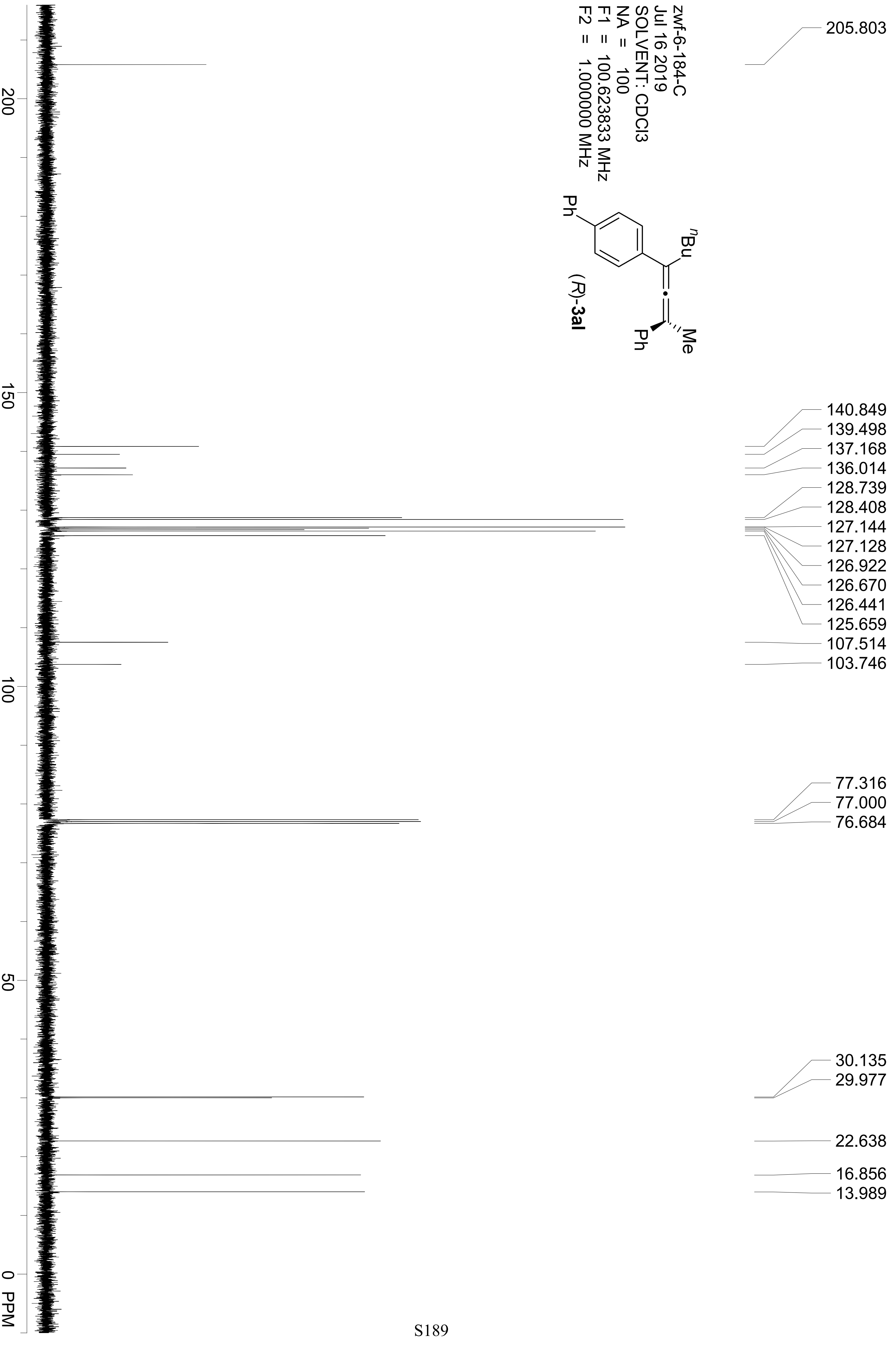

Supplementary Figure 117. ^{13}C NMR (100 MHz, $CDCl_3$) spectrum for (*R*)-**3al**

Area Percent Report

Agilent Technologies

sample zwf-7-033-OD-H-99.5-0.5-1.0-214

Data file: C:\Users\Public\Documents\ChemStation\1\Data\zwf-allenioc acid_LC 2019-09-07 14-54-21\025-P1-B3-zwf-7-033.D

Acquisition Data:

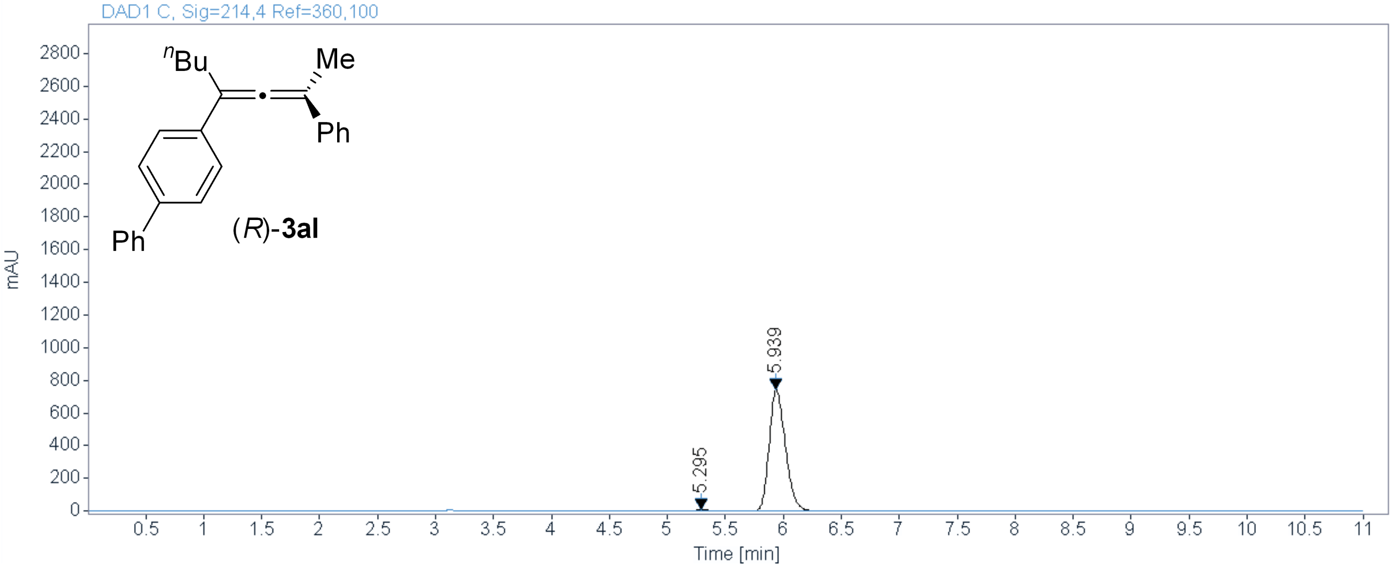

Signal: DAD1 C, Sig=214,4 Ref=360,100

RT [min]	Width [min]	Height	Area	Area%
5.295	0.1352	8.2855	74.7463	1.0139
5.939	0.1521	745.8649	7297.7358	98.9861
		Sum	7372.4821	100.0000

Supplementary Figure 118. HPLC spectrum for (*R*)-**3al**

Area Percent Report

Agilent Technologies

sample zwf-7-033-rac-OD-H-99.5-0.5-1.0-214

Data file: C:\Users\Public\Documents\ChemStation\1\Data\zwf-allenioc acid_LC 2019-09-07 14-54-21\026-P1-B4-zwf-7-033-rac.D

Acquisition Data:

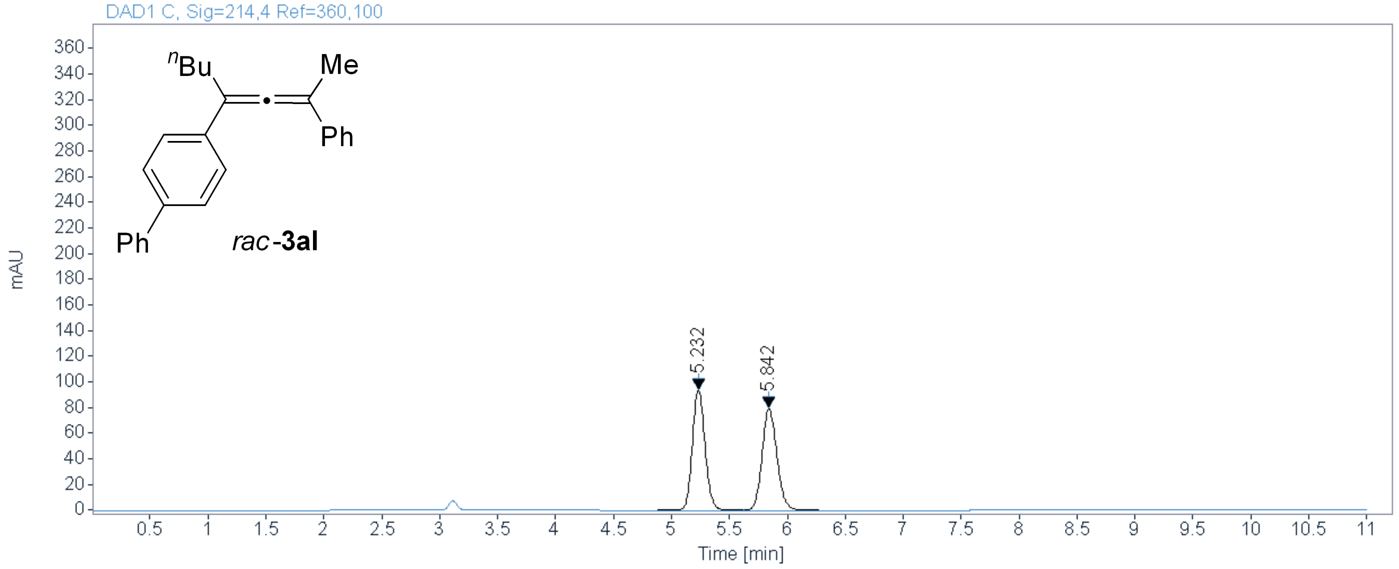

Signal: DAD1 C, Sig=214,4 Ref=360,100

RT [min]	Width [min]	Height	Area	Area%
5.232	0.1171	94.5666	722.1408	50.1139
5.842	0.1390	79.8692	718.8573	49.8861
		Sum	1440.9981	100.0000

Supplementary Figure 119. HPLC spectrum for *rac*-**3al**

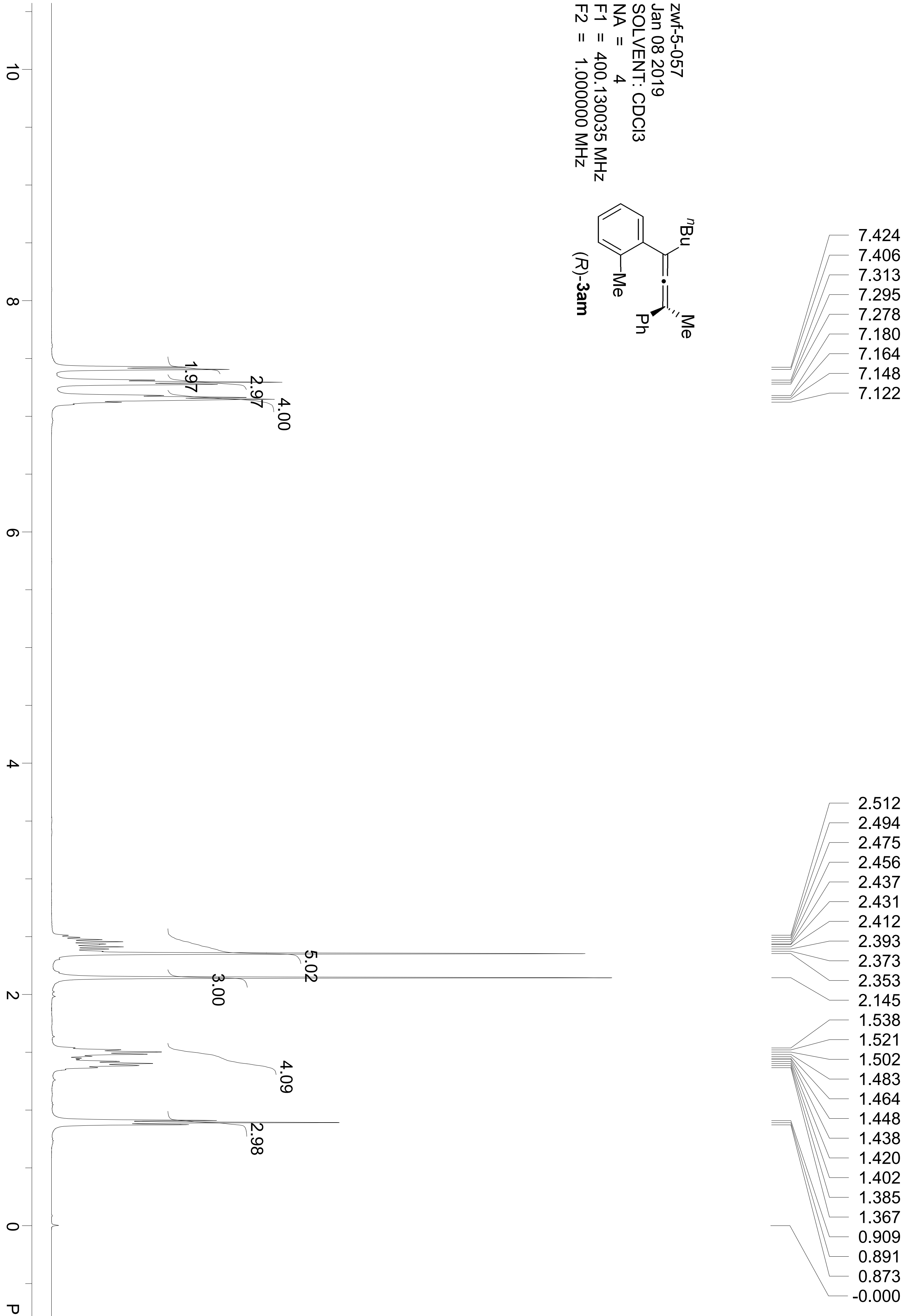

Supplementary Figure 120. ^{1}H NMR (400 MHz, $CDCl_3$) spectrum for (*R*)-**3am**

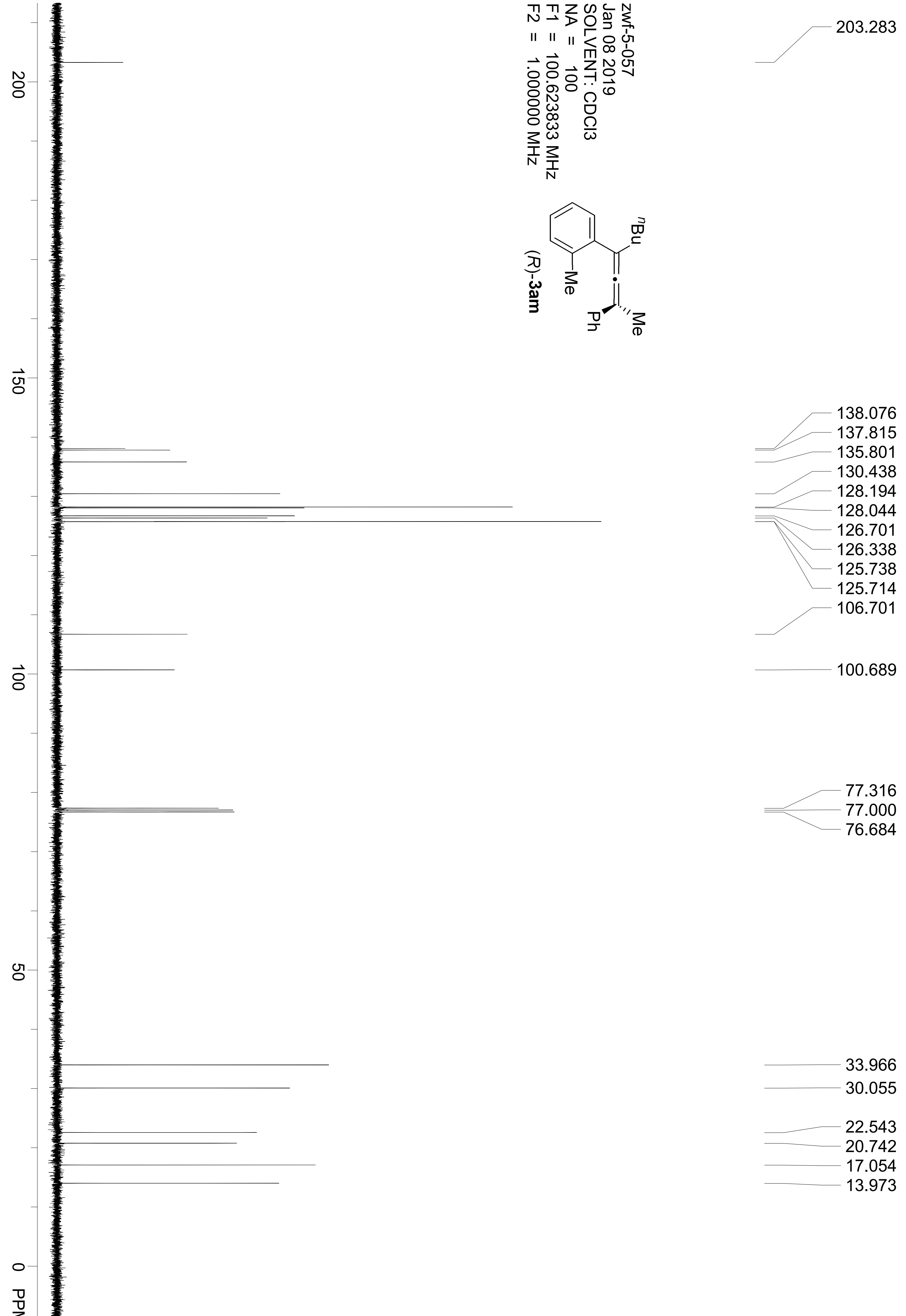

Supplementary Figure 121. ^{13}C NMR (100 MHz, $CDCl_3$) spectrum for (*R*)-**3am**

Default Individual Report

SAMPLE INFORMATION

Sample Name:	zwf-7-035	Acquired By:	System
Sample Type:	Unknown	Sample Set Name:	
Vial:	1:E,3	Acq. Method Set:	upc_pda_2019m
Injection #:	1	Processing Method	TEST
Injection Volume:	2.00 ul	Channel Name:	230.0nm
Run Time:	35.0 Minutes	Proc. Chnl. Descr.:	PDA Spectrum PDA 230.0 nm
Date Acquired:	9/11/2019 3:15:49 PM CST		
Date Processed:	9/12/2019 1:28:30 PM CST		

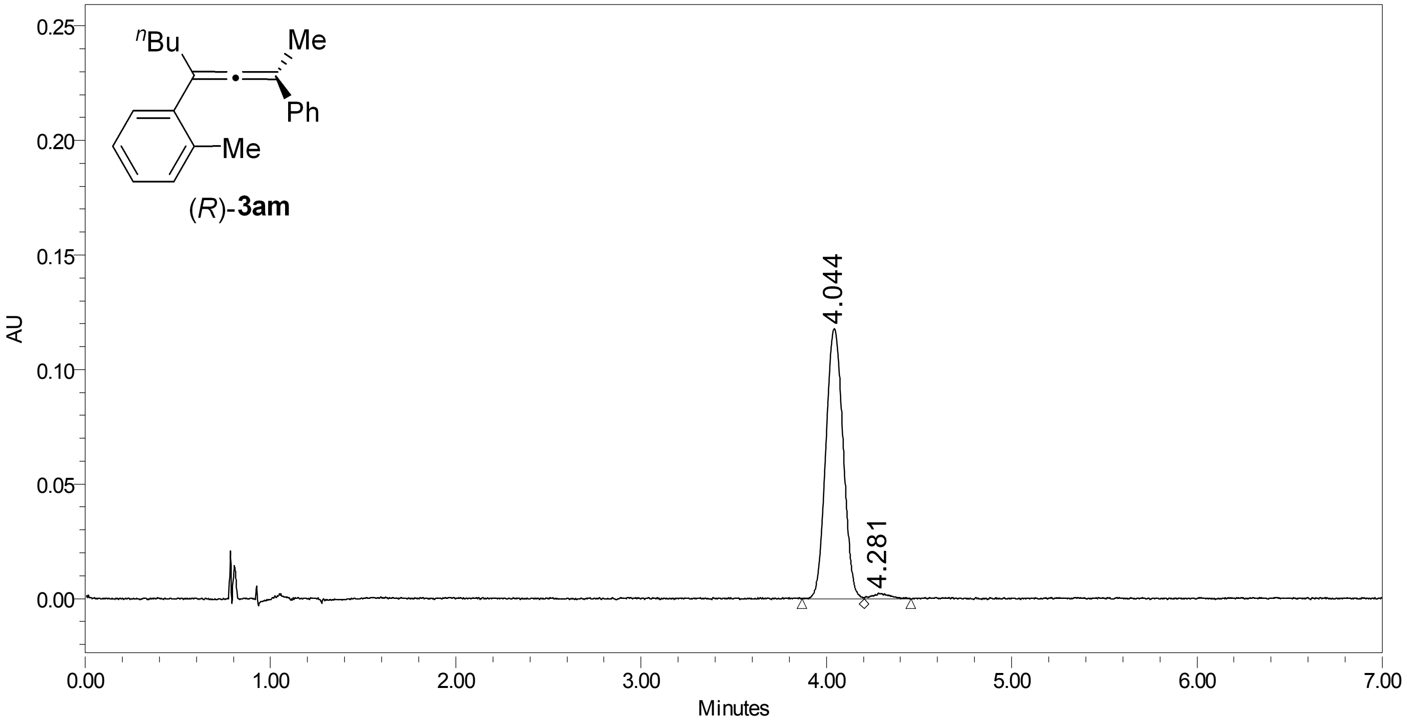

	RT	Peak Type	Height	Width (sec)	Area	% Area
1	4.044	Unknown	118051	20.202	772763	97.81
2	4.281	Unknown	2508	15.103	17334	2.19

Reported by User: System
Report Method: Default Individual Report
Report Method ID 17266
Page: 1 of 1

Project Name: TEST
Date Printed:
9/12/2019
1:43:38 PM PRC

Supplementary Figure 122. SFC spectrum for (*R*)-**3am**

Default Individual Report

SAMPLE INFORMATION

Sample Name:	zwf-5-056-rac	Acquired By:	System
Sample Type:	Unknown	Sample Set Name:	
Vial:	1:E,2	Acq. Method Set:	upc_pda_2019m
Injection #:	1	Processing Method	TEST
Injection Volume:	2.00 ul	Channel Name:	230.0nm
Run Time:	35.0 Minutes	Proc. Chnl. Descr.:	PDA Spectrum PDA 230.0 nm
Date Acquired:	9/11/2019 2:54:07 PM CST		
Date Processed:	9/12/2019 1:27:46 PM CST		

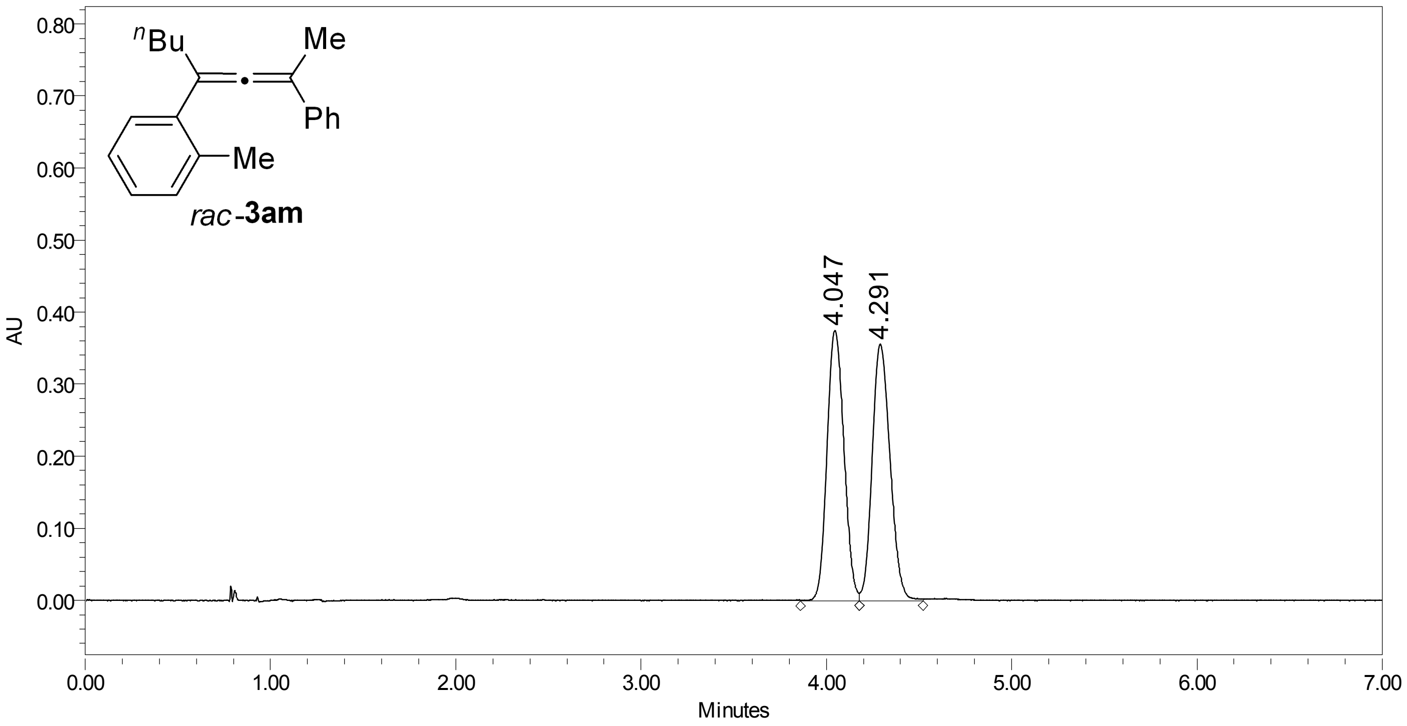

	RT	Peak Type	Height	Width (sec)	Area	% Area
1	4.047	Unknown	375579	19.052	2402145	49.77
2	4.291	Unknown	356304	20.604	2423985	50.23

Supplementary Figure 123. SFC spectrum for *rac*-**3am**

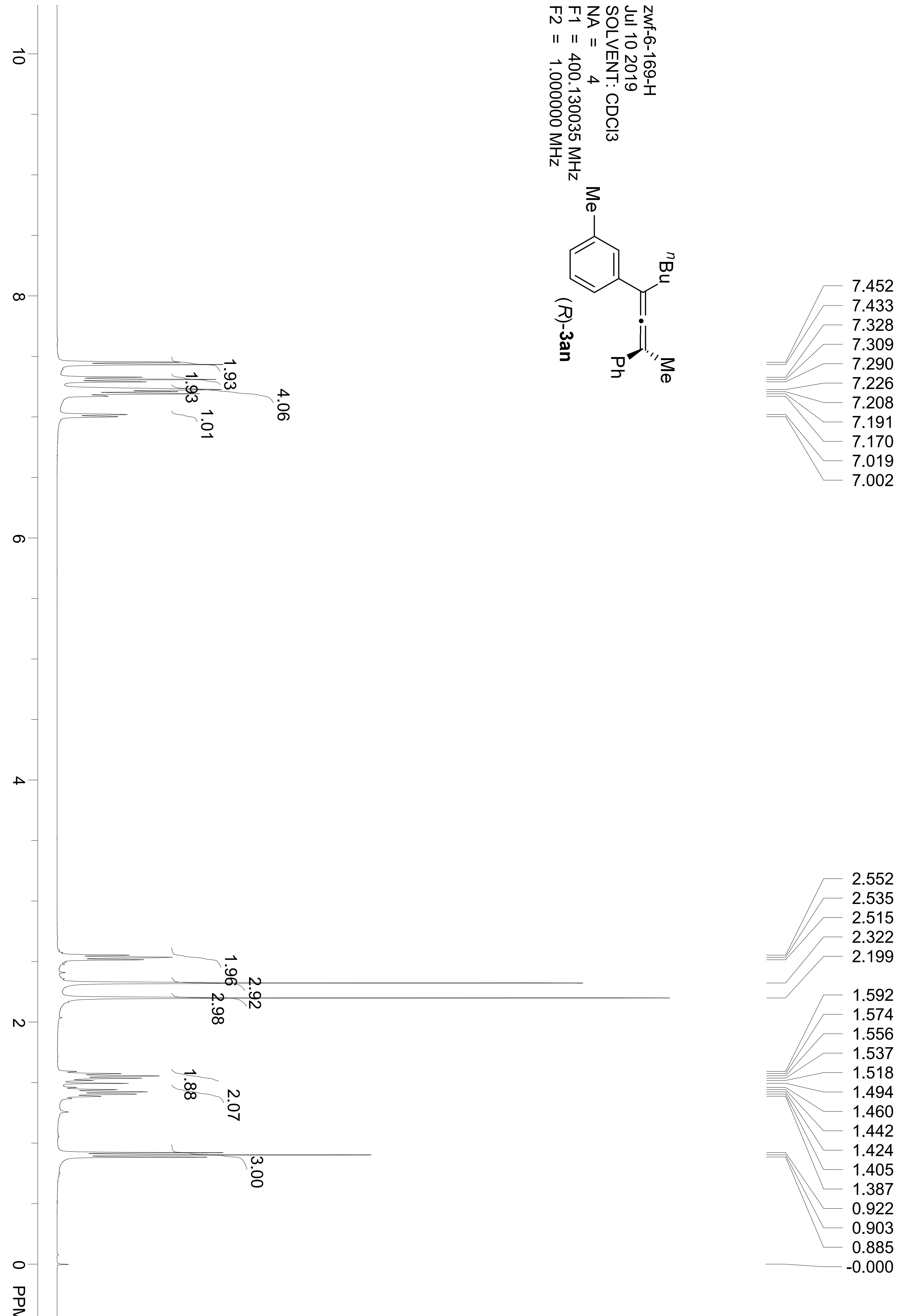

Supplementary Figure 124. ^{1}H NMR (400 MHz, $CDCl_3$) spectrum for (*R*)-**3an**

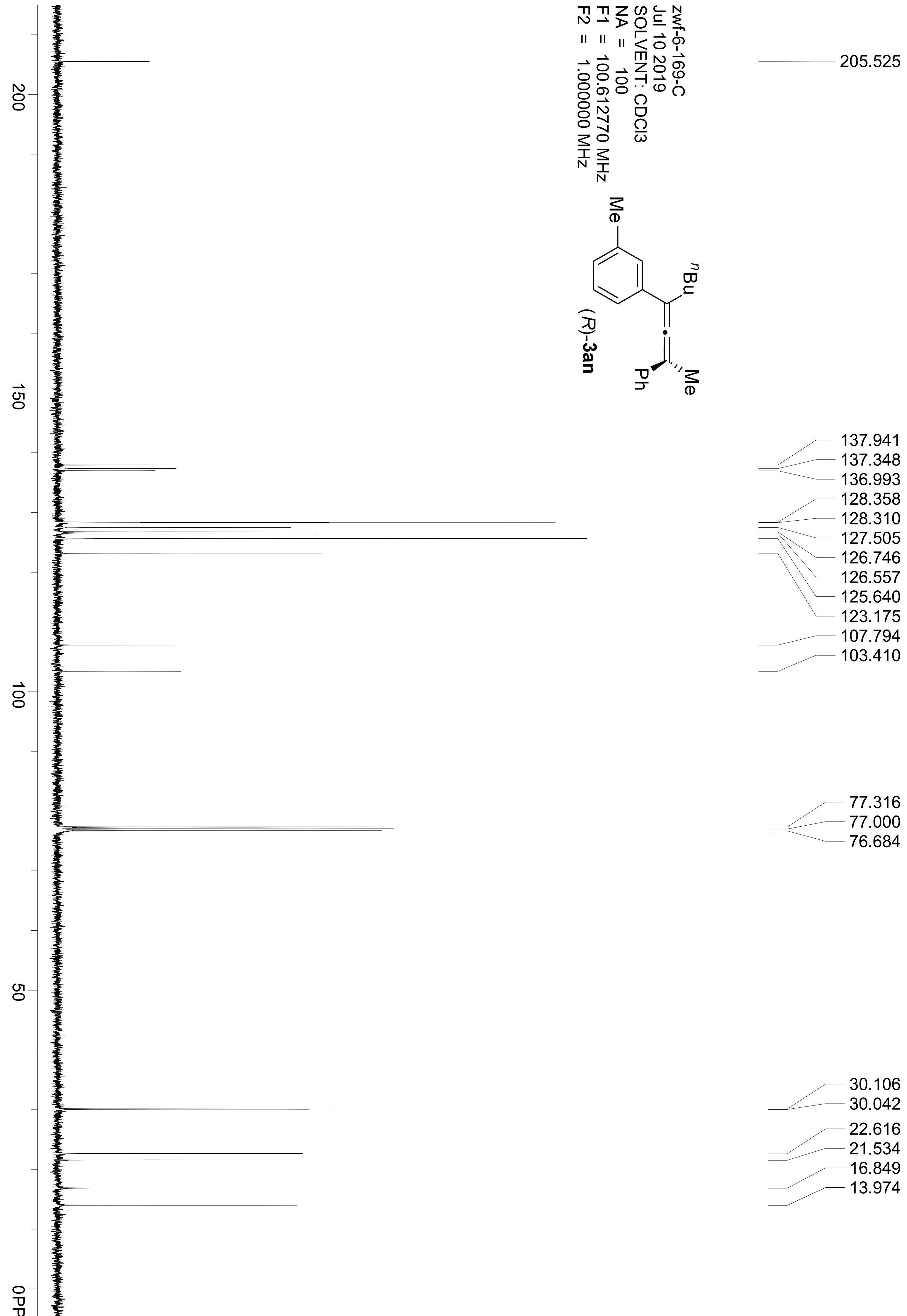

Supplementary Figure 125. ^{13}C NMR (100 MHz, $CDCl_3$) spectrum for (*R*)-**3an**

Area Percent Report

Agilent Technologies

sample zwf-7-027-OJ-H-99.5-0.5-0.7-214

Data file: C:\Users\Public\Documents\ChemStation\1\Data\zwf-allenioc acid_LC 2019-09-07 14-54-21\002-P1-C1-zwf-7-027.D

Acquisition Data:

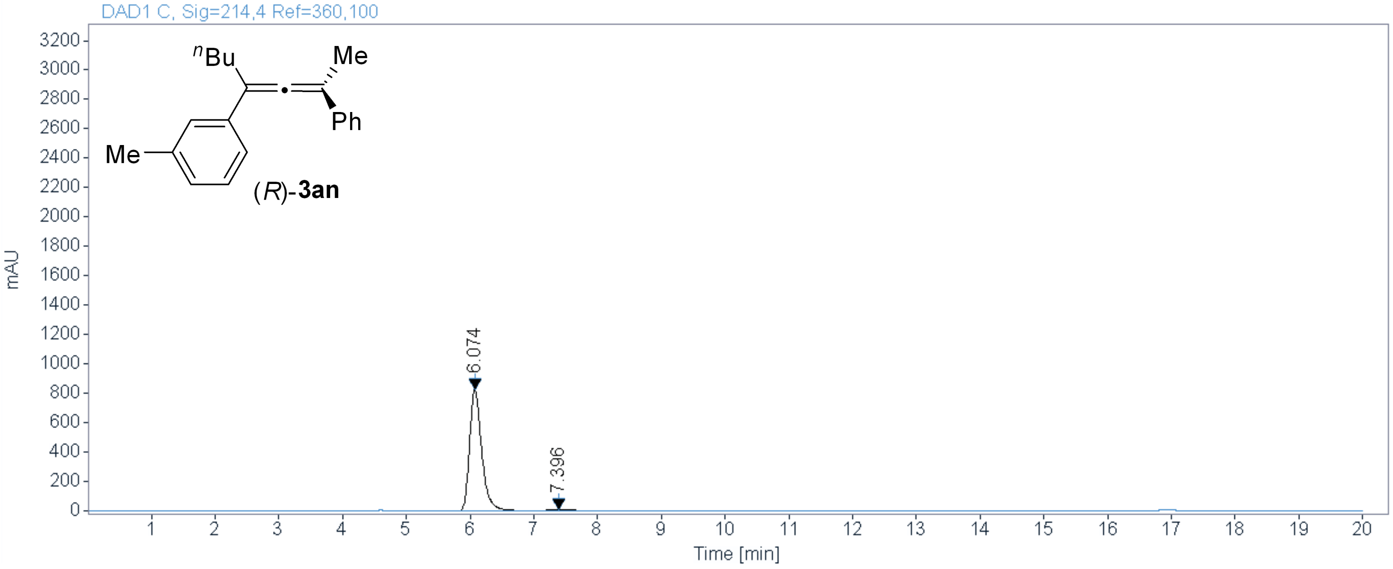

Signal: DAD1 C, Sig=214,4 Ref=360,100

RT [min]	Width [min]	Height	Area	Area%
6.074	0.2026	827.8472	10969.5996	97.4851
7.396	0.3971	11.1139	282.9916	2.5149
		Sum	11252.5912	100.0000

Supplementary Figure 126. HPLC spectrum for (*R*)-**3an**

Area Percent Report

Agilent Technologies

sample zwf-7-027-rac-OJ-H-99.5-0.5-0.7-214

Data file: C:\Users\Public\Documents\ChemStation\1\Data\zwf-allenioc acid_LC 2019-09-07 14-54-21\004-P1-C2-zwf-7-027-rac.D

Acquisition Data:

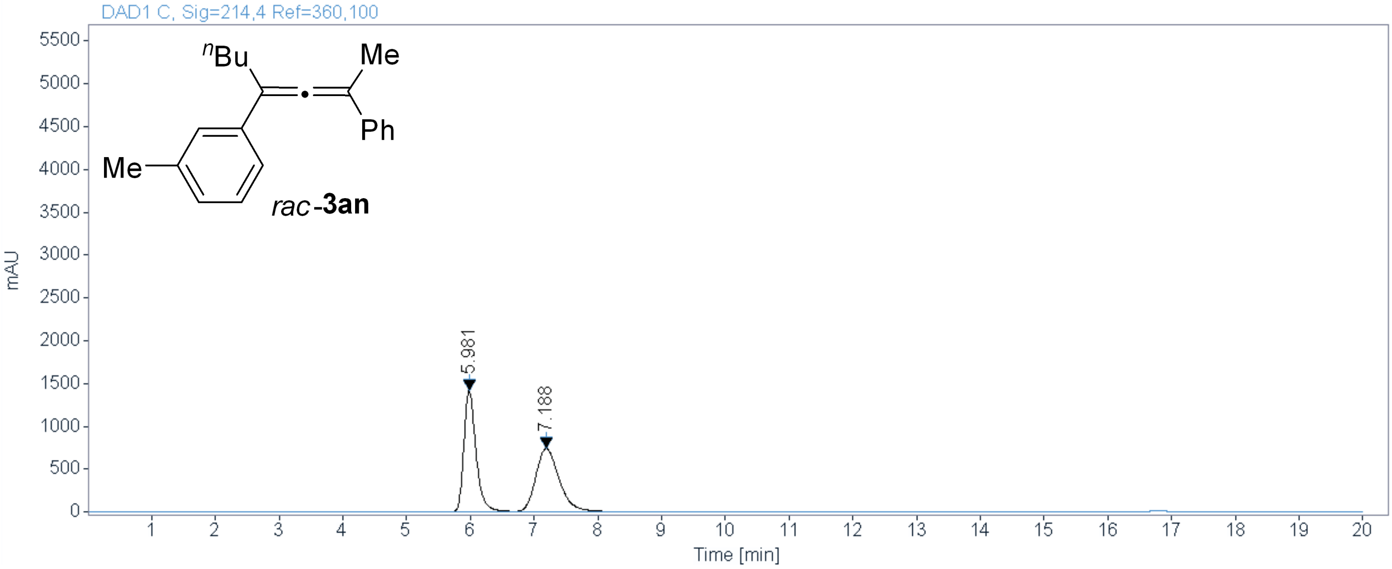

Signal: DAD1 C, Sig=214,4 Ref=360,100

RT [min]	Width [min]	Height	Area	Area%
5.981	0.2033	1421.1796	18923.6055	49.6639
7.188	0.4315	740.8430	19179.7363	50.3361
		Sum	38103.3418	100.0000

Supplementary Figure 127. HPLC spectrum for *rac*-**3an**

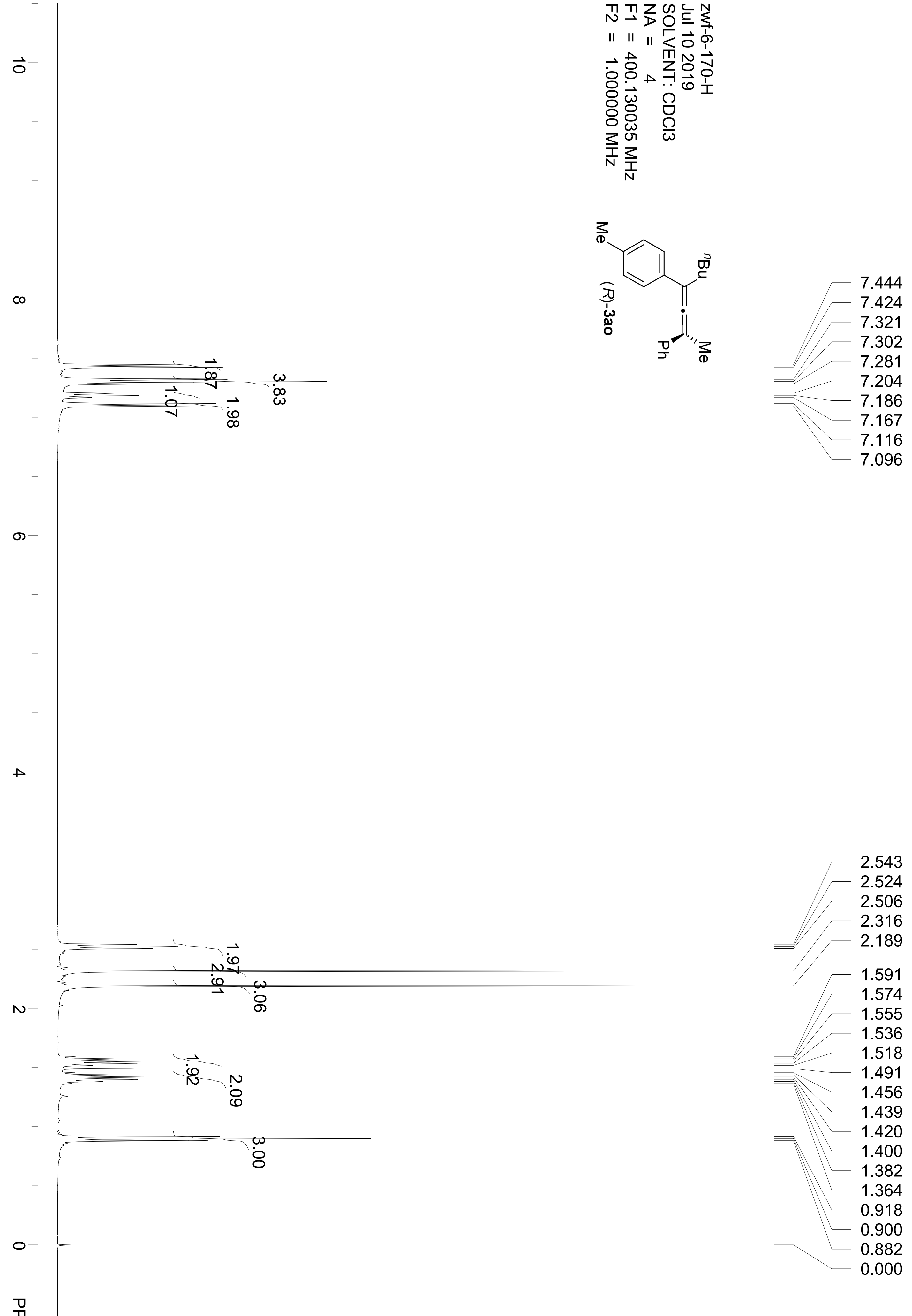

Supplementary Figure 128. ^{1}H NMR (400 MHz, $CDCl_3$) spectrum for (*R*)-**3ao**

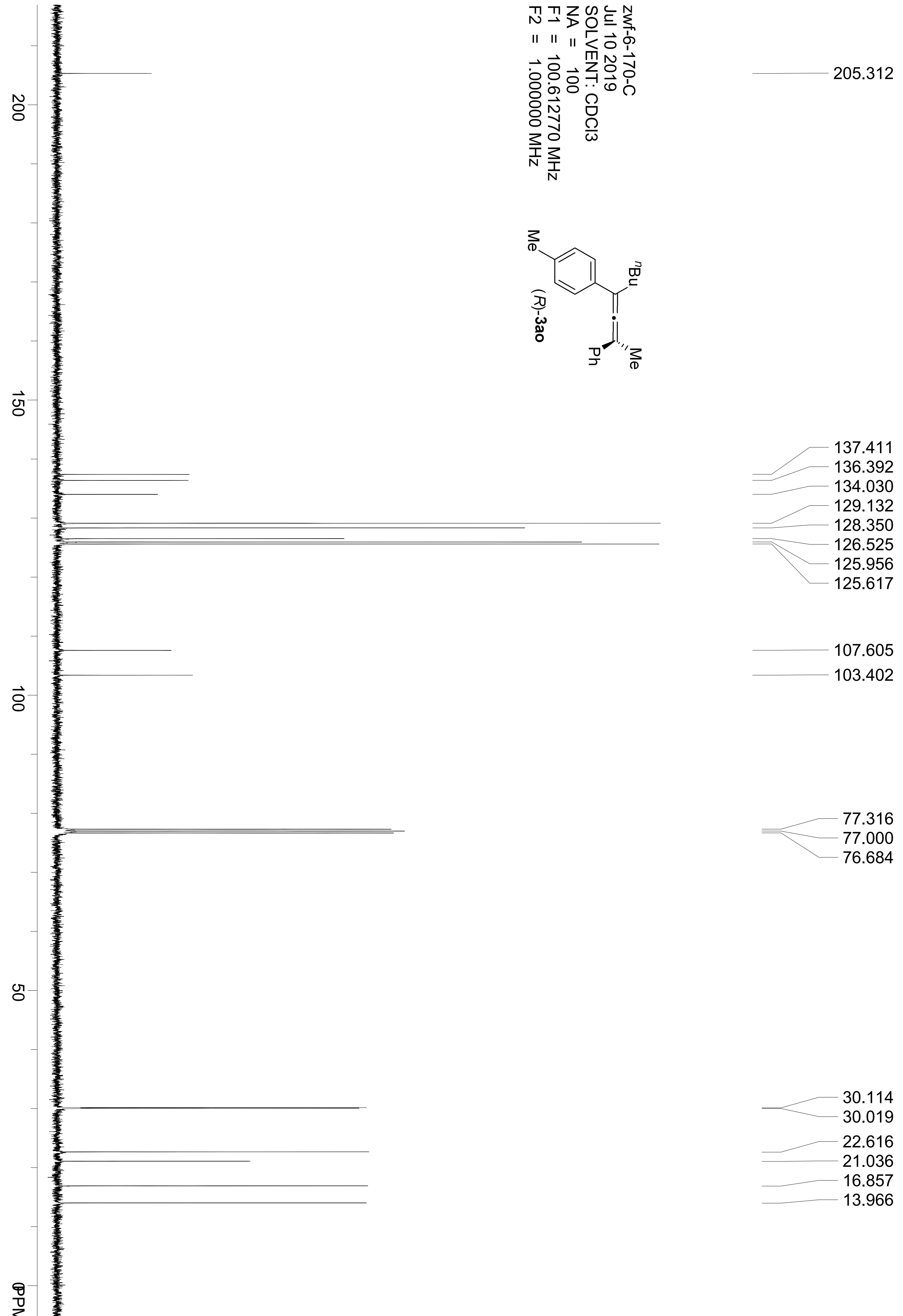

Supplementary Figure 129. ^{13}C NMR (100 MHz, $CDCl_3$) spectrum for (*R*)-**3ao**

Default Individual Report

SAMPLE INFORMATION

Sample Name:	zwf-7-028	Acquired By:	System
Sample Type:	Unknown	Sample Set Name:	
Vial:	1:F,3	Acq. Method Set:	upc_pda_2019m
Injection #:	1	Processing Method	TEST
Injection Volume:	2.00 ul	Channel Name:	PDA Ch2 214nm@4.8nm
Run Time:	35.0 Minutes	Proc. Chnl. Descr.:	PDA Ch2 214nm@4.8nm
Date Acquired:	9/11/2019 9:43:22 AM CST		
Date Processed:	9/12/2019 1:20:12 PM CST		

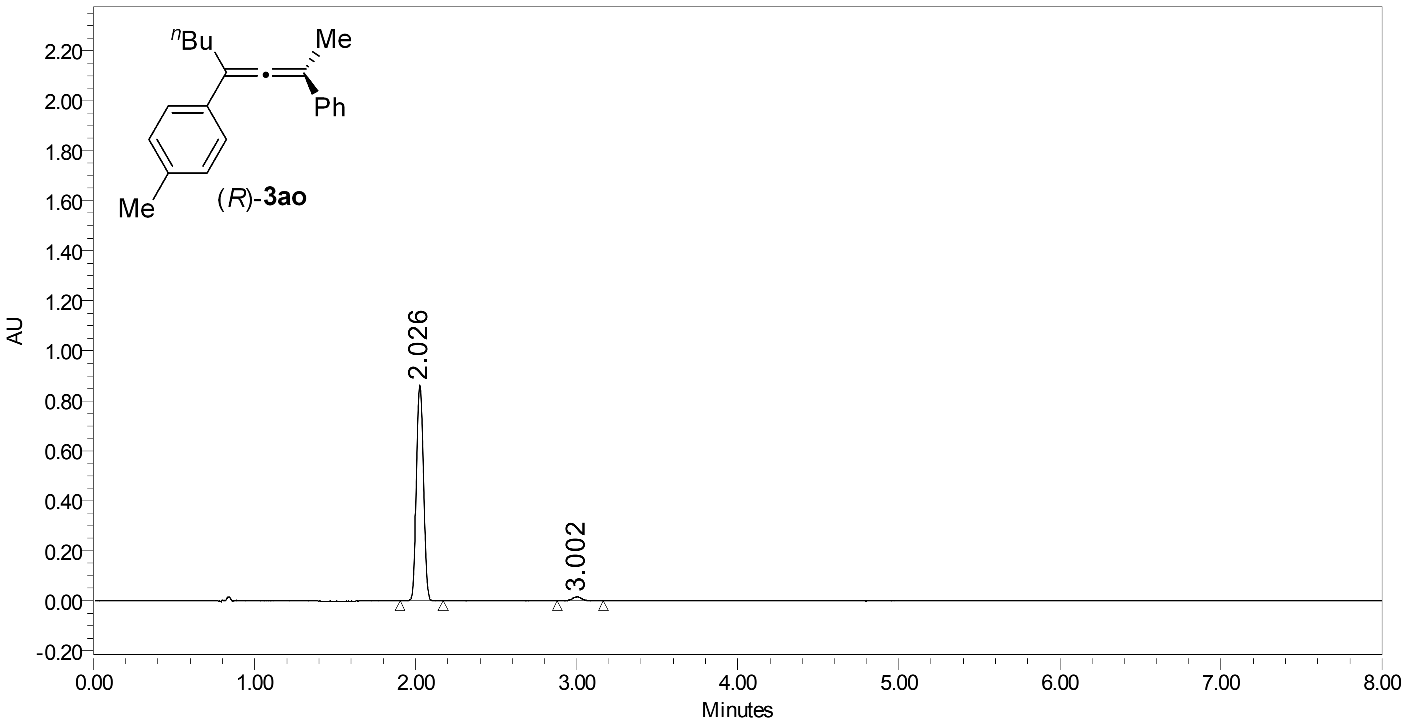

	RT	Peak Type	Height	Width (sec)	Area	% Area
1	2.026	Unknown	864050	16.050	2663627	97.56
2	3.002	Unknown	15794	17.150	66713	2.44

Reported by User: System
Report Method: Default Individual Report
Report Method IΣ 17262
Page: 1 of 1

Project Name: TEST
Date Printed:
9/12/2019
1:39:50 PM PRC

Supplementary Figure 130. SFC spectrum for (*R*)-**3ao**

Empower™ 3 SOFTWARE

Default Individual Report

SAMPLE INFORMATION

Sample Name:	zwf-6-164-rac	Acquired By:	System
Sample Type:	Unknown	Sample Set Name:	
Vial:	1:F,2	Acq. Method Set:	upc_pda_2019m
Injection #:	1	Processing Method	TEST
Injection Volume:	2.00 ul	Channel Name:	PDA Ch2 214nm@4.8nm
Run Time:	35.0 Minutes	Proc. Chnl. Descr.:	PDA Ch2 214nm@4.8nm
Date Acquired:	9/11/2019 9:33:34 AM CST		
Date Processed:	9/12/2019 1:19:31 PM CST		

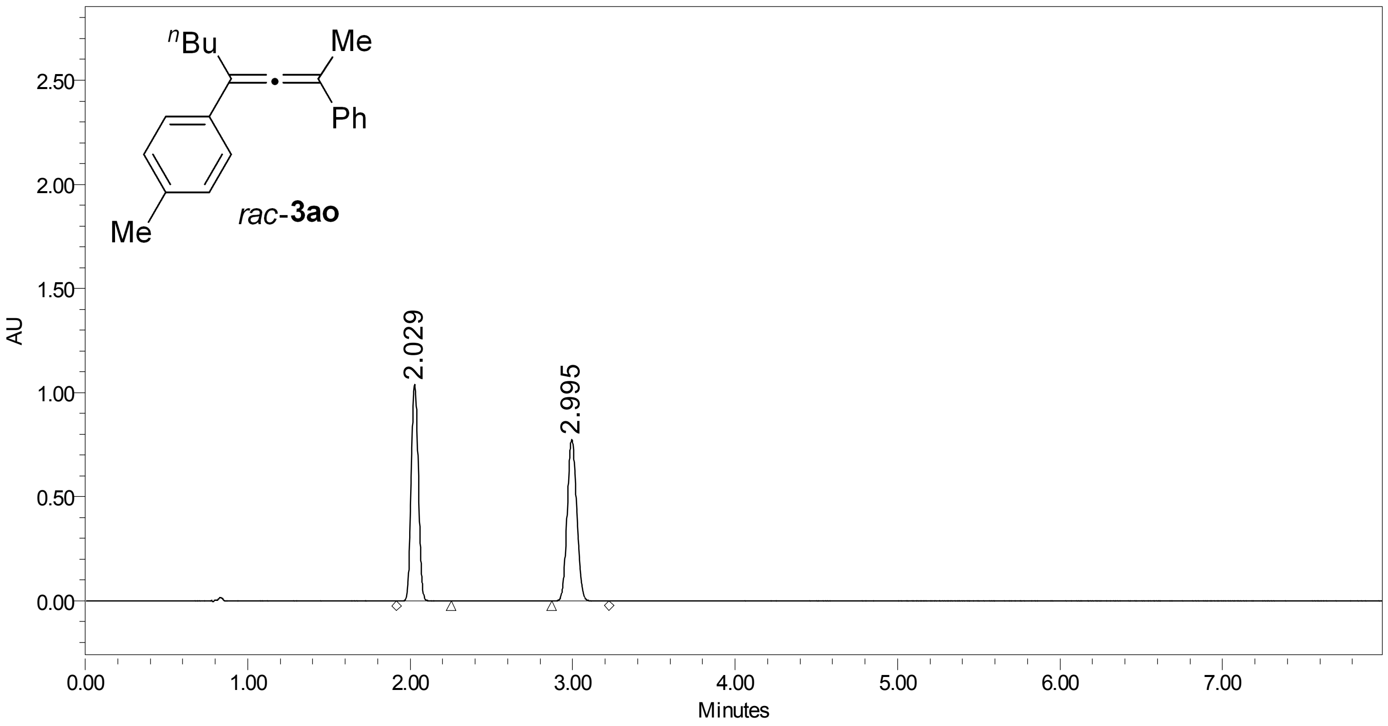

	RT	Peak Type	Height	Width (sec)	Area	% Area
1	2.029	Unknown	1038752	20.250	3156219	50.05
2	2.995	Unknown	776208	21.200	3150043	49.95

Reported by User: System
Report Method: Default Individual Report
Report Method ID 16683
Page: 1 of 1

Project Name: TEST
Date Printed:
9/12/2019
1:39:04 PM PRC

Supplementary Figure 131. SFC spectrum for *rac*-**3ao**

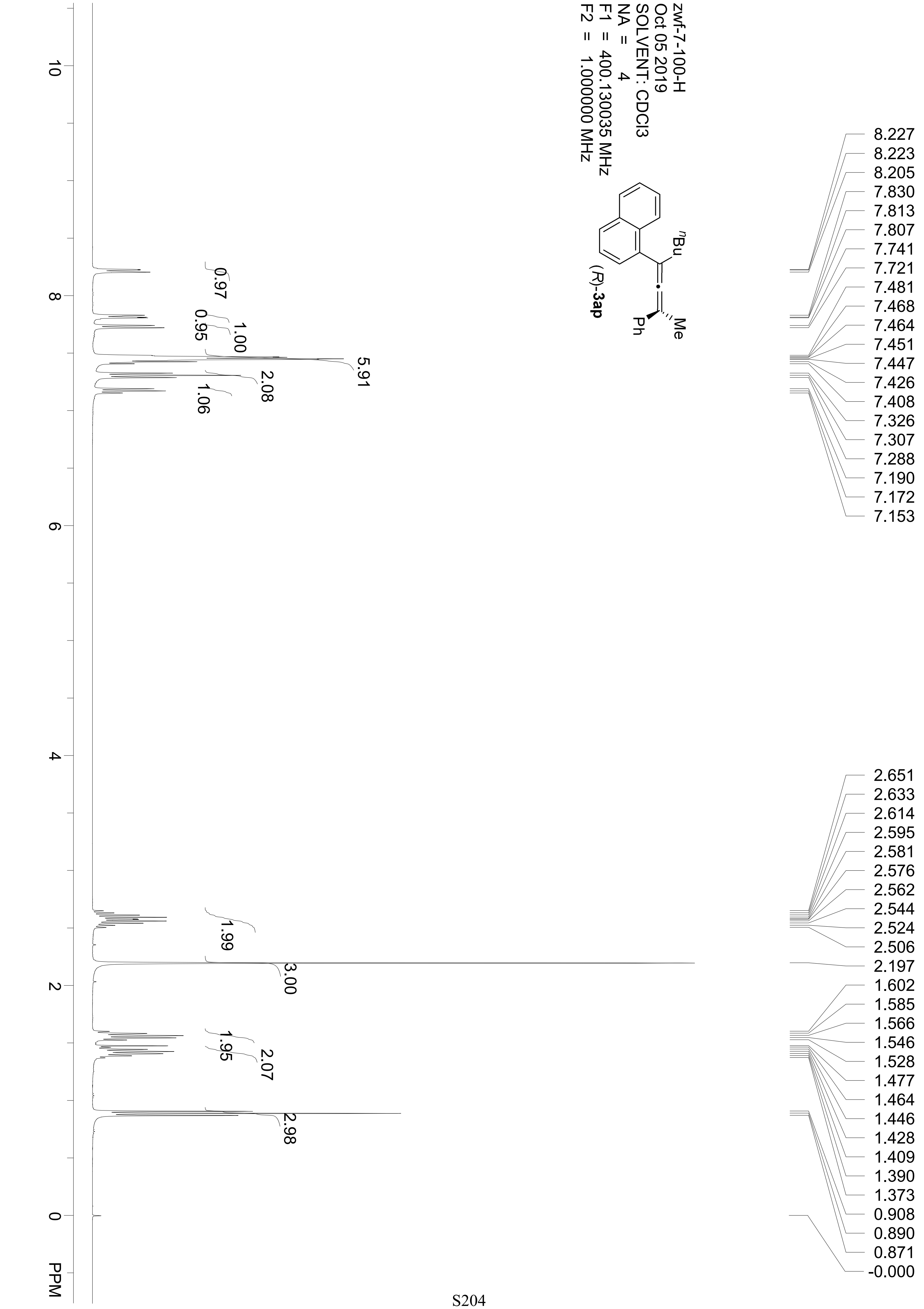

Supplementary Figure 132. ^{1}H NMR (400 MHz, $CDCl_3$) spectrum for (*R*)-**3ap**

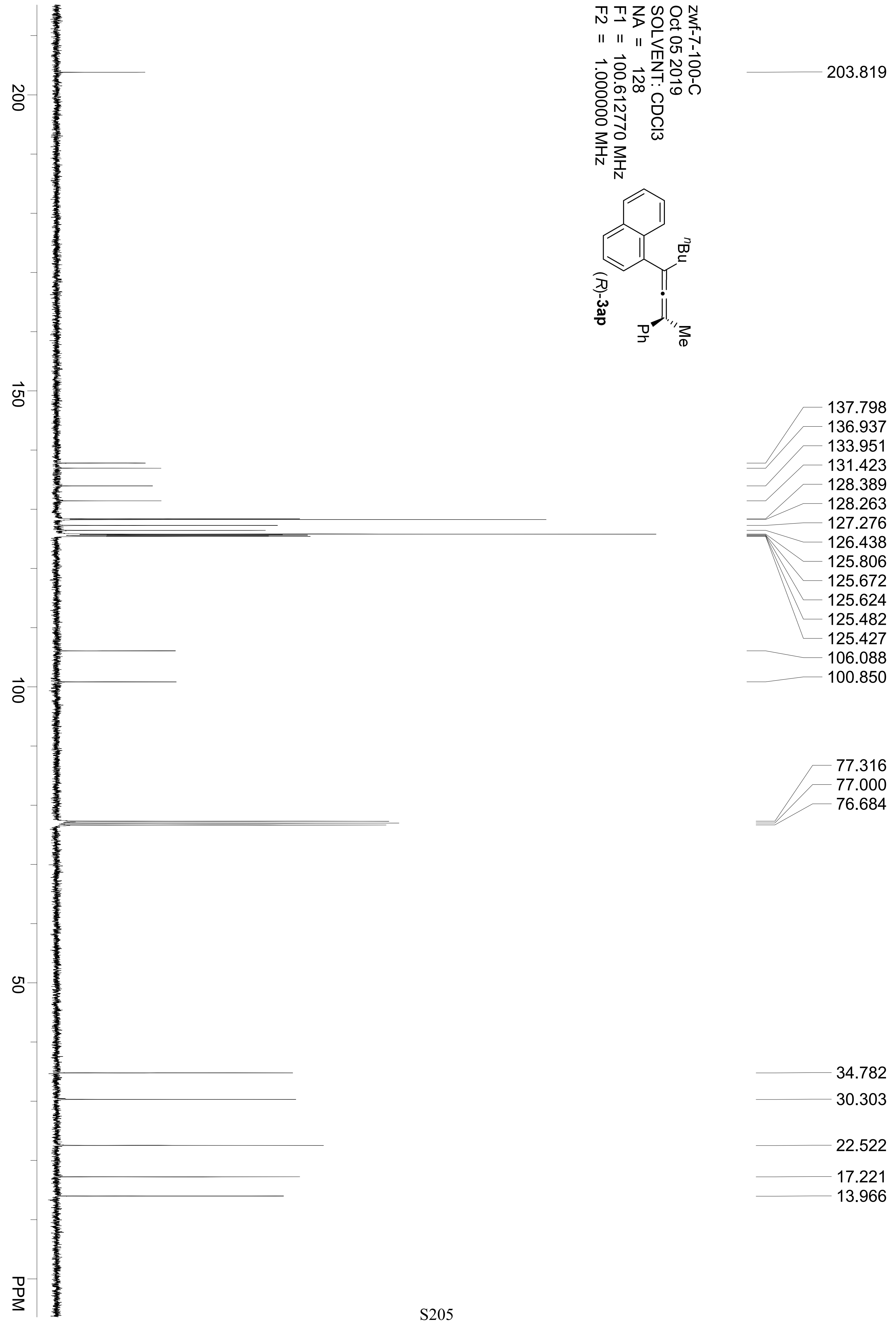

Supplementary Figure 133. ^{13}C NMR (100 MHz, $CDCl_3$) spectrum for (*R*)-**3ap**

Default Individual Report

SAMPLE INFORMATION

Sample Name:	zwf-7-100-chiral	Acquired By:	System
Sample Type:	Unknown	Sample Set Name:	
Vial:	1:B,1	Acq. Method Set:	upc_pda_2019m
Injection #:	2	Processing Method	TEST
Injection Volume:	2.00 ul	Channel Name:	PDA Ch3 210nm@4.8nm
Run Time:	40.0 Minutes	Proc. Chnl. Descr.:	PDA Ch3 210nm@4.8nm
Date Acquired:	10/10/2019 1:02:21 PM CST		
Date Processed:	10/10/2019 3:33:19 PM CST		

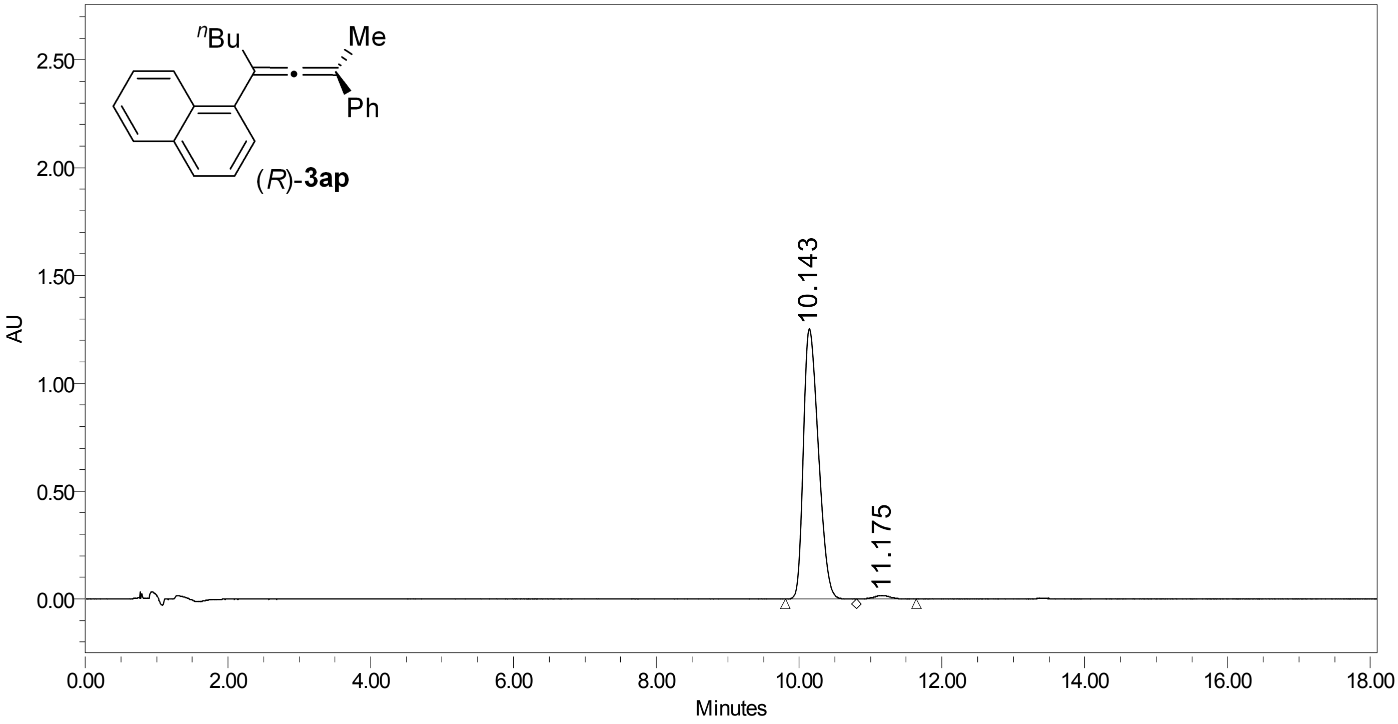

	RT	Peak Type	Height	Width (sec)	Area	% Area
1	10.143	Unknown	1253197	59.800	18530800	98.54
2	11.175	Unknown	16611	50.250	275185	1.46

Supplementary Figure 134. SFC spectrum for (*R*)-**3ap**

Empower™ 3 SOFTWARE

Default Individual Report

SAMPLE INFORMATION

Sample Name:	zwf-7-98-rac	Acquired By:	System
Sample Type:	Unknown	Sample Set Name:	
Vial:	1:a,1	Acq. Method Set:	upc_pda_2019m
Injection #:	1	Processing Method	TEST
Injection Volume:	2.00 ul	Channel Name:	PDA Ch3 210nm@4.8nm
Run Time:	40.0 Minutes	Proc. Chnl. Descr.:	PDA Ch3 210nm@4.8nm
Date Acquired:	10/10/2019 1:21:49 PM CST		
Date Processed:	10/10/2019 3:33:00 PM CST		

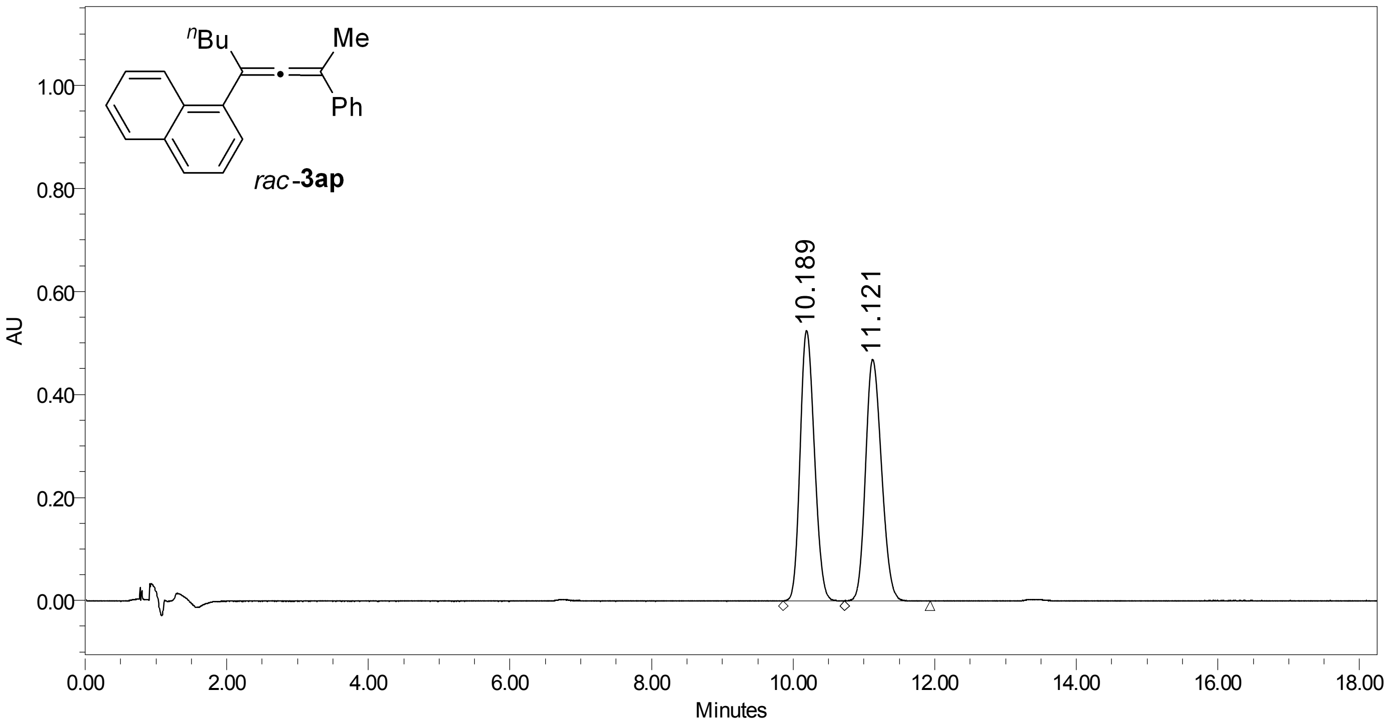

	RT	Peak Type	Height	Width (sec)	Area	% Area
1	10.189	Unknown	525264	52.200	7438106	50.00
2	11.121	Unknown	469381	72.400	7437056	50.00

Reported by User: System
Report Method: Default Individual Report
Report Method II 17925
Page: 1 of 1

Project Name: TEST
Date Printed:
10/10/2019
3:33:53 PM PRC

Supplementary Figure 135. SFC spectrum for *rac*-**3ap**

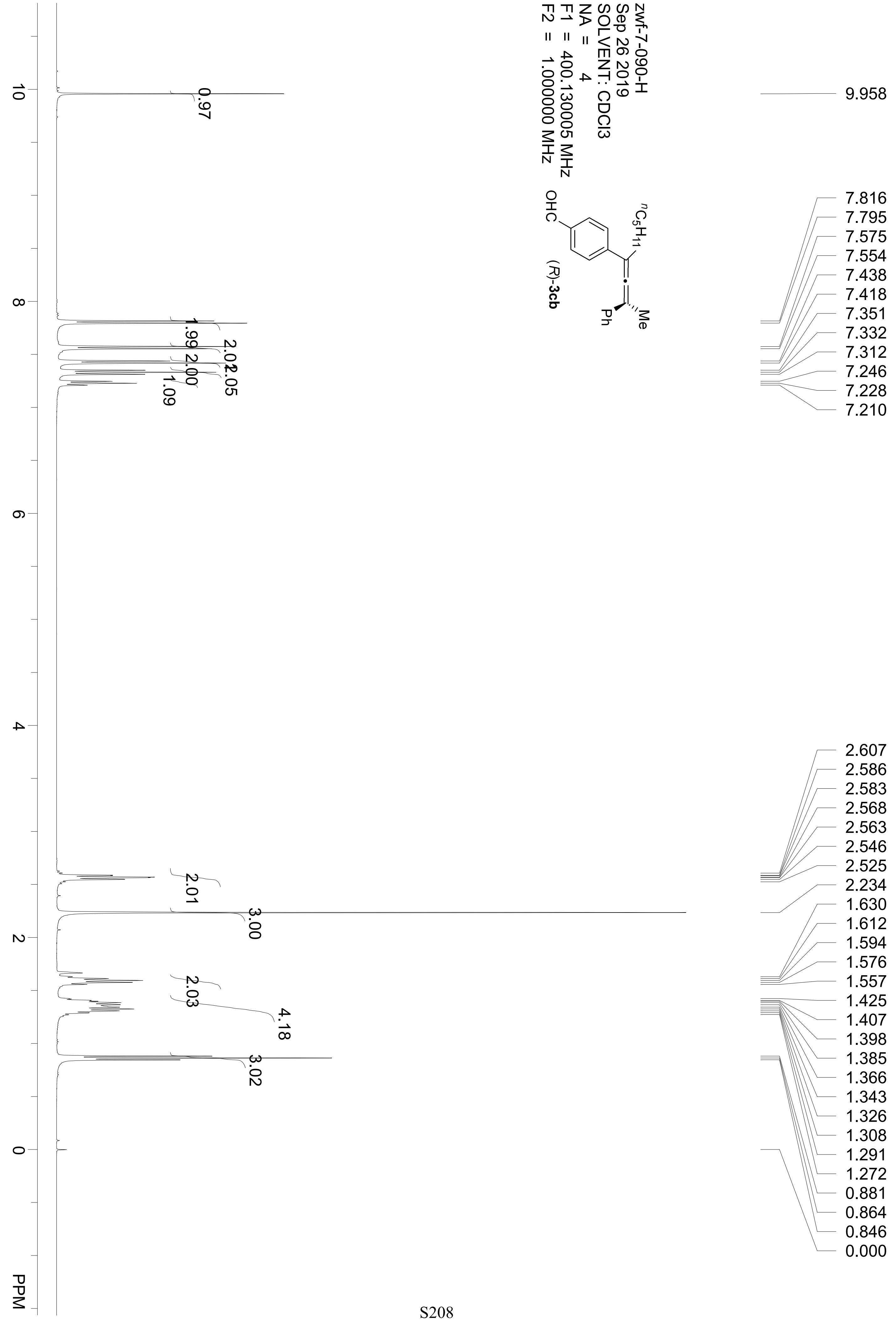

Supplementary Figure 136. ^{1}H NMR (400 MHz, $CDCl_3$) spectrum for (*R*)-**3cb**

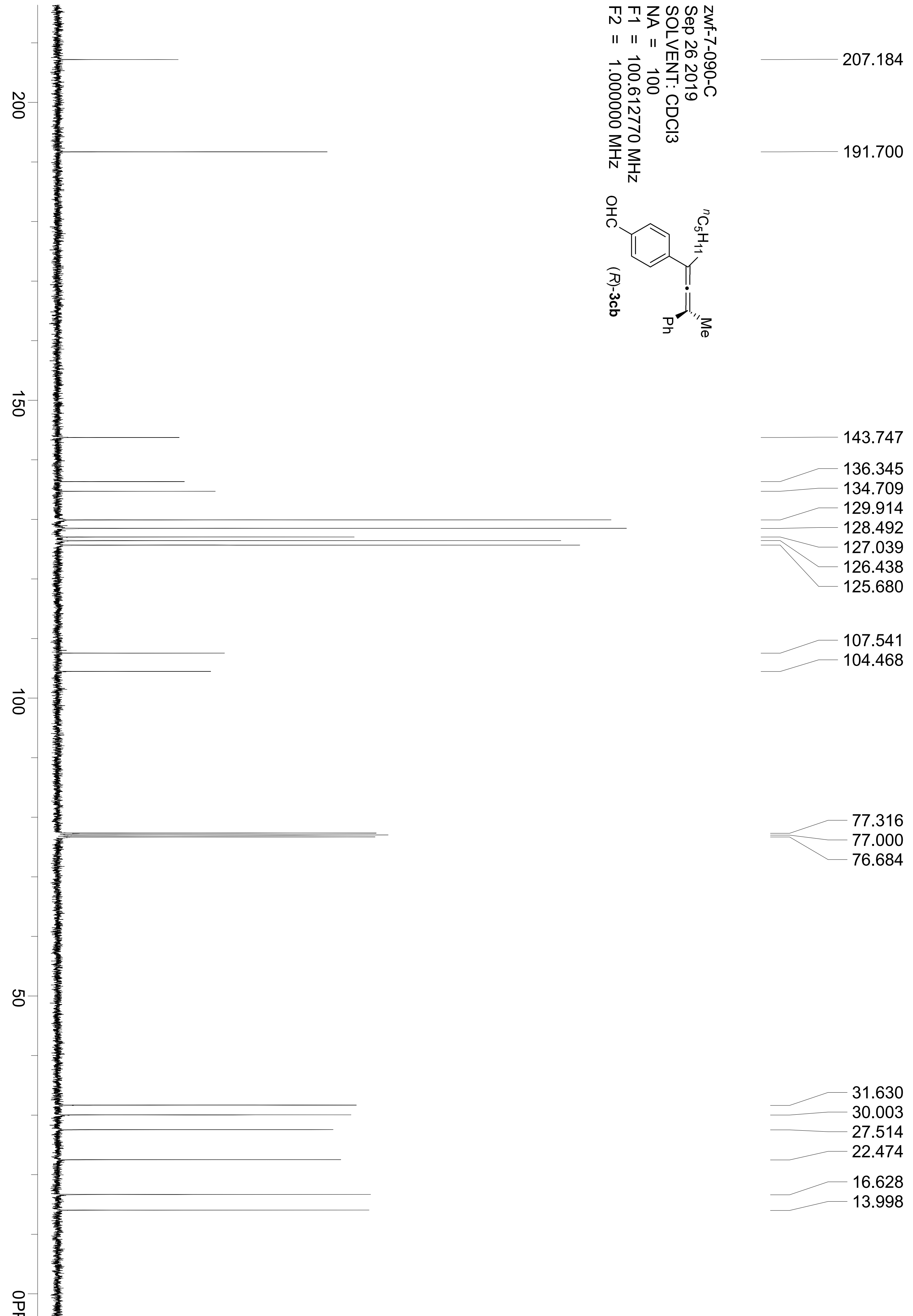

Supplementary Figure 137. ^{13}C NMR (100 MHz, $CDCl_3$) spectrum for (*R*)-**3cb**

Area Percent Report

Agilent Technologies

sample zwf-7-090-OD-H-99.5-0.5-1.0-214

Data file: C:\Users\Public\Documents\ChemStation\1\Data\wgl 2019-09-25 09-43-22\089-P1-C7-zwf-7-090.D

Acquisition Data:

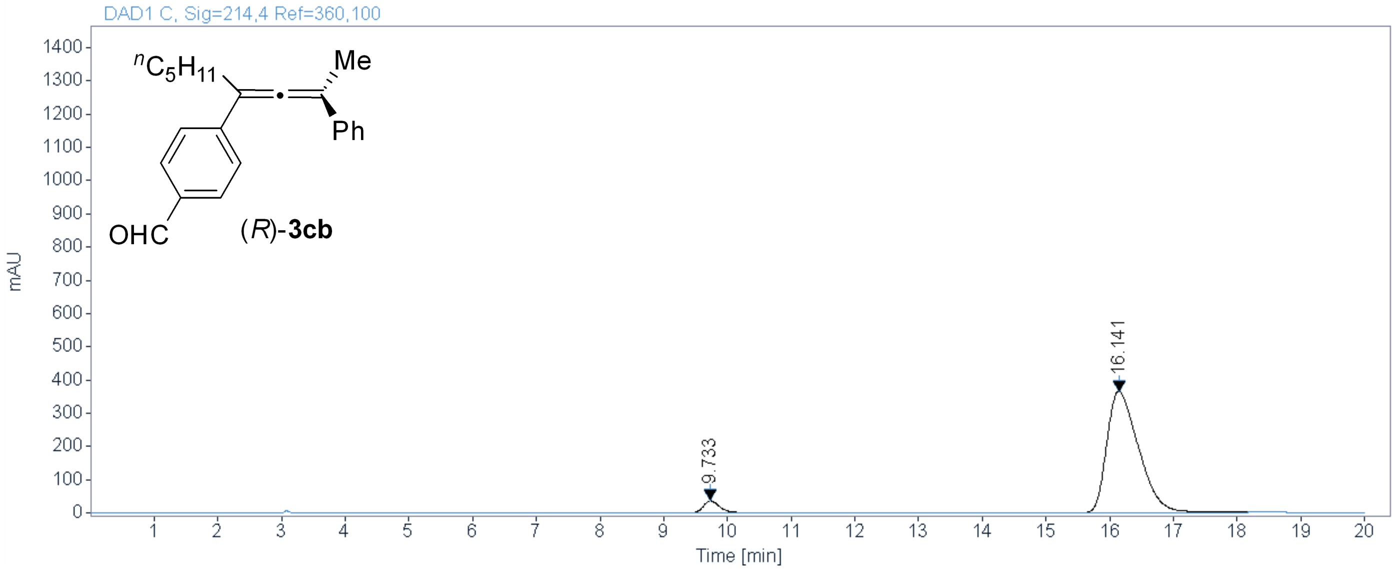

Signal: DAD1 C, Sig=214,4 Ref=360,100

RT [min]	Width [min]	Height	Area	Area%
9.733	0.2847	35.4172	605.0769	4.5140
16.141	0.5834	365.6386	12799.4004	95.4860
		Sum	13404.4773	100.0000

Supplementary Figure 138. HPLC spectrum for (*R*)-**3cb**

Area Percent Report

Agilent Technologies

sample zwf-7-090-rac-OD-H-99.5-0.5-1.0-214

Data file: C:\Users\Public\Documents\ChemStation\1\Data\wgl 2019-09-25 09-43-22\090-P1-C5-zwf-7-090-rac.D

Acquisition Data:

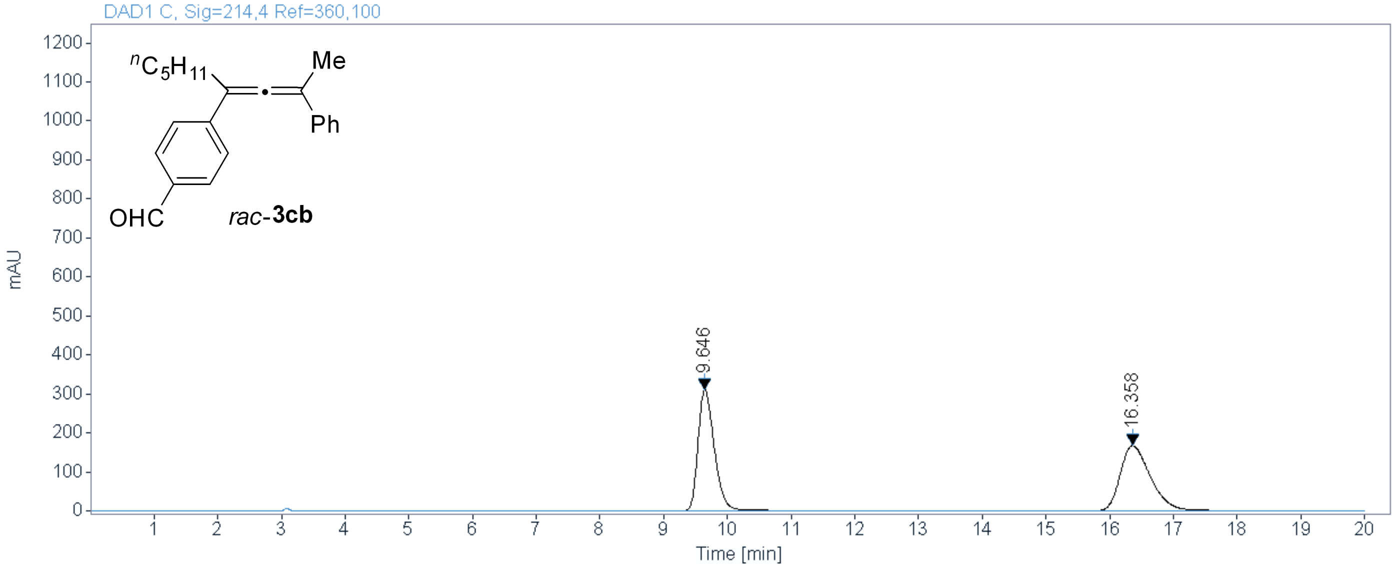

Signal: DAD1 C, Sig=214,4 Ref=360,100

RT [min]	Width [min]	Height	Area	Area%
9.646	0.2701	311.4226	5501.6401	49.7725
16.358	0.5088	167.4717	5551.9253	50.2275
		Sum	11053.5654	100.0000

Supplementary Figure 139. HPLC spectrum for *rac*-**3cb**

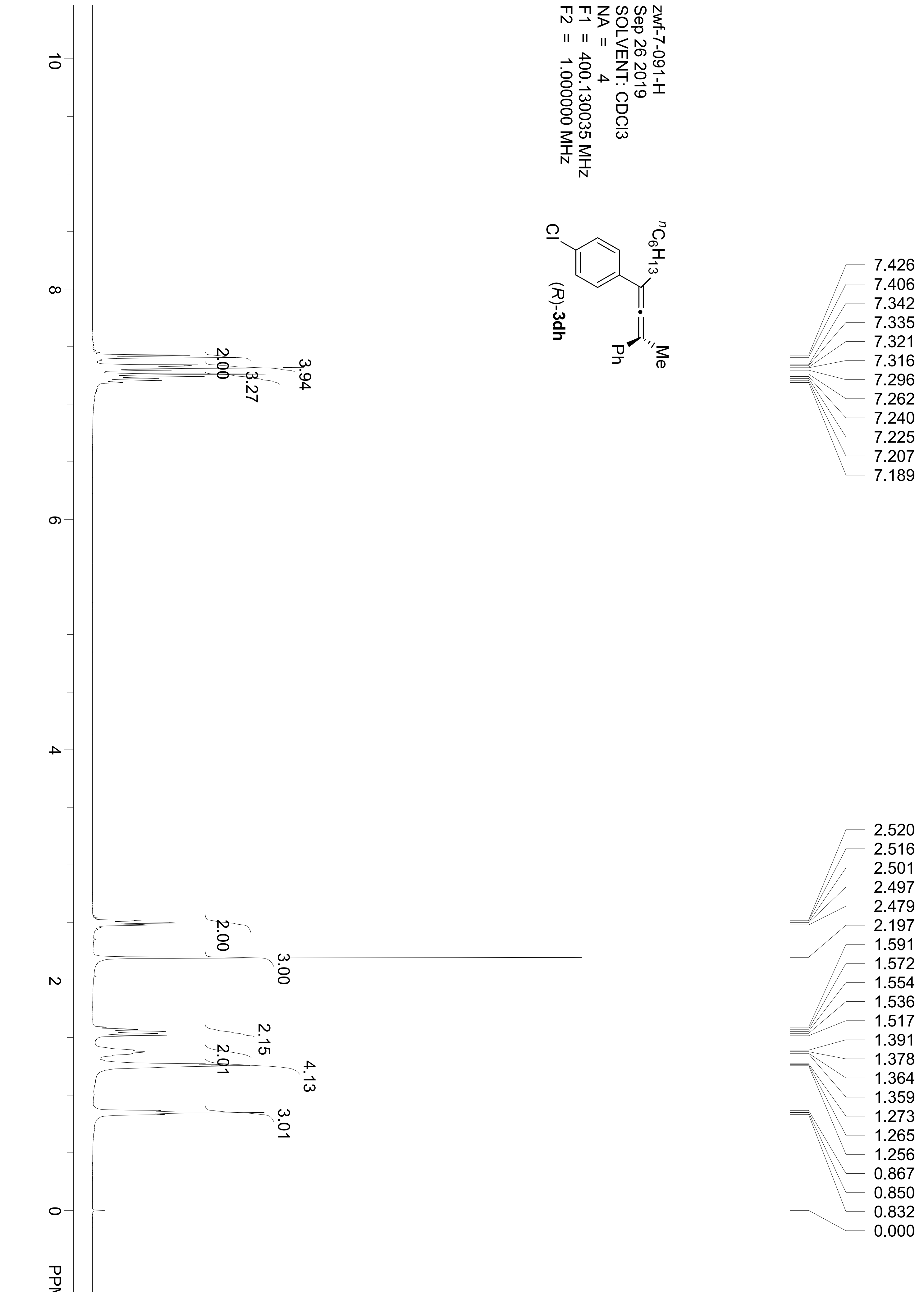

Supplementary Figure 140. ^{1}H NMR (400 MHz, $CDCl_3$) spectrum for (*R*)-**3dh**

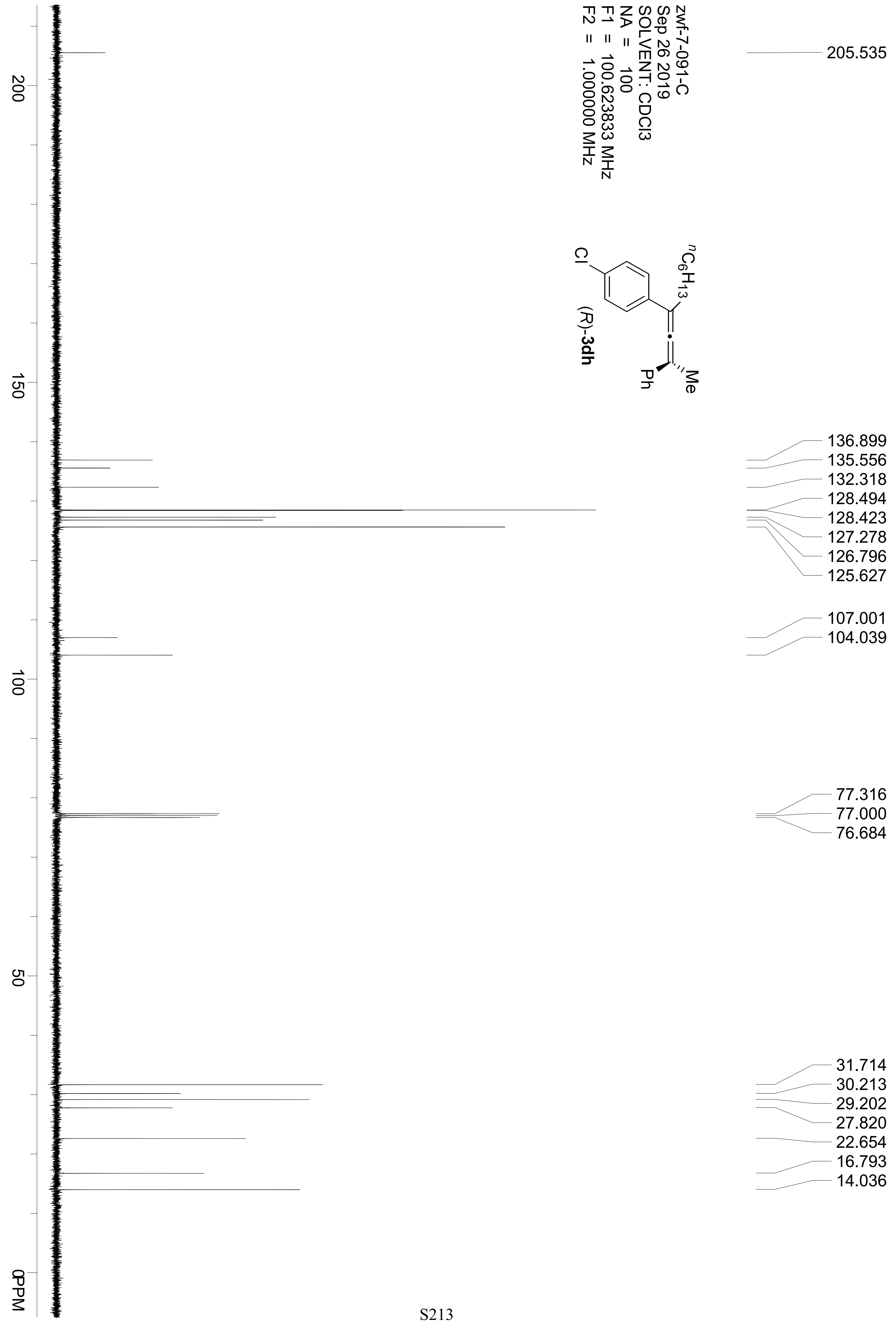

Supplementary Figure 141. ^{13}C NMR (100 MHz, $CDCl_3$) spectrum for (*R*)-**3dh**

Area Percent Report

Agilent Technologies

sample zwf-7-091-OD-H-100-0-0.5-214

Data file: C:\Users\Public\Documents\ChemStation\1\Data\wgl 2019-09-25 09-43-22\092-P1-C8-zwf-7-091.D

Acquisition Data:

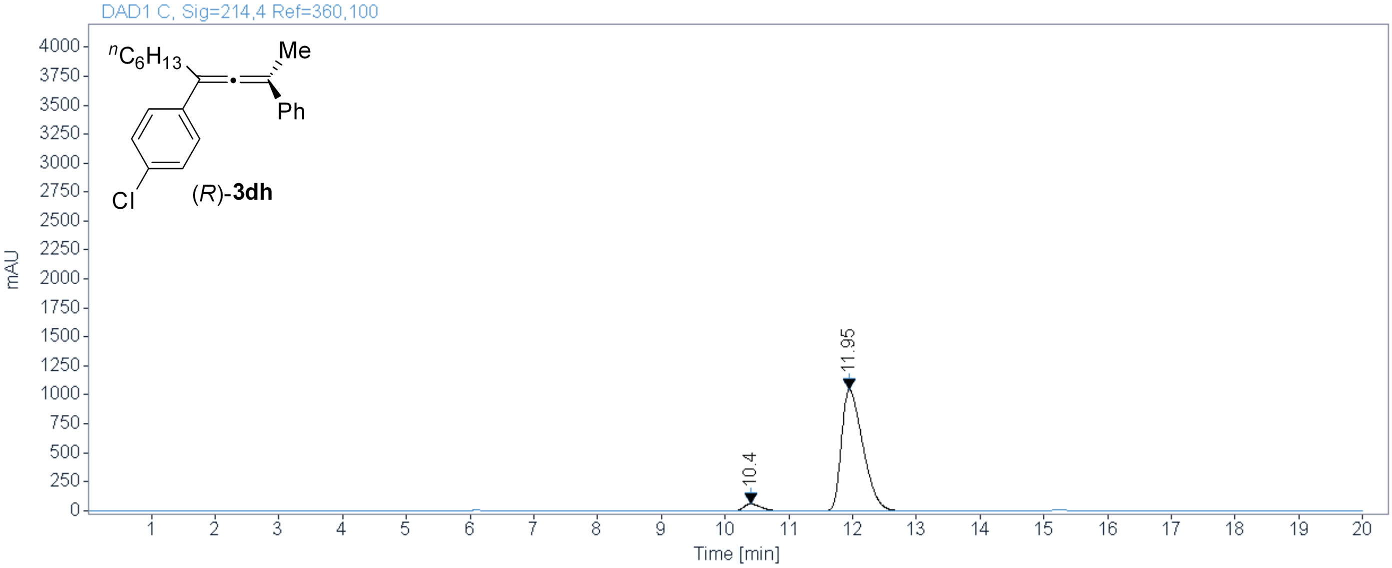

Signal: DAD1 C, Sig=214,4 Ref=360,100

RT [min]	Width [min]	Height	Area	Area%
10.400	0.3320	62.1780	1238.6973	4.7917
11.950	0.3907	1049.8787	24612.3008	95.2083
		Sum	25850.9980	100.0000

Supplementary Figure 142. HPLC spectrum for (*R*)-**3dh**

Area Percent Report

sample zwf-7-091-rac-OD-H-100-0-0.5-214

Data file: C:\Users\Public\Documents\ChemStation\1\Data\wgl 2019-09-25 09-43-22\093-P1-C6-zwf-7-091-rac.D

Acquisition Data:

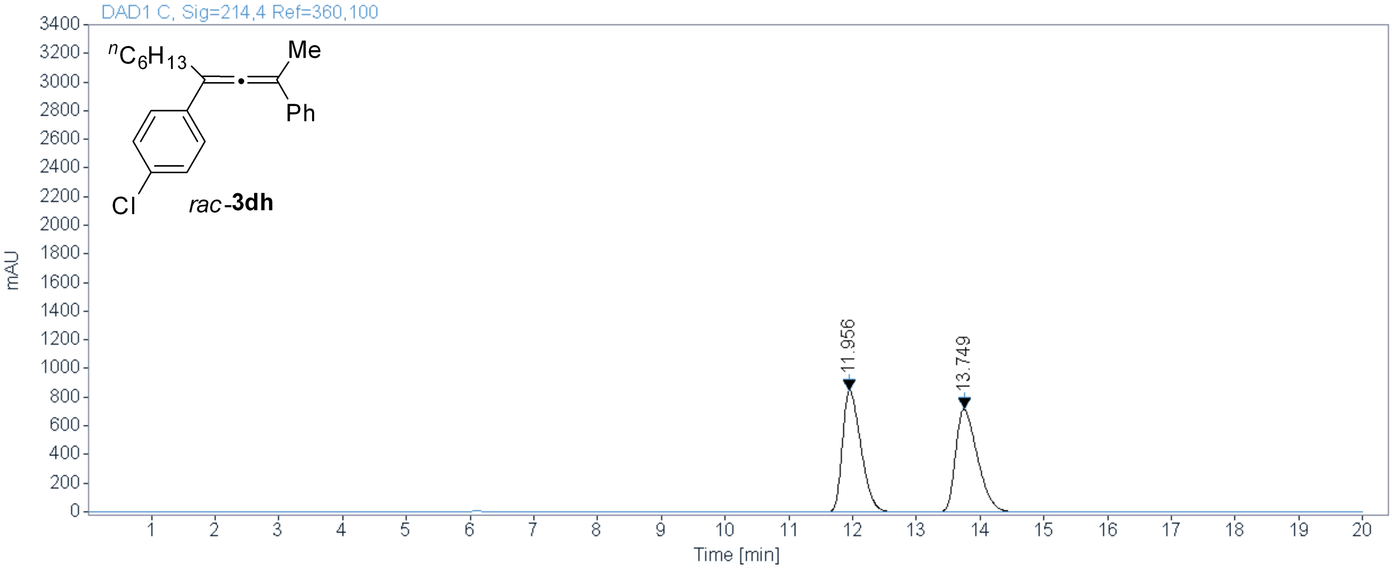

Signal: DAD1 C, Sig=214,4 Ref=360,100

RT [min]	Width [min]	Height	Area	Area%
11.956	0.3134	850.6918	17161.4609	49.9627
13.749	0.3715	717.5137	17187.1074	50.0373
		Sum	34348.5684	100.0000

Supplementary Figure 143. HPLC spectrum for *rac*-**3dh**

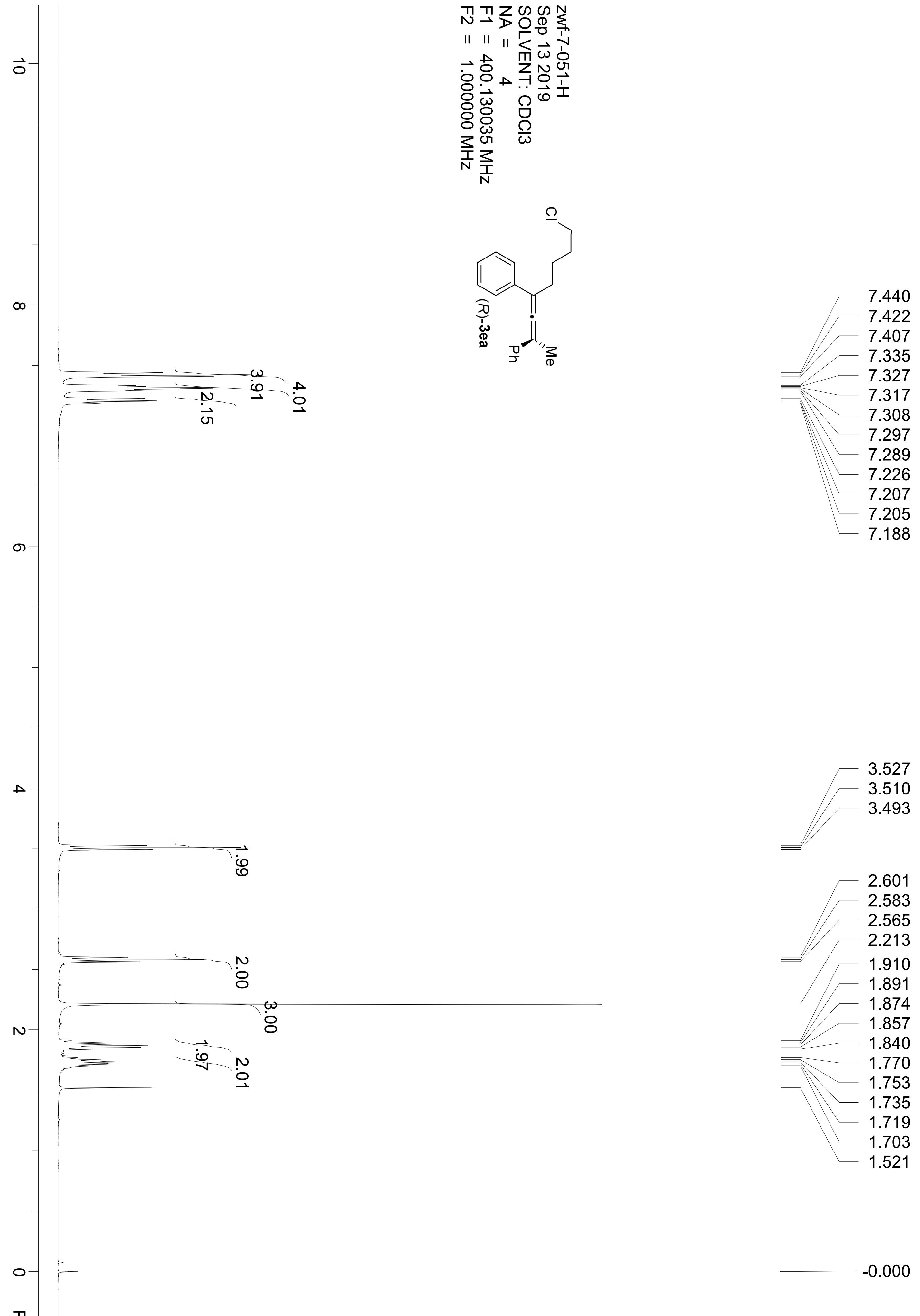

Supplementary Figure 144. ^{1}H NMR (400 MHz, $CDCl_3$) spectrum for (*R*)-**3ea**

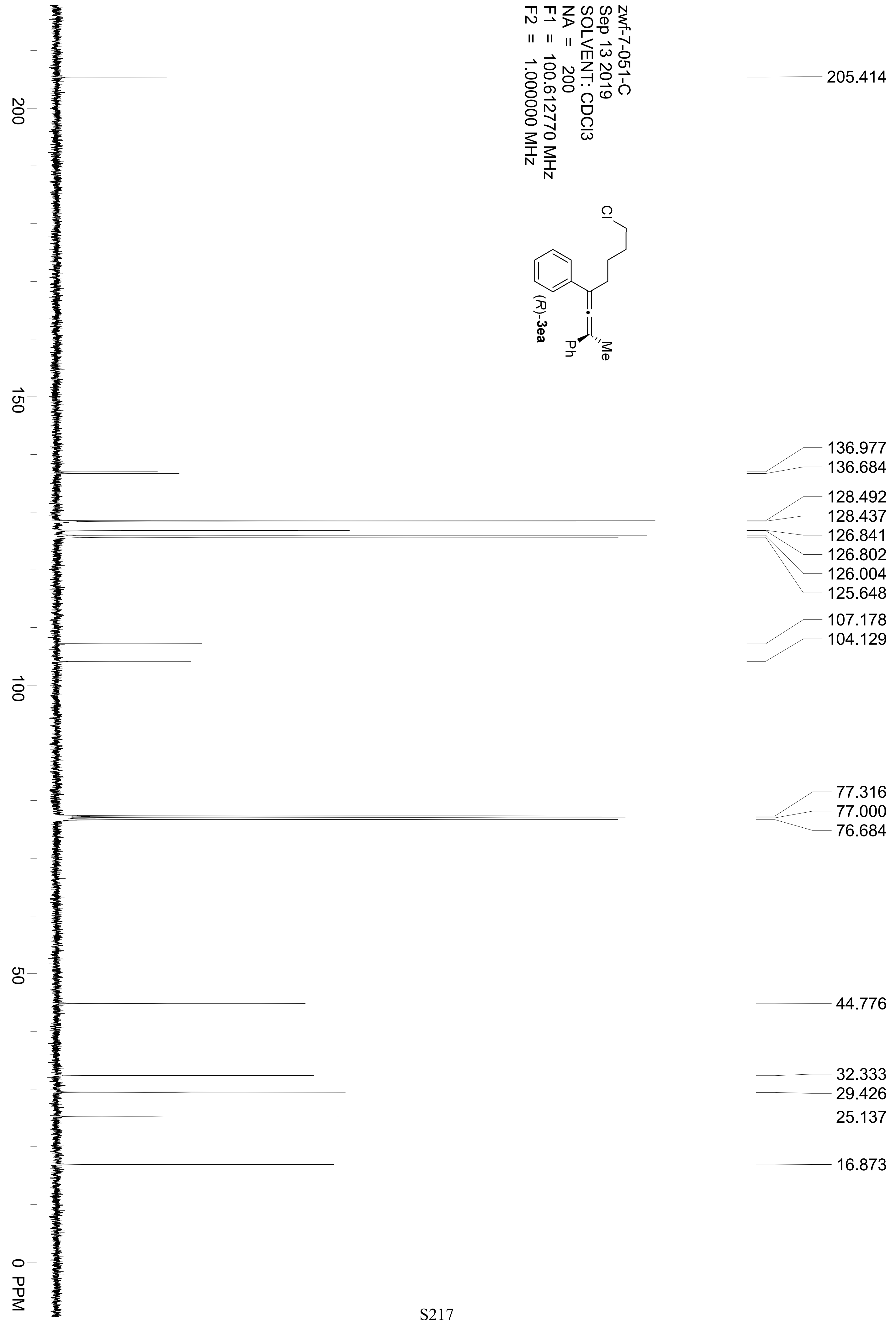

Supplementary Figure 145. ^{13}C NMR (100 MHz, $CDCl_3$) spectrum for (*R*)-**3ea**

Area Percent Report

Agilent Technologies

sample zwf-7-051-AD-H-99.5-0.5-0.5-214

Data file: C:\Users\Public\Documents\ChemStation\1\Data\zwf-allenioc acid_LC 2019-09-13 08-53-14\008-P1-C3-zwf-7-051.D

Acquisition Data:

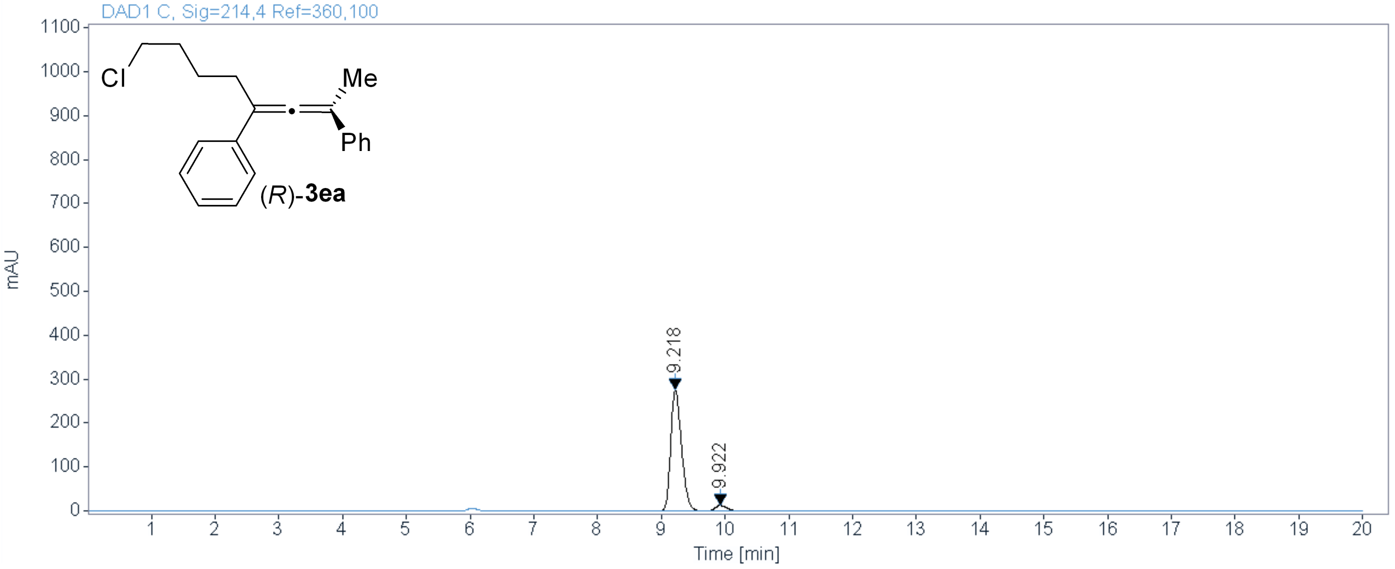

Signal: DAD1 C, Sig=214,4 Ref=360,100

RT [min]	Width [min]	Height	Area	Area%
9.218	0.1820	277.2317	3279.2808	95.2315
9.922	0.1957	12.7995	164.2011	4.7685
		Sum	3443.4819	100.0000

Supplementary Figure 146. HPLC spectrum for (*R*)-**3ea**

Area Percent Report

Agilent Technologies

sample zwf-7-051-rac-AD-H-99.5-0.5-0.5-214

Data file: C:\Users\Public\Documents\ChemStation\1\Data\zwf-allenioc acid_LC 2019-09-13 08-53-14\009-P1-C4-zwf-7-051-rac.D

Acquisition Data:

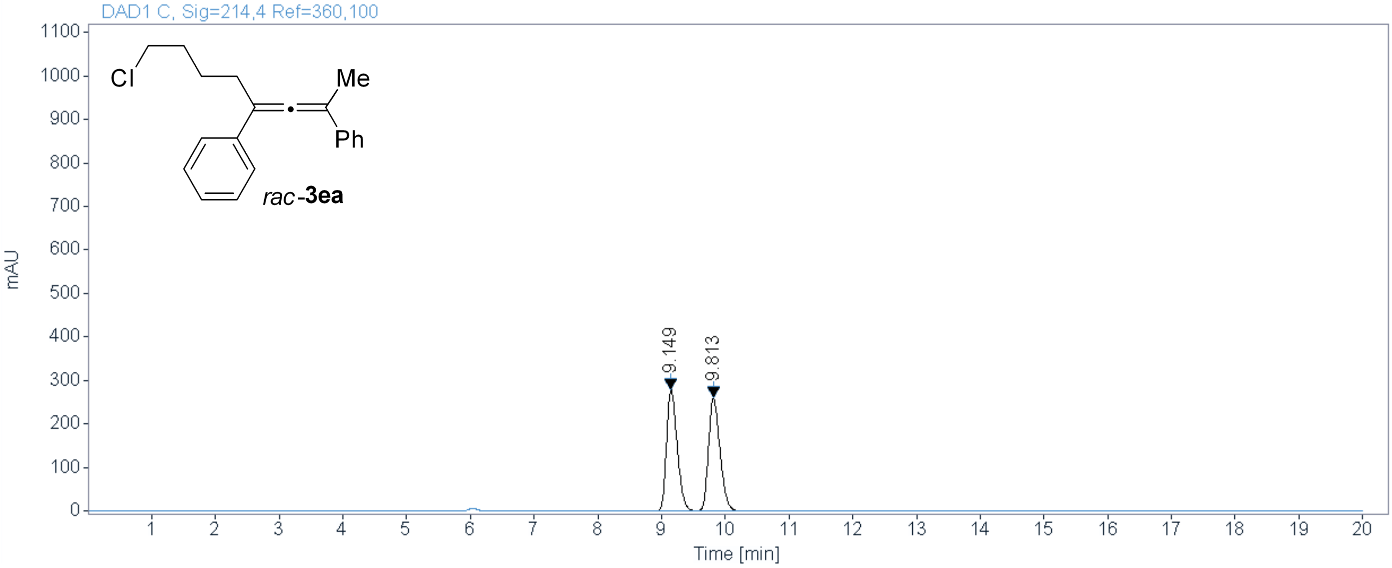

Signal: DAD1 C, Sig=214,4 Ref=360,100

RT [min]	Width [min]	Height	Area	Area%
9.149	0.1797	279.6477	3253.5459	50.0422
9.813	0.1920	259.5050	3248.0554	49.9578
		Sum	6501.6013	100.0000

Supplementary Figure 147. HPLC spectrum for *rac*-**3ea**

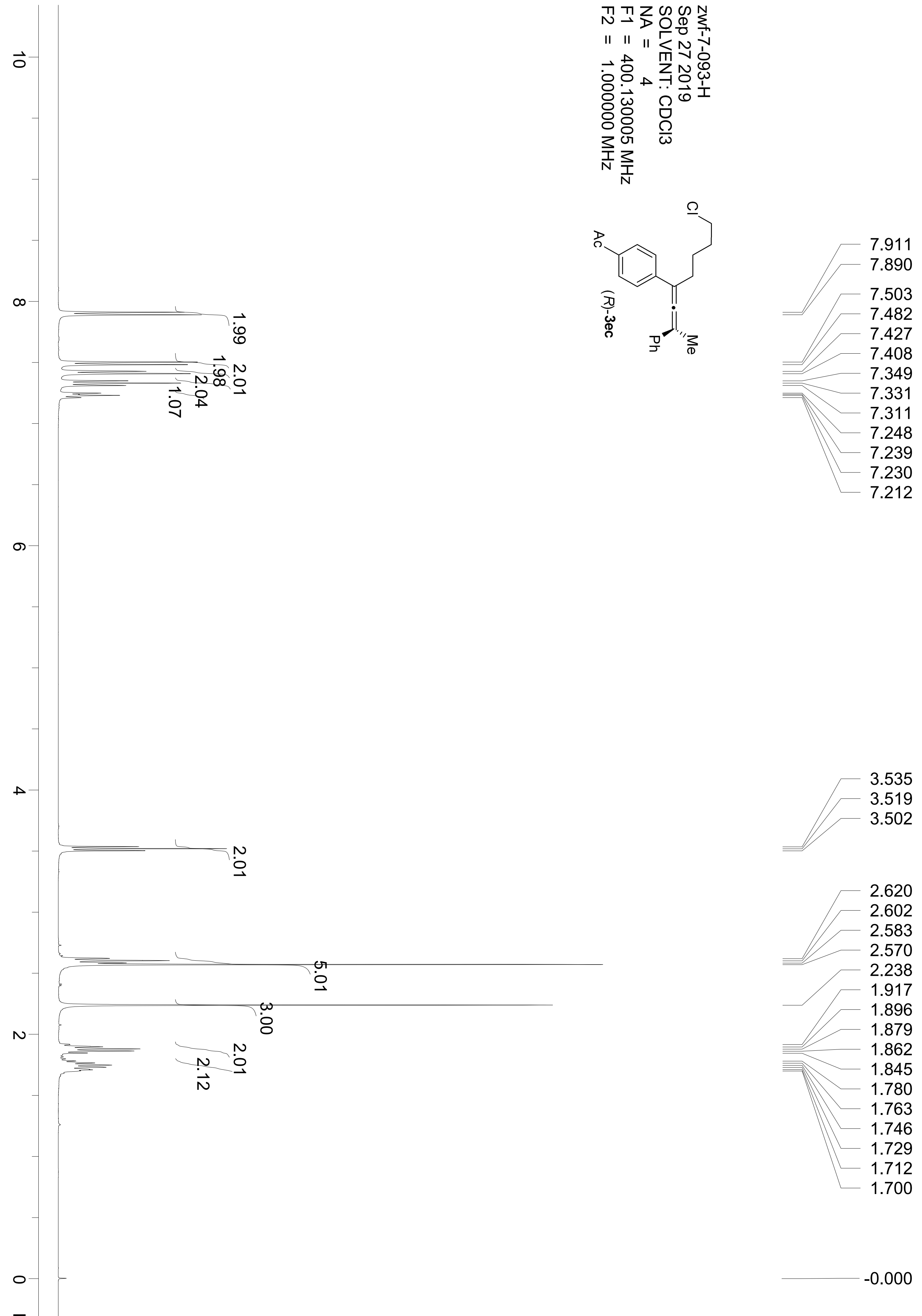

Supplementary Figure 148. ^{1}H NMR (400 MHz, $CDCl_3$) spectrum for (*R*)-**3ec**

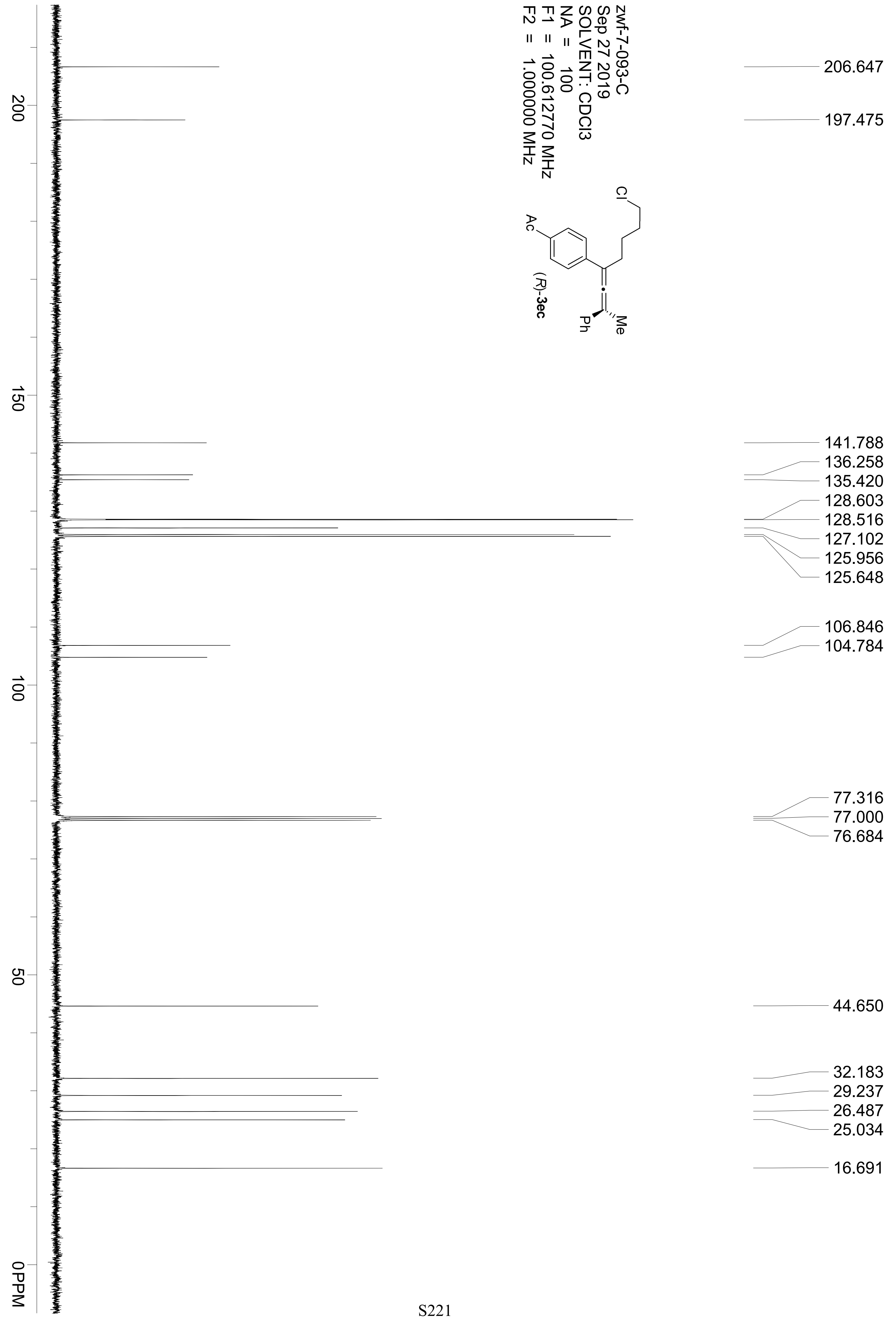

Supplementary Figure 149. ^{13}C NMR (100 MHz, $CDCl_3$) spectrum for (*R*)-**3ec**

Area Percent Report

Agilent Technologies

sample zwf-7-093-OD-H-98-2-1.0-214

Data file: C:\Users\Public\Documents\ChemStation\1\Data\hcf 2019-09-27 09-42-39\008-P1-C2-zwf-7-093.D

Acquisition Data:

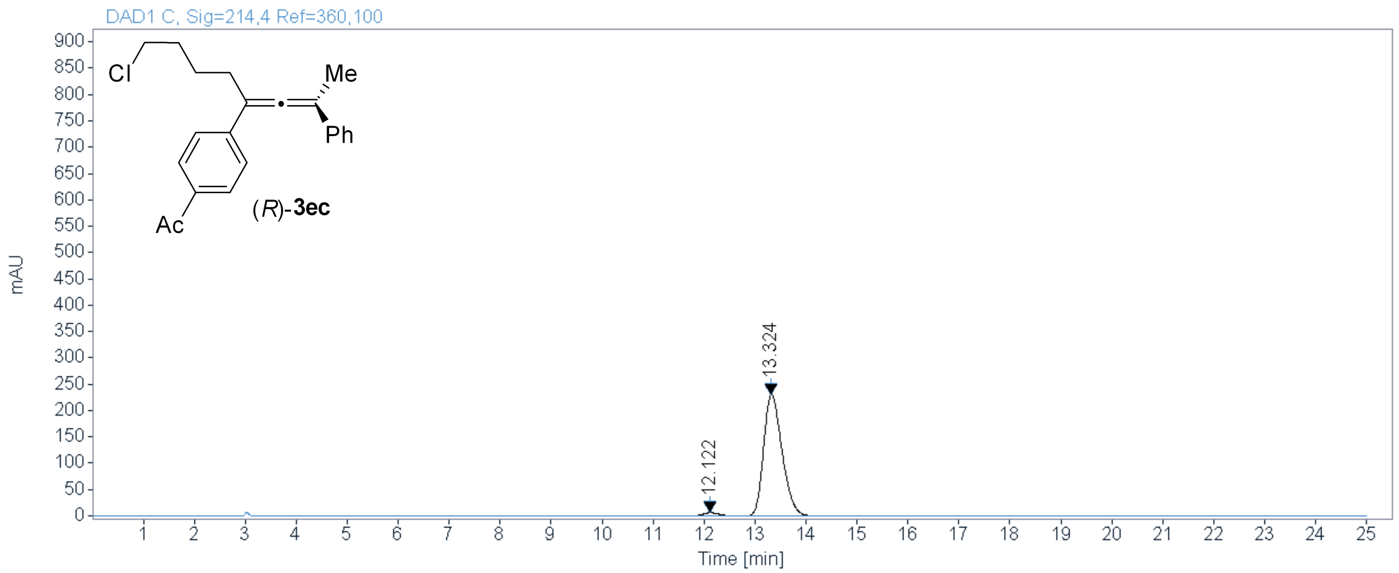

Signal: DAD1 C, Sig=214,4 Ref=360,100

RT [min]	Width [min]	Height	Area	Area%
12.122	0.3561	6.2297	142.0889	2.3805
13.324	0.3899	231.2650	5826.7412	97.6195
		Sum	5968.8301	100.0000

Supplementary Figure 150. HPLC spectrum for (*R*)-**3ec**

Area Percent Report

Agilent Technologies

sample zwf-7-093-rac-OD-H-98-2-1.0-214

Data file: C:\Users\Public\Documents\ChemStation\1\Data\hcf 2019-09-27 09-42-39\009-P1-C1-zwf-7-093-rac.D

Acquisition Data:

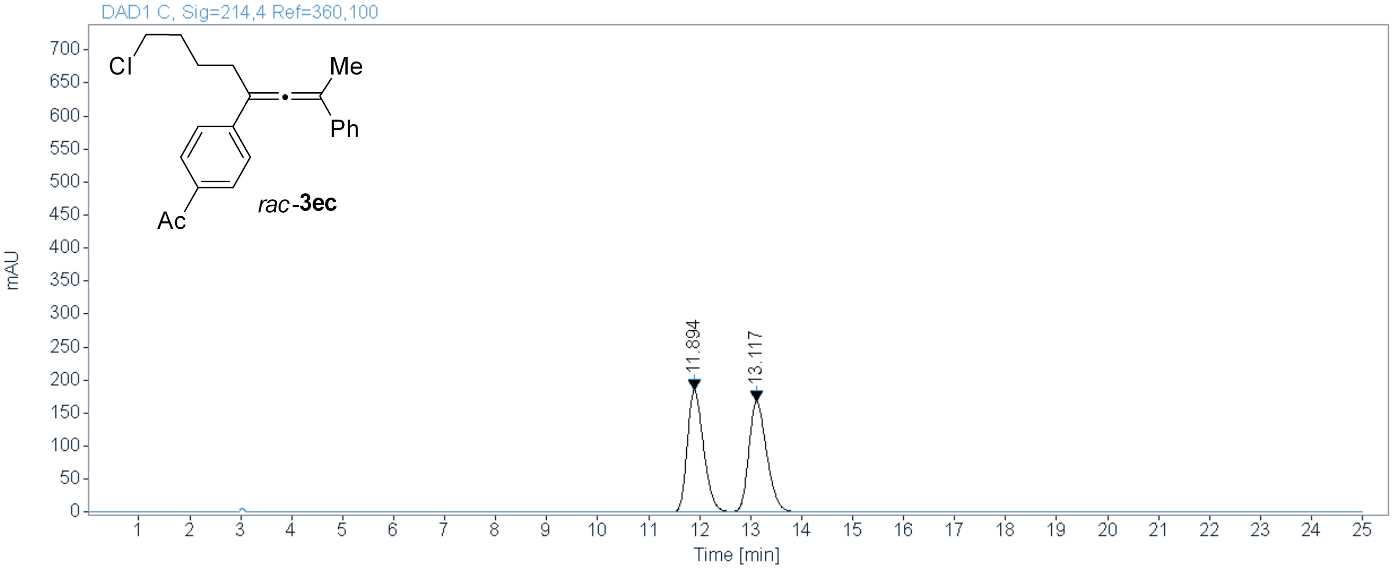

Signal: DAD1 C, Sig=214,4 Ref=360,100

RT [min]	Width [min]	Height	Area	Area%
11.894	0.3427	184.8089	4097.2197	49.9139
13.117	0.3800	167.7157	4111.3525	50.0861
		Sum	8208.5723	100.0000

Supplementary Figure 151. HPLC spectrum for *rac*-**3ec**

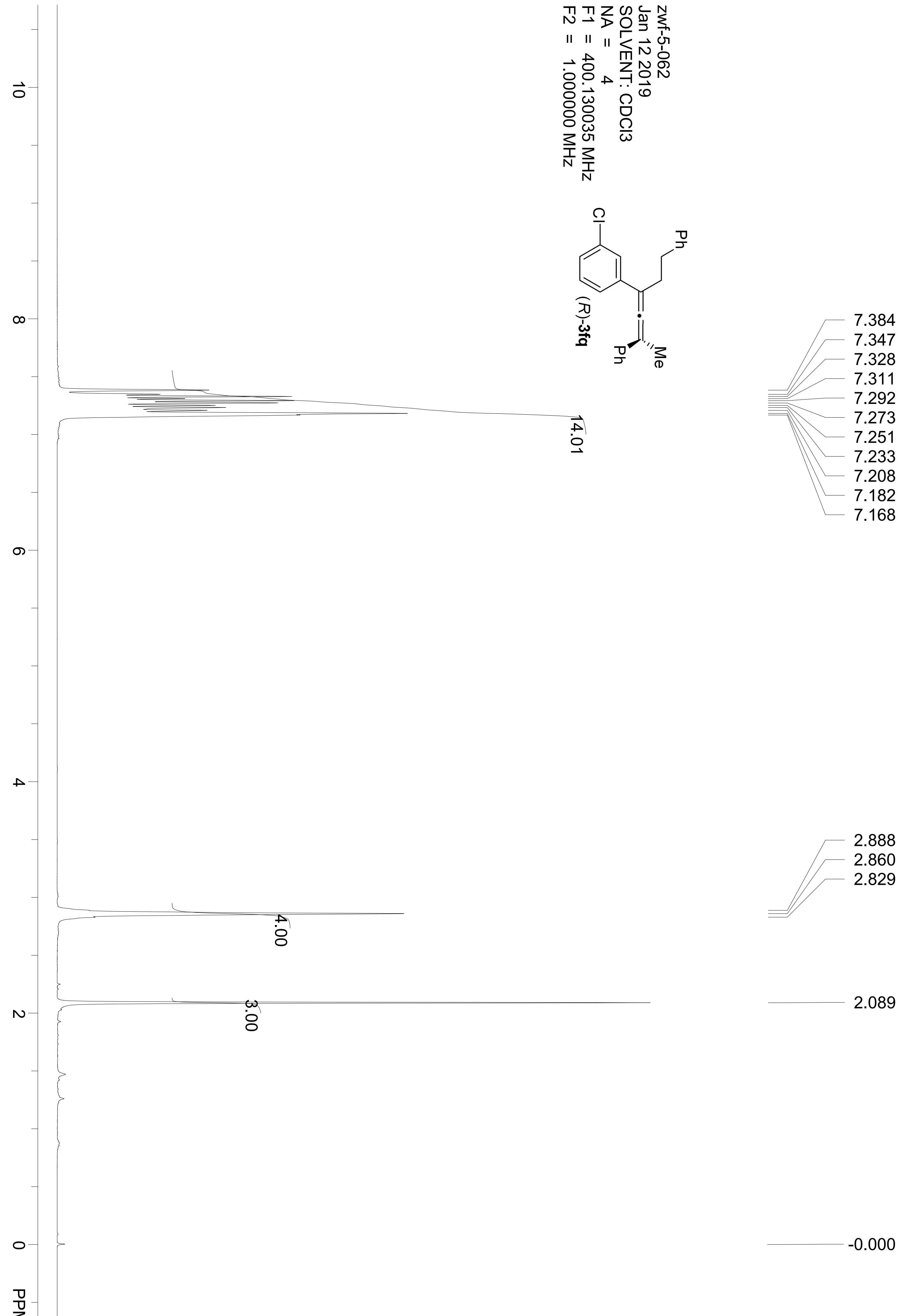

Supplementary Figure 152. ^{1}H NMR (400 MHz, $CDCl_3$) spectrum for (*R*)-**3fq**

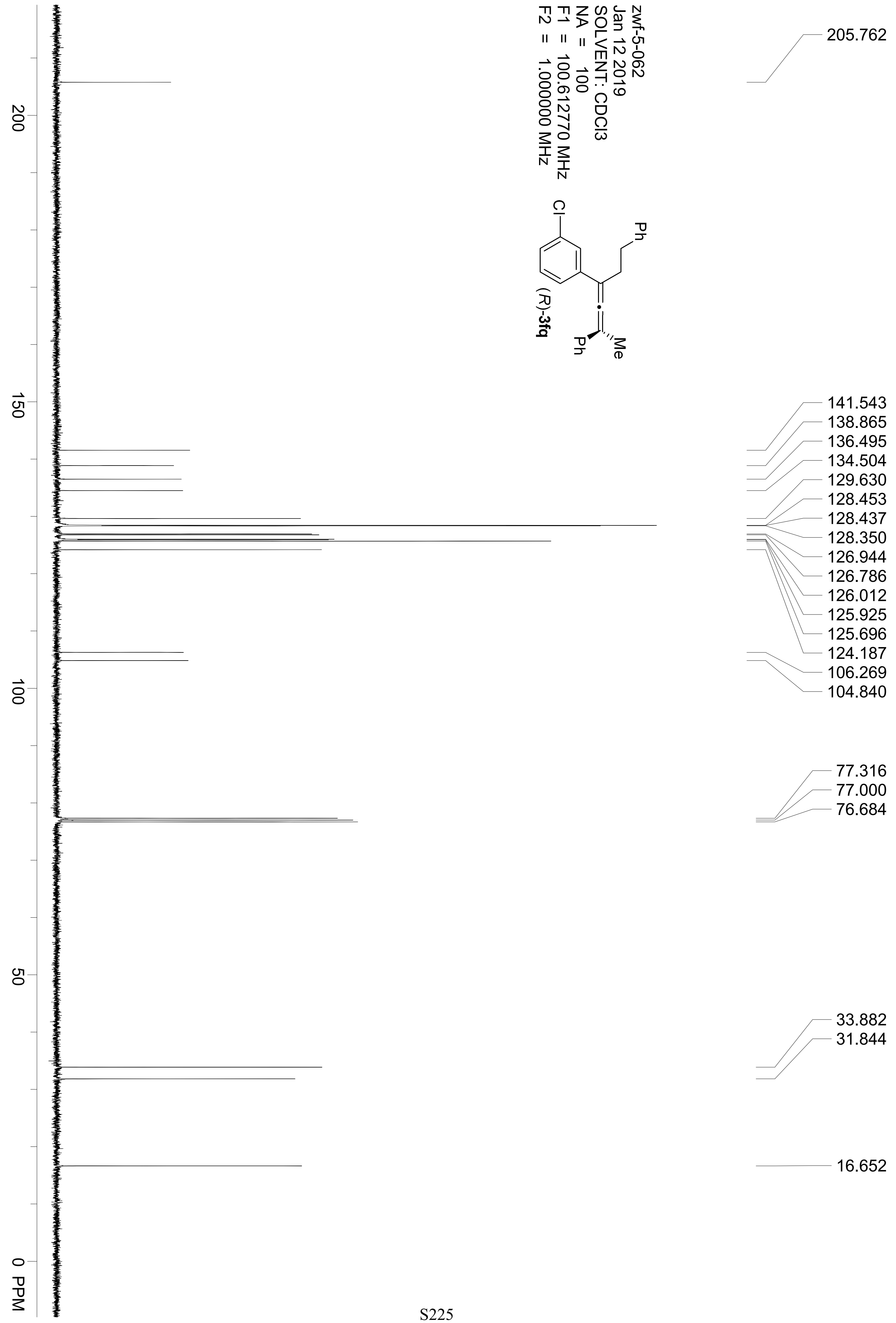

Supplementary Figure 153. ^{13}C NMR (100 MHz, $CDCl_3$) spectrum for (*R*)-**3fq**

Area Percent Report

sample zwf-7-052-OD-H-99.5-0.5-1.0-214

Data file: C:\Users\Public\Documents\ChemStation\1\Data\zwf-allenioc acid_LC 2019-09-13 08-53-14\012-P1-C5-zwf-7-052.D

Acquisition Data:

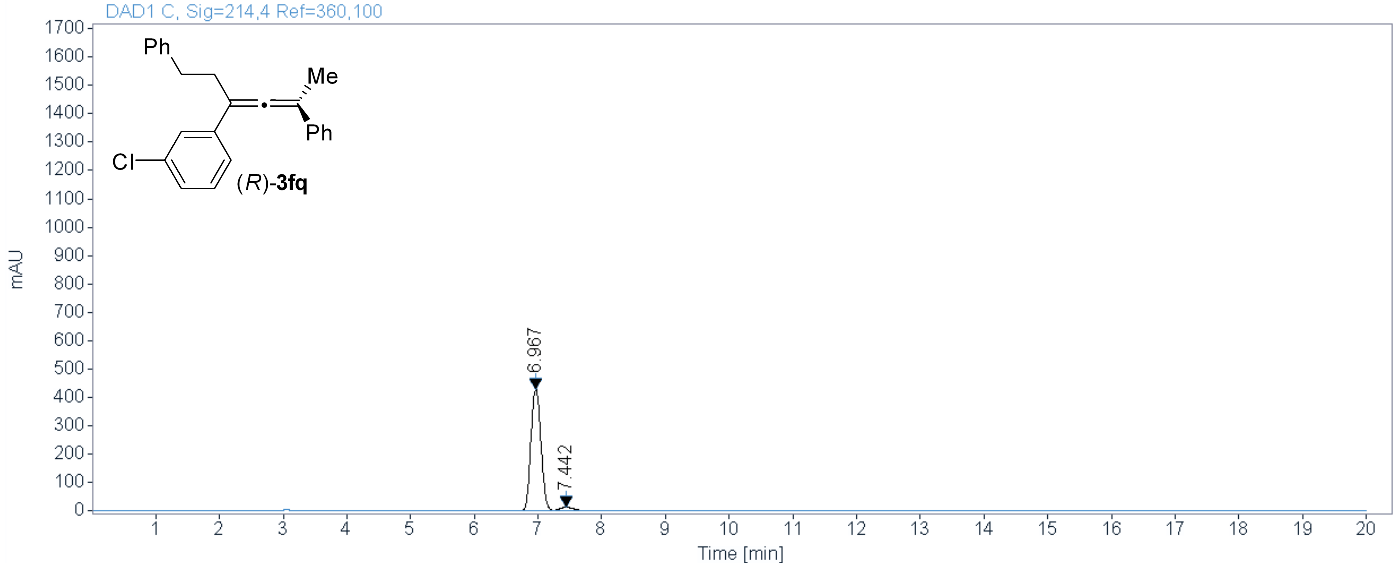

Signal: DAD1 C, Sig=214,4 Ref=360,100

RT [min]	Width [min]	Height	Area	Area%
6.967	0.1832	429.6477	4723.0391	95.9347
7.442	0.2702	12.3469	200.1413	4.0653
		Sum	4923.1804	100.0000

Supplementary Figure 154. HPLC spectrum for (*R*)-**3fq**

Area Percent Report

Agilent Technologies

sample zwf-7-052-rac-OD-H-99.5-0.5-1.0-214

Data file: C:\Users\Public\Documents\ChemStation\1\Data\zwf-allenioc acid_LC 2019-09-13 08-53-14\014-P1-C6-zwf-7-052-rac.D

Acquisition Data:

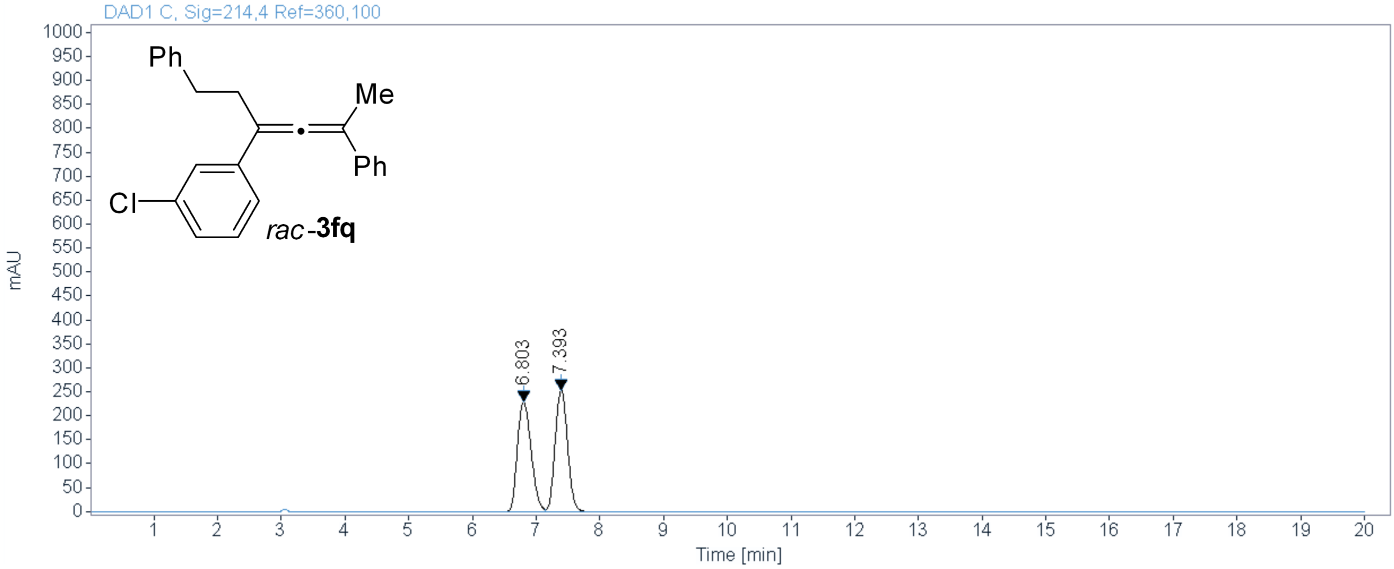

Signal: DAD1 C, Sig=214,4 Ref=360,100

RT [min]	Width [min]	Height	Area	Area%
6.803	0.2414	229.7916	3494.3013	50.1273
7.393	0.2177	253.9363	3476.5483	49.8727
		Sum	6970.8496	100.0000

Supplementary Figure 155. HPLC spectrum for *rac*-**3fq**

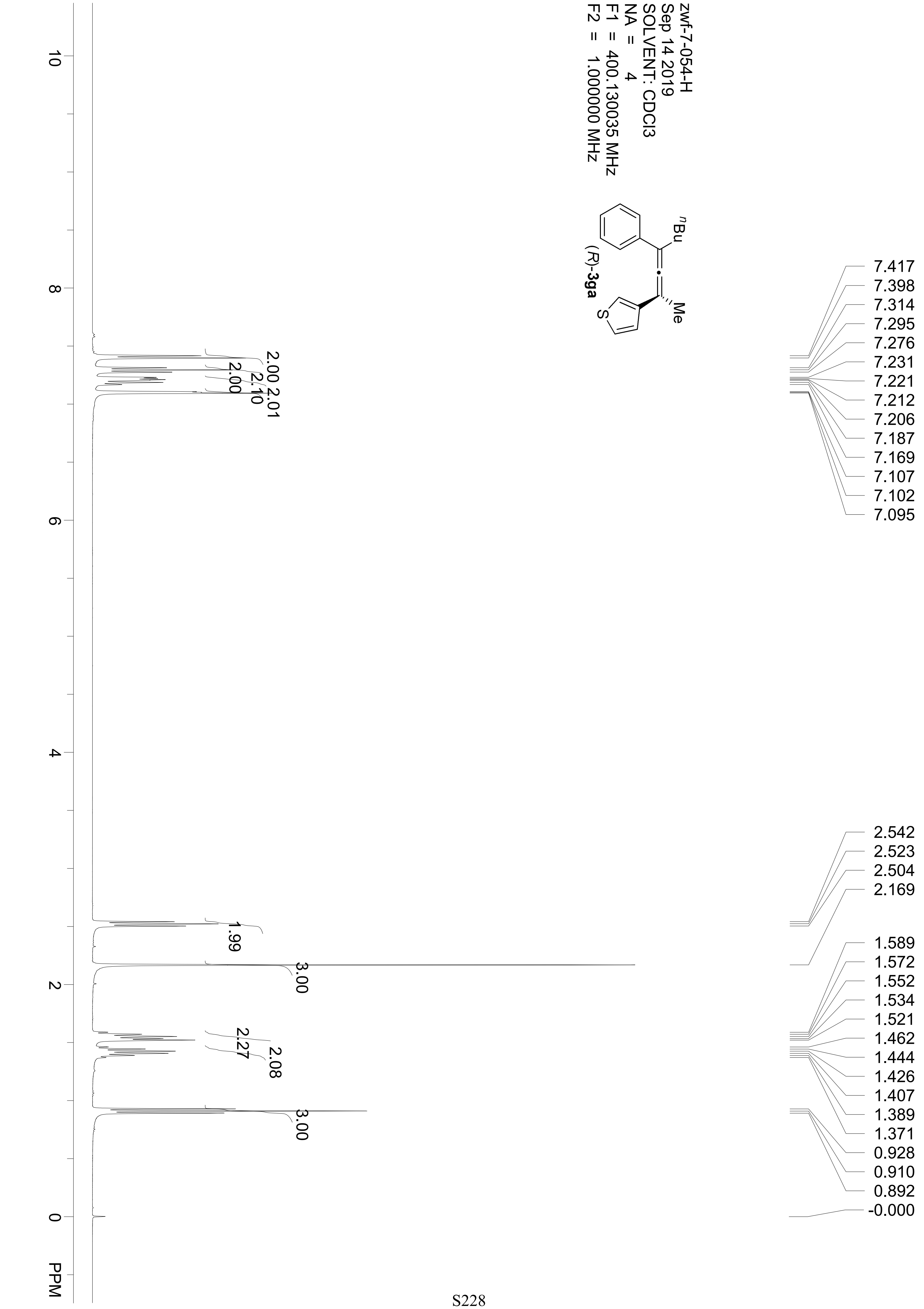

Supplementary Figure 156. ^{1}H NMR (400 MHz, $CDCl_3$) spectrum for (*R*)-**3ga**

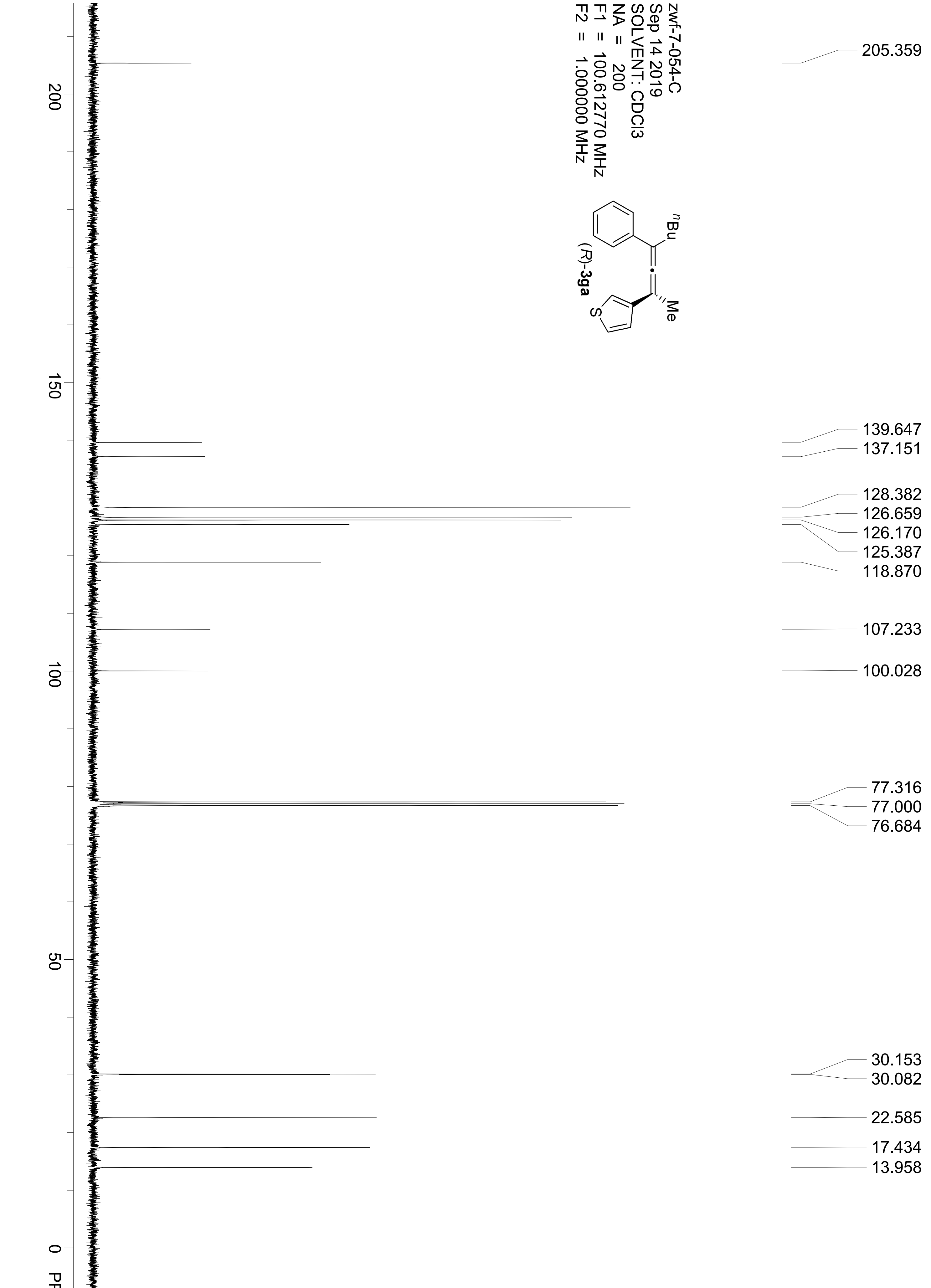

Supplementary Figure 157. ^{13}C NMR (100 MHz, $CDCl_3$) spectrum for (*R*)-**3ga**

Area Percent Report

Agilent Technologies

sample zwf-7-054-AD-H-99.5-0.5-0.5-214

Data file: C:\Users\Public\Documents\ChemStation\1\Data\WHN 2019-09-14 08-20-59\028-P1-C1-zwf-7-054.D

Acquisition Data:

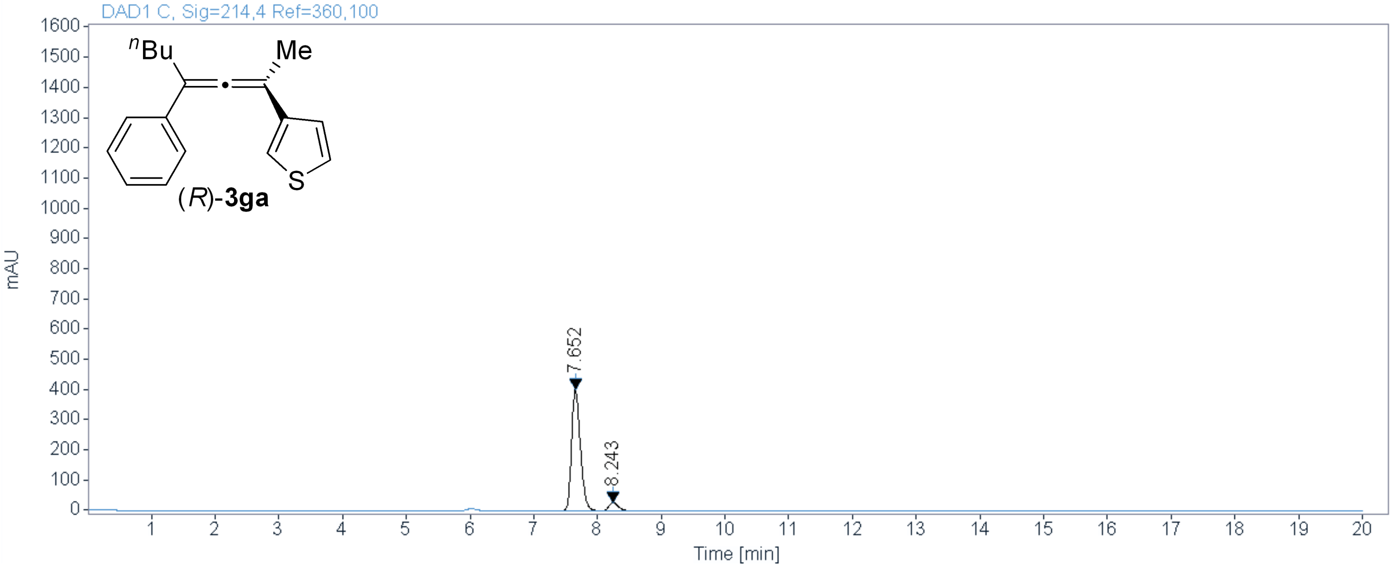

Signal: DAD1 C, Sig=214,4 Ref=360,100

RT [min]	Width [min]	Height	Area	Area%
7.652	0.1466	402.2389	3883.3853	93.2644
8.243	0.1596	26.9167	280.4589	6.7356
		Sum	4163.8442	100.0000

Supplementary Figure 158. HPLC spectrum for (*R*)-**3ga**

Area Percent Report

Agilent Technologies

sample zwf-7-054-rac-AD-H-99.5-0.5-0.5-214

Data file: C:\Users\Public\Documents\ChemStation\1\Data\WHN 2019-09-14 08-20-59\029-P1-C2-zwf-7-054-rac.D

Acquisition Data:

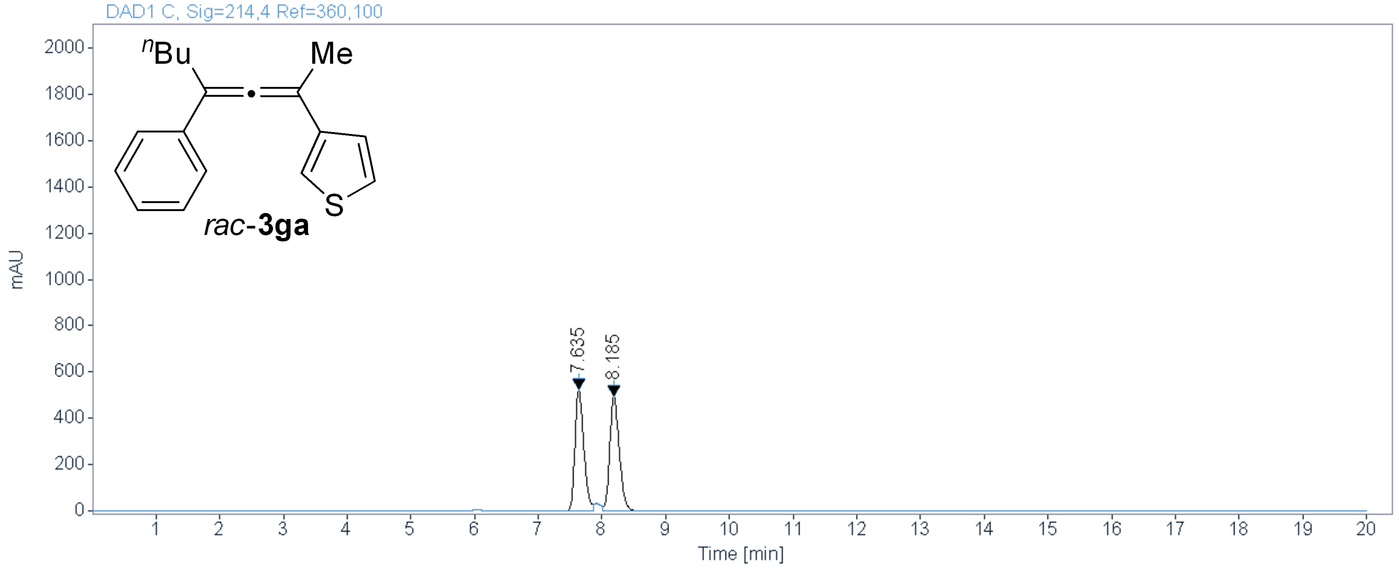

Signal: DAD1 C, Sig=214,4 Ref=360,100

RT [min]	Width [min]	Height	Area	Area%
7.635	0.1612	526.0174	5086.2109	49.5945
8.185	0.1598	495.0497	5169.3809	50.4055
		Sum	10255.5918	100.0000

Supplementary Figure 159. HPLC spectrum for *rac*-**3ga**

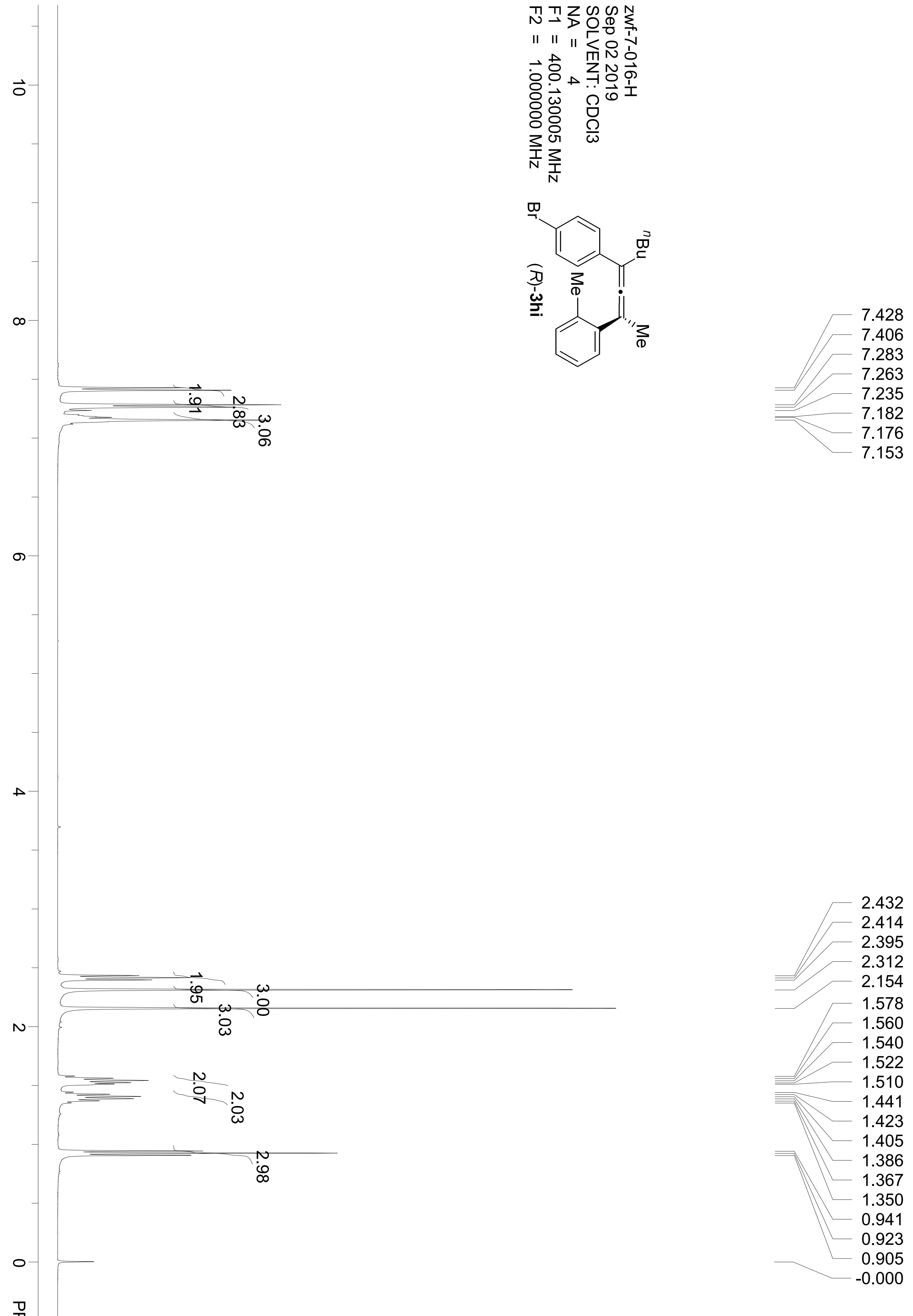

Supplementary Figure 160. ^{1}H NMR (400 MHz, $CDCl_3$) spectrum for (*R*)-**3hi**

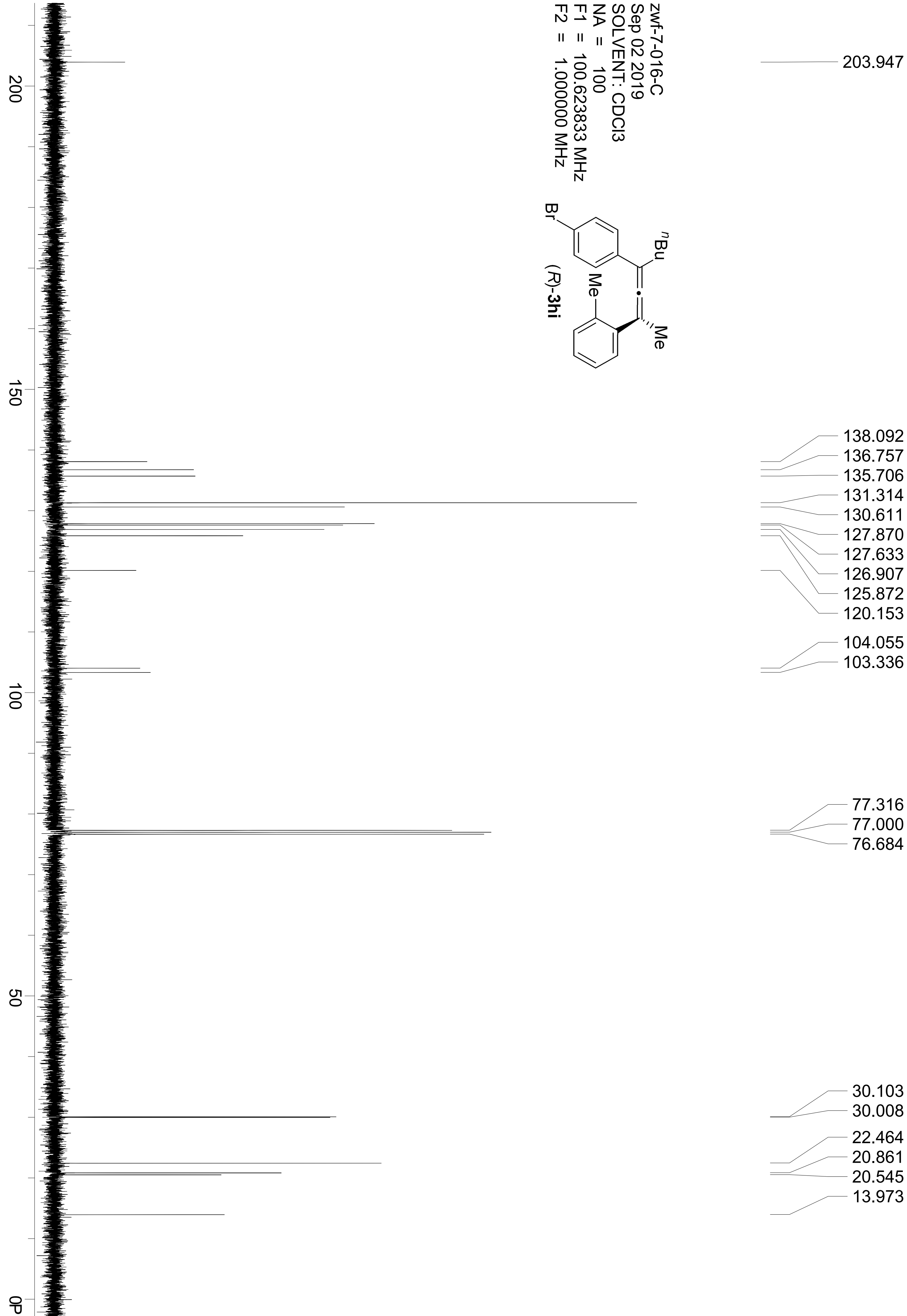

Supplementary Figure 161. ^{13}C NMR (100 MHz, $CDCl_3$) spectrum for (*R*)-**3hi**

Area Percent Report

sample zwf-7-016-AD-H-99.5-0.5-0.5-214

Data file: C:\Users\Public\Documents\ChemStation\1\Data\wgl 2019-09-03 07-35-18\034-P1-C7-zwf-7-016.D

Acquisition Data:

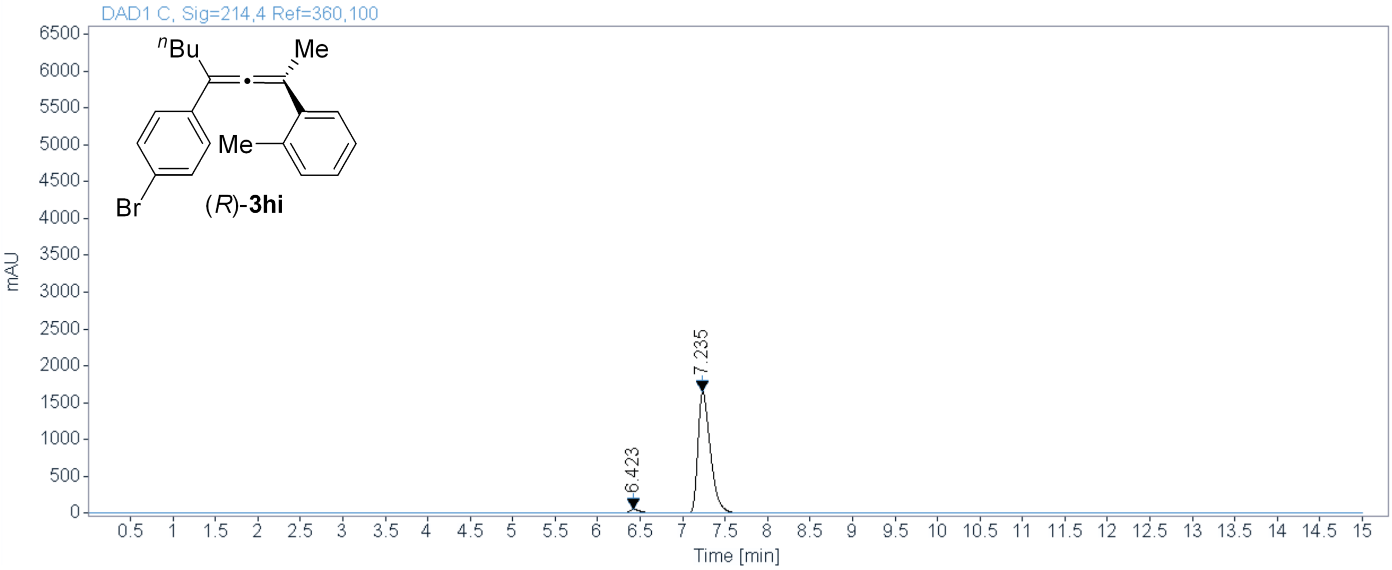

Signal: DAD1 C, Sig=214,4 Ref=360,100

RT [min]	Width [min]	Height	Area	Area%
6.423	0.1528	49.0613	449.6916	2.5944
7.235	0.1550	1654.6190	16883.4375	97.4056
		Sum	17333.1291	100.0000

Supplementary Figure 162. HPLC spectrum for (*R*)-**3hi**

Area Percent Report

Agilent Technologies

sample zwf-7-016-rac-AD-H-99.5-0.5-0.5-214

Data file: C:\Users\Public\Documents\ChemStation\1\Data\wgl 2019-09-03 07-35-18\035-P1-C8-zwf-7-016-rac.D

Acquisition Data:

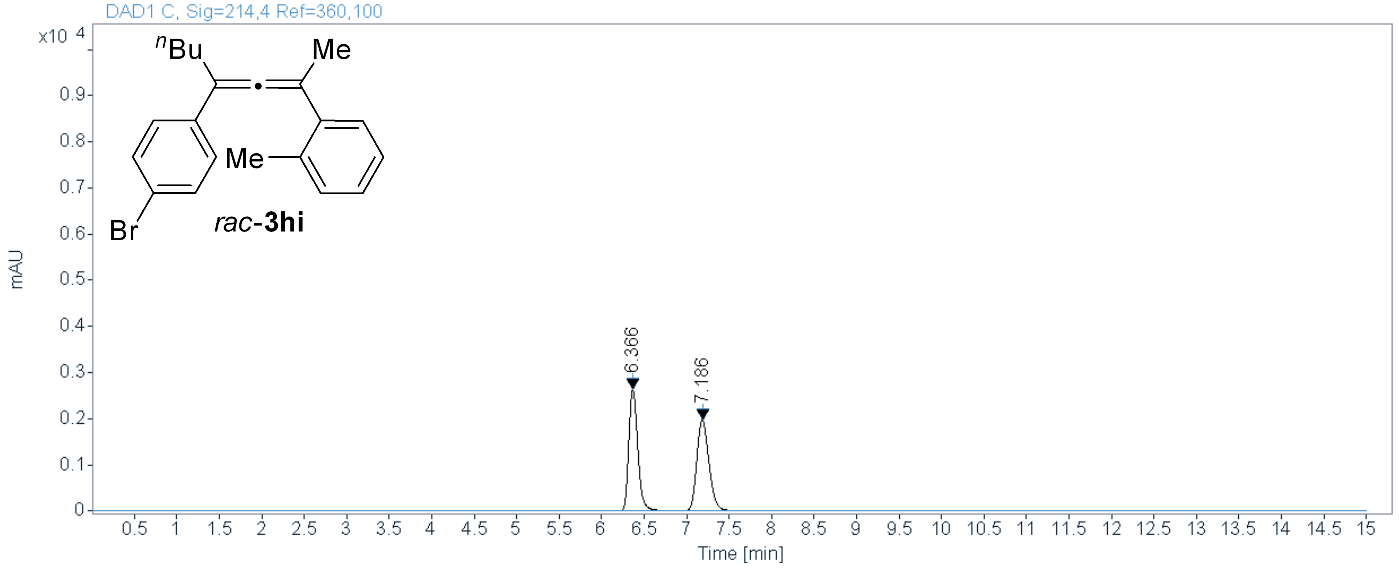

Signal: DAD1 C, Sig=214,4 Ref=360,100

RT [min]	Width [min]	Height	Area	Area%
6.366	0.1145	2643.2393	19595.0781	49.6255
7.186	0.1551	1981.4264	19890.8145	50.3745
		Sum	39485.8926	100.0000

Supplementary Figure 163. HPLC spectrum for *rac*-**3hi**

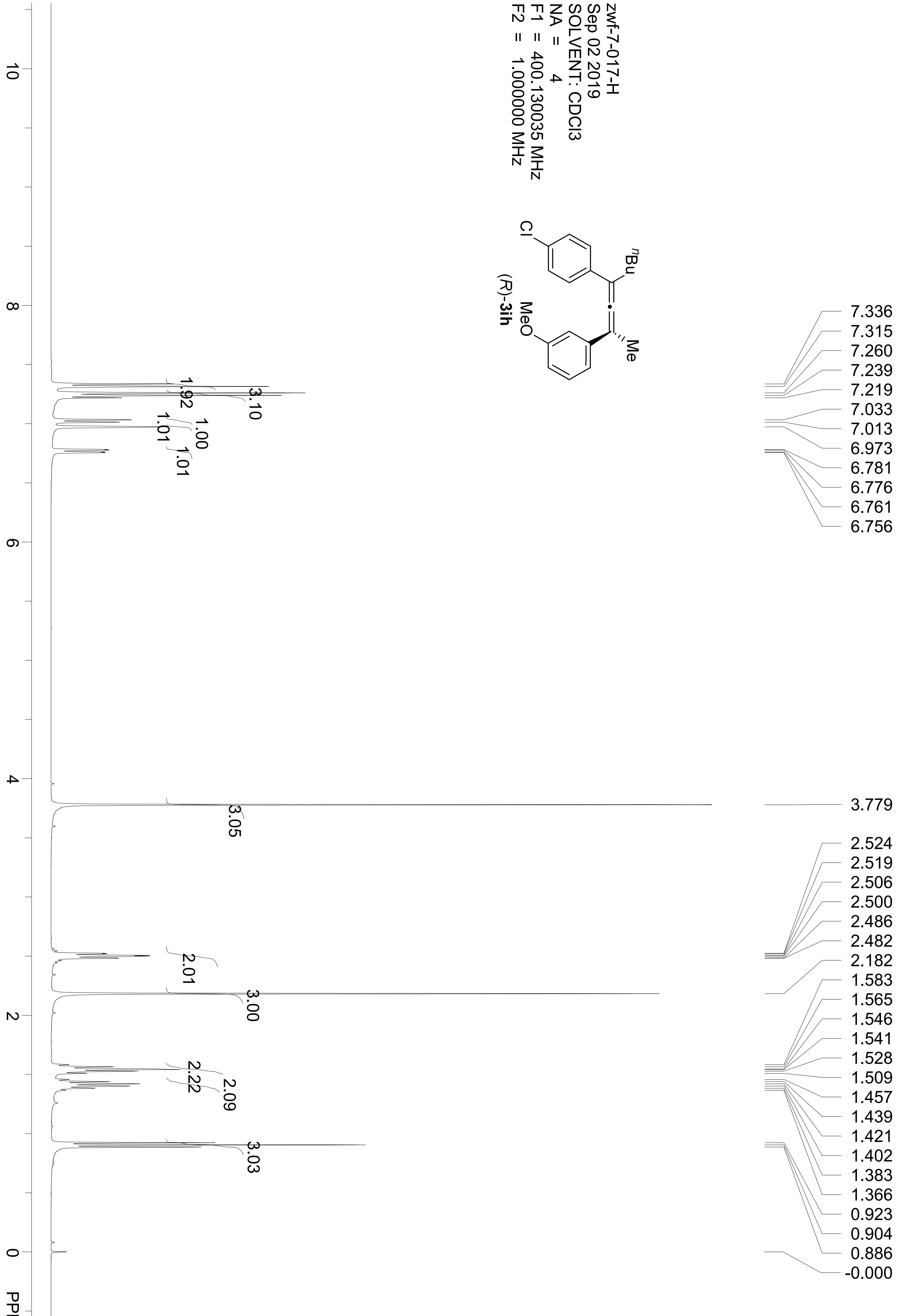

Supplementary Figure 164. ^{1}H NMR (400 MHz, $CDCl_3$) spectrum for (*R*)-**3ih**

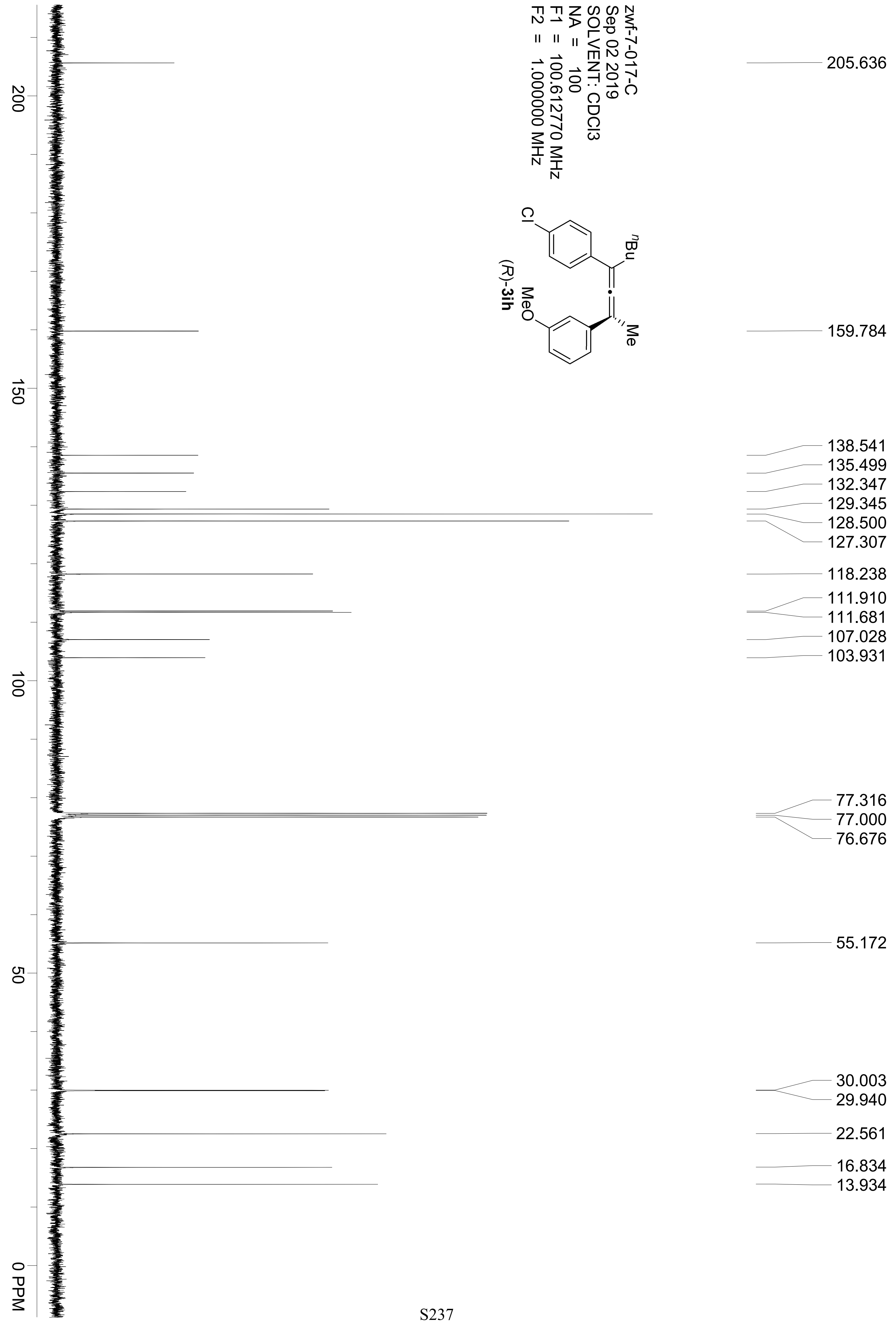

Supplementary Figure 165. ^{13}C NMR (100 MHz, $CDCl_3$) spectrum for (*R*)-**3ih**

Area Percent Report

sample zwf-7-017-OD-H-99.5-0.5-1.0-214

Data file: C:\Users\Public\Documents\ChemStation\1\Data\wgl 2019-09-03 07-35-18\042-P1-C9-zwf-7-017.D

Acquisition Data:

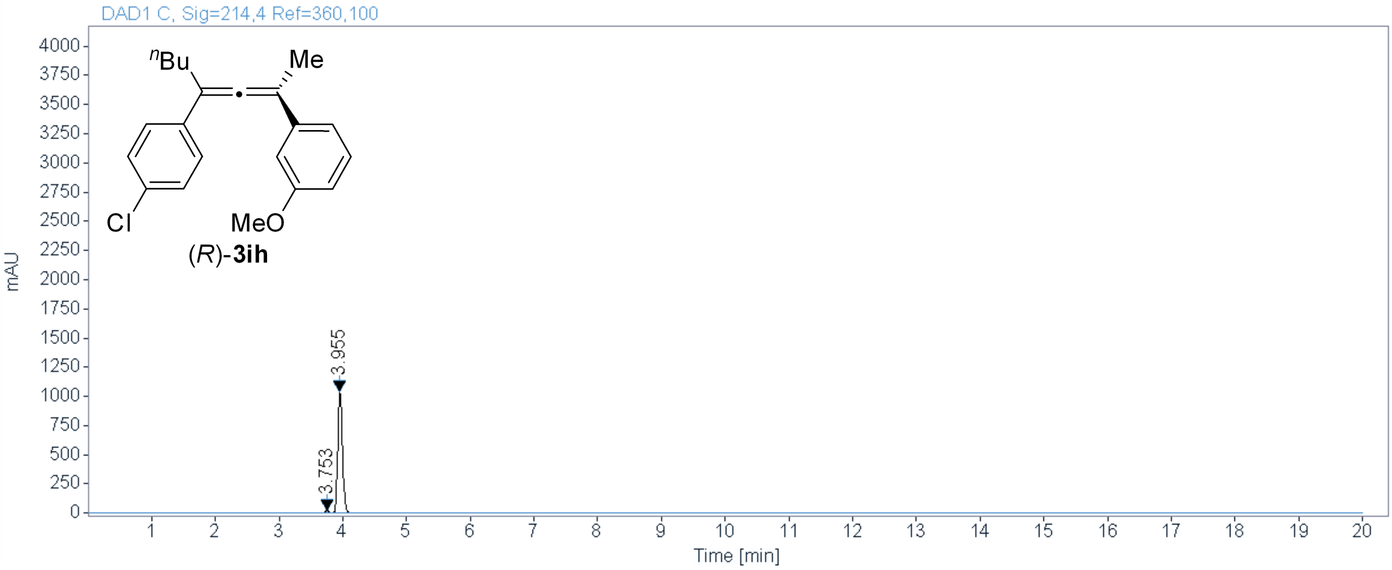

Signal: DAD1 C, Sig=214,4 Ref=360,100

RT [min]	Width [min]	Height	Area	Area%
3.753	0.0738	20.6555	98.8661	1.8301
3.955	0.0773	1043.3285	5303.4927	98.1699
		Sum	5402.3587	100.0000

Supplementary Figure 166. HPLC spectrum for (*R*)-**3ih**

Area Percent Report

sample zwf-7-017-rac-OD-H-99.5-0.5-1.0-214

Data file: C:\Users\Public\Documents\ChemStation\1\Data\wgl 2019-09-03 07-35-18\043-P1-C10-zwf-7-017-rac.D

Acquisition Data:

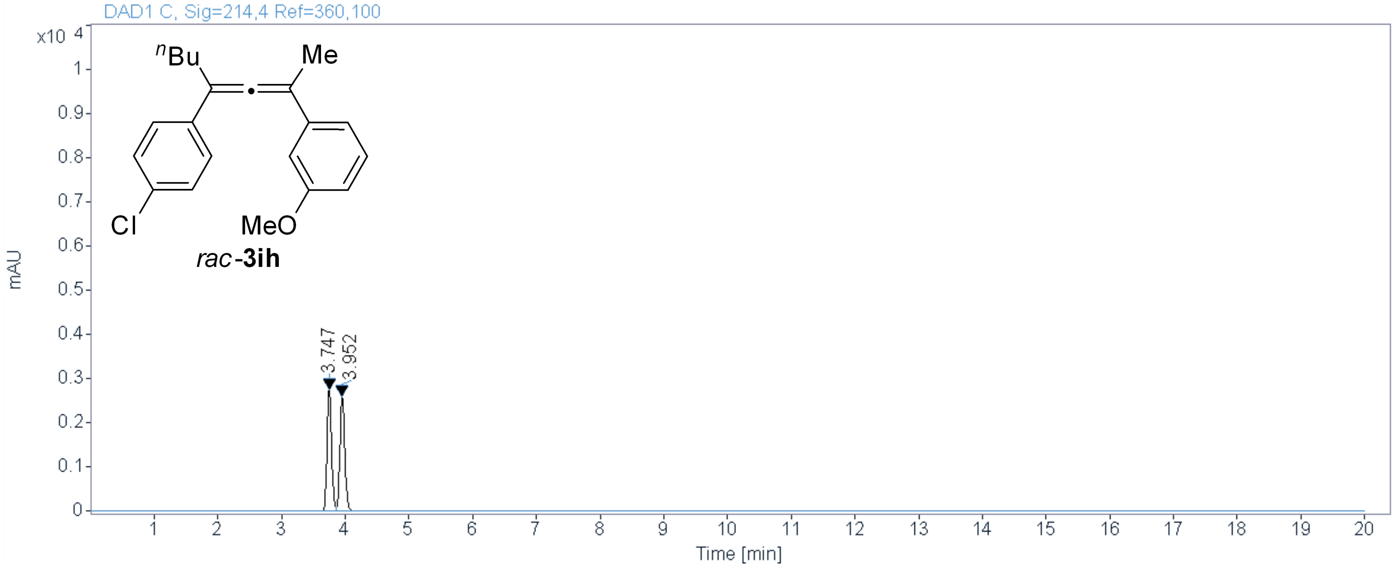

Signal: DAD1 C, Sig=214,4 Ref=360,100

RT [min]	Width [min]	Height	Area	Area%
3.747	0.0786	2762.8435	13877.3604	49.8817
3.952	0.0820	2614.4924	13943.1572	50.1183
		Sum	27820.5176	100.0000

Supplementary Figure 167. HPLC spectrum for *rac*-**3ih**

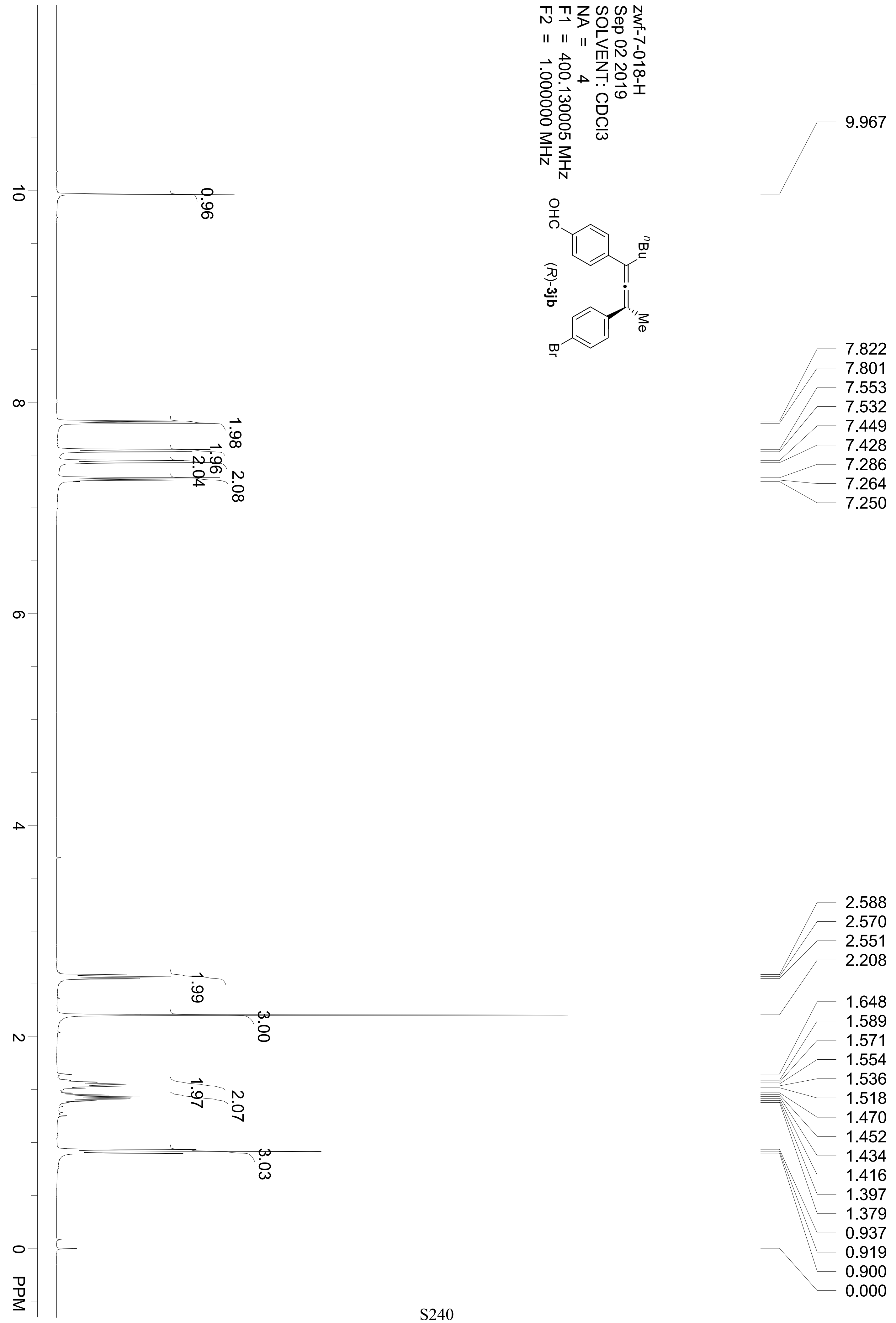

Supplementary Figure 168. ^{1}H NMR (400 MHz, $CDCl_3$) spectrum for (*R*)-**3jb**

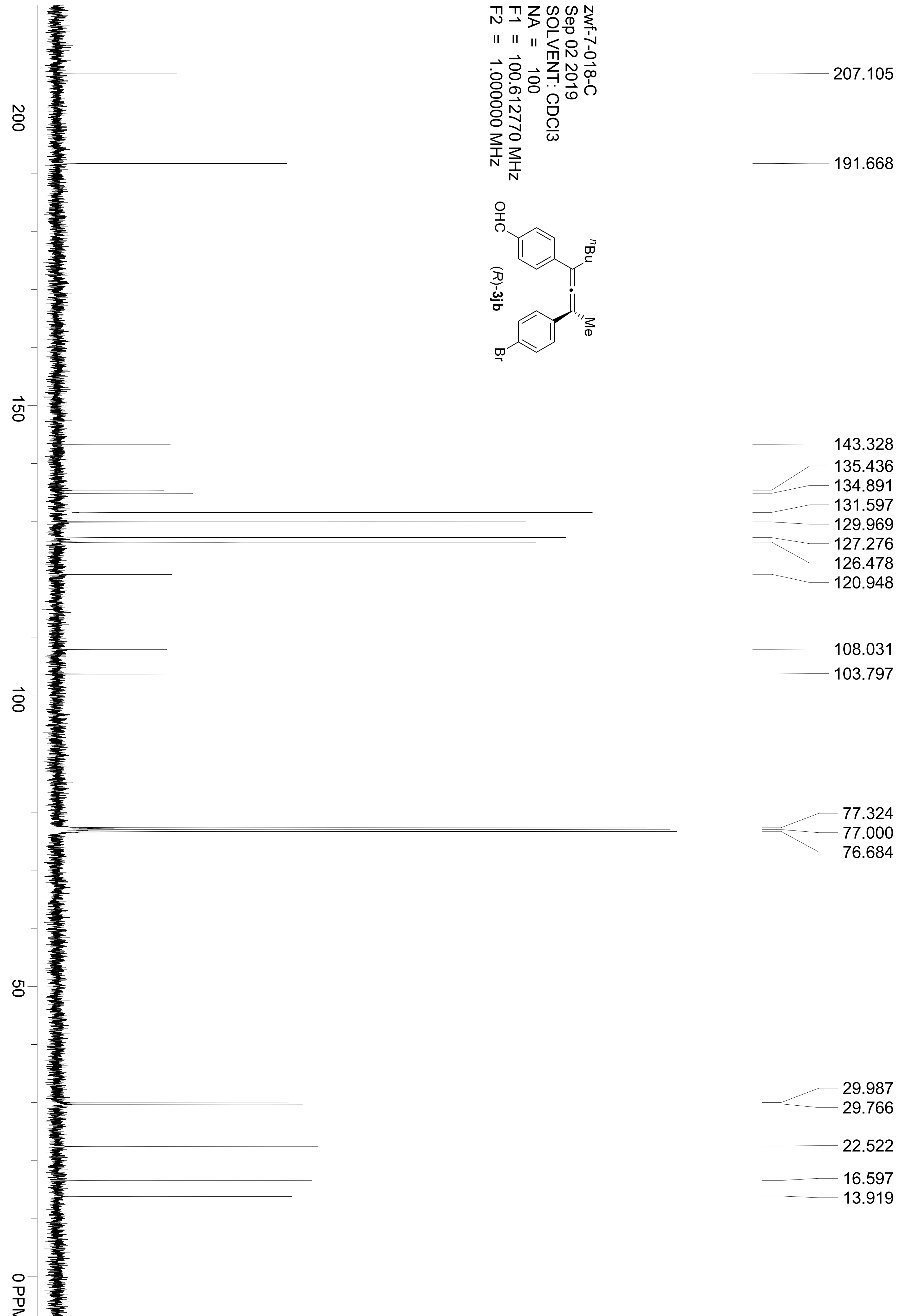

Supplementary Figure 169. ^{13}C NMR (100 MHz, $CDCl_3$) spectrum for (*R*)-**3jb**

Area Percent Report

Agilent Technologies

sample zwf-7-018-OD-H-99.5-0.5-1.0-214

Data file: C:\Users\Public\Documents\ChemStation\1\Data\wgl 2019-09-03 07-35-18\040-P1-C3-zwf-7-018.D

Acquisition Data:

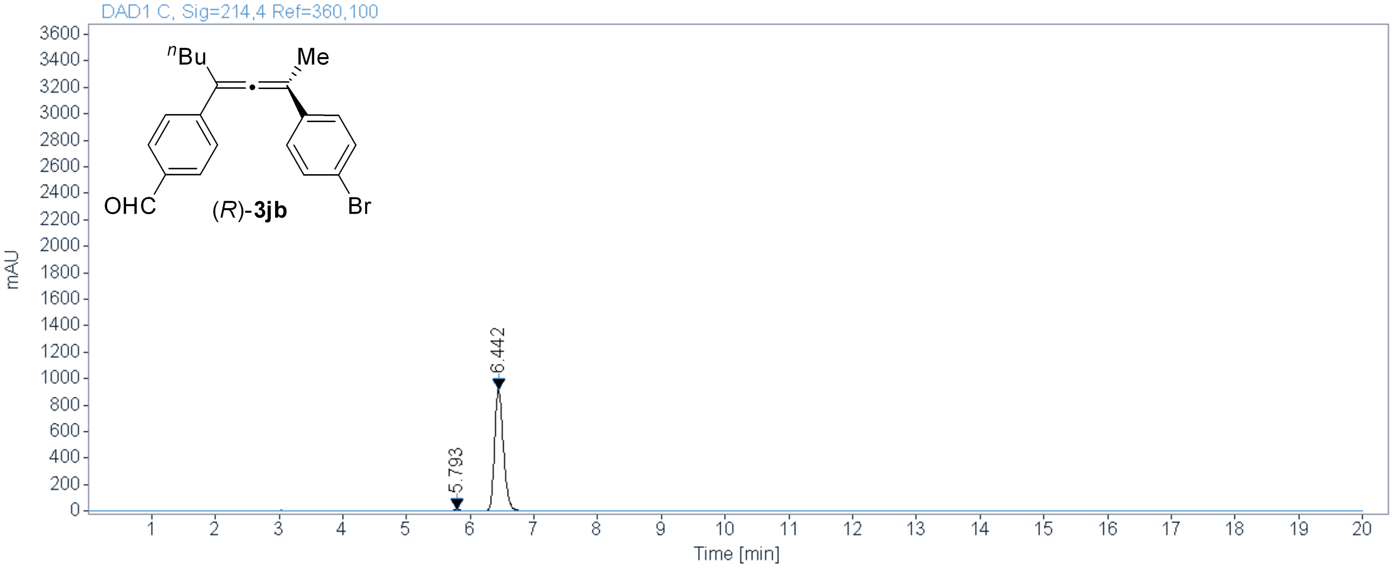

Signal: DAD1 C, Sig=214,4 Ref=360,100

RT [min]	Width [min]	Height	Area	Area%
5.793	0.1268	11.7848	95.7508	1.0564
6.442	0.1625	920.0229	8967.7363	98.9436
		Sum	9063.4872	100.0000

Supplementary Figure 170. HPLC spectrum for (*R*)-**3jb**

Area Percent Report

Agilent Technologies

sample zwf-7-018-rac-OD-H-99.5-0.5-1.0-214

Data file: C:\Users\Public\Documents\ChemStation\1\Data\wgl 2019-09-03 07-35-18\041-P1-C4-zwf-7-018-rac.D

Acquisition Data:

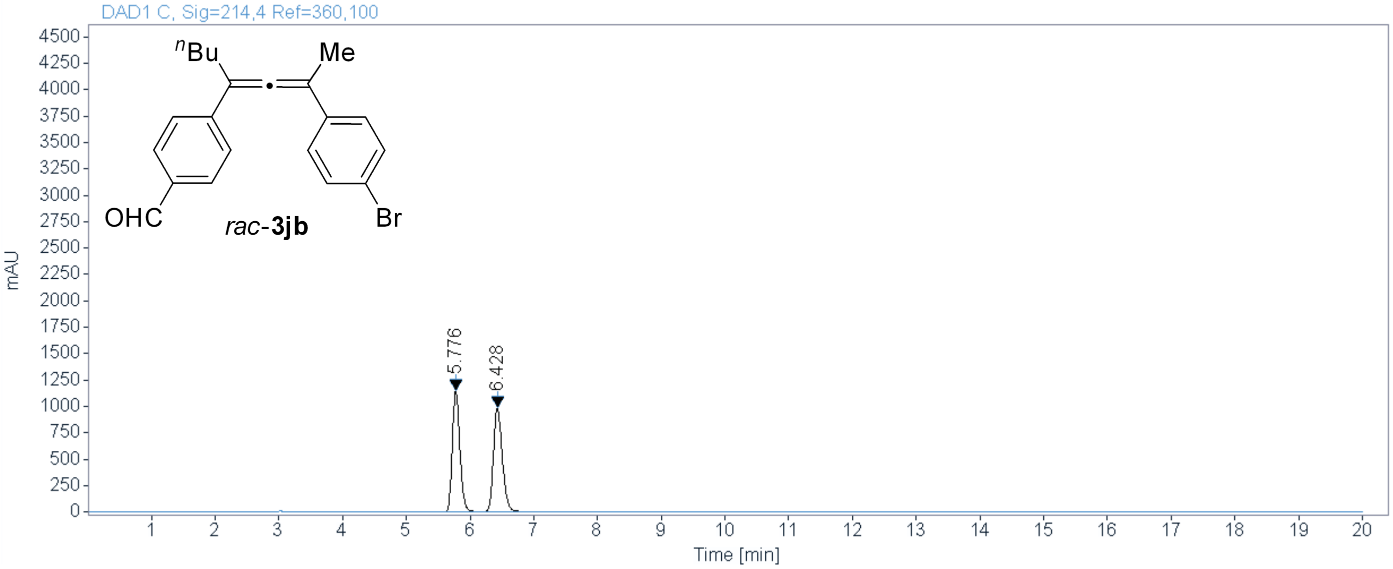

Signal: DAD1 C, Sig=214,4 Ref=360,100

RT [min]	Width [min]	Height	Area	Area%
5.776	0.1290	1156.0583	9611.2158	49.5714
6.428	0.1646	990.1088	9777.4150	50.4286
		Sum	19388.6309	100.0000

Supplementary Figure 171. HPLC spectrum for *rac*-**3jb**

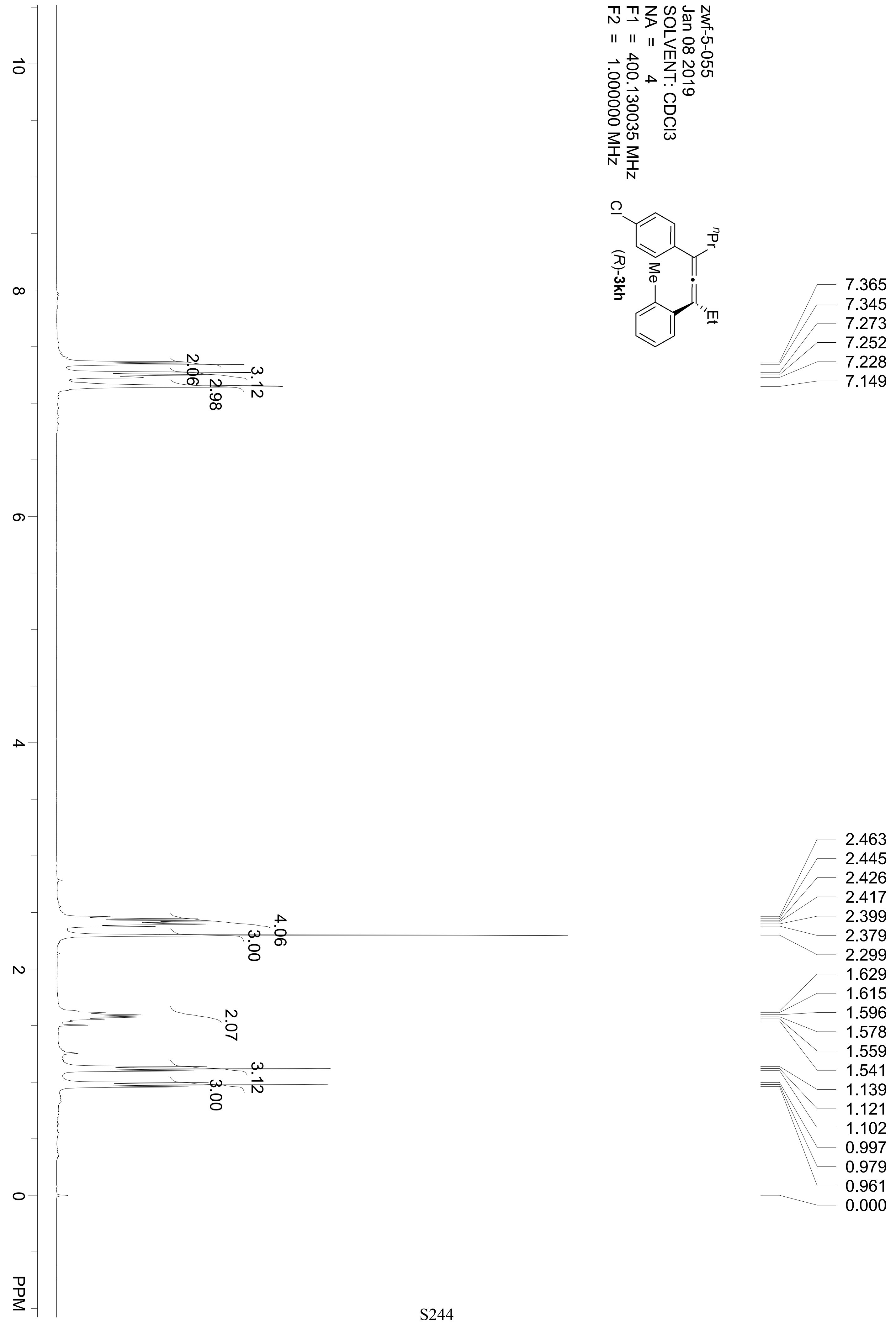

Supplementary Figure 172. ^{1}H NMR (400 MHz, $CDCl_3$) spectrum for (*R*)-**3kh**

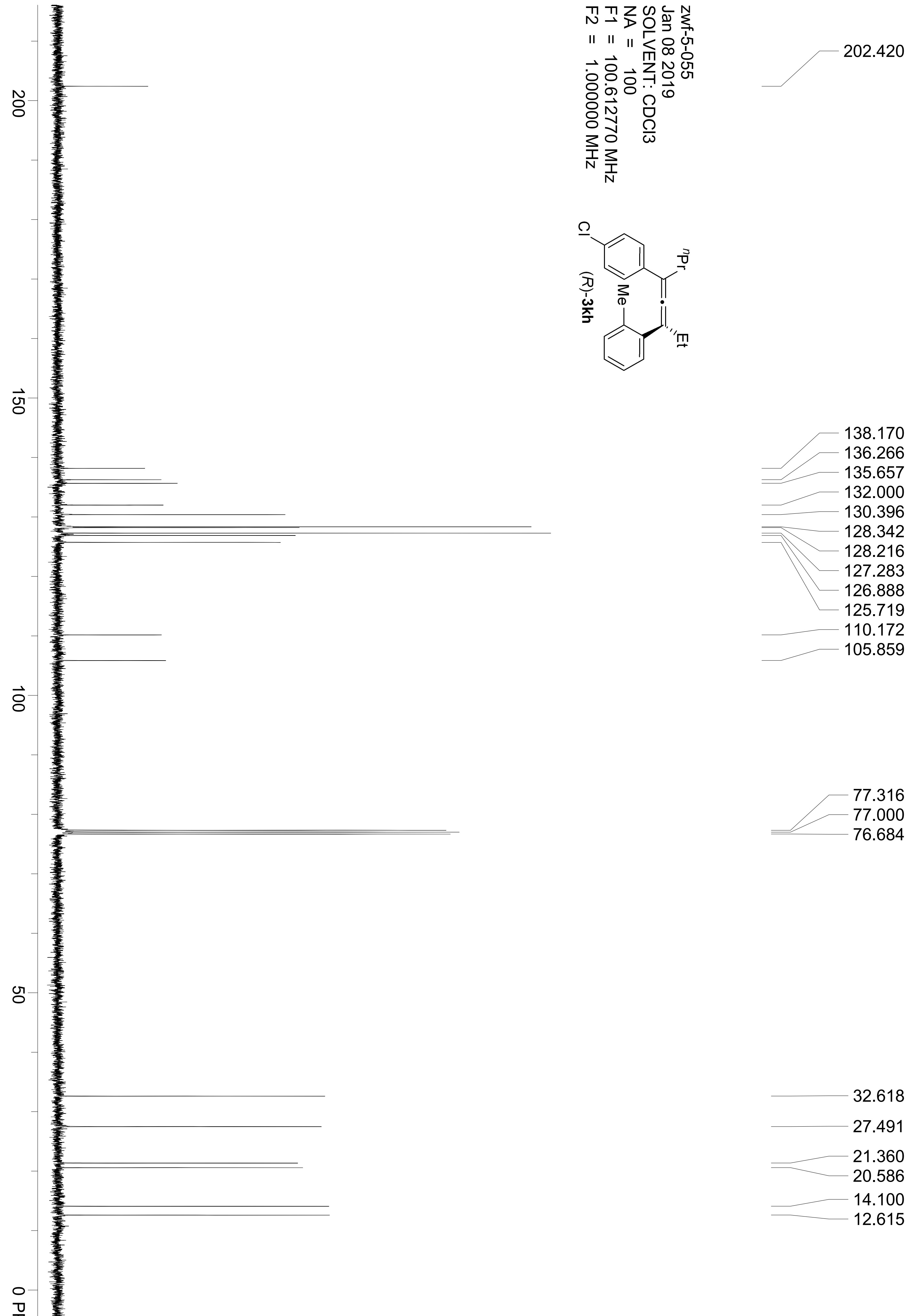

Supplementary Figure 173. ^{13}C NMR (100 MHz, $CDCl_3$) spectrum for (*R*)-**3kh**

Area Percent Report

Agilent Technologies

sample zwf-7-053-AD-H-99.5-0.5-0.5-214

Data file: C:\Users\Public\Documents\ChemStation\1\Data\WHN 2019-09-14 08-20-59\009-P1-C7-zwf-7-053.D

Acquisition Data:

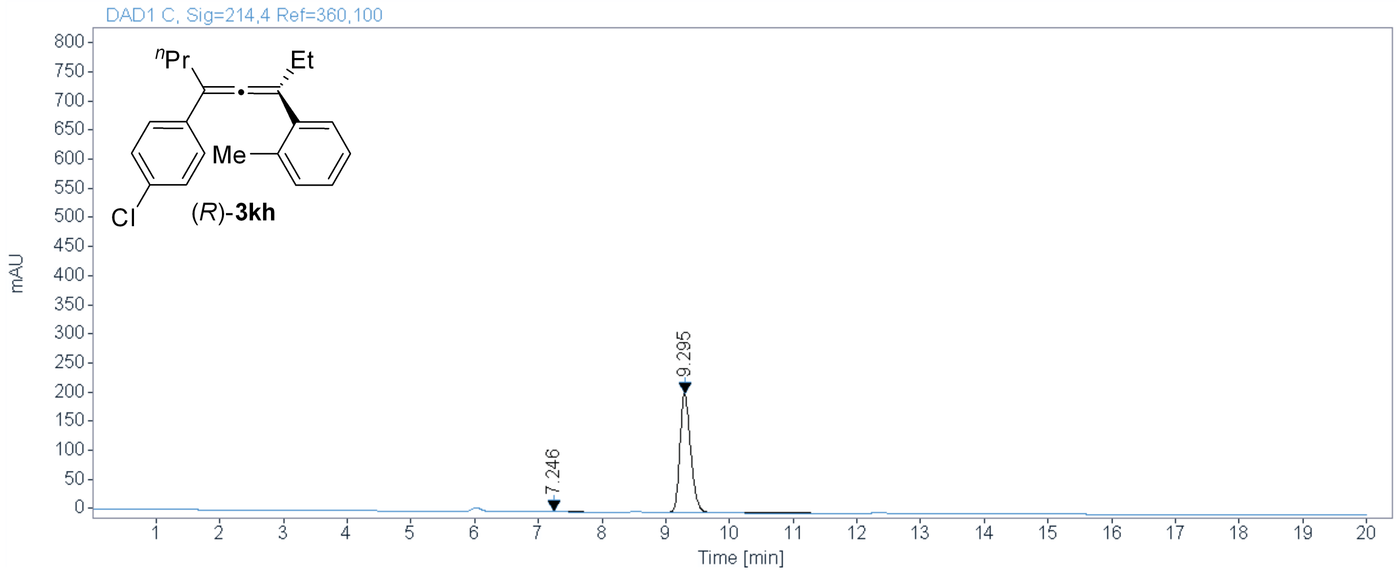

Signal: DAD1 C, Sig=214,4 Ref=360,100

RT [min]	Width [min]	Height	Area	Area%
7.246	0.1477	1.6805	16.3728	0.6757
9.295	0.1791	204.7993	2406.5342	99.3243
		Sum	2422.9069	100.0000

Supplementary Figure 174. HPLC spectrum for (*R*)-**3kh**

Area Percent Report

Agilent Technologies

sample zwf-7-053-rac-AD-H-99.5-0.5-0.5-214

Data file: C:\Users\Public\Documents\ChemStation\1\Data\WHN 2019-09-14 08-20-59\010-P1-C8-zwf-7-053-rac.D

Acquisition Data:

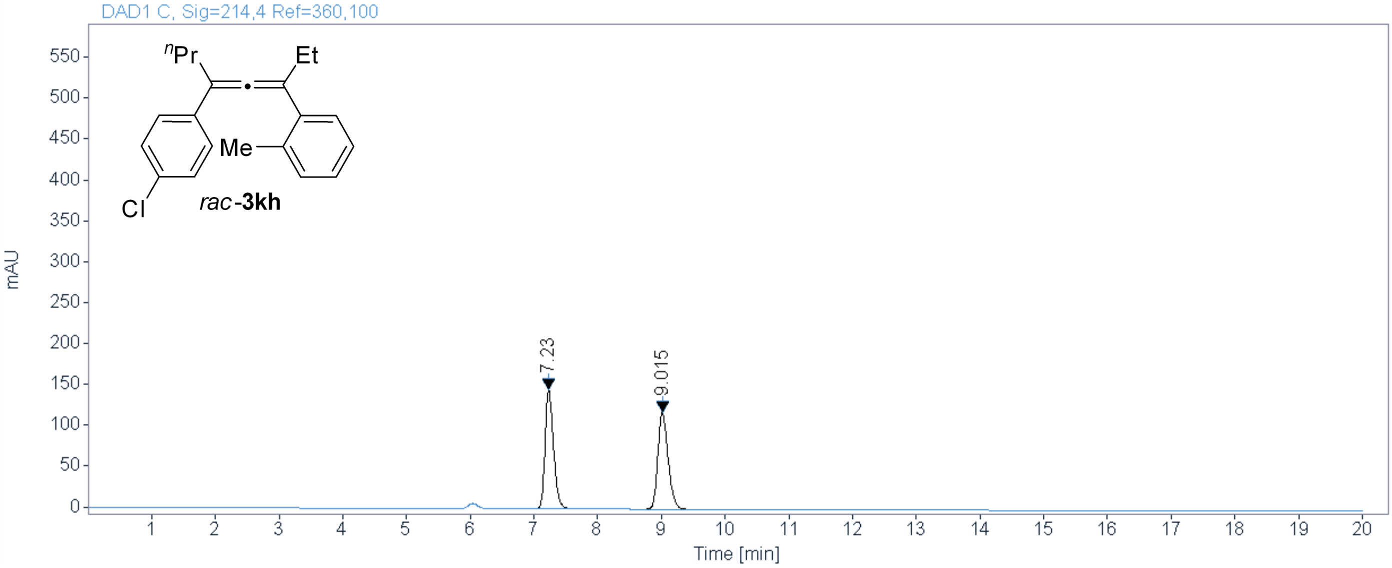

Signal: DAD1 C, Sig=214,4 Ref=360,100

RT [min]	Width [min]	Height	Area	Area%
7.230	0.1386	146.3657	1337.4911	50.0150
9.015	0.1734	118.6678	1336.6895	49.9850
		Sum	2674.1805	100.0000

Supplementary Figure 175. HPLC spectrum for *rac*-**3kh**

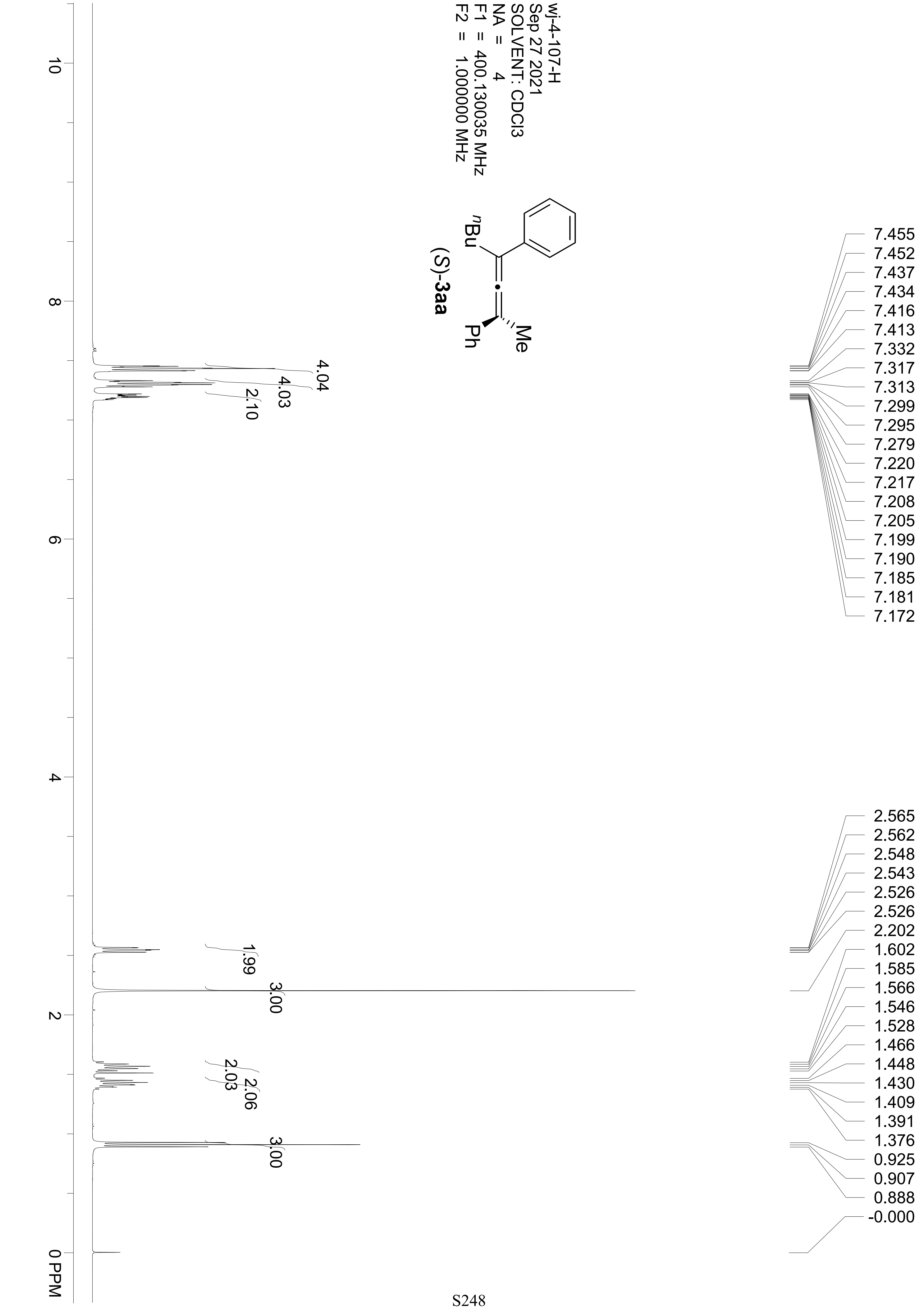

Supplementary Figure 176. ^{1}H NMR (400 MHz, $CDCl_3$) spectrum for (*S*)-**3aa**

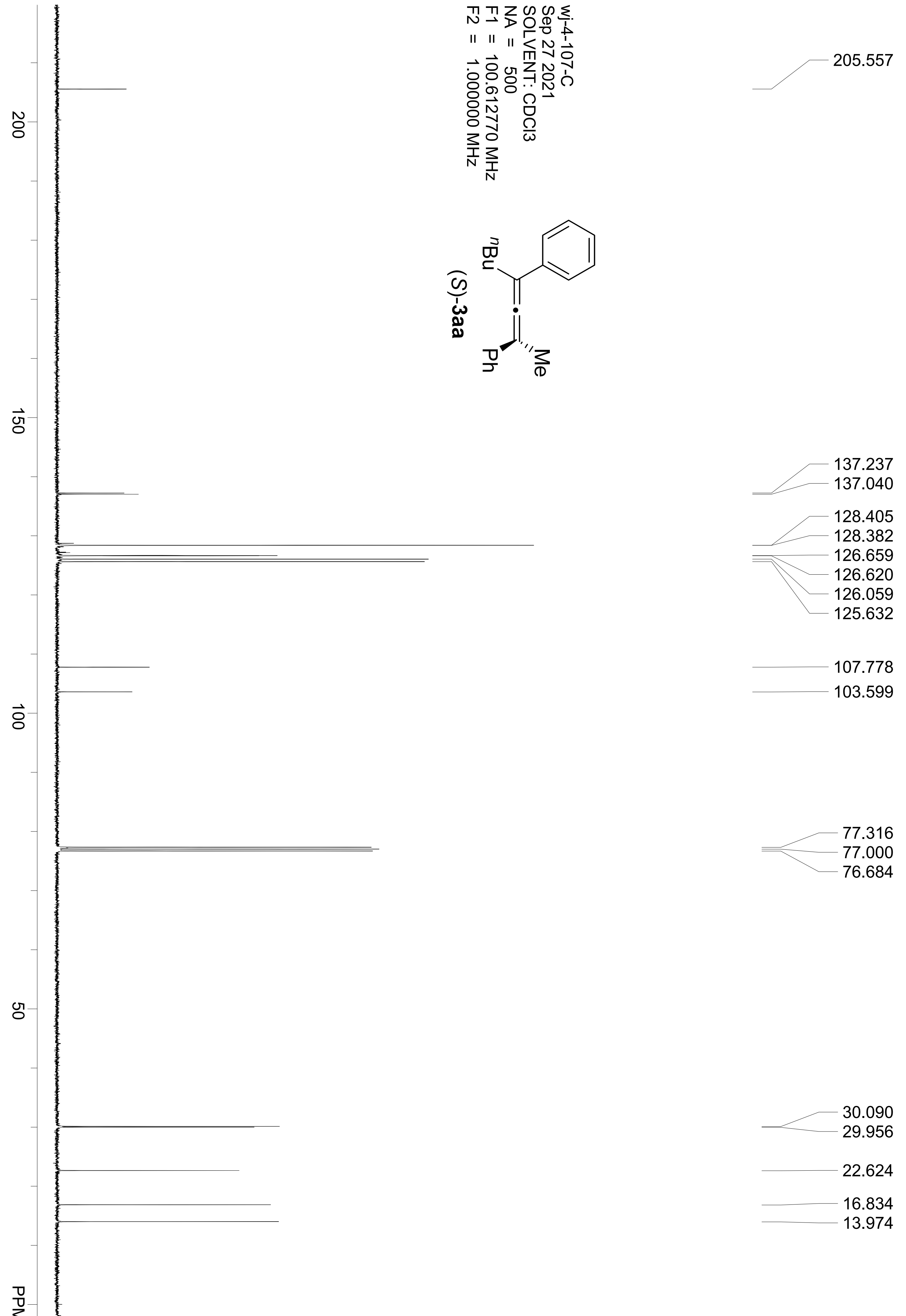

Supplementary Figure 177. ^{13}C NMR (100 MHz, $CDCl_3$) spectrum for (*S*)-**3aa**

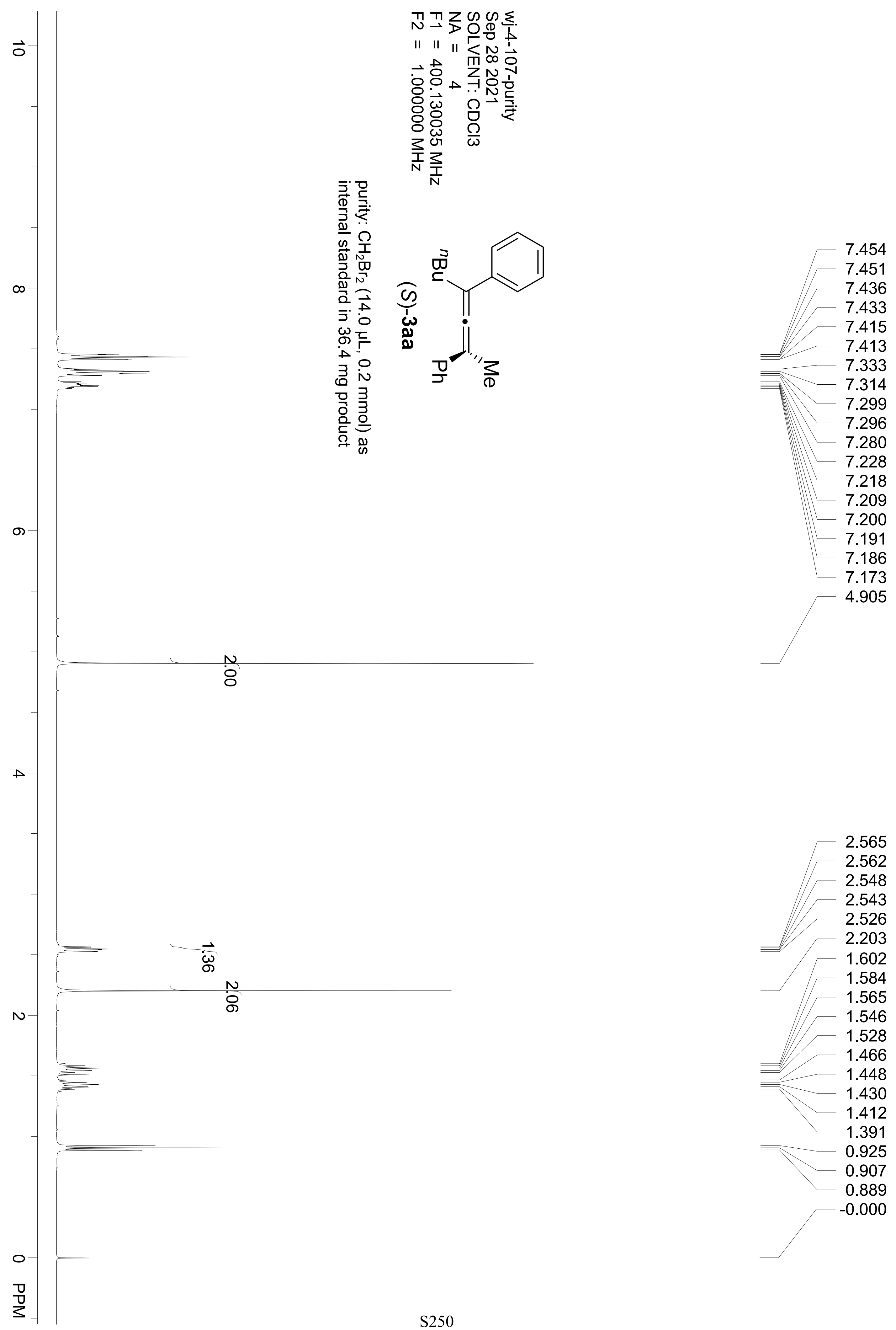

Supplementary Figure 178. Purity spectrum for (*S*)-**3aa**

Area Percent Report

sample wj-4-107-OJ-H-99.5-0.5-0.5-214

Data file: C:\Users\Public\Documents\ChemStation\1\Data\WJ_LC 2021-09-26 22-18-02\002-P2-C1-wj-4-107.D

Acquisition Data:

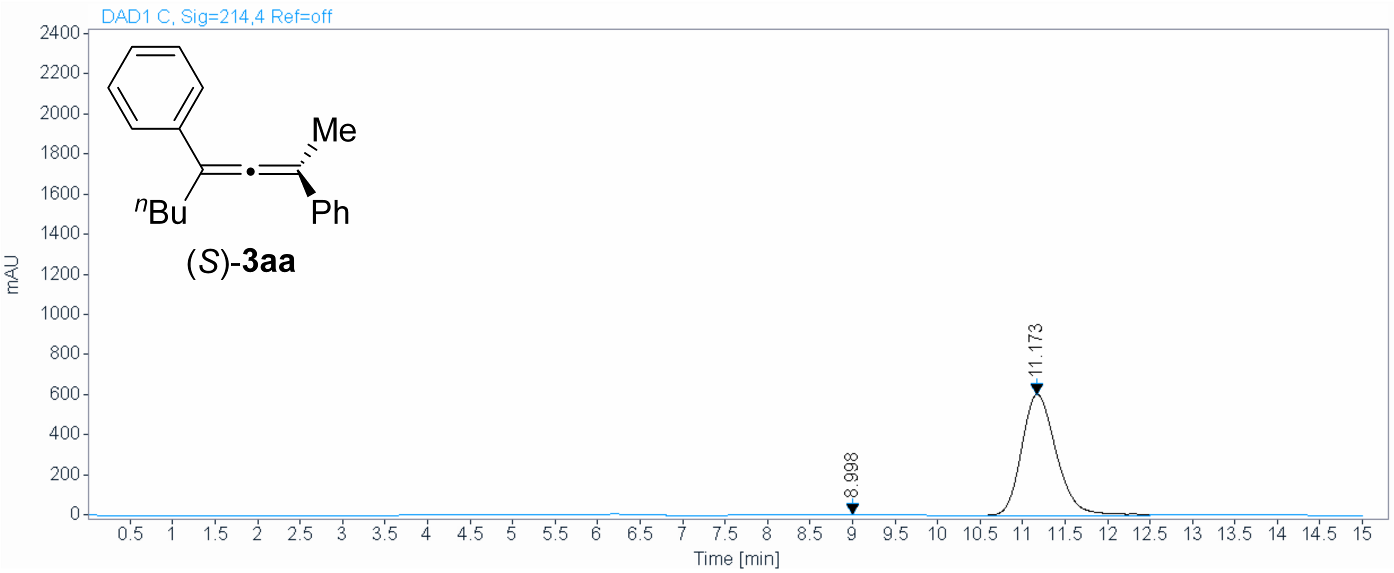

Signal: DAD1 C, Sig=214,4 Ref=off

RT [min]	Width [min]	Height	Area	Area%
8.998	0.3891	3.6079	98.0688	0.5667
11.173	0.4754	603.2621	17208.2188	99.4333
		Sum	17306.2875	100.0000

Supplementary Figure 179. HPLC spectrum for (*S*)-**3aa**

Area Percent Report

Agilent Technologies

sample wj-4-107-rac-OJ-H-99.5-0.5-0.5-214

Data file: C:\Users\Public\Documents\ChemStation\1\Data\WJ_LC 2021-09-26 22-18-02\003-P2-C2-wj-4-0107-rac.D

Acquisition Data:

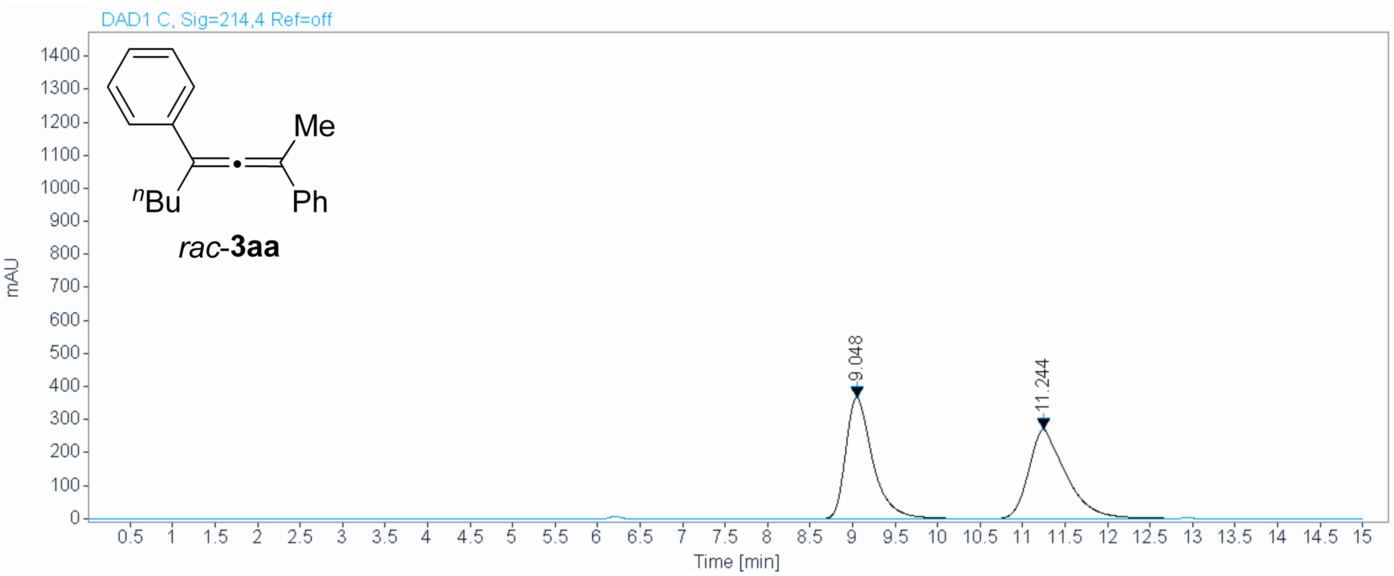

Signal: DAD1 C, Sig=214,4 Ref=off

RT [min]	Width [min]	Height	Area	Area%
9.048	0.3354	367.6170	8173.8438	49.7799
11.244	0.4275	270.6233	8246.1172	50.2201
		Sum	16419.9609	100.0000

Supplementary Figure 180. HPLC spectrum for *rac*-**3aa**

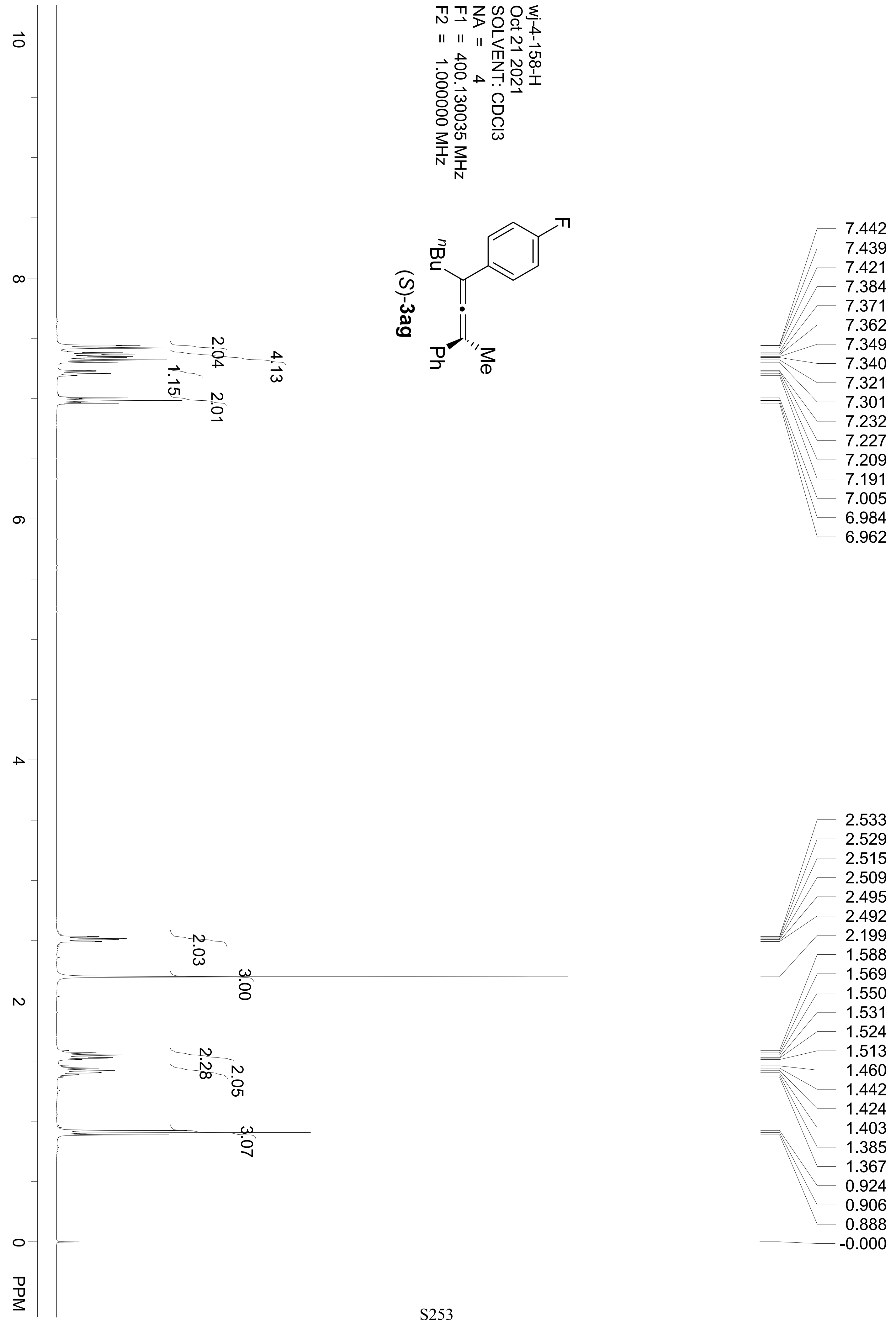

Supplementary Figure 181. ^{1}H NMR (400 MHz, $CDCl_3$) spectrum for (*S*)-**3ag**

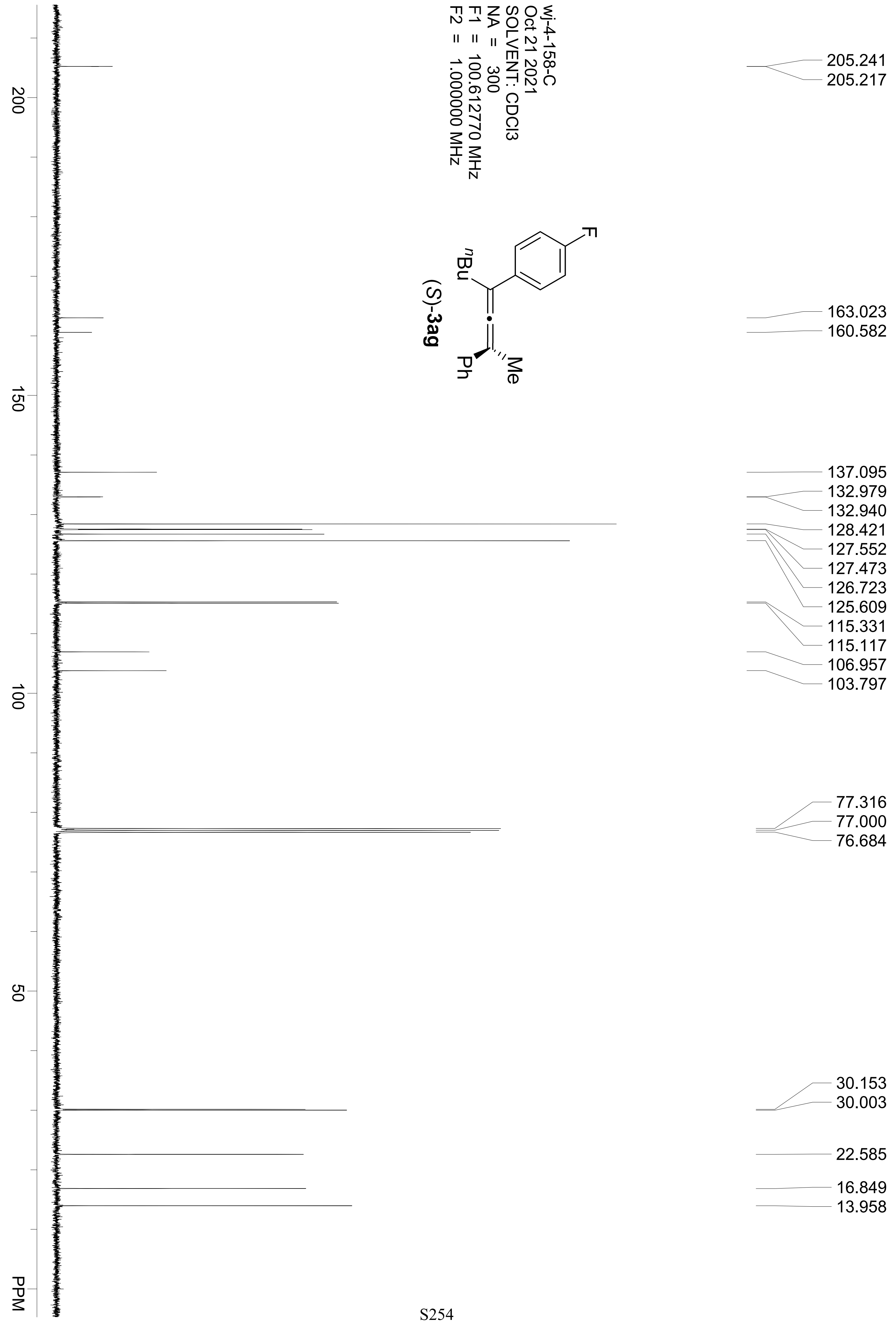

Supplementary Figure 182. ^{13}C NMR (100 MHz, $CDCl_3$) spectrum for (*S*)-**3ag**

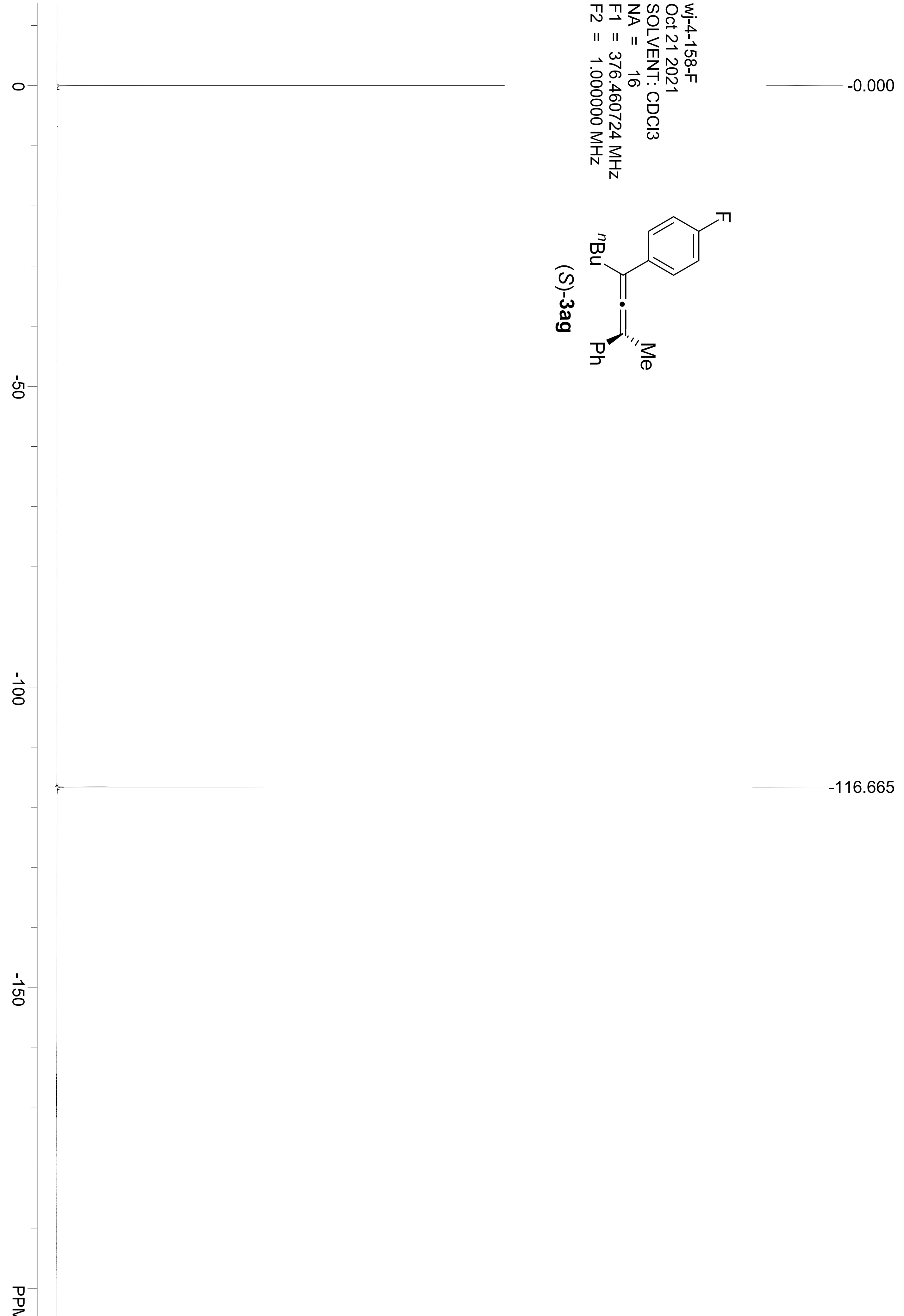

Supplementary Figure 183. ^{19}F NMR (376 MHz, $CDCl_3$) spectrum for (*S*)-**3ag**

Empower™ 3 SOFTWARE

Default Individual Report

SAMPLE INFORMATION

Sample Name:	wj-4-158-chiral	Acquired By:	System
Sample Type:	Unknown	Sample Set Name	
Vial:	1:F,6	Acq. Method Set:	test_1
Injection #:	3	Processing Method	Default
Injection Volume:	0.50 ul	Channel Name:	PDA Ch2 214nm@4.8nm
Run Time:	35.0 Minutes	Proc. Chnl. Descr.:	PDA Ch2 214nm@4.8nm

Date Acquired: 10/29/2021 9:19:30 AM CST
Date Processed: 10/29/2021 1:04:21 PM CST

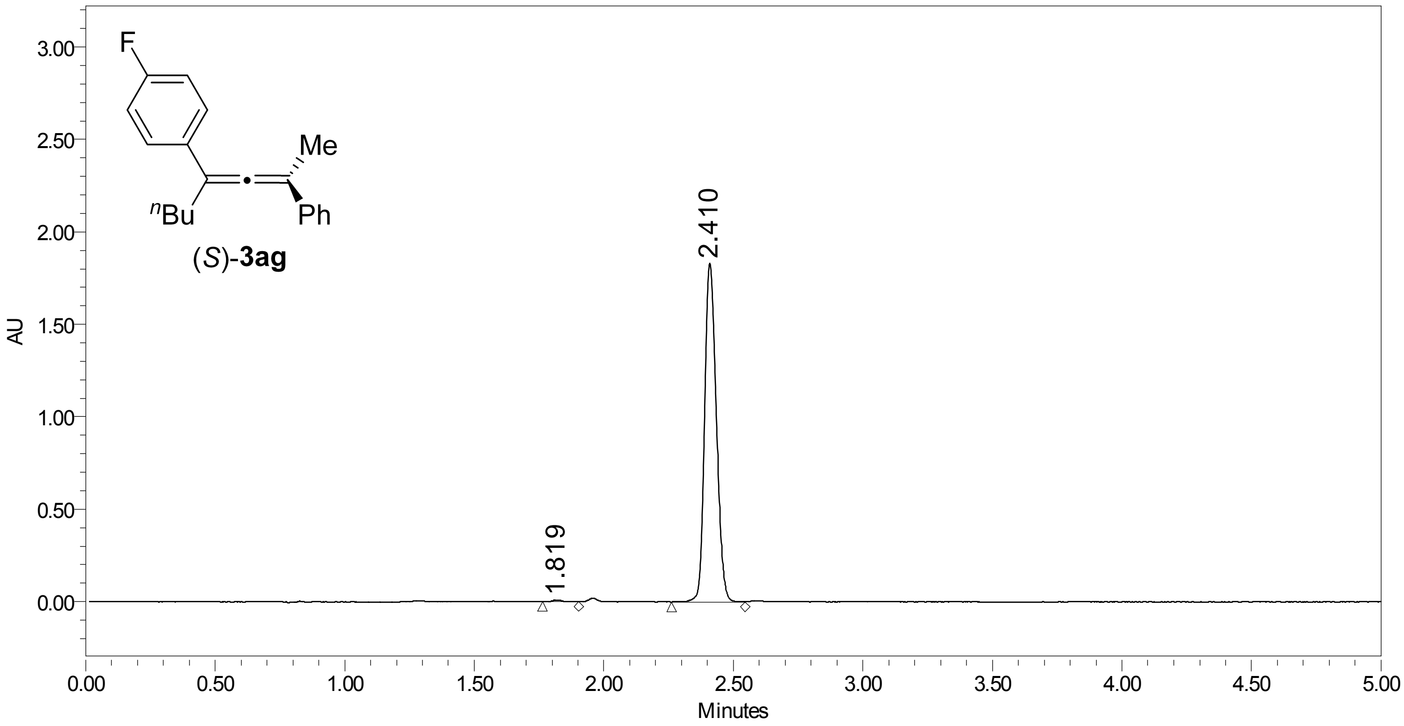

	RT	Height	Width (sec)	Area	% Area
1	1.819	9903	8.450	22474	0.39
2	2.410	1831200	17.100	5805965	99.61

Reported by User: System
Report Method: Default Individual Report
Report Method ID 6848
Page: 1 of 1

Project Name: 2021-2
Date Printed:
10/29/2021
1:24:30 PM PRC

Supplementary Figure 184. SFC spectrum for (*S*)-**3ag**

Default Individual Report

SAMPLE INFORMATION

Sample Name:	wj-4-158-rac	Acquired By:	System
Sample Type:	Unknown	Sample Set Name	
Vial:	1:F,5	Acq. Method Set:	test_1
Injection #:	1	Processing Method	Default
Injection Volume:	1.00 ul	Channel Name:	PDA Ch2 214nm@4.8nm
Run Time:	35.0 Minutes	Proc. Chnl. Descr.:	PDA Ch2 214nm@4.8nm
Date Acquired:	10/29/2021 8:52:43 AM CST		
Date Processed:	10/29/2021 1:04:50 PM CST		

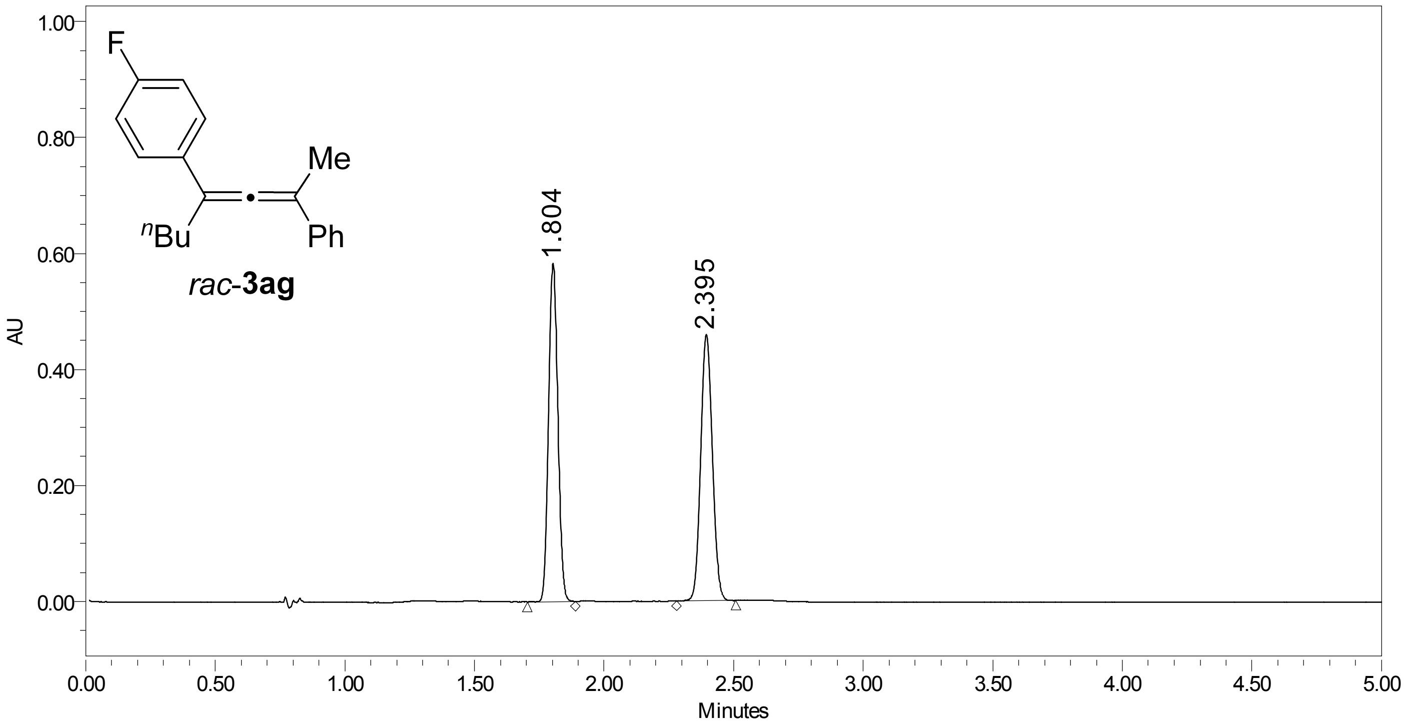

	RT	Height	Width (sec)	Area	% Area
1	1.804	583375	11.150	1448968	49.94
2	2.395	459340	13.750	1452528	50.06

Reported by User: System
Report Method: Default Individual Report
Report Method ID 6848
Page: 1 of 1

Project Name: 2021-2
Date Printed:
10/29/2021
1:24:17 PM PRC

Supplementary Figure 185. SFC spectrum for *rac*-**3ag**

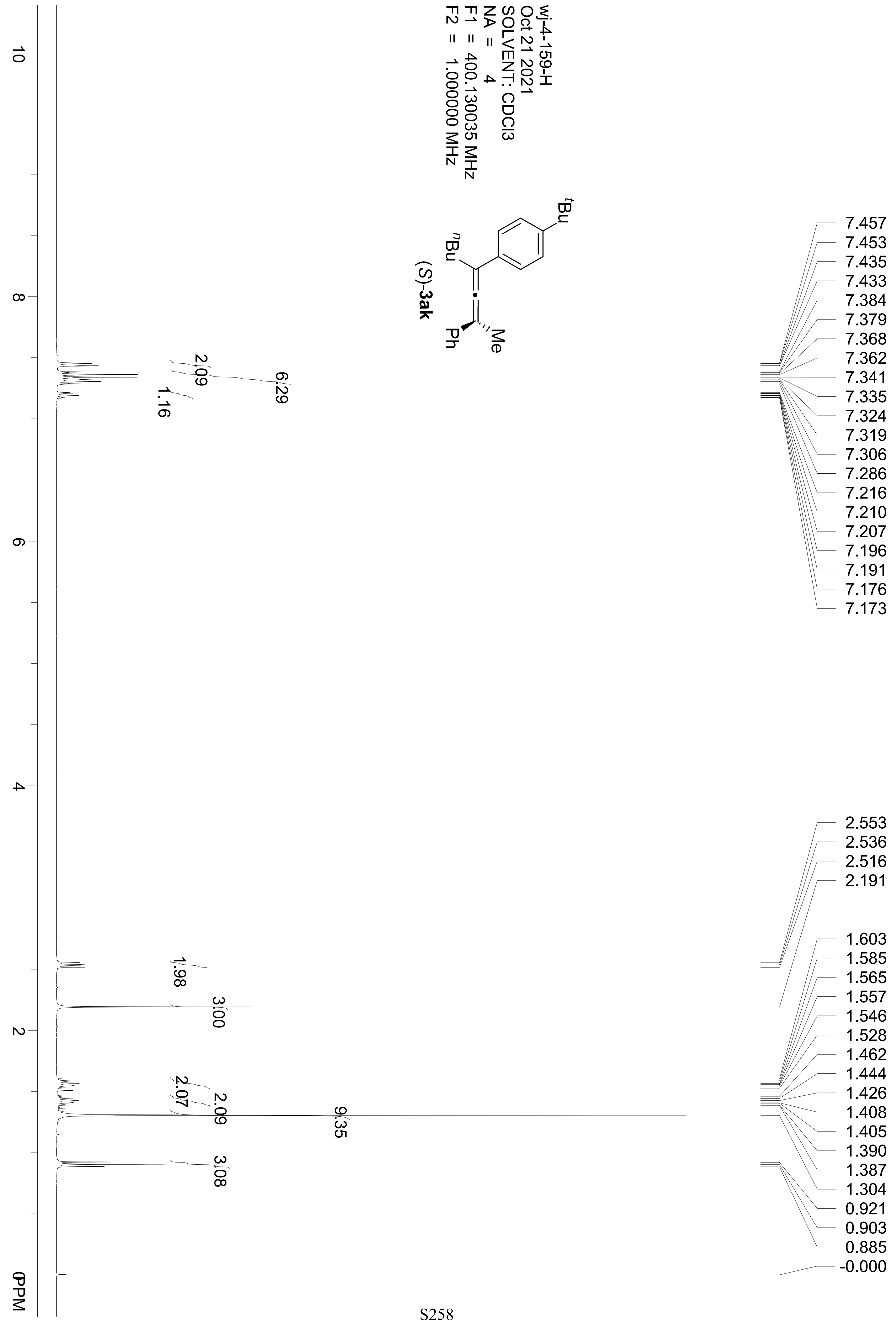

Supplementary Figure 186. ^{1}H NMR (400 MHz, $CDCl_3$) spectrum for (*S*)-**3ak**

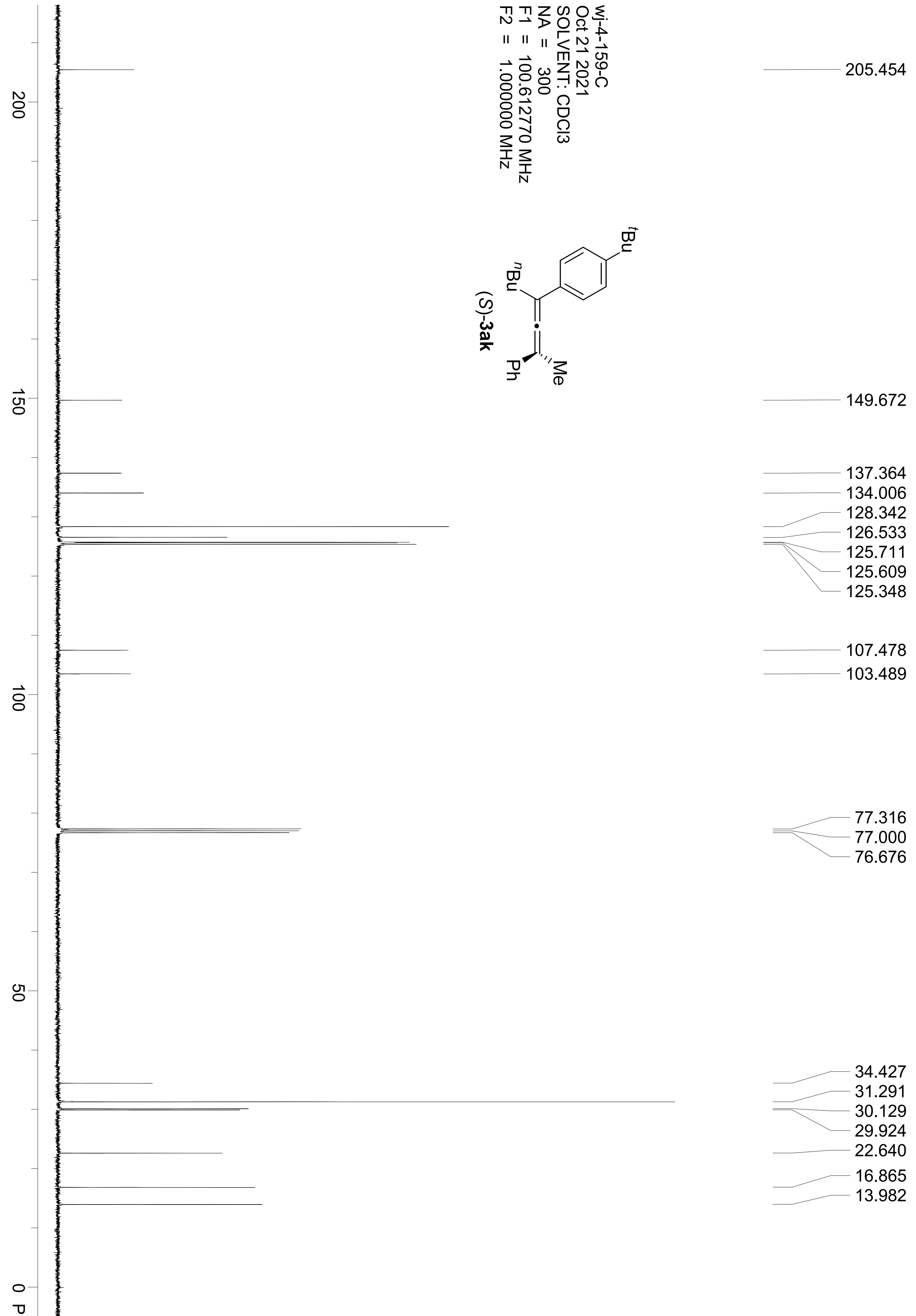

Supplementary Figure 187. ^{13}C NMR (100 MHz, $CDCl_3$) spectrum for (*S*)-**3ak**

Default Individual Report

SAMPLE INFORMATION

Sample Name:	wj-4-159-chiral	Acquired By:	System
Sample Type:	Unknown	Sample Set Name	
Vial:	1:F,8	Acq. Method Set:	test_1
Injection #:	1	Processing Method	Default
Injection Volume:	1.00 ul	Channel Name:	PDA Ch3 254nm@4.8nm
Run Time:	35.0 Minutes	Proc. Chnl. Descr.:	PDA Ch3 254nm@4.8nm
Date Acquired:	10/29/2021 1:06:01 PM CST		
Date Processed:	10/29/2021 1:23:15 PM CST		

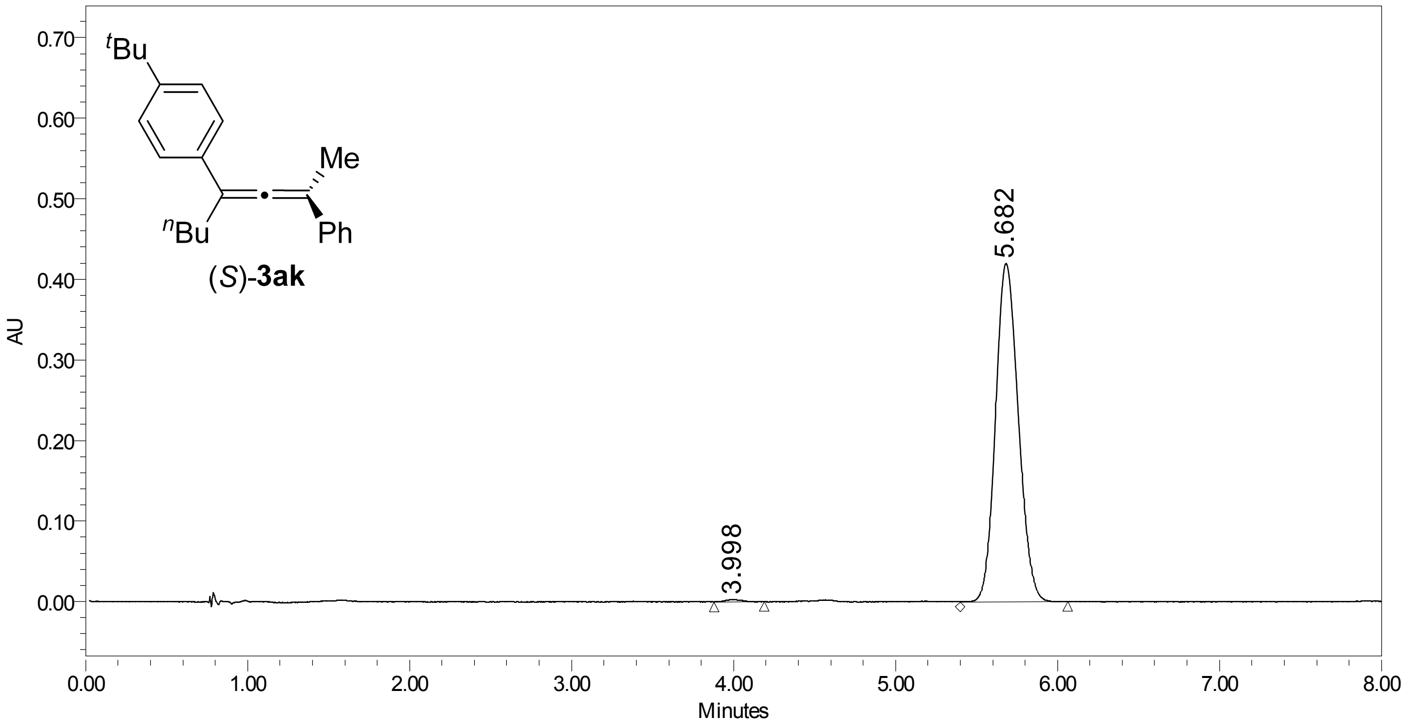

	RT	Height	Width (sec)	Area	% Area
1	3.998	3020	18.600	19202	0.46
2	5.682	420111	39.700	4137257	99.54

Reported by User: System
Report Method: Default Individual Report
Report Method IL 6850
Page: 1 of 1

Project Name: 2021-2
Date Printed:
10/29/2021
1:25:25 PM PRC

Supplementary Figure 188. SFC spectrum for (*S*)-**3ak**

Default Individual Report

SAMPLE INFORMATION

Sample Name:	wj-4-159-rac	Acquired By:	System
Sample Type:	Unknown	Sample Set Name	
Vial:	1:F,7	Acq. Method Set:	test_1
Injection #:	2	Processing Method	Default
Injection Volume:	1.50 ul	Channel Name:	PDA Ch3 254nm@4.8nm
Run Time:	35.0 Minutes	Proc. Chnl. Descr.:	PDA Ch3 254nm@4.8nm
Date Acquired:	10/29/2021 12:56:45 PM CST		
Date Processed:	10/29/2021 1:22:52 PM CST		

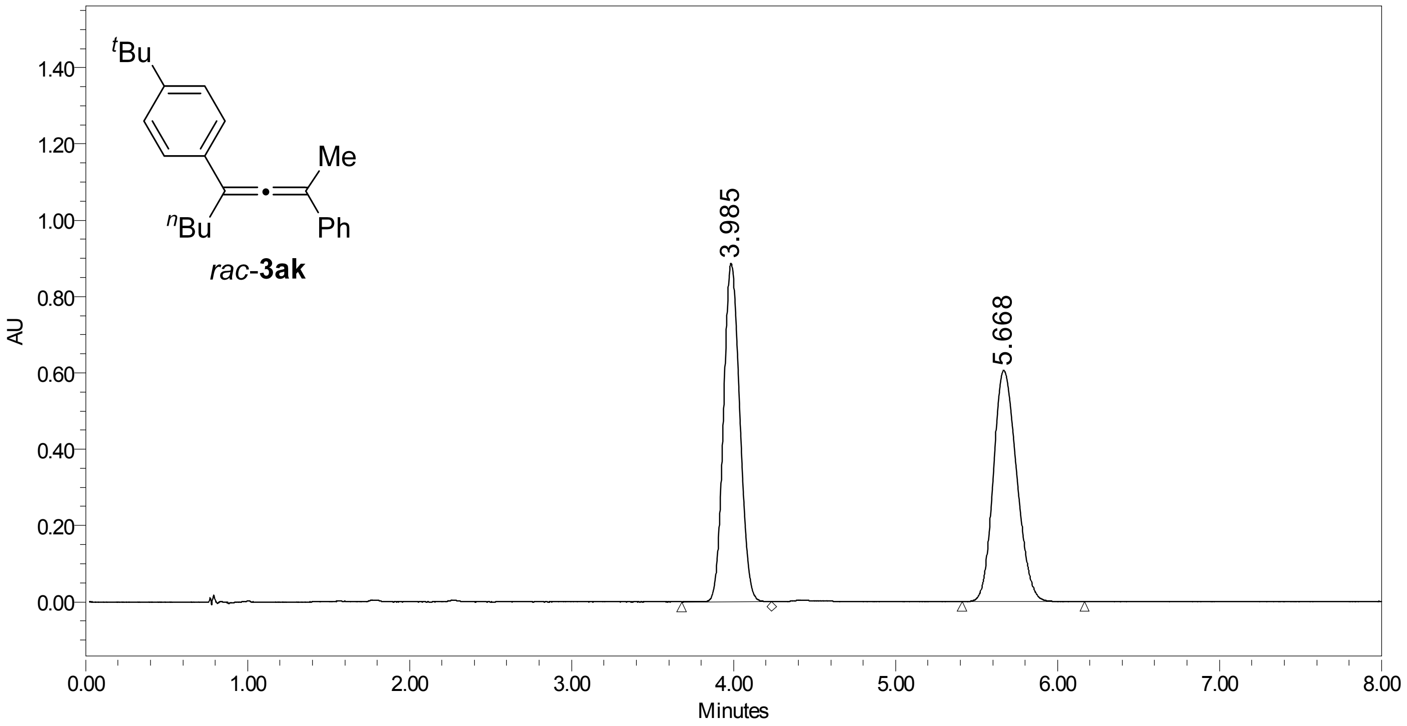

	RT	Height	Width (sec)	Area	% Area
1	3.985	886678	33.250	6170236	50.22
2	5.668	605910	45.300	6117031	49.78

Supplementary Figure 189. SFC spectrum for *rac*-**3ak**

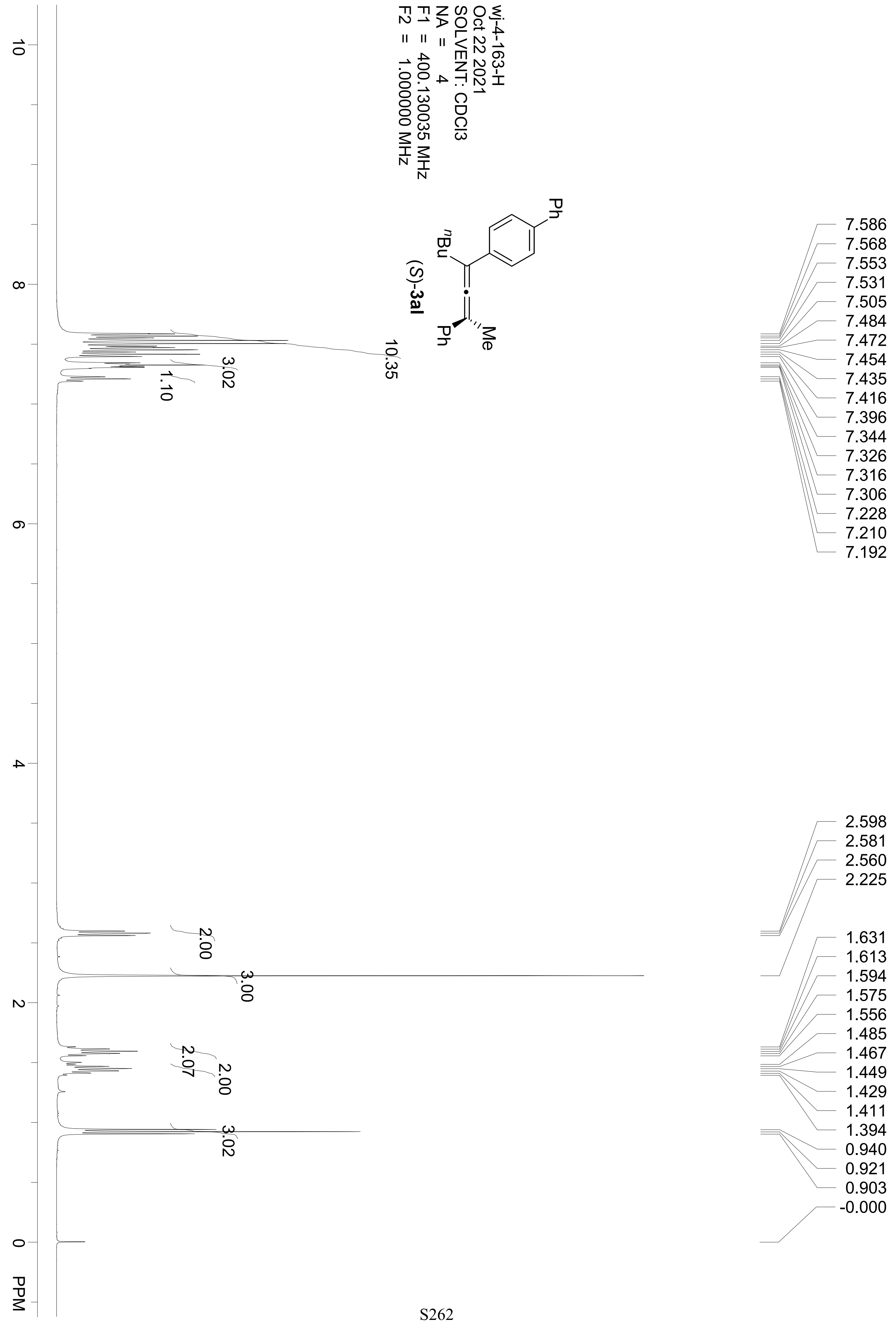

Supplementary Figure 190. ^{1}H NMR (400 MHz, $CDCl_3$) spectrum for (*S*)-**3al**

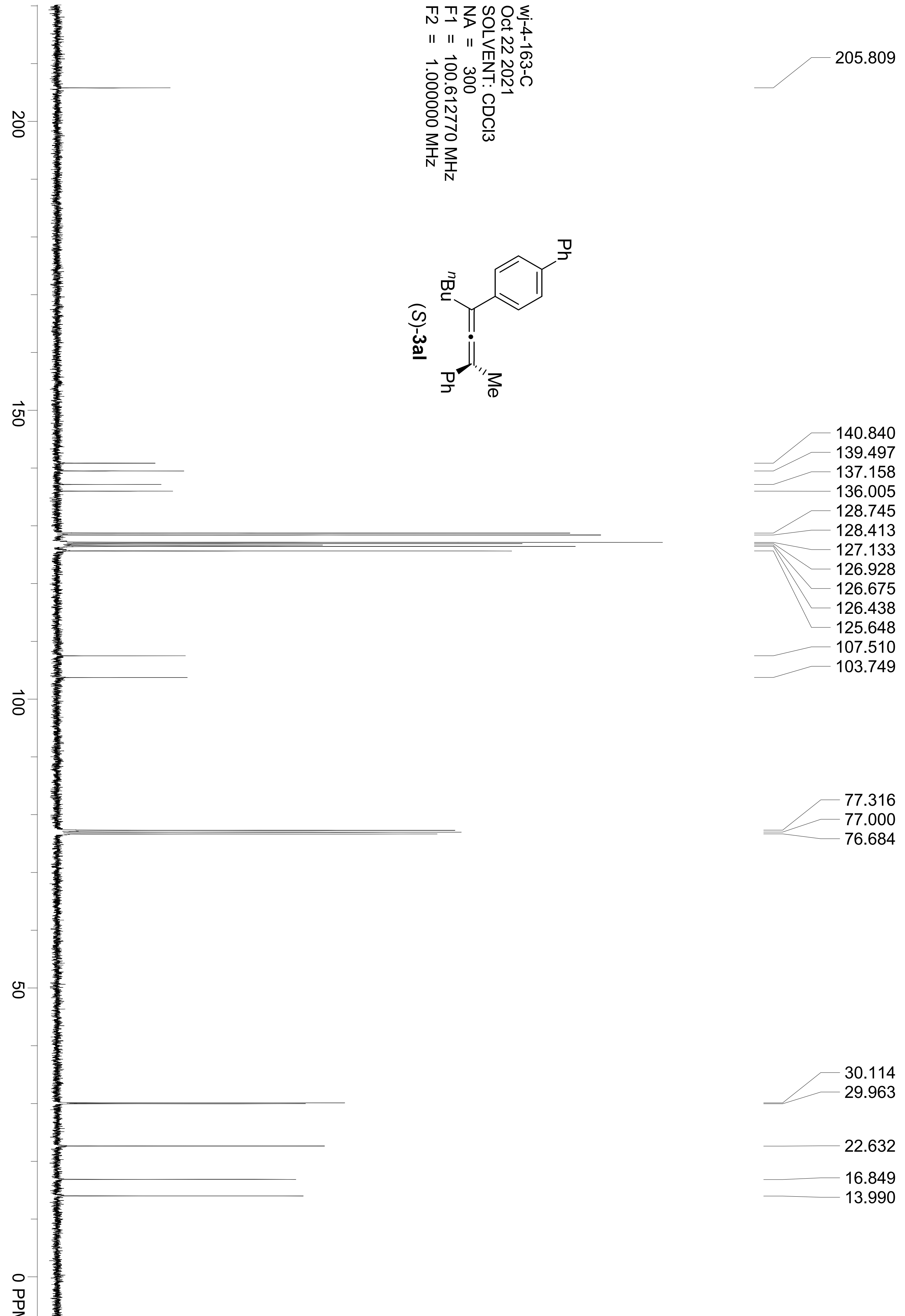

Supplementary Figure 191. ^{13}C NMR (100 MHz, $CDCl_3$) spectrum for (*S*)-**3al**

Area Percent Report

Agilent Technologies

sample wj-4-163-OD-H-99.5-0.5-1.0-214

Data file: C:\Users\Public\Documents\ChemStation\1\Data\WJ_LC 2021-10-22 08-38-56\040-P2-C1-wj-4-163.D

Acquisition Data:

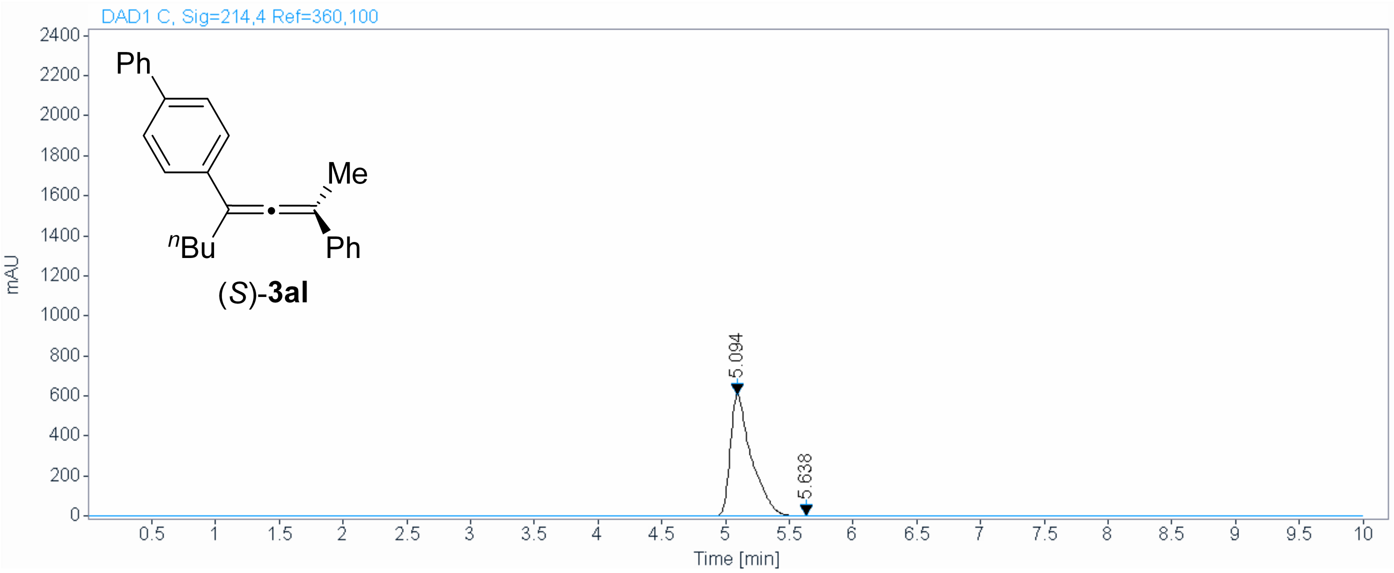

Signal: DAD1 C, Sig=214,4 Ref=360,100

RT [min]	Width [min]	Height	Area	Area%
5.094	0.1674	608.4805	7070.0327	99.5596
5.638	0.1823	2.3951	31.2755	0.4404
		Sum	7101.3082	100.0000

Supplementary Figure 192. HPLC spectrum for (*S*)-**3al**

Area Percent Report

Agilent Technologies

sample wj-4-163-rac-OD-H-99.5-0.5-1.0-214

Data file: C:\Users\Public\Documents\ChemStation\1\Data\WJ_LC 2021-10-22 08-38-56\041-P2-C2-wj-4-163-rac.D

Acquisition Data:

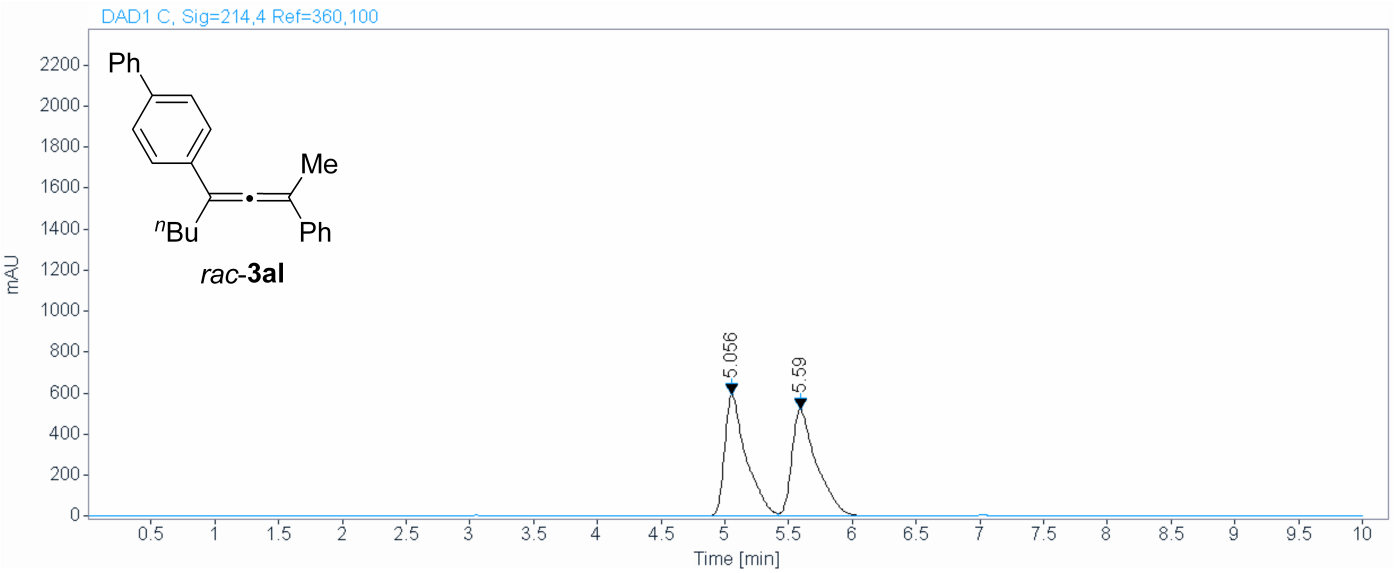

Signal: DAD1 C, Sig=214,4 Ref=360,100

RT [min]	Width [min]	Height	Area	Area%
5.056	0.1961	594.5739	6997.2534	49.8045
5.590	0.1957	521.8040	7052.1729	50.1955
		Sum	14049.4263	100.0000

Supplementary Figure 193. HPLC spectrum for *rac*-**3al**

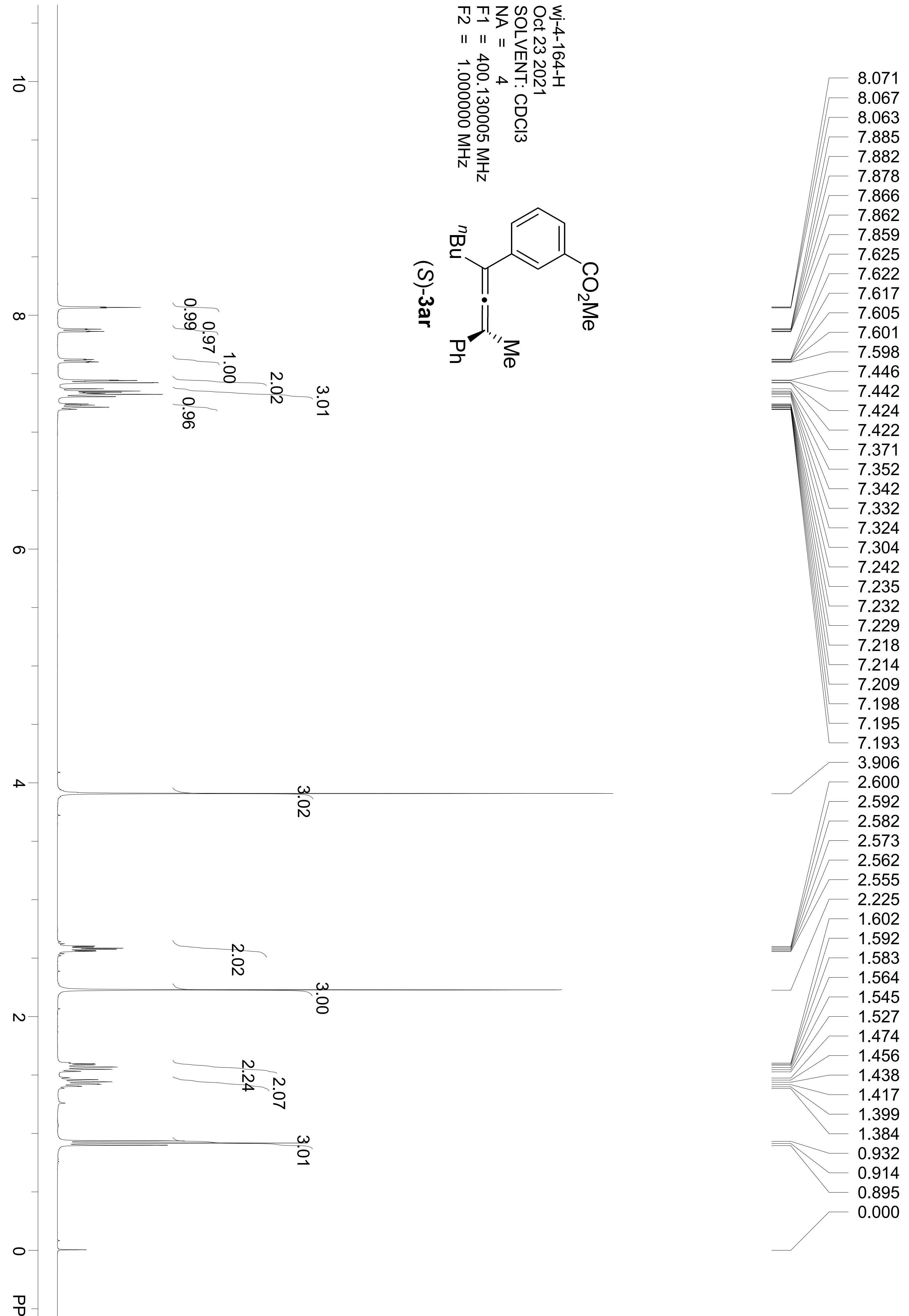

Supplementary Figure 194. ^{1}H NMR (400 MHz, $CDCl_3$) spectrum for (*S*)-**3ar**

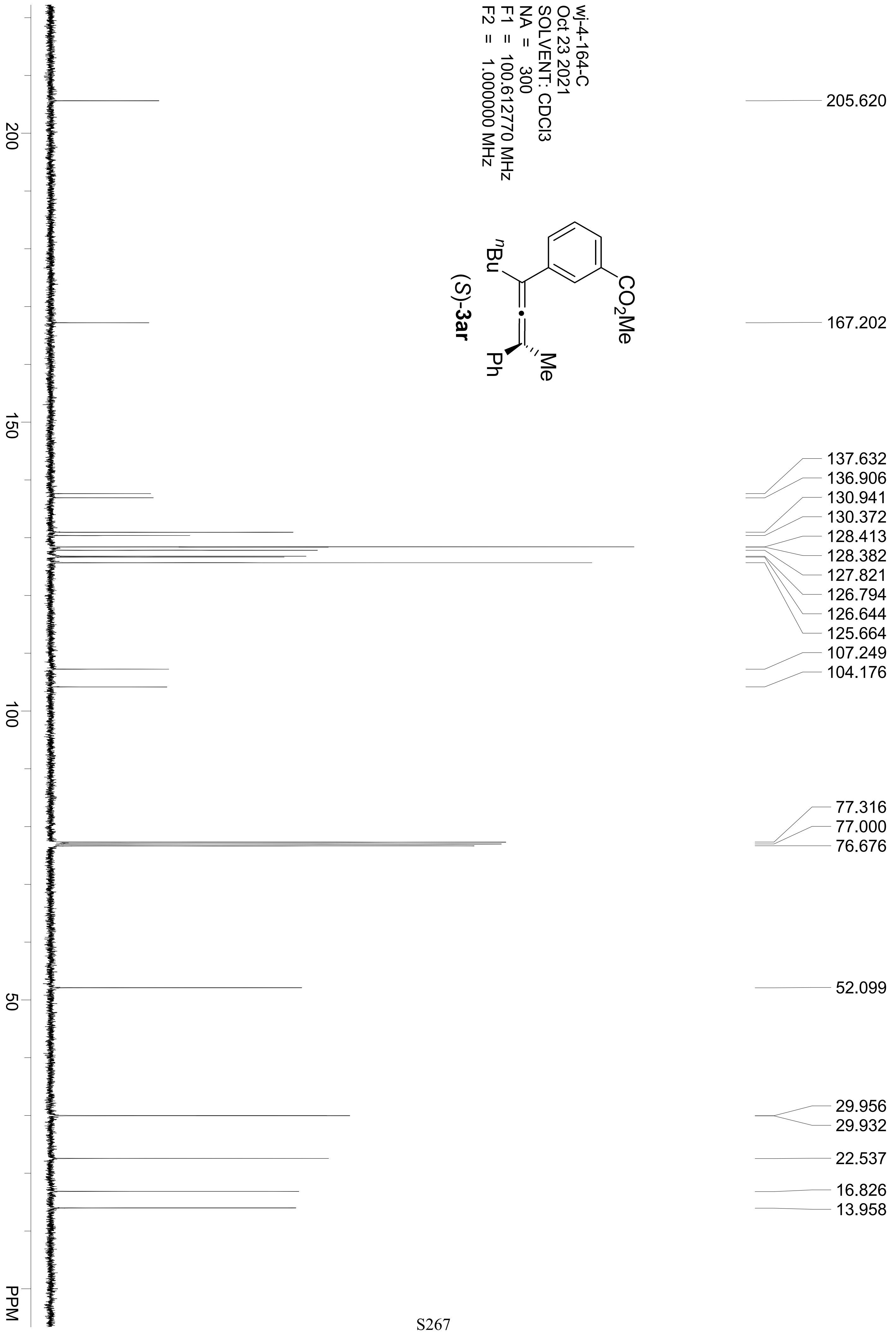

Supplementary Figure 195. ^{13}C NMR (100 MHz, $CDCl_3$) spectrum for (*S*)-**3ar**

Area Percent Report

sample wj-4-164-IC-99.5-0.5-0.5-214

Data file: C:\Users\Public\Documents\ChemStation\1\Data\WJ_LC 2021-10-23 14-45-23\003-P2-C3-wj-4-164.D

Acquisition Data:

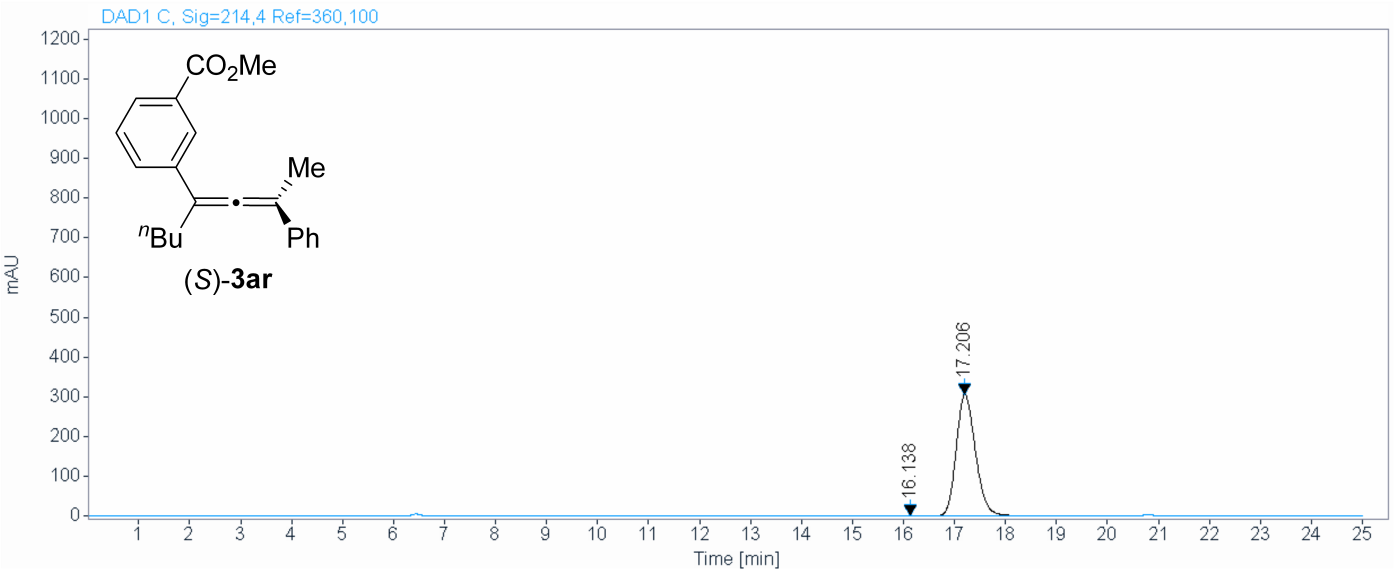

Signal: DAD1 C, Sig=214,4 Ref=360,100

RT [min]	Width [min]	Height	Area	Area%
16.138	0.5173	1.3567	42.1118	0.5236
17.206	0.4055	305.5087	8000.7871	99.4764
		Sum	8042.8989	100.0000

Supplementary Figure 196. HPLC spectrum for (*S*)-**3ar**

Area Percent Report

Agilent Technologies

sample wj-4-164-rac-IC-99.5-0.5-0.5-214

Data file: C:\Users\Public\Documents\ChemStation\1\Data\WJ_LC 2021-10-23 14-45-23\002-P2-C4-wj-4-164-RAC.D

Acquisition Data:

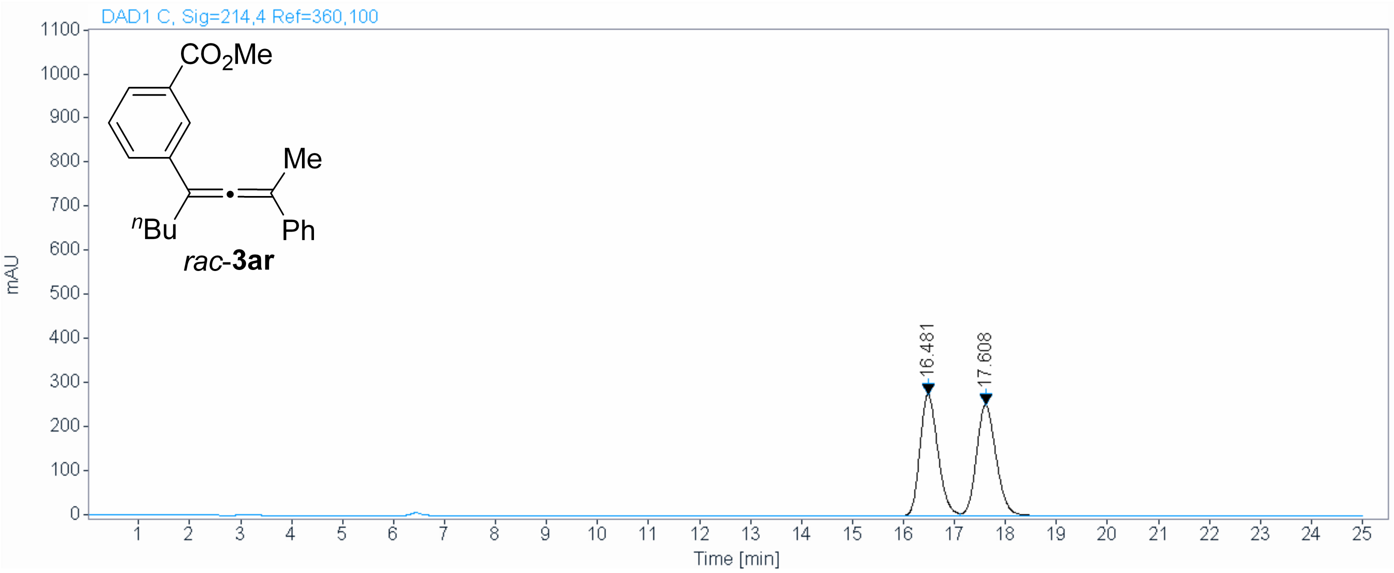

Signal: DAD1 C, Sig=214,4 Ref=360,100

RT [min]	Width [min]	Height	Area	Area%
16.481	0.3788	275.6980	6731.2705	49.8582
17.608	0.4172	252.1476	6769.5586	50.1418
		Sum	13500.8291	100.0000

Supplementary Figure 197. HPLC spectrum for *rac*-**3ar**

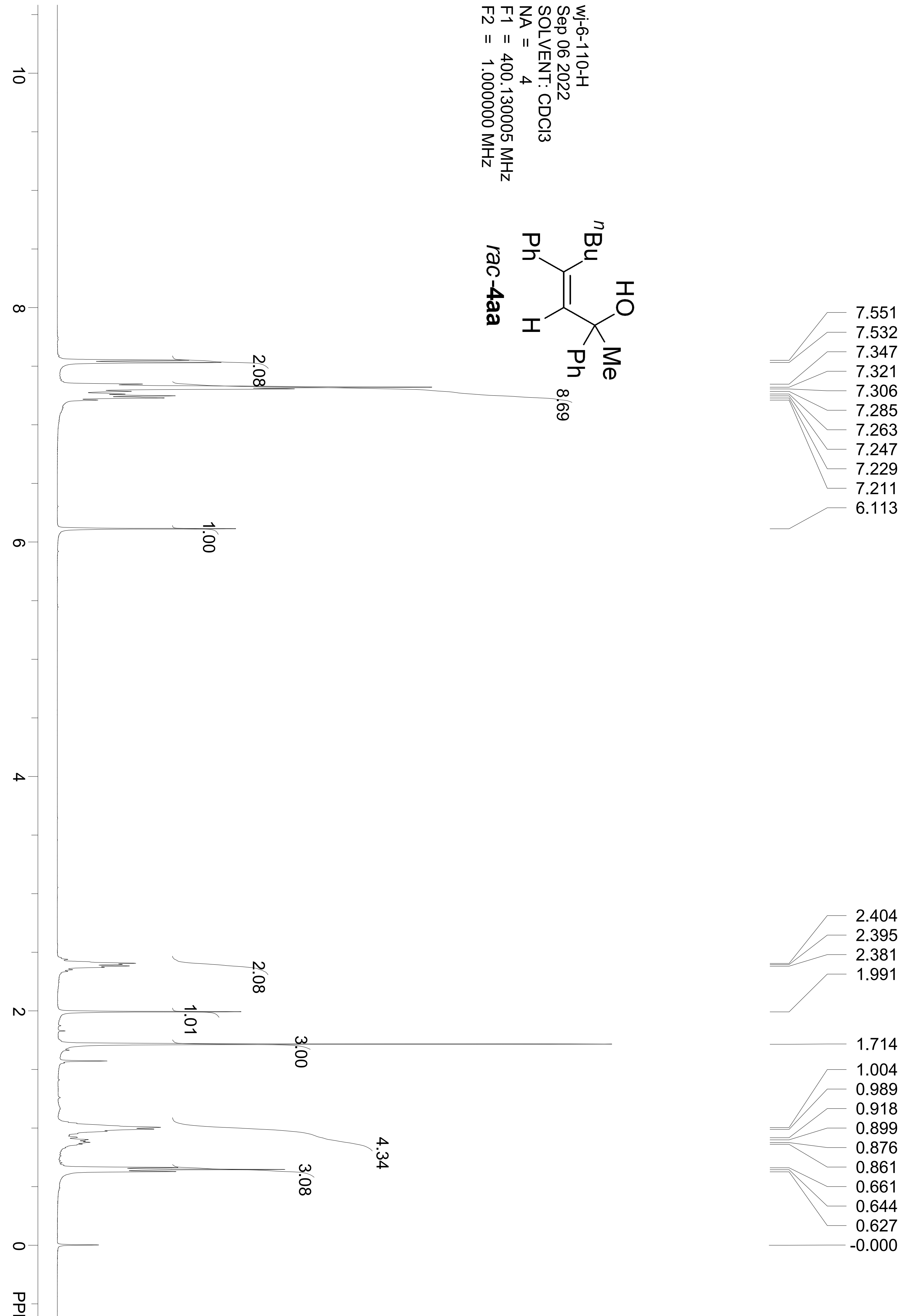

Supplementary Figure 198. ^{1}H NMR (400 MHz, $CDCl_3$) spectrum for *rac*-**4aa**

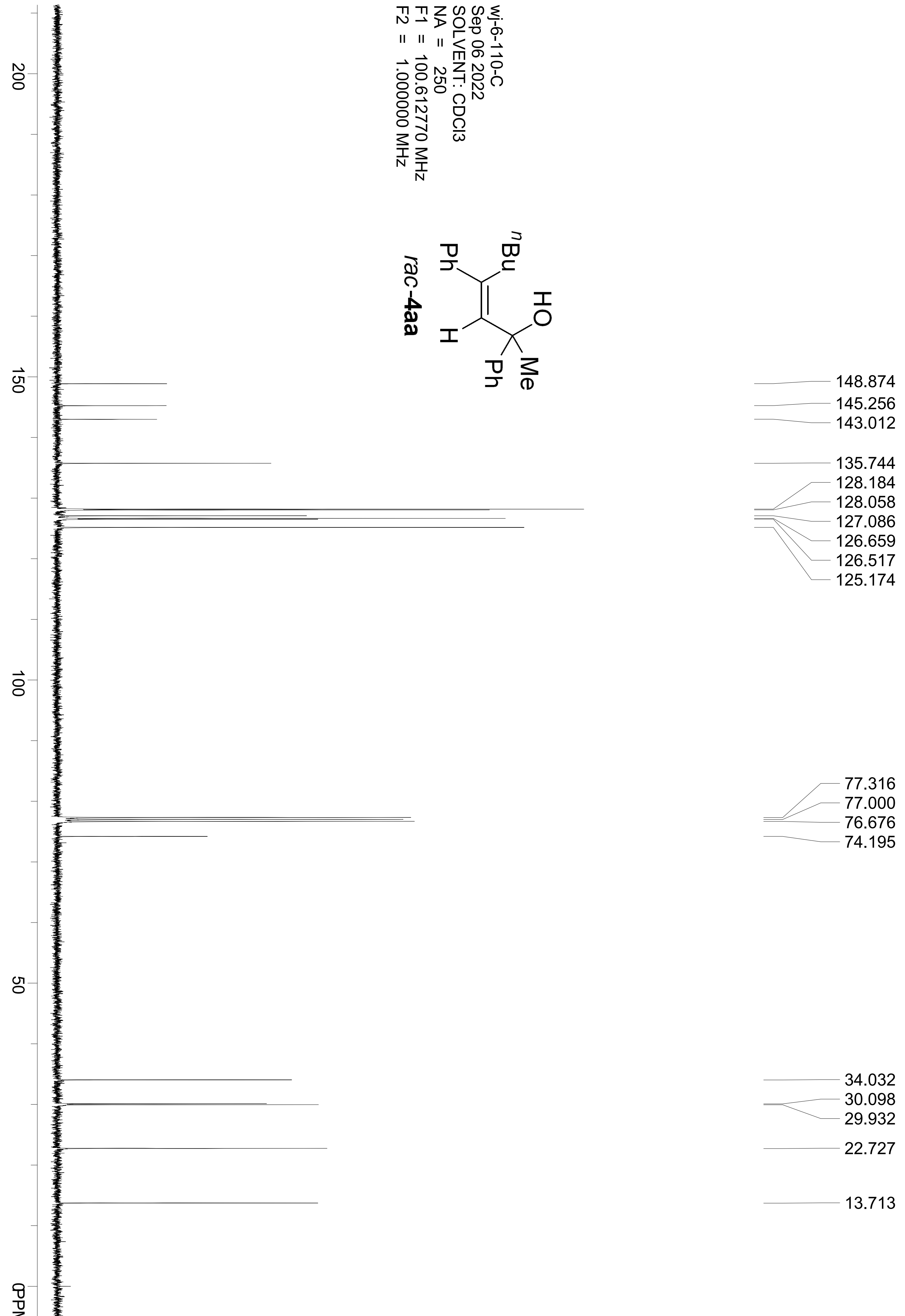

Supplementary Figure 199. ^{13}C NMR (100 MHz, $CDCl_3$) spectrum for *rac*-**4aa**

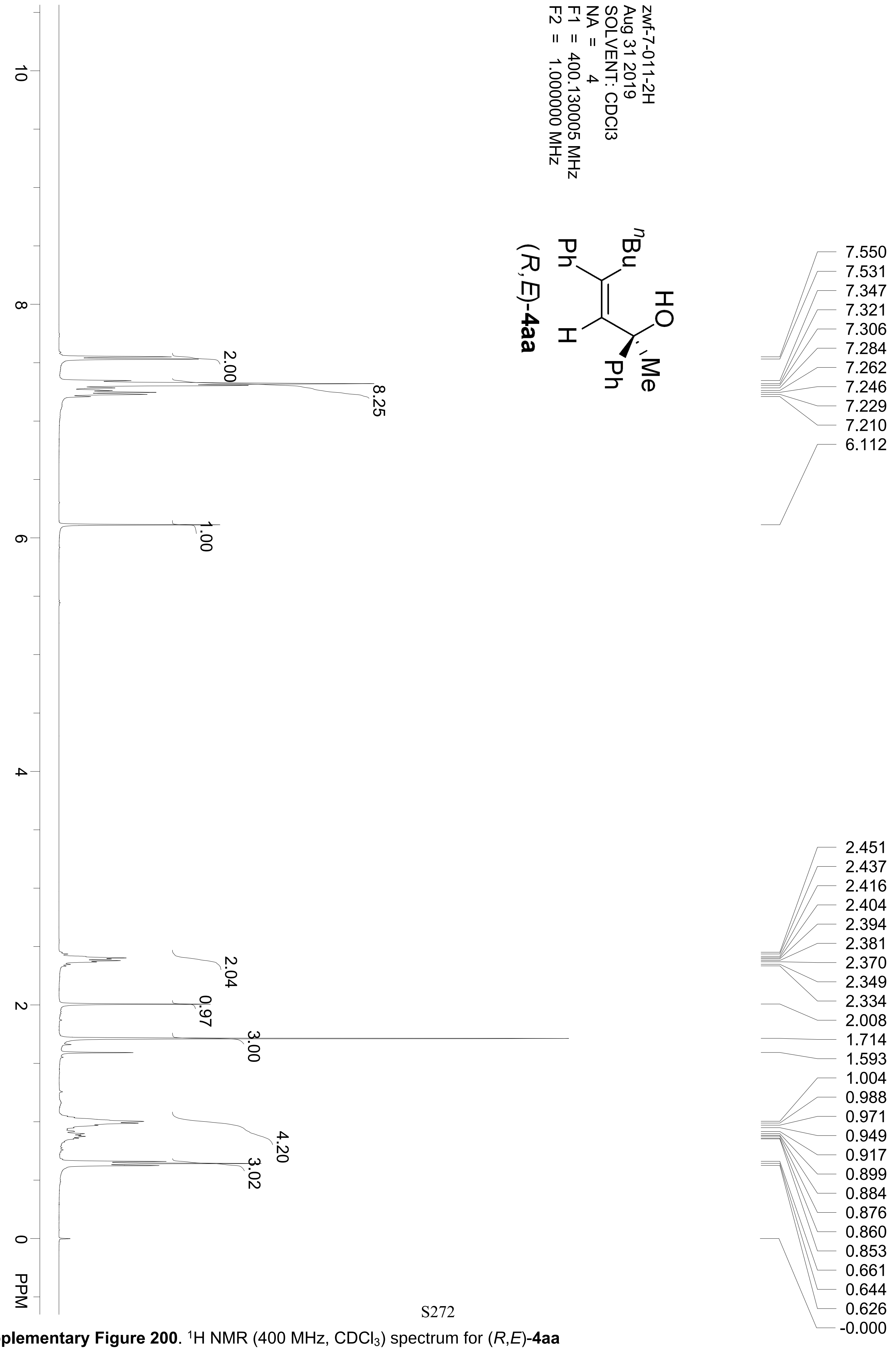

Supplementary Figure 200. ^{1}H NMR (400 MHz, $CDCl_3$) spectrum for (*R*,*E*)-**4aa**

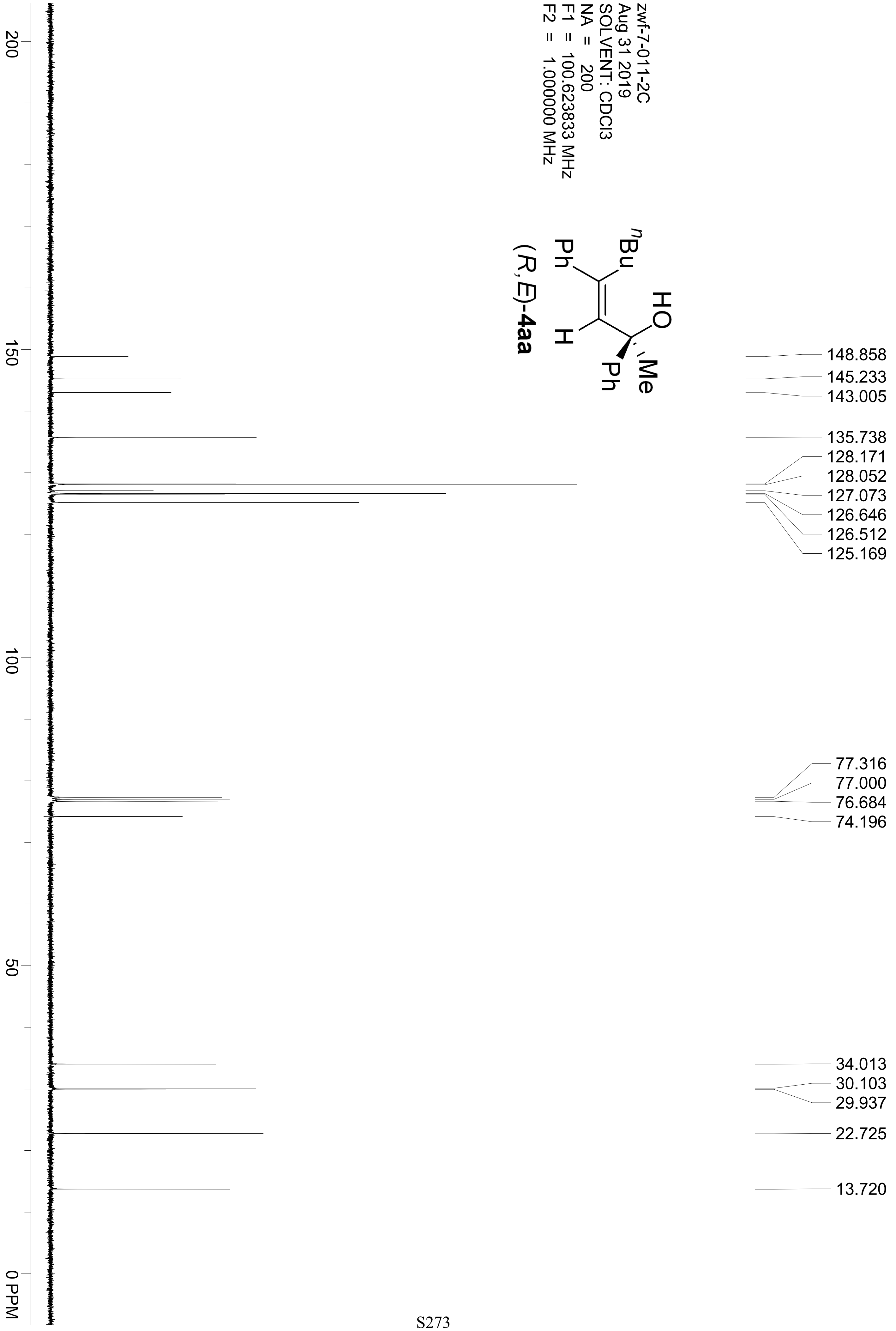

Supplementary Figure 201. ^{13}C NMR (100 MHz, $CDCl_3$) spectrum for (*R*,*E*)-**4aa**

Area Percent Report

Agilent Technologies

sample zwf-7-011-2-OD-H-90-10-1.0-214

Data file: C:\Users\Public\Documents\ChemStation\1\Data\zwf-allenioc acid_LC 2019-09-02 08-38-06\032-P1-C1-zwf-7-011-2.D

Acquisition Data:

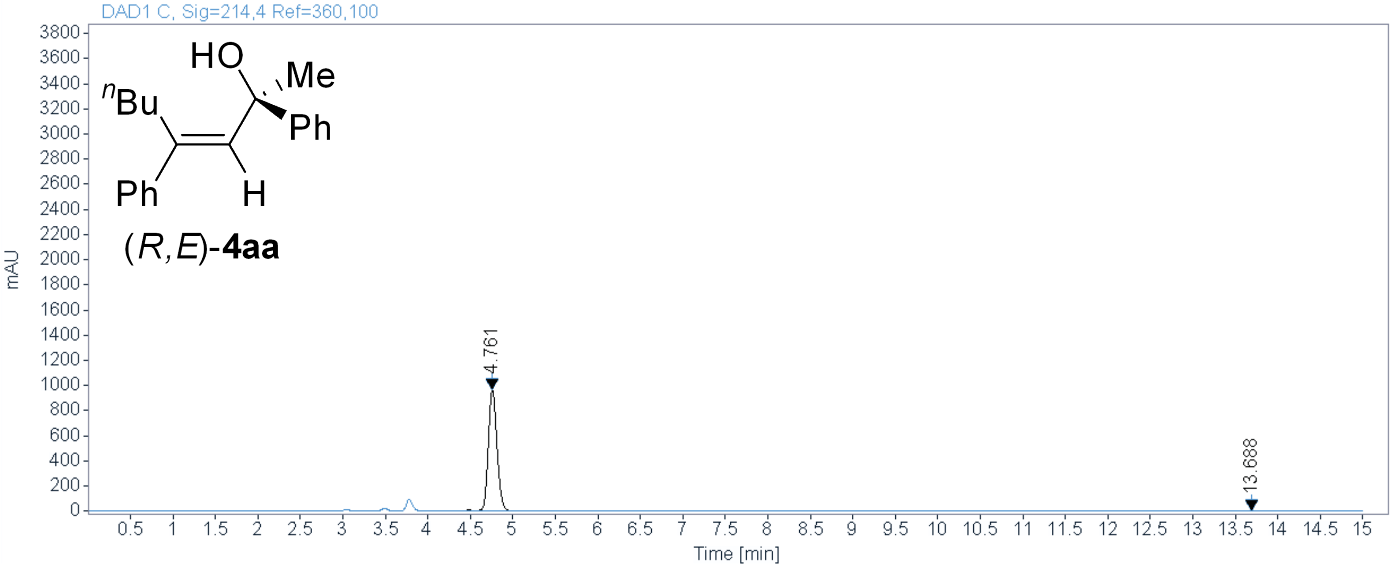

Signal: DAD1 C, Sig=214,4 Ref=360,100

RT [min]	Width [min]	Height	Area	Area%
4.761	0.1088	968.0427	6924.5059	99.0924
13.688	0.3465	2.4144	63.4255	0.9076
		Sum	6987.9314	100.0000

Supplementary Figure 202. HPLC spectrum for (*R*,*E*)-**4aa**

Area Percent Report

Agilent Technologies

sample zwf-7-011-2-rac-OD-H-90-10-1.0-214

Data file: C:\Users\Public\Documents\ChemStation\1\Data\zwf-allenioc acid_LC 2019-09-02 08-38-06\034-P1-C3-zwf-7-011-2-rac.D

Acquisition Data:

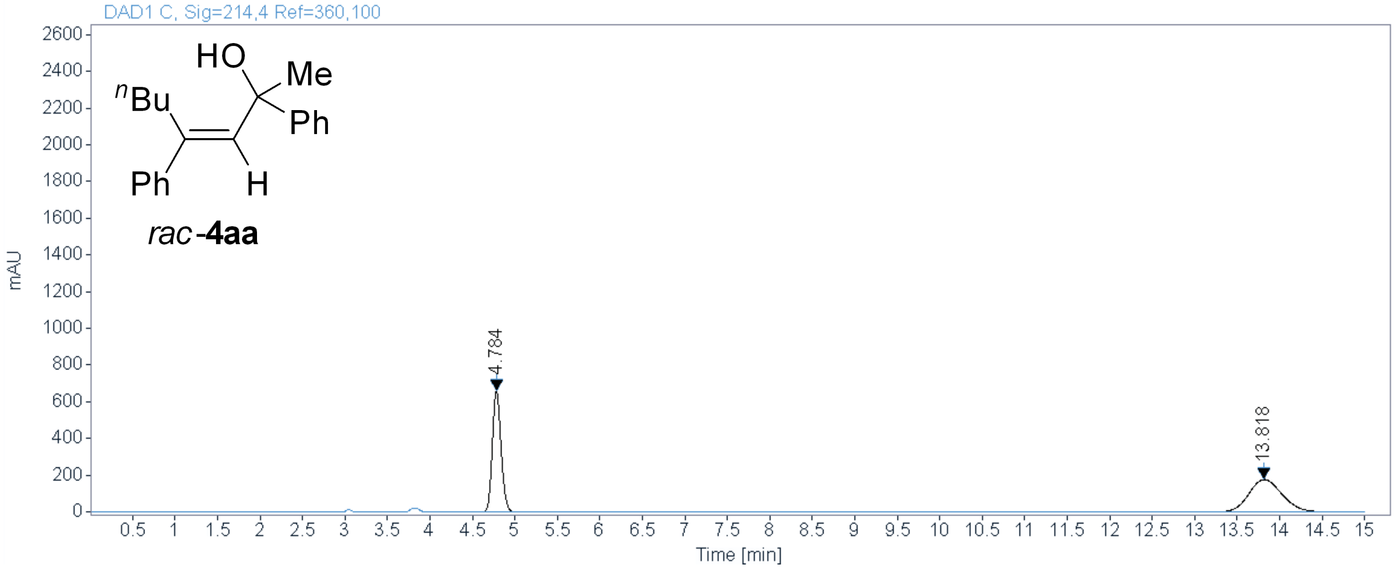

Signal: DAD1 C, Sig=214,4 Ref=360,100

RT [min]	Width [min]	Height	Area	Area%
4.784	0.1095	663.9503	4752.6680	49.8314
13.818	0.4226	176.2953	4784.8237	50.1686
		Sum	9537.4917	100.0000

Supplementary Figure 203. HPLC spectrum for *rac*-**4aa**

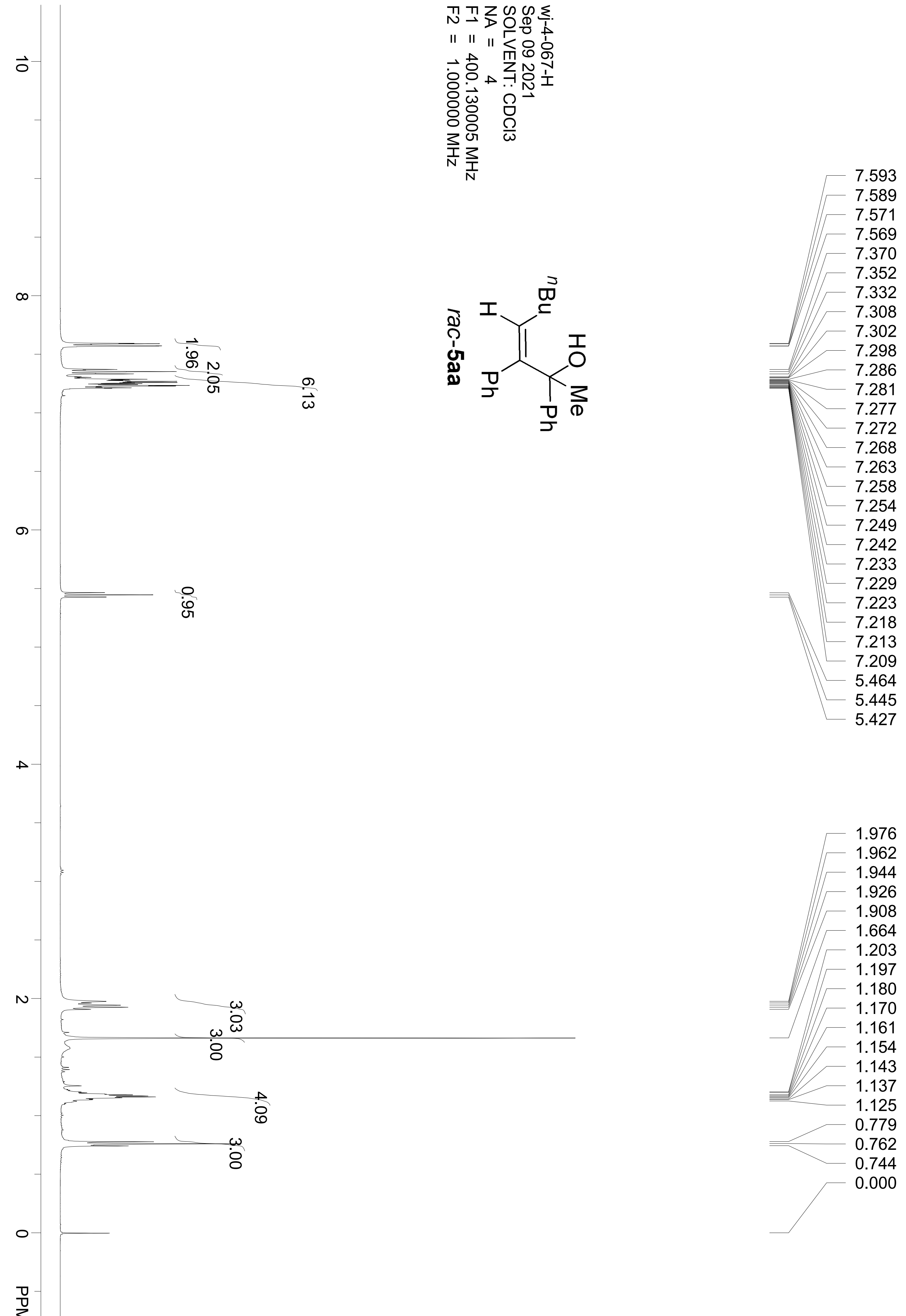

Supplementary Figure 204. ^{1}H NMR (400 MHz, $CDCl_3$) spectrum for *rac*-**5aa**

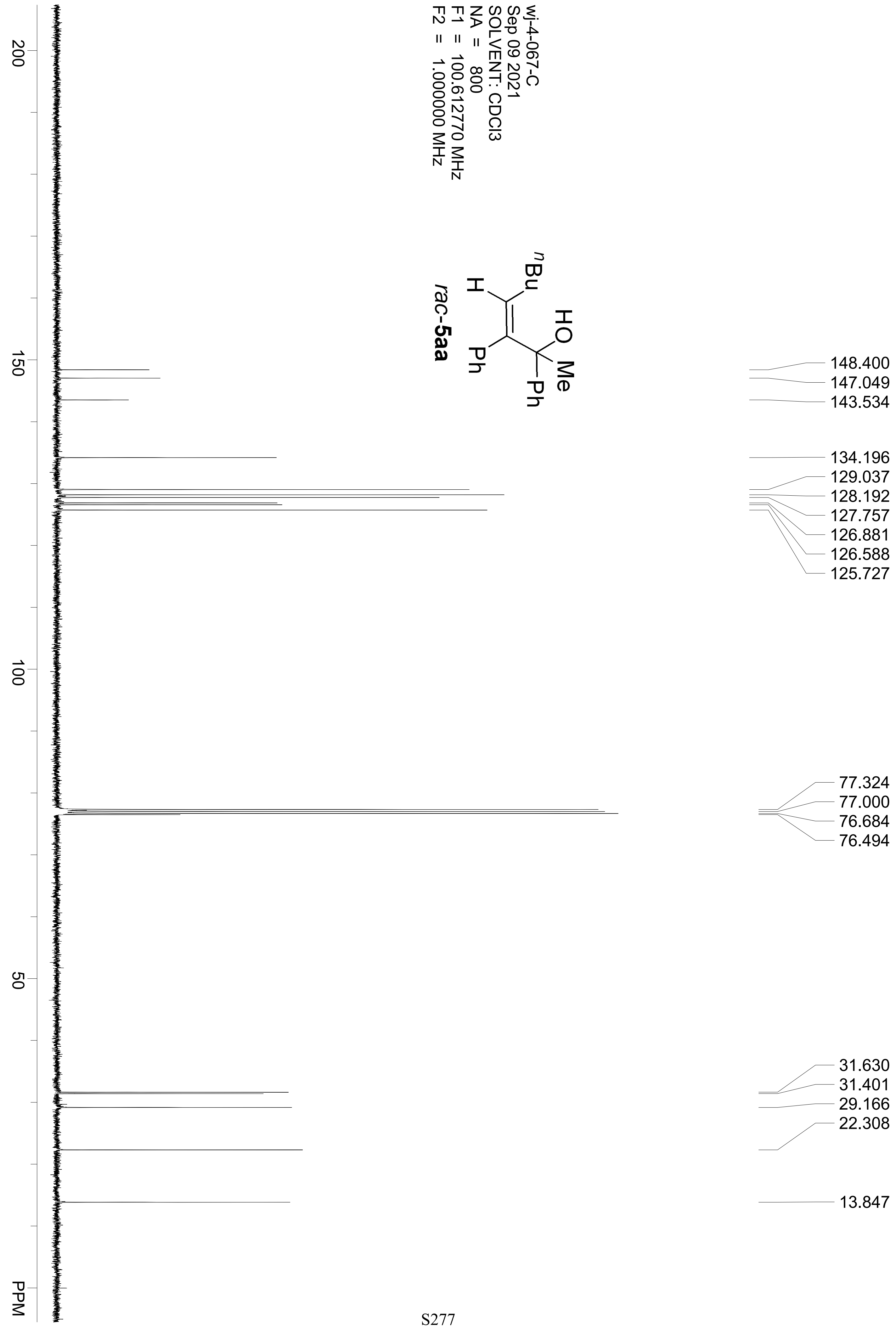

Supplementary Figure 205. ^{13}C NMR (100 MHz, $CDCl_3$) spectrum for *rac*-**5aa**

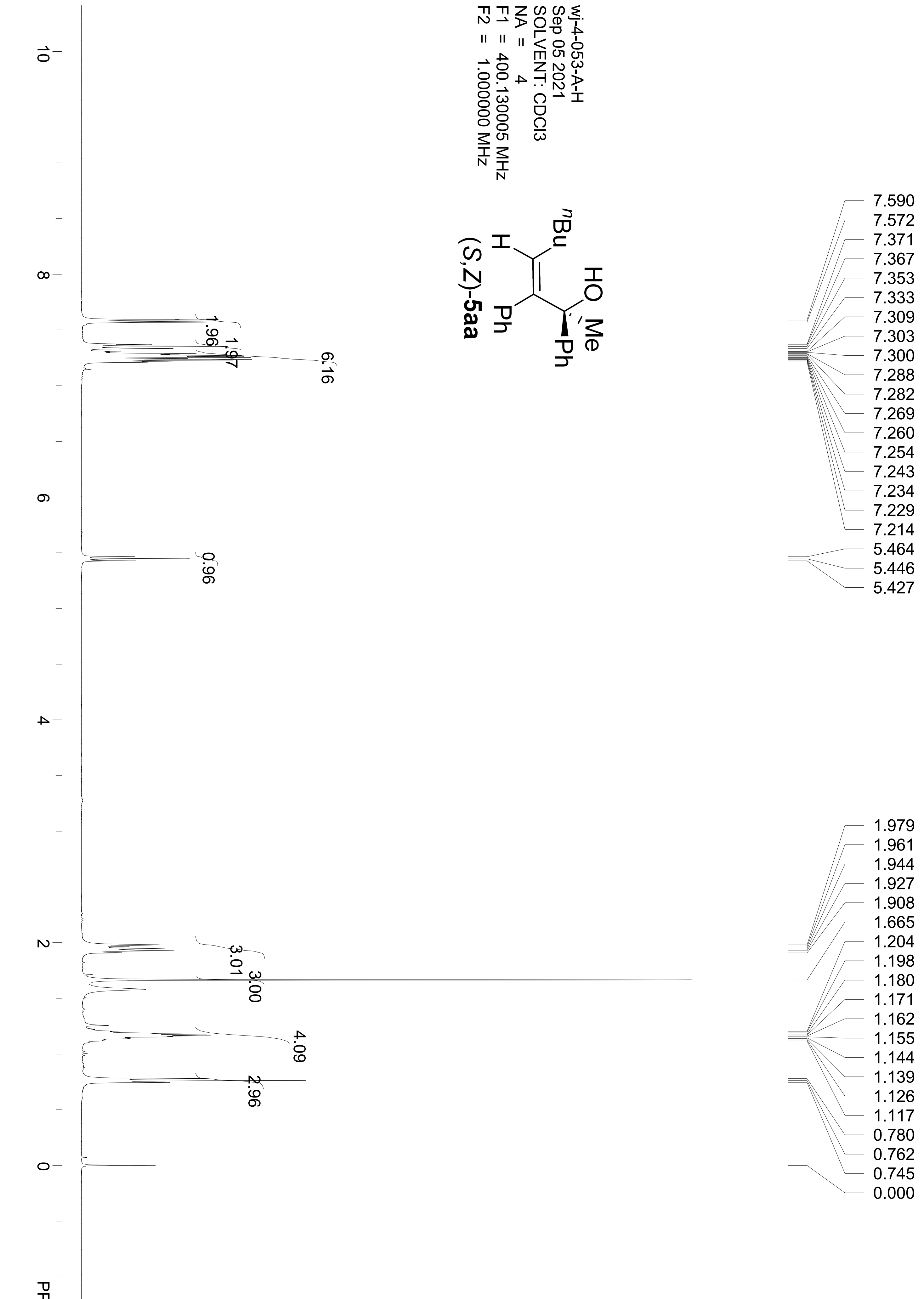

Supplementary Figure 206. ^{1}H NMR (400 MHz, $CDCl_3$) spectrum for (*S*,*Z*)-**5aa**

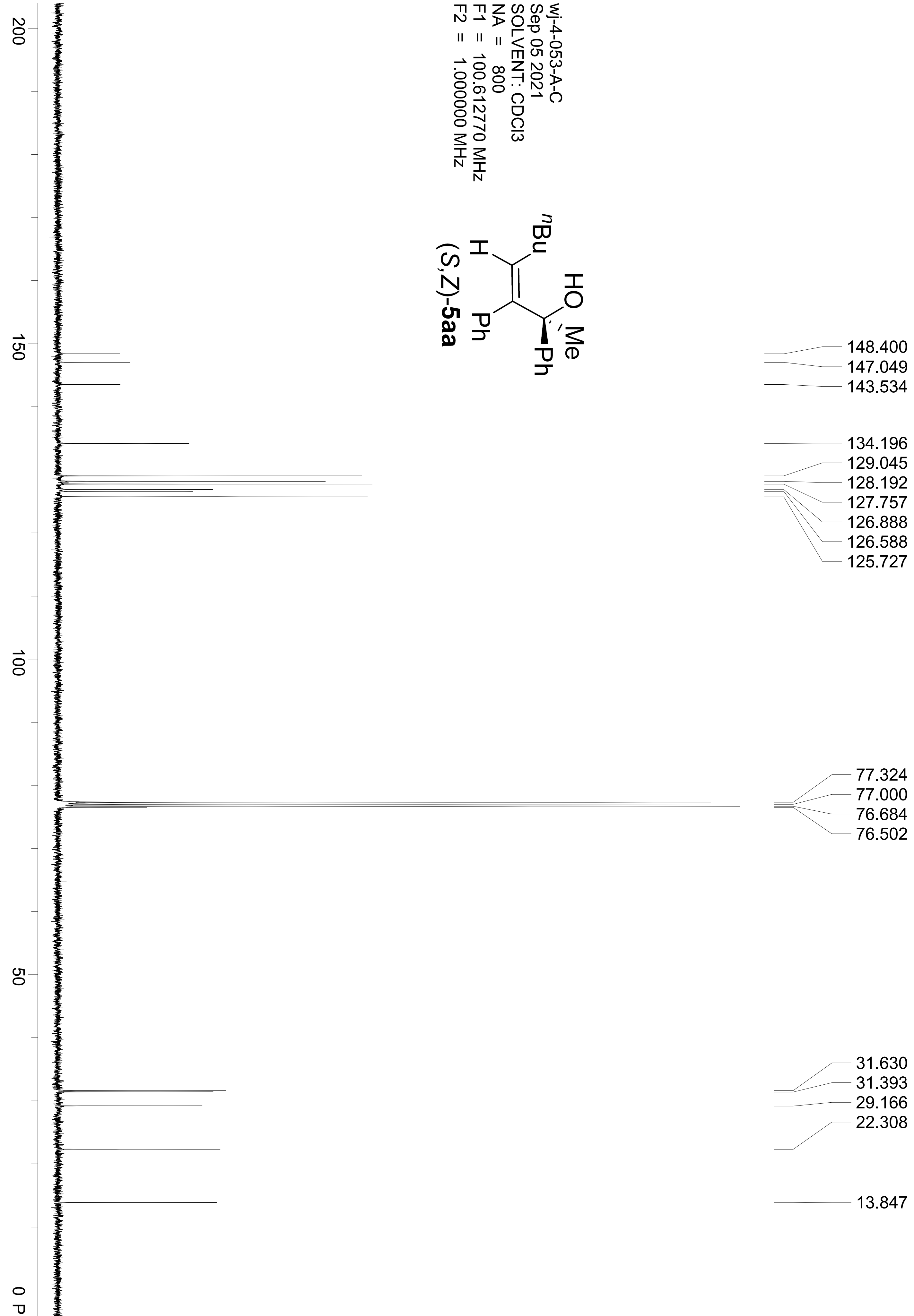

Supplementary Figure 207. ^{13}C NMR (100 MHz, $CDCl_3$) spectrum for (*S*,*Z*)-**5aa**

Area Percent Report

sample wj-4-053-A-IC-99-1-0.5-214

Data file: C:\Users\Public\Documents\ChemStation\1\Data\2023-06-24\HYK 2023-06-24 11-07-40\010-P2-C1-wj-4-053-A.D

Acquisition Data:

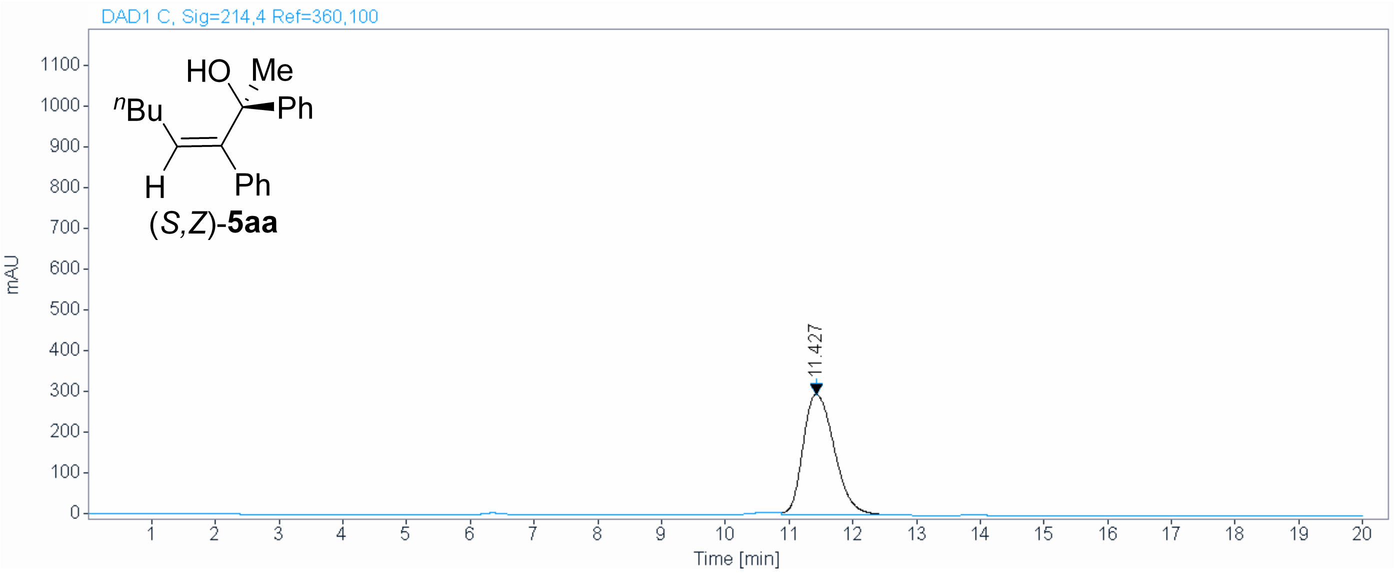

Signal: DAD1 C, Sig=214,4 Ref=360,100

RT [min]	Width [min]	Height	Area	Area%
11.427	0.5705	296.0749	10135.2637	100.0000
		Sum	10135.2637	100.0000

Supplementary Figure 208. HPLC spectrum for (*S*,*Z*)-**5aa**

Area Percent Report

sample wj-4-053-A-rac-IC-99-1-0.5-214

Data file: C:\Users\Public\Documents\ChemStation\1\Data\2023-06-24\HYK 2023-06-24 11-07-40\009-P2-C2-wj-4-053-A-rac.D

Acquisition Data:

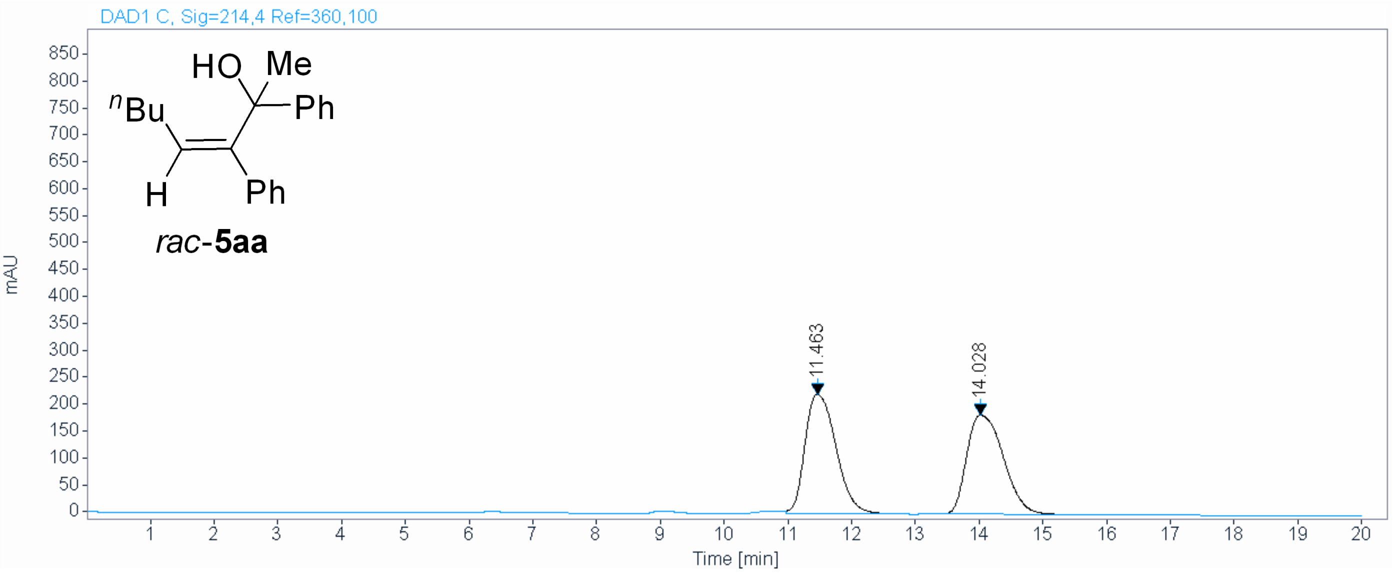

Signal: DAD1 C, Sig=214,4 Ref=360,100

RT [min]	Width [min]	Height	Area	Area%
11.463	0.5657	221.7226	7525.2817	50.4411
14.028	0.6445	184.1093	7393.6680	49.5589
		Sum	14918.9497	100.0000

Supplementary Figure 209. HPLC spectrum for *rac*-5**aa**

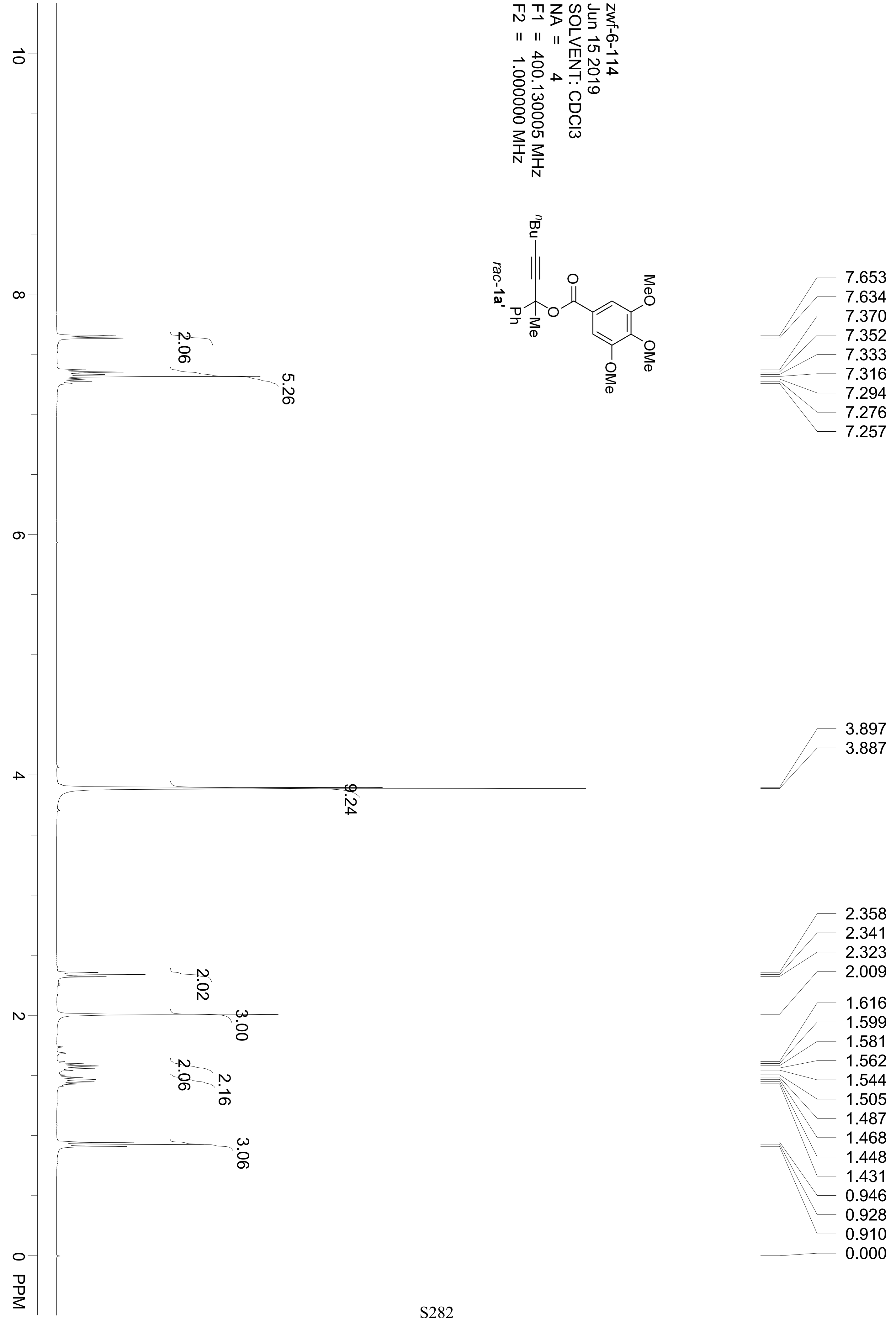

Supplementary Figure 210. ^{1}H NMR (400 MHz, $CDCl_3$) spectrum for *rac*-**1a'**

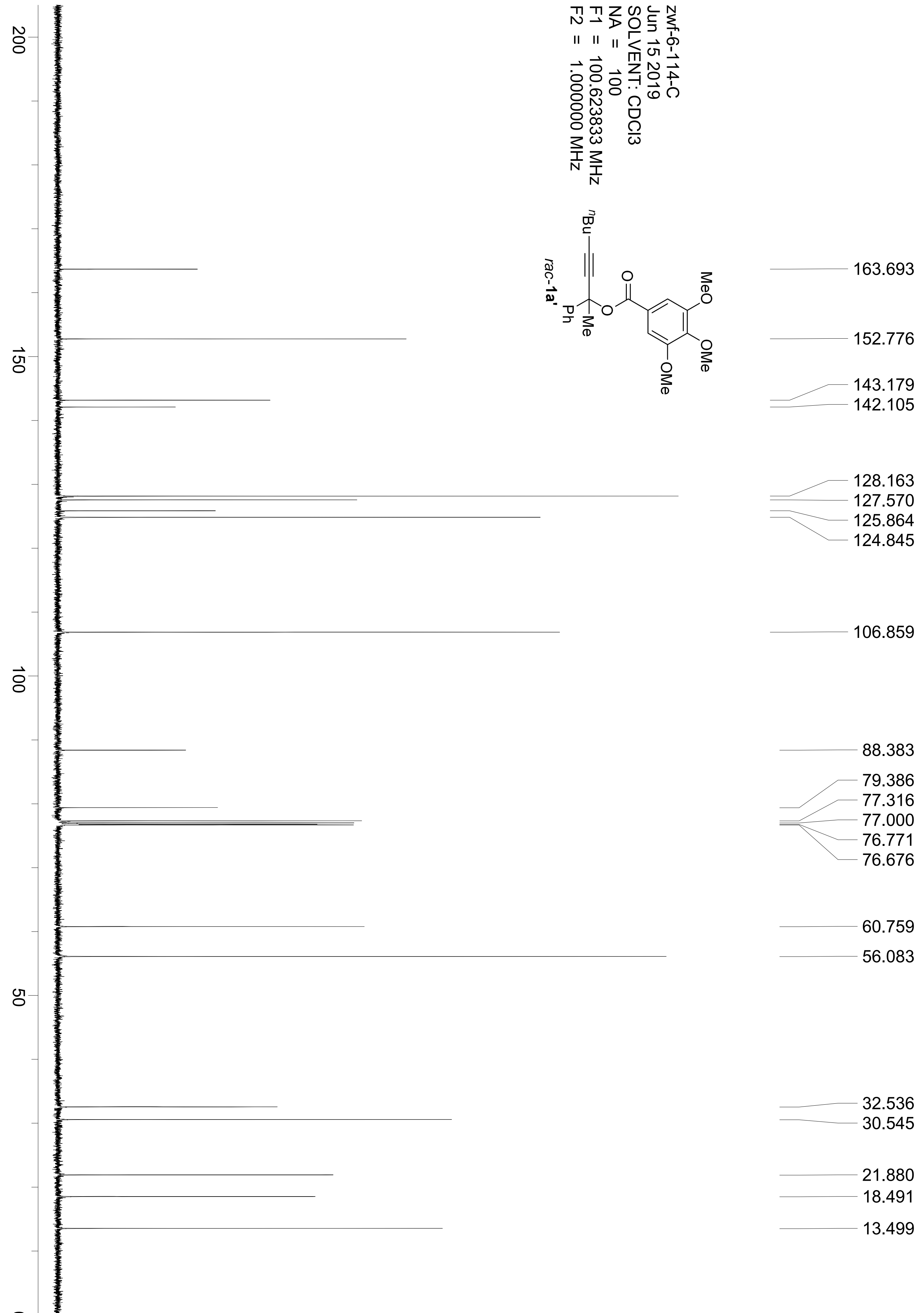

Supplementary Figure 211. ^{13}C NMR (100 MHz, $CDCl_3$) spectrum for *rac*-**1a'**

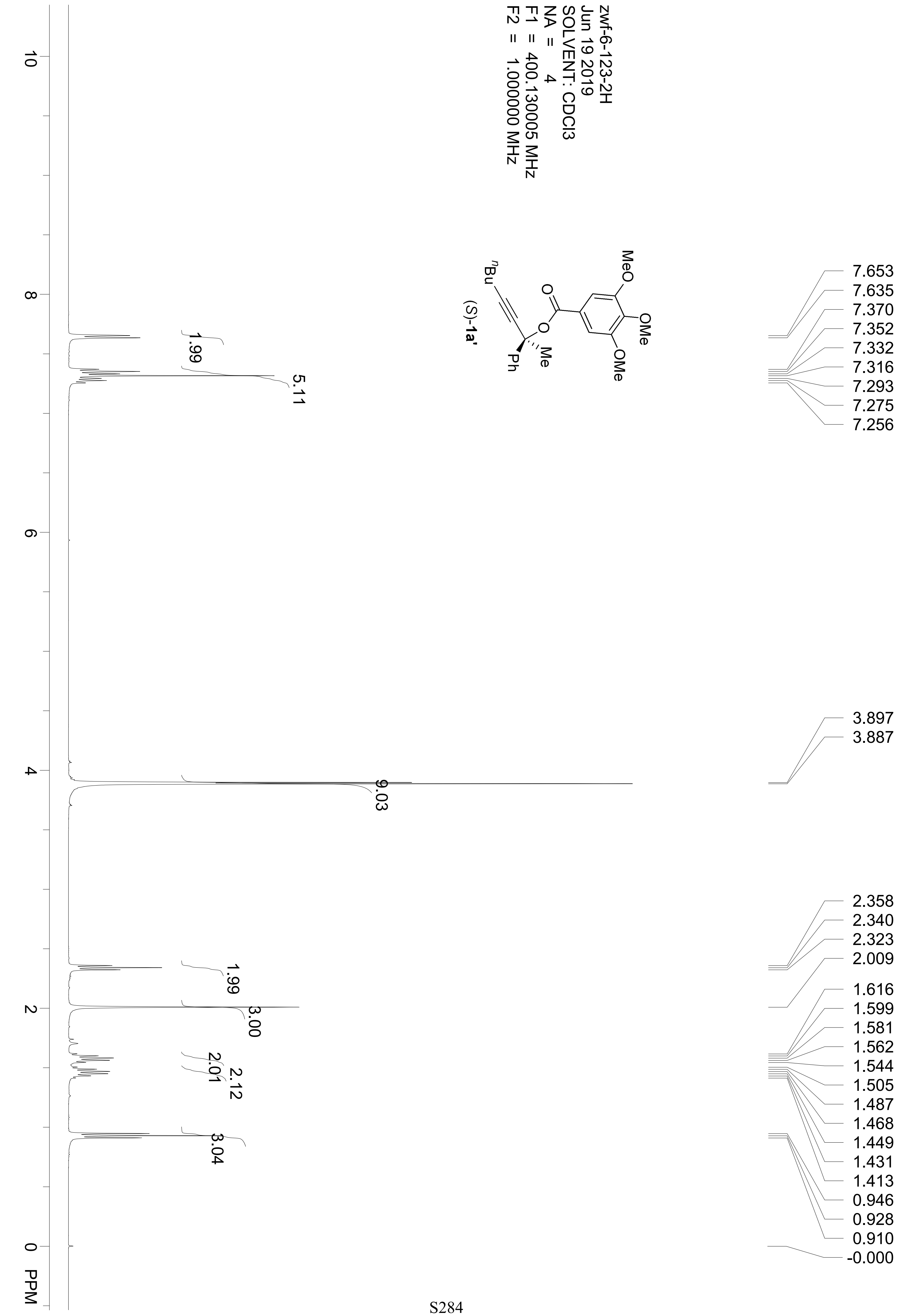

Supplementary Figure 212. ^{1}H NMR (400 MHz, $CDCl_3$) spectrum for (*S*)-**1a'**

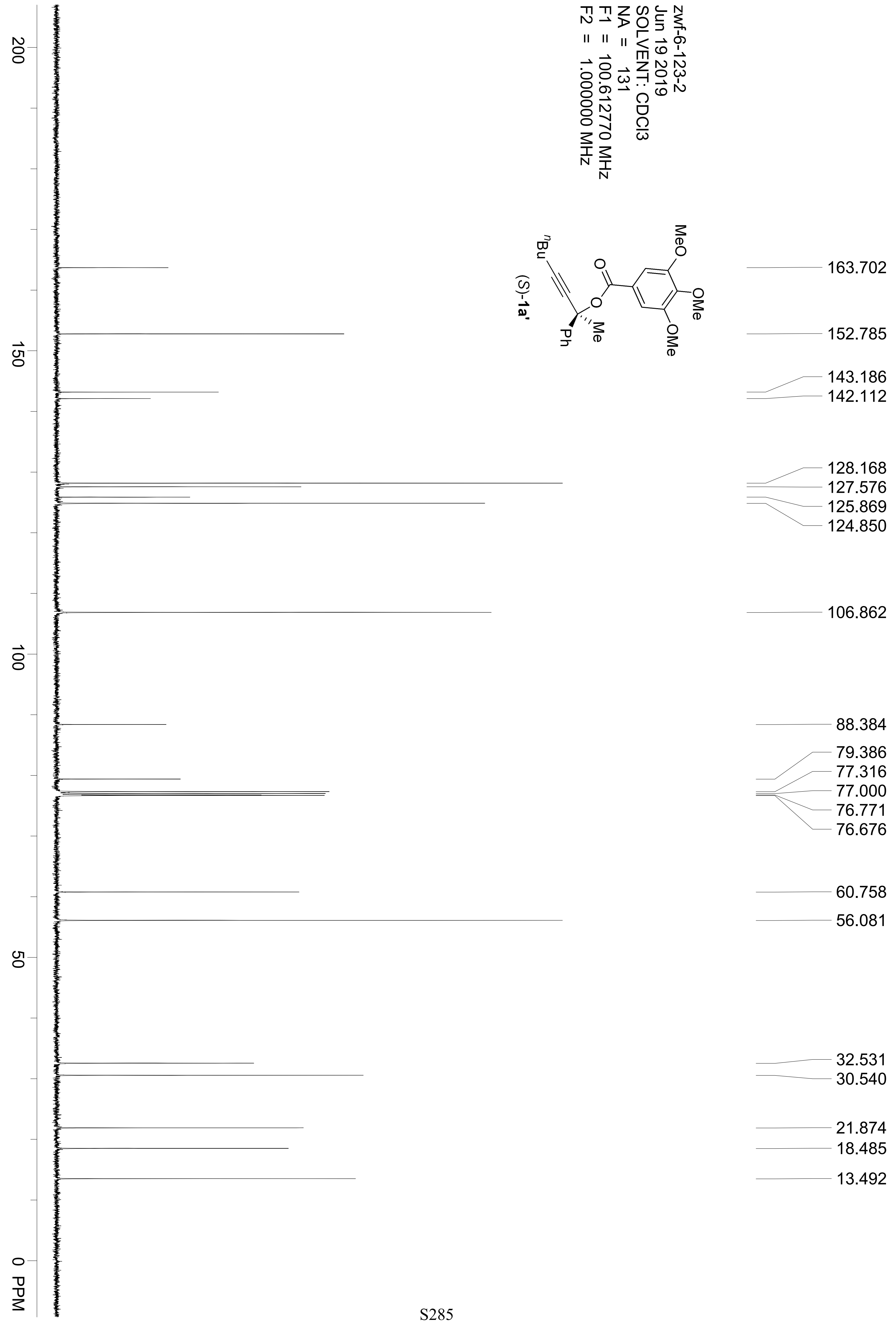

Supplementary Figure 213. ^{13}C NMR (100 MHz, $CDCl_3$) spectrum for (*S*)-**1a'**

Area Percent Report

Agilent Technologies

sample zwf-6-123-2-AD-H-98-2-1.0-214

Data file: C:\Users\Public\Documents\ChemStation\1\Data\zwf-allenioc acid_LC 2019-06-19 16-22-24\003-P1-C1-zwf-6-123-2.D

Acquisition Data: 6/19/2019 5:00:20 PM

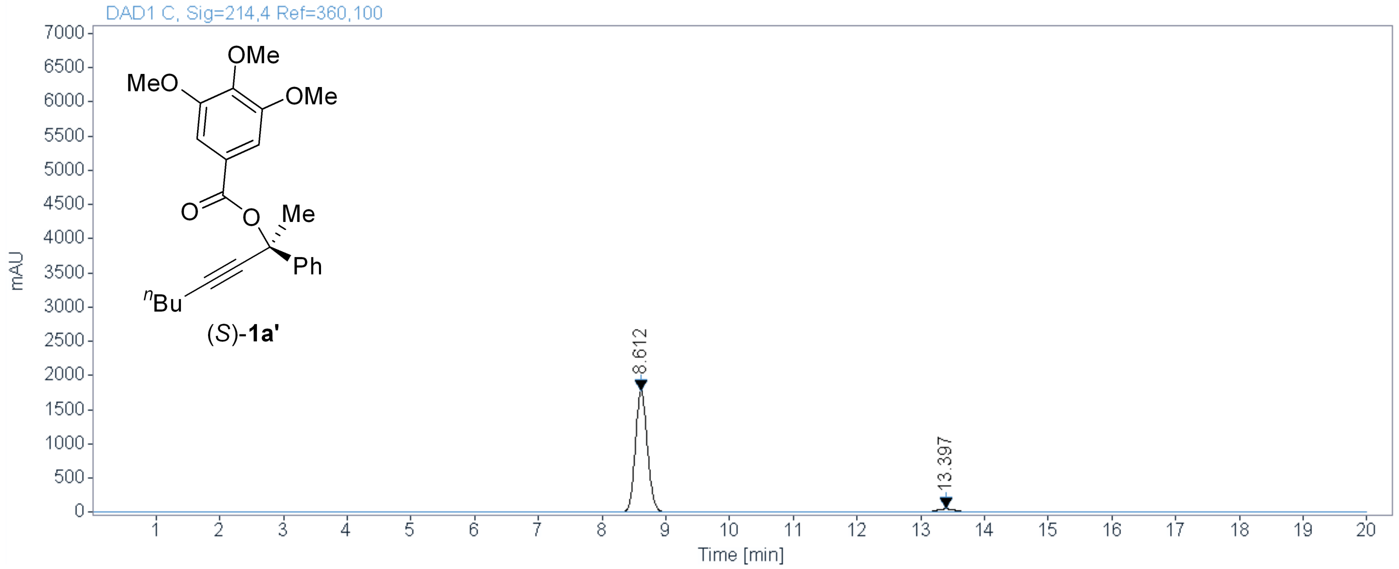

Signal: DAD1 C, Sig=214,4 Ref=360,100

RT [min]	Width [min]	Height	Area	Area%
8.612	0.2055	1780.5854	23494.4609	95.6399
13.397	0.3406	48.6948	1071.0924	4.3601
		Sum	24565.5533	100.0000

Supplementary Figure 214. HPLC spectrum for (*S*)-**1a'**

Area Percent Report

Agilent Technologies

sample zwf-6-123-2-rac-AD-H-98-2-1.0-214

Data file: C:\Users\Public\Documents\ChemStation\1\Data\zwf-allenioc acid_LC 2019-06-19 16-22-24\004-P1-C2-zwf-6-123-2-rac.D

Acquisition Data: 6/19/2019 5:21:18 PM

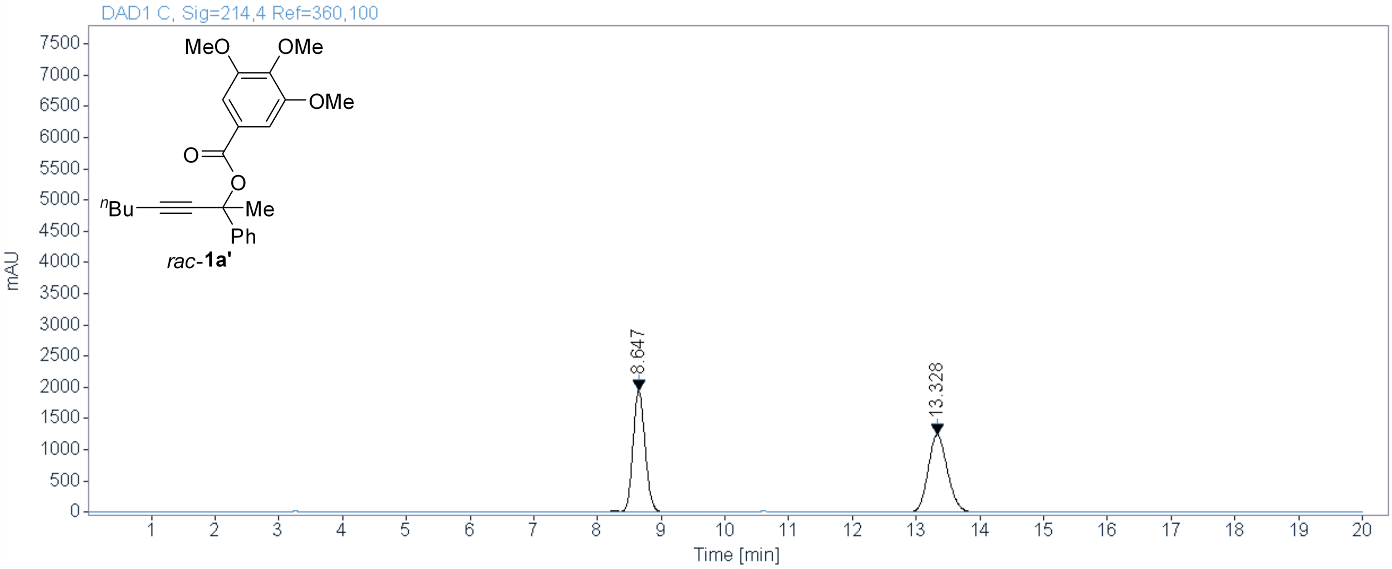

Signal: DAD1 C, Sig=214,4 Ref=360,100

RT [min]	Width [min]	Height	Area	Area%
8.647	0.2066	1950.3397	26072.8730	49.9448
13.328	0.3280	1239.2561	26130.5215	50.0552
		Sum	52203.3945	100.0000

Supplementary Figure 215. HPLC spectrum for *rac*-**1a'**

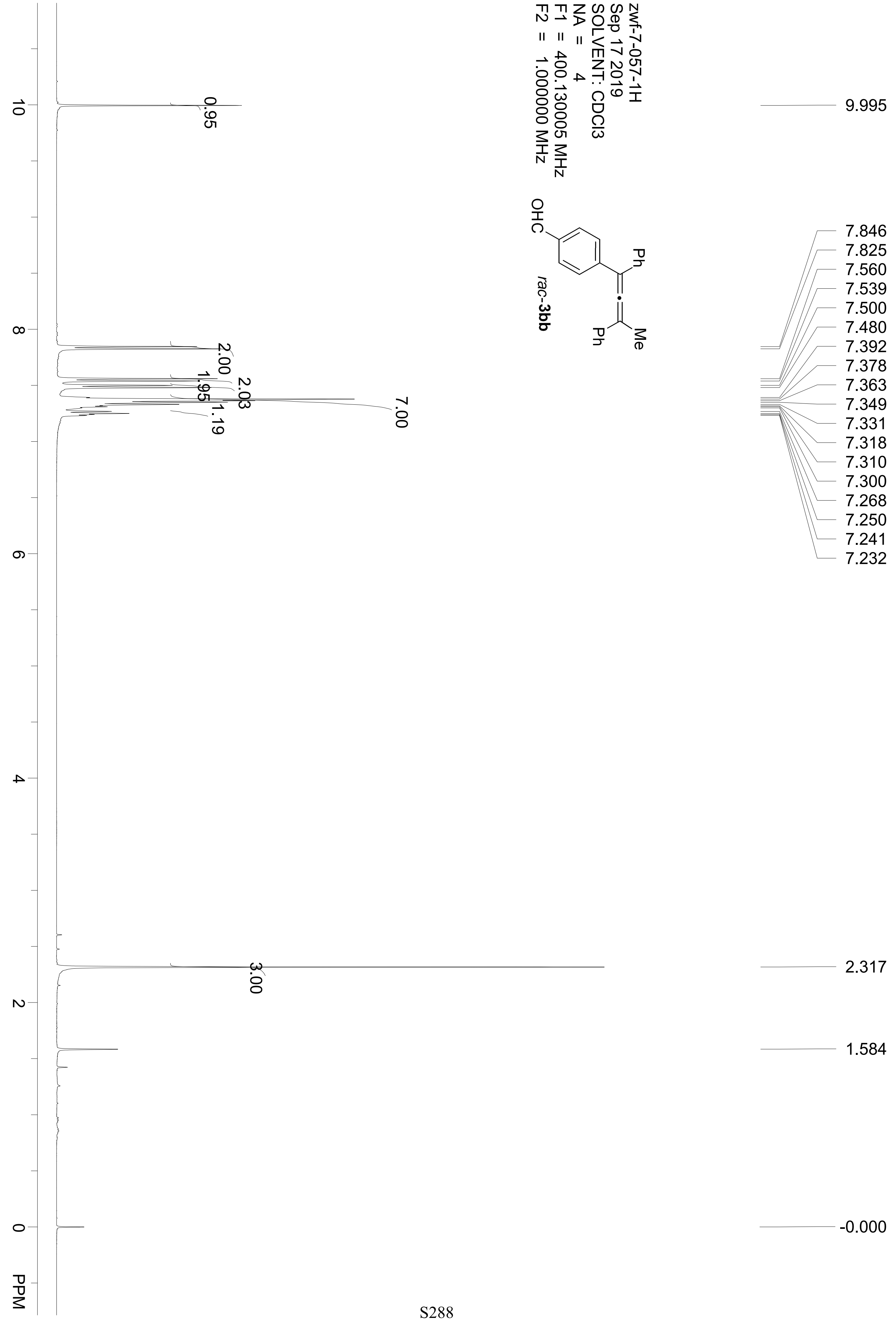

Supplementary Figure 216. ^{1}H NMR (400 MHz, $CDCl_3$) spectrum for *rac*-**3bb**

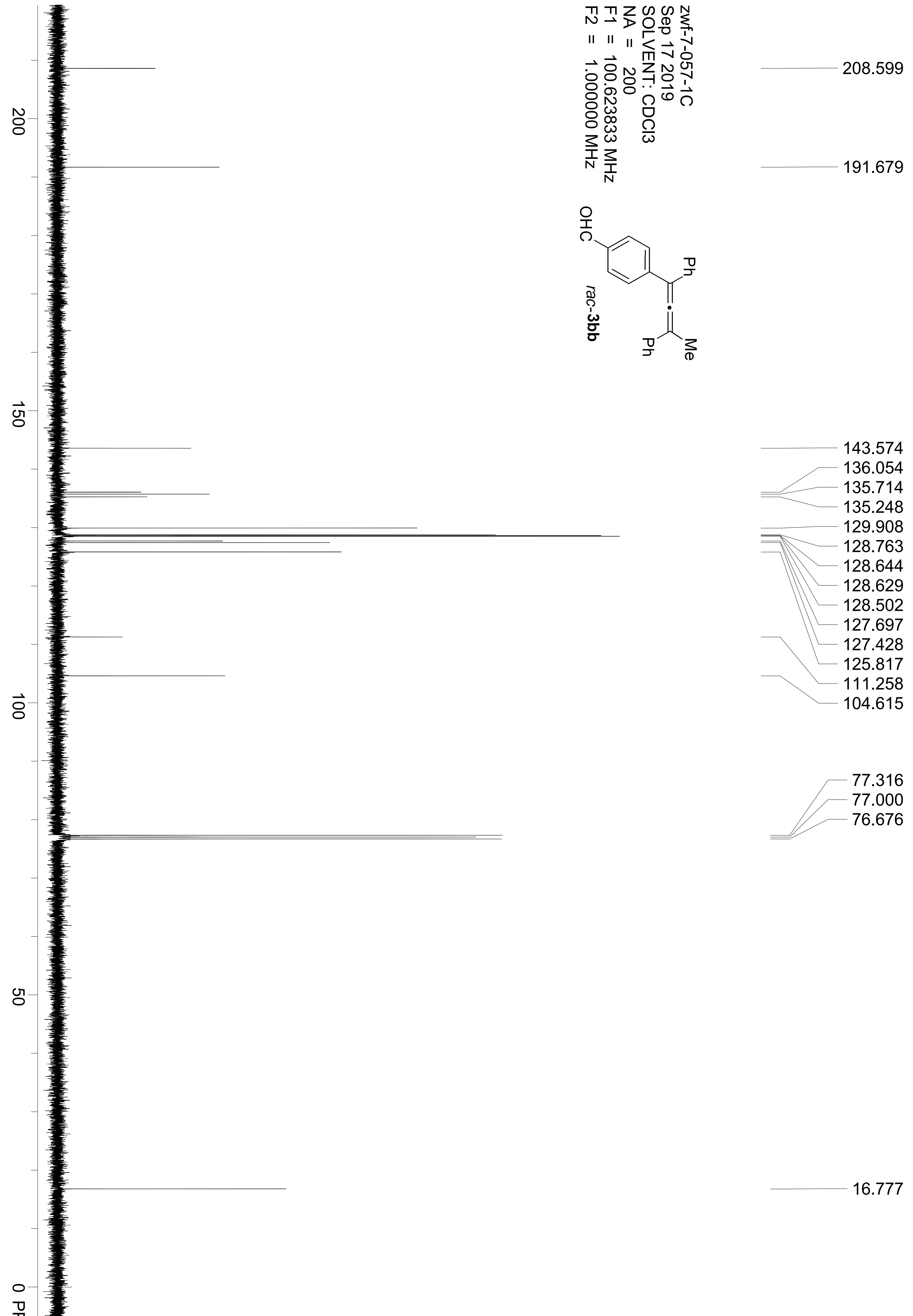

Supplementary Figure 217. ^{13}C NMR (100 MHz, $CDCl_3$) spectrum for *rac*-**3bb**

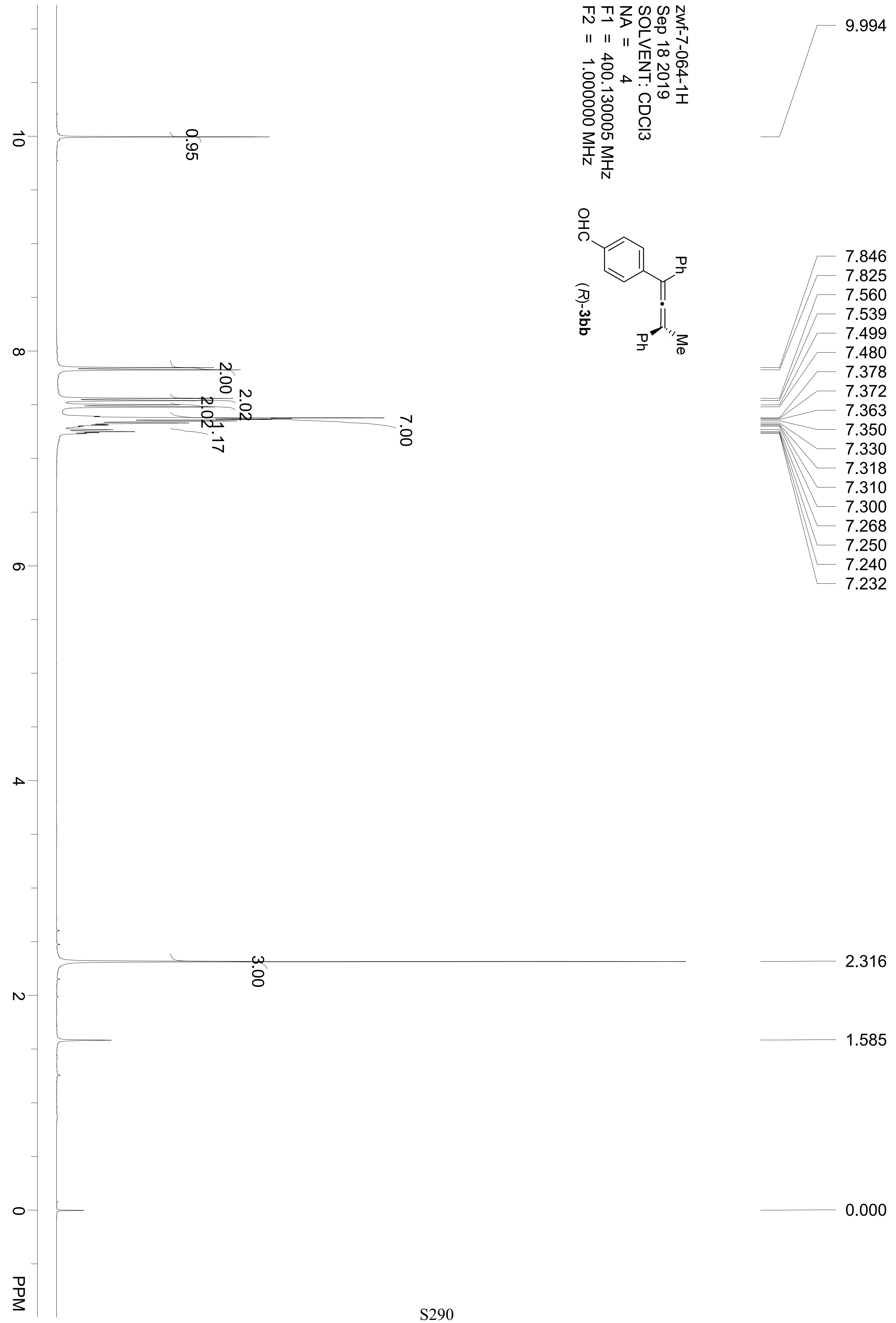

Supplementary Figure 218. ^{1}H NMR (400 MHz, $CDCl_3$) spectrum for (*R*)-**3bb**

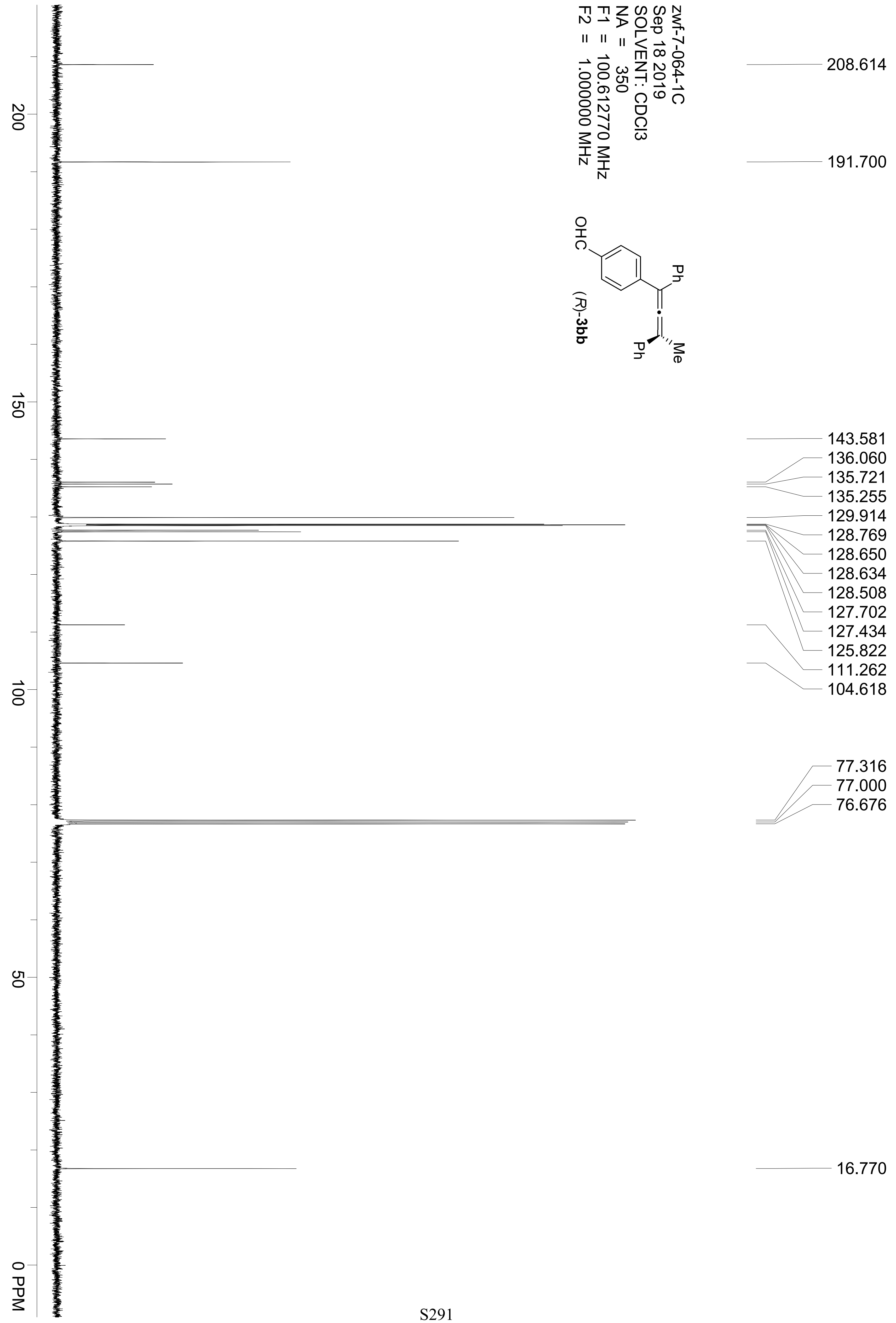

Supplementary Figure 219. ^{13}C NMR (100 MHz, $CDCl_3$) spectrum for (*R*)-**3bb**

Area Percent Report

Agilent Technologies

sample zwf-7-064-1-OD-H-98-2-1.0-214

Data file: C:\Users\Public\Documents\ChemStation\1\Data\wgl 2019-09-18 08-46-24\026-P1-C8-zwf-7-064-1.D

Acquisition Data:

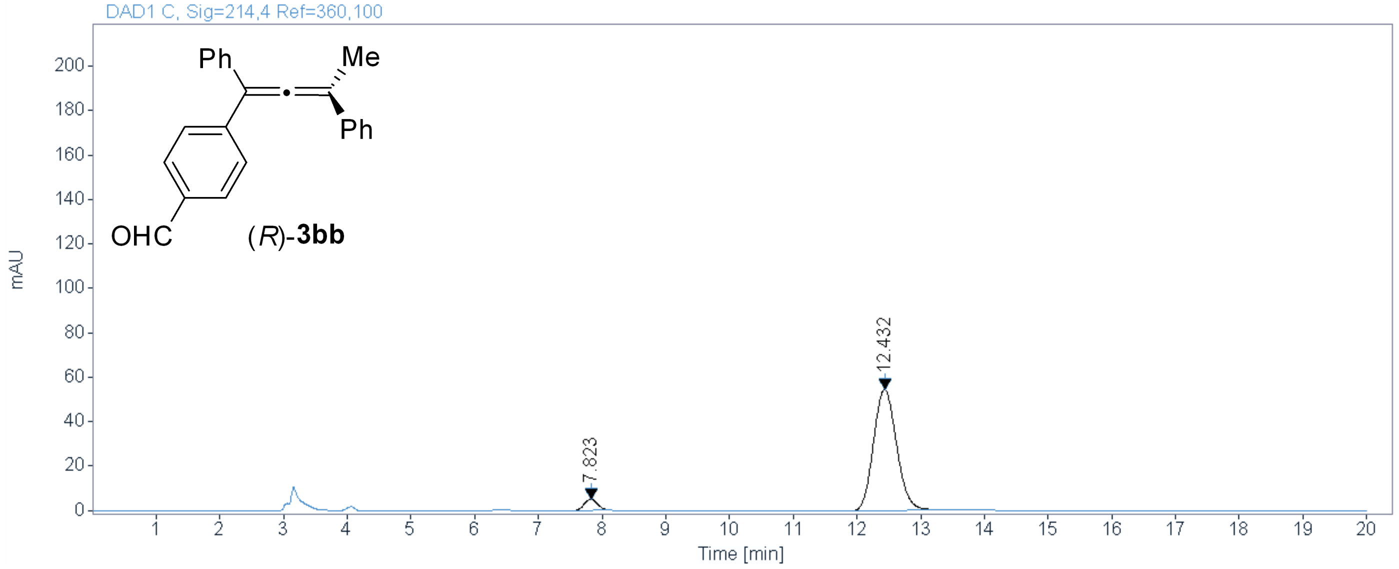

Signal: DAD1 C, Sig=214,4 Ref=360,100

RT [min]	Width [min]	Height	Area	Area%
7.823	0.2372	5.0199	71.4549	4.8959
12.432	0.3979	54.7247	1388.0266	95.1041
		Sum	1459.4815	100.0000

Supplementary Figure 220. HPLC spectrum for (*R*)-**3bb**

Area Percent Report

Agilent Technologies

sample zwf-7-064-1-rac-OD-H-98-2-1.0-214

Data file: C:\Users\Public\Documents\ChemStation\1\Data\wgl 2019-09-18 08-46-24\027-P1-C9-zwf-7-064-1-rac.D

Acquisition Data:

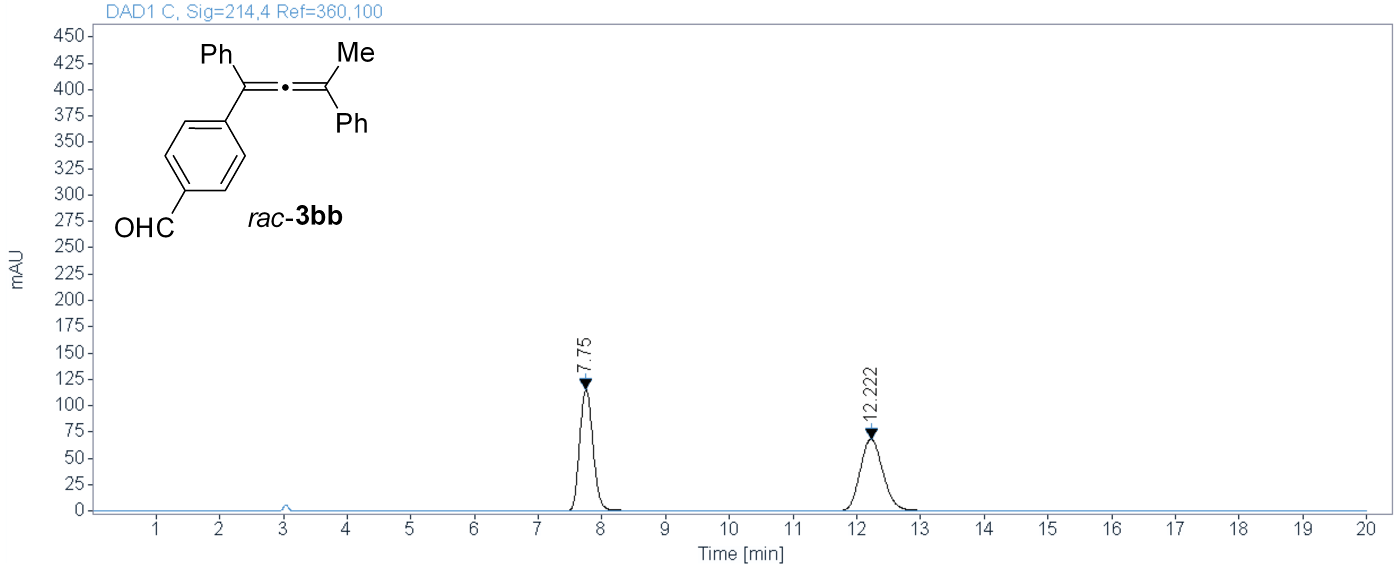

Signal: DAD1 C, Sig=214,4 Ref=360,100

RT [min]	Width [min]	Height	Area	Area%
7.750	0.2324	115.5424	1707.2632	49.9302
12.222	0.3946	68.2623	1712.0386	50.0698
		Sum	3419.3018	100.0000

Supplementary Figure 221. HPLC spectrum for *rac*-**3bb**

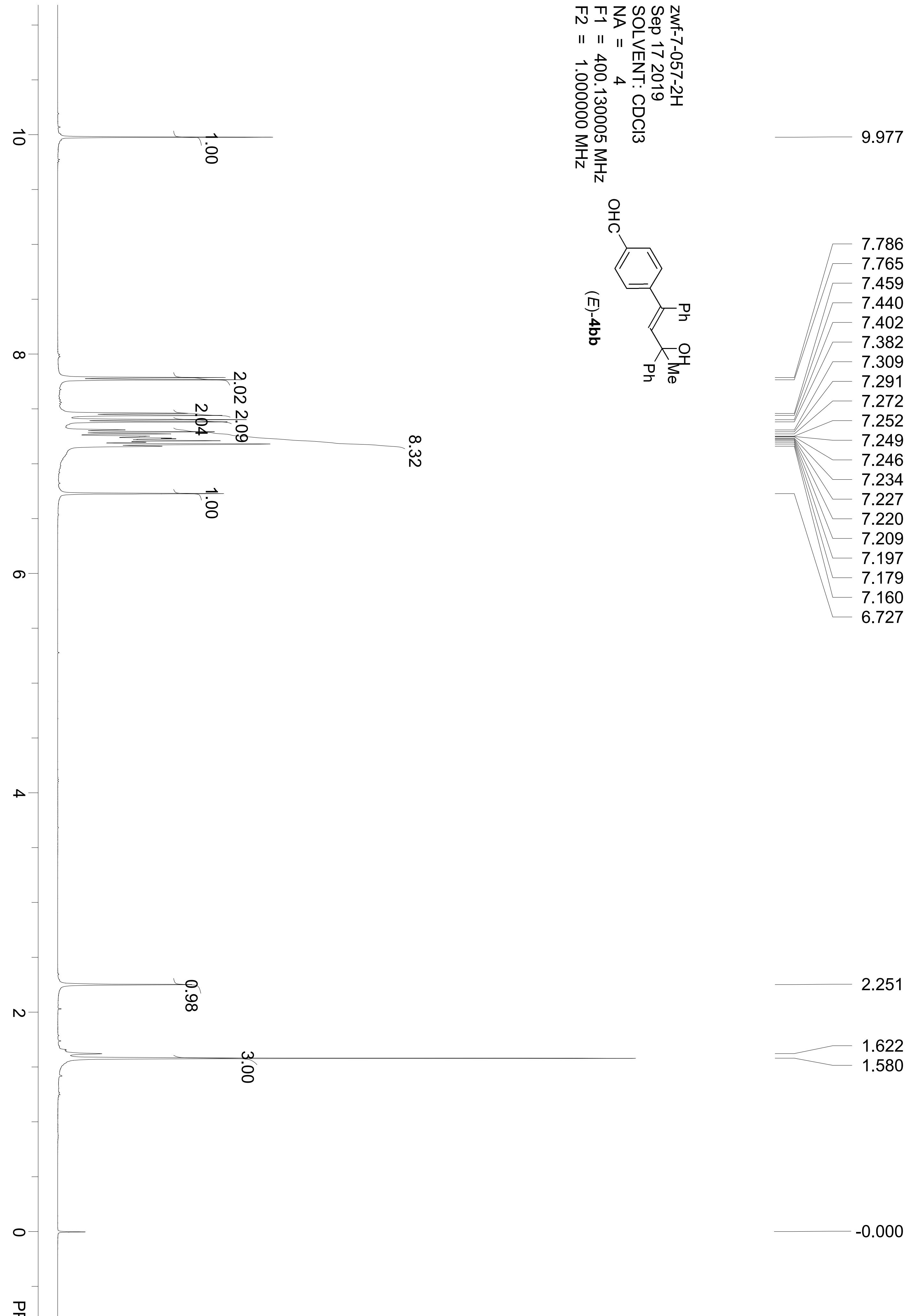

Supplementary Figure 222. ^{1}H NMR (400 MHz, $CDCl_3$) spectrum for (*E*)-**4bb**

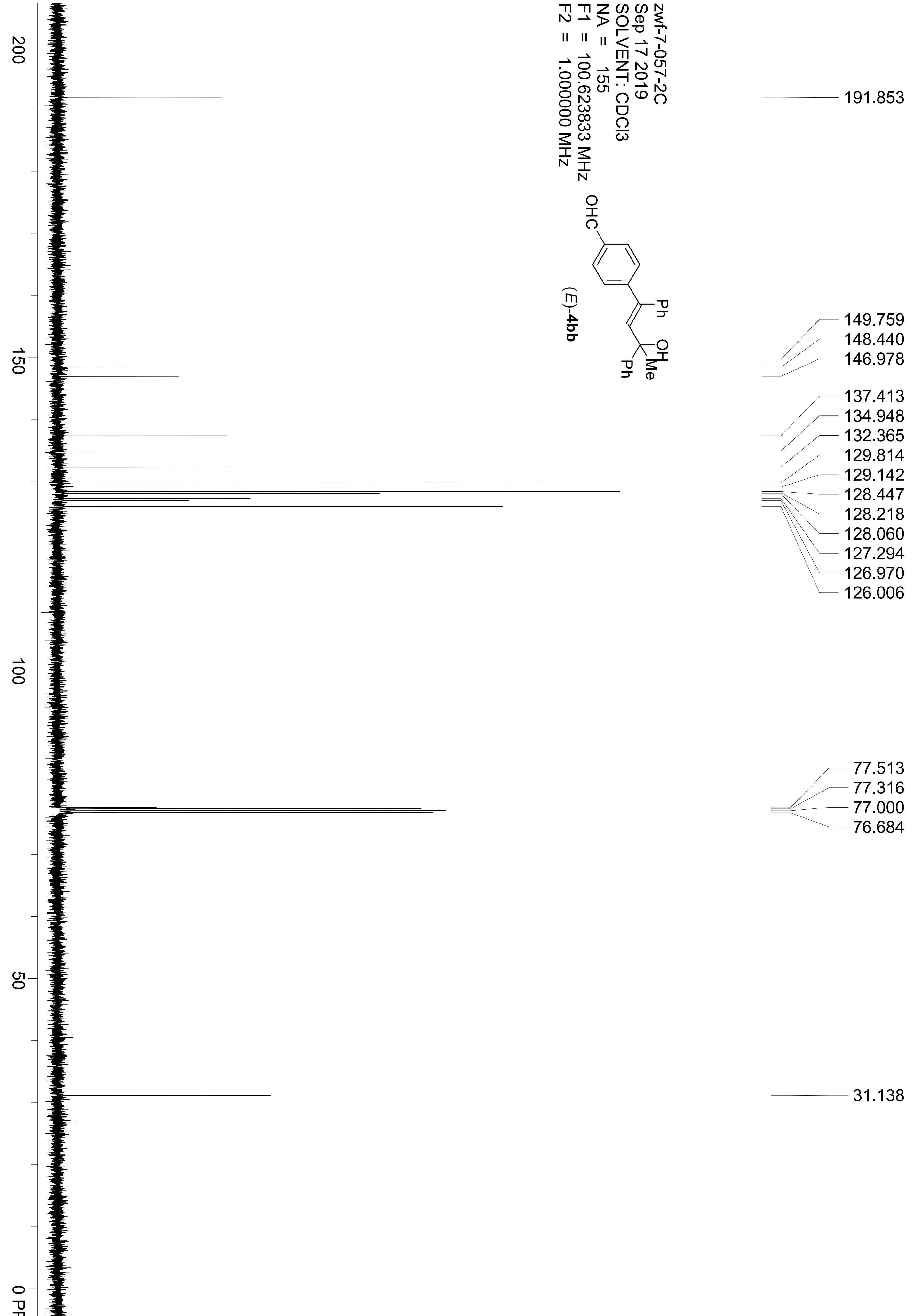

Supplementary Figure 223. ^{13}C NMR (100 MHz, $CDCl_3$) spectrum for (*E*)-**4bb**

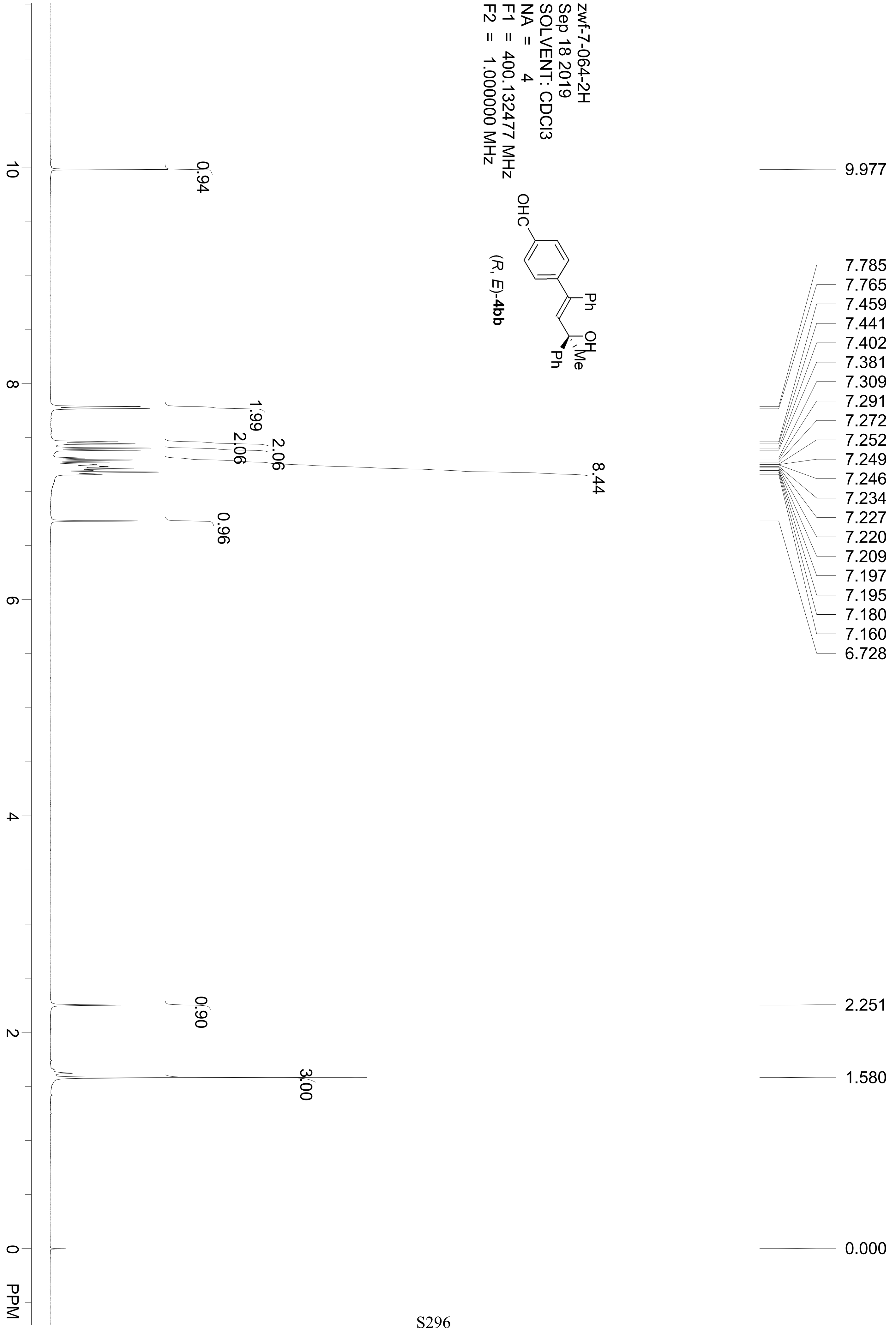

Supplementary Figure 224. ^{1}H NMR (400 MHz, $CDCl_3$) spectrum for (*R*,*E*)-**4bb**

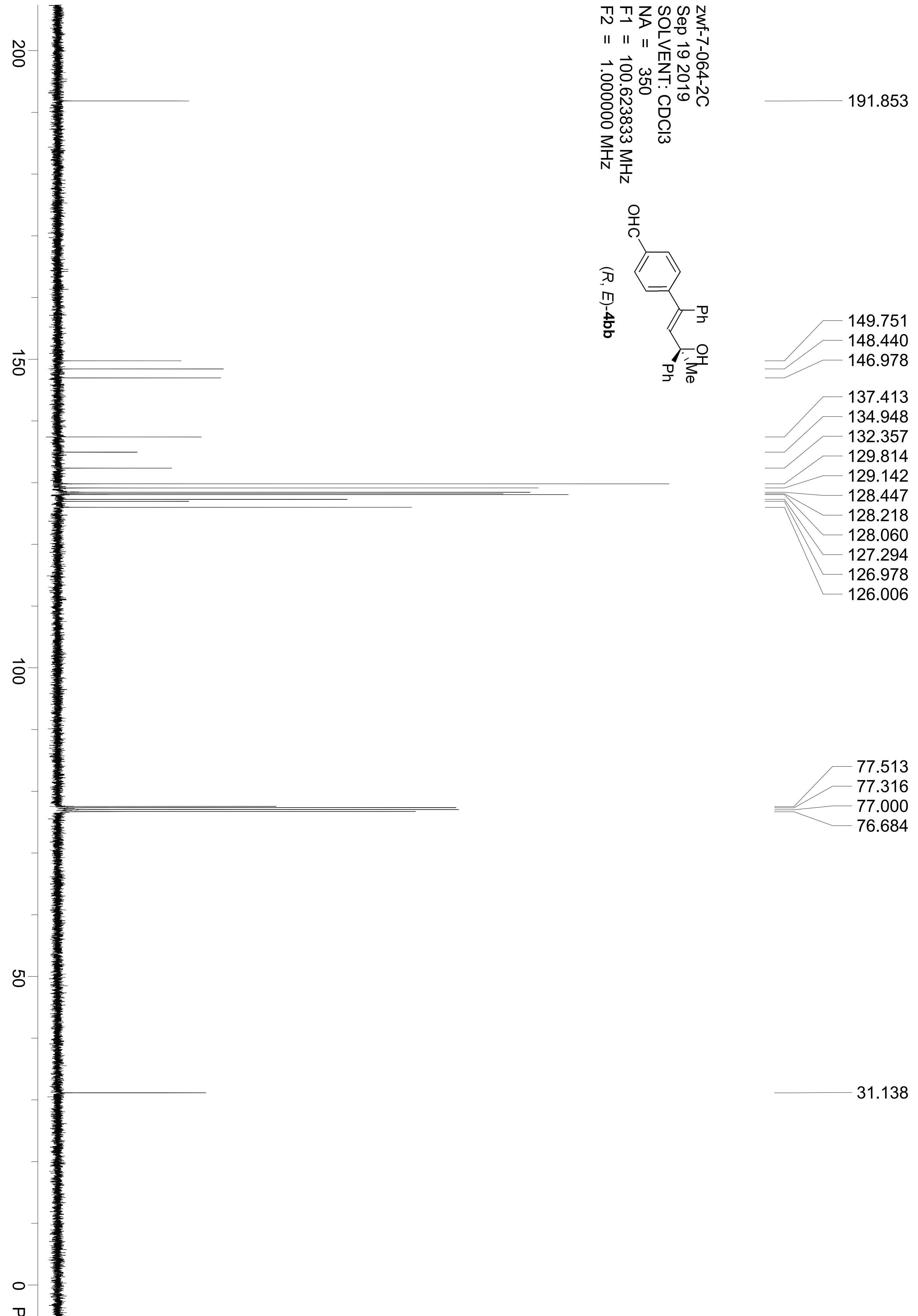

Supplementary Figure 225. ^{13}C NMR (100 MHz, $CDCl_3$) spectrum for (*R*,*E*)-**4bb**

Area Percent Report

Agilent Technologies

sample zwf-7-064-2-AD-H-90-10-1.0-214

Data file: C:\Users\Public\Documents\ChemStation\1\Data\wgl 2019-09-18 08-46-24\023-P1-C4-zwf-7-064-2.D

Acquisition Data:

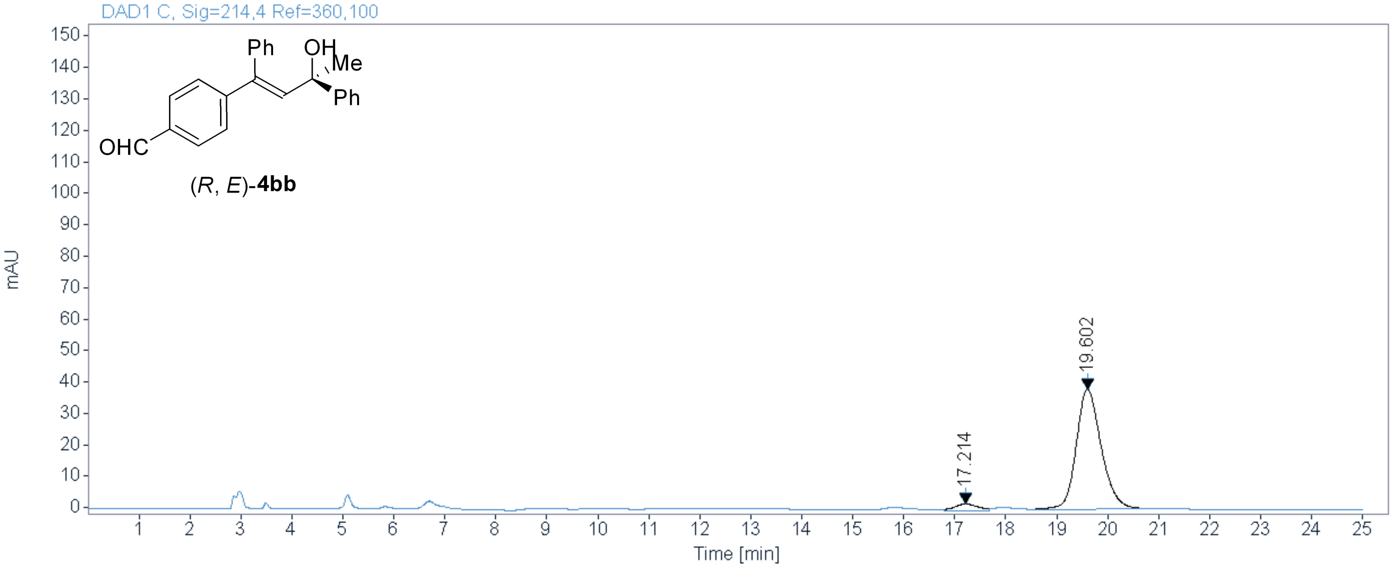

Signal: DAD1 C, Sig=214,4 Ref=360,100

RT [min]	Width [min]	Height	Area	Area%
17.214	0.5306	2.1389	68.1002	4.9959
19.602	0.5211	38.0491	1295.0326	95.0041
		Sum	1363.1328	100.0000

Supplementary Figure 226. HPLC spectrum for (*R*,*E*)-**4bb**

Area Percent Report

sample zwf-7-064-2-rac-AD-H-90-10-1.0-214

Data file: C:\Users\Public\Documents\ChemStation\1\Data\wgl 2019-09-18 08-46-24\022-P1-C3-zwf-7-064-2-rac.D

Acquisition Data:

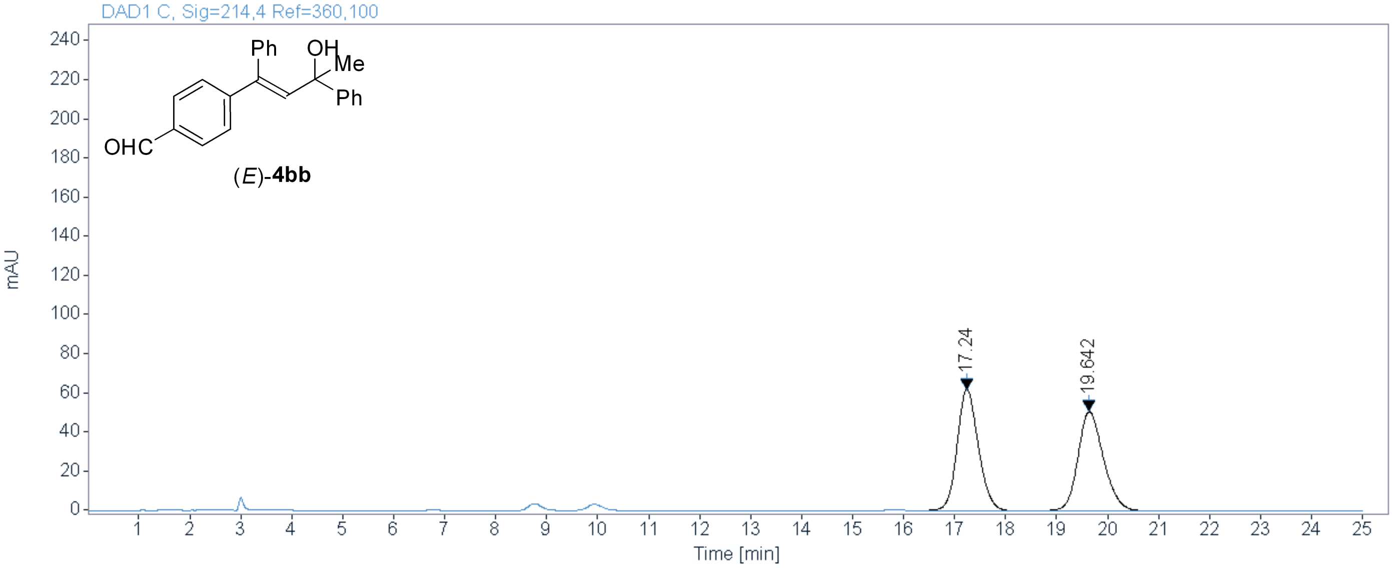

Signal: DAD1 C, Sig=214,4 Ref=360,100

RT [min]	Width [min]	Height	Area	Area%
17.240	0.4446	62.0650	1801.0583	50.2978
19.642	0.5369	50.7800	1779.7345	49.7022
		Sum	3580.7928	100.0000

Supplementary Figure 227. HPLC spectrum for (*E*)-**4bb**

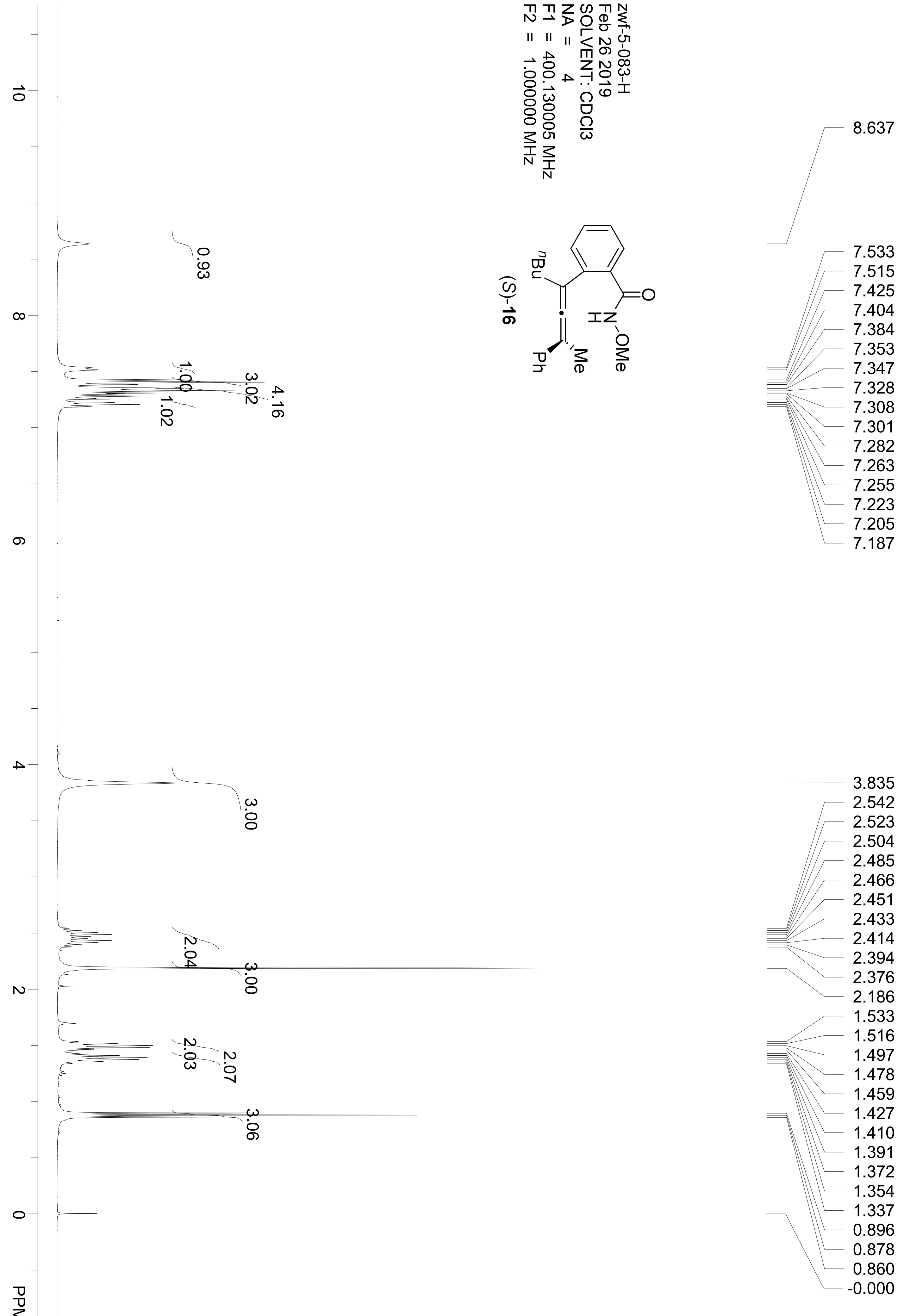

Supplementary Figure 228. ^{1}H NMR (400 MHz, $CDCl_3$) spectrum for (*S*)-**16**

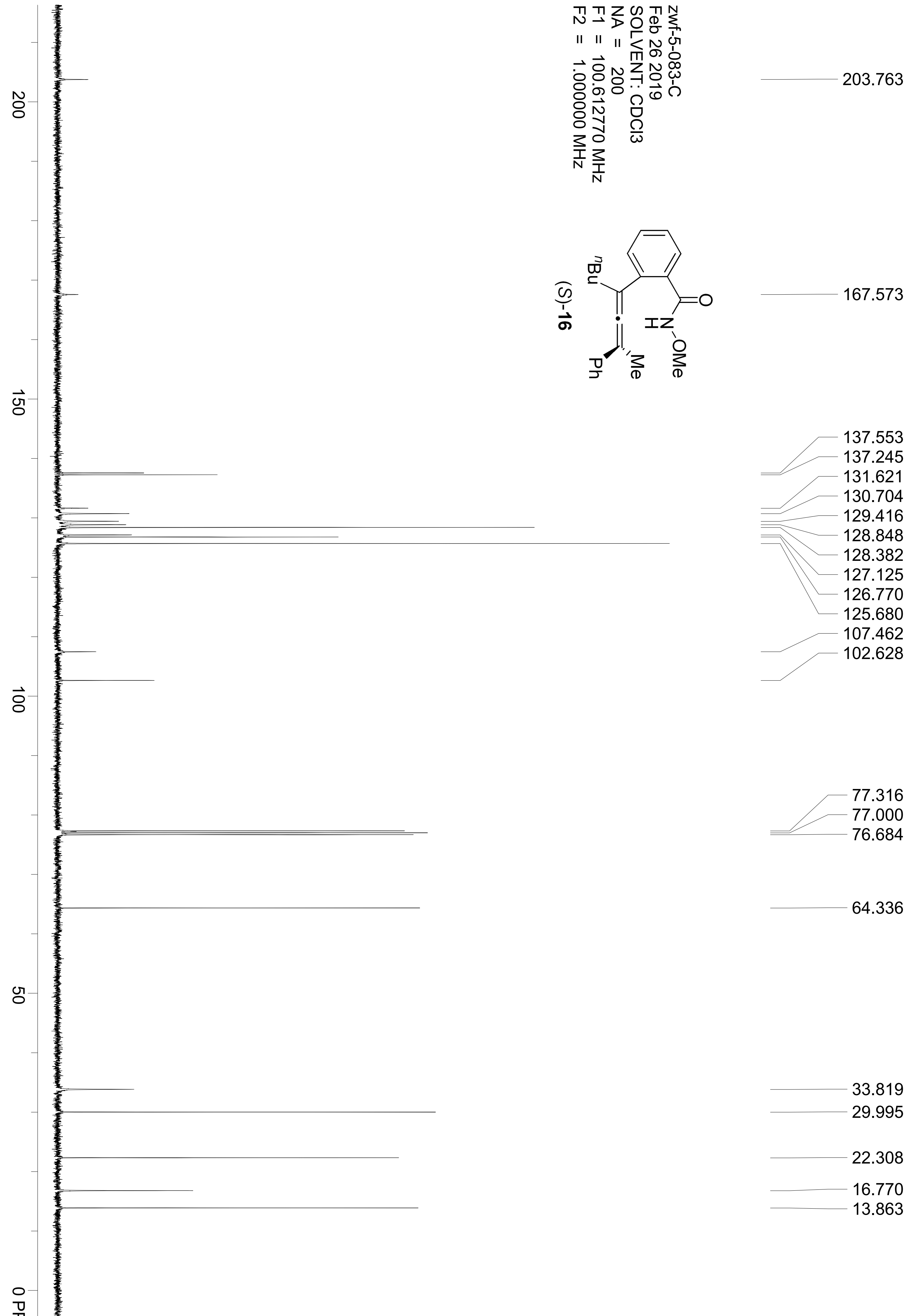

Supplementary Figure 229. ^{13}C NMR (100 MHz, $CDCl_3$) spectrum for (*S*)-**16**

Area Percent Report

Agilent Technologies

Sample name: zwf-5-083-IC-90-10-1.0-214

Data file: C:\Users\Public\Documents\ChemStation\1\Data\zwf-allenioc acid_LC 2019-02-25 20-20-08\002-P1-C1-zwf-5-083.D

Acquisition Data: 2/25/2019 8:38:07 PM

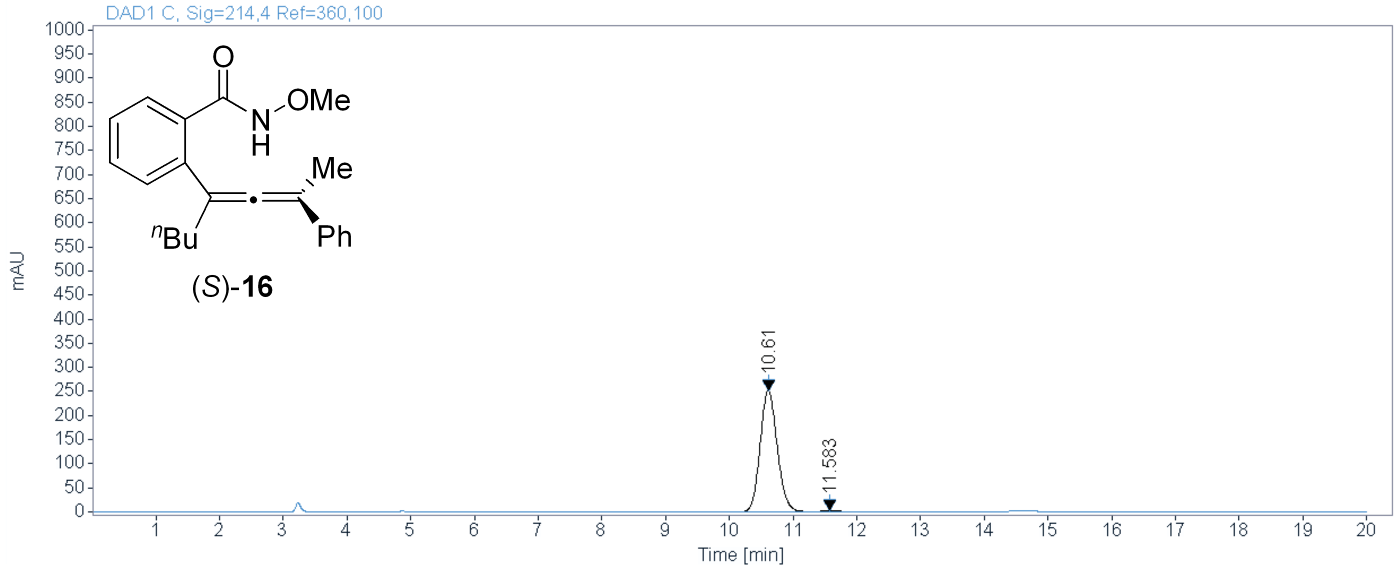

Signal: DAD1 C, Sig=214,4 Ref=360,100

RT [min]	Width [min]	Height	Area	Area%
10.610	0.2863	251.5373	4662.4648	99.4329
11.583	0.3070	1.3544	26.5898	0.5671
		Sum	4689.0546	100.0000

Supplementary Figure 230. HPLC spectrum for (*S*)-**16**

Area Percent Report

Agilent Technologies

Sample name: zwf-5-082-rac-IC-90-10-1.0-214

Data file: C:\Users\Public\Documents\ChemStation\1\Data\zwf-allenioc acid_LC 2019-02-25 20-20-08\003-P1-C2-zwf-5-082-rac.D

Acquisition Data: 2/25/2019 8:58:55 PM

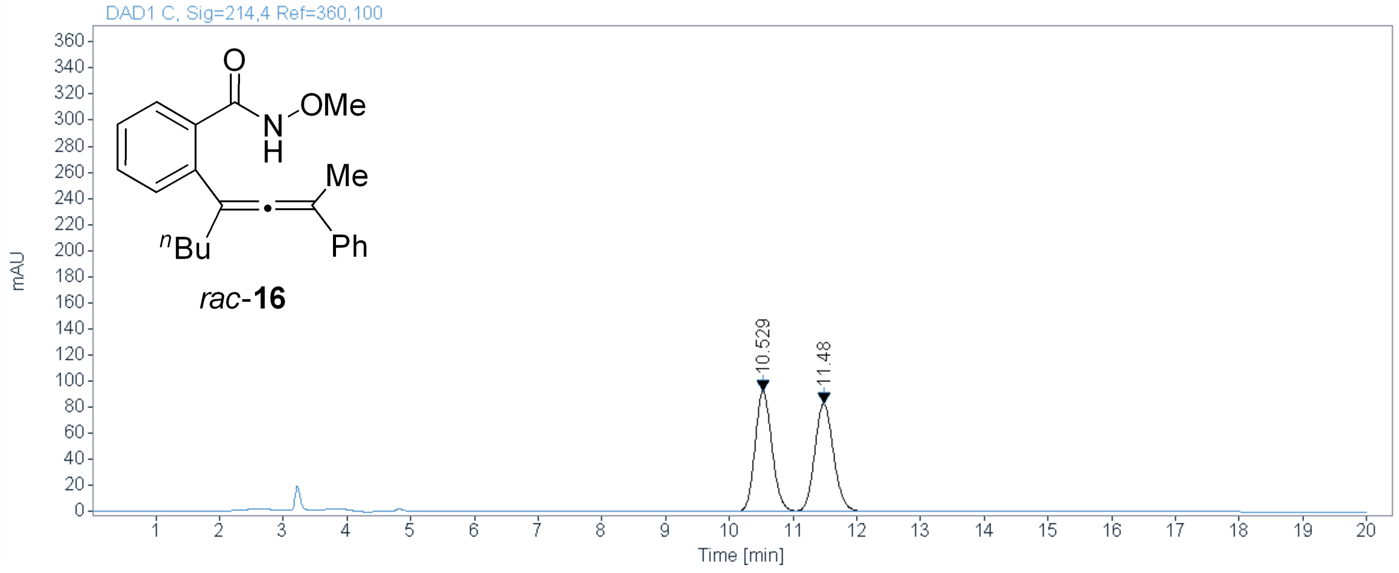

Signal: DAD1 C, Sig=214,4 Ref=360,100

RT [min]	Width [min]	Height	Area	Area%
10.529	0.2838	92.4369	1694.3567	50.0224
11.480	0.3168	82.6899	1692.8389	49.9776
		Sum	3387.1956	100.0000

Supplementary Figure 231. HPLC spectrum for *rac*-**16**

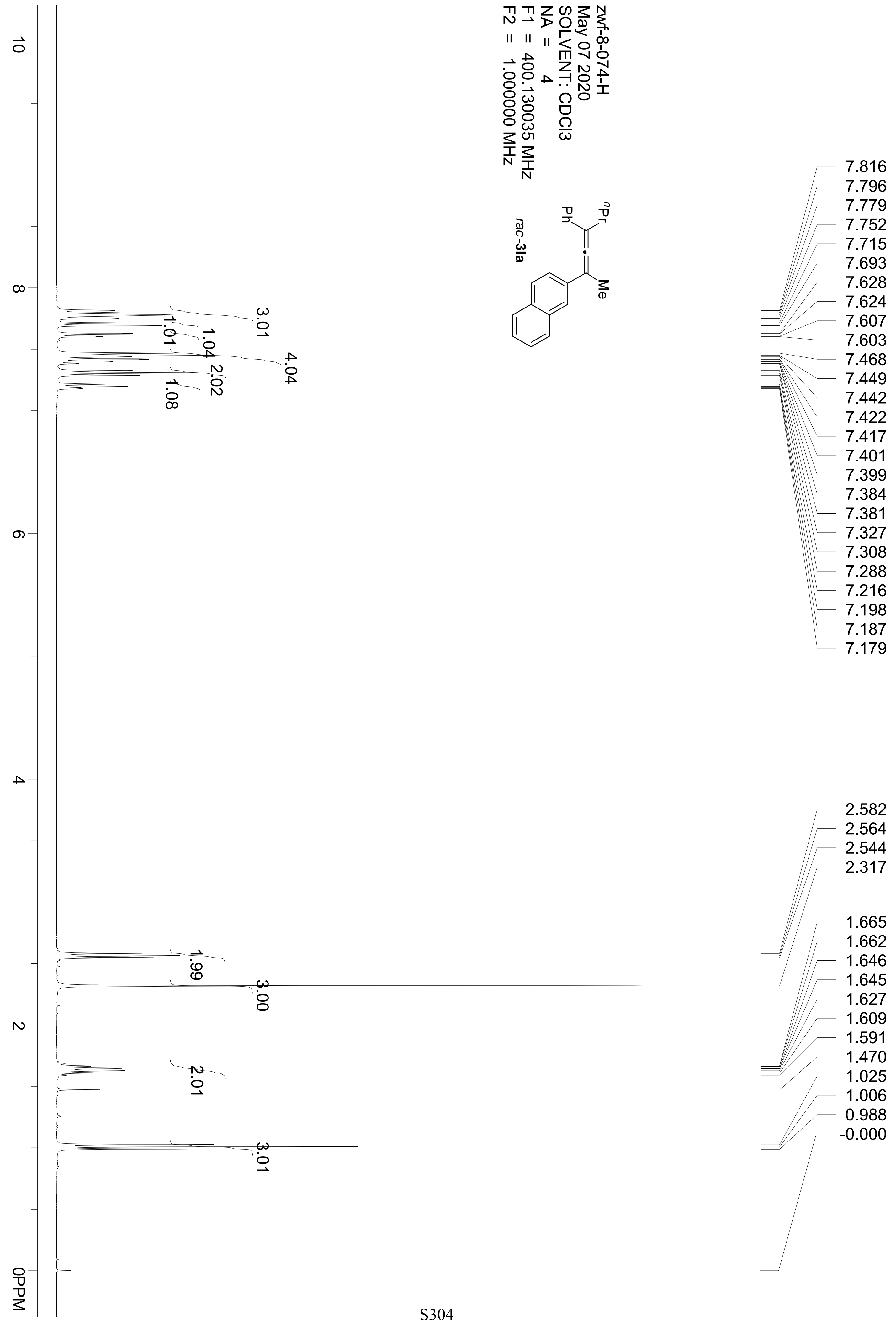

Supplementary Figure 232. ^{1}H NMR (400 MHz, $CDCl_3$) spectrum for *rac*-**3la**

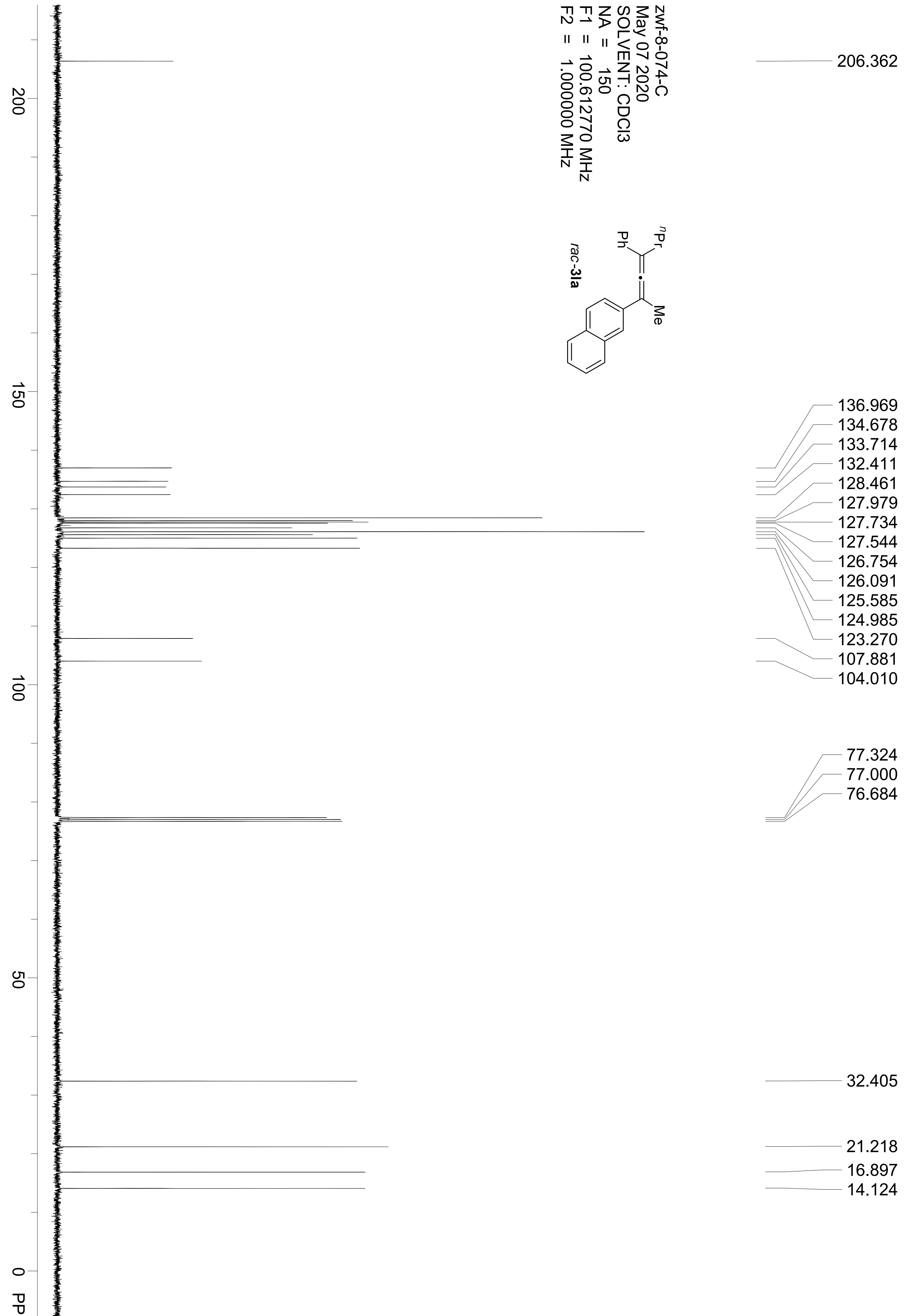

Supplementary Figure 233. ¹³C NMR (100 MHz, $CDCl_3$) spectrum for *rac*-**3la**

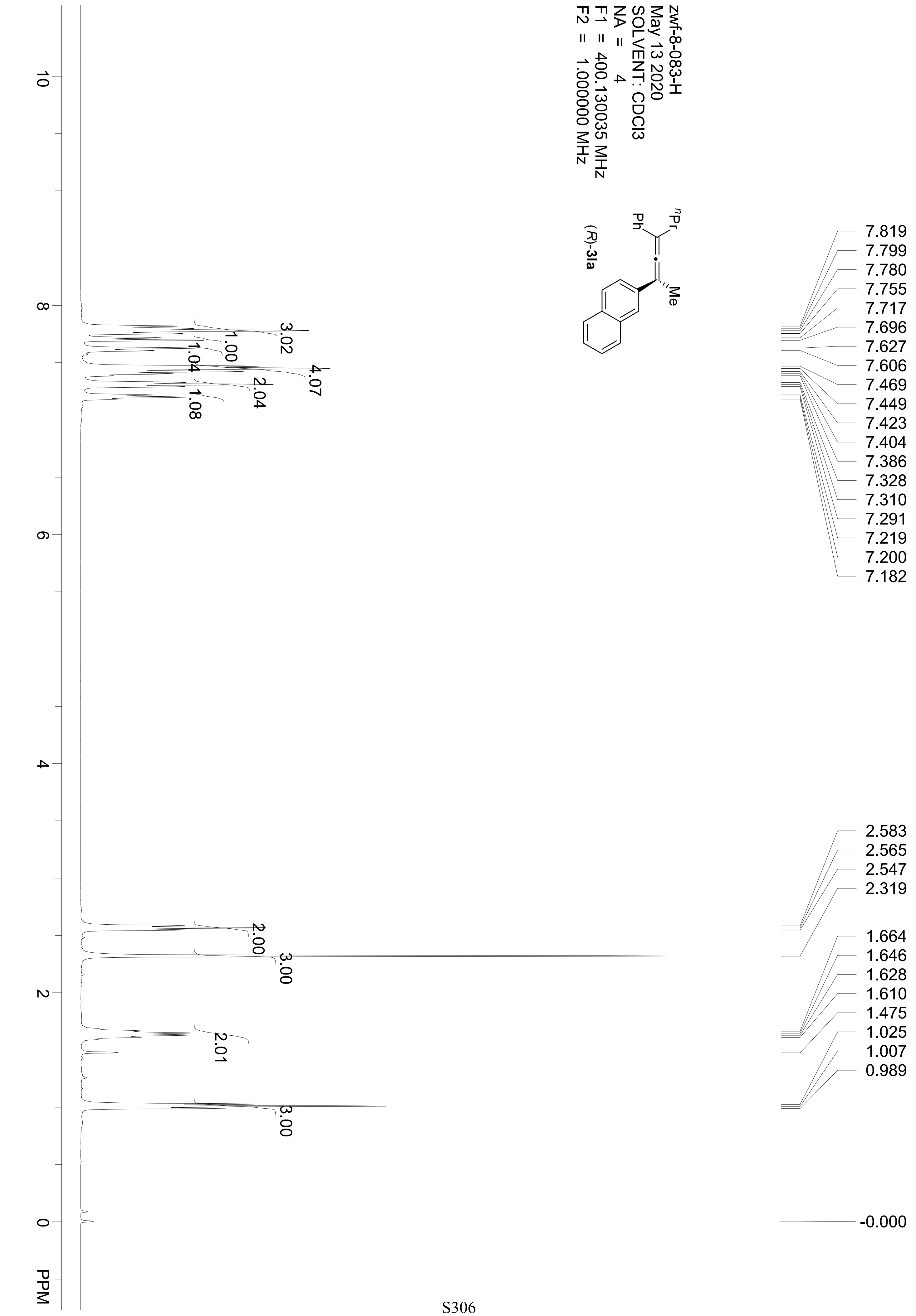

Supplementary Figure 234. ^{1}H NMR (400 MHz, $CDCl_3$) spectrum for (*R*)-**3la**

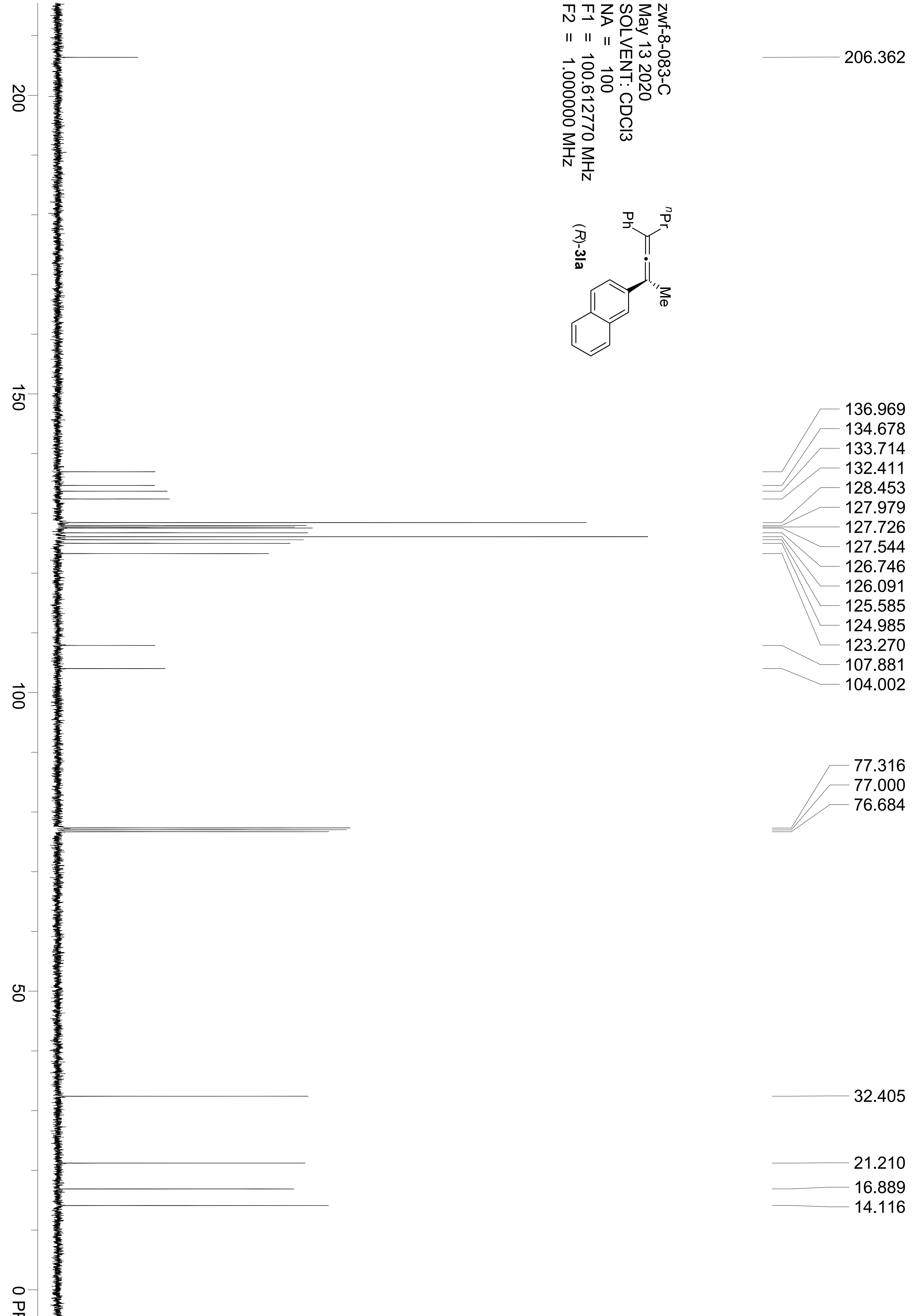

Supplementary Figure 235. ^{13}C NMR (100 MHz, $CDCl_3$) spectrum for (*R*)-**3la**

Area Percent Report

Agilent Technologies

sample zwf-8-083-AD-H-99.5-0.5-0.5-214

Data file: C:\Users\Public\Documents\ChemStation\1\Data\zwf-allenioc acid_LC 2020-05-13 19-54-53\003-P1-C1-zwf-7-083.D

Acquisition Data:

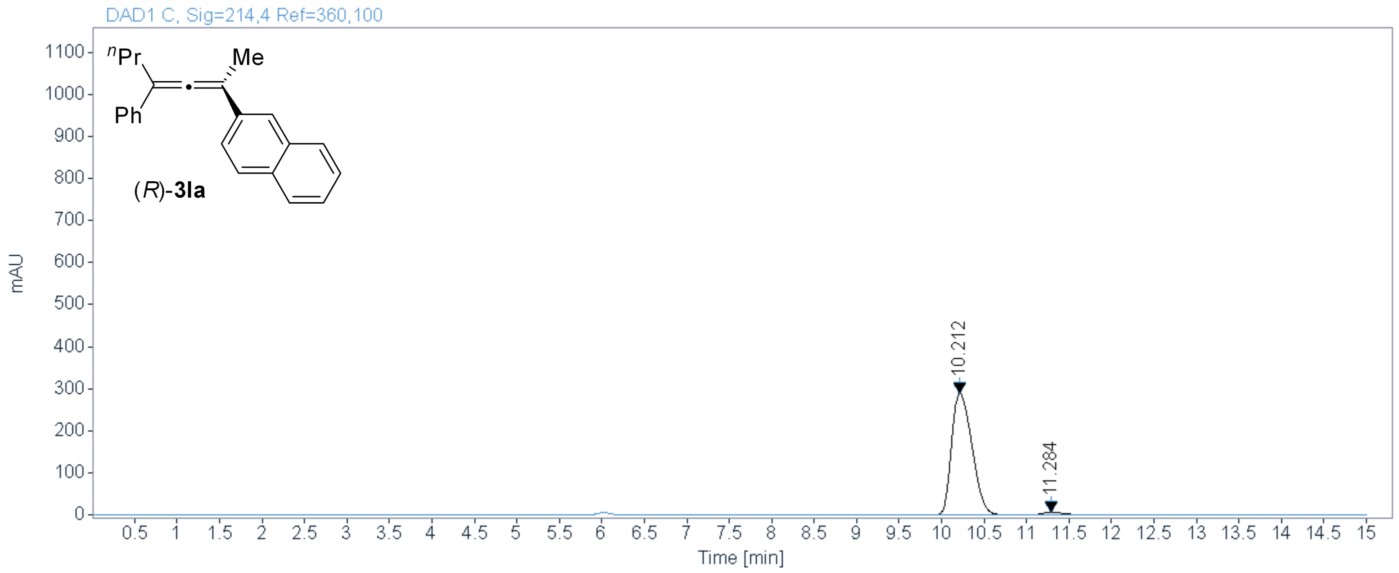

Signal: DAD1 C, Sig=214,4 Ref=360,100

RT [min]	Width [min]	Height	Area	Area%
10.212	0.2630	289.8290	4743.7715	97.8153
11.284	0.2924	6.0401	105.9501	2.1847
		Sum	4849.7216	100.0000

Supplementary Figure 236. HPLC spectrum for (*R*)-**3la**

Area Percent Report

Agilent Technologies

sample zwf-8-083-rac-AD-H-99.5-0.5-0.5-214

Data file: C:\Users\Public\Documents\ChemStation\1\Data\zwf-allenioc acid_LC 2020-05-13 19-54-53\002-P1-C3-zwf-7-083-raac.D

Acquisition Data:

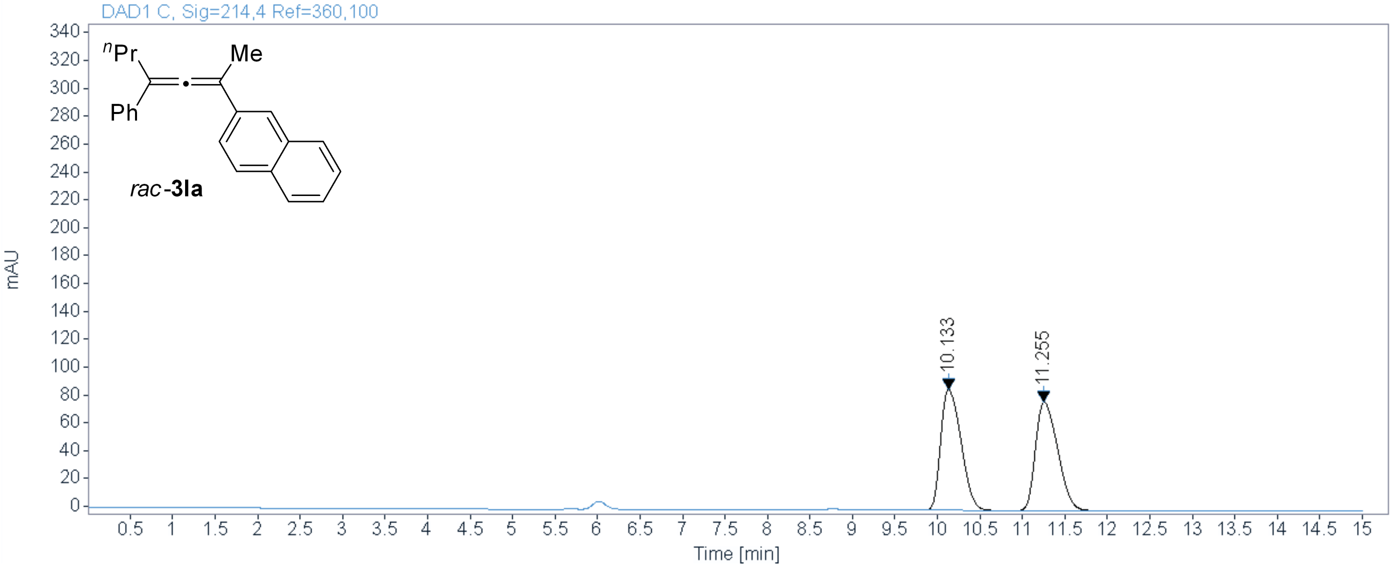

Signal: DAD1 C, Sig=214,4 Ref=360,100

RT [min]	Width [min]	Height	Area	Area%
10.133	0.2642	86.8181	1415.0702	49.8691
11.255	0.2927	78.0120	1422.4963	50.1309
		Sum	2837.5665	100.0000

Supplementary Figure 237. HPLC spectrum for *rac*-**3la**

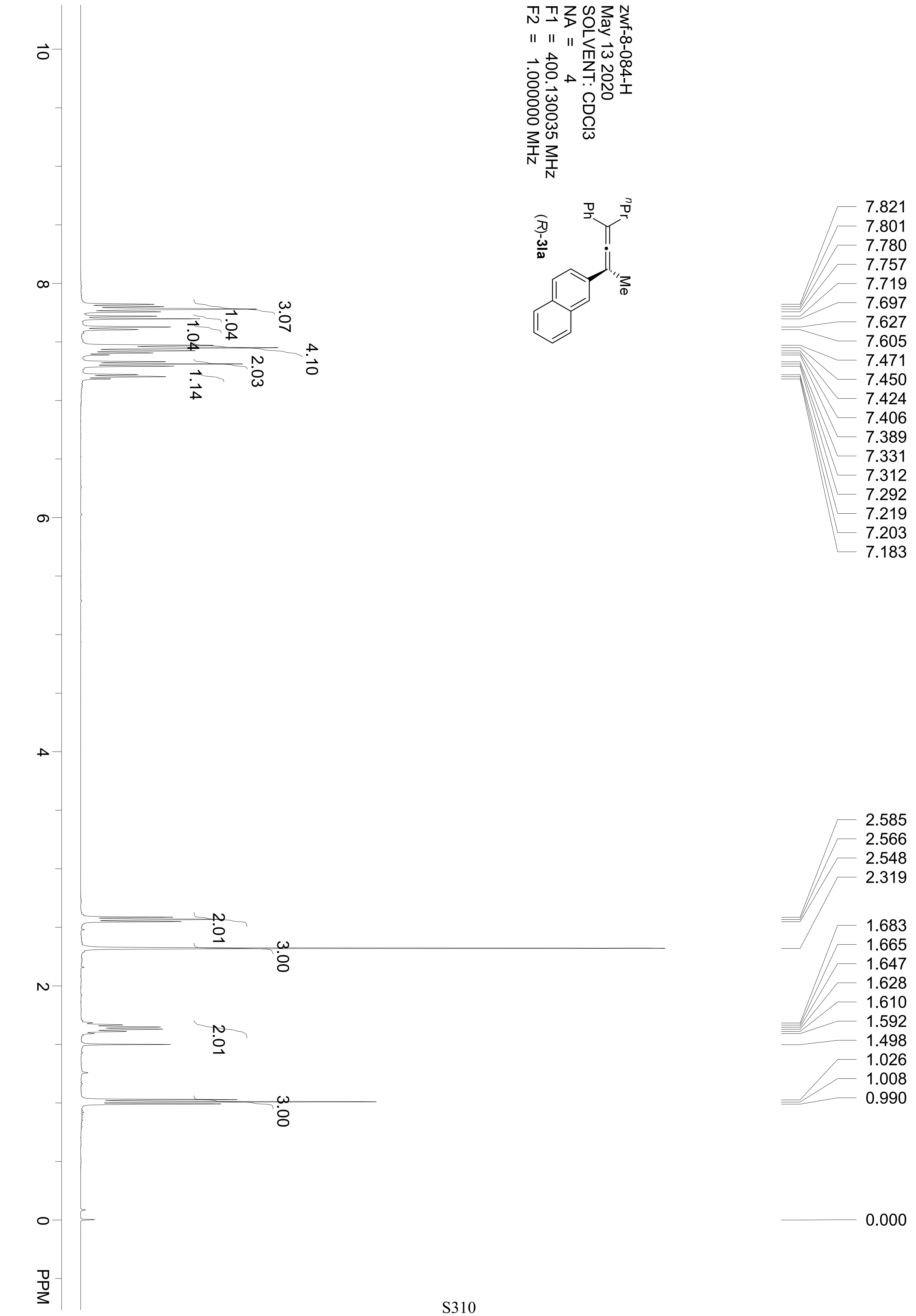

Supplementary Figure 238. ^{1}H NMR (400 MHz, $CDCl_3$) spectrum for (*R*)-**3la**

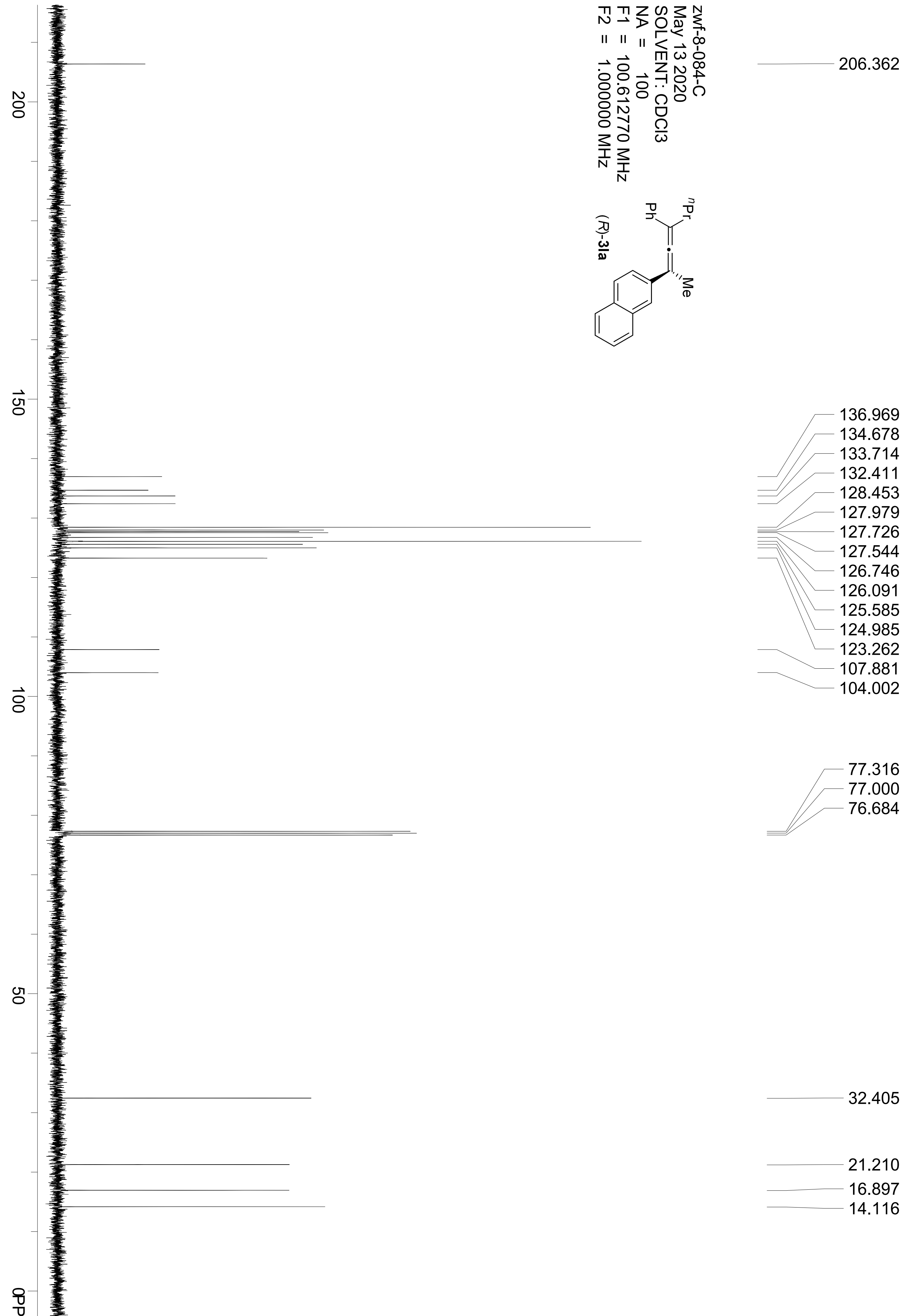

Supplementary Figure 239. ^{13}C NMR (100 MHz, $CDCl_3$) spectrum for (*R*)-**3la**

Area Percent Report

Agilent Technologies

sample: zwf-8-084-AD-H-99.5-0.5-0.5-214

Data file: C:\Users\Public\Documents\ChemStation\1\Data\zwf-allenioc acid_LC 2020-05-13 19-54-53\005-P1-C2-zwf-7-084.D

Acquisition Data:

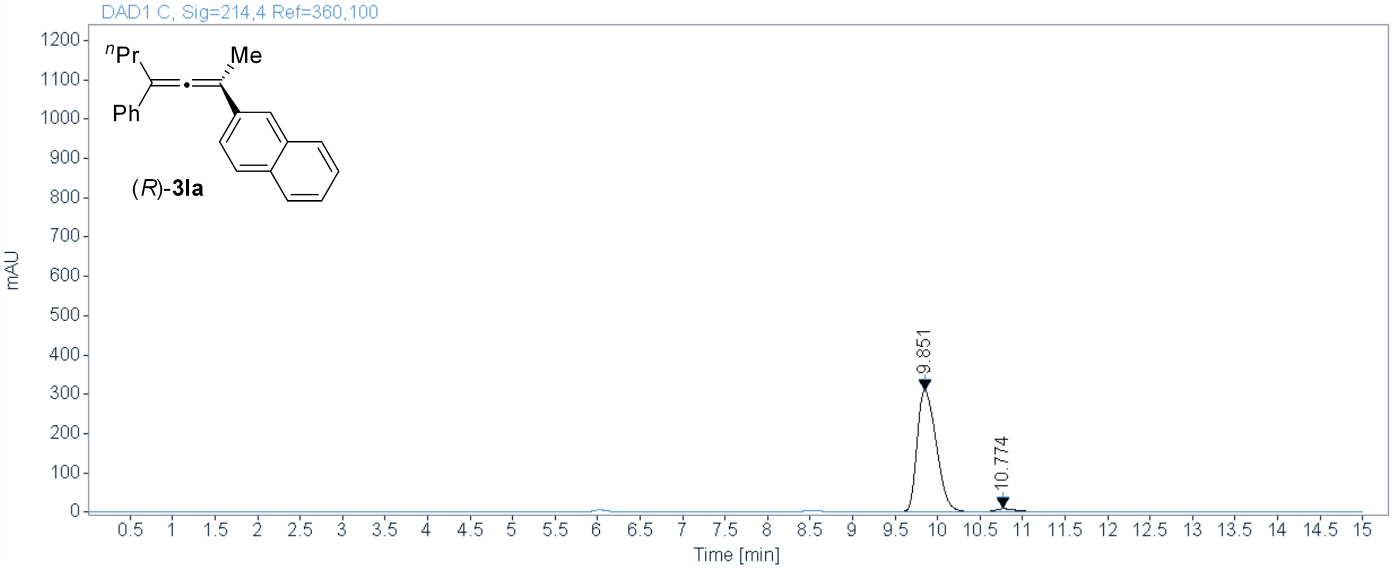

Signal: DAD1 C, Sig=214,4 Ref=360,100

RT [min]	Width [min]	Height	Area	Area%
9.851	0.2530	309.4717	4855.1455	97.5529
10.774	0.2754	7.3692	121.7881	2.4471
		Sum	4976.9336	100.0000

Supplementary Figure 240. HPLC spectrum for (*R*)-**3la**

Area Percent Report

Agilent Technologies

sample zwf-8-084-rac-AD-H-99.5-0.5-0.5-214

Data file: C:\Users\Public\Documents\ChemStation\1\Data\zwf-allenioc acid_LC 2020-05-13 19-54-53\006-P1-C3-zwf-7-083-raac.D

Acquisition Data:

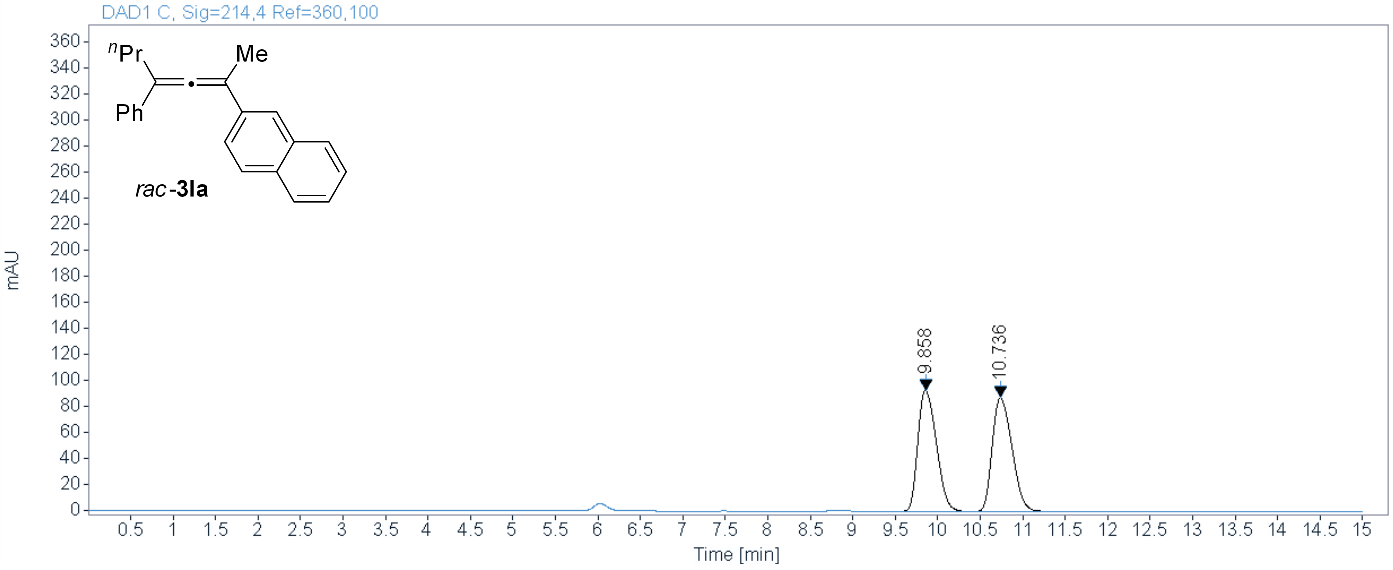

Signal: DAD1 C, Sig=214,4 Ref=360,100

RT [min]	Width [min]	Height	Area	Area%
9.858	0.2454	93.1981	1416.9442	49.9890
10.736	0.2592	87.4170	1417.5688	50.0110
		Sum	2834.5131	100.0000

Supplementary Figure 241. HPLC spectrum for *rac*-**3la**

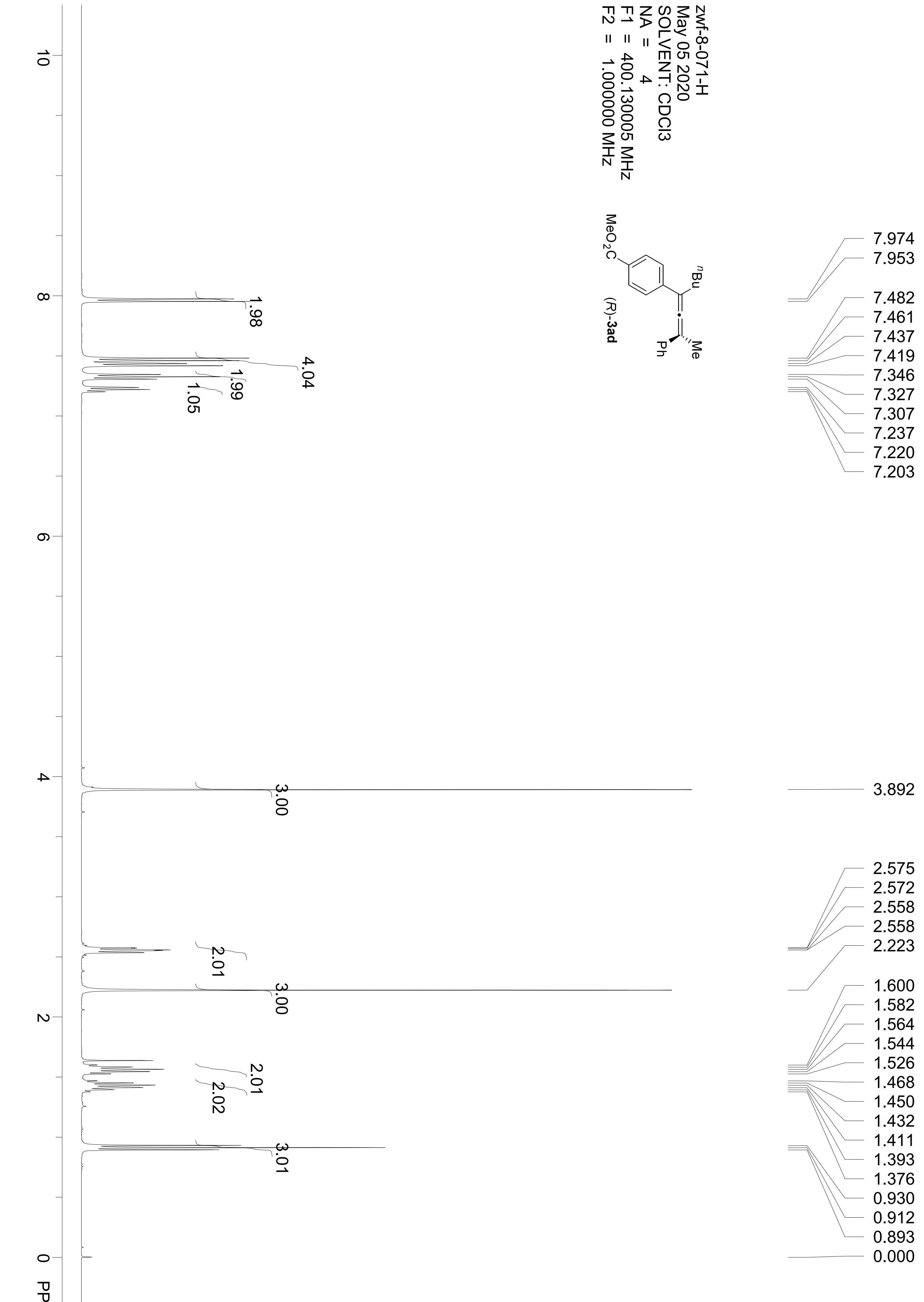

Supplementary Figure 242. ^{1}H NMR (400 MHz, $CDCl_3$) spectrum for (*R*)-**3ad**

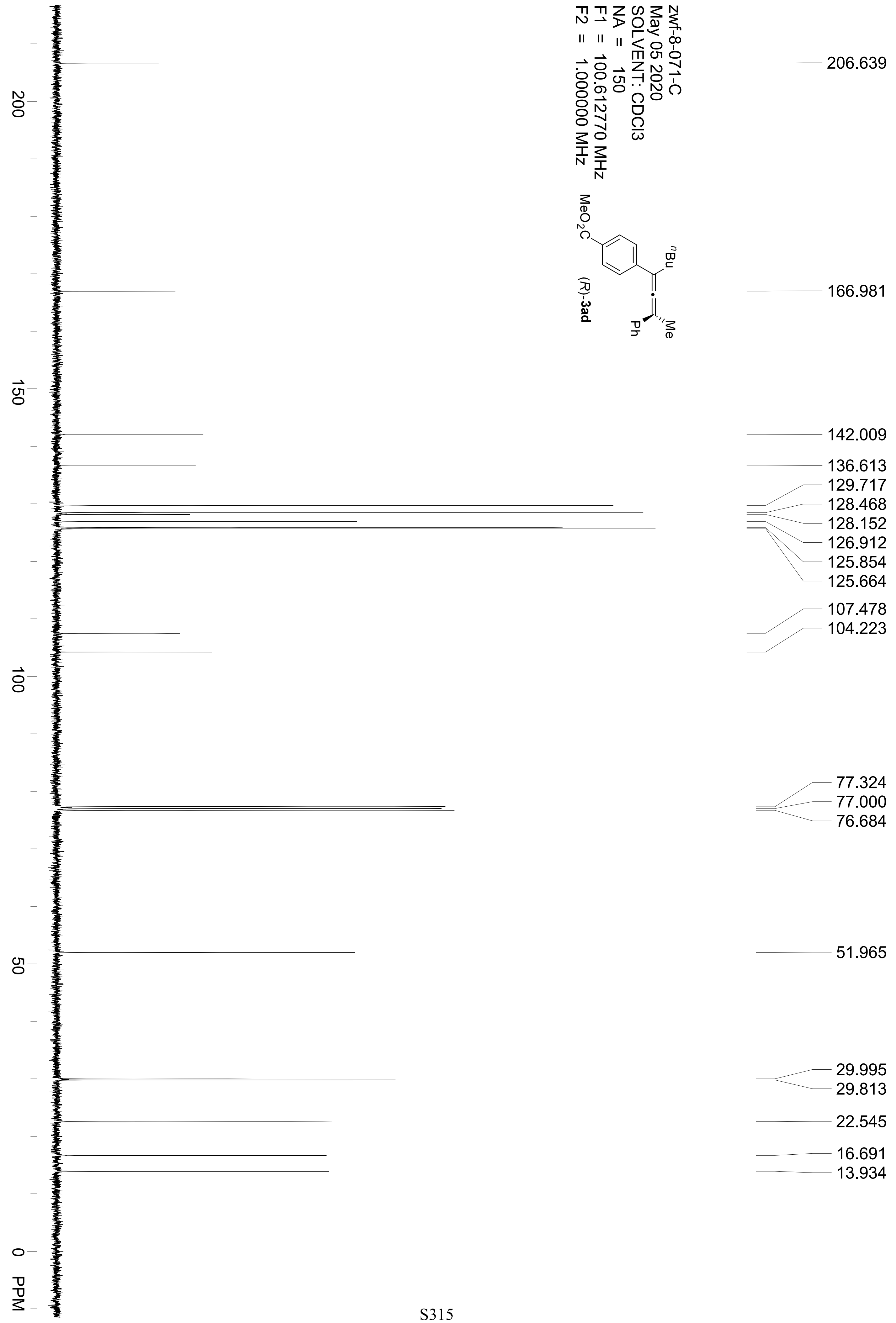

Supplementary Figure 243. ^{13}C NMR (100 MHz, $CDCl_3$) spectrum for (*R*)-**3ad**

Area Percent Report

sample zwf-8-071-OD-H-99.5-0.5-1.0-214

Data file: C:\Users\Public\Documents\ChemStation\1\Data\zwf-allenioc acid_LC 2020-05-04 21-20-37\002-P1-C1-zwf-8-071.D

Acquisition Data:

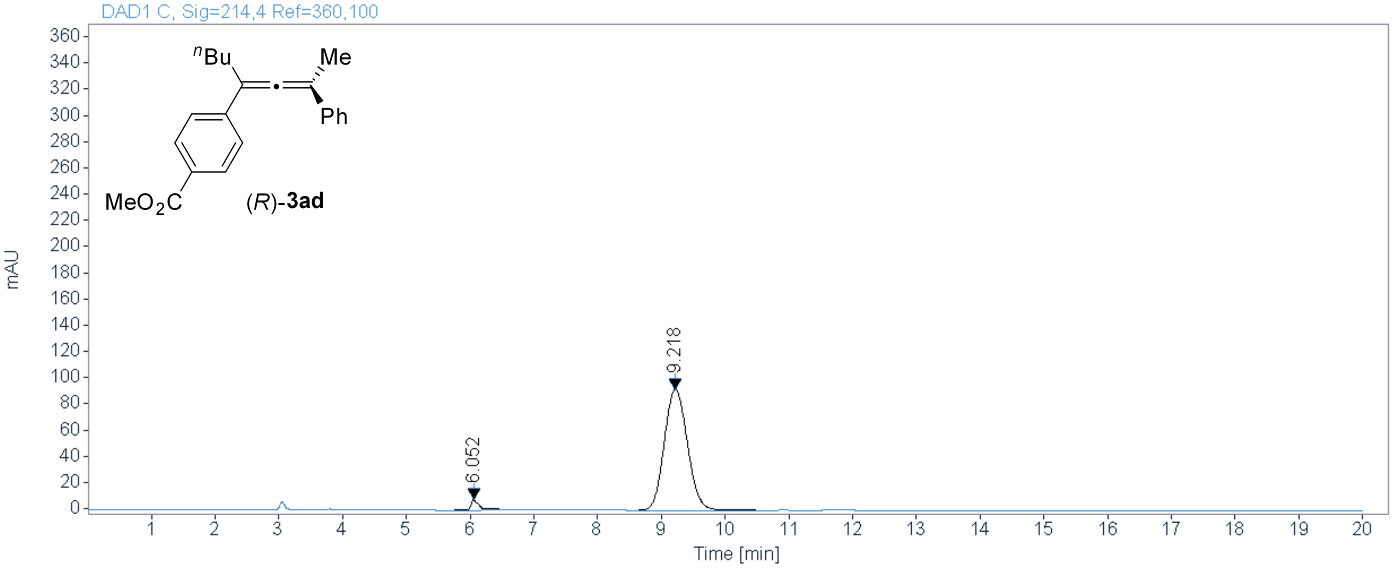

Signal: DAD1 C, Sig=214,4 Ref=360,100

RT [min]	Width [min]	Height	Area	Area%
6.052	0.1668	8.1531	81.6138	3.3656
9.218	0.4003	92.2621	2343.3418	96.6344
		Sum	2424.9556	100.0000

Supplementary Figure 244. HPLC spectrum for (*R*)-**3ad**

Area Percent Report

Agilent Technologies

sample zwf-8-072-rac-OD-H-99.5-0.5-1.0-214

Data file: C:\Users\Public\Documents\ChemStation\1\Data\zwf-allenioc acid_LC 2020-05-04 21-20-37\005-P1-C3-zwf-8-072-rac.D

Acquisition Data:

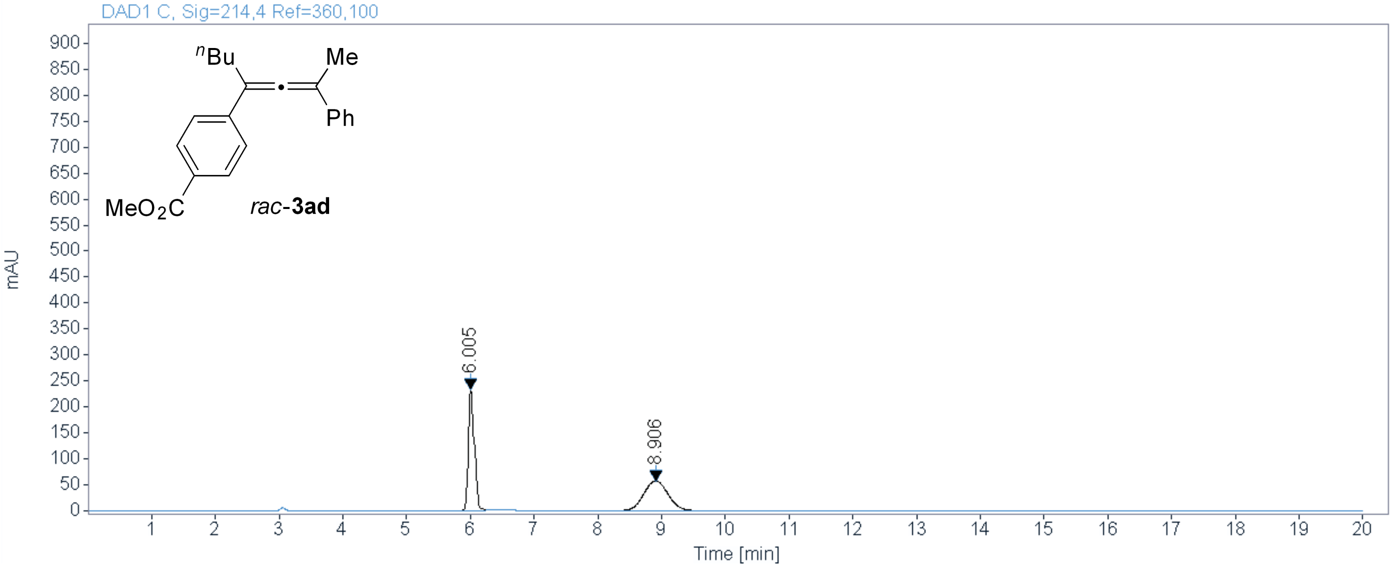

Signal: DAD1 C, Sig=214,4 Ref=360,100

RT [min]	Width [min]	Height	Area	Area%
6.005	0.1102	234.1500	1547.5454	50.2223
8.906	0.4193	57.4855	1533.8444	49.7777
		Sum	3081.3898	100.0000

Supplementary Figure 245. HPLC spectrum for *rac-3ad*

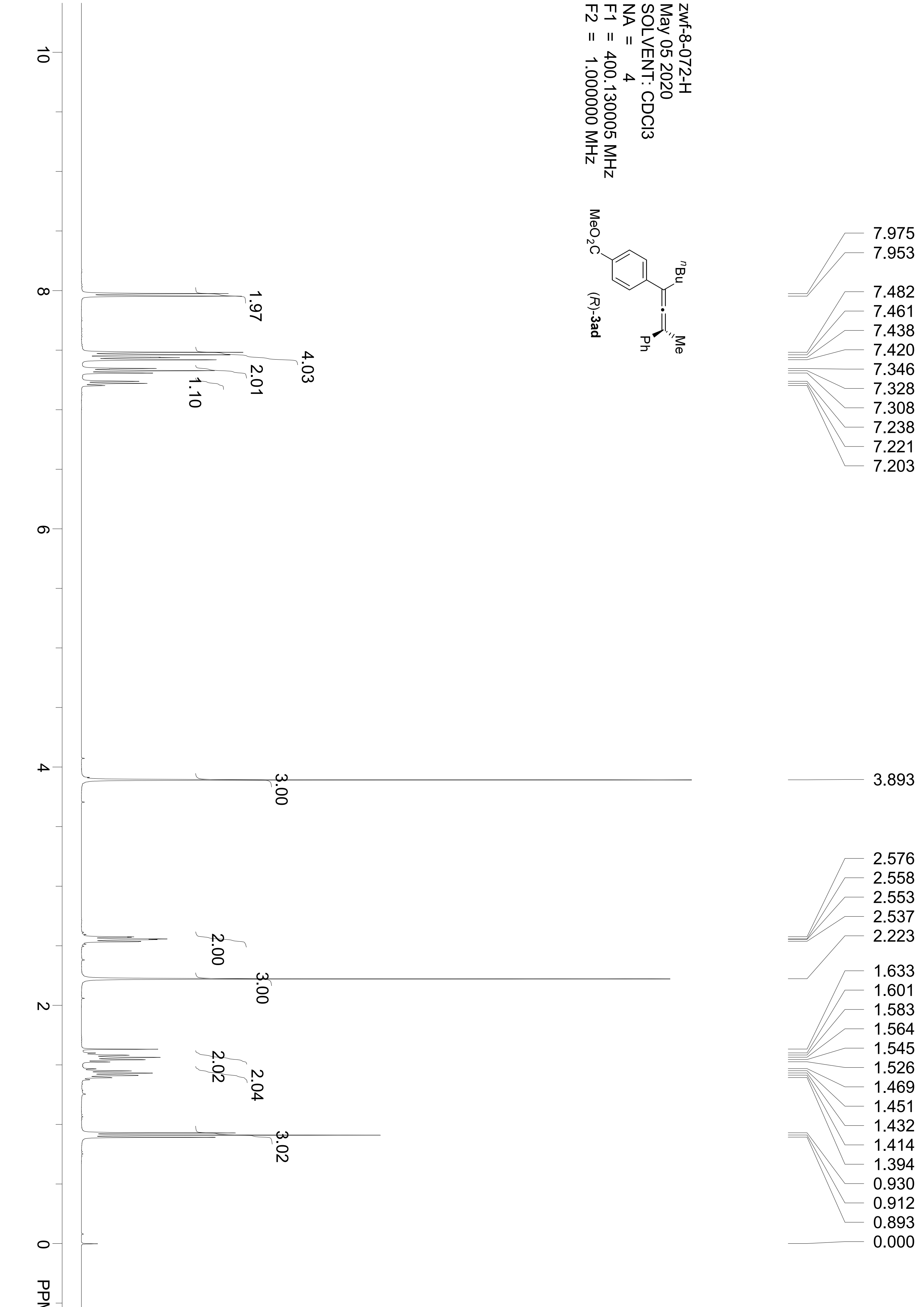

Supplementary Figure 246. ^{1}H NMR (400 MHz, $CDCl_3$) spectrum for (*R*)-**3ad**

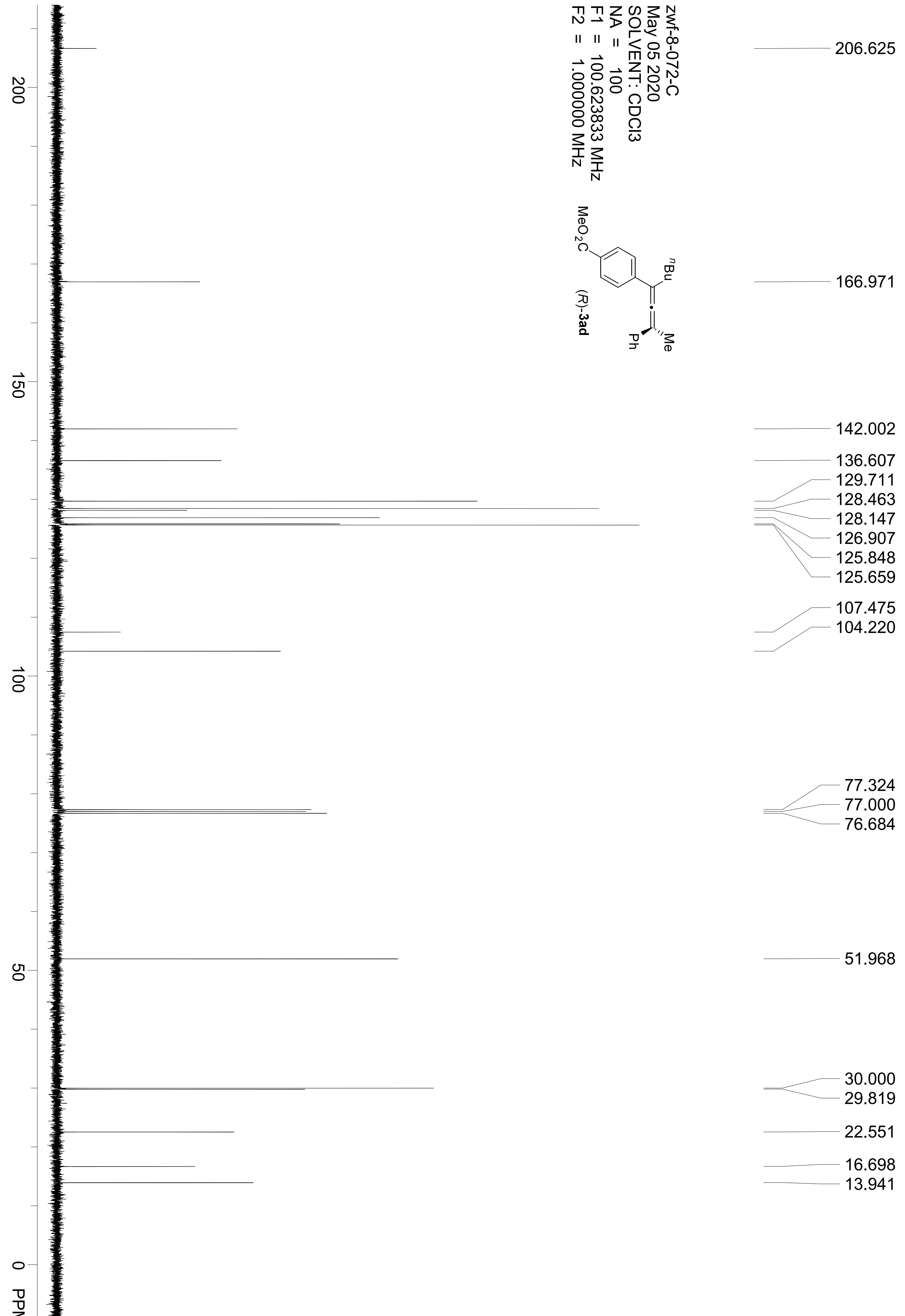

Supplementary Figure 247. ^{13}C NMR (100 MHz, $CDCl_3$) spectrum for (*R*)-**3ad**

Area Percent Report

sample zwf-8-072-OD-H-99.5-0.5-1.0-214

Data file: C:\Users\Public\Documents\ChemStation\1\Data\zwf-allenioc acid_LC 2020-05-04 21-20-37\004-P1-C2-zwf-8-072.D

Acquisition Data:

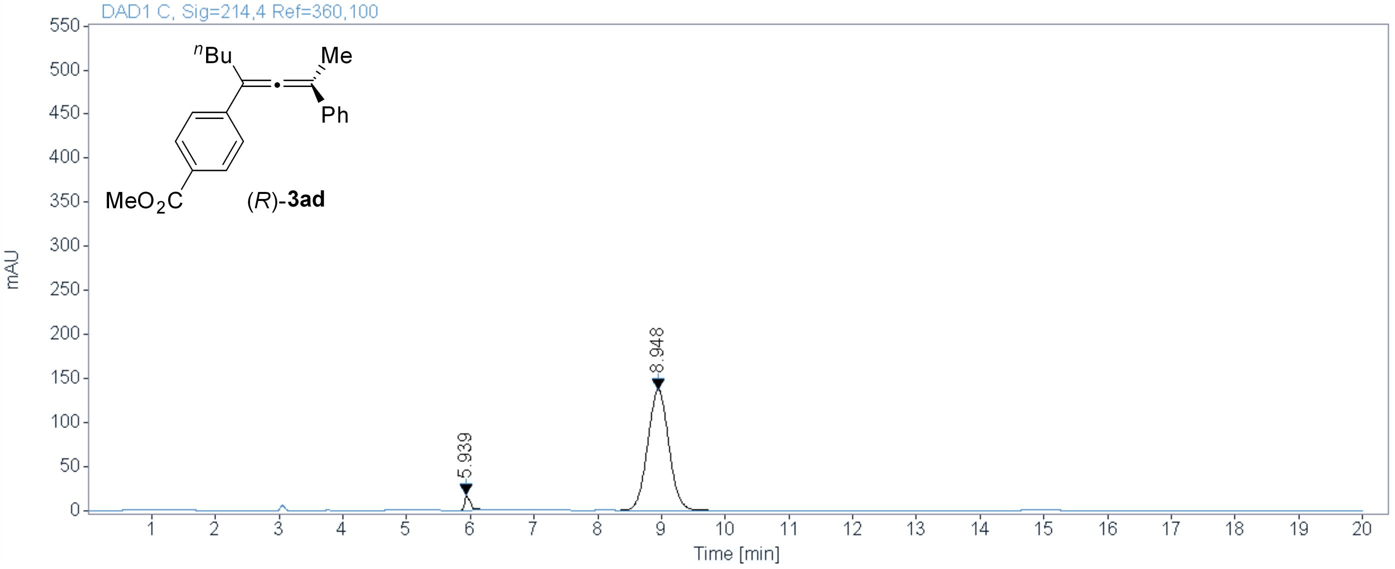

Signal: DAD1 C, Sig=214,4 Ref=360,100

RT [min]	Width [min]	Height	Area	Area%
5.939	0.1082	17.0789	110.8398	3.3903
8.948	0.3582	137.4148	3158.4514	96.6097
		Sum	3269.2912	100.0000

Supplementary Figure 248. HPLC spectrum for (*R*)-**3ad**

Area Percent Report

Agilent Technologies

sample zwf-8-072-rac-OD-H-99.5-0.5-1.0-214

Data file: C:\Users\Public\Documents\ChemStation\1\Data\zwf-allenioc acid_LC 2020-05-04 21-20-37\005-P1-C3-zwf-8-072-rac.D

Acquisition Data:

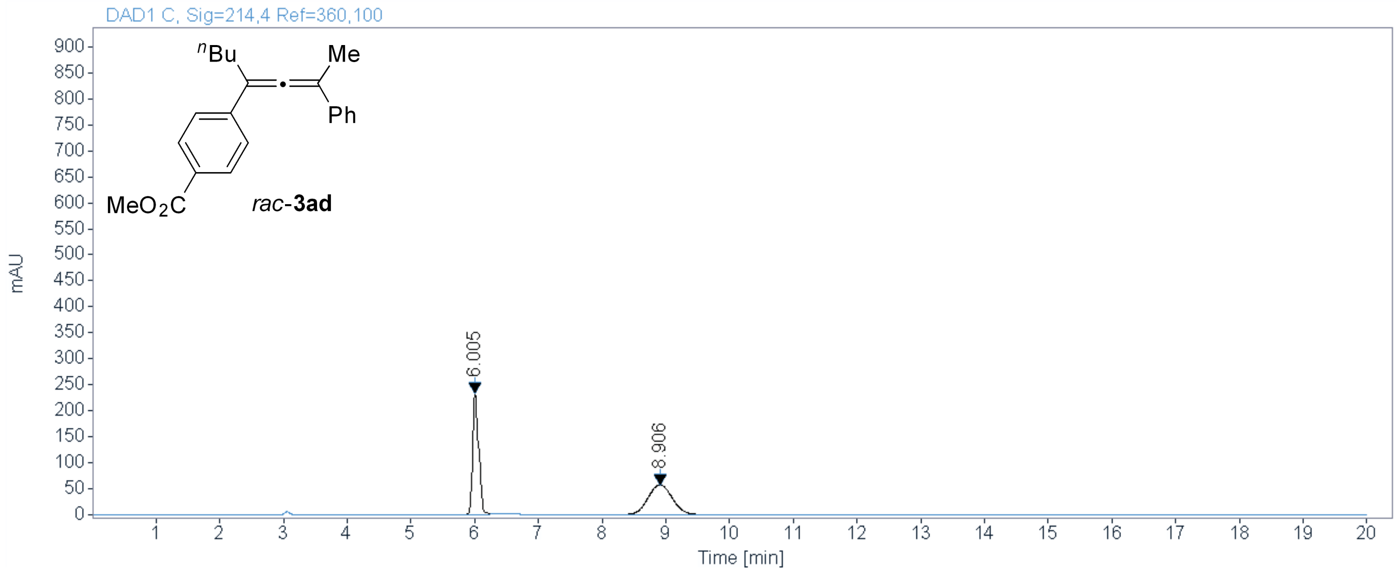

Signal: DAD1 C, Sig=214,4 Ref=360,100

RT [min]	Width [min]	Height	Area	Area%
6.005	0.1102	234.1500	1547.5454	50.2223
8.906	0.4193	57.4855	1533.8444	49.7777
		Sum	3081.3898	100.0000

Supplementary Figure 249. HPLC spectrum for *rac*-**3ad**

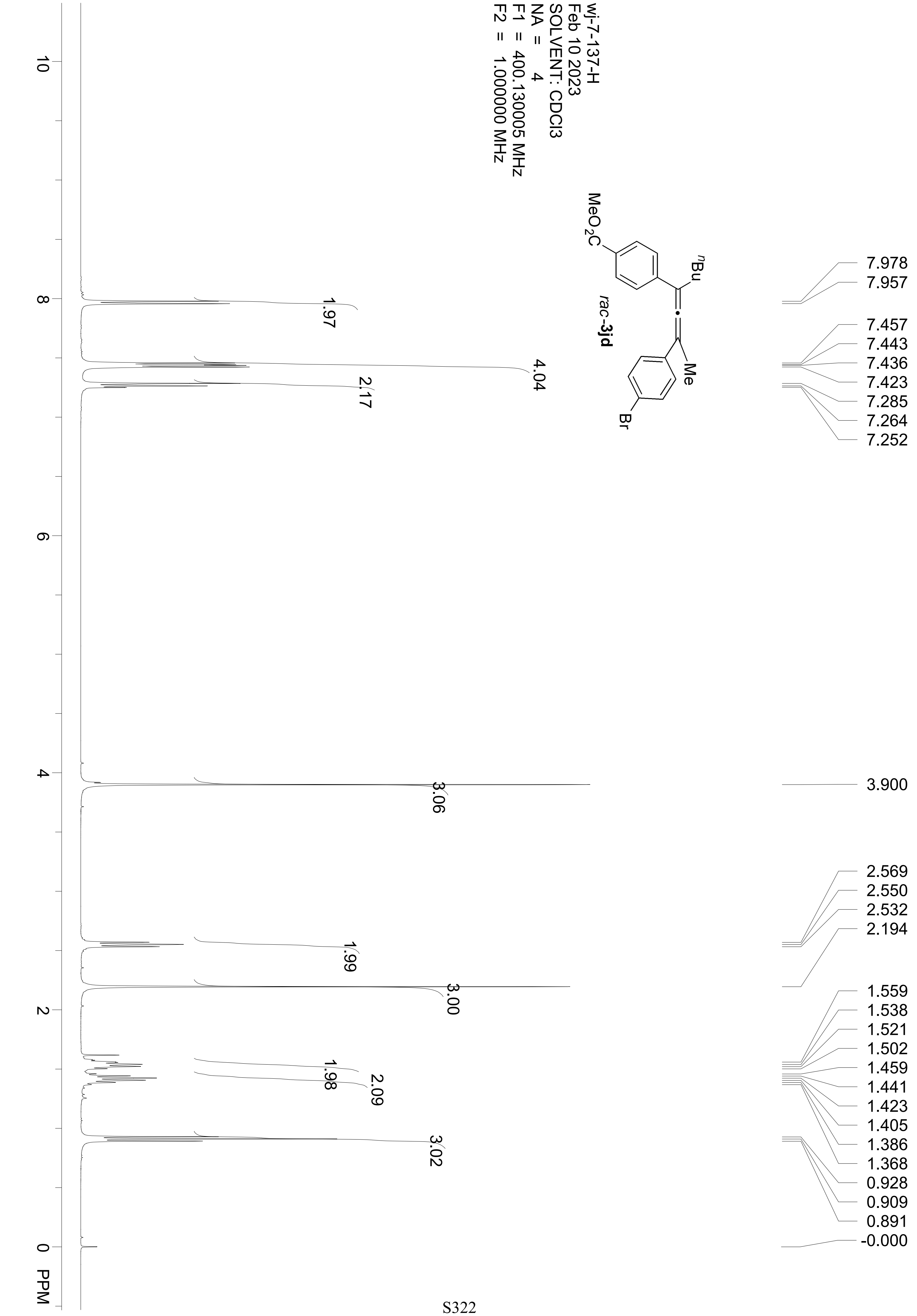

Supplementary Figure 250. ^{1}H NMR (400 MHz, $CDCl_3$) spectrum for *rac*-**3jd**

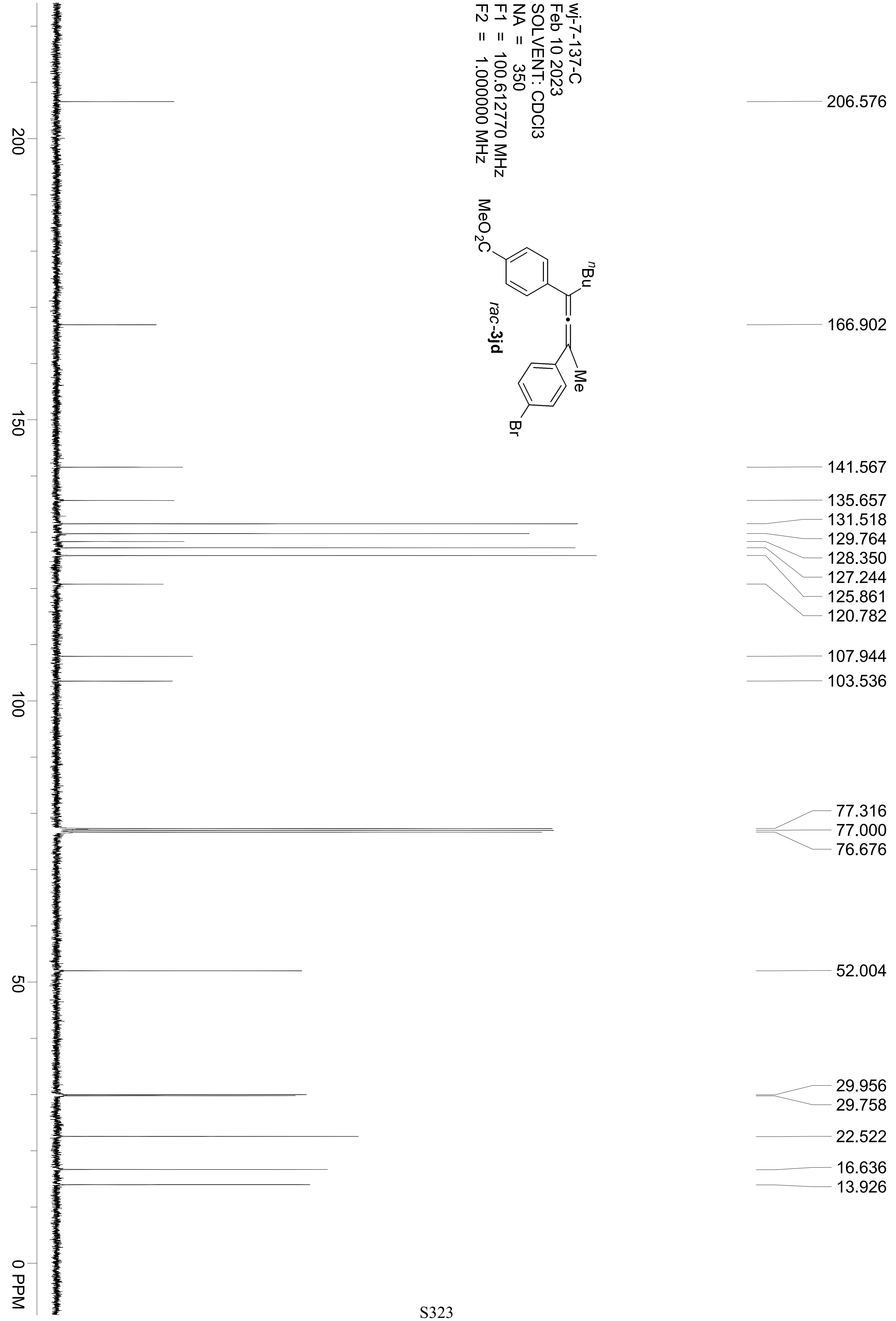

Supplementary Figure 251. ^{13}C NMR (100 MHz, $CDCl_3$) spectrum for *rac*-**3jd**

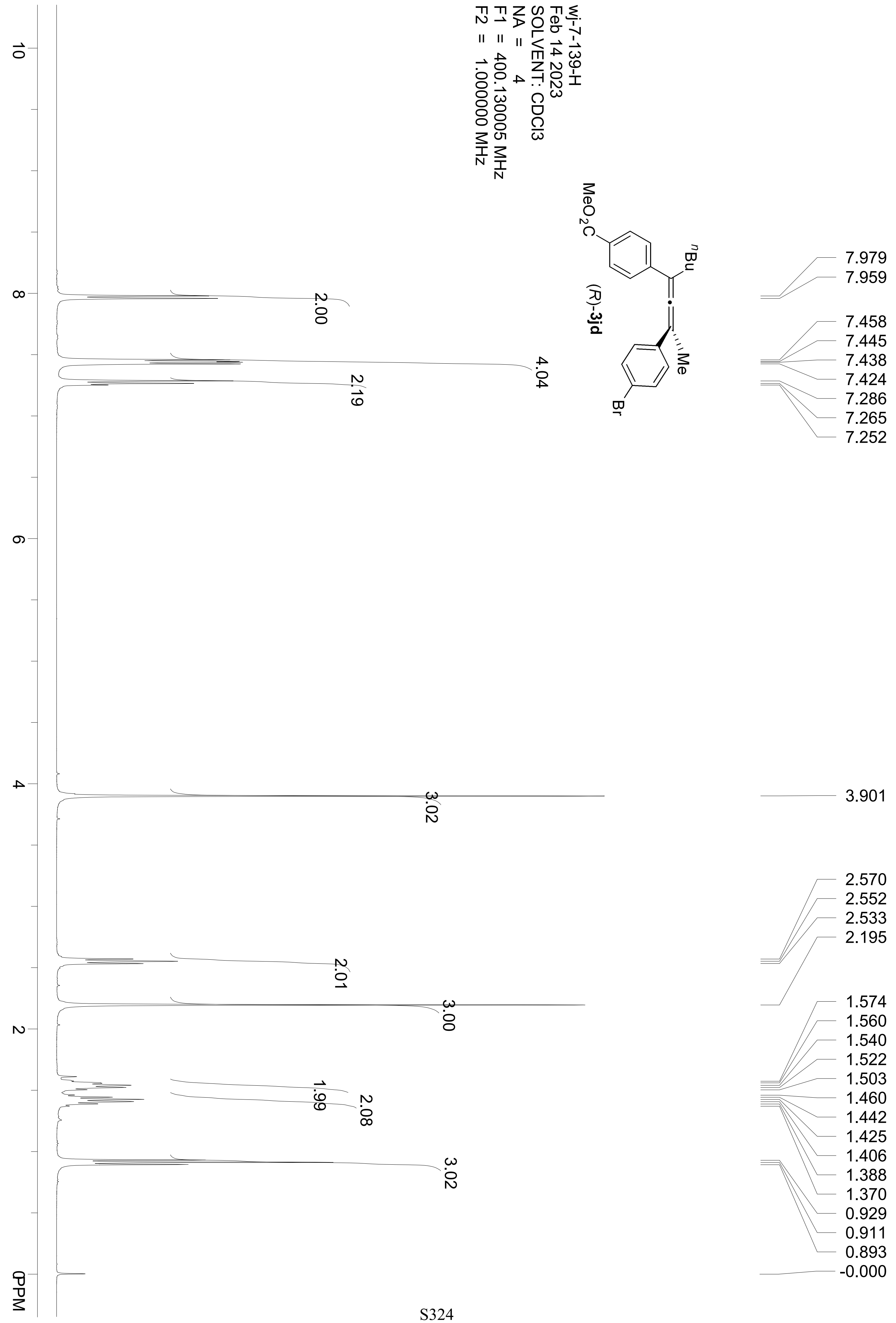

Supplementary Figure 252. ^{1}H NMR (400 MHz, $CDCl_3$) spectrum for (*R*)-**3jd**

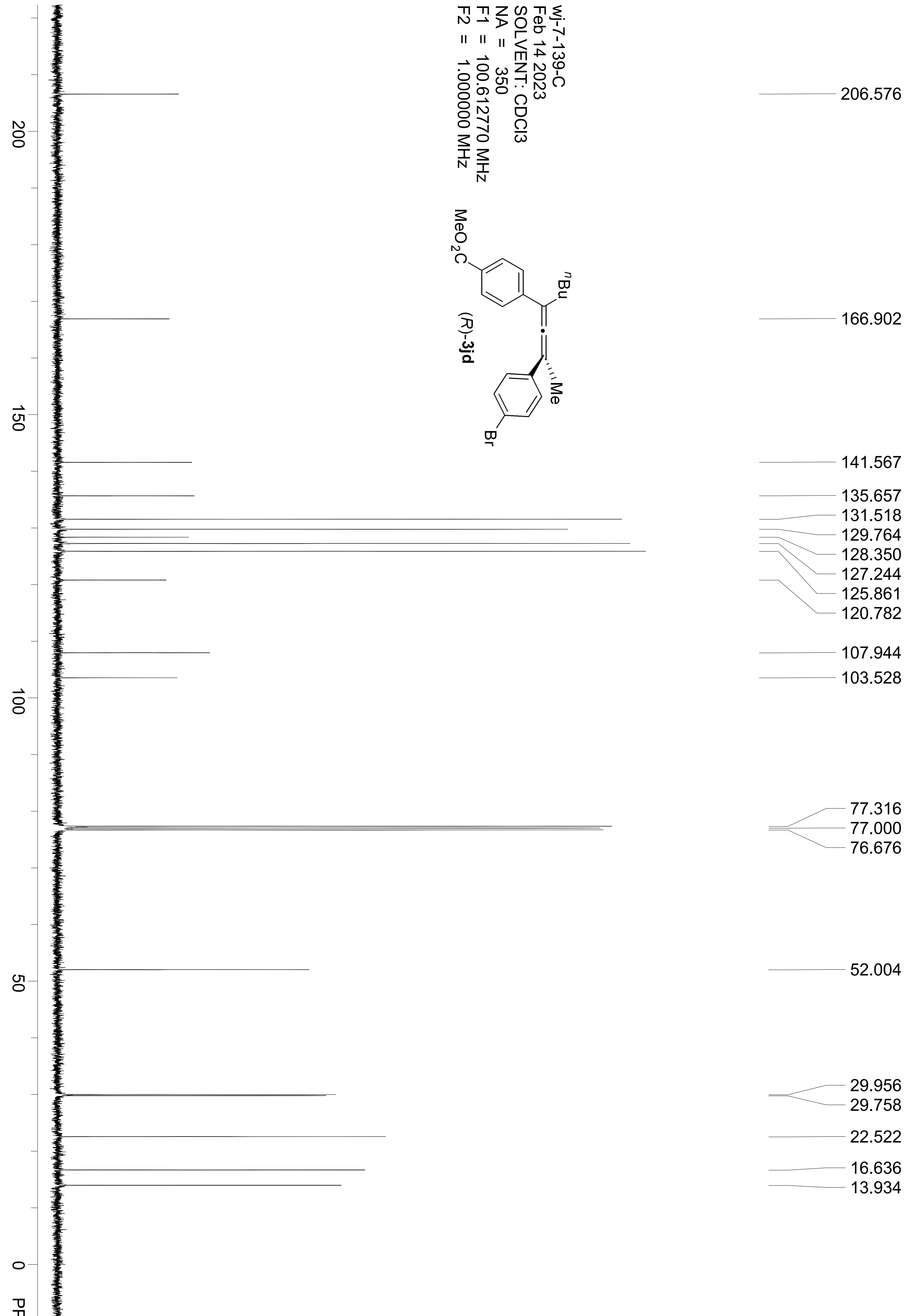

Supplementary Figure 253. ^{13}C NMR (100 MHz, $CDCl_3$) spectrum for (*R*)-**3jd**

Area Percent Report

sample wj-7-139-AD-H-98-2-1.0-214

Data file: C:\Users\Public\Documents\ChemStation\1\Data\2023-02-14\xzs_LC 2023-02-14 12-00-42\043-P2-C2-wj-7-139.D

Acquisition Data:

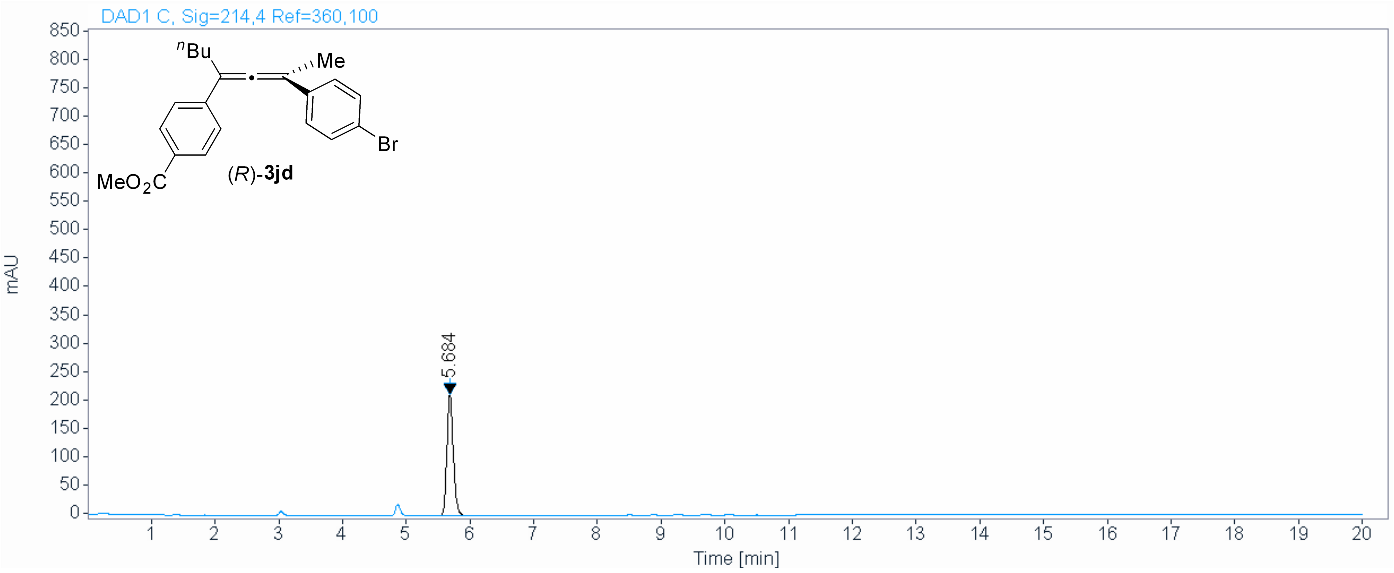

Signal: DAD1 C, Sig=214,4 Ref=360,100

RT [min]	Width [min]	Height	Area	Area%
5.684	0.1078	214.5440	1504.4891	100.0000
		Sum	1504.4891	100.0000

Supplementary Figure 254. HPLC spectrum for (*R*)-**3jd**

Area Percent Report

sample wj-7-139-rac-AD-H-98-2-1.0-214

Data file: C:\Users\Public\Documents\ChemStation\1\Data\2023-02-14\xzs_LC 2023-02-14 12-00-42\042-P2-C1-wj-7-139-rac.D

Acquisition Data:

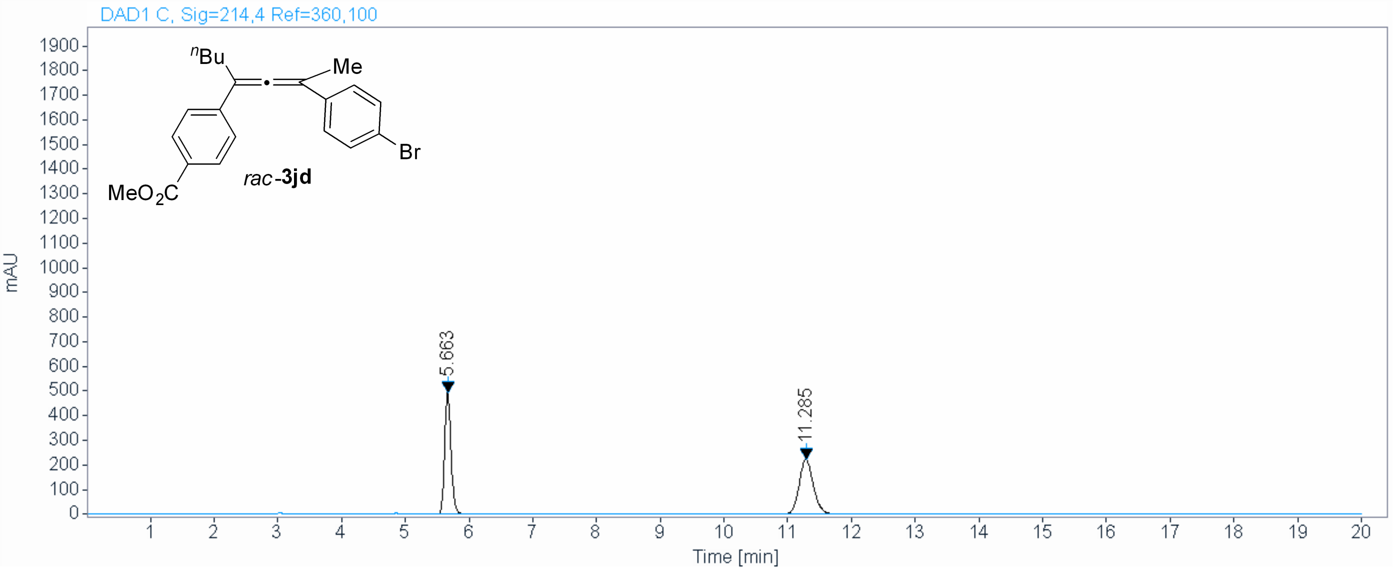

Signal: DAD1 C, Sig=214,4 Ref=360,100

RT [min]	Width [min]	Height	Area	Area%
5.663	0.1051	495.2145	3441.5920	49.9008
11.285	0.2418	221.7415	3455.2805	50.0992
		Sum	6896.8726	100.0000

Supplementary Figure 255. HPLC spectrum for *rac*-**3jd**

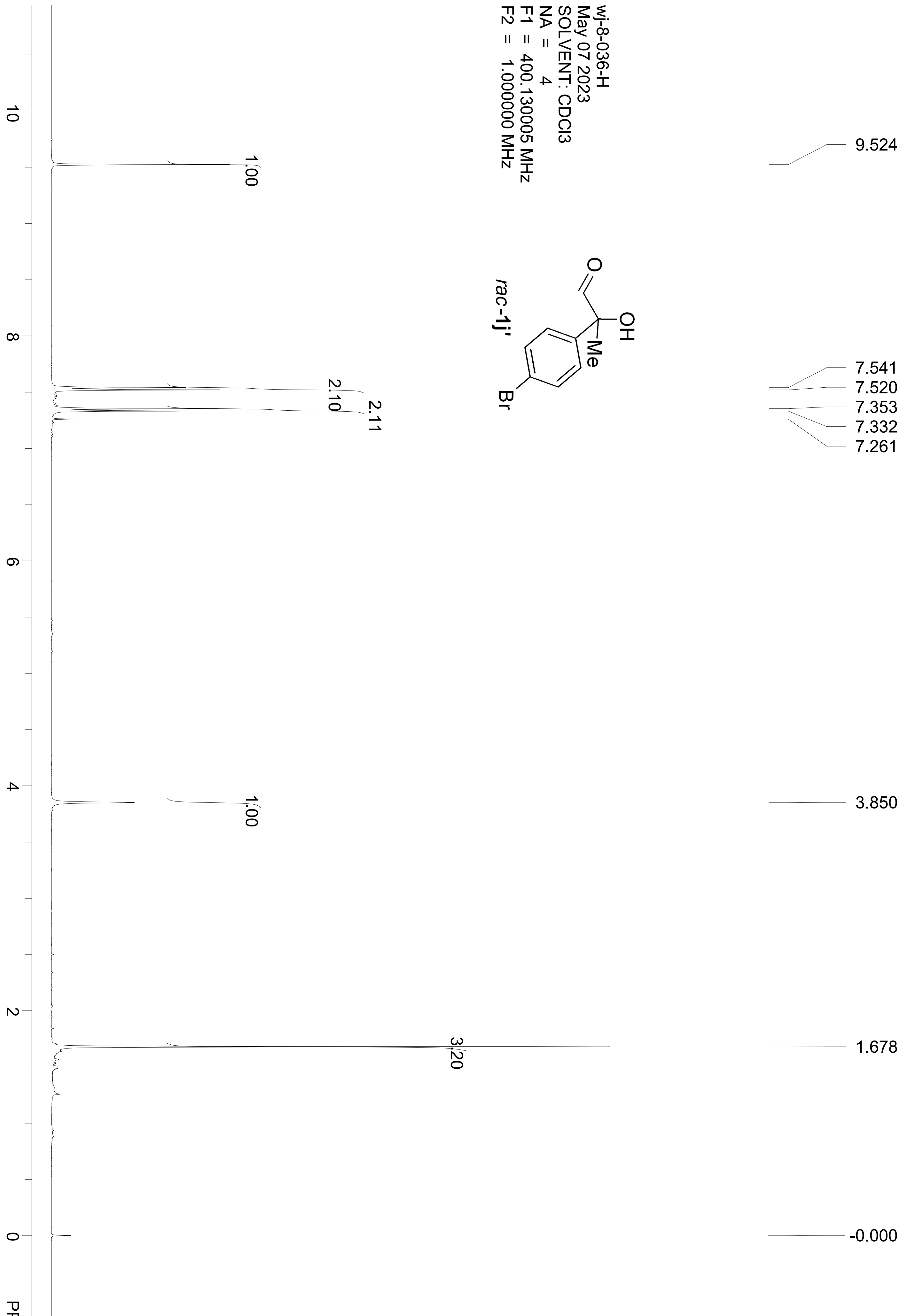

Supplementary Figure 256. ^{1}H NMR (400 MHz, $CDCl_3$) spectrum for *rac*-**1j'**

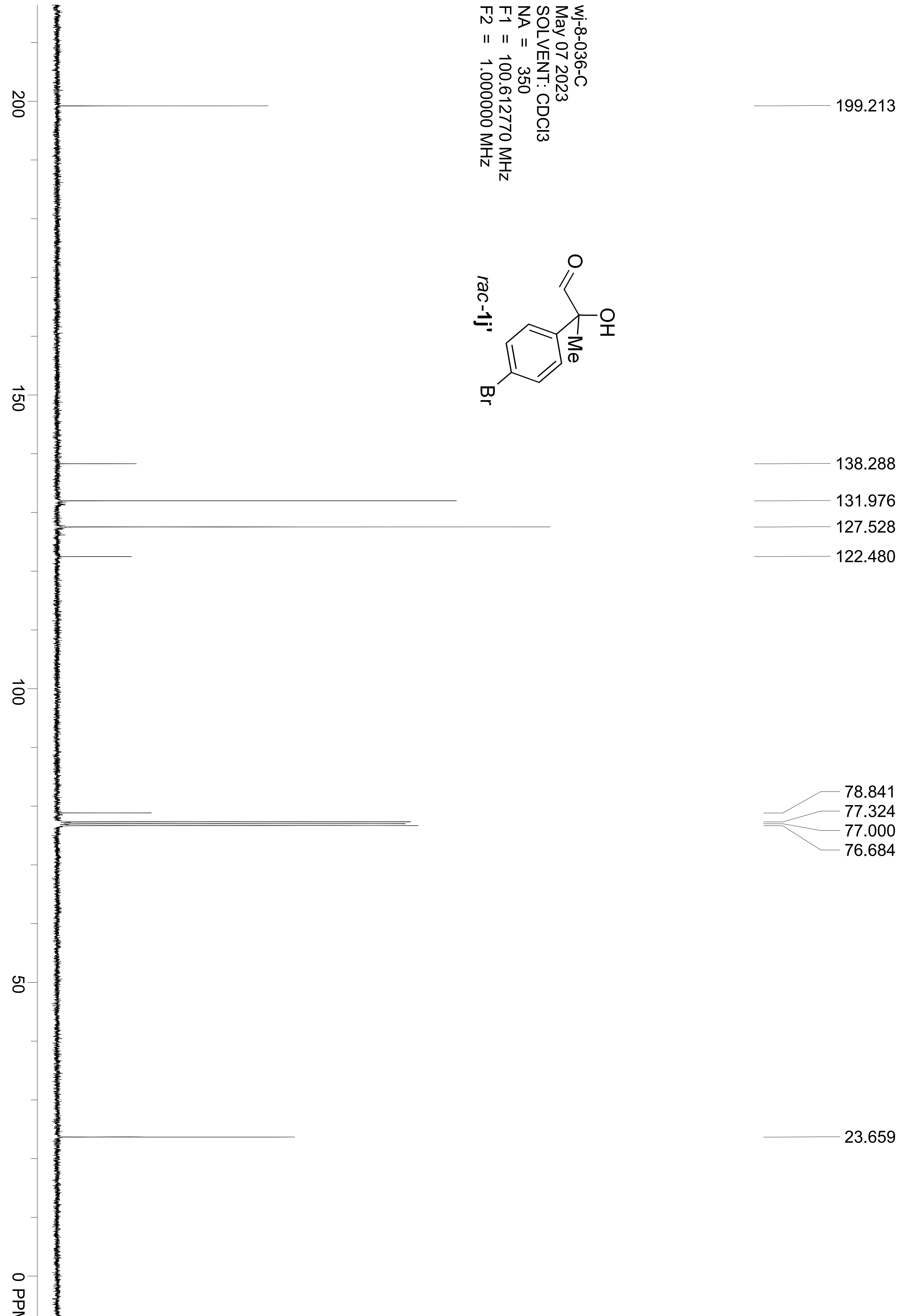

Supplementary Figure 257. ^{13}C NMR (100 MHz, $CDCl_3$) spectrum for *rac*-**1j'**

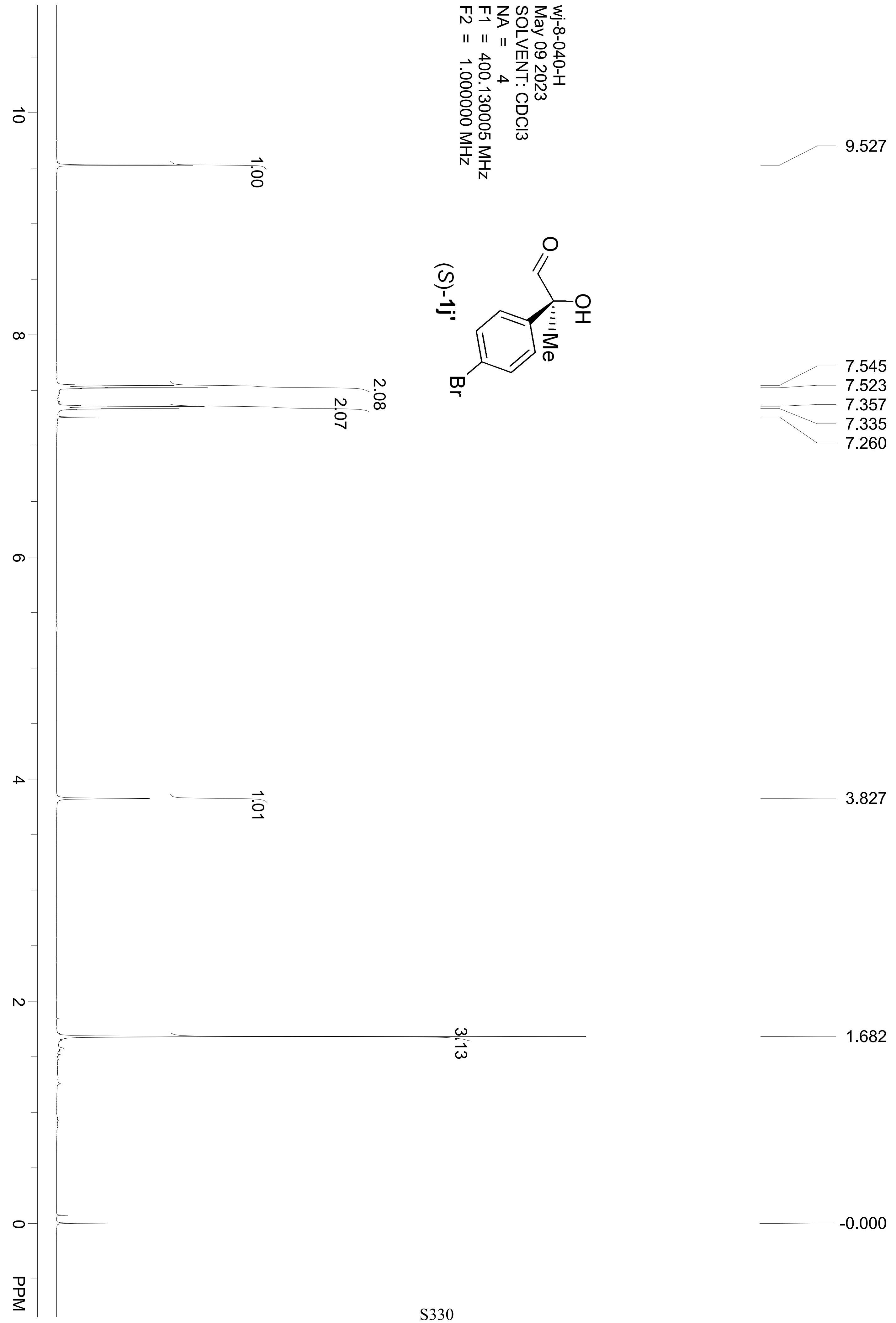

Supplementary Figure 258. ^{1}H NMR (400 MHz, $CDCl_3$) spectrum for (*S*)-**1j'**

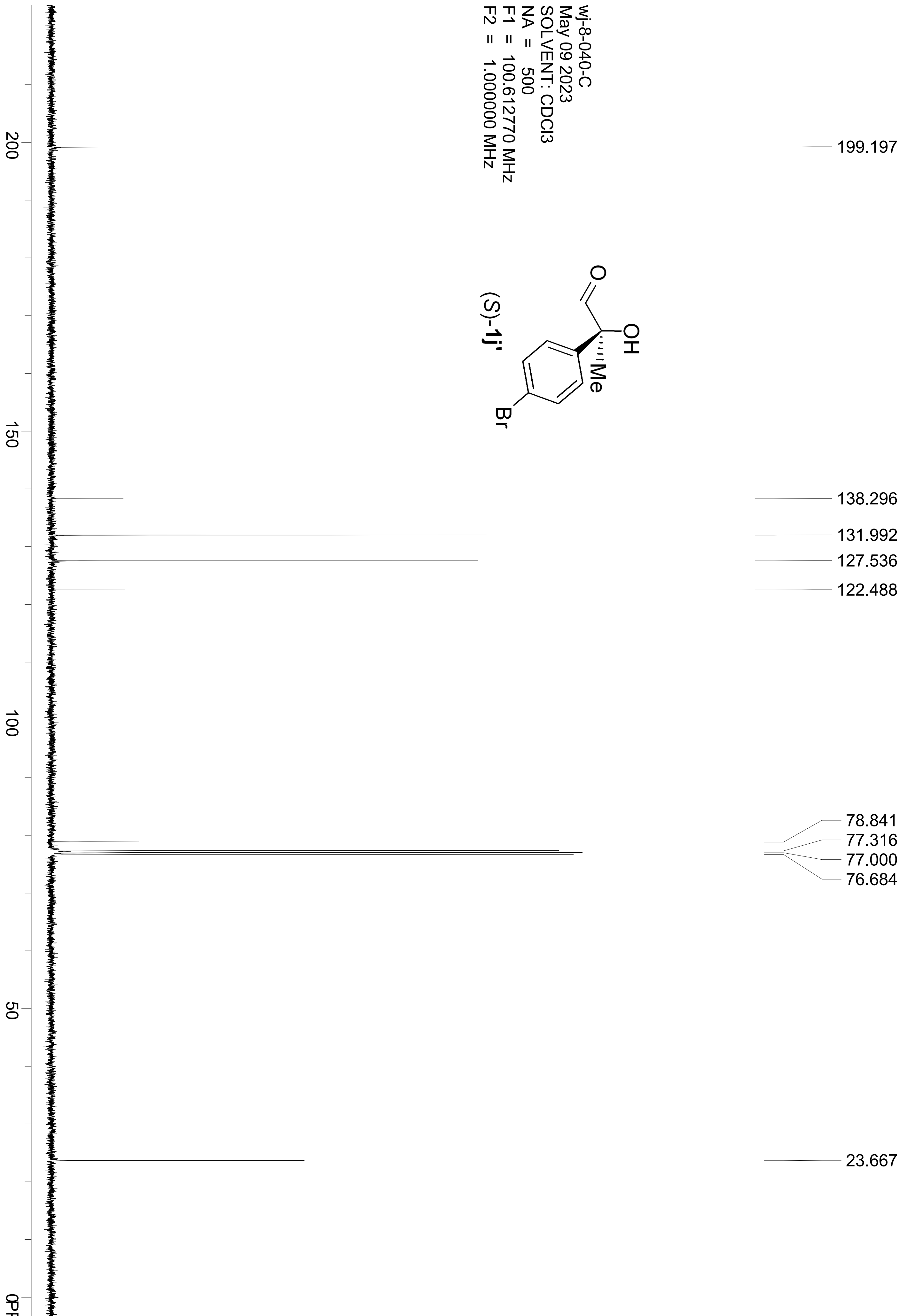

Supplementary Figure 259. ^{13}C NMR (100 MHz, $CDCl_3$) spectrum for (*S*)-**1j'**

Area Percent Report

sample wj-8-040-OJ-H-95-5-1.0-214

Data file: C:\Users\Public\Documents\ChemStation\1\Data\2023-05-11\xzs_LC 2023-05-11 08-38-38\040-P2-C1-wj-8-040.D

Acquisition Data:

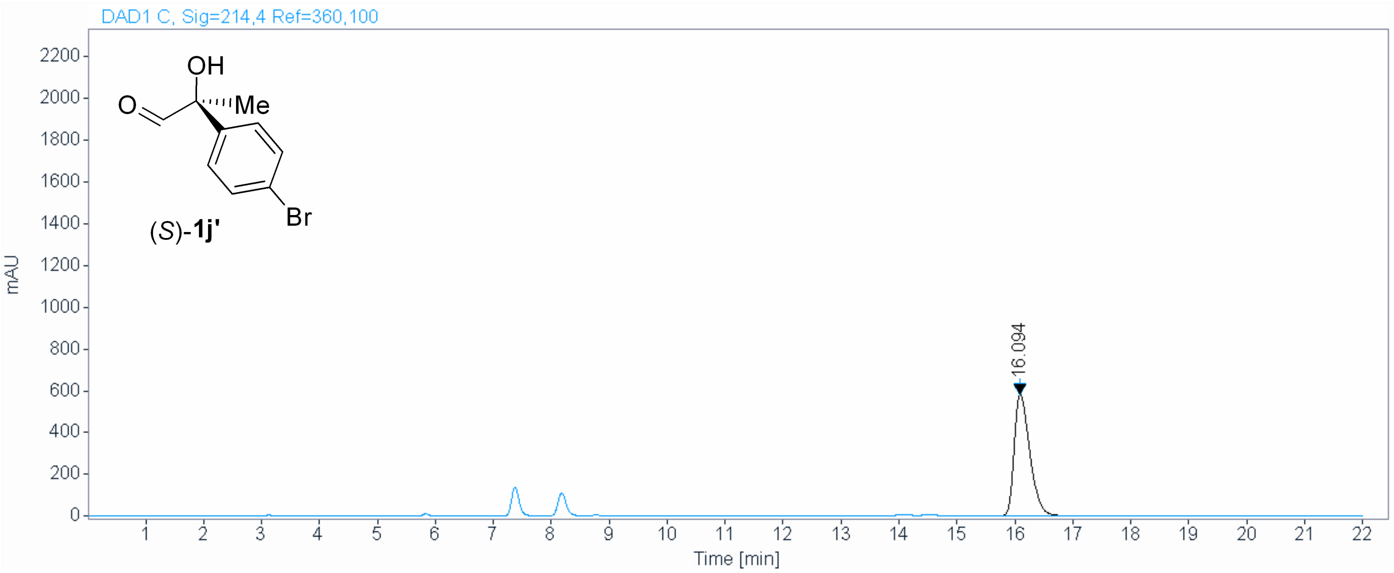

Signal: DAD1 C, Sig=214,4 Ref=360,100

RT [min]	Width [min]	Height	Area	Area%
16.094	0.3133	582.9282	10956.8496	100.0000
		Sum	10956.8496	100.0000

Supplementary Figure 260. HPLC spectrum for (*S*)-**1j'**

Area Percent Report

sample wj-8-040-rac-OJ-H-95-5-1.0-214

Data file: C:\Users\Public\Documents\ChemStation\1\Data\2023-05-11\xzs_LC 2023-05-11 08-38-38\041-P2-C2-wj-8-040-rac.D

Acquisition Data:

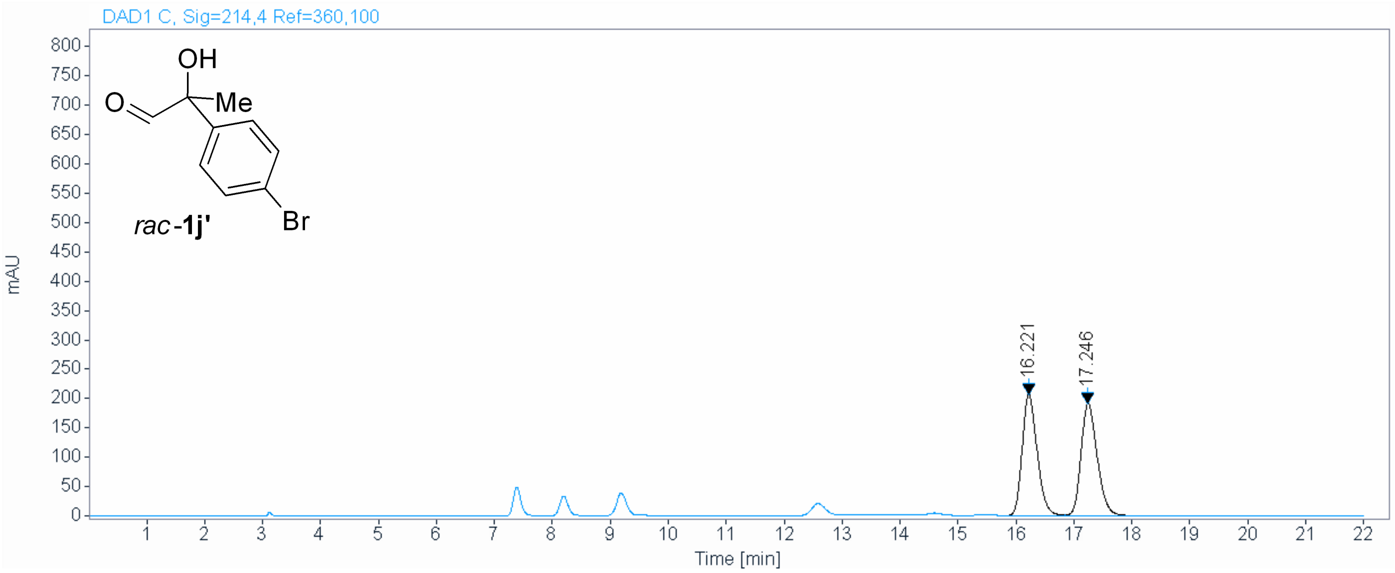

Signal: DAD1 C, Sig=214,4 Ref=360,100

RT [min]	Width [min]	Height	Area	Area%
16.221	0.2774	206.6175	3709.1831	49.8509
17.246	0.3244	191.6773	3731.3738	50.1491
		Sum	7440.5569	100.0000

Supplementary Figure 261. HPLC spectrum for *rac*-**1j'**

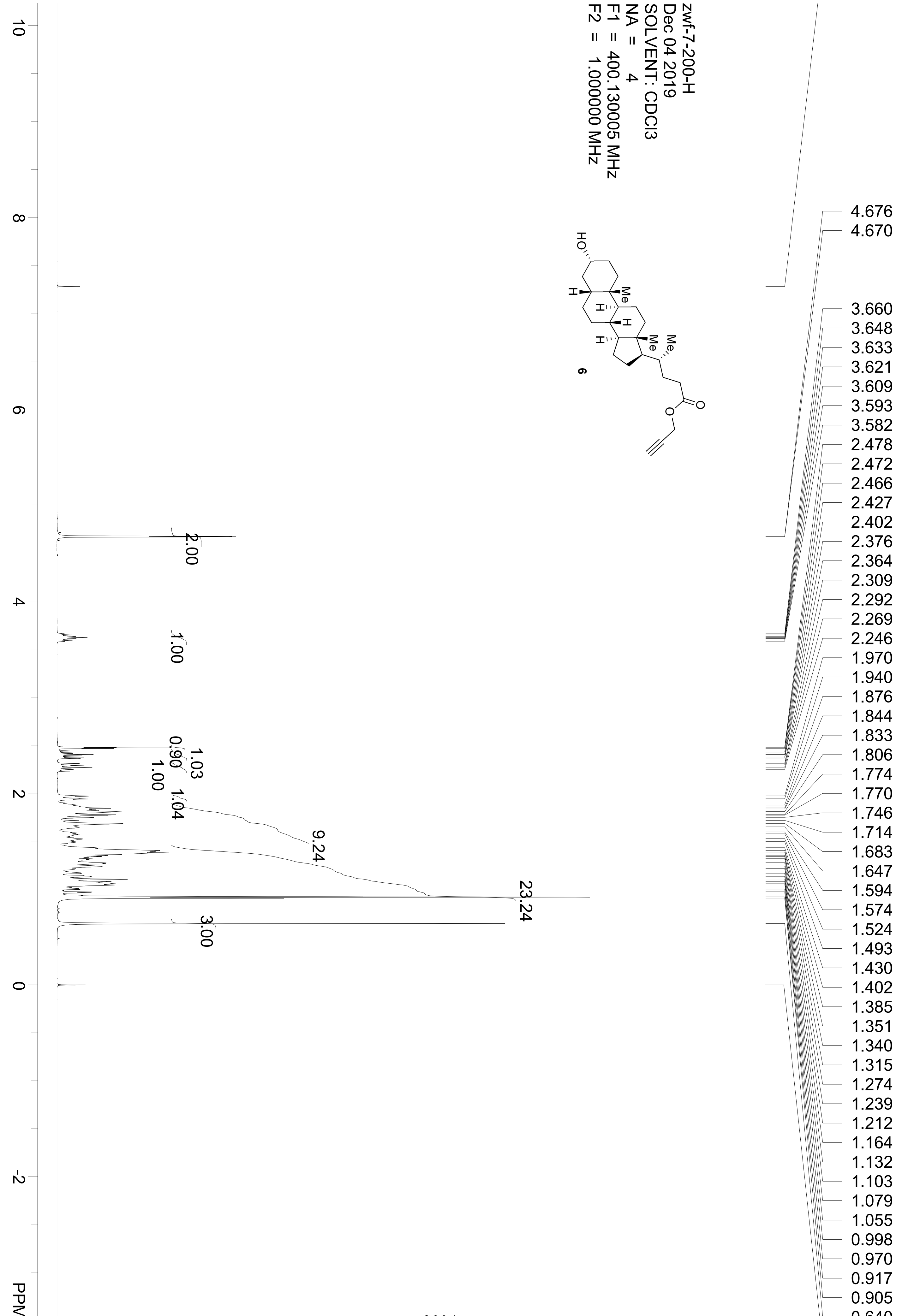

Supplementary Figure 262. ^{1}H NMR (400 MHz, $CDCl_3$) spectrum for **6**

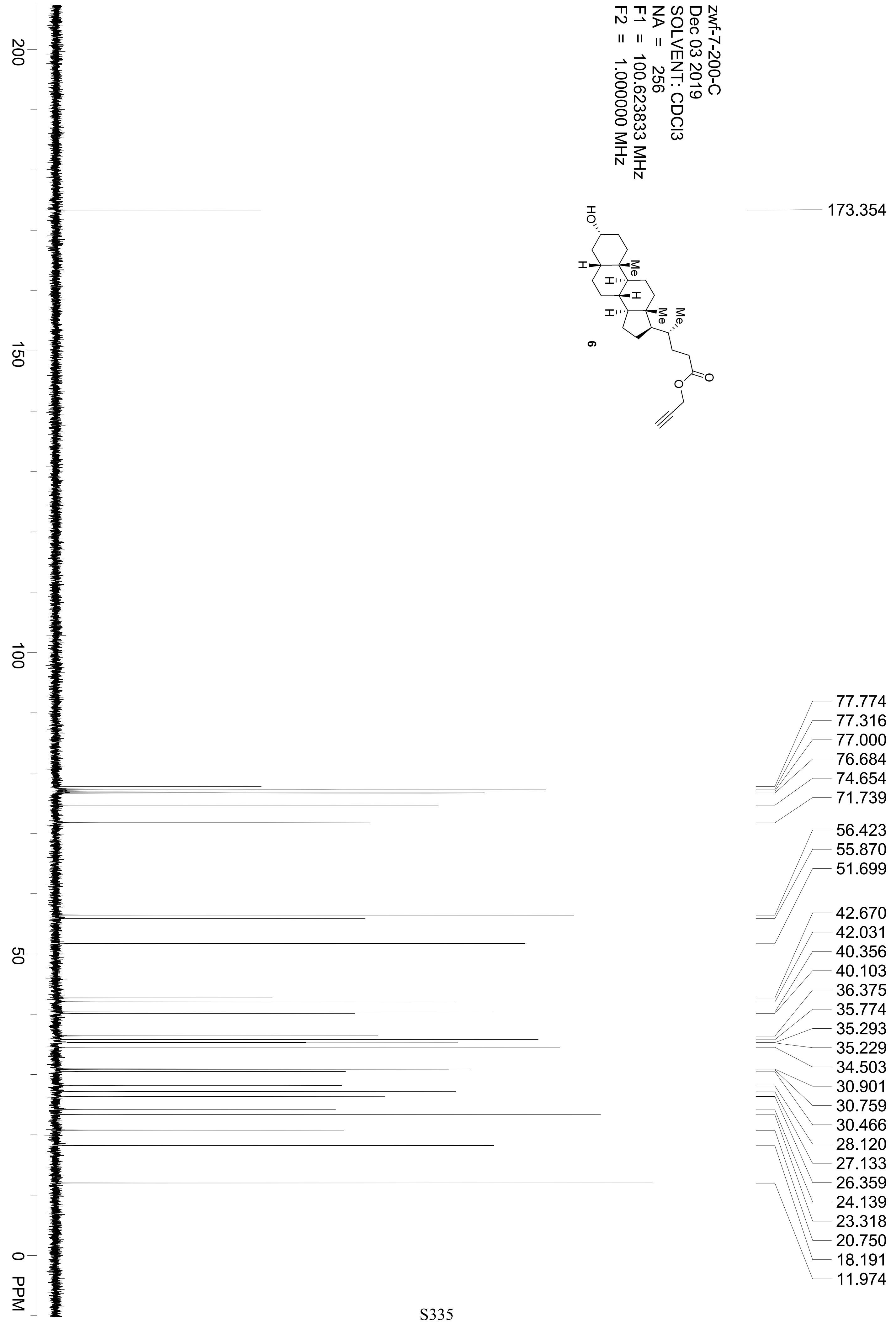

Supplementary Figure 263. ^{13}C NMR (100 MHz, $CDCl_3$) spectrum for **6**

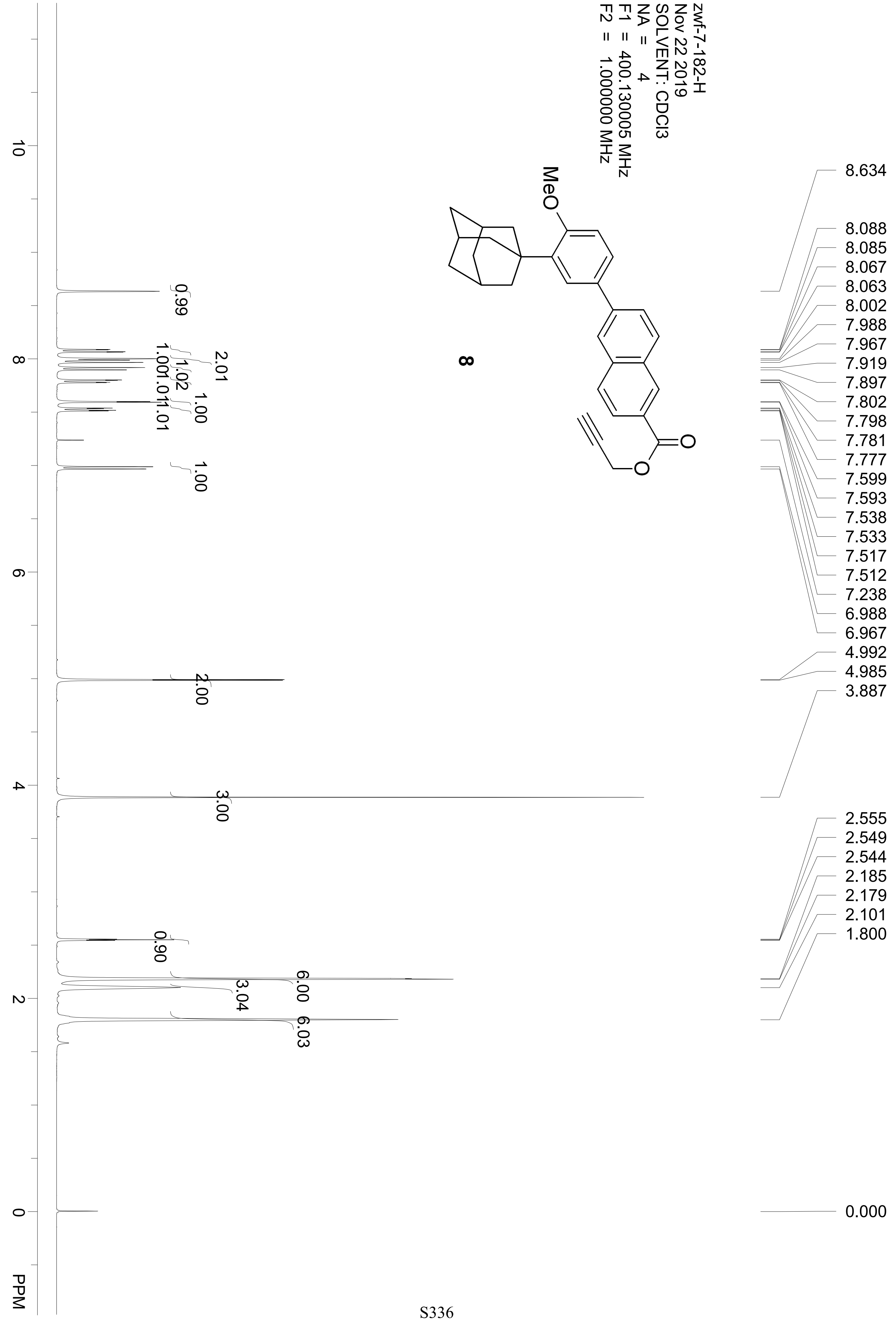

Supplementary Figure 264. ^{1}H NMR (400 MHz, $CDCl_3$) spectrum for **8**

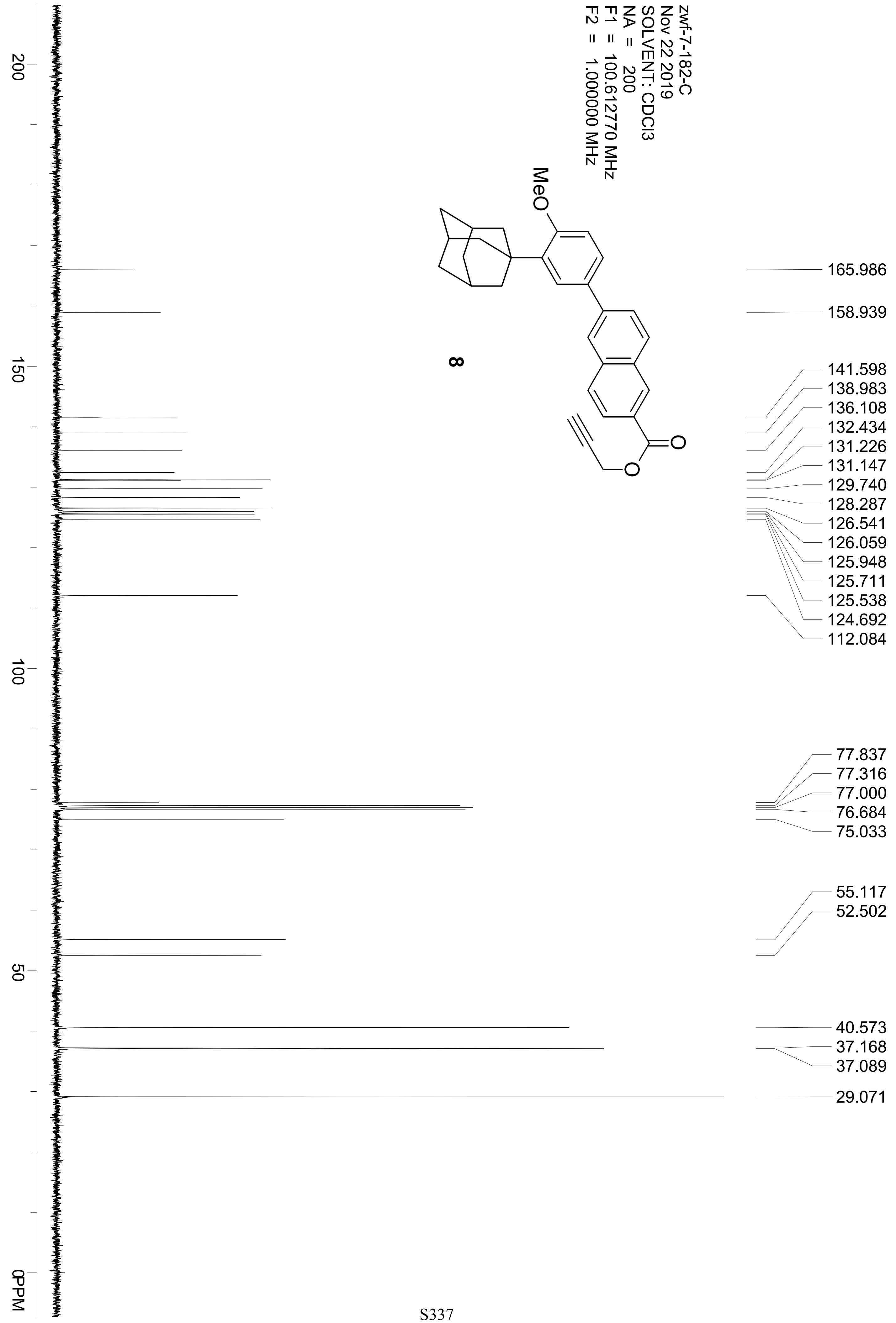

Supplementary Figure 265. ^{13}C NMR (100 MHz, $CDCl_3$) spectrum for **8**

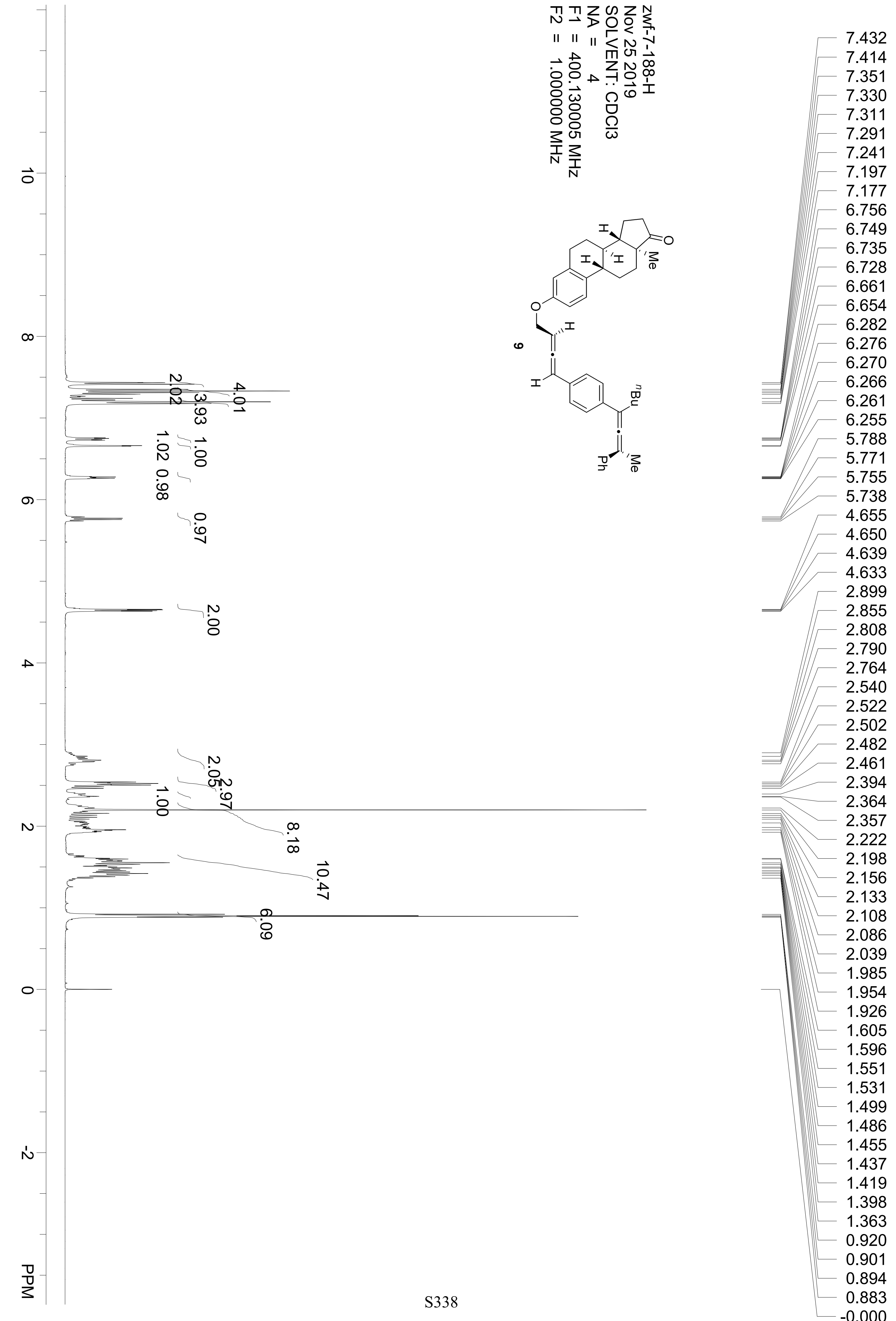

Supplementary Figure 266. ^{1}H NMR (400 MHz, $CDCl_3$) spectrum for **9**

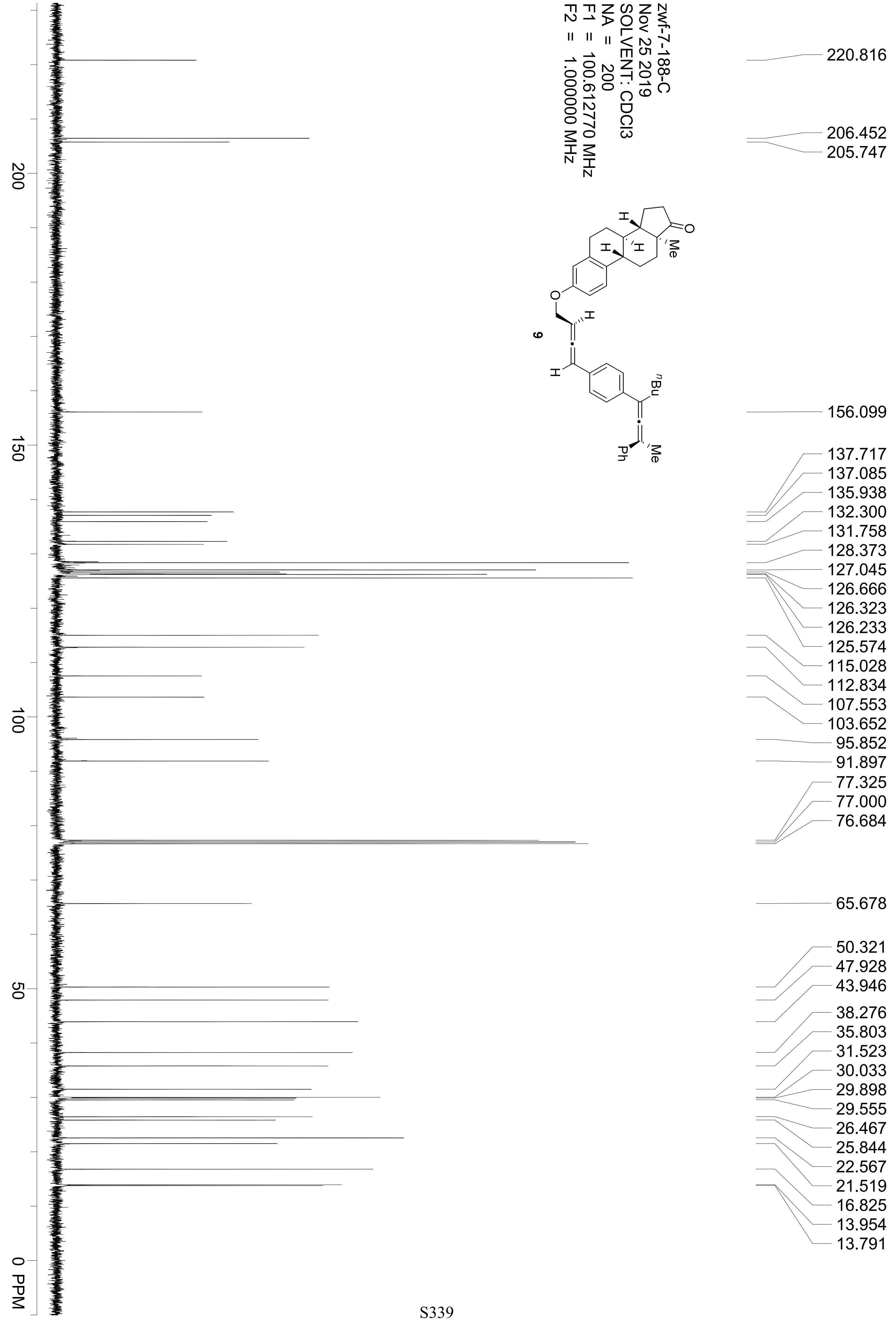

Supplementary Figure 267. ^{13}C NMR (100 MHz, $CDCl_3$) spectrum for **9**

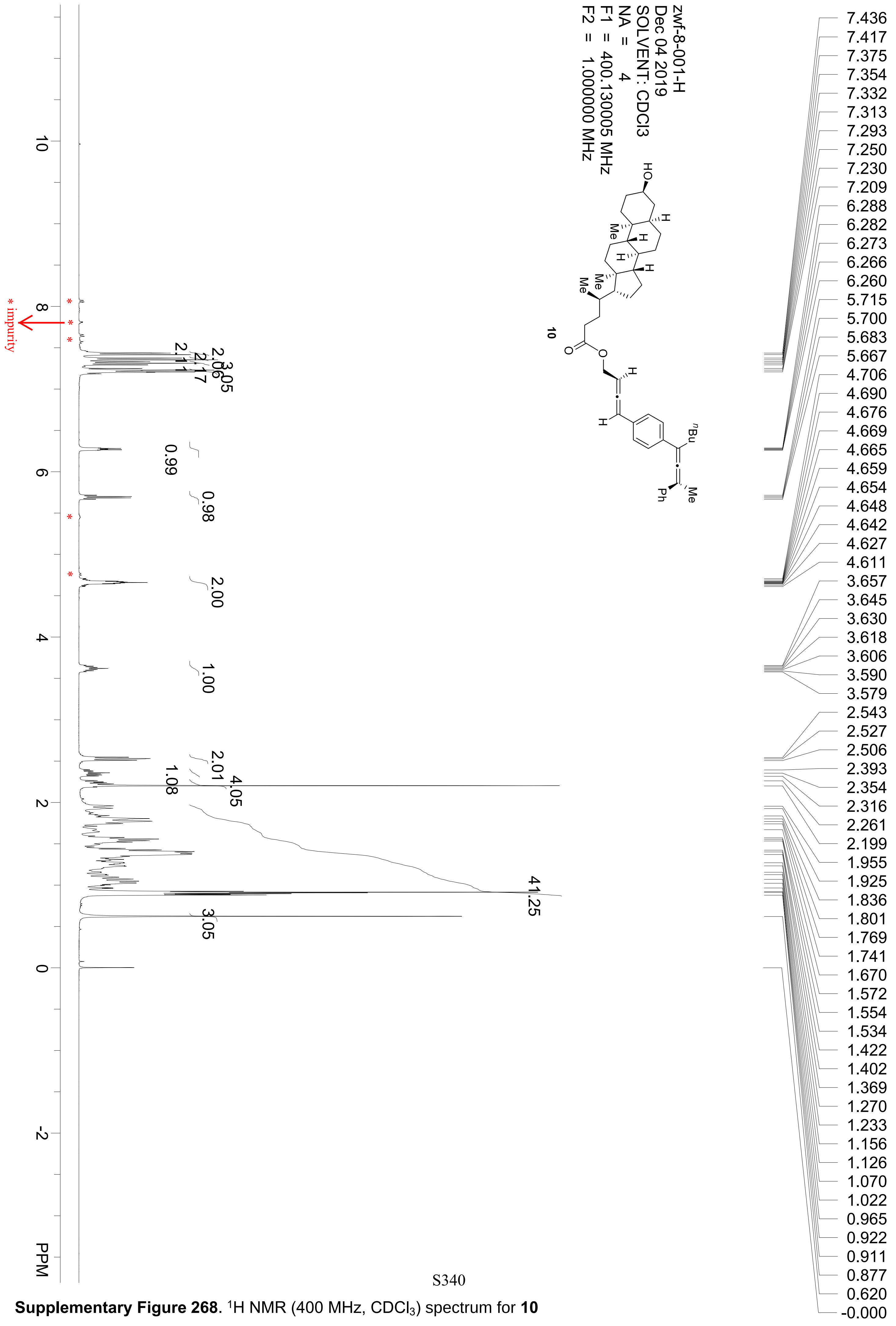

Supplementary Figure 268. ^{1}H NMR (400 MHz, $CDCl_3$) spectrum for **10**

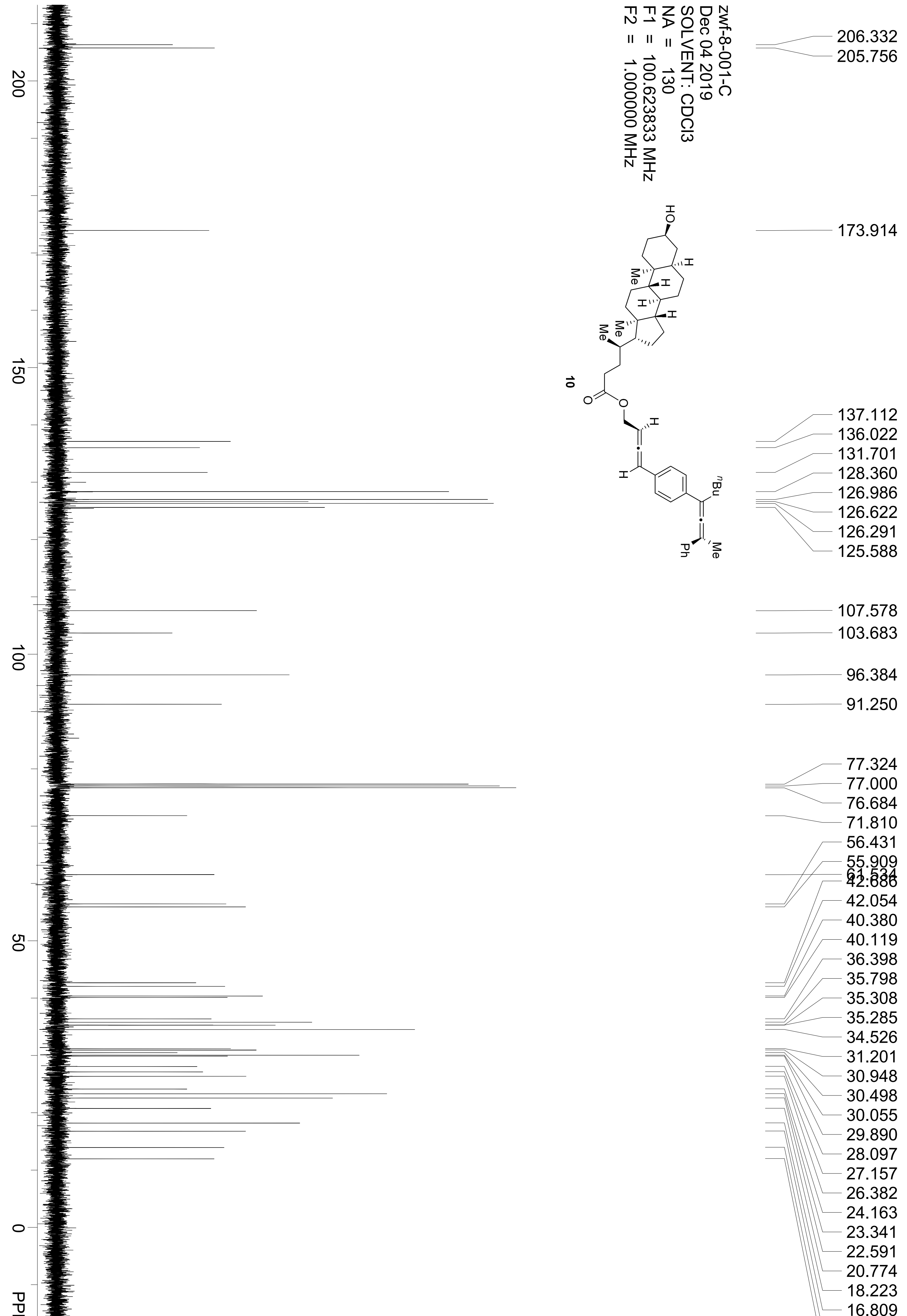

Supplementary Figure 269. ^{13}C NMR (100 MHz, $CDCl_3$) spectrum for **10**

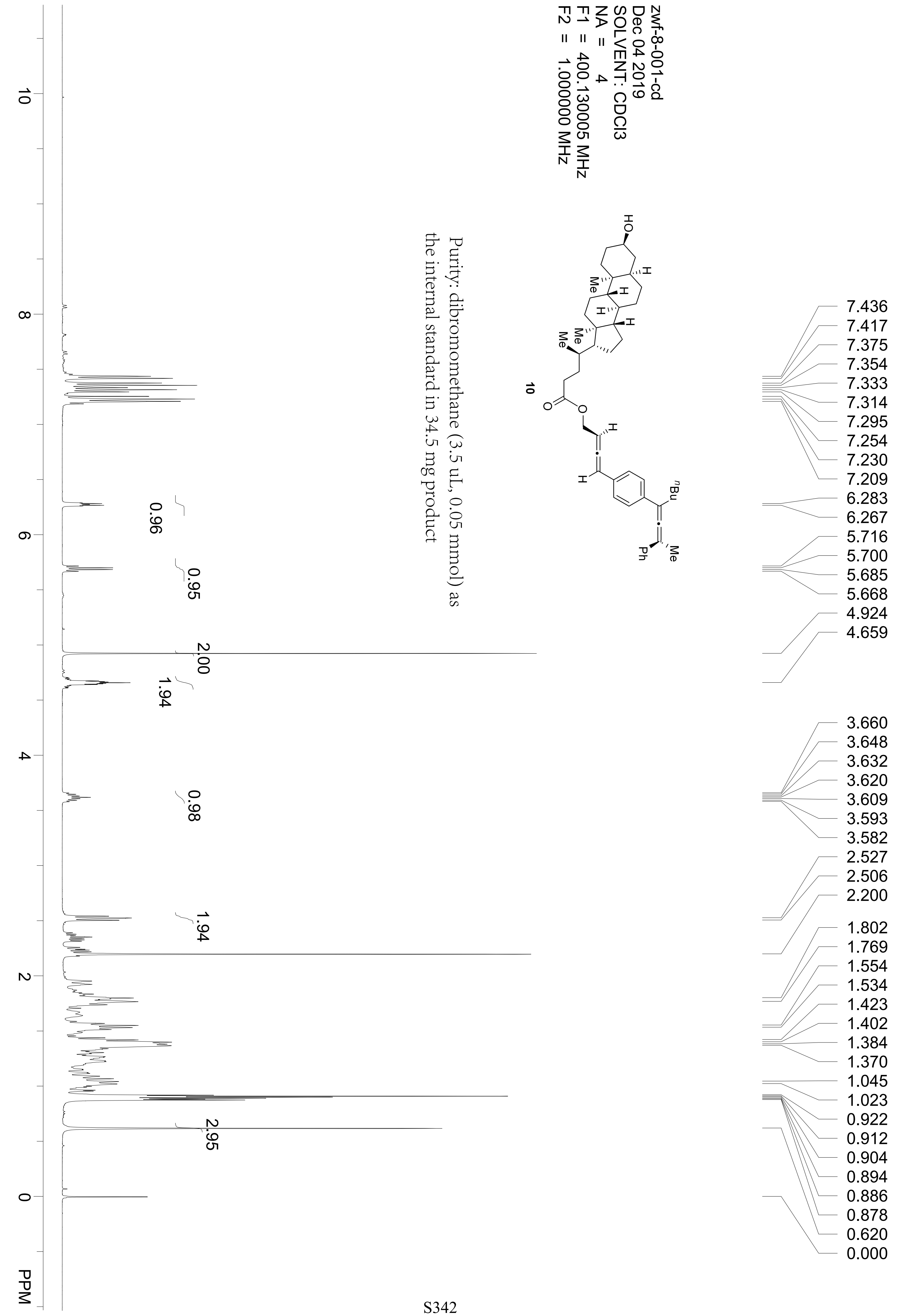

Supplementary Figure 270. Purity spectrum for **10**

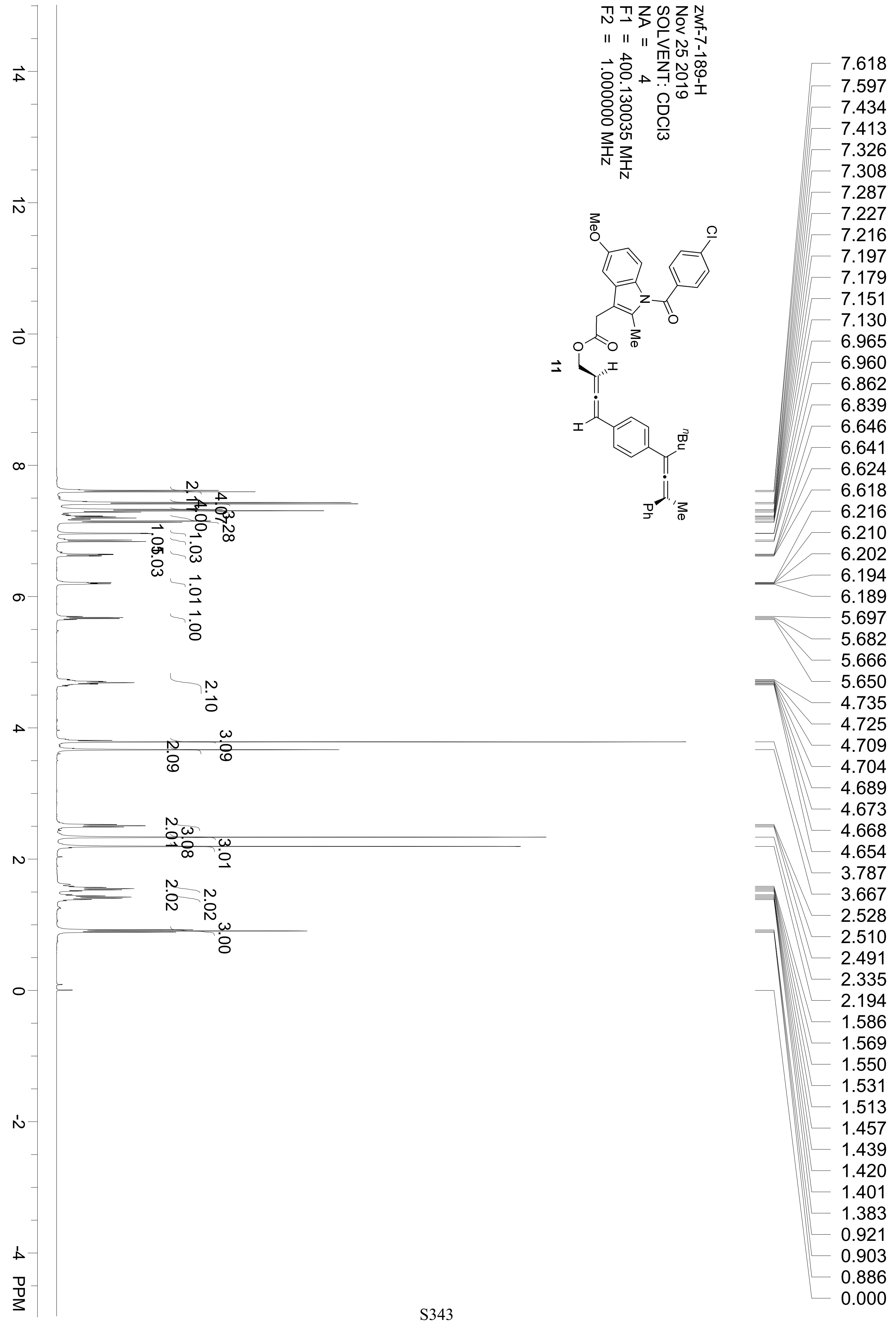

Supplementary Figure 271. ^{1}H NMR (400 MHz, $CDCl_3$) spectrum for **11**

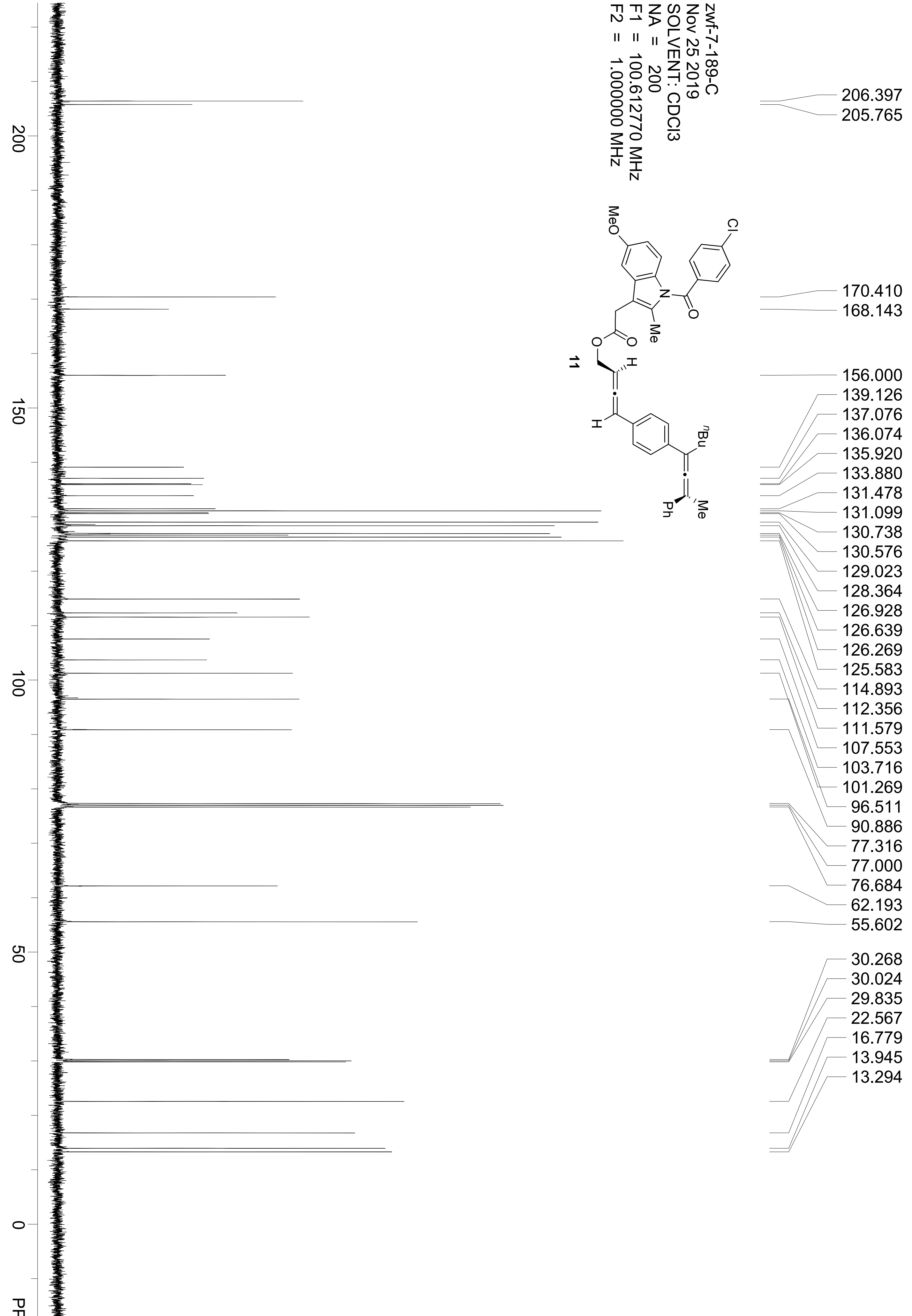

Supplementary Figure 272. ^{13}C NMR (100 MHz, $CDCl_3$) spectrum for **11**

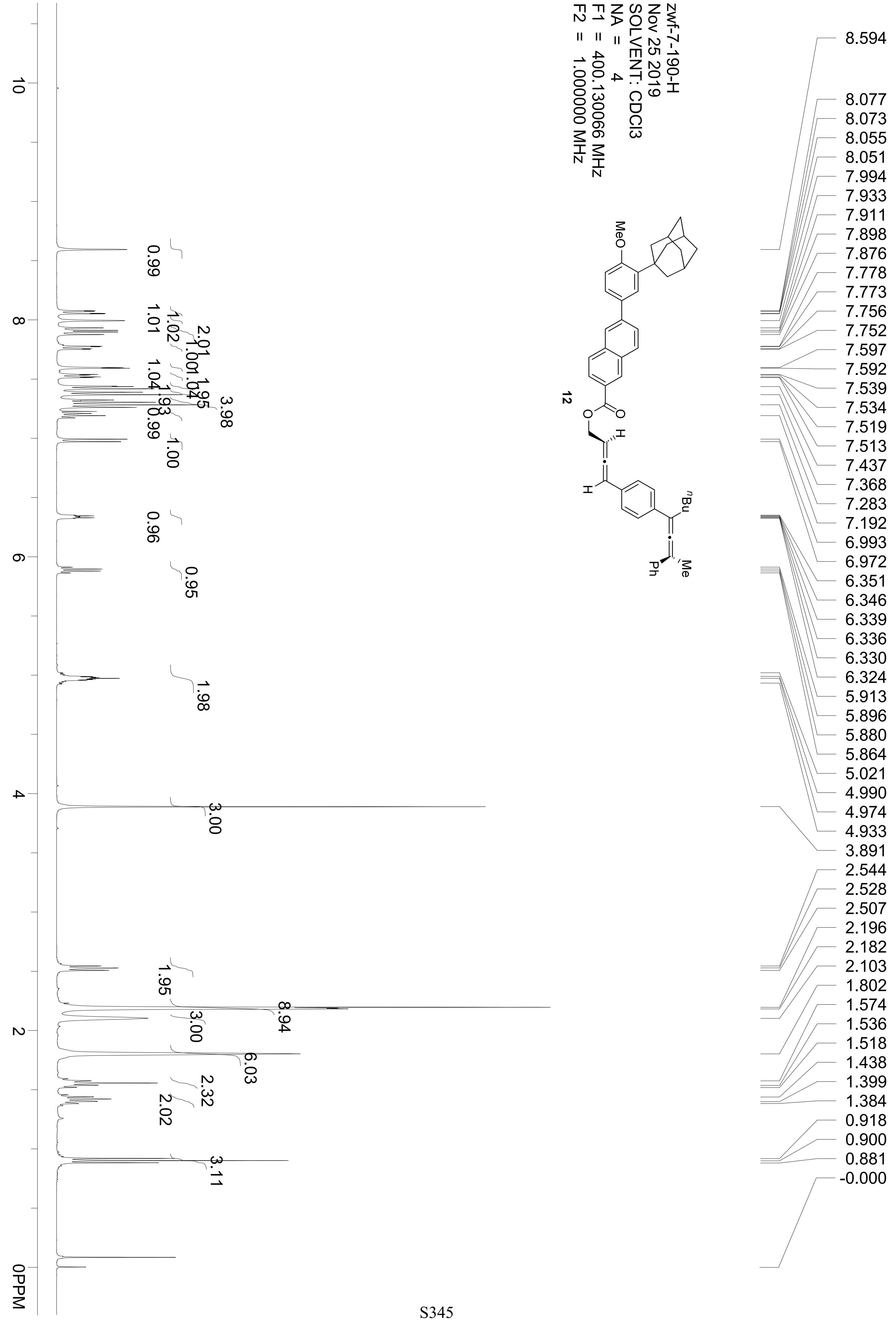

Supplementary Figure 273. ^{1}H NMR (400 MHz, $CDCl_3$) spectrum for **12**

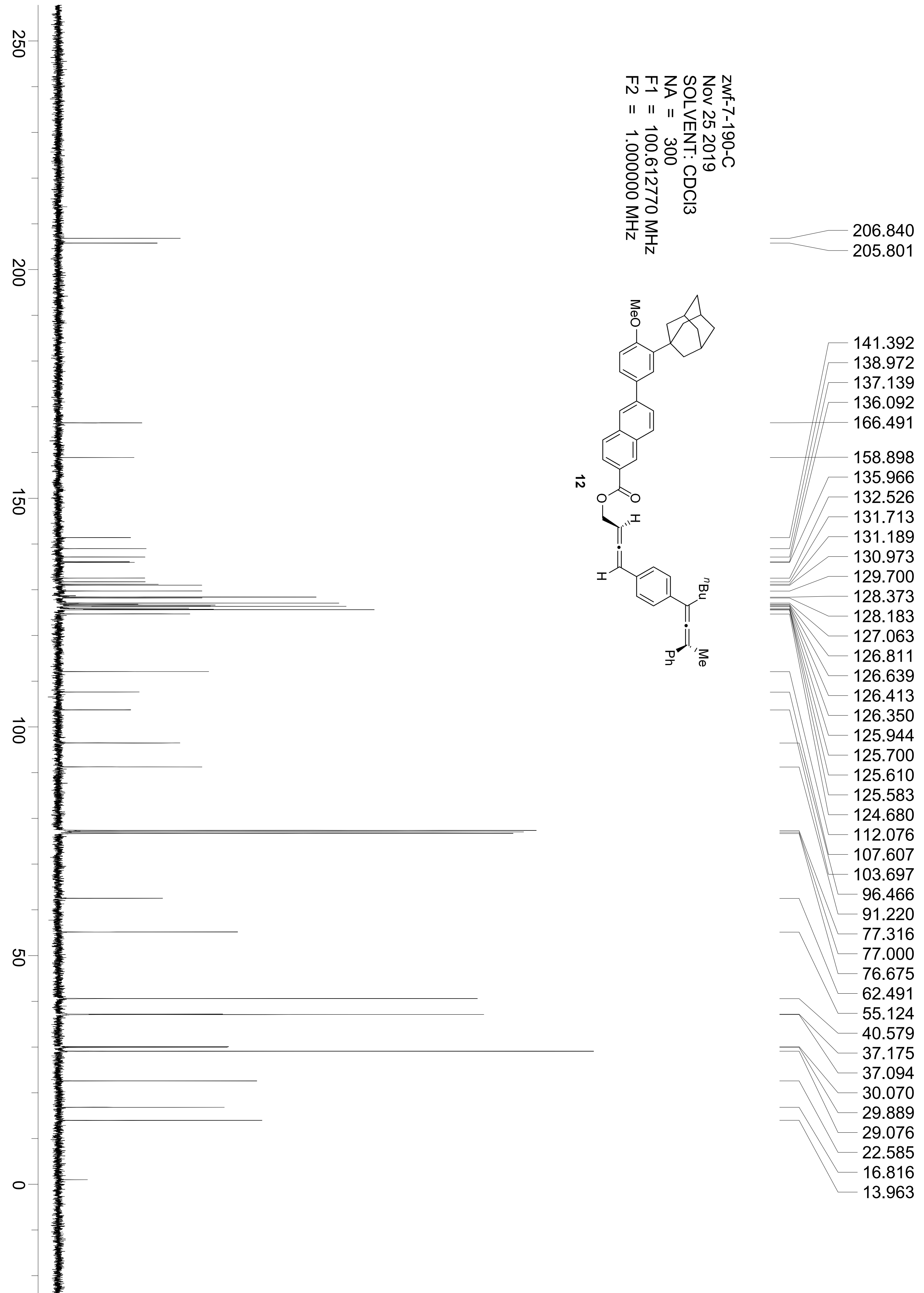

Supplementary Figure 274. ^{13}C NMR (100 MHz, $CDCl_3$) spectrum for **12**

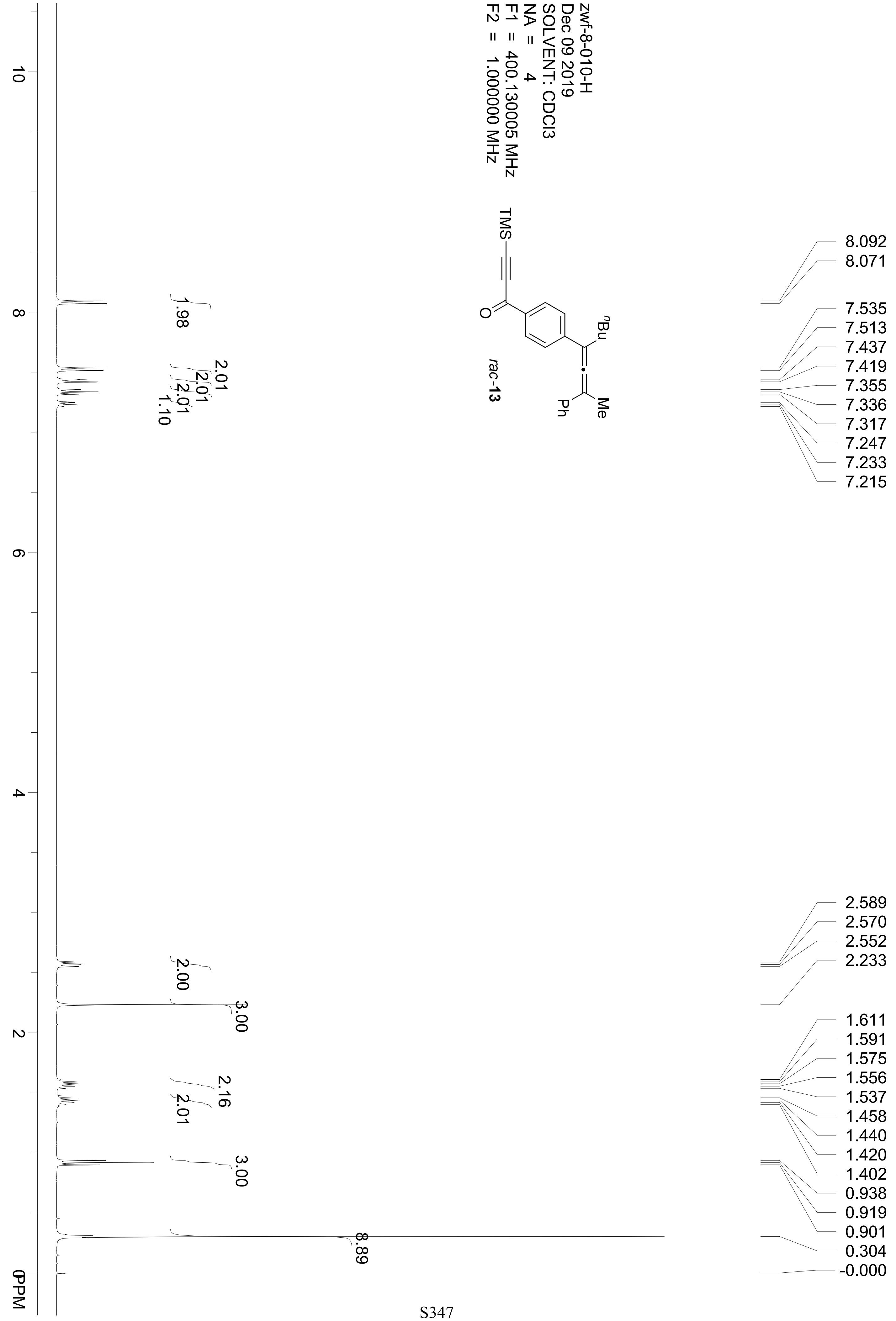

Supplementary Figure 275. ^{1}H NMR (400 MHz, $CDCl_3$) spectrum for *rac*-**13**

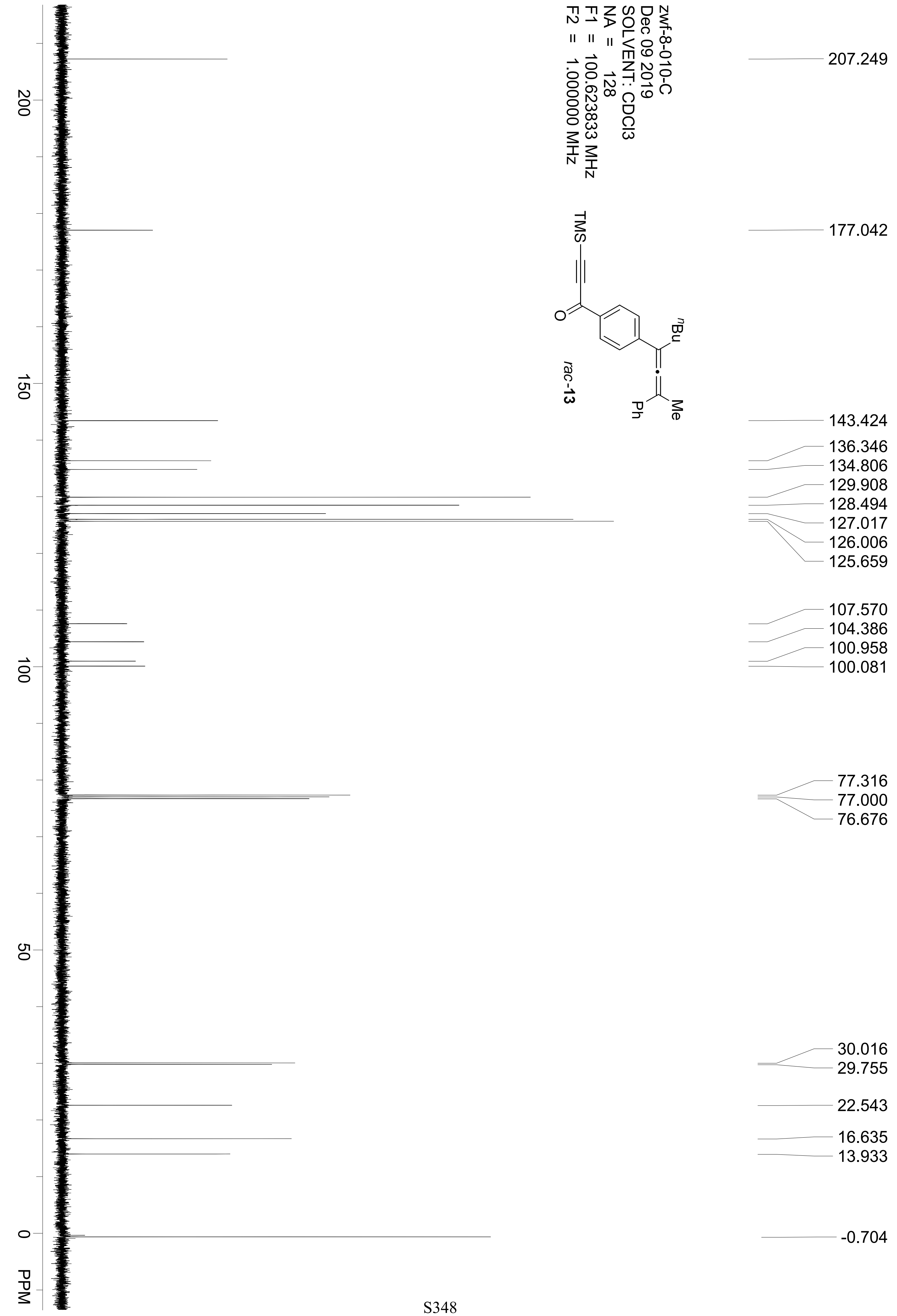

Supplementary Figure 276. ^{13}C NMR (100 MHz, $CDCl_3$) spectrum for *rac*-**13**

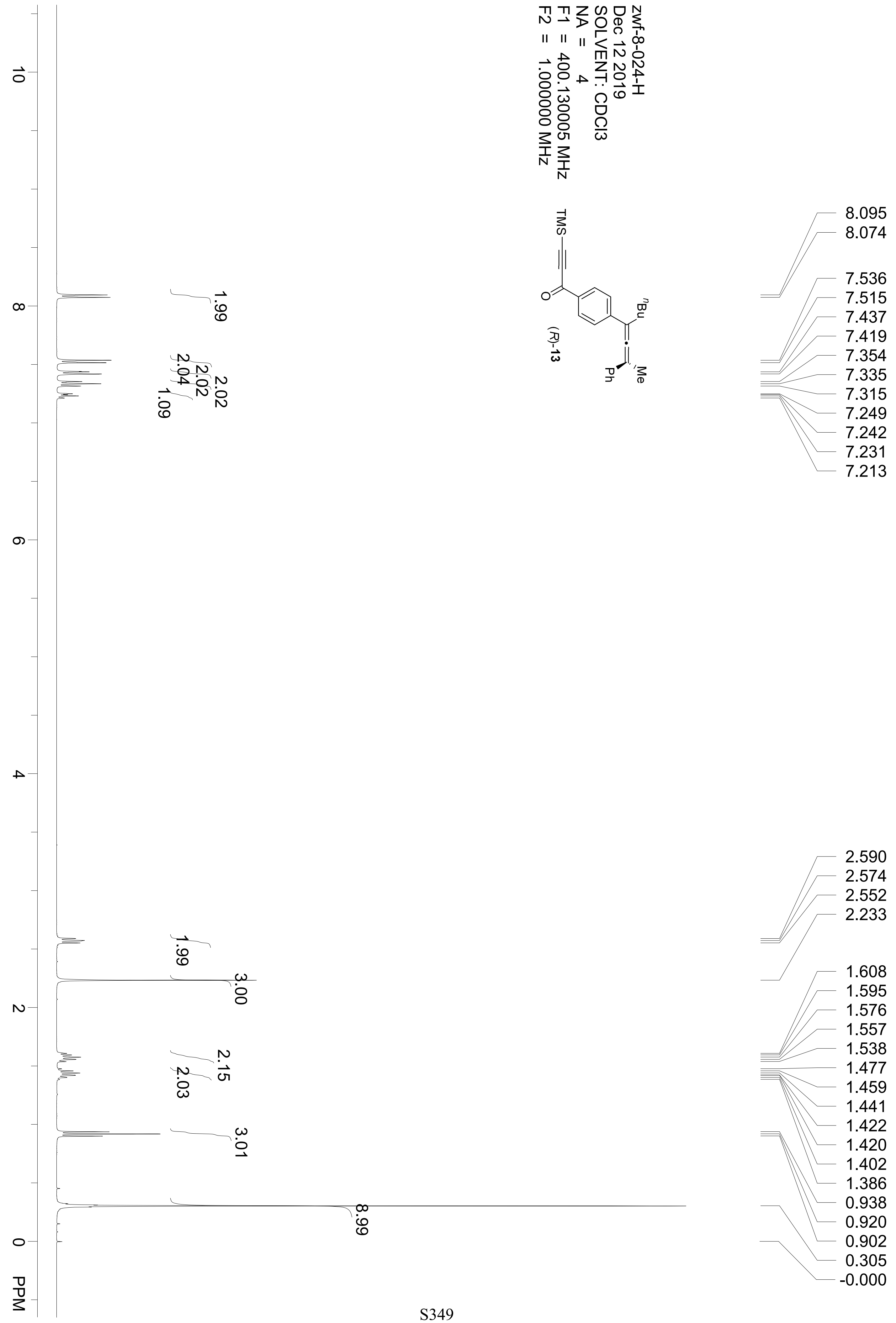

Supplementary Figure 277. ^{1}H NMR (400 MHz, $CDCl_3$) spectrum for (*R*)-**13**

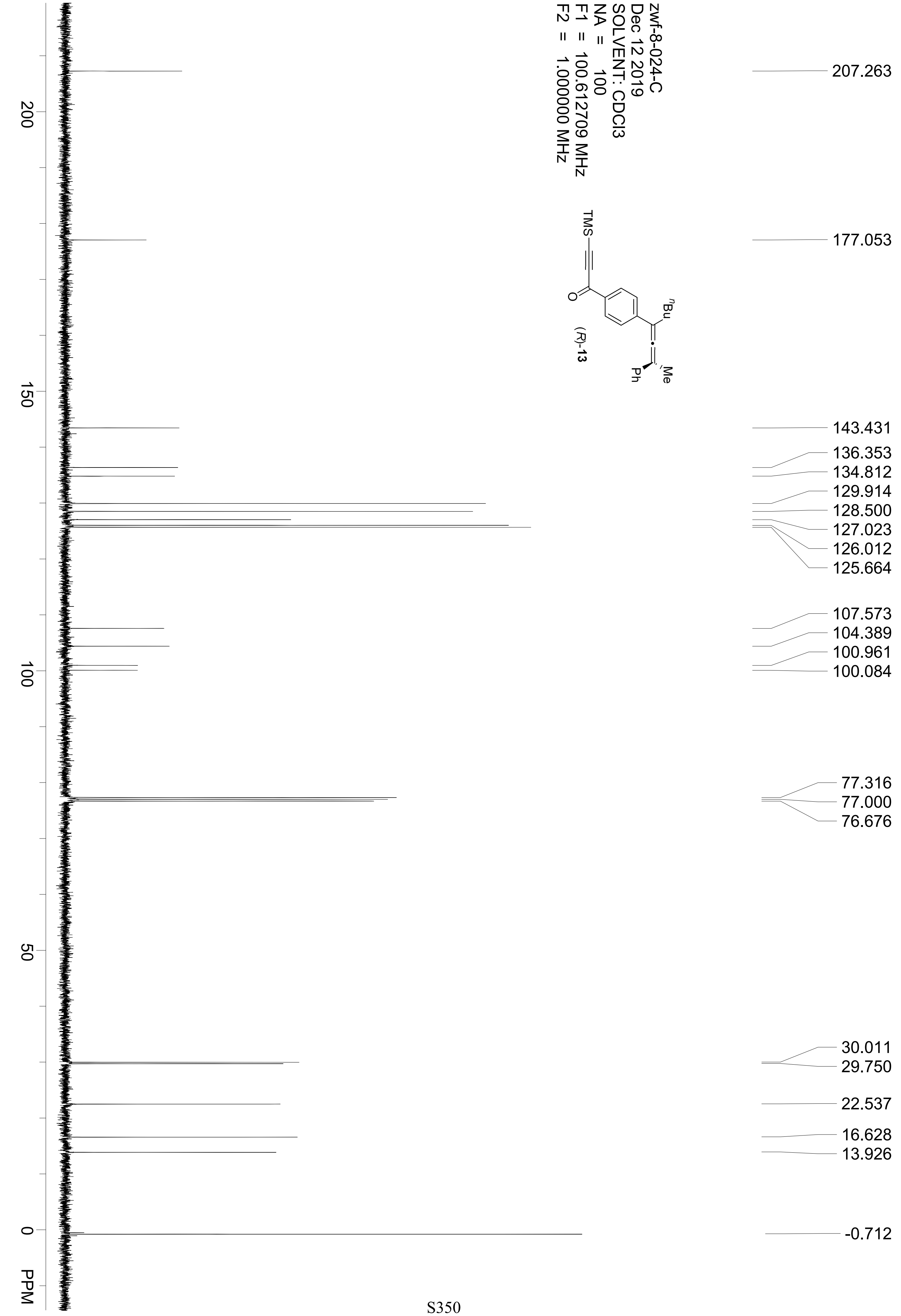

Supplementary Figure 278. ^{13}C NMR (100 MHz, $CDCl_3$) spectrum for (*R*)-**13**

Area Percent Report

Agilent Technologies

sample zwf-8-024-OD-H-99.5-0.5-1.0-214

Data file: C:\Users\Public\Documents\ChemStation\1\Data\zwf-allenioc acid_LC 2019-12-12 13-00-00\003-P1-C1-zwf-8-024.D

Acquisition Data:

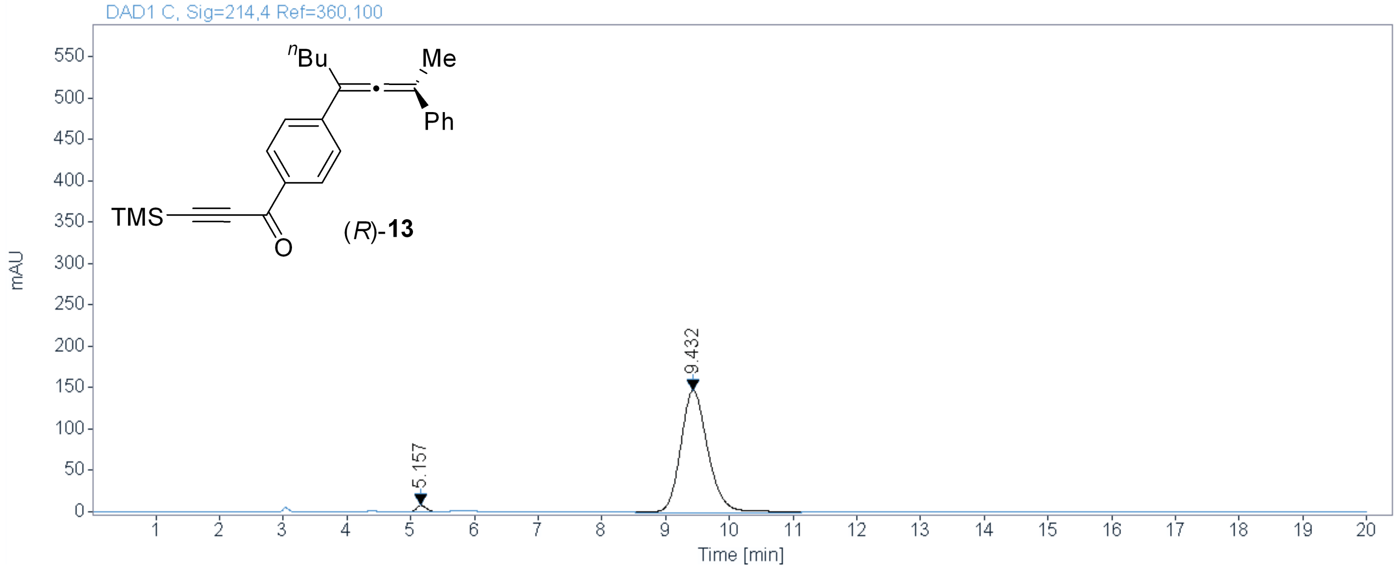

Signal: DAD1 C, Sig=214,4 Ref=360,100

RT [min]	Width [min]	Height	Area	Area%
5.157	0.1639	7.2866	71.6506	1.5416
9.432	0.5128	148.7195	4576.0190	98.4584
		Sum	4647.6696	100.0000

Supplementary Figure 279. HPLC spectrum for (*R*)-**13**

Area Percent Report

sample zwf-8-024-rac-OD-H-99.5-0.5-1.0-214

Data file: C:\Users\Public\Documents\ChemStation\1\Data\zwf-allenioc acid_LC 2019-12-12 13-00-00\004-P1-C2-zwf-8-024-rac.D

Acquisition Data:

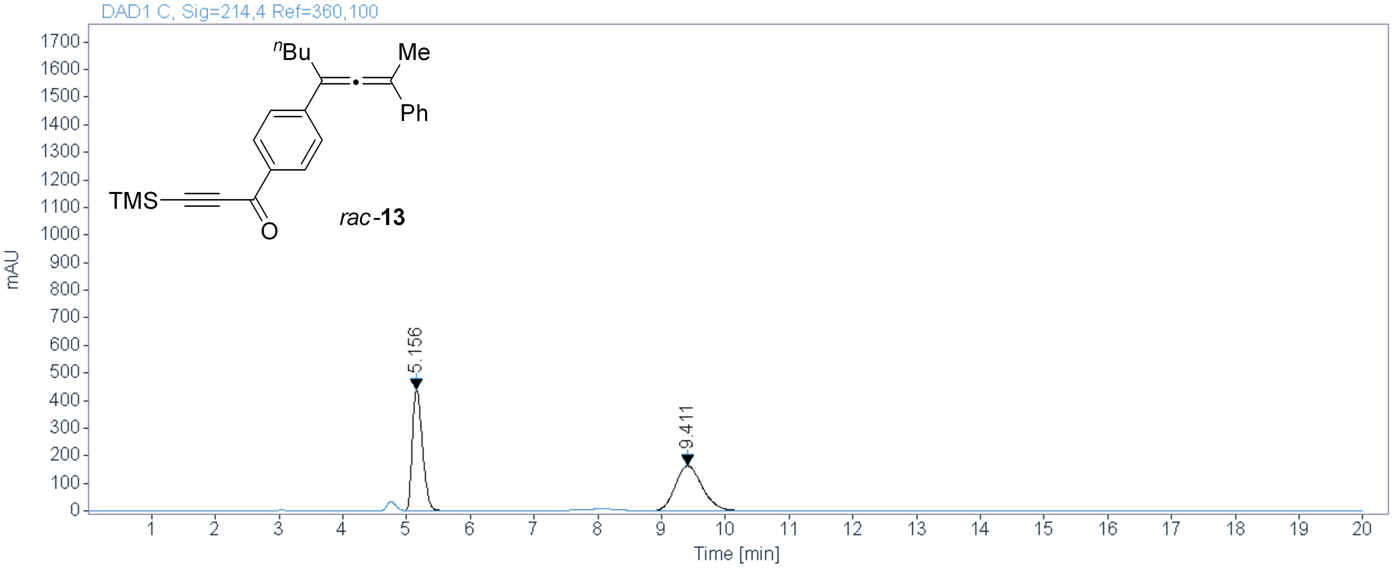

Signal: DAD1 C, Sig=214,4 Ref=360,100

RT [min]	Width [min]	Height	Area	Area%
5.156	0.1841	440.6638	4867.4404	50.0387
9.411	0.4607	163.5604	4859.9038	49.9613
		Sum	9727.3442	100.0000

Supplementary Figure 280. HPLC spectrum for *rac*-**13**

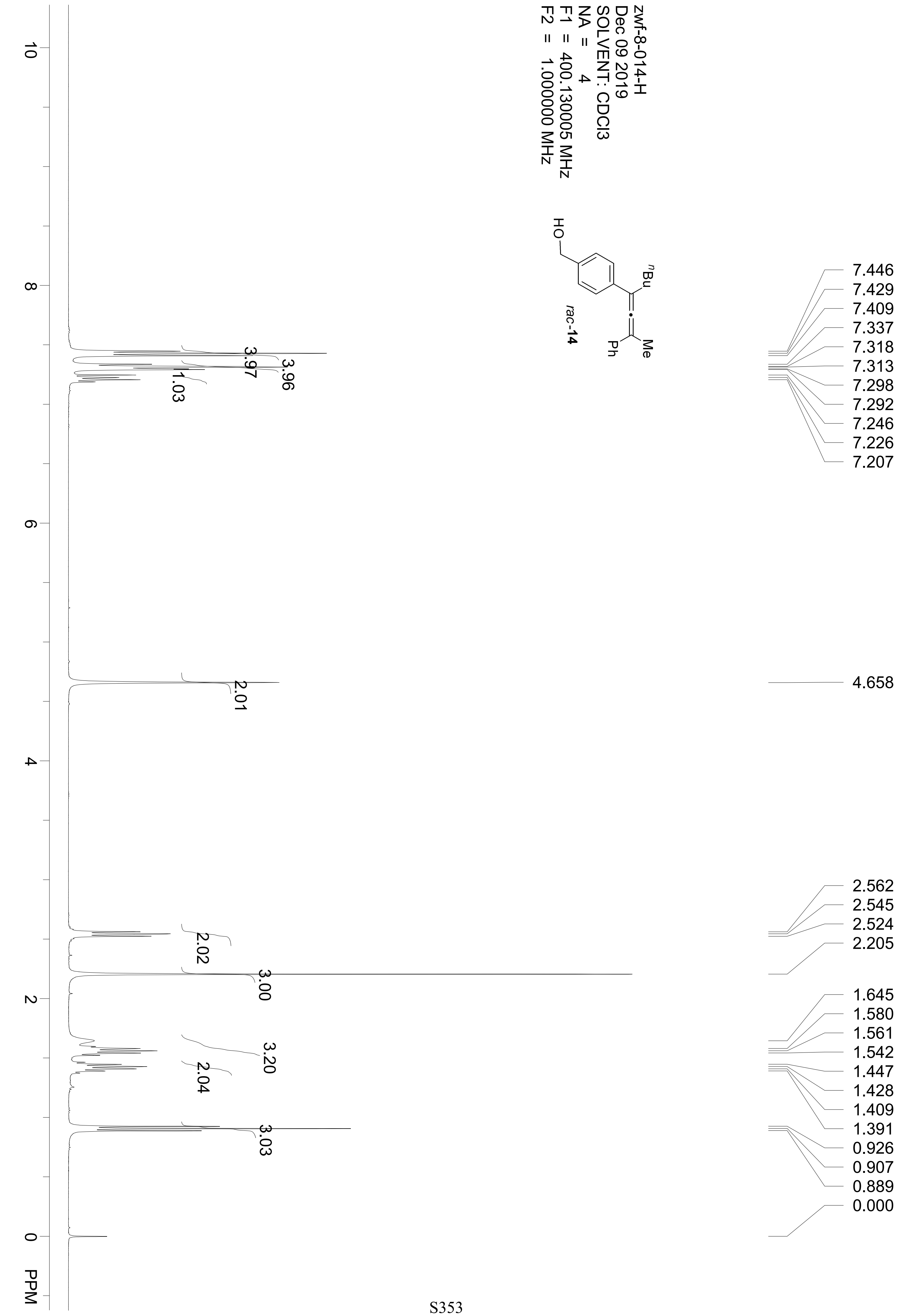

Supplementary Figure 281. ^{1}H NMR (400 MHz, $CDCl_3$) spectrum for *rac*-**14**

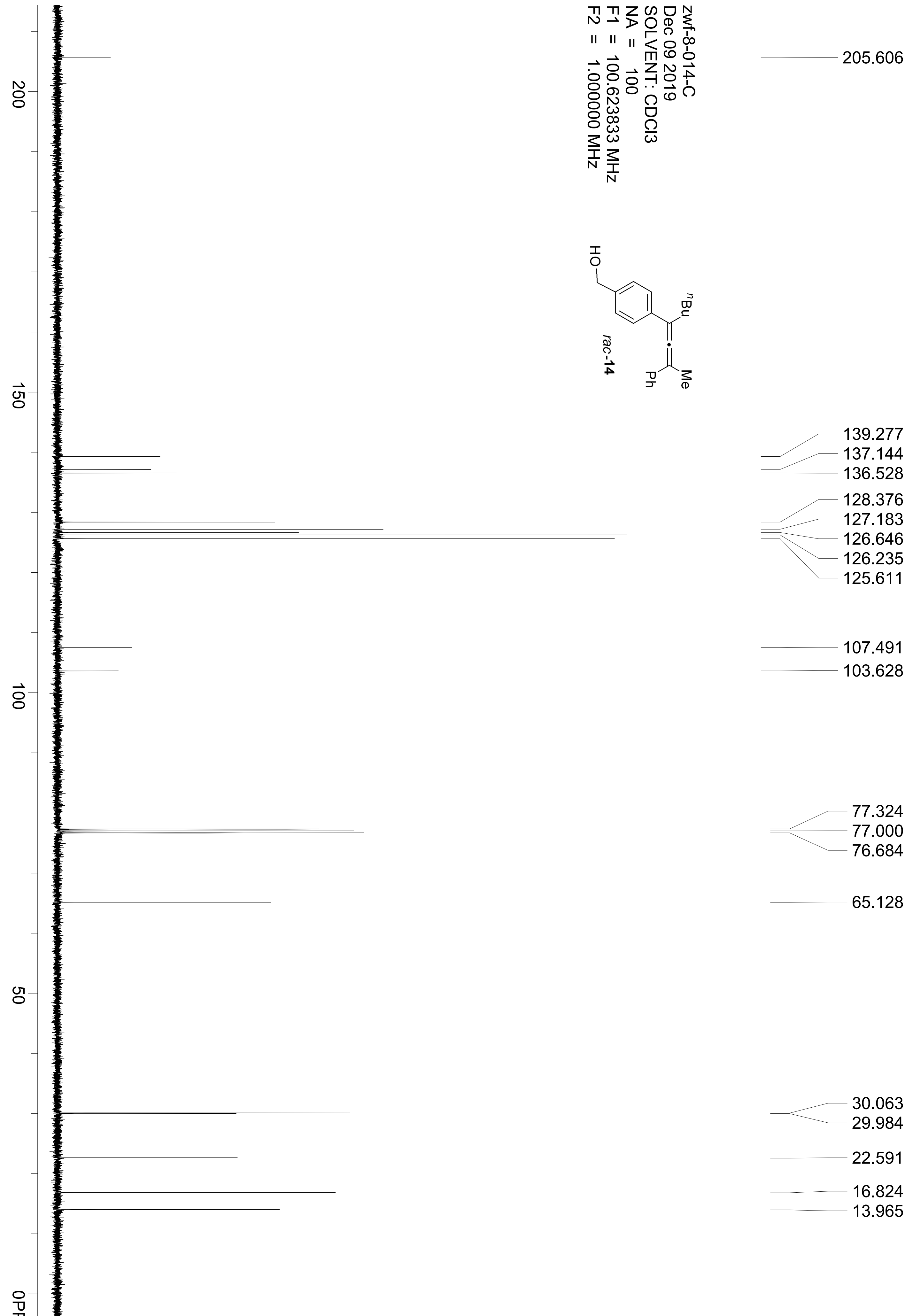

Supplementary Figure 282. ^{13}C NMR (100 MHz, $CDCl_3$) spectrum for *rac*-**14**

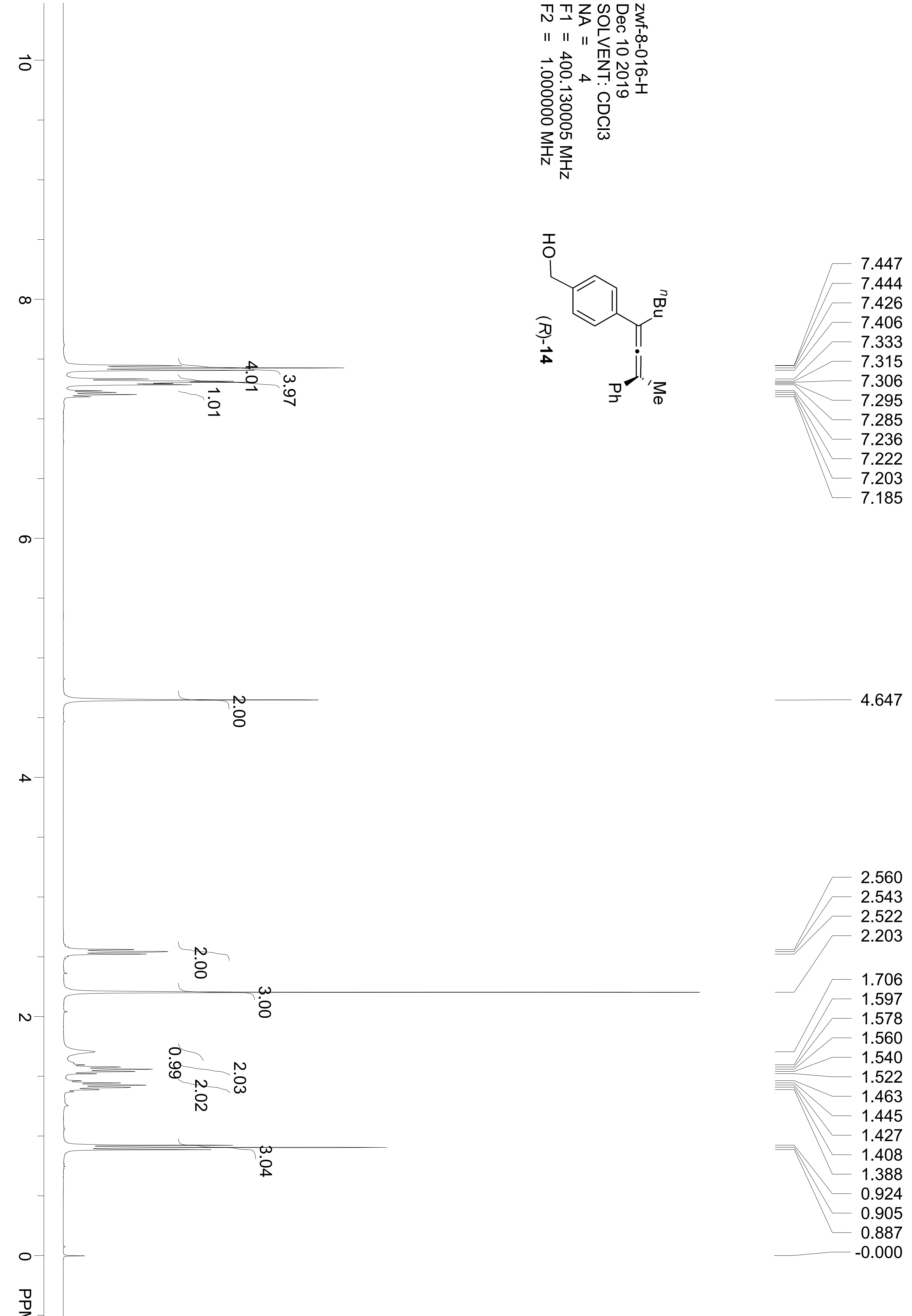

Supplementary Figure 283. ^{1}H NMR (400 MHz, $CDCl_3$) spectrum for (*R*)-**14**

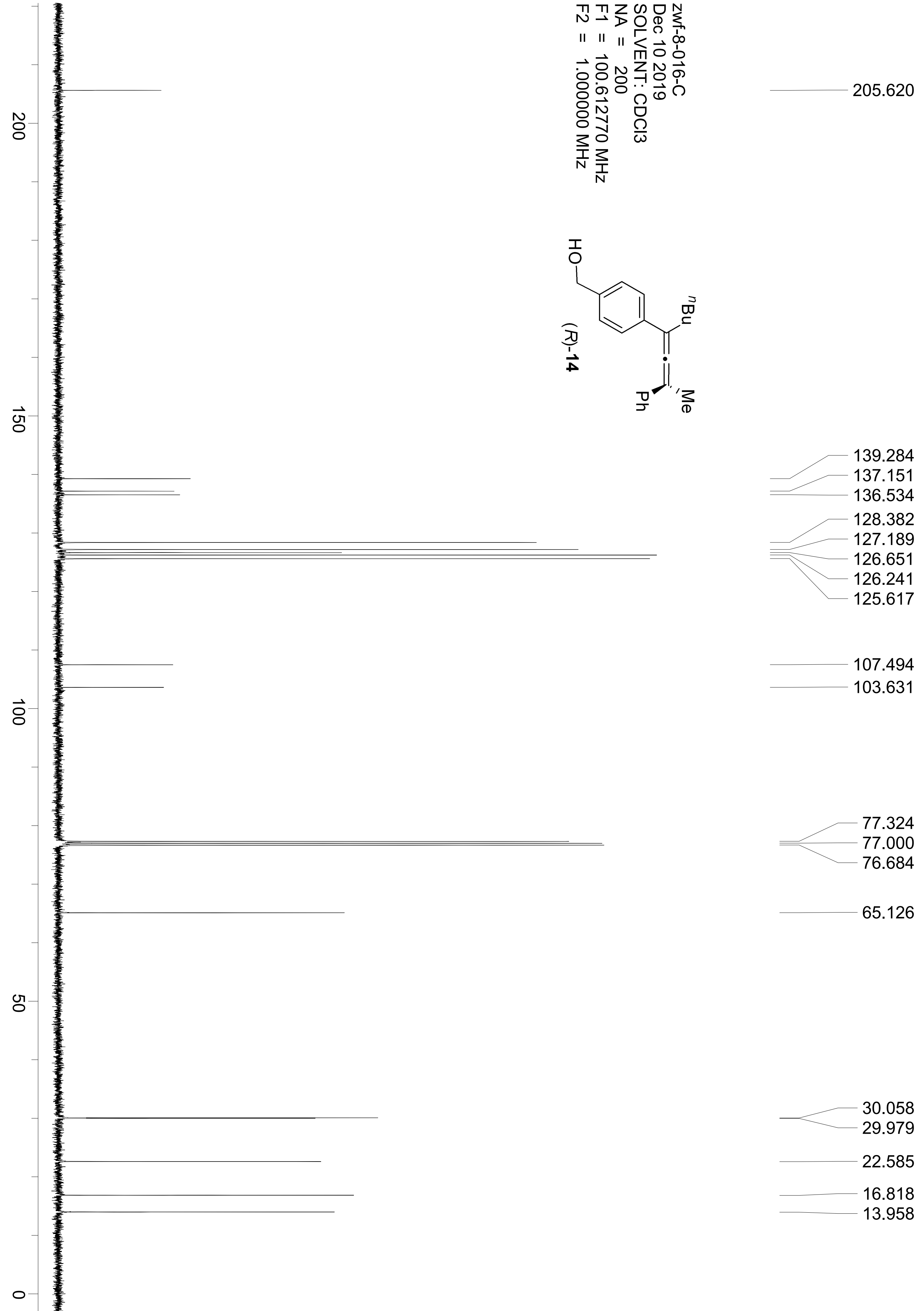

Supplementary Figure 284. ^{13}C NMR (100 MHz, $CDCl_3$) spectrum for (*R*)-**14**

Area Percent Report

Agilent Technologies

sample zwf-8-016-OD-H-98-2-1.0-214

Data file: C:\Users\Public\Documents\ChemStation\1\Data\zwf-allenioc acid_LC 2019-12-10 09-07-15\033-P1-C1-zwf-8-016.D

Acquisition Data:

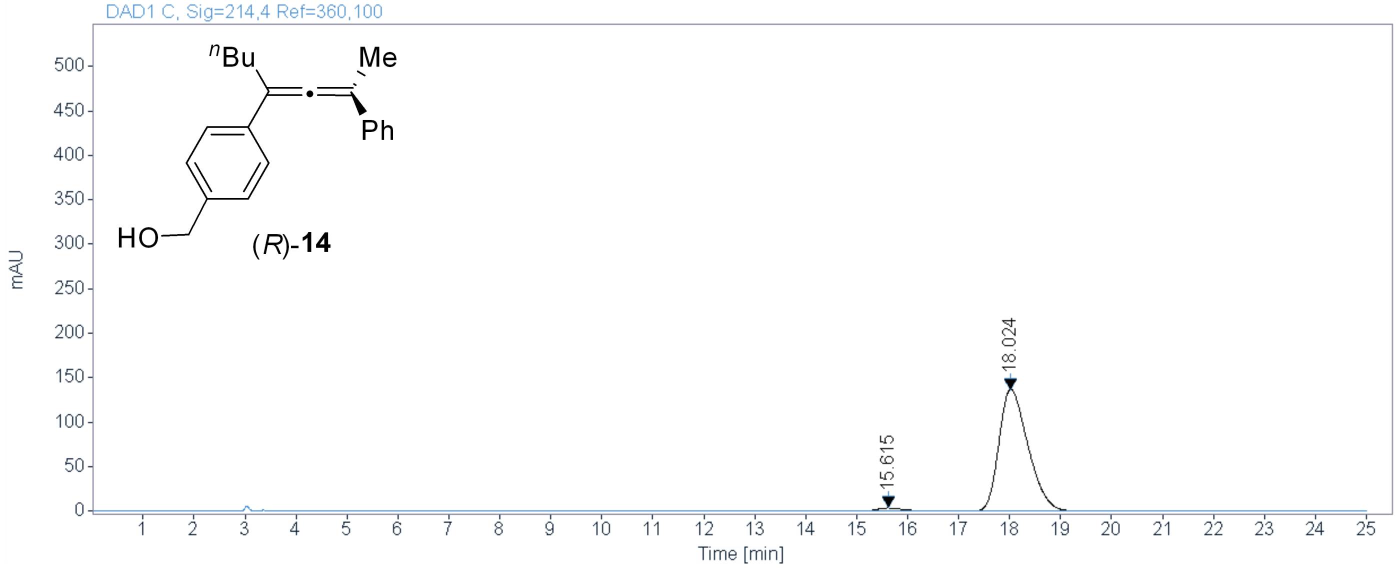

Signal: DAD1 C, Sig=214,4 Ref=360,100

RT [min]	Width [min]	Height	Area	Area%
15.615	0.5118	3.4172	115.3319	2.0885
18.024	0.6069	136.8792	5406.9336	97.9115
		Sum	5522.2655	100.0000

Supplementary Figure 285. HPLC spectrum for (*R*)-**14**

Area Percent Report

sample zwf-8-016-rac-OD-H-98-2-1.0-214

Data file: C:\Users\Public\Documents\ChemStation\1\Data\zwf-allenioc acid_LC 2019-12-10 09-07-15\034-P1-C2-zwf-8-016-rac.D

Acquisition Data:

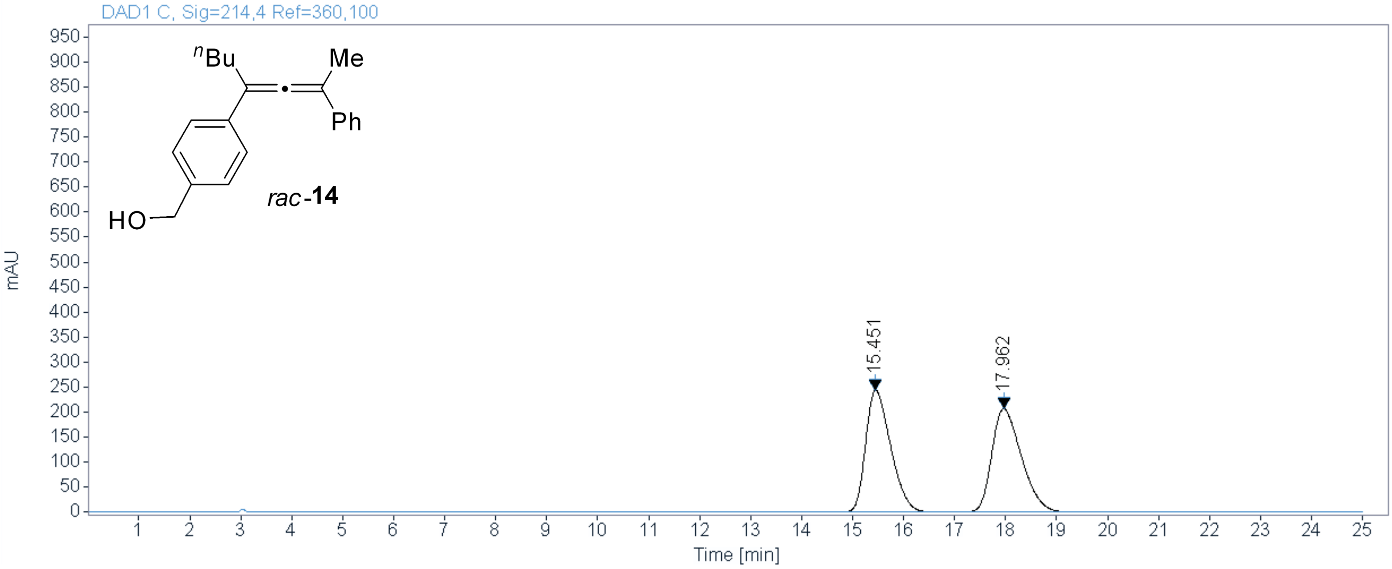

Signal: DAD1 C, Sig=214,4 Ref=360,100

RT [min]	Width [min]	Height	Area	Area%
15.451	0.5272	244.0662	8310.0879	50.0145
17.962	0.6211	206.5406	8305.2725	49.9855
		Sum	16615.3604	100.0000

Supplementary Figure 286. HPLC spectrum for *rac*-**14**

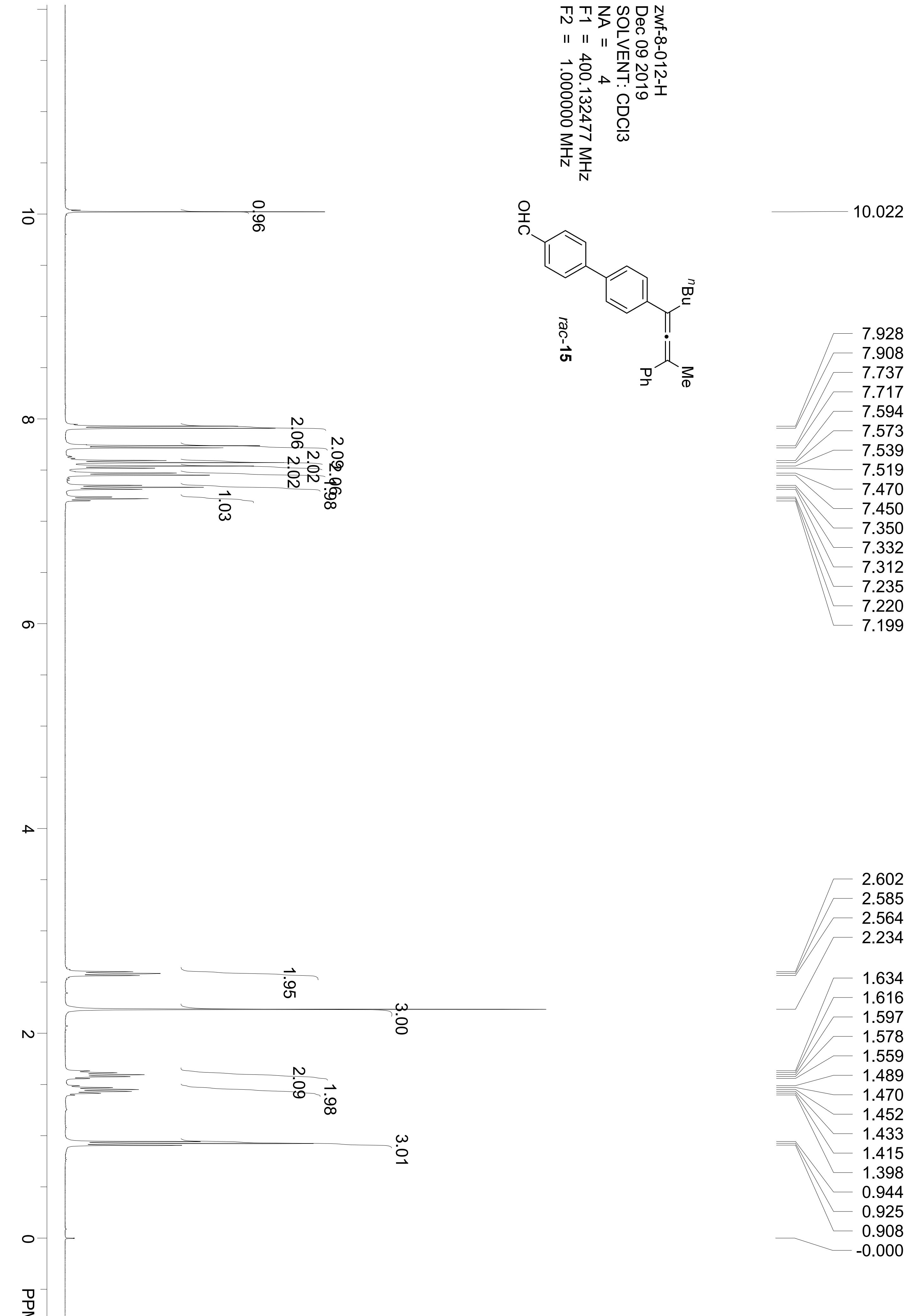

Supplementary Figure 287. ^{1}H NMR (400 MHz, $CDCl_3$) spectrum for *rac*-**15**

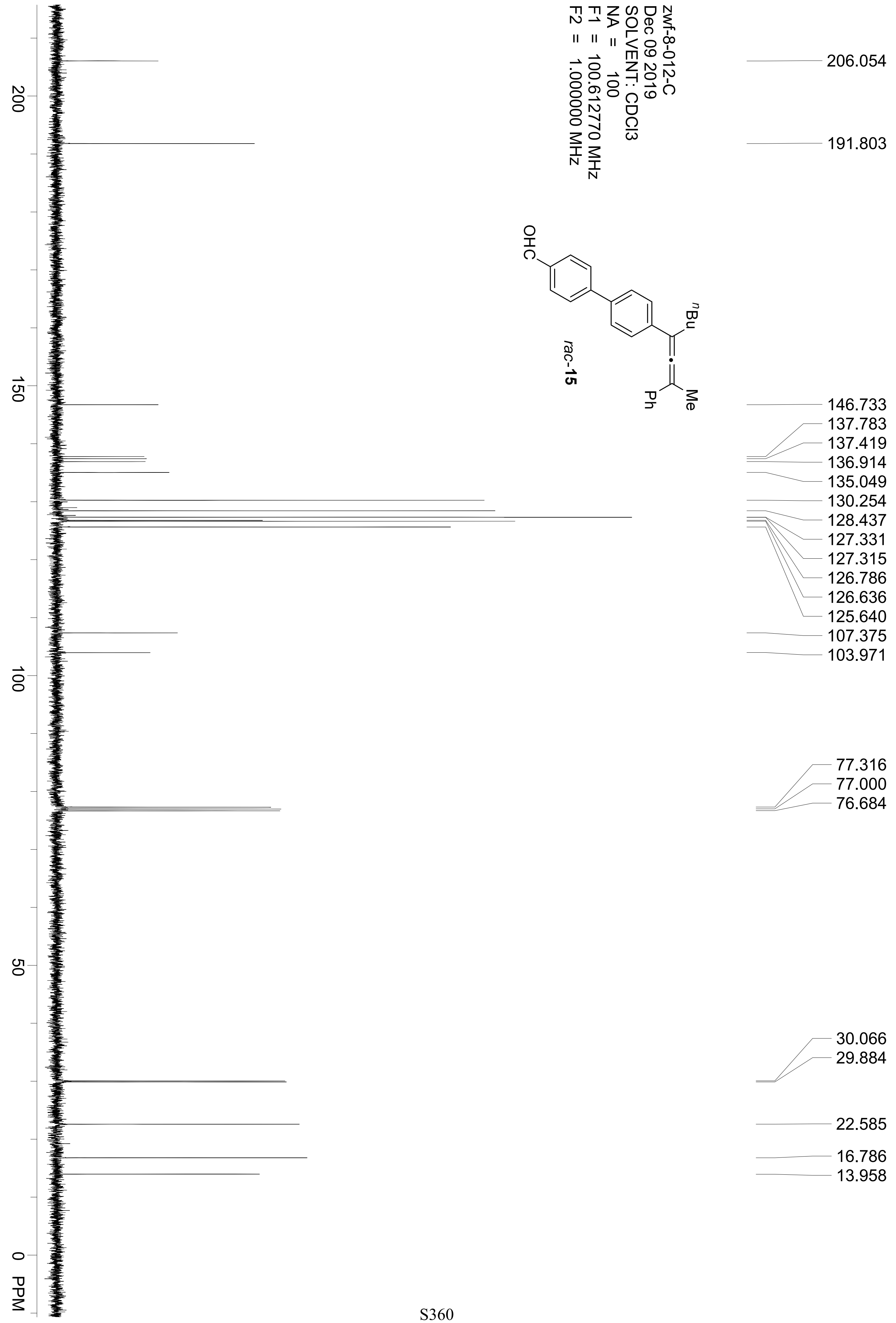

Supplementary Figure 288. ^{13}C NMR (100 MHz, $CDCl_3$) spectrum for *rac*-**15**

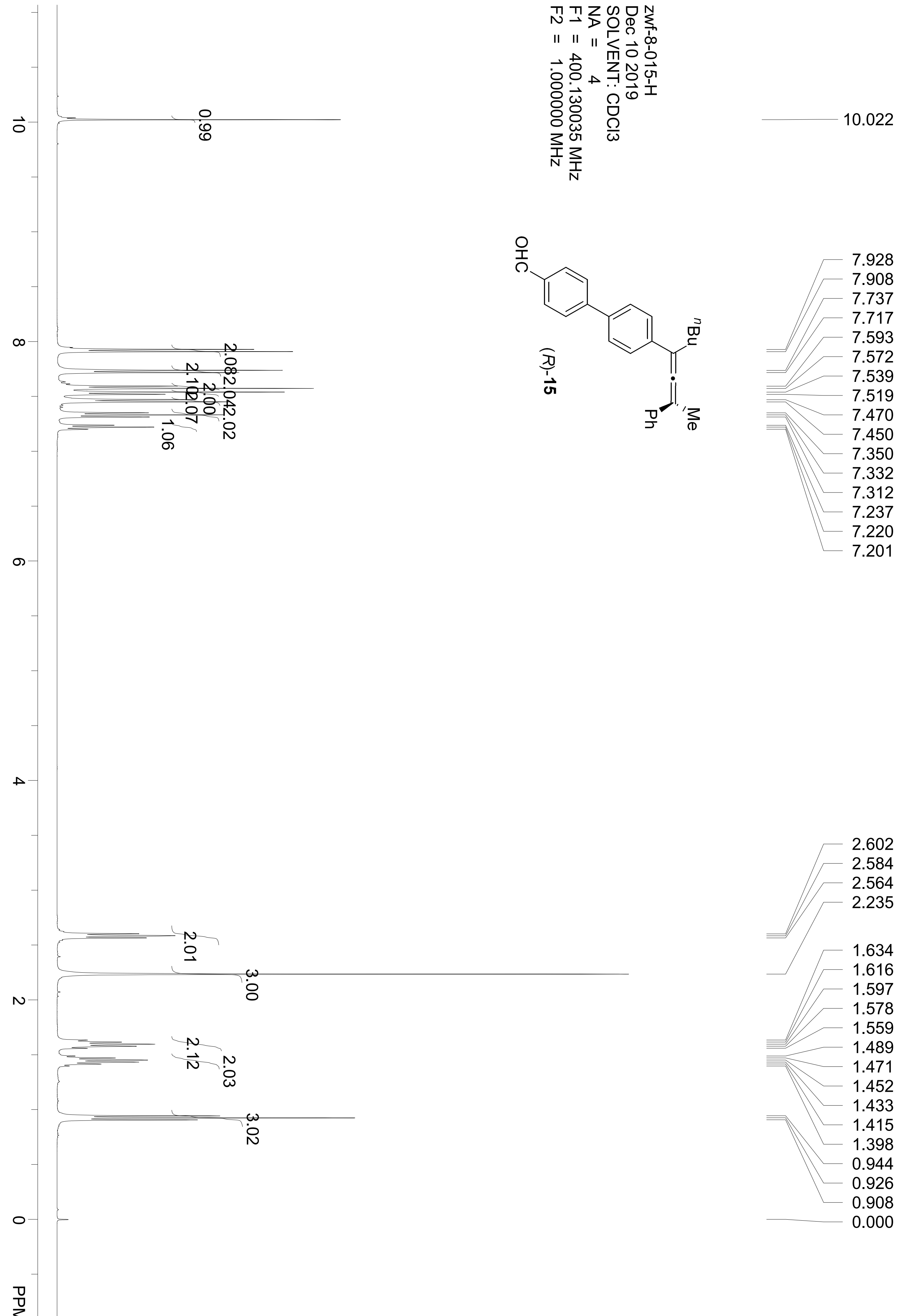

Supplementary Figure 289. ^{1}H NMR (400 MHz, $CDCl_3$) spectrum for (*R*)-**15**

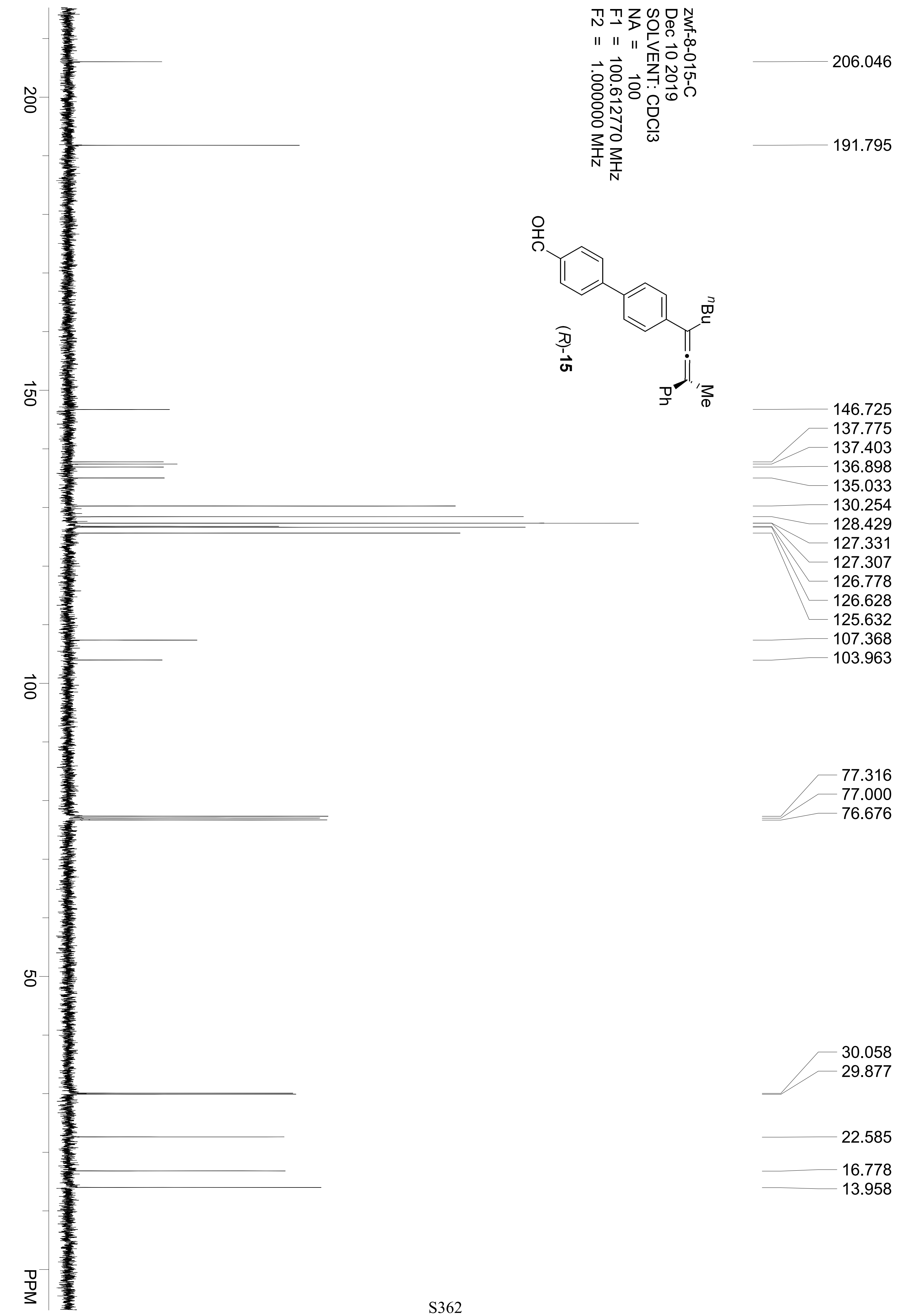

Supplementary Figure 290. ^{13}C NMR (100 MHz, $CDCl_3$) spectrum for (*R*)-**15**

Default Individual Report

SAMPLE INFORMATION

Sample Name:	zwf-8-015-chiral	Acquired By:	System
Sample Type:	Unknown	Sample Set Name:	
Vial:	2:F,3	Acq. Method Set:	upc_pda_2019m
Injection #:	1	Processing Method	TEST
Injection Volume:	2.00 ul	Channel Name:	PDA Ch1 254nm@4.8nm
Run Time:	40.0 Minutes	Proc. Chnl. Descr.:	PDA Ch1 254nm@4.8nm
Date Acquired:	12/17/2019 2:33:34 PM CST		
Date Processed:	12/18/2019 9:24:32 AM CST		

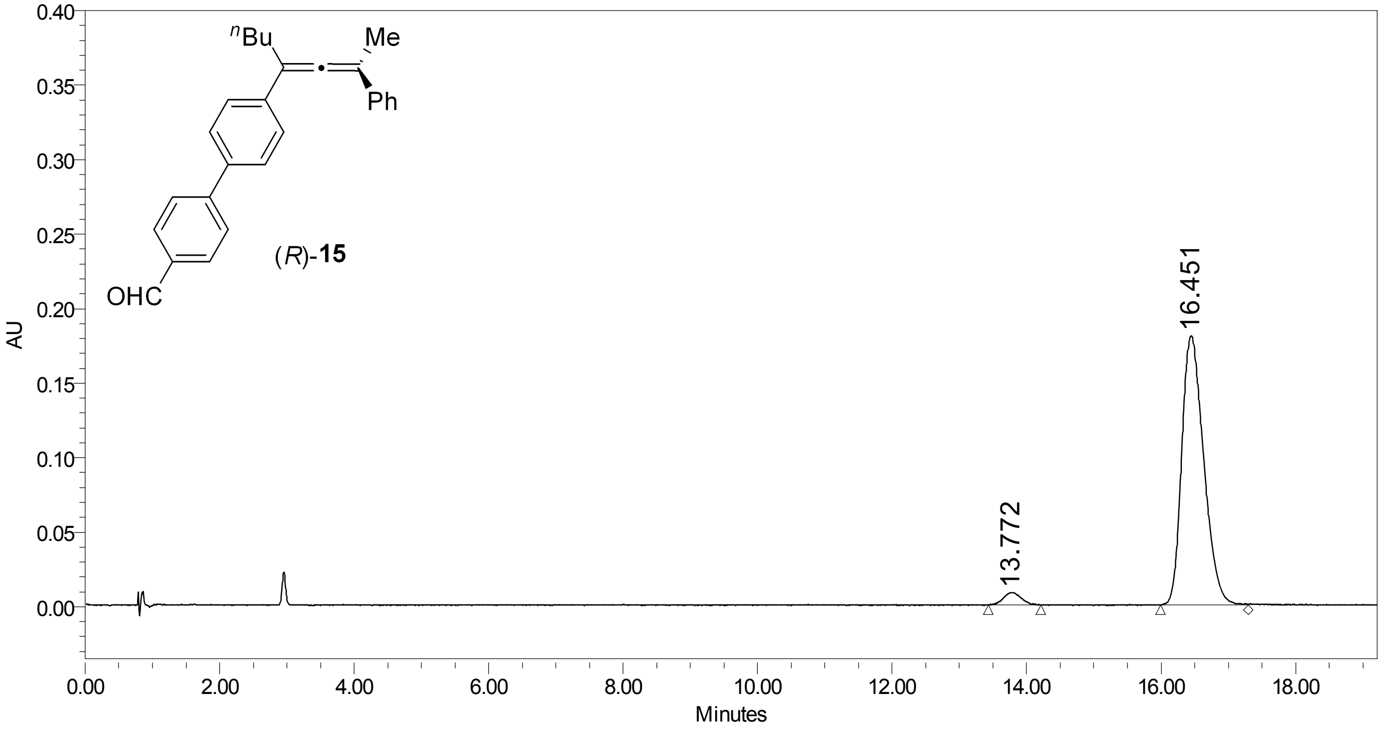

	RT	Peak Type	Height	Width (sec)	Area	% Area
1	13.772	Unknown	8417	46.700	155152	3.66
2	16.451	Unknown	180667	78.200	4078920	96.34

Supplementary Figure 291. SFC spectrum for (*R*)-**15**

SAMPLE INFORMATION

Sample Name:	zwf-8-12-rac-ad-3-ipa	Acquired By:	System
Sample Type:	Unknown	Sample Set Name:	
Vial:	2:F,1	Acq. Method Set:	upc_pda_2019m
Injection #:	1	Processing Method	TEST
Injection Volume:	2.00 ul	Channel Name:	PDA Ch1 254nm@4.8nm
Run Time:	40.0 Minutes	Proc. Chnl. Descr.:	PDA Ch1 254nm@4.8nm
Date Acquired:	12/17/2019 1:55:54 PM CST		
Date Processed:	12/18/2019 9:24:14 AM CST		

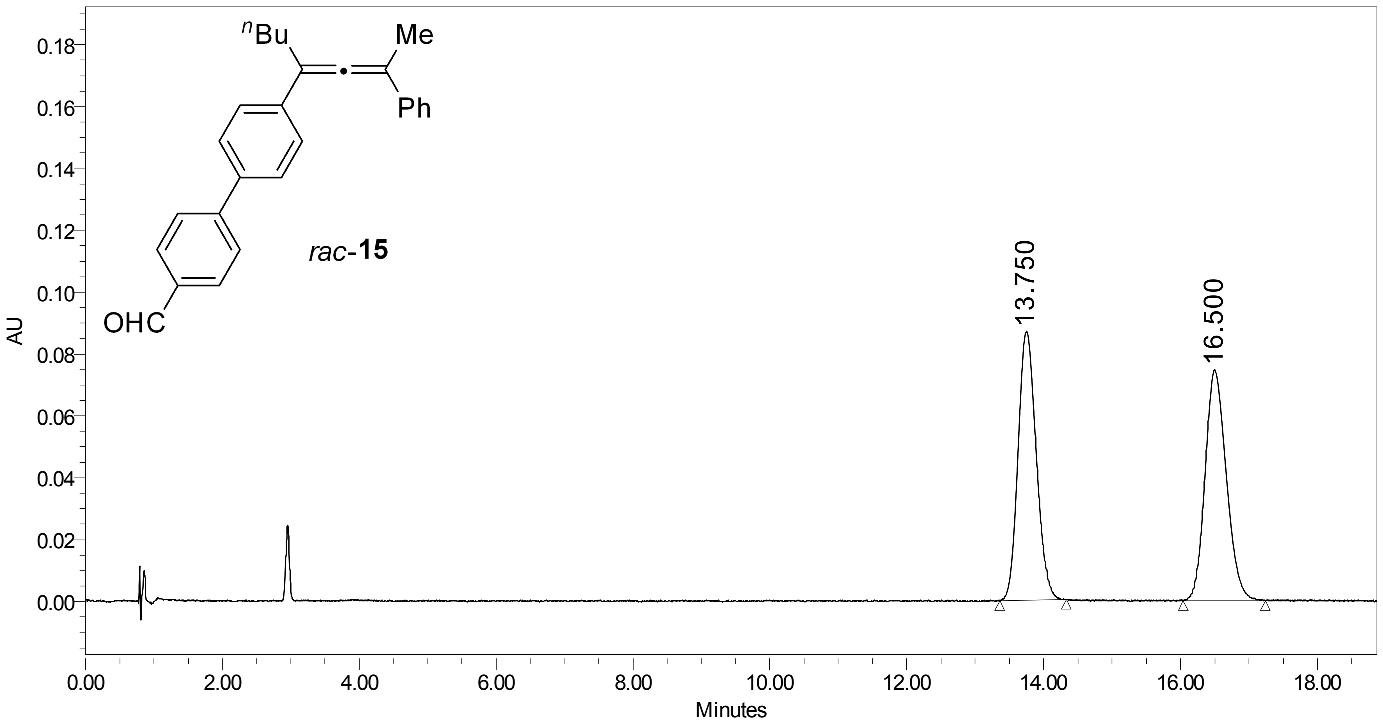

	RT	Peak Type	Height	Width (sec)	Area	% Area
1	13.750	Unknown	87045	58.300	1614293	49.92
2	16.500	Unknown	74554	72.200	1619207	50.08

Supplementary Figure 292. SFC spectrum for *rac*-**15**

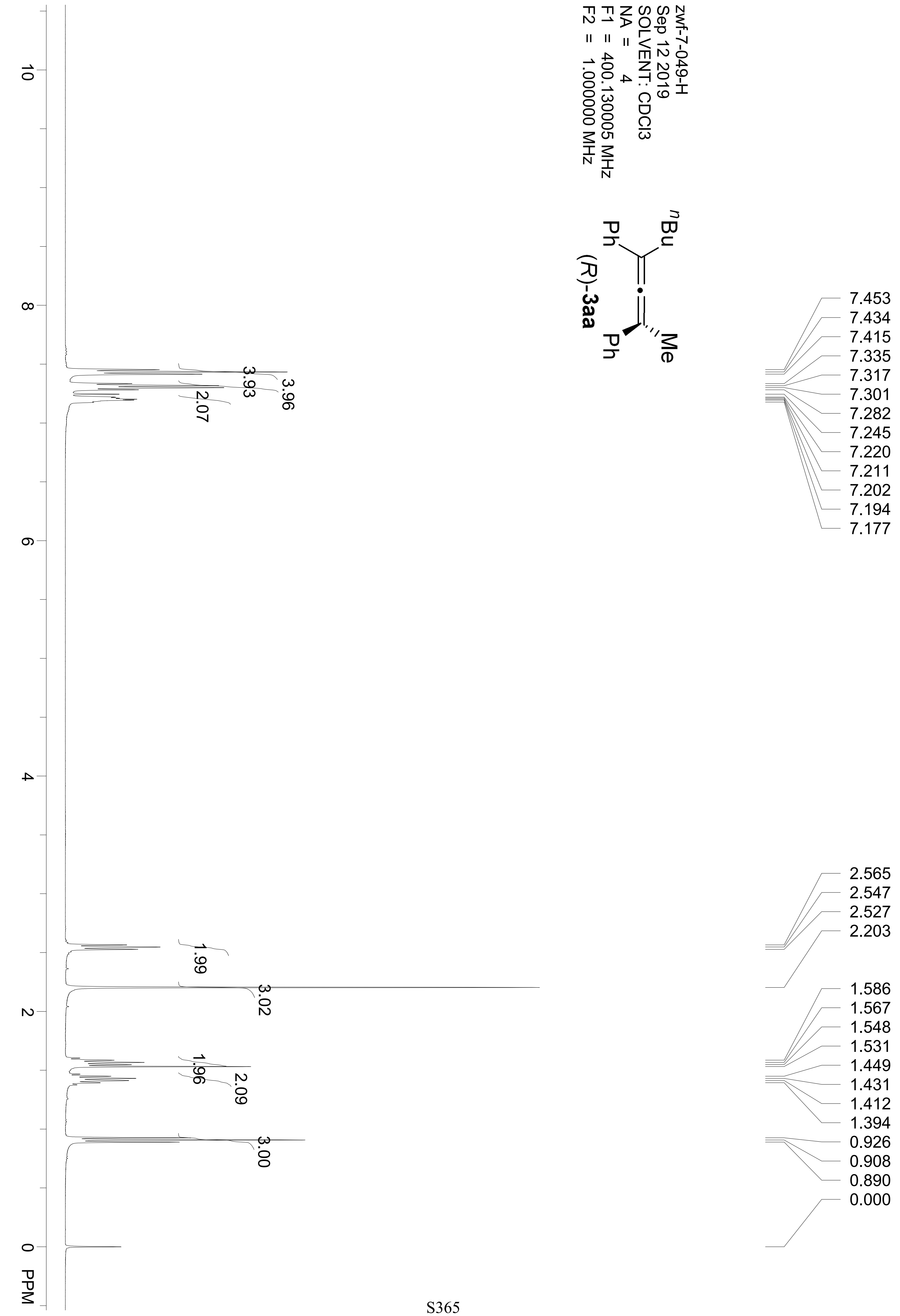

Supplementary Figure 293. ^{1}H NMR (400 MHz, $CDCl_3$) spectrum for (*R*)-**3aa**

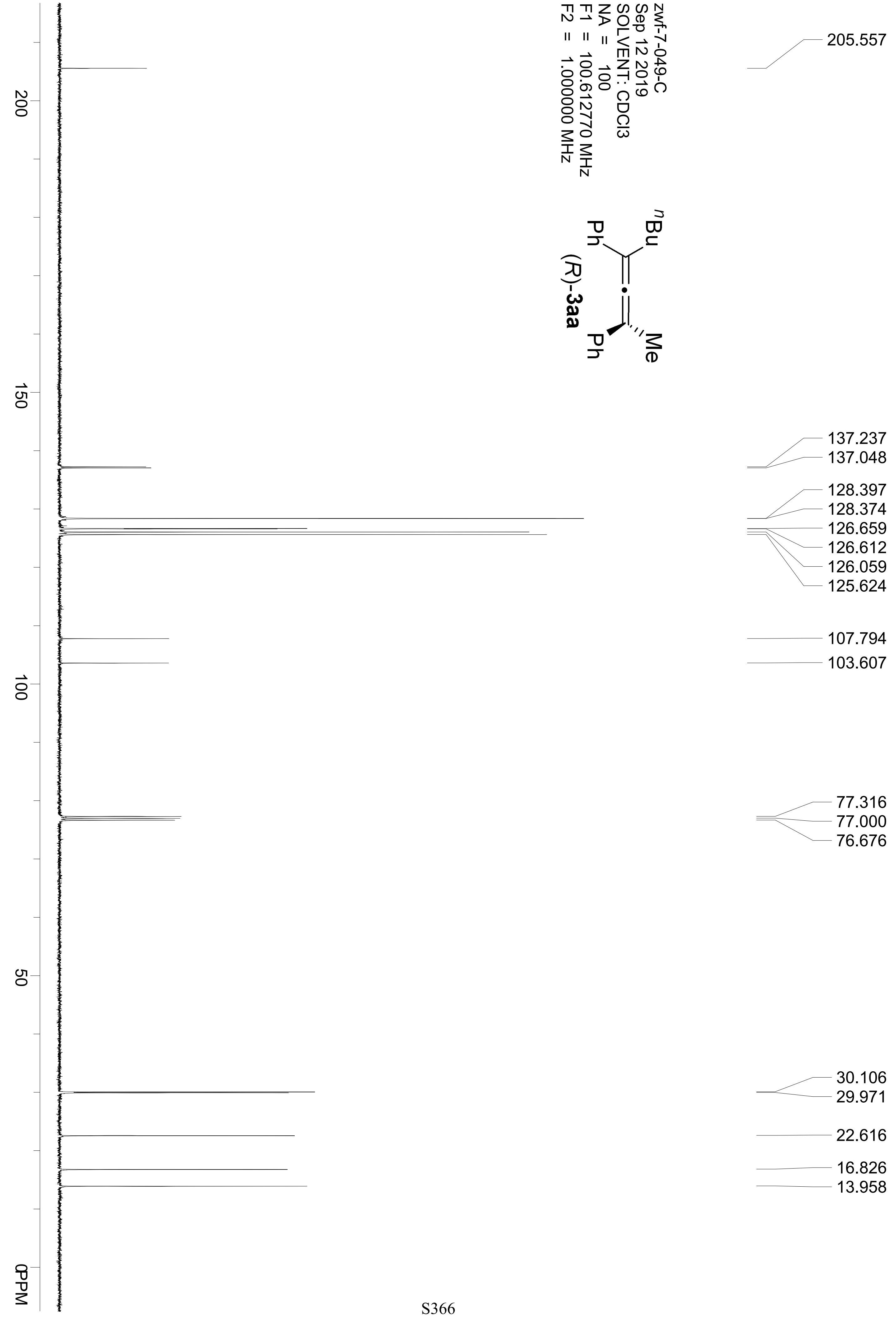

Supplementary Figure 294. ^{13}C NMR (100 MHz, $CDCl_3$) spectrum for (*R*)-**3aa**

Area Percent Report

Agilent Technologies

sample zwf-7-049-OJ-H-99.5-0.5-0.7-214

Data file: C:\Users\Public\Documents\ChemStation\1\Data\zwf-allenioc acid_LC 2019-09-11 21-59-05\003-P1-C1-zwf-7-049.D

Acquisition Data:

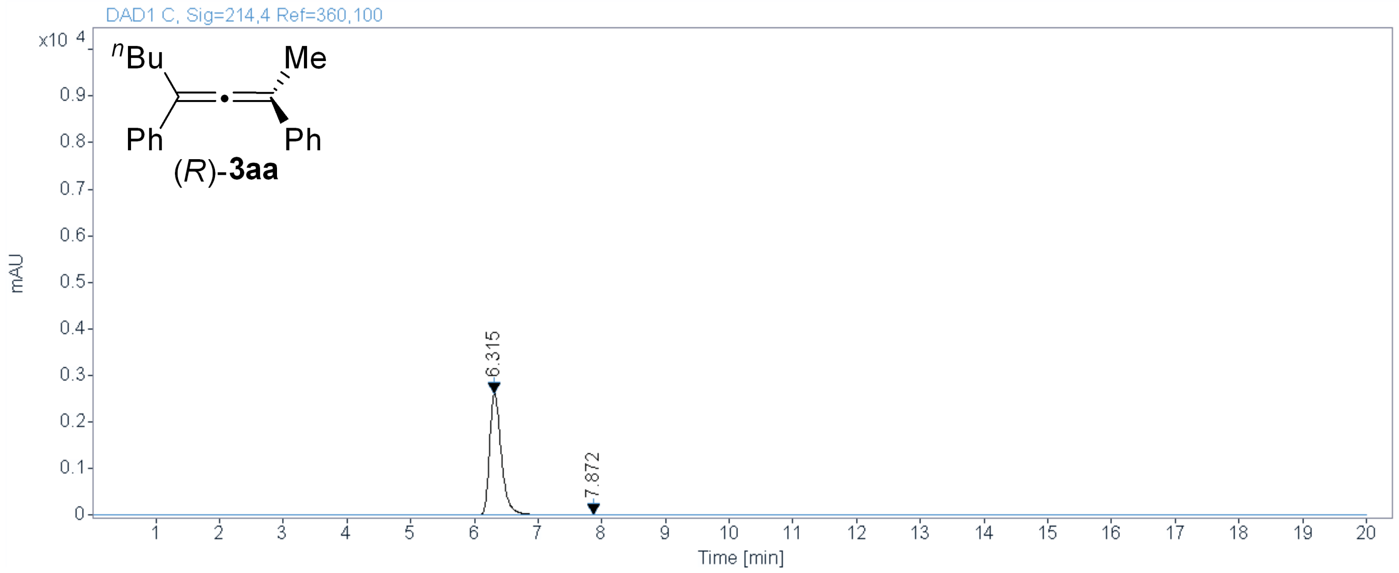

Signal: DAD1 C, Sig=214,4 Ref=360,100

RT [min]	Width [min]	Height	Area	Area%
6.315	0.1987	2620.1880	33834.6055	99.3373
7.872	0.3684	10.2114	225.7029	0.6627
		Sum	34060.3084	100.0000

Supplementary Figure 295. HPLC spectrum for (*R*)-**3aa**

Area Percent Report

sample zwf-7-049-rac-OJ-H-99.5-0.5-0.7-214

Data file: C:\Users\Public\Documents\ChemStation\1\Data\zwf-allenioc acid_LC 2019-09-11 21-59-05\004-P1-C2-zwf-7-049-rac.D

Acquisition Data:

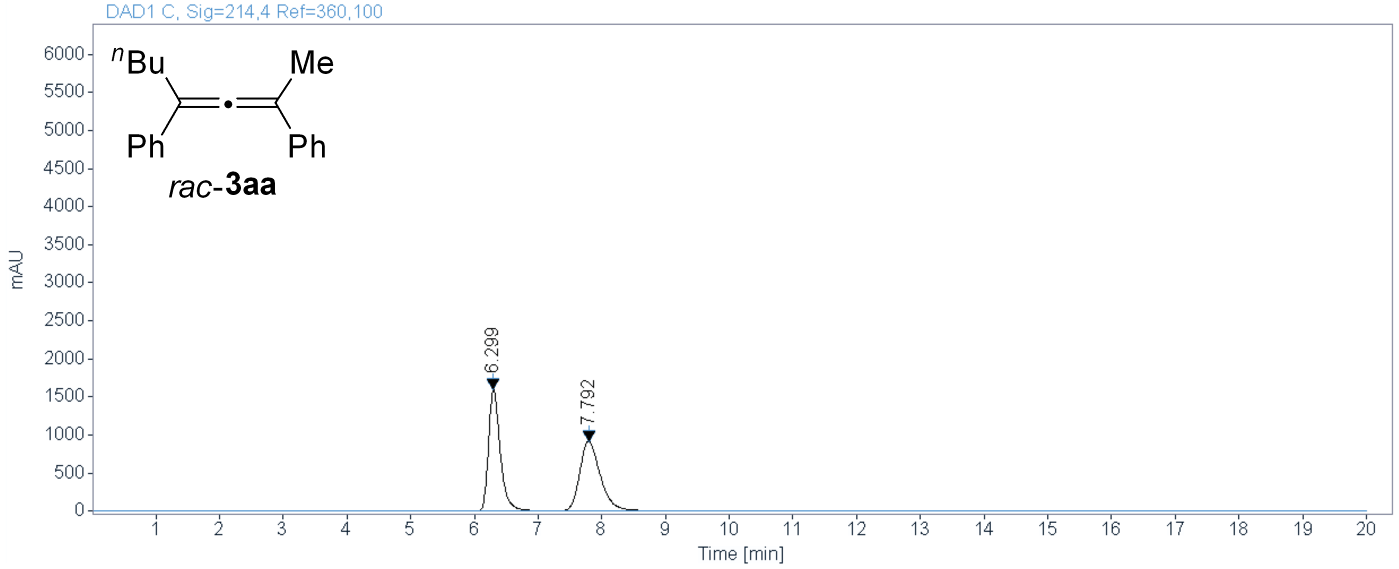

Signal: DAD1 C, Sig=214,4 Ref=360,100

RT [min]	Width [min]	Height	Area	Area%
6.299	0.1958	1599.3374	20534.9082	49.9660
7.792	0.3447	913.1305	20562.8613	50.0340
		Sum	41097.7695	100.0000

Supplementary Figure 296. HPLC spectrum for *rac*-**3aa**

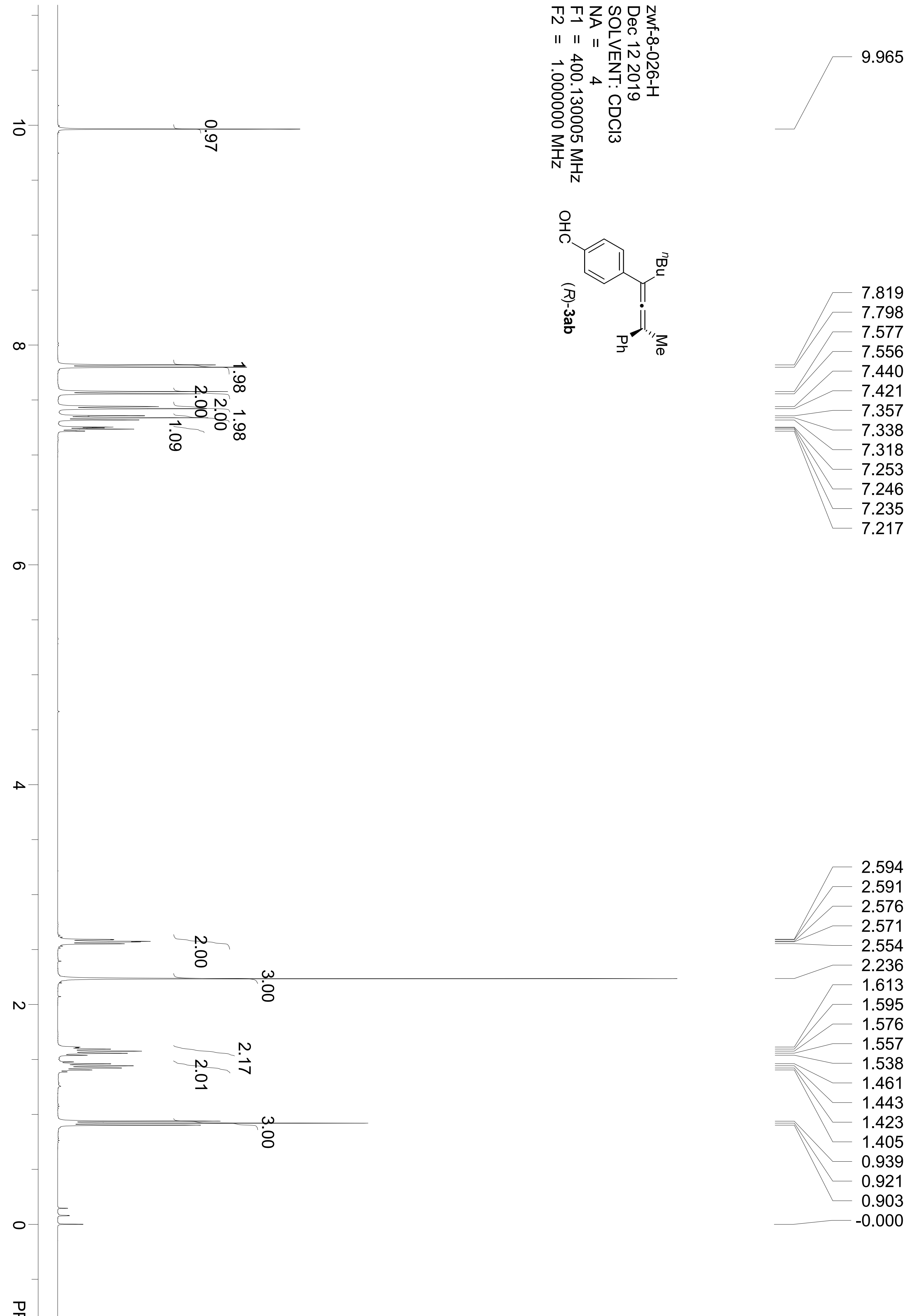

Supplementary Figure 297. ^{1}H NMR (400 MHz, $CDCl_3$) spectrum for (*R*)-**3ab**

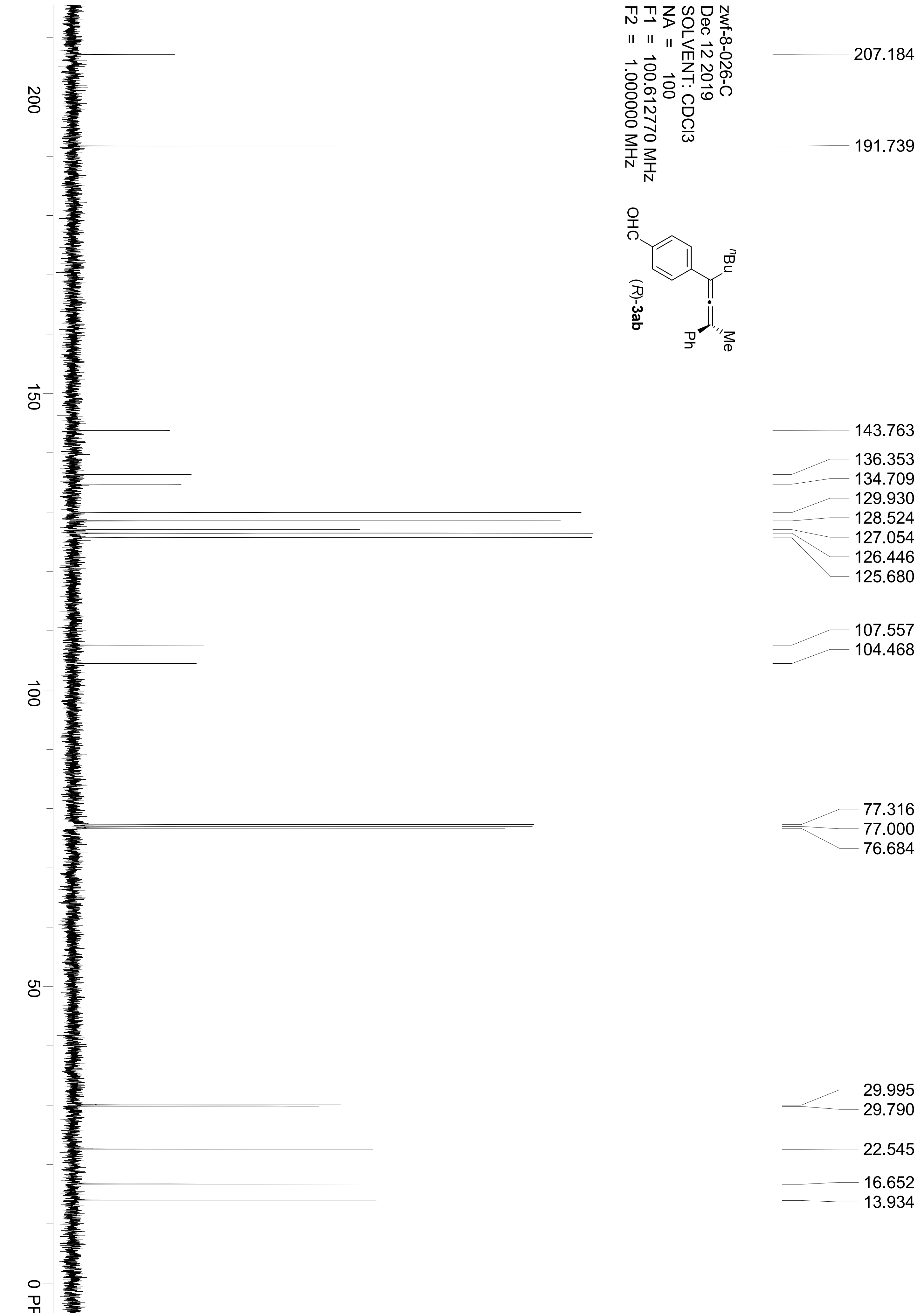

Supplementary Figure 298. ^{13}C NMR (100 MHz, $CDCl_3$) spectrum for (*R*)-**3ab**

Area Percent Report

Agilent Technologies

sample zwf-8-026-OD-H-99.5-0.5-1.0-214

Data file: C:\Users\Public\Documents\ChemStation\1\Data\SD-allenioc acid_LC 2019-12-12 16-16-43\007-P1-C1-zwf-8-026.D

Acquisition Data:

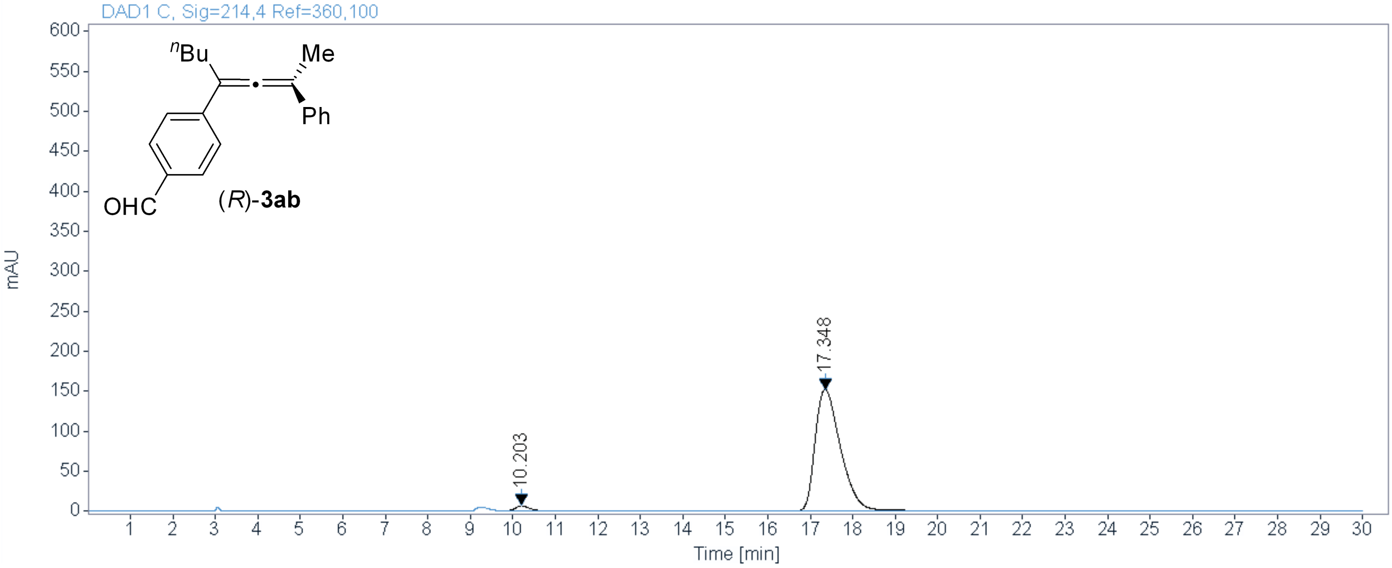

Signal: DAD1 C, Sig=214,4 Ref=360,100

RT [min]	Width [min]	Height	Area	Area%
10.203	0.3312	6.1666	132.7914	2.0715
17.348	0.6379	151.9784	6277.7349	97.9285
		Sum	6410.5263	100.0000

Supplementary Figure 299. HPLC spectrum for (*R*)-**3ab**

Area Percent Report

Agilent Technologies

sample zwf-8-026-rac-OD-H-99.5-0.5-1.0-214

Data file: C:\Users\Public\Documents\ChemStation\1\Data\SD-allenioc acid_LC 2019-12-12 16-16-43\008-P1-C2-zwf-8-026-rac.D

Acquisition Data:

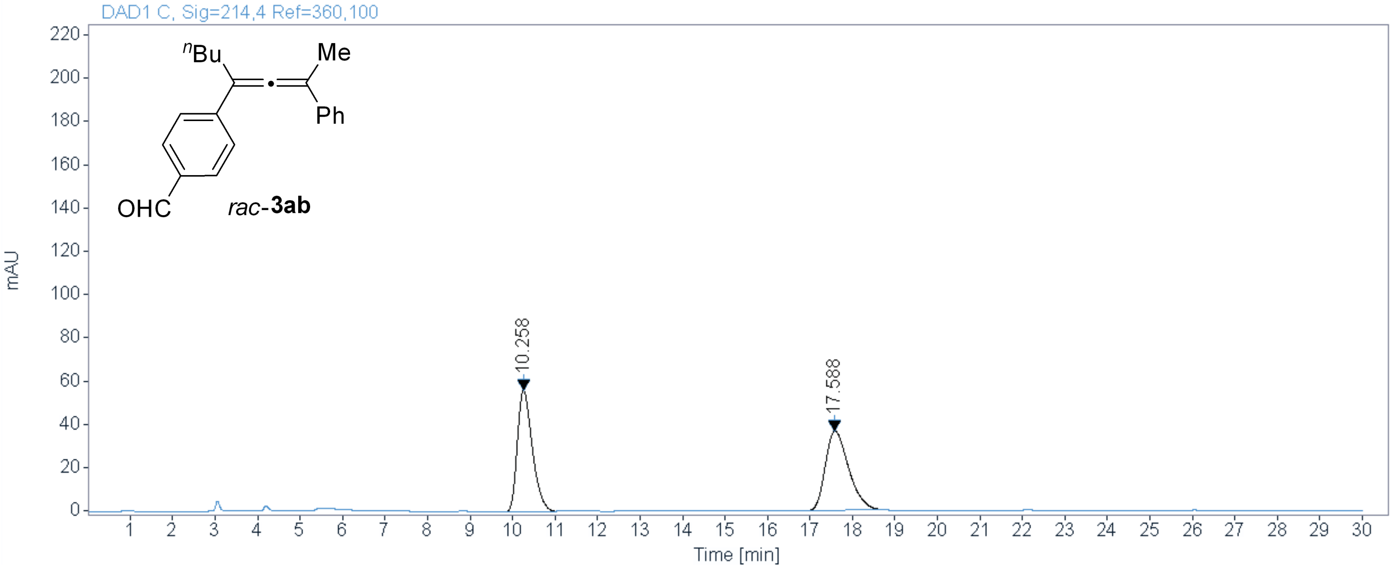

Signal: DAD1 C, Sig=214,4 Ref=360,100

RT [min]	Width [min]	Height	Area	Area%
10.258	0.3781	56.1389	1376.5123	49.4694
17.588	0.6407	36.5784	1406.0403	50.5306
		Sum	2782.5526	100.0000

Supplementary Figure 300. HPLC spectrum for *rac*-**3ab**

4. Supplementary references

1. Zhang, W.; Ma, S. *Chem. Commun.* **2018**, *54*, 6064.

2. Qin, A.; Qian, H.; Chen, Q.; Ma, S. *Chin. J. Chem.* **2020**, *38*, 372.

3. Wang, W.; Qian, H.; Ma, S. *Chin. J. Chem.* **2020**, *38*, 331-345.

4. Liu, N.; Zhi, Y.; Yao, J.; Xing, J.; Lu, T.; Dou, X. *Adv. Synth. Catal.* **2018**, *360*, 642-646.

5. Lowe, G. *J. Chem. Soc. Chem. Commun.* **1965**, 411-413.

6. Brewster, J. H. *Top. Stereochem.* **1967**, *2*, 1-72.

7. Ueda, T.; Tanaka, K.; Ichibakase, T.; Orito, Y.; Nakajima, M. *Tetrahedron*, **2010**, *66*, 7726-7731.

8. Kotani, S.; Kukita, K.; Tanaka, K.; Ichibakase, T.; Nakajima, M. *J. Org. Chem.*, **2014**, 79, 4817-4825.

9. Wang, J.; Zhang, W.; Wu, P.; Huang, C.; Zheng, Y.; Zheng, W.-F.; Qian, H.; Ma, S. *Org. Chem. Front.* **2020**, *7*, 3907-3911.

10. Wu, S.; Huang, X.; Wu, W.; Li, P.; Fu, C.; Ma, S. *Nat. Commun.* **2015**, *6*, 7946-7954.

11. Wu, S.; Huang, X.; Fu, C.; Ma, S. *Org. Chem. Front.* **2017**, *4*, 2002-2007.

12. Xiao, Q.; Tian, L.; Tan, R.; Xia, Y.; Qiu, D.; Zhang, Y.; Wang, J. *Org. Lett.* **2012**, *14*, 4230-4233.

13. Wen, X.; Shi, X.; Qiao, X.; Wu, Z.; Bai, G. *Chem. Commun.* **2017**, *53*, 5372-5375.

14. Huang, X.; Cao, T.; Han, Y.; Jiang, X. Lin, W.; Zhang, J.; Ma, S. *Chem. Commun.* **2015**, *51*, 6956.

15. Liu, J.; Xie, X.; Ma, S. *Synthesis* **2012**, *44*, 1569.

16. Miyaura, N.; Yanagi, T.; Suzuki, A. *Synth. Commun.* **1981**, *11*, 513.

17. Frisch, M. J.; Trucks, G. W.; Schlegel, H. B.; Scuseria, G. E.; Robb, M. A.; Cheeseman, J. R.; Scalmani, G.; Barone, V.; Mennucci, B.; Petersson, G. A.; Nakatsuji, H.; Caricato, M.; Li, X.; Hratchian, H. P.; Izmaylov, A. F.; Bloino, J.; Zheng, G.; Sonnenberg, J. L.; Hada, M.; Ehara, M.; Toyota, K.; Fukuda, R.; Hasegawa, J.; Ishida, M.; Nakajima, T.; Honda, Y.; Kitao, O.; Nakai, H.; Vreven, T.; Montgomery, J. A., Jr.; J. E, P.; Ogliaro, F.; Bearpark, M.; Heyd, J. J.; Brothers, E.; Kudin, K. N.; Staroverov,

V. N.; Keith, T.; Kobayashi, R.; Normand, J.; Raghavachari, K.; Rendell, A.; Burant, J. C.; Iyengar, S. S.; Tomasi, J.; Cossi, M.; Rega, N.; Millam, J. M.; Klene, M.; Knox, J. E.; Cross, J. B.; Bakken, V.; Adamo, C.; Jaramillo, J.; Gomperts, R.; Stratmann, R. E.; Yazyev, O.; Austin, A. J.; Cammi, R.; Pomelli, C.; Ochterski, J. W.; Martin, R. L.; Morokuma, K.; Zakrzewski, V. G.; Voth, G. A.; Salvador, P.; Dannenberg, J. J.; Dapprich, S.; Daniels, A. D.; Farkas, O.; Foresman, J. B.; Ortiz, J. V.; Cioslowski, J.; Fox, D. J. Gaussian 09, Revision D.01, Gaussian, Inc., Wallingford, CT, **2009**.

18. Hariharan, P. C.; Pople, J. A., The influence of polarization functions on molecular orbital hydrogenation energies. *Theor. Chim. Acta* **1973**, *28*, 213.

19. (a) Grimme, S.; Antony, J.; Ehrlich, S.; Krieg, H., A consistent and accurate ab initio parametrization of density functional dispersion correction (DFT-D) for the 94 elements H-Pu. *J. Chem. Phys.* **2010**, *132*, 154104; (b) Grimme, S.; Ehrlich, S.; Goerigk, L., Effect of the damping function in dispersion corrected density functional theory. *J. Comput. Chem.* **2011**, 32, 1456.

20. (a) P. J. Hay, W. R. Wadt, Ab initio effective core potentials for molecular calculations. Potentials for the transition metal atoms Sc to Hg. *J. Chem. Phys.* **1985**, *82*, 270; (b) W. R. Wadt, P. J. Hay, Ab initio effective core potentials for molecular calculations. Potentials for main group elements Na to Bi. *J. Chem. Phys.* **1985**, *82*, 284; (c) P. J. Hay, W. R. Wadt, Ab initio effective core potentials for molecular calculations. Potentials for K to Au including the outermost core orbitals. *J. Chem. Phys.* **1985**, *82*, 299.

21. (a) P. J. Hay and W. R. Wadt, *J. Chem. Phys.*, **1985**, *82*, 270; (b) W. R. Wadt and P. J. Hay, *J. Chem. Phys.*, **1985**, *82*, 284; (c) P. J. Hay and W. R. Wadt, *J. Chem. Phys.*, **1985**, *82*, 299.

22. (a) K. Fukui, Formulation of the reaction coordinate. *J. Phys. Chem.* **74**, 4161-4163 (1970). (b) C. Gonzalez, H. B. Schlegel, An improved algorithm for reaction path following. *J. Chem. Phys.* **90**, 2154-2161 (1989). (c) C. Gonzalez, H. B. Schlegel, Reaction path following in mass-weighted internal coordinates. *J. Phys. Chem.* **94**, 5523-5527 (1990).

23. (a) Y. Zhao, D. G. Truhlar, Density functionals with broad applicability in chemistry.

Acc. Chem. Res. **41**, 157-167 (2008). (b) Y. Zhao, D. G. Truhlar, The M06 suite of density functionals for main group thermochemistry, thermochemical kinetics, noncovalent interactions, excited states, and transition elements: two new functionals and systematic testing of four M06-class functionals and 12 other functionals. *Theor. Chem. Acc.* **120**, 215-241 (2008).

24. D. Andrae, U. Häußermann, M. Dolg, H. Stoll, H. H. Preuß, Energy-adjusted *ab initio* pseudopotentials for the second and third row transition elements. *Theor. Chim. Acta*, 1990, **77**, 123-141.

25. G. Scalmani, M. J. Frisch, Continuous surface charge polarizable continuum models of solvation. I. General formalism. *J. Chem. Phys.* **2010**, *132*, 114110.
