# Supplementary material for: Stereoselectivity control in Rh-catalyzed β-OH elimination for chiral allene formation
Source: Nat Commun. 2023 Nov 16;14:7399. doi: 10.1038/s41467-023-42660-1 (PMC10651921; doi:10.1038/s41467-023-42660-1)
Supplement: Supplementary file 2 — Description of Additional Supplementary Files [file 41467_2023_42660_MOESM2_ESM.pdf]

File Name: **Supplementary Data 1**

Description: Cartesian coordinates for the optimized structures
